# Development of a yeast cell surface display method using the SpyTag/SpyCatcher system

**DOI:** 10.1038/s41598-021-90593-w

**Published:** 2021-05-26

**Authors:** Kaho Kajiwara, Wataru Aoki, Naoki Koike, Mitsuyoshi Ueda

**Affiliations:** 1 Division of Applied Life Sciences, Graduate School of Agriculture, Kyoto University, Sakyo-ku, Kyoto, 606-8502 Japan; 2 JST, CREST, 7 Goban-cho, Chiyoda-ku, Tokyo, 102-0076 Japan; 3 TechnoPro, Inc. TechnoPro R&D, Company, Tokyo, Japan

**Keywords:** Protein design, High-throughput screening

## Abstract

Yeast cell surface display (YSD) has been used to engineer various proteins, including antibodies. Directed evolution, which subjects a gene to iterative rounds of mutagenesis, selection and amplification, is useful for protein engineering. In vivo continuous mutagenesis, which continuously diversifies target genes in the host cell, is a promising tool for accelerating directed evolution. However, combining in vivo continuous evolution and YSD is difficult because mutations in the gene encoding the anchor proteins may inhibit the display of target proteins on the cell surface. In this study, we have developed a modified YSD method that utilises SpyTag/SpyCatcher-based in vivo protein ligation. A nanobody fused with a SpyTag of 16 amino acids and an anchor protein fused with a SpyCatcher of 113 amino acids are encoded by separate gene cassettes and then assembled via isopeptide bond formation. This system achieved a high display efficiency of more than 90%, no intercellular protein ligation events, and the enrichment of target cells by cell sorting. These results suggested that our system demonstrates comparable performance with conventional YSD methods; therefore, it can be an appropriate platform to be integrated with in vivo continuous evolution.

## Introduction

**Figure 1:**
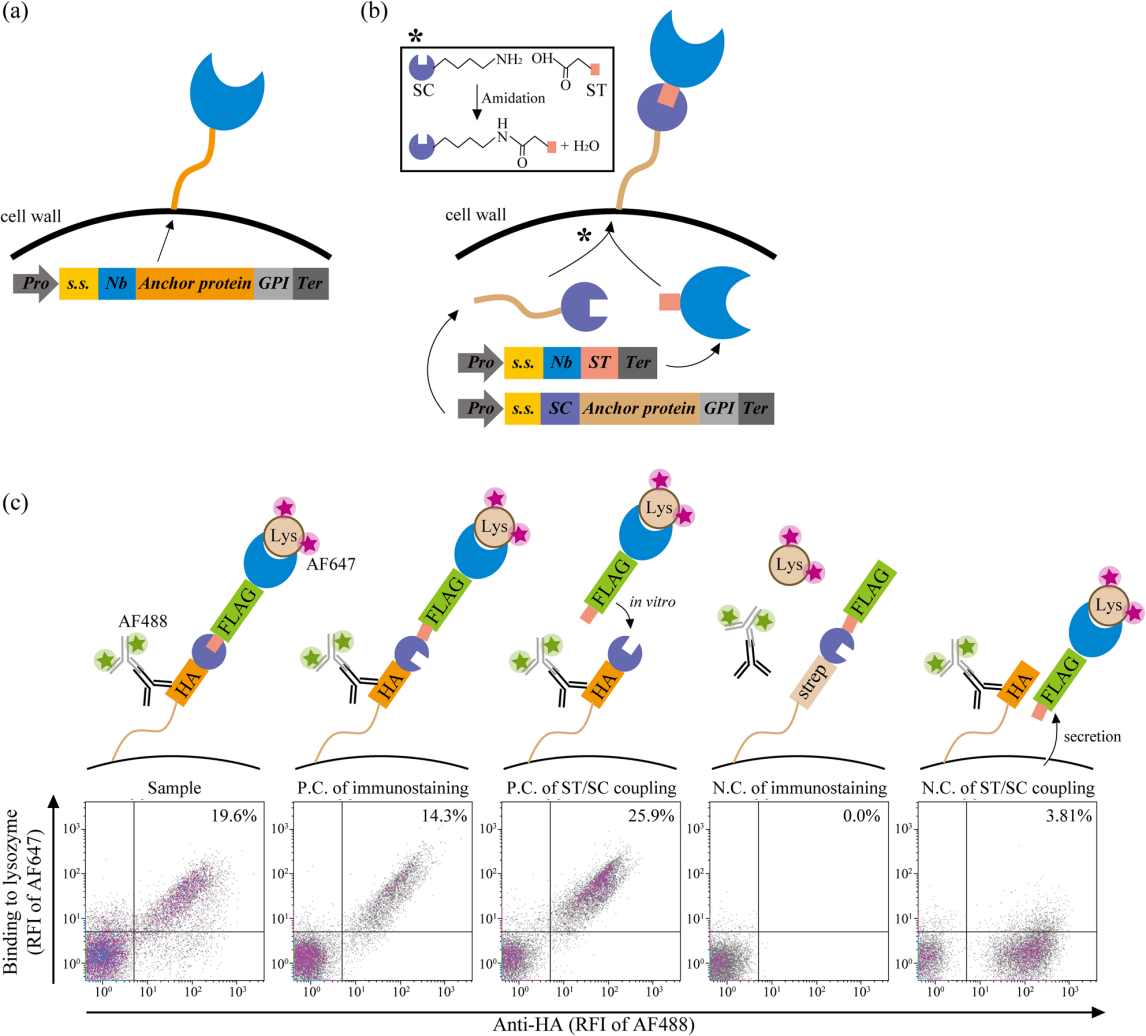
Yeast cell surface display of the nanobodies generated by SpyTag/SpyCatcher-based protein ligation. The schematic of **(a)** a conventional and **(b)** the SpyTag/SpyCatcher-based yeast cell surface display. The asterisk denotes the intracellular isopeptide bond formation between a SpyCatcher and a SpyTag. The reactive Lys31 in SpyCatcher and Asp117 in SpyTag are shown in the inset. **(c)** The confirmation of the cell surface display of anti-lysozyme nanobodies based on the in vivo and in vitro SpyTag/SpyCatcher-based protein ligation. Details of plasmids construction for each strain are shown in Supplementary Information Fig. S2. Each sample was stained with mouse anti-HA tag antibody, AF488-conjugated anti-mouse antibody and AF647-labelled lysozyme. The ratio of yeast cells is shown in the upper right (UR) corner of each graph. The data shown are representative of two independent experiments. *Pro* promoter; *s.s.* secretion signal; *Nb* nanobody; *GPI* glycosylphosphatidylinositol attachment signal; *Ter* terminator; *ST* SpyTag; *SC* SpyCatcher; *N.C.* negative control; *Lys* hen egg-white lysozyme; *RFI* relative fluorescence intensity. This figure was created using Illustrator CS2 (https://www.adobe.com/).

Yeast cell surface display (YSD) is a versatile technology for protein engineering^1–6^. In YSD, a protein of interest (POI) is genetically fused to a cell surface anchor protein and covalently tethered to the cell wall via a glycosylphosphatidylinositol (GPI) attachment signal (Fig. 1a). The activities of the displayed proteins can be evaluated rapidly and quantitatively using flow cytometry^7^. Furthermore, YSD has the advantages of eukaryotic folding machinery and safe pathogen-free protein production^8,9,13^.

Directed evolution is widely used to improve the properties of a POI^10–14^. Conventional directed evolution is a time-consuming process involving labor-intensitive rounds of in vitro gene diversification, the transformation of randomised genes into host cells, the selection of improved genes and the extraction of improved gene sequences from host cells (Supplementary Information Fig. S1).

Conversely, in vivo continuous evolution, in which target genes are diversified within an organism, has garnered much attention as a way to accelerate protein engineering^15–21^. In this strategy, a mutagenic enzyme which specifically recognizes a target gene continuously diversifies it by simply culturing the host cells (Supplementary Information Fig. S1). For example, one of the in vivo continuous evolution methods uses a cytidine deaminase fused to a T7 RNA polymerase introduces mutations into only a target gene under T7 promoter^18^. This method has achieved an on-target mutation rate in the order of 10^−3^ bp^−1^ and has succeeded in generating mitogen-activated protein kinase 1 that is resistant to both selumetinib and trametinib in just 2 weeks^18^.

Although in vivo continuous evolution is useful, it requires some modification for its integration with YSD because mutagenesis can also introduce detrimental nonsense and missense mutations into the gene encoding the anchor protein, thus inhibiting the cell surface display of the POI. Therefore, a method to display the produced POI in *trans* to the anchor proteins is needed for integrating YSD and in vivo continuous evolution.

Protein/peptide ligation may enable the integration of YSD and in vivo continuous evolution. In vivo protein or peptide ligation has been achieved using SpyTag/SpyCatcher-related technologies^22,23^. The SpyTag/SpyCatcher system was developed by splitting a CnaB2 domain of *Streptococcus pyogenes* fibronectin-binding protein FbaB harboring an internal isopeptide bond^24^. SpyCatcher contains a reactive Lys and catalytic Glu while SpyTag includes a reactive Asp, and the isopeptide bond is generated between SpyCatcher and SpyTag to form a covalently bound complex. The SpyTag/SpyCatcher system has been used to immobilize foreign proteins onto nanoparticles^25–27^ and bacterial outer membrane vesicles^25–30^. Recently, cell surface display of enzymes in *E. coli* using SpyTag/SpyCatcher has been reported^31^, but there was no example of displaying fusion proteins produced by post-translational ligation in eukaryotic cells.

Here, we report a modified YSD system utilising in vivo protein ligation. We used nanobodies (VHH fragments) as the proteins to be displayed and 649-stalk, a synthetic anchor protein effective in displaying functional nanobodies, as a scaffold^5,32^. A nanobody and 649-stalk were successfully assembled via post-translational protein ligation and then displayed on the yeast cell surface (Fig. 1b). We were able to achieve a display efficiency of more than 90%. During the process, we observed no intercellular protein ligation events. Furthermore, the target cells could be easily enriched via cell sorting. By integrating YSD with in vivo continuous evolution methods, our platform can be a powerful screening tool for protein engineering.

## Results

### Cell surface display of nanobodies using SpyTag/SpyCatcher-based protein ligation

We utilised the SpyTag/SpyCatcher system to ligate the nanobodies and anchor proteins in yeast. We designed two gene cassettes to fuse the synthetic anchor protein 649-stalk with SpyCatcher (113 amino acids) and a nanobody with SpyTag (16 amino acids). The two fusion proteins can be bound covalently in yeast via the post-translational isopeptide formation between SpyTag and SpyCatcher. The final fusion product is expected to be transported via the secretion pathway and anchored to the cell wall via a GPI attachment signal (Fig. 1b).

We introduced the constructed gene cassettes in yeast cells to test our scheme’s feasibility (Supplementary Information Fig. S2). We initially used a multicopy pRS425 vector to produce the SpyCatcher-anchor protein and a multicopy pRS423 vector for the nanobody-SpyTag. Flow cytometry analysis demonstrated a successful display of nanobodies on the yeast cell surface when the yeast cells produced both the SpyCatcher-anchor protein and Lys Nb-SpyTag (Fig. 1c). Protein ligation also occurred when we added purified Lys Nb-SpyTag to yeast cells producing only the SpyCatcher-anchor protein (Fig. 1c, Supplementary Information Fig. S2 and Fig. S3). These results indicated that SpyTag/SpyCatcher-based protein ligation worked well to display the separately produced and then ligated nanobodies and anchor proteins.

### Improvement of display efficiency of SpyTag/SpyCatcher-mediated YSD

**Figure 2:**
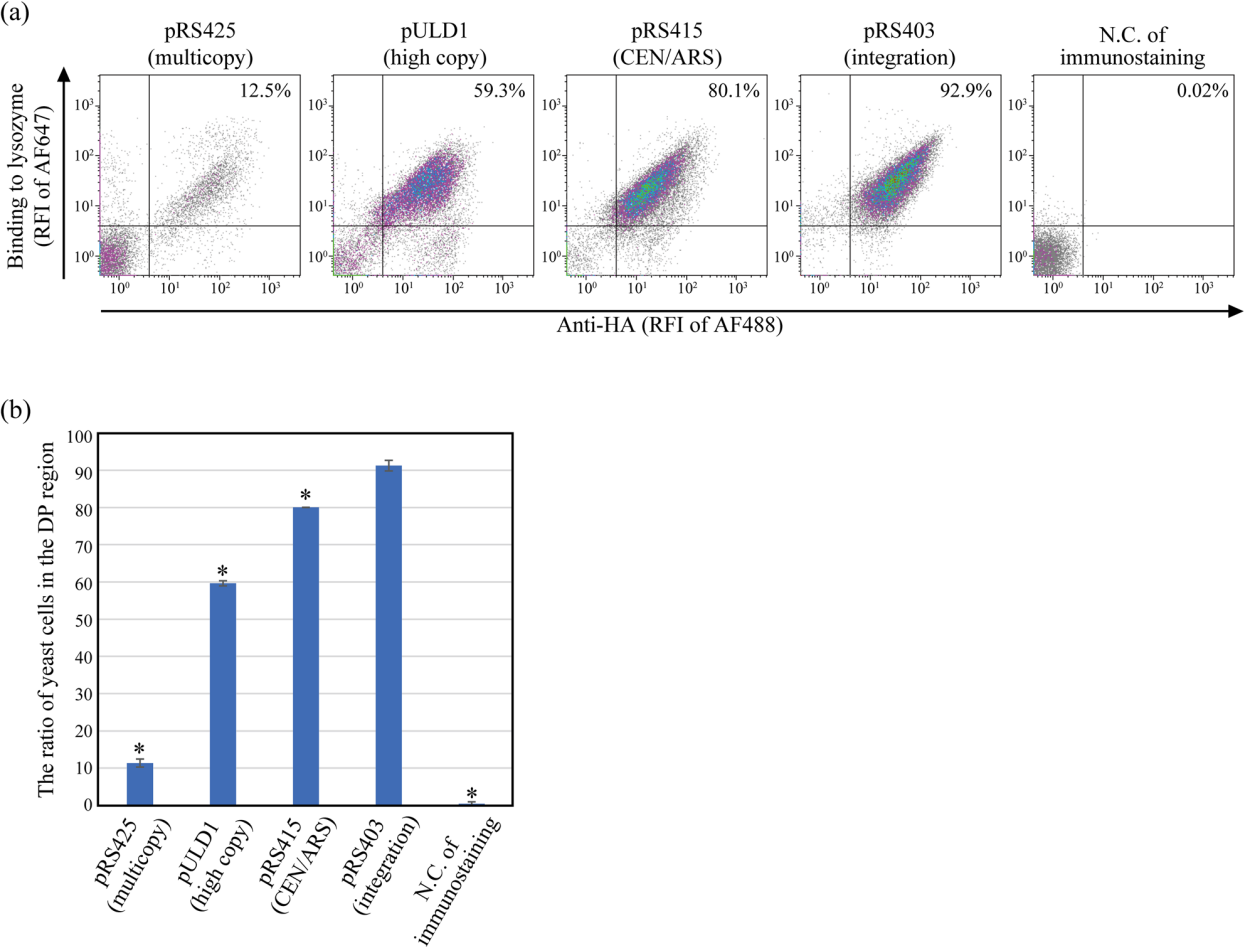
Improving of display efficiency by selecting an optimal vector to produce the SpyCatcher-anchor protein. The vector for the negative control yeast, used to indicate nonspecific absorption during immunostaining and flow cytometry, did not contain a nanobody-encoding sequence nor an HA tag; instead, it contained a FLAG tag. Each sample was stained with mouse anti-HA tag antibody, AF488-conjugated anti-mouse antibody and AF647-labelled lysozyme. **(a)** Density plots of the flow cytometry analysis. The ratio of yeast cells is shown in the upper right (UR) corner of each graph. The data shown are representative of three independent experiments. **(b)** Comparison of the double-positive (DP) regions represent yeast cells with strong AF488 and AF647 signals. The data from three independent experiments were represented as means ± standard deviations. Dunnett’s test was used to compare the display efficiency of the pRS403 strain to other strains (*p < 0.005). This figure was created using Illustrator CS2 (https://www.adobe.com/).

We sought to improve the display efficiency of the SpyCatcher-anchor protein because the number of yeast cells displaying the SpyCatcher-anchor protein was very low in our initial experiment (Fig. 1c). We designed a follow-up experiment to identify an optimal backbone vector to produce the SpyCatcher–anchor protein. The multicopy pRS425 vector, a high copy pULD1 vector^33^, a centromeric pRS415 vector and an integrative pRS403 vector were compared. We changed only backbone sequences, and used the same gene cassette as the initial construct (Supplementary Information Fig. S2). Also, the initial multicopy vector used to produce nanobody-SpyTag was changed to centromeric vector. This is because too many copies of nanobody-SpyTag vectors in a cell could make it challenging to identify the strong binder sequences in future studies with in vivo continuous evolution. We investigated the effect of the backbone sequences on the display efficiency and the expression level (Fig. 2 and Supplementary Information Table S1). The pRS403 strain showed the highest ratio of double-positive yeast cells; thus, it had the highest display efficiency of functional nanobodies (Fig. 2). This is probably because the pRS403 vector was transformed into yeast by stable single-copy integration^34,35^. The expression level of the integrative pRS403 vector was lower than that of the multi-copy pRS421 vector and the high-copy pULD1 vector, indicating that high expression levels did not improve display efficiencies (Supplementary Information Table S1)^35^. We used the pRS403 vector to produce the SpyCatcher-anchor protein and the p415 vector to produce nanobody-SpyTag in the subsequent experiments because the display efficiency is more important than the expression level for library screening.

**Figure 3:**
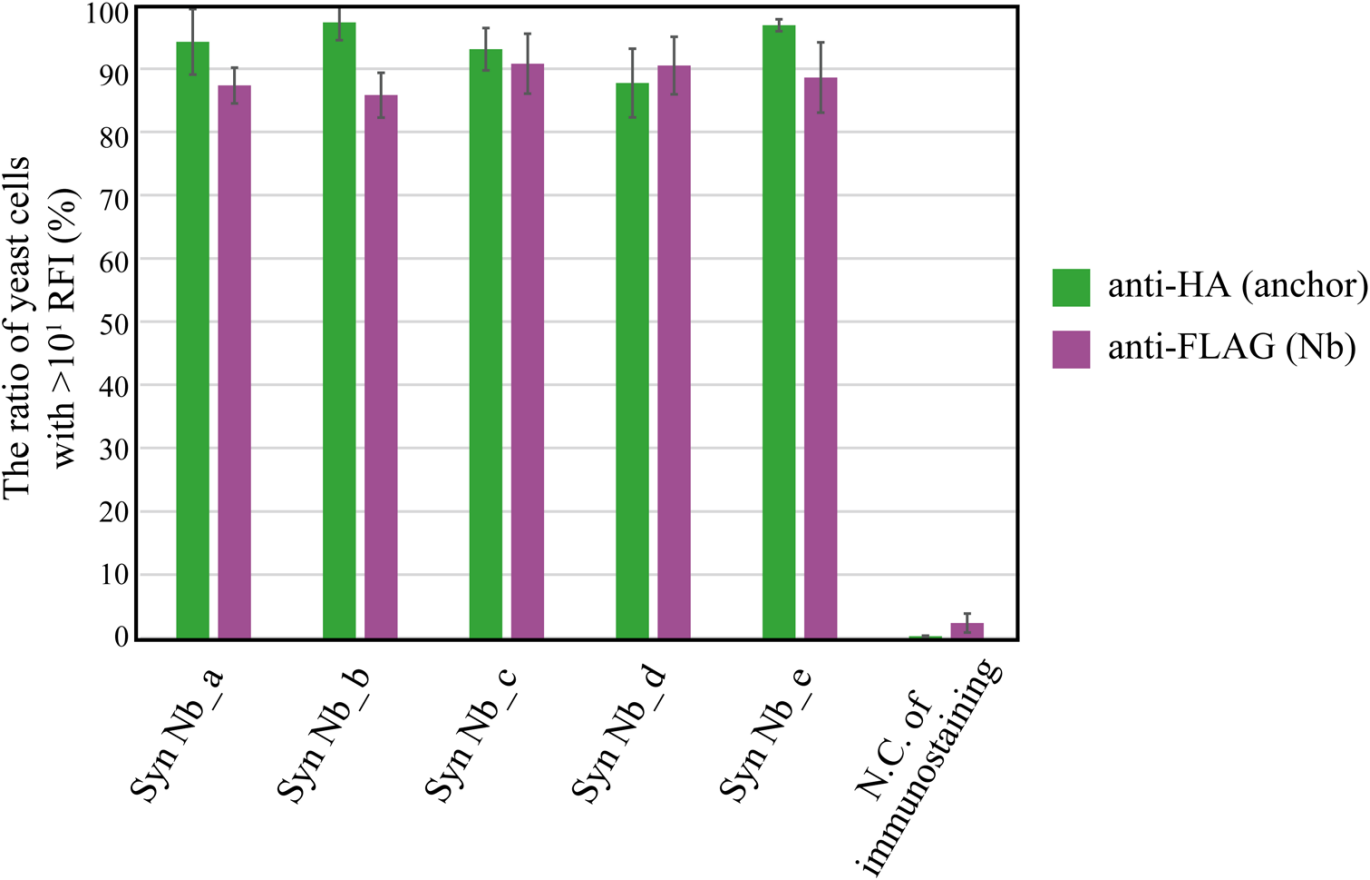
Yeast cell surface display of various nanobodies derived from a synthetic nanobody library. Five nanobody-encoding nucleotide sequences were isolated from the synthetic nanobody library (see “Methods”) and cloned into pRS415 with a FLAG tag and a SpyTag. The multiple alignments of the five nanobodies were also performed (Supplementary Information Fig. S4). The plasmids were transformed into the BY4741 strain harbouring a plasmid encoding the 649-stalk anchor protein fused with an HA tag. The negative control strain, used to indicate the nonspecific absorption during immunostaining for flow cytometry, did not contain an HA nor a FLAG tag. Each sample was stained using mouse anti-HA tag antibody and AF488-conjugated anti-mouse antibody, or mouse anti-FLAG tag antibody and AF647-conjugated anti-mouse antibody. The data of three independent experiments were represented as means ± standard deviations. *Syn Nb* nanobody derived from the synthetic library, *N.C.* negative control, *RFI* relative fluorescence intensity. This figure was created using Illustrator CS2 (https://www.adobe.com/).

Next, we investigated the universal applicability of our YSD method using the SpyTag/SpyCatcher system by examining whether we could display various nanobodies randomly isolated from a synthetic library. We designed a synthetic nanobody library based on a consensus framework derived from an anti-β-lactamase nanobody (cAbBCII10)^36^. The framework was combined with randomised complementary determining regions (CDRs) that recapitulate amino acid position-specific variation in natural llama immunological repertoires^5^. Using the Sanger sequencing, we confirmed that the five nanobody clones isolated from the synthetic library had randomised CDRs (Supplementary Information Fig. S4 and Fig. S5). These nanobodies were used to investigate whether our platform can display randomized nanobodies and to show the feasibility of de novo nanobody screening in future studies. We introduced the vectors encoding each synthetic nanobody into *Saccharomyces cerevisiae*. We found that both HA tag fused to the anchor protein and FLAG tag fused to the synthetic nanobodies were detected with high display efficiencies (Fig. 3). These results confirmed the universal applicability of our system for displaying various nanobodies on the yeast cell surface.

### Confirmation of intracellular SpyTag/SpyCatcher-based protein ligation

**Figure 4:**
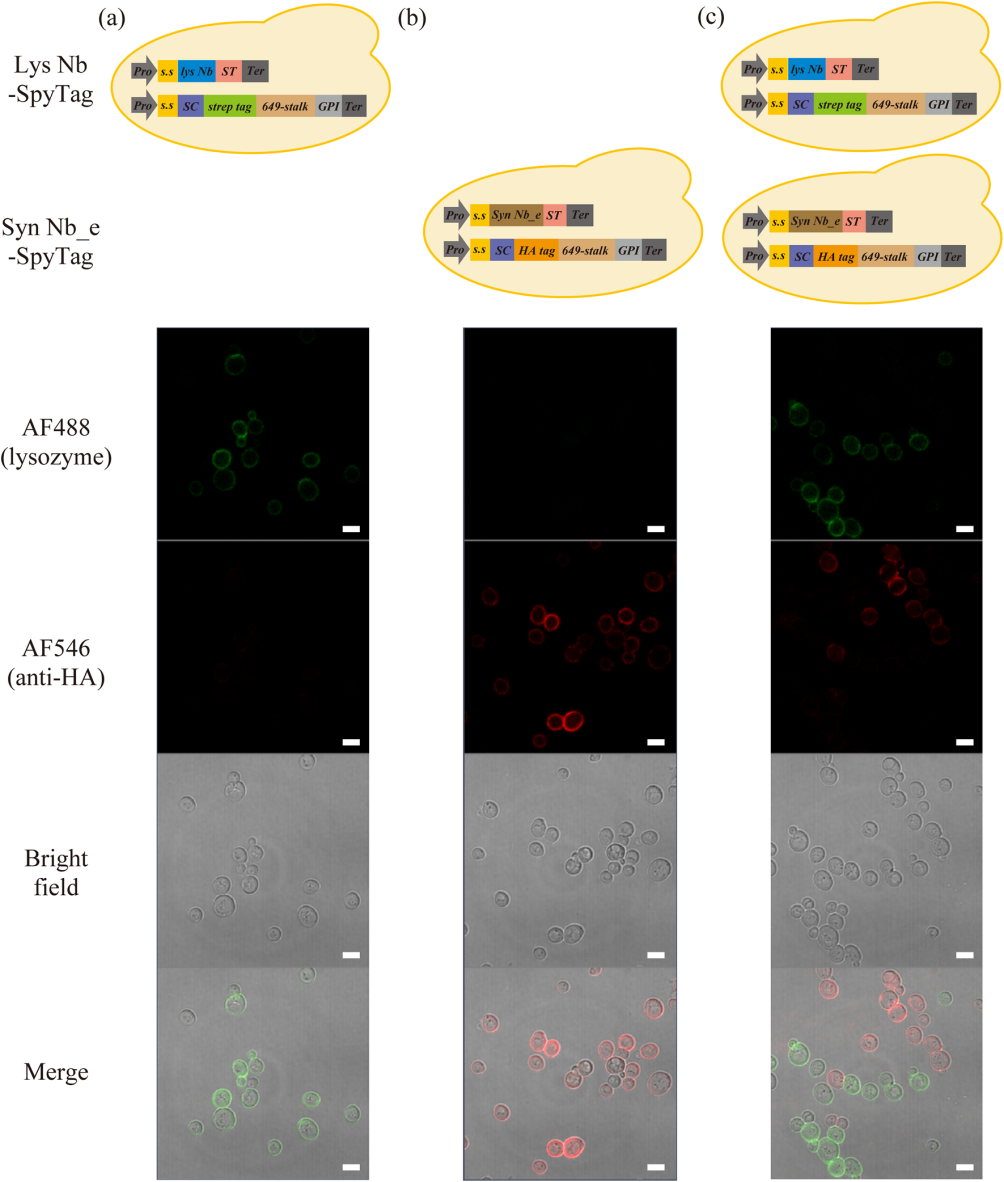
Detection of potential intercellular protein ligation events via microscopy. Yeast cells producing anti-lysozyme nanobody (Lys Nb-SpyTag) **(a)** or nanobody_e from the synthetic library (Syn Nb_e-SpyTag) **(b)** were co-cultured **(c)** to detect for intracellular or intercellular protein ligation between the nanobody-SpyTag and the anchor-SpyCatcher fusion proteins. Each sample was stained with the mouse anti-HA tag antibody, AF546-conjugated anti-mouse antibody and AF488-labelled lysozyme. No AF488- and AF546- both positive cells were detected in co-cultured sample. *Pro* promoter; *s.s* secretion signal; *SC* SpyCatcher; *649-stalk* a synthetic anchor protein consisting of 649 amino acids; *ST* SpyTag; *GPI* glycosylphosphatidylinositol attachment signal; *Ter* terminator. Scale bars, 5 µm. This figure was created using Illustrator CS2 (https://www.adobe.com/).

During a screening with our system, intercellular protein ligation events, in which the nanobody–SpyTag secreted from a yeast cell is ligated to the SpyCatcher–anchor protein of neighbouring cells, will be unfavourable. We searched for potential intercellular protein ligation using microscopy. We prepared two yeast strains which produced different nanobody-SpyTag proteins (Lys Nb-SpyTag or Syn Nb_e-SpyTag) and SpyCatcher-anchor proteins (strep-tagged or HA-tagged). First, we cultured the two yeast strains separately and stained them with AF488-labelled lysozyme and anti-HA-AF546 antibody. The yeast strain producing Lys Nb-SpyTag or Syn Nb_e-SpyTag was successfully labelled by AF488-labelled lysozyme or AF546-labelled anti-HA antibody, respectively (Fig. 4ab). Next, we co-cultured the two strains in one-pot. The co-cultured cells producing Lys Nb-SpyTag or Syn Nb_e-SpyTag were positive for either AF488 or AF546; there were no double-positive cells that showed fluorescence signals of both AF488 and AF546 (Fig. 4c). These results indicated that the isopeptide bond formation between SpyTag and SpyCatcher mostly occurred intracellularly.

### Enrichment of target binders by cell sorting

**Figure 5:**
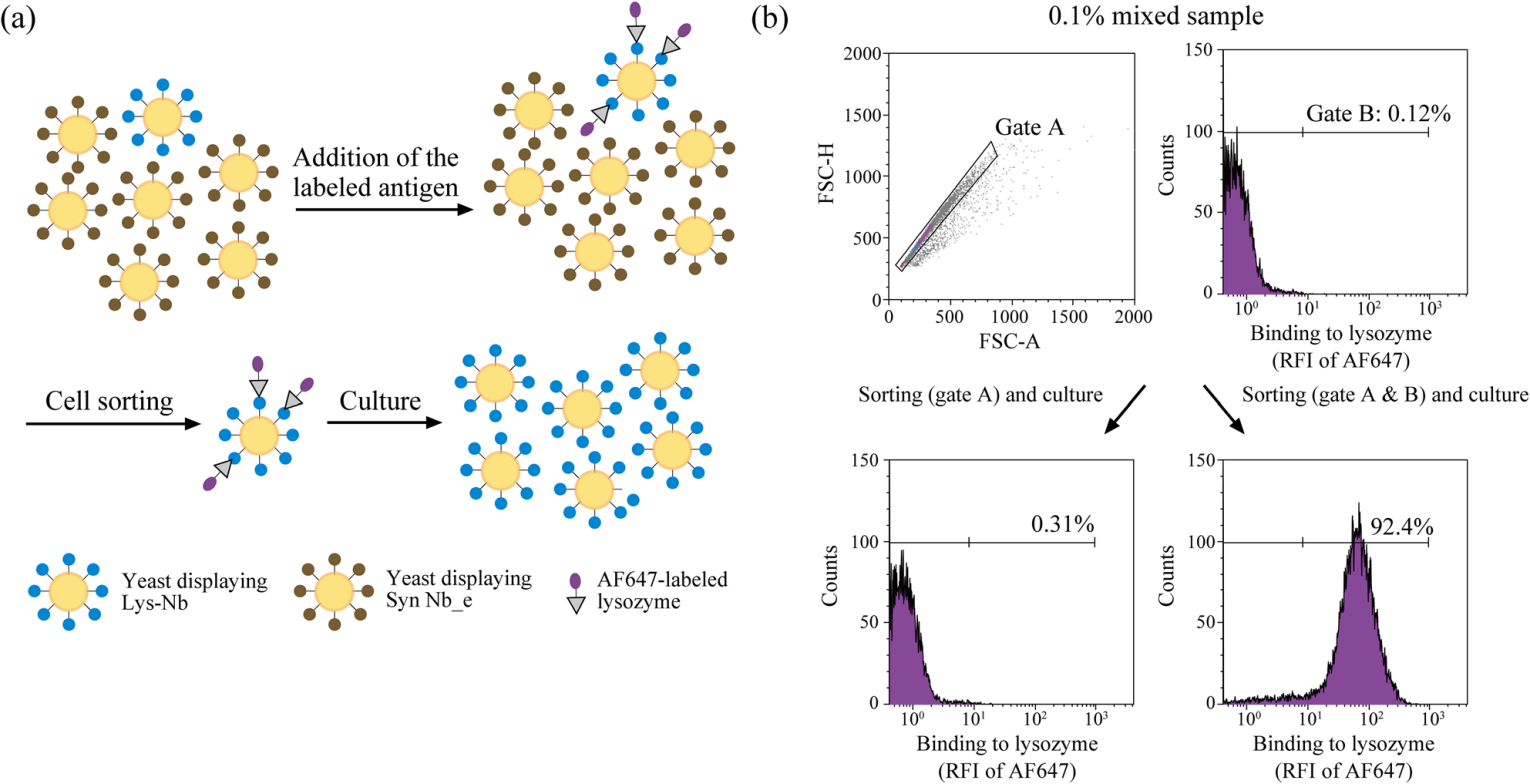
Target cell enrichment by cell sorting. **(a)** The schematic of nanobody selection. Antigens (grey triangles) are labelled with AF647 (purple circle). Yeast cells displaying nanobodies against the antigen were detected by flow cytometry and isolated via cell sorting. **(b)** Yeast cells displaying the anti-lysozyme nanobody (Lys Nb) were enriched from the 0.1% mixed culture. Yeast strains producing Lys Nb or nanobody_e from the synthetic library (Syn Nb_e) were co-cultured at the ratio of 1:999. The sample was incubated with AF647-labelled lysozyme and analysed by flow cytometry. AF647-positive (gate B) were sorted and recovered in a selection medium for 72 h. As a control, the singlet yeast cells (gate A) were also sorted and recovered in the same procedure. The recovered yeast cells were stained again with AF647-labelled lysozyme and analysed by flow cytometry. The ratio of yeast cells in the right regions is shown in each graph. The data shown were represented by three independent experiments. *RFI* relative fluorescence intensity. This figure was created using Illustrator CS2 (https://www.adobe.com/).

Cell sorting is a powerful strategy to isolate antigen-specific nanobodies from a library^37^. We further tested the feasibility of SpyTag/SpyCatcher-mediated YSD as a screening platform by enriching yeast cells producing Lys Nb-SpyTag. Lys Nb-SpyTag and Syn Nb_e-SpyTag were co-cultured from 1:999 ratio and the 0.1% mixed sample was generated. The 0.1% mixed sample was incubated with AF647-labelled lysozyme and analysed with flow cytometry (Fig. 5a). The AF647-positive singlet cells (gates A and B) were sorted and recovered. The singlet cells (gate A) were also sorted as a control. These recovered samples were cultured and then incubated again with AF647-labelled lysozyme. Consequently, the percentage of AF647-positive cells increased from 0.12% to 92.4% during a single enrichment process against the AF647-positive singlet cells (Fig. 5b).

## Discussion

Here, we developed a SpyTag/SpyCatcher-based nanobody display system in which the nanobodies and anchor proteins produced by different gene cassettes were ligated in yeast cells. Utilising this system, more than 90% of the yeast cells successfully displayed functional nanobodies on their cell surface (Fig. 2); the display efficiency was comparable with that of conventional yeast cell surface display methods^32^. The high display efficiency was achieved using an integrative pRS403 vector for the stable production of the anchor proteins^34,35^ and the improved SpyTag003/SpyCatcher003 system, which facilitated the formation of an irreversible covalent bond at a rate approaching the diffusion limit^23^.

Since the anchor proteins and passengers, for example, nanobodies, are produced separately in our system, the selective, directed evolution of the passengers can be achieved when combined with the in vivo continuous evolution of target genes in future studies. Recently, Liu et al. conducted a similar study that utilised Aga1 as an anchor protein and Aga2-fused nanobodies as passengers^20^. SpyTag, comprising only 16 amino acids, is smaller than Aga2 of 87 amino acids; hence, our system may be less likely to generate detrimental nonsense and missense mutations that inhibit the cell surface display of nanobodies.

Our system may be useful not only as a screening platform but also as a display platform for complex proteins that are difficult to display using conventional methods. For example, a YSD of full-length IgG with secretion and capture strategies has been reported; however, the system requires complex processes, such as the addition of purified streptavidin-ZZ or chemical modifications of yeast cells^38–40^. Since our system uses a simple secretion and capture strategy without the addition of any external components or chemical modifications of yeast cells, it may become an optimal strategy for cell surface display of complex proteins.

In summary, we have successfully developed a SpyTag/SpyCatcher-mediated yeast cell surface display system with a display efficiency of more than 90% and no observed intercellular protein ligation events. Furthermore, the target cells can be easily enriched by cell sorting. Therefore, our system will be a powerful tool for screening libraries constructed with in vivo continuous evolution.

## Methods

### Reagents

Supplementary Information Table S2 describes all antibodies used in this study and the dilution ratio.

### Construction of plasmids

DNA fragments encoding the improved α-factor secretion signal^41^, anti-hen egg-white lysozyme nanobody cAbLys3^42^, synthetic anchor protein 649-stalk^5^, SrtA pentamutant^43^ and improved SpyCatcher^23^ were synthesised using gBlocks Gene Fragments (Integrated DNA Technology, Coralville, IA, USA). These genes were cloned into backbone vectors using an In-fusion Cloning Kit (Takara Bio USA Inc., Shiga, Japan) and amplified in competent *E. coli* DH5α (*F*^*−*^*Φ80lacZΔM15 Δ(lacZYA-argF) U169 endA1 recA1 hsdR17(r *^*−*^*K, m* ^+^*K) deoR supE44 thi-1 λ*^*−*^*gyrA96 relA1*). The transformed *E. coli* bacteria were cultured in a Luria–Bertani medium (1% [w/v] tryptone, 0.5% [w/v] yeast extract and 1% [w/v] sodium chloride) containing 100 µg/mL ampicillin. The full sequences of the plasmids used in this study are included in Supplementary Information.

### Construction of yeast strains for YSD

The *S. cerevisiae* strain BY4741 (*MATa, his3Δ1, leu2Δ0, met15Δ0, ura3Δ0*) was used as the host for the cell surface display. Yeast cells were transformed with the constructed plasmids using a Frozen EZ Yeast Transformation II Kit (Zymo Research, Irvine, CA, USA). The transformants were screened on a synthetic defined (SD) solid medium (0.67% [w/v] yeast nitrogen base without amino acids, 2% [w/v] glucose, 50 mM HEPES adjusted to pH 7.0 with 1 N NaOH and 2% [w/v] agar) with appropriate amino acids and a nucleobase (0.002% [w/v] l-histidine, 0.012% [w/v] l-leucine, 0.002% [w/v] l-methionine and 0.002% [w/v] uracil). The selected colonies were precultured in liquid SD media at 30 °C and 250 rpm overnight. Then, the optical density of the cultures was measured at 600 nm, and the primary cultures were started at the OD600 of 0.1.

### Secretary production of nanobody-SpyTag in *Pichia pastoris* and SDS-PAGE

The *Pichia pastoris* strain GS115 (*his4Δ, Mut*^+^) was used as the host for the secretory production of a nanobody–SpyTag fusion protein. The constructed pPIC9K-based plasmid was digested by SacI (Toyobo, Osaka, Japan), purified with a MinElute PCR Purification Kit (QIAGEN, Hilden, Germany) and introduced into *P. pastoris* using a Frozen EZ Yeast Transformation II Kit. The transformants were screened on a minimal dextrose solid medium (1.34% [w/v] yeast nitrogen base without amino acids, 2% [w/v] glucose and 2% [w/v] agar). The selected colonies were cultured in the buffered glycerol-complex (BMGY) medium (1% [w/v] yeast extract, 2% [w/v] peptone, 1% [w/v] glycerol, 0.1 M potassium phosphate buffer pH 6.0, 2.68% [w/v] yeast nitrogen base without amino acids and 400 µg/mL biotin) at 30 °C and 250 rpm for 24 h; the grown cells were transferred to buffered methanol-complex (BMMY) medium (1% [w/v] yeast extract, 2% [w/v] peptone, 0.1 M potassium phosphate buffer pH 6.0, 2.68% [w/v] yeast nitrogen base without amino acids, 400 µg/mL biotin and 0.5% [v/v] methanol) and cultured at 30 °C and 250 rpm for another 24 h. Afterwards, the BMMY culture was centrifuged to collect the supernatant and analyse it with SDS-PAGE. For in vitro isopeptide formation, the supernatants were filtered using 0.5 µm filters and concentrated using Amicon Ultra-15 Centrifugal Filters Ultracel-3K (Merck Millipore, Burlington, MA, USA) at 8000×*g* for 60 min. Then, 10 mL of phosphate-buffered saline (PBS) was added to the Amicon Ultracel-3K unit and centrifuged at 8000×*g* for 60 min. The buffer replacement procedure was repeated twice.

### Generation of a synthetic nanobody library

A synthetic DNA library encoding diversified nanobodies was constructed using two-step overlap-extension PCR (OE-PCR). A custom trimer mix was created to synthesise randomised primers (Glen Research, Sterling, VA, USA). A set of eight primers was synthesised at a concentration of 100 μM (Supplementary Information Table S3). Mixed pools A, B and C containing 2 μM of each primer were prepared. The three mixed pools used different P7_for primers (mixed pools A, B and C used P7a_for, P7b_for and P7c_for, respectively) to create corresponding CDR3 synthetic sequences of 12, 16, or 20 amino acids.

One microlitre of each mixed pool was used in a 50 μL PCR reaction that also included 1 × GC buffer, 200 µM dNTPs, 0.04 µM each primer mix, 1.5 mM MgCl_2_ and 1.0 unit of Phusion DNA Polymerase (New England Biolabs, Ipswich, MA, USA). The OE-PCR reactions were performed for 35 cycles. The full-length nanobody DNA fragments from each pool were purified using a FastGene Gel/PCR Extraction Kit. The purified nanobody DNA fragments and pULD1-based plasmid for surface display (pYSD) were each digested with SpeI (Toyobo) and SfiI (Takara Bio).

The digested nanobody DNA fragments from mixes A, B and C were mixed at a molar ratio of 1:2:1; the mixture would be referred to as the nanobody DNA library hereafter. The nanobody DNA library and the digested backbone plasmid were mixed at an equimolar ratio and ligated using the Ligation high (Toyobo). We transformed the ligated DNA into *E. coli* DH5α using chemical transformation and cultured the transformed *E. coli* in LBA media. After incubation, the plasmids from five colonies were extracted and sequenced using the Sanger sequencing method.

### Immunofluorescence labelling of yeast cells for flow cytometry

Immunofluorescence labelling was performed to detect nanobody display on the yeast cell surface and measure the percentage of the yeast cells displaying nanobodies or an anchor protein, or both. The fluorescence intensity of the labelled yeast cells was evaluated via flow cytometry. The cell density of each sample was measured at OD600. Approximately 4.5 × 10^6^ cells were subjected to immunofluorescence labelling. After centrifugation at 1000×*g* for 5 min, the cells were washed with PBS (pH 7.2), resuspended in PBS containing 1% bovine serum albumin, and incubated for 30 min at room temperature. At corresponding dilutions (Supplementary Information Table S2), primary antibodies were added for incubation at room temperature with gentle shaking on a rotary shaker for 1 h. The cells were then washed with PBS and incubated with secondary antibodies at room temperature with gentle shaking on a rotary shaker for 1.5 h. To evaluate functional display of anti-lysozyme nanobody, Alexa Flour 647 (AF647)-labeled lysozyme was also added with secondary antibodies. Lysozyme was labelled with fluorescence using an Alexa Fluor 647 Microscale Protein Labelling Kit (Invitrogen Corporation, Carlsbad, CA, USA) to produce Alexa Flour 647 (AF647)-labelled lysozyme. During immunofluorescence labelling, the AF647-labelled lysozyme was added at a dilution ratio of 1:500 with the secondary antibodies. Afterwards, the cells were washed with PBS, suspended in PBS and analysed with a flow cytometer (JSAN; Bay Bioscience, Kobe, Japan). The fluorescence of AF647 was detected with an excitation at 640 nm and emission at 661 ± 10 nm, and AF488 was detected with an excitation at 488 nm and emission at 535 ± 23 nm. Then, the fluorescence intensity of 20,000 yeast cells was displayed in a density plot or a histogram. Data were analysed using the Kaluza software (Beckman Coulter, Brea, CA, USA). In the density plot, the ratio of the upper right (UR) corner of the plot, which represented both AF488- and AF647-positive cells, was quantified. In the histogram, the ratio of the right region which represented strong fluorescent intensity cells were quantified.

### Confocal laser scanning fluorescence microscopy to detect potential intercellular protein ligation

In addition to the immunofluorescent labelling described the previous section, Alexa Flour 488 (AF488)-labelled lysozyme was also added with secondary antibodies. The fluorescence labelling of the lysozyme was performed using an Alexa Fluor 488 Microscale Protein Labelling Kit (Invitrogen). During immunofluorescence labelling, the AF488-labelled lysozyme was added at the dilution of 1:100 with the secondary antibodies. The cells were observed by confocal laser scanning fluorescence microscopy (LSM700; Carl Zeiss, Oberkochen, Germany). Fluorescence of AF488 and AF546 was observed using 488 and 561 nm lasers, respectively. The acquired images were processed using the Zen lite software.

### Statistical analysis

In Figs. 2 and 3, the data from three independent experiments were represented as means ± standard deviations. Dunnett’s test was used to calculate p value in Fig. 2.

## Supplementary Information

Supplementary Information.

## Data Availability

All relevant data are within the manuscript and its supplementary information file.
