# Supplementary material for: Development of a yeast cell surface display method using the SpyTag/SpyCatcher system
Source: Sci Rep. 2021 May 26;11:11059. doi: 10.1038/s41598-021-90593-w (PMC8155107; doi:10.1038/s41598-021-90593-w)
Supplement: Supplementary file 1 — Supplementary Information. [file 41598_2021_90593_MOESM1_ESM.pdf]

TITLE

Development of a yeast cell surface display method using the SpyTag/SpyCatcher system

AUTHORS AND AFFILIATIONS

Kaho Kajiwara[1], Wataru Aoki[1,2], Naoki Koike[3], Mitsuyoshi Ueda[1,2*]

[1]Division of Applied Life Sciences, Graduate School of Agriculture, Kyoto University, Sakyo-ku, Kyoto, 606-8502, Japan

[2]JST, CREST, 7 Goban-cho, Chiyoda-ku, Tokyo, 102-0076, Japan

[3]TechnoPro, Inc. TechnoPro R&D, Company

*Correspondence should be addressed to: Mitsuyoshi Ueda

Tel.: +81-75-753-6495; Fax: +81-75-753-6112; E-mail: miueda@kais.kyoto-u.ac.jp

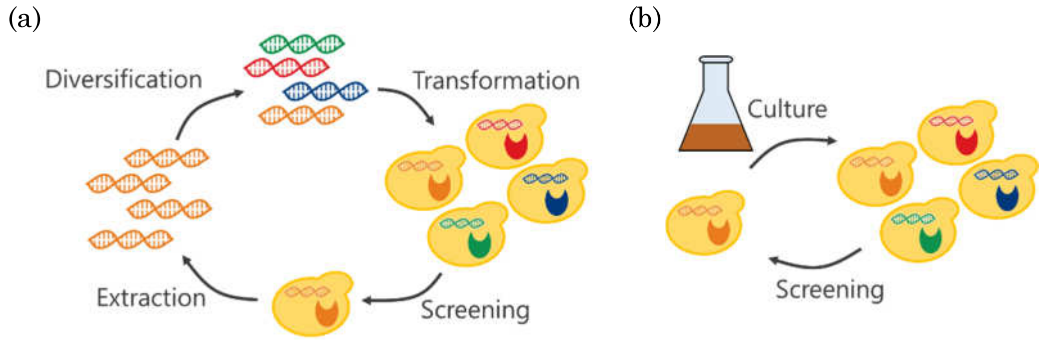

Figure S1 The process of directed evolution using (a) *in vitro* mutagenesis and (b) *in vivo* mutagenesis. Protein of interest can be evolved by subjecting to iterative rounds of (a) or (b). This figure was created using Illustrator CS2 (https://www.adobe.com/).

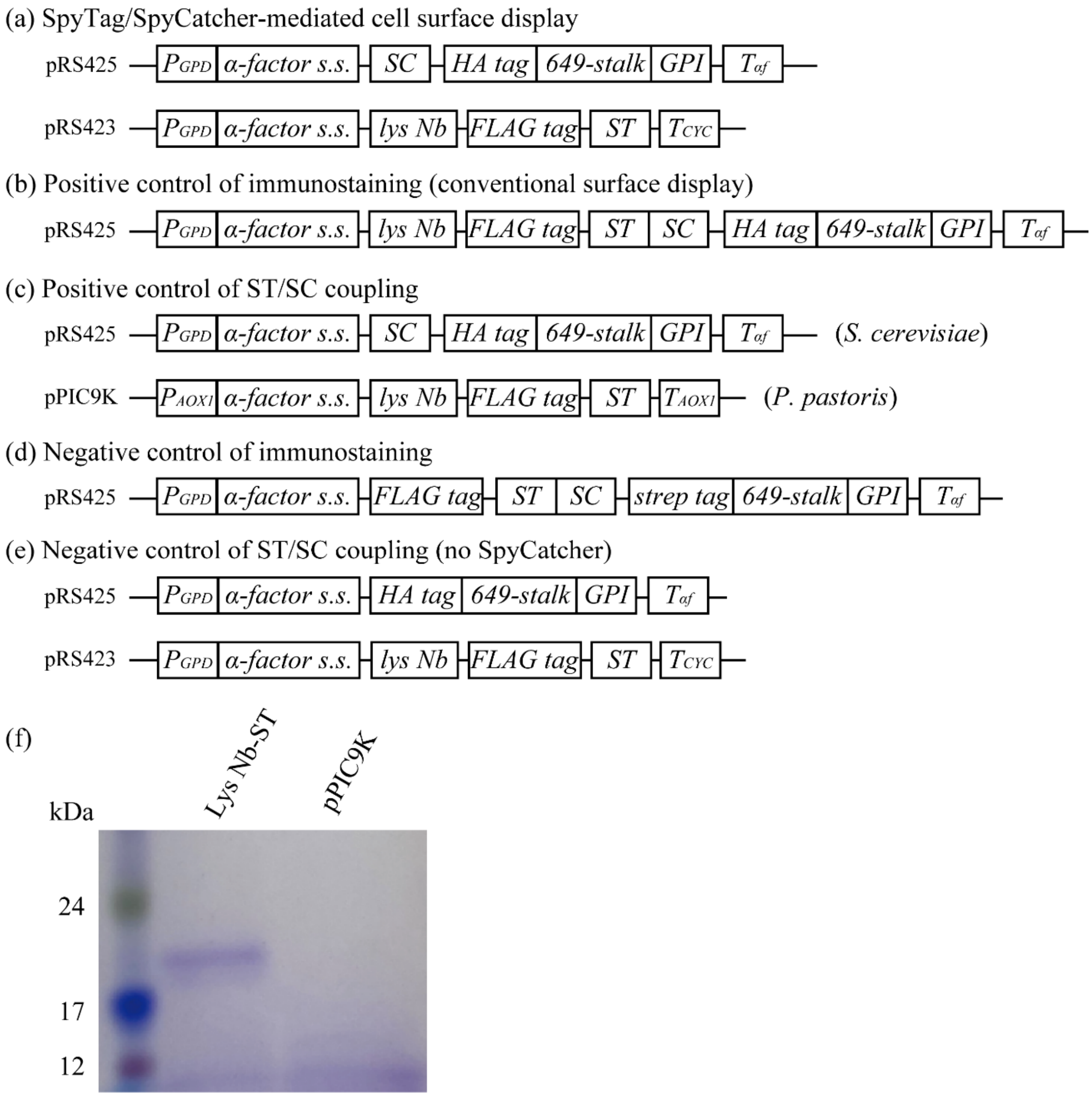

(a) SpyTag/SpyCatcher-mediated cell surface display
pRS425 P_{GPD} α-factor s.s. SC HA tag 649-stalk GPI $T_{\alpha f}$
pRS423 P_{GPD} α-factor s.s. lys Nb FLAG tag ST T_{CYC}
(b) Positive control of immunostaining (conventional surface display)
pRS425 P_{GPD} α-factor s.s. lys Nb FLAG tag ST SC HA tag 649-stalk GPI $T_{\alpha f}$
(c) Positive control of ST/SC coupling
pRS425 P_{GPD} α-factor s.s. SC HA tag 649-stalk GPI $T_{\alpha f}$ (S. cerevisiae)
pPIC9K P_{AOX1} α-factor s.s. lys Nb FLAG tag ST T_{AOX1} (P. pastoris)
(d) Negative control of immunostaining
pRS425 P_{GPD} α-factor s.s. FLAG tag ST SC strep tag 649-stalk GPI $T_{\alpha f}$
(e) Negative control of ST/SC coupling (no SpyCatcher)
pRS425 P_{GPD} α-factor s.s. HA tag 649-stalk GPI $T_{\alpha f}$
pRS423 P_{GPD} α-factor s.s. lys Nb FLAG tag ST T_{CYC}
(f)
Lys Nb-ST
pPIC9K
kDa
24
17
12

Figure S2 Design of SpyTag/SpyCatcher-mediated cell surface display. Genetic constructs for (a) SpyTag/SpyCatcher mediated surface display and (b)-(e) control strains. (b) Positive control strain of immunostaining. This strain produced anti-lysozyme nanobody and an anchor protein as a fusion protein. (c) Positive control strain of ST/SC coupling. The nananobody-SpyTag fusion protein was produced in *Pichia pastoris*, and mixed with a SpyCatcher-displaying yeast strain for *in vitro* isopeptide formation. (d) Negative control strain of immunostaining. This strain had no nanobody sequence and a FLAG tag instead of an HA tag, and used to examine the nonspecific absorption during staining for the flow cytometry analysis. (e) Negative control strain of ST/SC coupling. This strain had no SpyCatcher sequence and used to examine nonspecific protein ligation events. The pRS423 backbone vector was transformed into (b)-(d) strains with pRS425 constructs. P_{GPD}, glyceraldehyde-3-phosphate dehydrogenase promoter; *α-factor s.s.*, improved secretion signal of mating factor α preprotein from *Saccharomyces serevisiae*; *SC*, SpyCatcher; *649-stalk*, a synthetic anchor protein consisting of 649 amino acids; *GPI*, glycosylphosphatidylinositol attachment signal; $T_{\alpha f}$, the terminator region from *S. cerevisiae* gene encoding mating factor alpha; *lys Nb*, anti-hen egg-white lysozyme nanobody; ST, SpyTag; T_{CYC}, the terminator region from *S. cerevisiae* gene encoding Cytochrome c; P_{AOX1}, alcohol oxidase I promoter; T_{AOX1}, alcohol oxidase I terminator. (f) SDS-PAGE analysis of anti-hen egg-white lysozyme nanobody produced in *P. pastoris*. The culture medium was applied to an SDS-PAGE gel, and proteins were stained by Coomassie Brilliant Blue. pPIC9K is a backbone vector used as a negative control. This figure was created using Illustrator CS2 (https://www.adobe.com/).

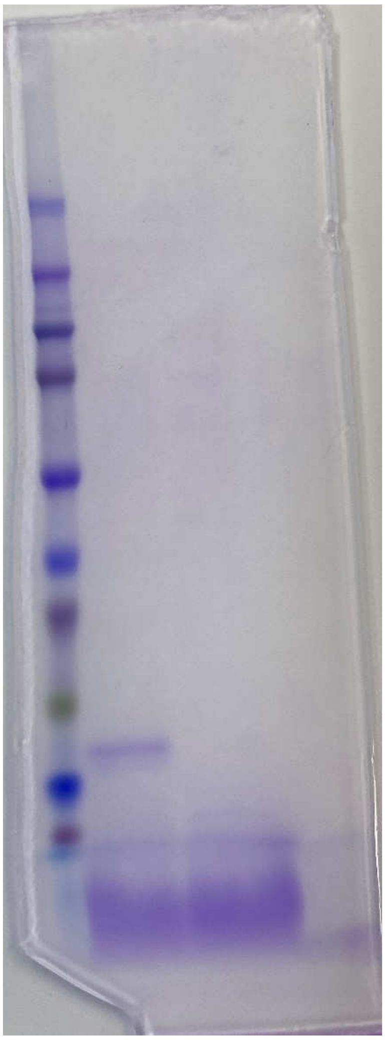

Figure S3 Full-length gel image of Supplementary Information Figure S2f

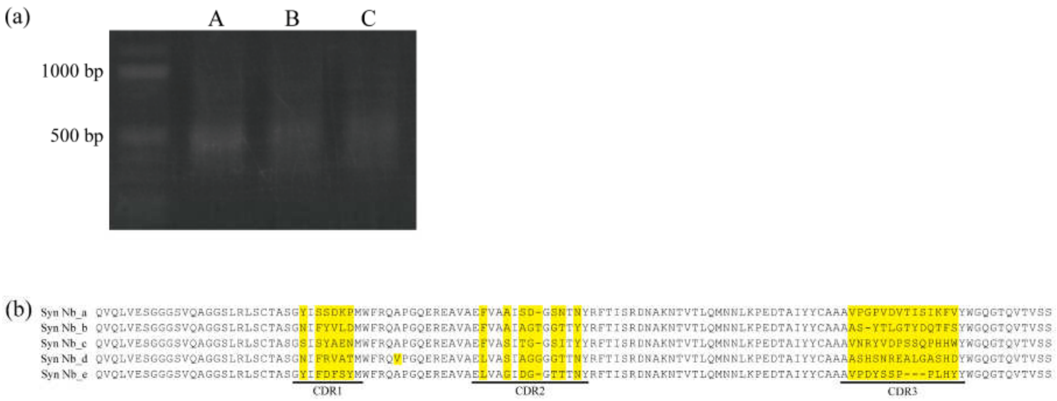

Figure S4 Construction of a synthetic nanobody library. (a) Agarose gel electrophoresis of overlap-extension -PCR products. A/B/C are derived from DNA pool mix A (351 bp), mix B (363 bp) or mix C (375 bp) respectively (see Methods). (b) Multiple alignment of five nanobodies derived from the synthetic nanobody library. Sequence alignment was carried out using MUSCLE software MegaX (version 10. 2). All sequences of nanobodies are shown and randomized regions are highlighted by yellow. CDR, complementary determining region. This figure was created using Illustrator CS2 (https://www.adobe.com/).

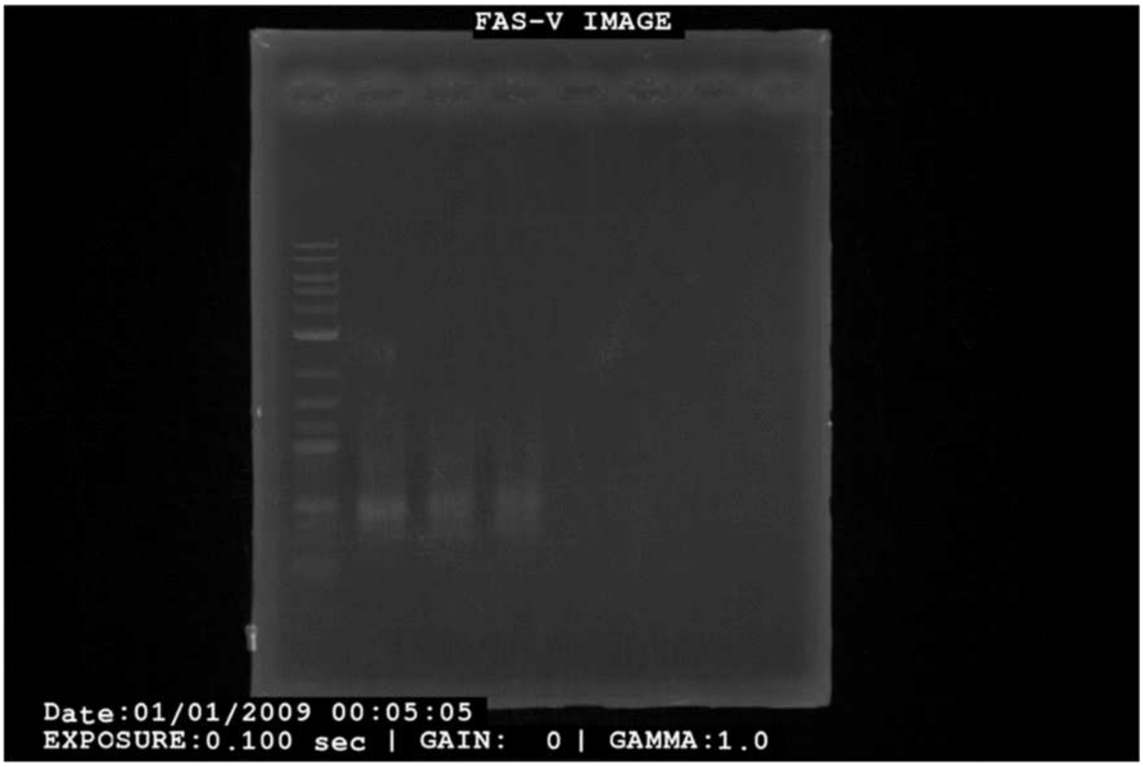

Figure S5 Full-length gel image of Supplementary Information Figure 4a. We did not set up the date of the gel imaging device FAS-V, and the described date is not correct.

Table S1 Mean relative fluorescence intensity of double-positive cells

Backbone vector	Origin	Copy number	AF488 (anti-HA)	AF647 (binding to lysozyme)
pRS425	2 micron	Medium	331 ± 92	152 ± 41
pULD1	2 micron	High	152 ± 44	84.9 ± 11
pRS415	CEN/ARS	Low	92 ± 14	73 ± 3.0
pRS403	Integration	Single	71 ± 20	75 ± 1.8

The density plots in Fig. 2 were used to calculate mean relative fluorescence intensity. The data from three independent experiments were represented as means ± standard deviations.

Table S2 Antibodies used for immunofluorescence labeling

Antibody	Supplier	Product number	Dilution ratio
Mouse anti-HA	Nacalai Tesque	06340-54	1:1000
Mouse anti-FLAG	Sigma-Aldrich	F3040	1:1000
*AF488-conjugated goat anti-mouse	Invitrogen	A-11001	1:1000
*AF546-conjugated goat anti-mouse	Invitrogen	A-11003	1:500
*AF647-conjugated goat anti-mouse	Abcam	ab150115	1:1000

*Secondary antibodies

Table S3 Primers for construction of the synthetic nanobody library

Name	Sequence
P1_for	caagtccagctggtcgagtcaggtggtggatctgtccaagccggggggtc
P2_rev	gcctgacgccgtacatgatagtcttagagaccccccggcttggac
P3_for	gactatcatgtacggcgtcaggcWMTattTYT####atgtggttcaggcaagcaccg
P4_rev	ttccgcaactgcctctctttcctggcccggtgcttgcctgaaccacat
P5_for	ggaaagagaggcagttgcggaaYTTgttgccRSTattRVT#ggtRSTANTaccWATtatcgttttactattagcagagacaacgcaaaaaatac
P6_rev	cgctgcggcacaataatagatcgcagtatcttctggcttcagattattcatttgtagcgtaacagtatttttgcgttgtctctgctaatagtaaaacgata
P7a_for	cgatctattattgtgccgcagcgGYT#######YWT#tattggggacaaggaacacaggtcac
P7b_for	cgatctattattgtgccgcagcgGYT###########YWT#tattggggacaaggaacacaggtcac
P7c_for	cgatctattattgtgccgcagcgGYT###############YWT#tattggggacaaggaacacaggtcac
P8_rev	agatgatactgtgacctgtgttccttgtccccaata

means Custom Trimer Mix

14% Y

12% G

10% S

9% D

7% T

6% R

6% A

5% L

5% V

4% P

4% N

3% F

3% E

3% I

3% W

2% Q

2% K

2% H

0% C

0% M

0% Stop

Plasmid maps

Full sequences of plasmids used in this study are shown below.

ST/SC ligation / pAnchor-HA (pRS425) (10599 bp)

ACACCGAACTGAGATACCTACAGCGTGAGCTATGAGAAAGCGCCACGCTTCCCGAAGGGAGAAAGGCGGACAGGTATCCGGTAAGCGGCAGGGTCGGAACAGGAGAG
TGTGGCTTGACTCTATGGATGTCGCACTCGATACTCTTTCGCGGTGCGAAGGGCTTCCCTCTTTCCGCCTGTCCATAGGCCATTCGCCGTCCCAGCCTTGTCCTCTC

20 40 60 80 100

CGCACGAGGGAGCTTCCAGGGGGAAACGCCTGGTATCTTTATAGTCCTGTCGGGTTTCGCCACCTCTGACTTGAGCGTCGATTTTTGTGATGCTCGTCAGGGGGGCG
GCGTGCTCCCTCGAAGGTCCCCCTTTGCGGACCATAGAAATATCAGGACAGCCCAAAGCGGTGGAGACTGAACTCGCAGCTAAAAACACTACGAGCAGTCCCCCCGC

120 140 160 180 200

GAGCCTATGGAAAAACGCCAGCAACGCGGCCTTTTTACGGTTCCTGGCCTTTTGCTGGCCTTTTGCTCACATGTTCTTTCCTGCGTTATCCCCTGATTCTGTGGATA
CTCGGATACCTTTTTGCGGTCGTTGCGCCGGAAAAATGCCAAGGACCGGAAAACGACCGGAAAACGAGTGTACAAGAAAGGACGCAATAGGGGACTAAGACACCTAT

220 240 260 280 300 320

ACCGTATTACCGCCTTTGAGTGAGCTGATACCGCTCGCCGCAGCCGAACGACCGAGCGCAGCGAGTCAGTGAGCGAGGAAGCGGAAGAGCGCCCAATACGCAAACCG
TGGCATAATGGCGGAAACTCACTCGACTATGGCGAGCGGCGTCGGCTTGCTGGCTCGCGTCGCTCAGTCACTCGCTCCTTCGCCTTCTCGCGGGTTATGCGTTTGGC

340 360 380 400 420

CCTCTCCCCGCGCGTTGGCCGATTCATTAATGCAGCTGGCACGACAGGTTTCCCGACTGGAAAGCGGGCAGTGAGCGCAACGCAATTAATGTGAGTTAGCTCACTCA
GGAGAGGGGCGCGCAACCGGCTAAGTAATTACGTCGACCGTGCTGTCCAAAGGGCTGACCTTTCGCCCGTCACTCGCGTTGCGTTAATTACACTCAATCGAGTGAGT

440 460 480 500 520

TTAGGCACCCCAGGCTTTACACTTTATGCTTCCGGCTCGTATGTTGTGTGGAATTGTGAGCGGATAACAATTTCACACAGGAAACAGCTATGACCATGATTACGCCA
AATCCGTGGGGTCCGAAATGTGAAATACGAAGGCCGAGCATACAACACACCTTAACACTCGCCTATTGTTAAAGTGTGTCCTTTGTCGATACTGGTACTAATGCGGT

lac promoter LacO M13-rev

540 560 580 600 620 640

AGCGCGCAATTAACCCTCACTAAAGGGAACAAAAGCTGGAGCTAGTATACTCTTTCTTCAACAATTAAATACTCTCGGTAGCCAAGTTGGTTTAAGGCGCAAGACTG
TCGCGCGTTAATTGGGAGTGATTTCCCTTGTTTTCGACCTCGATCATATGAGAAAGAAGTTGTTAATTTATGAGAGCCATCGGTTCAACCAAATTCCGCGTTCTGAC

T3
T3 promoter

660 680 700 720 740

TAATTTATCACTACGAAATCTTGAGATCGGGCGTTCGACTCGCCCCCGGGAGAGATGGCCGGCATGGTCCCAGCCTCCTCGCTGGCGCCGGCTGGGCAACACCTTCG
ATTAAATAGTGATGCTTTAGAACTCTAGCCCGCAAGCTGAGCGGGGGCCCTCTCTACCGGCCGTACCAGGGTCGGAGGAGCGACCGCGGCCGACCCGTTGTGGAAGC

760 780 800 820 840

GGTGGCGAATGGGACTTTTTATGTGCGTATTGCTTTCAGTTTTAGAGCTAGAAATAGCAAGTTAAAATAAGGCTAGTCCGTTATCAACTTGAAAAAGTGGCACCGAG
CCACCGCTTACCCTGAAAAATACACGCATAACGAAAGTCAAAATCTCGATCTTTATCGTTCAATTTTATTCCGATCAGGCAATAGTTGAACTTTTTCACCGTGGCTC

860 880 900 920 940 960

TCGGTGCTTTTTTTATTTTTTGTCACTATTGTTATGTAAAATGCCACCTCTGACAGTATGGAACGCAAACTTCTGTCTAGTGGATAGTCGACAAGCTTACCAGTTCT
AGCCACGAAAAAAATAAAAAACAGTGATAACAATACATTTTACGGTGGAGACTGTCATACCTTGCGTTTGAAGACAGATCACCTATCAGCTGTTCGAATGGTCAAGA

GPD ...ter

980 1,000 1,020 1,040 1,060

CACACGGAACACCACTAATGGACACAAATTCGAAATACTTTGACCCTATTTTCGAGGACCTTGTCACCTTGAGCCCAAGAGAGCCAAGATTTAAATTTTCCTATGAC
GTGTGCCTTGTGGTGATTACCTGTGTTTAAGCTTTATGAAACTGGGATAAAAGCTCCTGGAACAGTGGAACTCGGGTTCTCTCGGTTCTAAATTTAAAAGGATACTG
GPD promoter
1,080 1,100 1,120 1,140 1,160

TTGATGCAAATTCCCAAAGCTAATAACATGCAAGACACGTACGGTCAAGAAGACATATTTGACCTCTTAACAGGTTCAGACGCGACTGCCTCATCAGTAAGACCCGT
AACTACGTTTAAGGGTTTCGATTATTGTACGTTCTGTGCATGCCAGTTCTTCTGTATAAACTGGAGAATTGTCCAAGTCTGCGCTGACGGAGTAGTCATTCTGGGCA
GPD promoter
1,180 1,200 1,220 1,240 1,260 1,280

TGAAAAGAACTTACCTGAAAAAAACGAATATATACTAGCGTTGAATGTTAGCGTCAACAACAAGAAGTTTAATGACGCGGAGGCCAAGGCAAAAAGATTCCTTGATT
ACTTTTCTTGAATGGACTTTTTTTGCTTATATATGATCGCAACTTACAATCGCAGTTGTTGTTCTTCAAATTACTGCGCCTCCGGTTCCGTTTTTCTAAGGAACTAA
GPD promoter
1,300 1,320 1,340 1,360 1,380

ACGTAAGGGAGTTAGAATCATTTTGAATAAAAAACACGCTTTTTCAGTTCGAGTTTATCATTATCAATACTGCCATTTCAAAGAATACGTAAATAATTAATAGTAGT
TGCATTCCCTCAATCTTAGTAAAACTTATTTTTTGTGCGAAAAAGTCAAGCTCAAATAGTAATAGTTATGACGGTAAAGTTTCTTATGCATTTATTAATTATCATCA
GPD promoter
1,400 1,420 1,440 1,460 1,480

GATTTTCCTAACTTTATTTAGTCAAAAAATTAGCCTTTTAATTCTGCTGTAACCCGTACATGCCCAAAATAGGGGGCGGGTTACACAGAATATATAACATCGTAGGT
CTAAAAGGATTGAAATAAATCAGTTTTTTAATCGGAAAATTAAGACGACATTGGGCATGTACGGGTTTTATCCCCCGCCCAATGTGTCTTATATATTGTAGCATCCA
GPD promoter
1,500 1,520 1,540 1,560 1,580 1,600

GTCTGGGTGAACAGTTTATTCCTGGCATCCACTAAATATAATGGAGCCCGCTTTTTAAGCTGGCATCCAGAAAAAAAAAGAATCCCAGCACCAAAATATTGTTTTCT
CAGACCCACTTGTCAAATAAGGACCGTAGGTGATTTATATTACCTCGGGCGAAAAATTCGACCGTAGGTCTTTTTTTTTCTTAGGGTCGTGGTTTTATAACAAAAGA
GPD promoter
1,620 1,640 1,660 1,680 1,700

TCACCAACCATCAGTTCATAGGTCCATTCTCTTAGCGCAACTACAGAGAACAGGGGCACAAACAGGCAAAAAACGGGCACAACCTCAATGGAGTGATGCAACCTGCC
AGTGGTTGGTAGTCAAGTATCCAGGTAAGAGAATCGCGTTGATGTCTCTTGTCCCCGTGTTTGTCCGTTTTTTGCCCGTGTTGGAGTTACCTCACTACGTTGGACGG
GPD promoter
1,720 1,740 1,760 1,780 1,800

TGGAGTAAATGATGACACAAGGCAATTGACCCACGCATGTATCTATCTCATTTTCTTACACCTTCTATTACCTTCTGCTCTCTCTGATTTGGAAAAAGCTGAAAAAA
ACCTCATTTACTACTGTGTTCCGTTAACTGGGTGCGTACATAGATAGAGTAAAAGAATGTGGAAGATAATGGAAGACGAGAGAGACTAAACCTTTTTCGACTTTTTT
GPD promoter
1,820 1,840 1,860 1,880 1,900 1,920

AAGGTTGAAACCAGTTCCCTGAAATTATTCCCCTACTTGACTAATAAGTATATAAAGACGGTAGGTATTGATTGTAATTCTGTAAATCTATTTCTTAAACTTCTTAA
TTCCAACTTTGGTCAAGGGACTTTAATAAGGGGATGAACTGATTATTCATATATTTCTGCCATCCATAACTAACATTAAGACATTTAGATAAAGAATTTGAAGAATT

GPD promoter

1,940 1,960 1,980 2,000 2,020

ATTCTACTTTTATAGTTAGTCTTTTTTTTAGTTTTAAAACACCAGAACTTAGTTTCGACGGATTCTAGAACTAGTGGATCCATGAGATTCCCATCTATCTTCACCGC
TAAGATGAAAATATCAATCAGAAAAAAAATCAAAATTTTGTGGTCTTGAATCAAAGCTGCCTAAGATCTTGATCACCTAGGTACTCTAAGGGTAGATAGAAGTGGCG

1 2 3 4 5 6 7 8 9
M R F P S I F T A

GPD promoter
Mating fac...ha leader

2,040 2,060 2,080 2,100 2,120 2,140

TGTTTTGTTCGCTGCTTCTTCTGCTTTGGCTGCTCCAGCTAACACCACCACCGAAGACGAAACCGCTCAAATCCCAGCTGAAGCTGTTATCGACTACTCTGACTTGG
ACAAAACAAGCGACGAAGAAGACGAAACCGACGAGGTCGATTGTGGTGGTGGCTTCTGCTTTGGCGAGTTTAGGGTCGACTTCGACAATAGCTGATGAGACTGAACC

10 11 12 13 14 15 16 17 18 19 20 21 22 23 24 25 26 27 28 29 30 31 32 33 34 35 36 37 38 39 40 41 42 43 44
V L F A A S S A L A A P A N T T T E D E T A Q I P A E A V I D Y S D L

Mating factor alpha leader

2,160 2,180 2,200 2,220 2,240

AAGGTGACTTCGACGCTGCTGCTTTGCCATTGTCTAACTCTACCAACAACGGTTTGTCTTCTACCAACACCACCATCGCTTCTATCGCTGCTAAGGAAGAAGGTGTT
TTCCACTGAAGCTGCGACGACGAAACGGTAACAGATTGAGATGGTTGTTGCCAAACAGAAGATGGTTGTGGTGGTAGCGAAGATAGCGACGATTCCTTCTTCCACAA

45 46 47 48 49 50 51 52 53 54 55 56 57 58 59 60 61 62 63 64 65 66 67 68 69 70 71 72 73 74 75 76 77 78 79 80
E G D F D A A A L P L S N S T N N G L S S T N T T I A S I A A K E E G V

Mating factor alpha leader

2,260 2,280 2,300 2,320 2,340

CAATTGGACAAGAGAGAAGCTAGCGCAGTAACTACCTTGTCTGGCTTATCAGGGGAACAGGGTCCTAGCGGTGACATGACGACAGAGGAGGACAGTGCGACCCACAT
GTTAACCTGTTCTCTCTTCGATCGCGTCATTGATGGAACAGACCGAATAGTCCCCTTGTCCCAGGATCGCCACTGTACTGCTGTCTCCTCCTGTCACGCTGGGTGTA

81 82 83 84 85 86
Q L D K R E

2 4 6 8 10 12 14 16 18 20 22 24 26
V T T L S G L S G E Q G P S G D M T T E E D S A T H I

Mating...eader
SpyCatcher

2,360 2,380 2,400 2,420 2,440 2,460

AAAGTTTAGCAAAAGAGATGAAGATGGAAGGGAACTAGCTGGAGCGACCATGGAACTTCGTGACAGCTCAGGGAAGACTATATCAACGTGGATCTCAGATGGTCACG
TTTCAAATCGTTTTCTCTACTTCTACCTTCCCTTGATCGACCTCGCTGGTACCTTGAAGCACTGTCGAGTCCCTTCTGATATAGTTGCACCTAGAGTCTACCAGTGC

28 30 32 34 36 38 40 42 44 46 48 50 52 54 56 58 60 62
K F S K R D E D G R E L A G A T M E L R D S S G K T I S T W I S D G H

SpyCatcher

2,480 2,500 2,520 2,540 2,560

```
TCAAGGACTTCTATTTGTACCCCGGAAAGTACACATTTGTGGAAACCGCCGCGCCGGATGGCTACGAGGTAGCGACCCCGATAGAATTCACCGTTAACGAGGACGGC
AGTTCCTGAAGATAAACATGGGGCCTTTCATGTGTAAACACCTTTGGCGGCGCGGCCTACCGATGCTCCATCGCTGGGGCTATCTTAAGTGGCAATTGCTCCTGCCG
 64   66   68   70   72   74   76   78   80   82   84   86   88   90   92   94   96   98
V K D F Y L Y P G K Y T F V E T A A P D G Y E V A T P I E F T V N E D G
SpyCatcher
2,580   2,600   2,620   2,640   2,660
```

```
CAAGTCACCGTAGATGGAGAGGCGACAGAGGGTGATGCCCACACTGGGGGCTCCGGCGGATCTGGTGGGAGCGGATCCTACCCATACGATGTTCCAGATTACGCTCA
GTTCAGTGGCATCTACCTCTCCGCTGTCTCCCACTACGGGTGTGACCCCCGAGGCCGCCTAGACCACCCTCGCCTAGGATGGGTATGCTACAAGGTCTAATGCGAGT
 100  102  104  106  108  110  112  1 2 3 4 5 6 7 8 9 10 11 1 2 3 4 5 6 7 8 9
Q V T V D G E A T E G D A H T G G S G G S G G S G S Y P Y D V P D Y A Q
SpyCatcher   HA tag
2,680   2,700   2,720   2,740   2,760   2,780
```

```
AACATCCTCACCCTCTACAGAAGCATCAGTATCAACCTCTAGTACCTCTTCCTCTGCTTCACAATCATCTGACCCAACTACAACATCTTCGTCCAGTTCATCGTCTT
TTGTAGGAGTGGGAGATGTCTTCGTAGTCATAGTTGGAGATCATGGAGAAGGAGACGAAGTGTTAGTAGACTGGGTTGATGTTGTAGAAGCAGGTCAAGTAGCAGAA
 2   4   6   8   10   12   14   16   18   20   22   24   26   28   30   32   34   36
T S S P S T E A S V S T S S T S S S A S Q S S D P T T T S S S S S S S
649 stalk
2,800   2,820   2,840   2,860   2,880
```

```
CCCCATCTTCCCAATCTGAGGAAATTTCATCATCACCGACGGTCTCAACTACACCATCAACTTCTTCATCATCTTCCTCAATGACTTCAACCACCACAACAAAGTCA
GGGGTAGAAGGGTTAGACTCCTTTAAAGTAGTAGTGGCTGCCAGAGTTGATGTGGTAGTTGAAGAAGTAGTAGAAGGAGTTACTGAAGTTGGTGGTGTTGTTTCAGT
 38   40   42   44   46   48   50   52   54   56   58   60   62   64   66   68   70   72
S P S S Q S E E I S S S P T V S T T P S T S S S S S S M T S T T T T K S
649 stalk
2,900   2,920   2,940   2,960   2,980
```

```
ATCTCAACTTCCACTACAAGTTCAGCTCCAGTTACAGATGTGACAGTTTCCTCATCGCCTAGTAAATCTACCTCTACTTCGACAAGTACAGAAACATCTAAAACACC
TAGAGTTGAAGGTGATGTTCAAGTCGAGGTCAATGTCTACACTGTCAAAGGAGTAGCGGATCATTTAGATGGAGATGAAGCTGTTCATGTCTTTGTAGATTTTGTGG
 74   76   78   80   82   84   86   88   90   92   94   96   98   100   102   104   106   108
I S T S T T S S A P V T D V T V S S S P S K S T S T S T S T E T S K T P
649 stalk
3,000   3,020   3,040   3,060   3,080   3,100
```

```
TACTTCAATGACAGAGTATACATCTAGTACATCGATAATTTCGACTCCAGTTAGTCACTCGCAGACAGGTTTGTCGGCTTCATCAAGTTCATCATCTACAACATCCG
ATGAAGTTACTGTCTCATATGTAGATCATGTAGCTATTAAAGCTGAGGTCAATCAGTGAGCGTCTGTCCAAACAGCCGAAGTAGTTCAAGTAGTAGATGTTGTAGGC
 110   112   114   116   118   120   122   124   126   128   130   132   134   136   138   140   142
T S M T E Y T S S T S I I S T P V S H S Q T G L S A S S S S S S T T S
649 stalk
3,120   3,140   3,160   3,180   3,200
```

```
GTTCTTCGTCCACTAAATCAGAAAGTTCGACAACATCTGGCTCTTCCCAGTCCGTGGAATCAACCTCCAGCCACGCCACTGTTCTTGCTAATTCCGCAGAAATGGTC
CAAGAAGCAGGTGATTTAGTCTTTCAAGCTGTTGTAGACCGAGAAGGGTCAGGCACCTTAGTTGGAGGTCGGTGCGGTGACAAGAACGATTAAGGCGTCTTTACCAG
144   146   148   150   152   154   156   158   160   162   164   166   168   170   172   174   176   178
G S S S T K S E S S T T S G S S Q S V E S T S S H A T V L A N S A E M V
649 stalk
3,220   3,240   3,260   3,280   3,300

ACAACATCCTCTAGTTCATCCTCAACATCCGAAATGTCATTAACTAGTACTGCTACCAGTGTACCAGTCTCATCTAGTAGCAGTACGACATATTCTACTAGCGCATC
TGTTGTAGGAGATCAAGTAGGAGTTGTAGGCTTTACAGTAATTGATCATGACGATGGTCACATGGTCAGAGTAGATCATCGTCATGCTGTATAAGATGATCGCGTAG
180   182   184   186   188   190   192   194   196   198   200   202   204   206   208   210   212   214
T T S S S S S S T S E M S L T S T A T S V P V S S S S S T T Y S T S A S
649 stalk
3,320   3,340   3,360   3,380   3,400   3,420

TACACAAGCCGTCACTACAACATCTTCTTCCACTGTATCTACAACTTCTTCTAGTACAACGTTAACAAGCGCATTCACACATTCTTCAACCACATCGTCCGACCAGC
ATGTGTTCGGCAGTGATGTTGTAGAAGAAGGTGACATAGATGTTGAAGAAGATCATGTTGCAATTGTTCGCGTAAGTGTGTAAGAAGTTGGTGTAGCAGGCTGGTCG
216   218   220   222   224   226   228   230   232   234   236   238   240   242   244   246   248   250
T Q A V T T T S S S T V S T T S S S T T L T S A F T H S S T T S S D Q
649 stalk
3,440   3,460   3,480   3,500   3,520

CACCCAGCGACACTACAAGTCCATCTACGACACACGAACCTCATGTAACCACTCAGACGTCATCAGAAACATCTTCTTCTAAGTCATCTTCTACTTCTTCTTCAAGT
GTGGGTCGCTGTGATGTTCAGGTAGATGCTGTGTGCTTGGAGTACATTGGTGAGTCTGCAGTAGTCTTTGTAGAAGAAGATTCAGTAGAAGATGAAGAAGAAGTTCA
252   254   256   258   260   262   264   266   268   270   272   274   276   278   280   282   284   286
P P S D T T S P S T T H E P H V T T Q T S S E T S S S K S S S T S S S S
649 stalk
3,540   3,560   3,580   3,600   3,620

ACATCTCAAACCTCTGAGTCTGCAACACCATCCGATTCCGTATCACCTGGAAGTTCTACATCAACATCTTCTAGTAGCACTTCTACTTCCACTTCTATTTCCAGTGG
TGTAGAGTTTGGAGACTCAGACGTTGTGGTAGGCTAAGGCATAGTGGACCTTCAAGATGTAGTTGTAGAAGATCATCGTGAAGATGAAGGTGAAGATAAAGGTCACC
288   290   292   294   296   298   300   302   304   306   308   310   312   314   316   318   320   322
T S Q T S E S A T P S D S V S P G S S T S T S S S S T S T S T S I S S G
649 stalk
3,640   3,660   3,680   3,700   3,720   3,740

AGAAACGACAACTTCTTCTTCTTCATCATCTGCCACGACCACTTCTAACAGCGCAACCTTGTCAGTCTCTACCACACAAACTTCGATTGAAGCCAGTTCATCTACTA
TCTTTGCTGTTGAAGAAGAAGAAGTAGTAGACGGTGCTGGTGAAGATTGTCGCGTTGGAACAGTCAGAGATGGTGTGTTTGAAGCTAACTTCGGTCAAGTAGATGAT
324   326   328   330   332   334   336   338   340   342   344   346   348   350   352   354   356
E T T T S S S S S S A T T T S N S A T L S V S T T Q T S I E A S S S T
649 stalk
3,760   3,780   3,800   3,820   3,840
```

```
CATCTACATCTAGTTCAACAATTACAACTTCAAGTAGTAGCGCTCACATATCGTCGAAATCTCAATCTAGTATTACCTATCCCTCTTCCTCGACATCTTCATCTACA
GTAGATGTAGATCAAGTTGTTAATGTTGAAGTTCATCATCGCGAGTGTATAGCAGCTTTAGAGTTAGATCATAATGGATAGGGAGAAGGAGCTGTAGAAGTAGATGT
358 360 362 364 366 368 370 372 374 376 378 380 382 384 386 388 390 392
T S T S S S T I T T S S S S A H I S S K S Q S S I T Y P S S S T S S S T
649 stalk
3,860 3,880 3,900 3,920 3,940

TCGTCCTCAATTTCTAGCGAATCTGAAAGTTTTGAATCGACATCAGCAGAAGATGCTCCATCAACAGCACCTTCATCAAGTGTCTCTTCTAAGAGTTCTACCTCTAC
AGCAGGAGTTAAAGATCGCTTAGACTTTCAAAACTTAGCTGTAGTCGTCTTCTACGAGGTAGTTGTCGTGGAAGTAGTTCACAGAGAAGATTCTCAAGATGGAGATG
394 396 398 400 402 404 406 408 410 412 414 416 418 420 422 424 426 428
S S S I S S E S E S F E S T S A E D A P S T A P S S S V S S K S S T S T
649 stalk
3,960 3,980 4,000 4,020 4,040 4,060

AACATCAAGCACATCGACATCTTCAAGCACTCCATCTCCATCACCATCTTCCGTGAGTTCTTCCTCCACCAGCTCATTGACAACTTCTGCTGTATCAACACCAGCTA
TTGTAGTTCGTGTAGCTGTAGAAGTTCGTGAGGTAGAGGTAGTGGTAGAAGGCACTCAAGAAGGAGGTGGTCGAGTAACTGTTGAAGACGACATAGTTGTGGTCGAT
430 432 434 436 438 440 442 444 446 448 450 452 454 456 458 460 462 464
T S S T S T S S S T P S P S P S S V S S S S T S S L T T S A V S T P A
649 stalk
4,080 4,100 4,120 4,140 4,160

CCTCTCATTCTCAAAGTACTGTAGTAACCACCACTACTATTACTACATCAACAGGTCCAGTGATGTCTACGACAACAGCTTATTCTTCTAGTTCTACTAGCAGCTCG
GGAGAGTAAGAGTTTCATGACATCATTGGTGGTGATGATAATGATGTAGTTGTCCAGGTCACTACAGATGCTGTTGTCGAATAAGAAGATCAAGATGATCGTCGAGC
466 468 470 472 474 476 478 480 482 484 486 488 490 492 494 496 498 500
T S H S Q S T V V T T T T I T T S T G P V M S T T T A Y S S S S T S S S
649 stalk
4,180 4,200 4,220 4,240 4,260 4,280

GAATCTTCTGAGGTTCAGTCTGTCATGTCATCTACGCCTAGTTCAACATCAACAACAACCAGTTCGGAATCTACTTCATCTAGCTCCACAGCTTCTACCTCACCATC
CTTAGAAGACTCCAAGTCAGACAGTACAGTAGATGCGGATCAAGTTGTAGTTGTTGTTGGTCAAGCCTTAGATGAAGTAGATCGAGGTGTCGAAGATGGAGTGGTAG
502 504 506 508 510 512 514 516 518 520 522 524 526 528 530 532 534 536
E S S E V Q S V M S S T P S S T S T T T S S E S T S S S S T A S T S P S
649 stalk
4,300 4,320 4,340 4,360 4,380

AACCTCGCAAACTTTCGAAACTTCTCCTACTATAGGAGGTGTCCCCTCAACCACTTCATTTGTCTCTACGCCAACAACGAAATTGTCGCACACTACTTCCACTATGA
TTGGAGCGTTTGAAAGCTTTGAAGAGGATGATATCCTCCACAGGGGAGTTGGTGAAGTAAACAGAGATGCGGTTGTTGCTTTAACAGCGTGTGATGAAGGTGATACT
538 540 542 544 546 548 550 552 554 556 558 560 562 564 566 568 570
T S Q T F E T S P T I G G V P S T T S F V S T P T T K L S H T T S T M
649 stalk
4,400 4,420 4,440 4,460 4,480
```

```
CAGCACAGTCCGATAGTAAGTCTACCCACTCCTCAAGCACATCGACAGAAGATAAATCATCCACTGCTTCTGCAGTTGACGAAAGCACTACAACATCCACTTCCACG
GTCGTGTCAGGCTATCATTCAGATGGGTGAGGAGTTCGTGTAGCTGTCTTCTATTTAGTAGGTGACGAAGACGTCAACTGCTTTCGTGATGTTGTAGGTGAAGGTGC
572 574 576 578 580 582 584 586 588 590 592 594 596 598 600 602 604 606
T A Q S D S K S T H S S S T S T E D K S S T A S A V D E S T T T S T S T
649 stalk
4,500 4,520 4,540 4,560 4,580 4,600
```

```
GAGTCTACTACATCAGTAACATCAGGCACCTCCCATTCCGCTAAAGAATCTTCGTCAAATTCTAAGGTGTATAGTTCACAGACAGCACACTCATCCATAAGTGTTGC
CTCAGATGATGTAGTCATTGTAGTCCGTGGAGGGTAAGGCGATTTCTTAGAAGCAGTTTAAGATTCCACATATCAAGTGTCTGTCGTGTGAGTAGGTATTCACAACG
608 610 612 614 616 618 620 622 624 626 628 630 632 634 636 638 640 642
E S T T S V T S G T S H S A K E S S S N S K V Y S S Q T A H S S I S V A
649 stalk
4,620 4,640 4,660 4,680 4,700
```

```
ATCATCACCTAGTACAAAGGGCGCCCAAATCCAATCTTCTATGGTTGAAATCTCTACCTACGCTGGTTCTGCTAACTCTGTTAACGCTGGTGCTGGTGCTGGTGCTT
TAGTAGTGGATCATGTTTCCCGCGGGTTTAGGTTAGAAGATACCAACTTTAGAGATGGATGCGACCAAGACGATTGAGACAATTGCGACCACGACCACGACCACGAA
644 646 648 650 652 654 656 658 660 662 664 666 668 670 672 674 676 678
S S P S T K G A Q I Q S S M V E I S T Y A G S A N S V N A G A G A G A
649 stalk          GPI anchor
4,720 4,740 4,760 4,780 4,800
```

```
TGTTCTTGTTGTTGTCTTTGGCTATCATCTAATGATTAATTAACTCGAGATCTGATAACAACAGTGTAGATGTAACAAAATCGACTTTGTTCCCACTGTACTTTTAG
ACAAGAACAACAACAGAAACCGATAGTAGATTACTAATTAATTGAGCTCTAGACTATTGTTGTCACATCTACATTGTTTTAGCTGAAACAAGGGTGACATGAAAATC
680 682 684 686 688 1
L F L L L S L A I I *
GPI anchor          alpha factor terminator
4,820 4,840 4,860 4,880 4,900 4,920
```

```
CTCGTACAAAATACAATATACTTTTCATTTCTCCGTAAACAACATGTTTTCCCATGTAATATCCTTTTCTATTTTTCGTTCCGTTACCAACTTTACACATACTTTAT
GAGCATGTTTTATGTTATATGAAAAGTAAAGAGGCATTTGTTGTACAAAAGGGTACATTATAGGAAAAGATAAAAAGCAAGGCAATGGTTGAAATGTGTATGAAATA
alpha factor terminator
4,940 4,960 4,980 5,000 5,020
```

```
ATAGCTATTCACTTCTATACACTAAAAAACTAAGACAATTTTAATTTTGCTGCCTGCCATATTTCAATTTGTTATAAATTCCTATAATTTATCCTATTAGTAGCTAA
TATCGATAAGTGAAGATATGTGATTTTTTGATTCTGTTAAAATTAAAACGACGGACGGTATAAAGTTAAACAATATTTAAGGATATTAAATAGGATAATCATCGATT
alpha factor terminator
5,040 5,060 5,080 5,100 5,120
```

```
AAAAAGATGAATGTGAATCGAATCCTAAGAGAATTGGCCGGTACCCAATTCGCCCTATAGTGAGTCGTATTACGCGCGCTCACTGGCCGTCGTTTTACAACGTCGTG
TTTTTCTACTTACACTTAGCTTAGGATTCTCTTAACCGGCCATGGGTTAAGCGGGATATCACTCAGCATAATGCGCGCGAGTGACCGGCAGCAAAATGTTGCAGCAC
alpha factor terminator          T7          M13-fwd
5,140 5,160 5,180 5,200 5,220 5,240
```

ACTGGGAAAACCCTGGCGTTACCCAACTTAATCGCCTTGCAGCACATCCCCCTTTCGCCAGCTGGCGTAATAGCGAAGAGGCCCGCACCGATCGCCCTTCCCAACAG
TGACCCTTTTGGGACCGCAATGGGTTGAATTAGCGGAACGTCGTGTAGGGGGAAAGCGGTCGACCGCATTATCGCTTCTCCGGGCGTGGCTAGCGGGAAGGGTTGTC
LacZ alpha
5,260 5,280 5,300 5,320 5,340

TTGCGCAGCCTGAATGGCGAATGGACGCGCCCTGTAGCGGCGCATTAAGCGCGGCGGGTGTGGTGGTTACGCGCAGCGTGACCACTACACTTGCCAGCGCCCTAGCG
AACGCGTCGGACTTACCGCTTACCTGCGCGGGACATCGCCGCGTAATTCGCGCCGCCCACACCACCAATGCGCGTCGCACTGGTGATGTGAACGGTCGCGGGATCGC
LacZ alpha
5,360 5,380 5,400 5,420 5,440

CCCGCTCCTTTCGCTTTCTTCCCTTCCTTTCTCGCCACGTTCGCCGGCTTTCCCCGTCAAGCTCTAAATCGGGGGCTCCCTTTAGGGTTCCGATTTAGTGCTTTACG
GGGCGAGGAAAGCGAAAGAAGGGAAGGAAAGAGCGGTGCAAGCGGCCGAAAGGGGCAGTTCGAGATTTAGCCCCCGAGGGAAATCCCAAGGCTAAATCACGAAATGC
5,460 5,480 5,500 5,520 5,540 5,560

GCACCTCGACCCCAAAAAACTTGATTAGGGTGATGGTTCACGTAGTGGGCCATCGCCCTGATAGACGGTTTTTCGCCCTTTGACGTTGGAGTCCACGTTCTTTAATA
CGTGGAGCTGGGGTTTTTTGAACTAATCCCACTACCAAGTGCATCACCCGGTAGCGGGACTATCTGCCAAAAAGCGGGAAACTGCAACCTCAGGTGCAAGAAATTAT
5,580 5,600 5,620 5,640 5,660

GTGGACTCTTGTTCCAAACTGGAACAACACTCAACCCTATCTCGGTCTATTCTTTTGATTTATAAGGGATTTTGCCGATTTCGGCCTATTGGTTAAAAAATGAGCTG
CACCTGAGAACAAGGTTTGACCTTGTTGTGAGTTGGGATAGAGCCAGATAAGAAAACTAAATATTCCCTAAAACGGCTAAAGCCGGATAACCAATTTTTTACTCGAC
5,680 5,700 5,720 5,740 5,760

ATTTAACAAAAATTTAACGCGAATTTTAACAAAATATTAACGCTTACAATTTCCTGATGCGGTATTTTCTCCTTACGCATCTGTGCGGTATTTCACACCGCATAGGG
TAAATTGTTTTTAAATTGCGCTTAAAATTGTTTTATAATTGCGAATGTTAAAGGACTACGCCATAAAAGAGGAATGCGTAGACACGCCATAAAGTGTGGCGTATCCC
5,780 5,800 5,820 5,840 5,860 5,880

TAATAACTGATATAATTAAATTGAAGCTCTAATTTGTGAGTTTAGTATACATGCATTTACTTATAATACAGTTTTTTAAGCAAGGATTTTCTTAACTTCTTCGGCGA
ATTATTGACTATATTAATTTAACTTCGAGATTAAACACTCAAATCATATGTACGTAAATGAATATTATGTCAAAAAATTCGTTCCTAAAAGAATTGAAGAAGCCGCT
LEU2
5,900 5,920 5,940 5,960 5,980

CAGCATCACCGACTTCGGTGGTACTGTTGGAACCACCTAAATCACCAGTTCTGATACCTGCATCCAAAACCTTTTTAACTGCATCTTCAATGGCCTTACCTTCTTCA
GTCGTAGTGGCTGAAGCCACCATGACAACCTTGGTGGATTTAGTGGTCAAGACTATGGACGTAGGTTTTGGAAAAATTGACGTAGAAGTTACCGGAATGGAAGAAGT
LEU2
6,000 6,020 6,040 6,060 6,080

GGCAAGTTCAATGACAATTTCAACATCATTGCAGCAGACAAGATAGTGGCGATAGGGTTGACCTTATTCTTTGGCAAATCTGGAGCAGAACCGTGGCATGGTTCGTA
CCGTTCAAGTTACTGTTAAAGTTGTAGTAACGTCGTCTGTTCTATCACCGCTATCCCAACTGGAATAAGAAACCGTTTAGACCTCGTCTTGGCACCGTACCAAGCAT
LEU2
6,100 6,120 6,140 6,160 6,180 6,200

CAAACCAAATGCGGTGTTCTTGTCTGGCAAAGAGGCCAAGGACGCAGATGGCAACAAACCCAAGGAACCTGGGATAACGGAGGCTTCATCGGAGATGATATCACCAA
GTTTGGTTTACGCCACAAGAACAGACCGTTTCTCCGGTTCCTGCGTCTACCGTTGTTTGGGTTCCTTGGACCCTATTGCCTCCGAAGTAGCCTCTACTATAGTGGTT
LEU2
6,220 6,240 6,260 6,280 6,300

ACATGTTGCTGGTGATTATAATACCATTTAGGTGGGTTGGGTTCTTAACTAGGATCATGGCGGCAGAATCAATCAATTGATGTTGAACCTTCAATGTAGGGAATTCG
TGTACAACGACCACTAATATTATGGTAAATCCACCCAACCCAAGAATTGATCCTAGTACCGCCGTCTTAGTTAGTTAACTACAACTTGGAAGTTACATCCCTTAAGC
LEU2
6,320 6,340 6,360 6,380 6,400 6,420

TTCTTGATGGTTTCCTCCACAGTTTTTCTCCATAATCTTGAAGAGGCCAAAACATTAGCTTTATCCAAGGACCAAATAGGCAATGGTGGCTCATGTTGTAGGGCCAT
AAGAACTACCAAAGGAGGTGTCAAAAAGAGGTATTAGAACTTCTCCGGTTTTGTAATCGAAATAGGTTCCTGGTTTATCCGTTACCACCGAGTACAACATCCCGGTA
LEU2
6,440 6,460 6,480 6,500 6,520

GAAAGCGGCCATTCTTGTGATTCTTTGCACTTCTGGAACGGTGTATTGTTCACTATCCCAAGCGACACCATCACCATCGTCTTCCTTTCTCTTACCAAAGTAAATAC
CTTTCGCCGGTAAGAACACTAAGAAACGTGAAGACCTTGCCACATAACAAGTGATAGGGTTCGCTGTGGTAGTGGTAGCAGAAGGAAAGAGAATGGTTTCATTTATG
LEU2
6,540 6,560 6,580 6,600 6,620

CTCCCACTAATTCTCTGACAACAACGAAGTCAGTACCTTTAGCAAATTGTGGCTTGATTGGAGATAAGTCTAAAAGAGAGTCGGATGCAAAGTTACATGGTCTTAAG
GAGGGTGATTAAGAGACTGTTGTTGCTTCAGTCATGGAAATCGTTTAACACCGAACTAACCTCTATTCAGATTTTCTCTCAGCCTACGTTTCAATGTACCAGAATTC
LEU2
6,640 6,660 6,680 6,700 6,720 6,740

TTGGCGTACAATTGAAGTTCTTTACGGATTTTTAGTAAACCTTGTTCAGGTCTAACACTACCGGTACCCCATTTAGGACCACCCACAGCACCTAACAAAACGGCATC
AACCGCATGTTAACTTCAAGAAATGCCTAAAAATCATTTGGAACAAGTCCAGATTGTGATGGCCATGGGGTAAATCCTGGTGGGTGTCGTGGATTGTTTTGCCGTAG
LEU2
6,760 6,780 6,800 6,820 6,840

AGCCTTCTTGGAGGCTTCCAGCGCCTCATCTGGAAGTGGAACACCTGTAGCATCGATAGCAGCACCACCAATTAAATGATTTTCGAAATCGAACTTGACATTGGAAC
TCGGAAGAACCTCCGAAGGTCGCGGAGTAGACCTTCACCTTGTGGACATCGTAGCTATCGTCGTGGTGGTTAATTTACTAAAAGCTTTAGCTTGAACTGTAACCTTG
LEU2
6,860 6,880 6,900 6,920 6,940

GAACATCAGAAATAGCTTTAAGAACCTTAATGGCTTCGGCTGTGATTTCTTGACCAACGTGGTCACCTGGCAAAACGACGATCTTCTTAGGGGCAGACATTAGAATG
CTTGTAGTCTTTATCGAAATTCTTGGAATTACCGAAGCCGACACTAAAGAACTGGTTGCACCAGTGGACCGTTTTGCTGCTAGAAGAATCCCCGTCTGTAATCTTAC
LEU2
6,960 6,980 7,000 7,020 7,040 7,060

GTATATCCTTGAAATATATATATATATATATNTNGCTGAAATGTAAAAGGTAAGAAAAGTTAGAAAGTAAGACGATTGCTAACCACCTATTGGAAAAAACAATAGGTCC
CATATAGGAACTTTATATATATATATATATANANCGACTTTACATTTTCCATTCTTTTCAATCTTTCATTCTGCTAACGATTGGTGGATAACCTTTTTTGTTATCCAGG
7,080 7,100 7,120 7,140 7,160

TTAAATAATATTGTCAACTTCAAGTATTGTGATGCAAGCATTTAGTCATGAACGCTTCTCTATTCTATATGAAAAGCCGGTTCCGGCGCTCTCACCTTTCCTTTTTC
AATTTATTATAACAGTTGAAGTTCATAACACTACGTTCGTAAATCAGTACTTGCGAAGAGATAAGATATACTTTTCGGCCAAGGCCGCGAGAGTGGAAAGGAAAAAG

7,180 7,200 7,220 7,240 7,260

TCCCAATTTTTCAGTTGAAAAAGGTATATGCGTCAGGCGACCTCTGAAATTAACAAAAAATTTCCAGTCATCGAATTTGATTCTGTGCGATAGCGCCCCTGTGTGTT
AGGGTTAAAAAGTCAACTTTTTCCATATACGCAGTCCGCTGGAGACTTTAATTGTTTTTTAAAGGTCAGTAGCTTAAACTAAGACACGCTATCGCGGGGACACACAA

7,280 7,300 7,320 7,340 7,360 7,380

CTCGTTATGTTGAGGAAAAAAATAATGGTTGCTAAGAGATTCGAACTCTTGCATCTTACGATACCTGAGTATTCCCACAGTTTGAAAAGCTGTGGTATGGTGCACTC
GAGCAATACAACTCCTTTTTTTATTACCAACGATTCTCTAAGCTTGAGAACGTAGAATGCTATGGACTCATAAGGGTGTCAAACTTTTCGACACCATACCACGTGAG

7,400 7,420 7,440 7,460 7,480

TCAGTACAATCTGCTCTGATGCCGCATAGTTAAGCCAGCCCCGACACCCGCCAACACCCGCTGACGCGCCCTGACGGGCTTGTCTGCTCCCGGCATCCGCTTACAGA
AGTCATGTTAGACGAGACTACGGCGTATCAATTCGGTCGGGGCTGTGGGCGGTTGTGGGCGACTGCGCGGGACTGCCCGAACAGACGAGGGCCGTAGGCGAATGTCT

7,500 7,520 7,540 7,560 7,580

CAAGCTGTGACCGTCTCCGGGAGCTGCATGTGTCAGAGGTTTTCACCGTCATCACCGAAACGCGCGAGACGAAAGGGCCTCGTGATACGCCTATTTTTATAGGTTAA
GTTCGACACTGGCAGAGGCCCTCGACGTACACAGTCTCCAAAAGTGGCAGTAGTGGCTTTGCGCGCTCTGCTTTCCCGGAGCACTATGCGGATAAAAATATCCAATT

7,600 7,620 7,640 7,660 7,680 7,700

TGTCATGATAATAATGGTTTCTTAGTATGATCCAATATCAAAGGAAATGATAGCATTGAAGGATGAGACTAATCCAATTGAGGAGTGGCAGCATATAGAACAGCTAA
ACAGTACTATTATTACCAAAGAATCATACTAGGTTATAGTTTCCTTTACTATCGTAACTTCCTACTCTGATTAGGTTAACTCCTCACCGTCGTATATCTTGTCGATT

2 micron origin

7,720 7,740 7,760 7,780 7,800

AGGGTAGTGCTGAAGGAAGCATACGATACCCCGCATGGAATGGGATAATATCACAGGAGGTACTAGACTACCTTTCATCCTACATAAATAGACGCATATAAGTACGC
TCCCATCACGACTTCCTTCGTATGCTATGGGGCGTACCTTACCCTATTATAGTGTCCTCCATGATCTGATGGAAAGTAGGATGTATTTATCTGCGTATATTCATGCG

2 micron origin

7,820 7,840 7,860 7,880 7,900

ATTTAAGCATAAACACGCACTATGCCGTTCTTCTCATGTATATATATATACAGGCAACACGCAGATATAGGTGCGACGTGAACAGTGAGCTGTATGTGCGCAGCTCG
TAAATTCGTATTTGTGCGTGATACGGCAAGAAGAGTACATATATATATATGTCCGTTGTGCGTCTATATCCACGCTGCACTTGTCACTCGACATACACGCGTCGAGC

2 micron origin

7,920 7,940 7,960 7,980 8,000 8,020

CGTTGCATTTTCGGAAGCGCTCGTTTTCGGAAACGCTTTGAAGTTCCTATTCCGAAGTTCCTATTCTCTAGAAAGTATAGGAACTTCAGAGCGCTTTTGAAAACCAA
GCAACGTAAAAGCCTTCGCGAGCAAAAGCCTTTGCGAAACTTCAAGGATAAGGCTTCAAGGATAAGAGATCTTTCATATCCTTGAAGTCTCGCGAAAACTTTTGGTT

FRT

2 micron origin

8,040 8,060 8,080 8,100 8,120

AAGCGCTCTGAAGACGCACTTTCAAAAAACCAAAAACGCACCGGACTGTAACGAGCTACTAAAATATTGCGAATACCGCTTCCACAAACATTGCTCAAAAGTATCTC
TTCGCGAGACTTCTGCGTGAAAGTTTTTTGGTTTTTGCGTGGCCTGACATTGCTCGATGATTTTATAACGCTTATGGCGAAGGTGTTTGTAACGAGTTTTCATAGAG

2 micron origin

8,140 8,160 8,180 8,200 8,220

TTTGCTATATATCTCTGTGCTATATCCCTATATAACCTACCCATCCACCTTTCGCTCCTTGAACTTGCATCTAAACTCGACCTCTACATTTTTTATGTTTATCTCTA
AAACGATATATAGAGACACGATATAGGGATATATTGGATGGGTAGGTGGAAAGCGAGGAACTTGAACGTAGATTTGAGCTGGAGATGTAAAAAATACAAATAGAGAT

2 micron origin

8,240 8,260 8,280 8,300 8,320 8,340

GTATTACTCTTTAGACAAAAAAATTGTAGTAAGAACTATTCATAGAGTGAATCGAAAACAATACGAAAATGTAAACATTTCCTATACGTAGTATATAGAGACAAAAT
CATAATGAGAAATCTGTTTTTTTAACATCATTCTTGATAAGTATCTCACTTAGCTTTTGTTATGCTTTTACATTTGTAAAGGATATGCATCATATATCTCTGTTTTA

2 micron origin

8,360 8,380 8,400 8,420 8,440

AGAAGAAACCGTTCATAATTTTCTGACCAATGAAGAATCATCAACGCTATCACTTTCTGTTCACAAAGTATGCGCAATCCACATCGGTATAGAATATAATCGGGGAT
TCTTCTTTGGCAAGTATTAAAAGACTGGTTACTTCTTAGTAGTTGCGATAGTGAAAGACAAGTGTTTCATACGCGTTAGGTGTAGCCATATCTTATATTAGCCCCTA

2 micron origin

8,460 8,480 8,500 8,520 8,540 8,560

GCCTTTATCTTGAAAAAATGCACCCGCAGCTTCGCTAGTAATCAGTAAACGCGGGAAGTGGAGTCAGGCTTTTTTTATGGAAGAGAAAATAGACACCAAAGTAGCCT
CGGAAATAGAACTTTTTTACGTGGGCGTCGAAGCGATCATTAGTCATTTGCGCCCTTCACCTCAGTCCGAAAAAAATACCTTCTCTTTTATCTGTGGTTTCATCGGA

2 micron origin

8,580 8,600 8,620 8,640 8,660

TCTTCTAACCTTAACGGACCTACAGTGCAAAAAGTTATCAAGAGACTGCATTATAGAGCGCACAAAGGAGAAAAAAAGTAATCTAAGATGCTTTGTTAGAAAAATAG
AGAAGATTGGAATTGCCTGGATGTCACGTTTTTCAATAGTTCTCTGACGTAATATCTCGCGTGTTTCCTCTTTTTTTCATTAGATTCTACGAAACAATCTTTTTATC

2 micron origin

8,680 8,700 8,720 8,740 8,760

CGCTCTCGGGATGCATTTTTGTAGAACAAAAAAGAAGTATAGATTCTTTGTTGGTAAAATAGCGCTCTCGCGTTGCATTTCTGTTCTGTAAAAATGCAGCTCAGATT
GCGAGAGCCCTACGTAAAAACATCTTGTTTTTTCTTCATATCTAAGAAACAACCATTTTATCGCGAGAGCGCAACGTAAAGACAAGACATTTTTACGTCGAGTCTAA

2 micron origin

8,780 8,800 8,820 8,840 8,860 8,880

CTTTGTTTGAAAAATTAGCGCTCTCGCGTTGCATTTTTGTTTTACAAAAATGAAGCACAGATTCTTCGTTGGTAAAATAGCGCTTTCGCGTTGCATTTCTGTTCTGT
GAAACAAACTTTTTAATCGCGAGAGCGCAACGTAAAAACAAAATGTTTTTACTTCGTGTCTAAGAAGCAACCATTTTATCGCGAAAGCGCAACGTAAAGACAAGACA

2 micron origin

8,900 8,920 8,940 8,960 8,980

```
AAAAATGCAGCTCAGATTCTTTGTTTGAAAAATTAGCGCTCTCGCGTTGCATTTTTGTTCTACAAAATGAAGCACAGATGCTTCGTTCAGGTGGCACTTTTCGGGGA
TTTTTACGTCGAGTCTAAGAAACAAACTTTTTAATCGCGAGAGCGCAACGTAAAAACAAGATGTTTTACTTCGTGTCTACGAAGCAAGTCCACCGTGAAAAGCCCCT
                                        2 micron origin
       9,000                9,020                9,040                9,060                9,080

AATGTGCGCGGAACCCCTATTTGTTTATTTTTCTAAATACATTCAAATATGTATCCGCTCATGAGACAATAACCCTGATAAATGCTTCAATAATATTGAAAAAGGAA
TTACACGCGCCTTGGGGATAAACAAATAAAAAGATTTATGTAAGTTTATACATAGGCGAGTACTCTGTTATTGGGACTATTTACGAAGTTATTATAACTTTTTCCTT
   9,100                9,120                9,140                9,160                9,180                9,200

GAGTATGAGTATTCAACATTTCCGTGTCGCCCTTATTCCCTTTTTTGCGGCATTTTGCCTTCCTGTTTTTGCTCACCCAGAAACGCTGGTGAAAGTAAAAGATGCTG
CTCATACTCATAAGTTGTAAAGGCACAGCGGGAATAAGGGAAAAAACGCCGTAAAACGGAAGGACAAAAACGAGTGGGTCTTTGCGACCACTTTCATTTTCTACGAC
            9,220                9,240                9,260                9,280                9,300

AAGATCAGTTGGGTGCACGAGTGGGTTACATCGAACTGGATCTCAACAGCGGTAAGATCCTTGAGAGTTTTCGCCCCGAAGAACGTTTTCCAATGATGAGCACTTTT
TTCTAGTCAACCCACGTGCTCACCCAATGTAGCTTGACCTAGAGTTGTCGCCATTCTAGGAACTCTCAAAAGCGGGGCTTCTTGCAAAAGGTTACTACTCGTGAAAA
                                                                                                 AmpR
      9,320                9,340                9,360                9,380                9,400

AAAGTTCTGCTATGTGGCGCGGTATTATCCCGTATTGACGCCGGGCAAGAGCAACTCGGTCGCCGCATACACTATTCTCAGAATGACTTGGTTGAGTACTCACCAGT
TTTCAAGACGATACACCGCGCCATAATAGGGCATAACTGCGGCCCGTTCTCGTTGAGCCAGCGGCGTATGTGATAAGAGTCTTACTGAACCAACTCATGAGTGGTCA
                                            AmpR
 9,420                9,440                9,460                9,480                9,500                9,520

CACAGAAAAGCATCTTACGGATGGCATGACAGTAAGAGAATTATGCAGTGCTGCCATAACCATGAGTGATAACACTGCGGCCAACTTACTTCTGACAACGATCGGAG
GTGTCTTTTCGTAGAATGCCTACCGTACTGTCATTCTCTTAATACGTCACGACGGTATTGGTACTCACTATTGTGACGCCGGTTGAATGAAGACTGTTGCTAGCCTC
                                            AmpR
           9,540                9,560                9,580                9,600                9,620

GACCGAAGGAGCTAACCGCTTTTTTGCACAACATGGGGGATCATGTAACTCGCCTTGATCGTTGGGAACCGGAGCTGAATGAAGCCATACCAAACGACGAGCGTGAC
CTGGCTTCCTCGATTGGCGAAAAAACGTGTTGTACCCCCTAGTACATTGAGCGGAACTAGCAACCCTTGGCCTCGACTTACTTCGGTATGGTTTGCTGCTCGCACTG
                                            AmpR
      9,640                9,660                9,680                9,700                9,720

ACCACGATGCCTGTAGCAATGGCAACAACGTTGCGCAAACTATTAACTGGCGAACTACTTACTCTAGCTTCCCGGCAACAATTAATAGACTGGATGGAGGCGGATAA
TGGTGCTACGGACATCGTTACCGTTGTTGCAACGCGTTTGATAATTGACCGCTTGATGAATGAGATCGAAGGGCCGTTGTTAATTATCTGACCTACCTCCGCCTATT
                                            AmpR
 9,740                9,760                9,780                9,800                9,820                9,840

AGTTGCAGGACCACTTCTGCGCTCGGCCCTTCCGGCTGGCTGGTTTATTGCTGATAAATCTGGAGCCGGTGAGCGTGGGTCTCGCGGTATCATTGCAGCACTGGGGC
TCAACGTCCTGGTGAAGACGCGAGCCGGGAAGGCCGACCGACCAAATAACGACTATTTAGACCTCGGCCACTCGCACCCAGAGCGCCATAGTAACGTCGTGACCCCG
                                            AmpR
            9,860                9,880                9,900                9,920                9,940
```

CAGATGGTAAGCCCTCCCGTATCGTAGTTATCTACACGACGGGGAGTCAGGCAACTATGGATGAACGAAATAGACAGATCGCTGAGATAGGTGCCTCACTGATTAAG
GTCTACCATTCGGGAGGGCATAGCATCAATAGATGTGCTGCCCCTCAGTCCGTTGATACCTACTTGCTTTATCTGTCTAGCGACTCTATCCACGGAGTGACTAATTC

AmpR

9,960 9,980 10,000 10,020 10,040

CATTGGTAACTGTCAGACCAAGTTTACTCATATATACTTTAGATTGATTTAAAACTTCATTTTTAATTTAAAAGGATCTAGGTGAAGATCCTTTTTGATAATCTCAT
GTAACCATTGACAGTCTGGTTCAAATGAGTATATATGAAATCTAACTAAATTTTGAAGTAAAAATTAAATTTTCCTAGATCCACTTCTAGGAAAAACTATTAGAGTA

10,060 10,080 10,100 10,120 10,140 10,160

GACCAAAATCCCTTAACGTGAGTTTTCGTTCCACTGAGCGTCAGACCCCGTAGAAAAGATCAAAGGATCTTCTTGAGATCCTTTTTTTCTGCGCGTAATCTGCTGCT
CTGGTTTTAGGGAATTGCACTCAAAAGCAAGGTGACTCGCAGTCTGGGGCATCTTTTCTAGTTTCCTAGAAGAACTCTAGGAAAAAAAGACGCGCATTAGACGACGA

10,180 10,200 10,220 10,240 10,260

TGCAAACAAAAAAACCACCGCTACCAGCGGTGGTTTGTTTGCCGGATCAAGAGCTACCAACTCTTTTTCCGAAGGTAACTGGCTTCAGCAGAGCGCAGATACCAAAT
ACGTTTGTTTTTTTGGTGGCGATGGTCGCCACCAAACAAACGGCCTAGTTCTCGATGGTTGAGAAAAAGGCTTCCATTGACCGAAGTCGTCTCGCGTCTATGGTTTA

10,280 10,300 10,320 10,340 10,360

ACTGTTCTTCTAGTGTAGCCGTAGTTAGGCCACCACTTCAAGAACTCTGTAGCACCGCCTACATACCTCGCTCTGCTAATCCTGTTACCAGTGGCTGCTGCCAGTGG
TGACAAGAAGATCACATCGGCATCAATCCGGTGGTGAAGTTCTTGAGACATCGTGGCGGATGTATGGAGCGAGACGATTAGGACAATGGTCACCGACGACGGTCACC

10,400 10,420 10,440 10,460 10,480

CGATAAGTCGTGTCTTACCGGGTTGGACTCAAGACGATAGTTACCGGATAAGGCGCAGCGGTCGGGCTGAACGGGGGGTTCGTGCACACAGCCCAGCTTGGAGCGAA
GCTATTCAGCACAGAATGGCCCAACCTGAGTTCTGCTATCAATGGCCTATTCCGCGTCGCCAGCCCGACTTGCCCCCCAAGCACGTGTGTCGGGTCGAACCTCGCTT

10,500 10,520 10,540 10,560 10,580

CGACCT
GCTGGA

10,595

ST/SC ligation / pAnchor_P.C. of immunostaining...

ACACCGAACTGAGATACCTACAGCGTGAGCTATGAGAAAGCGCCACGCTTCCCGAAGGGAGAAAGGCGGACAGGTATCCGGTAAGCGGCAGGGTCGGAACAGGAGAG
TGTGGCTTGACTCTATGGATGTCGCACTCGATACTCTTTCGCGGTGCGAAGGGCTTCCCTCTTTCCGCCTGTCCATAGGCCATTCGCCGTCCCAGCCTTGTCCTCTC

20 40 60 80 100

CGCACGAGGGAGCTTCCAGGGGGAAACGCCTGGTATCTTTATAGTCCTGTCGGGTTTCGCCACCTCTGACTTGAGCGTCGATTTTTGTGATGCTCGTCAGGGGGGCG
GCGTGCTCCCTCGAAGGTCCCCCTTTGCGGACCATAGAAATATCAGGACAGCCCAAAGCGGTGGAGACTGAACTCGCAGCTAAAAACACTACGAGCAGTCCCCCCGC

120 140 160 180 200

GAGCCTATGGAAAAACGCCAGCAACGCGGCCTTTTTACGGTTCCTGGCCTTTTGCTGGCCTTTTGCTCACATGTTCTTTCCTGCGTTATCCCCTGATTCTGTGGATA
CTCGGATACCTTTTTGCGGTCGTTGCGCCGGAAAAATGCCAAGGACCGGAAAACGACCGGAAAACGAGTGTACAAGAAAGGACGCAATAGGGGACTAAGACACCTAT

220 240 260 280 300 320

ACCGTATTACCGCCTTTGAGTGAGCTGATACCGCTCGCCGCAGCCGAACGACCGAGCGCAGCGAGTCAGTGAGCGAGGAAGCGGAAGAGCGCCCAATACGCAAACCG
TGGCATAATGGCGGAAACTCACTCGACTATGGCGAGCGGCGTCGGCTTGCTGGCTCGCGTCGCTCAGTCACTCGCTCCTTCGCCTTCTCGCGGGTTATGCGTTTGGC

340 360 380 400 420

CCTCTCCCCGCGCGTTGGCCGATTCATTAATGCAGCTGGCACGACAGGTTTCCCGACTGGAAAGCGGGCAGTGAGCGCAACGCAATTAATGTGAGTTAGCTCACTCA
GGAGAGGGGCGCGCAACCGGCTAAGTAATTACGTCGACCGTGCTGTCCAAAGGGCTGACCTTTCGCCCGTCACTCGCGTTGCGTTAATTACACTCAATCGAGTGAGT

440 460 480 500 520

TTAGGCACCCCAGGCTTTACACTTTATGCTTCCGGCTCGTATGTTGTGTGGAATTGTGAGCGGATAACAATTTCACACAGGAAACAGCTATGACCATGATTACGCCA
AATCCGTGGGGTCCGAAATGTGAAATACGAAGGCCGAGCATACAACACACCTTAACACTCGCCTATTGTTAAAGTGTGTCCTTTGTCGATACTGGTACTAATGCGGT

lac promoter LacO M13-rev

540 560 580 600 620 640

AGCGCGCAATTAACCCTCACTAAAGGGAACAAAAGCTGGAGCTAGTATACTCTTTCTTCAACAATTAAATACTCTCGGTAGCCAAGTTGGTTTAAGGCGCAAGACTG
TCGCGCGTTAATTGGGAGTGATTTCCCTTGTTTTCGACCTCGATCATATGAGAAAGAAGTTGTTAATTTATGAGAGCCATCGGTTCAACCAAATTCCGCGTTCTGAC

T3
T3 promoter

660 680 700 720 740

TAATTTATCACTACGAAATCTTGAGATCGGGCGTTCGACTCGCCCCCGGGAGAGATGGCCGGCATGGTCCCAGCCTCCTCGCTGGCGCCGGCTGGGCAACACCTTCG
ATTAAATAGTGATGCTTTAGAACTCTAGCCCGCAAGCTGAGCGGGGGCCCTCTCTACCGGCCGTACCAGGGTCGGAGGAGCGACCGCGGCCGACCCGTTGTGGAAGC

760 780 800 820 840

GGTGGCGAATGGGACTTTTTATGTGCGTATTGCTTTCAGTTTTAGAGCTAGAAATAGCAAGTTAAAATAAGGCTAGTCCGTTATCAACTTGAAAAAGTGGCACCGAG
CCACCGCTTACCCTGAAAAATACACGCATAACGAAAGTCAAAATCTCGATCTTTATCGTTCAATTTTATTCCGATCAGGCAATAGTTGAACTTTTTCACCGTGGCTC

860 880 900 920 940 960

TCGGTGCTTTTTTTATTTTTTGTCACTATTGTTATGTAAAATGCCACCTCTGACAGTATGGAACGCAAACTTCTGTCTAGTGGATAGTCGACAAGCTTACCAGTTCT
AGCCACGAAAAAAATAAAAAACAGTGATAACAATACATTTTACGGTGGAGACTGTCATACCTTGCGTTTGAAGACAGATCACCTATCAGCTGTTCGAATGGTCAAGA

GPD ...ter

980 1,000 1,020 1,040 1,060

CACACGGAACACCACTAATGGACACAAATTCGAAATACTTTGACCCTATTTTCGAGGACCTTGTCACCTTGAGCCCAAGAGAGCCAAGATTTAAATTTTCCTATGAC
GTGTGCCTTGTGGTGATTACCTGTGTTTAAGCTTTATGAAACTGGGATAAAAGCTCCTGGAACAGTGGAACTCGGGTTCTCTCGGTTCTAAATTTAAAAGGATACTG
GPD promoter
1,080 1,100 1,120 1,140 1,160

TTGATGCAAATTCCCAAAGCTAATAACATGCAAGACACGTACGGTCAAGAAGACATATTTGACCTCTTAACAGGTTCAGACGCGACTGCCTCATCAGTAAGACCCGT
AACTACGTTTAAGGGTTTCGATTATTGTACGTTCTGTGCATGCCAGTTCTTCTGTATAAACTGGAGAATTGTCCAAGTCTGCGCTGACGGAGTAGTCATTCTGGGCA
GPD promoter
1,180 1,200 1,220 1,240 1,260 1,280

TGAAAAGAACTTACCTGAAAAAAACGAATATATACTAGCGTTGAATGTTAGCGTCAACAACAAGAAGTTTAATGACGCGGAGGCCAAGGCAAAAAGATTCCTTGATT
ACTTTTCTTGAATGGACTTTTTTTGCTTATATATGATCGCAACTTACAATCGCAGTTGTTGTTCTTCAAATTACTGCGCCTCCGGTTCCGTTTTTCTAAGGAACTAA
GPD promoter
1,300 1,320 1,340 1,360 1,380

ACGTAAGGGAGTTAGAATCATTTTGAATAAAAAACACGCTTTTTCAGTTCGAGTTTATCATTATCAATACTGCCATTTCAAAGAATACGTAAATAATTAATAGTAGT
TGCATTCCCTCAATCTTAGTAAAACTTATTTTTTGTGCGAAAAAGTCAAGCTCAAATAGTAATAGTTATGACGGTAAAGTTTCTTATGCATTTATTAATTATCATCA
GPD promoter
1,400 1,420 1,440 1,460 1,480

GATTTTCCTAACTTTATTTAGTCAAAAAATTAGCCTTTTAATTCTGCTGTAACCCGTACATGCCCAAAATAGGGGGCGGGTTACACAGAATATATAACATCGTAGGT
CTAAAAGGATTGAAATAAATCAGTTTTTTAATCGGAAAATTAAGACGACATTGGGCATGTACGGGTTTTATCCCCCGCCCAATGTGTCTTATATATTGTAGCATCCA
GPD promoter
1,500 1,520 1,540 1,560 1,580 1,600

GTCTGGGTGAACAGTTTATTCCTGGCATCCACTAAATATAATGGAGCCCGCTTTTTAAGCTGGCATCCAGAAAAAAAAAGAATCCCAGCACCAAAATATTGTTTTCT
CAGACCCACTTGTCAAATAAGGACCGTAGGTGATTTATATTACCTCGGGCGAAAAATTCGACCGTAGGTCTTTTTTTTTCTTAGGGTCGTGGTTTTATAACAAAAGA
GPD promoter
1,620 1,640 1,660 1,680 1,700

TCACCAACCATCAGTTCATAGGTCCATTCTCTTAGCGCAACTACAGAGAACAGGGGCACAAACAGGCAAAAAACGGGCACAACCTCAATGGAGTGATGCAACCTGCC
AGTGGTTGGTAGTCAAGTATCCAGGTAAGAGAATCGCGTTGATGTCTCTTGTCCCCGTGTTTGTCCGTTTTTTGCCCGTGTTGGAGTTACCTCACTACGTTGGACGG
GPD promoter
1,720 1,740 1,760 1,780 1,800

TGGAGTAAATGATGACACAAGGCAATTGACCCACGCATGTATCTATCTCATTTTCTTACACCTTCTATTACCTTCTGCTCTCTCTGATTTGGAAAAAGCTGAAAAAA
ACCTCATTTACTACTGTGTTCCGTTAACTGGGTGCGTACATAGATAGAGTAAAAGAATGTGGAAGATAATGGAAGACGAGAGAGACTAAACCTTTTTCGACTTTTTT
GPD promoter
1,820 1,840 1,860 1,880 1,900 1,920

AAGGTTGAAACCAGTTCCCTGAAATTATTCCCCTACTTGACTAATAAGTATATAAAGACGGTAGGTATTGATTGTAATTCTGTAAATCTATTTCTTAAACTTCTTAA
TTCCAACTTTGGTCAAGGGACTTTAATAAGGGGATGAACTGATTATTCATATATTTCTGCCATCCATAACTAACATTAAGACATTTAGATAAAGAATTTGAAGAATT

GPD promoter

1,940 1,960 1,980 2,000 2,020

ATTCTACTTTTATAGTTAGTCTTTTTTTTAGTTTTAAAACACCAGAACTTAGTTTCGACGGATTCTAGAACTAGTGGATCCATGAGATTCCCATCTATCTTCACCGC
TAAGATGAAAATATCAATCAGAAAAAAAATCAAAATTTTGTGGTCTTGAATCAAAGCTGCCTAAGATCTTGATCACCTAGGTACTCTAAGGGTAGATAGAAGTGGCG

1 2 3 4 5 6 7 8 9
M R F P S I F T A

GPD promoter
Mating fac...ha leader

2,040 2,060 2,080 2,100 2,120 2,140

TGTTTTGTTCGCTGCTTCTTCTGCTTTGGCTGCTCCAGCTAACACCACCACCGAAGACGAAACCGCTCAAATCCCAGCTGAAGCTGTTATCGACTACTCTGACTTGG
ACAAAACAAGCGACGAAGAAGACGAAACCGACGAGGTCGATTGTGGTGGTGGCTTCTGCTTTGGCGAGTTTAGGGTCGACTTCGACAATAGCTGATGAGACTGAACC

10 11 12 13 14 15 16 17 18 19 20 21 22 23 24 25 26 27 28 29 30 31 32 33 34 35 36 37 38 39 40 41 42 43 44
V L F A A S S A L A A P A N T T T E D E T A Q I P A E A V I D Y S D L

Mating factor alpha leader

2,160 2,180 2,200 2,220 2,240

AAGGTGACTTCGACGCTGCTGCTTTGCCATTGTCTAACTCTACCAACAACGGTTTGTCTTCTACCAACACCACCATCGCTTCTATCGCTGCTAAGGAAGAAGGTGTT
TTCCACTGAAGCTGCGACGACGAAACGGTAACAGATTGAGATGGTTGTTGCCAAACAGAAGATGGTTGTGGTGGTAGCGAAGATAGCGACGATTCCTTCTTCCACAA

45 46 47 48 49 50 51 52 53 54 55 56 57 58 59 60 61 62 63 64 65 66 67 68 69 70 71 72 73 74 75 76 77 78 79 80
E G D F D A A A L P L S N S T N N G L S S T N T T I A S I A A K E E G V

Mating factor alpha leader

2,260 2,280 2,300 2,320 2,340

CAATTGGACAAGAGAGAAagatctggcggccgcgcatgcgctagcctcgagGATGTGCAGTTGCAAGCATCCGGCGGCGGTTCTGTTCAGGCTGGTGGTTCTCTAAG
GTTAACCTGTTCTCTCTTtctagaccgccggcgcgtacgcgatcggagctcCTACACGTCAACGTTCGTAGGCCGCCGCCAAGACAAGTCCGACCACCAAGAGATTC

81 82 83 84 85 86
Q L D K R E

2 4 6 8 10 12 14 16 18
D V Q L Q A S G G G S V Q A G G S L R

Mating...eader
anti-lysozyme nanobody

2,360 2,380 2,400 2,420 2,440 2,460

ATTAAGTTGTGCTGCTTCAGGTTATACCATCGGCCCATACTGCATGGGTTGGTTTCGTCAAGCTCCAGGCAAAGAAAGGGAGGGAGTCGCAGCCATTAACATGGGCG
TAATTCAACACGACGAAGTCCAATATGGTAGCCGGGTATGACGTACCCAACCAAAGCAGTTCGAGGTCCGTTTCTTTCCCTCCCTCAGCGTCGGTAATTGTACCCGC

20 22 24 26 28 30 32 34 36 38 40 42 44 46 48 50 52 54
L S C A A S G Y T I G P Y C M G W F R Q A P G K E R E G V A A I N M G

anti-lysozyme nanobody

2,480 2,500 2,520 2,540 2,560

GCGGTATCACATATTATGCAGACTCCGTAAAAGGTAGATTTACTATCTCTCAGGACAATGCTAAGAACACGGTCTATCTGTTAATGAATTCATTGGAGCCCGAGGAC
CGCCATAGTGTATAATACGTCTGAGGCATTTTCCATCTAAATGATAGAGAGTCCTGTTACGATTCTTGTGCCAGATAGACAATTACTTAAGTAACCTCGGGCTCCTG
56 58 60 62 64 66 68 70 72 74 76 78 80 82 84 86 88 90
G G I T Y Y A D S V K G R F T I S Q D N A K N T V Y L L M N S L E P E D
anti-lysozyme nanobody
2,580 2,600 2,620 2,640 2,660

ACTGCCATATACTACTGTGCCGCAGATTCAACCATTTACGCTTCCTATTACGAATGCGGACACGGATTAAGTACTGGAGGTTACGGTTATGATTCCTGGGGTCAAGG
TGACGGTATATGATGACACGGCGTCTAAGTTGGTAAATGCGAAGGATAATGCTTACGCCTGTGCCTAATTCATGACCTCCAATGCCAATACTAAGGACCCCAGTTCC
92 94 96 98 100 102 104 106 108 110 112 114 116 118 120 122 124 126
T A I Y Y C A A D S T I Y A S Y Y E C G H G L S T G G Y G Y D S W G Q G
anti-lysozyme nanobody
2,680 2,700 2,720 2,740 2,760 2,780

CACACAAGTCACGGTATCATCAGGCGGATCAGACTATAAGGATGACGACGATAAGGCTGGAGGTTCGAGGGGAGTCCCTCACATAGTGATGGTGGATGCTTACAAAA
GTGTGTTCAGTGCCATAGTAGTCCGCCTAGTCTGATATTCCTACTGCTGCTATTCCGACCTCCAAGCTCCCCTCAGGGAGTGTATCACTACCACCTACGAATGTTTT
128 130 132 1 2 3 1 2 3 4 5 6 7 8 1 2 3 4 1 2 3 4 5 6 7 8 9 10 11 12 13
T Q V T V S S G G S D Y K D D D D K A G G S R G V P H I V M V D A Y K
anti-lys...anobody FLAG tag SpyTag
2,800 2,820 2,840 2,860 2,880

GGTATAAAGTAACTACCTTGTCTGGCTTATCAGGGGAACAGGGTCCTAGCGGTGACATGACGACAGAGGAGGACAGTGCGACCCACATAAAGTTTAGCAAAAGAGAT
CCATATTTCATTGATGGAACAGACCGAATAGTCCCCTTGTCCCAGGATCGCCACTGTACTGCTGTCTCCTCCTGTCACGCTGGGTGTATTTCAAATCGTTTTCTCTA
14 15 16 2 4 6 8 10 12 14 16 18 20 22 24 26 28 30 32
R Y K V T T L S G L S G E Q G P S G D M T T E E D S A T H I K F S K R D
SpyCatcher
2,900 2,920 2,940 2,960 2,980

GAAGATGGAAGGGAACTAGCTGGAGCGACCATGGAACTTCGTGACAGCTCAGGGAAGACTATATCAACGTGGATCTCAGATGGTCACGTCAAGGACTTCTATTTGTA
CTTCTACCTTCCCTTGATCGACCTCGCTGGTACCTTGAAGCACTGTCGAGTCCCTTCTGATATAGTTGCACCTAGAGTCTACCAGTGCAGTTCCTGAAGATAAACAT
34 36 38 40 42 44 46 48 50 52 54 56 58 60 62 64 66 68
E D G R E L A G A T M E L R D S S G K T I S T W I S D G H V K D F Y L Y
SpyCatcher
3,000 3,020 3,040 3,060 3,080 3,100

CCCCGGAAAGTACACATTTGTGGAAACCGCCGCGCCGGATGGCTACGAGGTAGCGACCCCGATAGAATTCACCGTTAACGAGGACGGCCAAGTCACCGTAGATGGAG
GGGGCCTTTCATGTGTAAACACCTTTGGCGGCGCGGCCTACCGATGCTCCATCGCTGGGGCTATCTTAAGTGGCAATTGCTCCTGCCGGTTCAGTGGCATCTACCTC
70 72 74 76 78 80 82 84 86 88 90 92 94 96 98 100 102 104
P G K Y T F V E T A A P D G Y E V A T P I E F T V N E D G Q V T V D G
SpyCatcher
3,120 3,140 3,160 3,180 3,200

```
AGGCGACAGAGGGTGATGCCCACACTGGGGGCTCCGGCGGATCTGGTGGGAGCGGATCCTACCCATACGATGTTCCAGATTACGCTCAAACATCCTCACCCTCTACA
TCCGCTGTCTCCCACTACGGGTGTGACCCCCGAGGCCGCCTAGACCACCCTCGCCTAGGATGGGTATGCTACAAGGTCTAATGCGAGTTTGTAGGAGTGGGAGATGT
  106   108   110   112    1  2  3  4  5  6  7  8  9  10 11  1  2  3  4  5  6  7  8  9     2     4     6
 E  A  T  E  G  D  A  H  T  G  G  S  G  G  S  G  G  S  G  S  Y  P  Y  D  V  P  D  Y  A  Q  T  S  S  P  S  T
SpyCatcher                                                   HA tag                    649 stalk
        3,220               3,240               3,260               3,280               3,300
```

```
GAAGCATCAGTATCAACCTCTAGTACCTCTTCCTCTGCTTCACAATCATCTGACCCAACTACAACATCTTCGTCCAGTTCATCGTCTTCCCCATCTTCCCAATCTGA
CTTCGTAGTCATAGTTGGAGATCATGGAGAAGGAGACGAAGTGTTAGTAGACTGGGTTGATGTTGTAGAAGCAGGTCAAGTAGCAGAAGGGGTAGAAGGGTTAGACT
 8     10    12    14    16    18    20    22    24    26    28    30    32    34    36    38    40    42
 E  A  S  V  S  T  S  S  T  S  S  S  A  S  Q  S  S  D  P  T  T  T  S  S  S  S  S  S  S  S  P  S  S  Q  S  E
649 stalk
  3,320               3,340               3,360               3,380               3,400               3,420
```

```
GGAAATTTCATCATCACCGACGGTCTCAACTACACCATCAACTTCTTCATCATCTTCCTCAATGACTTCAACCACCACAACAAAGTCAATCTCAACTTCCACTACAA
CCTTTAAAGTAGTAGTGGCTGCCAGAGTTGATGTGGTAGTTGAAGAAGTAGTAGAAGGAGTTACTGAAGTTGGTGGTGTTGTTTCAGTTAGAGTTGAAGGTGATGTT
  44    46    48    50    52    54    56    58    60    62    64    66    68    70    72    74    76    78
  E  I  S  S  S  P  T  V  S  T  T  P  S  T  S  S  S  S  S  S  M  T  S  T  T  T  T  K  S  I  S  T  S  T  T
649 stalk
            3,440               3,460               3,480               3,500               3,520
```

```
GTTCAGCTCCAGTTACAGATGTGACAGTTTCCTCATCGCCTAGTAAATCTACCTCTACTTCGACAAGTACAGAAACATCTAAAACACCTACTTCAATGACAGAGTAT
CAAGTCGAGGTCAATGTCTACACTGTCAAAGGAGTAGCGGATCATTTAGATGGAGATGAAGCTGTTCATGTCTTTGTAGATTTTGTGGATGAAGTTACTGTCTCATA
  80    82    84    86    88    90    92    94    96    98    100   102   104   106   108   110   112   114
 S S  A  P  V  T  D  V  T  V  S  S  S  P  S  K  S  T  S  T  S  T  S  T  E  T  S  K  T  P  T  S  M  T  E  Y
649 stalk
      3,540               3,560               3,580               3,600               3,620
```

```
ACATCTAGTACATCGATAATTTCGACTCCAGTTAGTCACTCGCAGACAGGTTTGTCGGCTTCATCAAGTTCATCATCTACAACATCCGGTTCTTCGTCCACTAAATC
TGTAGATCATGTAGCTATTAAAGCTGAGGTCAATCAGTGAGCGTCTGTCCAAACAGCCGAAGTAGTTCAAGTAGTAGATGTTGTAGGCCAAGAAGCAGGTGATTTAG
   116   118   120   122   124   126   128   130   132   134   136   138   140   142   144   146   148   150
 T  S  S  T  S  I  I  S  T  P  V  S  H  S  Q  T  G  L  S  A  S  S  S  S  S  S  T  T  S  G  S  S  S  T  K  S
649 stalk
 3,640               3,660               3,680               3,700               3,720               3,740
```

```
AGAAAGTTCGACAACATCTGGCTCTTCCCAGTCCGTGGAATCAACCTCCAGCCACGCCACTGTTCTTGCTAATTCCGCAGAAATGGTCACAACATCCTCTAGTTCAT
TCTTTCAAGCTGTTGTAGACCGAGAAGGGTCAGGCACCTTAGTTGGAGGTCGGTGCGGTGACAAGAACGATTAAGGCGTCTTTACCAGTGTTGTAGGAGATCAAGTA
    152   154   156   158   160   162   164   166   168   170   172   174   176   178   180   182   184
  E  S  S  T  T  S  G  S  S  Q  S  V  E  S  T  S  S  H  A  T  V  L  A  N  S  A  E  M  V  T  T  S  S  S  S
649 stalk
            3,760               3,780               3,800               3,820               3,840
```

```
CCTCAACATCCGAAATGTCATTAACTAGTACTGCTACCAGTGTACCAGTCTCATCTAGTAGCAGTACGACATATTCTACTAGCGCATCTACACAAGCCGTCACTACA
GGAGTTGTAGGCTTTACAGTAATTGATCATGACGATGGTCACATGGTCAGAGTAGATCATCGTCATGCTGTATAAGATGATCGCGTAGATGTGTTCGGCAGTGATGT
186  188  190  192  194  196  198  200  202  204  206  208  210  212  214  216  218  220
S S T S E M S L T S T A T S V P V S S S S S T T Y S T S A S T Q A V T T
649 stalk
3,860  3,880  3,900  3,920  3,940

ACATCTTCTTCCACTGTATCTACAACTTCTTCTAGTACAACGTTAACAAGCGCATTCACACATTCTTCAACCACATCGTCCGACCAGCCACCCAGCGACACTACAAG
TGTAGAAGAAGGTGACATAGATGTTGAAGAAGATCATGTTGCAATTGTTCGCGTAAGTGTGTAAGAAGTTGGTGTAGCAGGCTGGTCGGTGGGTCGCTGTGATGTTC
222  224  226  228  230  232  234  236  238  240  242  244  246  248  250  252  254  256
T S S S T V S T T S S S T T L T S A F T H S S T T S S D Q P P S D T T S
649 stalk
3,960  3,980  4,000  4,020  4,040  4,060

TCCATCTACGACACACGAACCTCATGTAACCACTCAGACGTCATCAGAAACATCTTCTTCTAAGTCATCTTCTACTTCTTCTTCAAGTACATCTCAAACCTCTGAGT
AGGTAGATGCTGTGTGCTTGGAGTACATTGGTGAGTCTGCAGTAGTCTTTGTAGAAGAAGATTCAGTAGAAGATGAAGAAGAAGTTCATGTAGAGTTTGGAGACTCA
258  260  262  264  266  268  270  272  274  276  278  280  282  284  286  288  290  292
P S T T H E P H V T T Q T S S E T S S S K S S S T S S S S T S Q T S E
649 stalk
4,080  4,100  4,120  4,140  4,160

CTGCAACACCATCCGATTCCGTATCACCTGGAAGTTCTACATCAACATCTTCTAGTAGCACTTCTACTTCCACTTCTATTTCCAGTGGAGAAACGACAACTTCTTCT
GACGTTGTGGTAGGCTAAGGCATAGTGGACCTTCAAGATGTAGTTGTAGAAGATCATCGTGAAGATGAAGGTGAAGATAAAGGTCACCTCTTTGCTGTTGAAGAAGA
294  296  298  300  302  304  306  308  310  312  314  316  318  320  322  324  326  328
S A T P S D S V S P G S S T S T S S S S T S T S T S I S S G E T T T S S
649 stalk
4,180  4,200  4,220  4,240  4,260  4,280

TCTTCATCATCTGCCACGACCACTTCTAACAGCGCAACCTTGTCAGTCTCTACCACACAAACTTCGATTGAAGCCAGTTCATCTACTACATCTACATCTAGTTCAAC
AGAAGTAGTAGACGGTGCTGGTGAAGATTGTCGCGTTGGAACAGTCAGAGATGGTGTGTTTGAAGCTAACTTCGGTCAAGTAGATGATGTAGATGTAGATCAAGTTG
330  332  334  336  338  340  342  344  346  348  350  352  354  356  358  360  362  364
S S S S A T T T S N S A T L S V S T T Q T S I E A S S S T T S T S S S T
649 stalk
4,300  4,320  4,340  4,360  4,380

AATTACAACTTCAAGTAGTAGCGCTCACATATCGTCGAAATCTCAATCTAGTATTACCTATCCCTCTTCCTCGACATCTTCATCTACATCGTCCTCAATTTCTAGCG
TTAATGTTGAAGTTCATCATCGCGAGTGTATAGCAGCTTTAGAGTTAGATCATAATGGATAGGGAGAAGGAGCTGTAGAAGTAGATGTAGCAGGAGTTAAAGATCGC
366  368  370  372  374  376  378  380  382  384  386  388  390  392  394  396  398
I T T S S S S A H I S S K S Q S S I T Y P S S S T S S S T S S S I S S
649 stalk
4,400  4,420  4,440  4,460  4,480
```

```
AATCTGAAAGTTTTGAATCGACATCAGCAGAAGATGCTCCATCAACAGCACCTTCATCAAGTGTCTCTTCTAAGAGTTCTACCTCTACAACATCAAGCACATCGACA
TTAGACTTTCAAAACTTAGCTGTAGTCGTCTTCTACGAGGTAGTTGTCGTGGAAGTAGTTCACAGAGAAGATTCTCAAGATGGAGATGTTGTAGTTCGTGTAGCTGT
400  402  404  406  408  410  412  414  416  418  420  422  424  426  428  430  432  434
E S E S F E S T S A E D A P S T A P S S S V S S K S S T S T T S S T S T
649 stalk
4,500  4,520  4,540  4,560  4,580  4,600

TCTTCAAGCACTCCATCTCCATCACCATCTTCCGTGAGTTCTTCCTCCACCAGCTCATTGACAACTTCTGCTGTATCAACACCAGCTACCTCTCATTCTCAAAGTAC
AGAAGTTCGTGAGGTAGAGGTAGTGGTAGAAGGCACTCAAGAAGGAGGTGGTCGAGTAACTGTTGAAGACGACATAGTTGTGGTCGATGGAGAGTAAGAGTTTCATG
436  438  440  442  444  446  448  450  452  454  456  458  460  462  464  466  468  470
S S S T P S P S P S S V S S S S T S S L T T S A V S T P A T S H S Q S T
649 stalk
4,620  4,640  4,660  4,680  4,700

TGTAGTAACCACCACTACTATTACTACATCAACAGGTCCAGTGATGTCTACGACAACAGCTTATTCTTCTAGTTCTACTAGCAGCTCGGAATCTTCTGAGGTTCAGT
ACATCATTGGTGGTGATGATAATGATGTAGTTGTCCAGGTCACTACAGATGCTGTTGTCGAATAAGAAGATCAAGATGATCGTCGAGCCTTAGAAGACTCCAAGTCA
472  474  476  478  480  482  484  486  488  490  492  494  496  498  500  502  504  506
V V T T T T I T T S T G P V M S T T T A Y S S S S T S S S E S S E V Q
649 stalk
4,720  4,740  4,760  4,780  4,800

CTGTCATGTCATCTACGCCTAGTTCAACATCAACAACAACCAGTTCGGAATCTACTTCATCTAGCTCCACAGCTTCTACCTCACCATCAACCTCGCAAACTTTCGAA
GACAGTACAGTAGATGCGGATCAAGTTGTAGTTGTTGTTGGTCAAGCCTTAGATGAAGTAGATCGAGGTGTCGAAGATGGAGTGGTAGTTGGAGCGTTTGAAAGCTT
508  510  512  514  516  518  520  522  524  526  528  530  532  534  536  538  540  542
S V M S S T P S S T S T T T S S E S T S S S S T A S T S P S T S Q T F E
649 stalk
4,820  4,840  4,860  4,880  4,900  4,920

ACTTCTCCTACTATAGGAGGTGTCCCCTCAACCACTTCATTTGTCTCTACGCCAACAACGAAATTGTCGCACACTACTTCCACTATGACAGCACAGTCCGATAGTAA
TGAAGAGGATGATATCCTCCACAGGGGAGTTGGTGAAGTAAACAGAGATGCGGTTGTTGCTTTAACAGCGTGTGATGAAGGTGATACTGTCGTGTCAGGCTATCATT
544  546  548  550  552  554  556  558  560  562  564  566  568  570  572  574  576  578
T S P T I G G V P S T T S F V S T P T T K L S H T T S T M T A Q S D S K
649 stalk
4,940  4,960  4,980  5,000  5,020

GTCTACCCACTCCTCAAGCACATCGACAGAAGATAAATCATCCACTGCTTCTGCAGTTGACGAAAGCACTACAACATCCACTTCCACGGAGTCTACTACATCAGTAA
CAGATGGGTGAGGAGTTCGTGTAGCTGTCTTCTATTTAGTAGGTGACGAAGACGTCAACTGCTTTCGTGATGTTGTAGGTGAAGGTGCCTCAGATGATGTAGTCATT
580  582  584  586  588  590  592  594  596  598  600  602  604  606  608  610  612
S T H S S S T S T E D K S S T A S A V D E S T T T S T S T E S T T S V
649 stalk
5,040  5,060  5,080  5,100  5,120
```

CATCAGGCACCTCCCATTCCGCTAAAGAATCTTCGTCAAATTCTAAGGTGTATAGTTCACAGACAGCACACTCATCCATAAGTGTTGCATCATCACCTAGTACAAAG
GTAGTCCGTGGAGGGTAAGGCGATTTCTTAGAAGCAGTTTAAGATTCCACATATCAAGTGTCTGTCGTGTGAGTAGGTATTCACAACGTAGTAGTGGATCATGTTTC
614 616 618 620 622 624 626 628 630 632 634 636 638 640 642 644 646 648
T S G T S H S A K E S S S N S K V Y S S Q T A H S S I S V A S S P S T K
649 stalk
5,140 5,160 5,180 5,200 5,220 5,240

GGCGCCCAAATCCAATCTTCTATGGTTGAAATCTCTACCTACGCTGGTTCTGCTAACTCTGTTAACGCTGGTGCTGGTGCTGGTGCTTTGTTCTTGTTGTTGTCTTT
CCGCGGGTTTAGGTTAGAAGATACCAACTTTAGAGATGGATGCGACCAAGACGATTGAGACAATTGCGACCACGACCACGACCACGAAACAAGAACAACAACAGAAA
650 652 654 656 658 660 662 664 666 668 670 672 674 676 678 680 682 684
G A Q I Q S S M V E I S T Y A G S A N S V N A G A G A G A L F L L L S L
GPI anchor
5,260 5,280 5,300 5,320 5,340

GGCTATCATCTAATGATTAATTAACTCGAGATCTGATAACAACAGTGTAGATGTAACAAAATCGACTTTGTTCCCACTGTACTTTTAGCTCGTACAAAATACAATAT
CCGATAGTAGATTACTAATTAATTGAGCTCTAGACTATTGTTGTCACATCTACATTGTTTTAGCTGAAACAAGGGTGACATGAAAATCGAGCATGTTTTATGTTATA
686 688 1
A I I *
GP...r
alpha factor terminator
5,360 5,380 5,400 5,420 5,440

ACTTTTCATTTCTCCGTAAACAACATGTTTTCCCATGTAATATCCTTTTCTATTTTTCGTTCCGTTACCAACTTTACACATACTTTATATAGCTATTCACTTCTATA
TGAAAAGTAAAGAGGCATTTGTTGTACAAAAGGGTACATTATAGGAAAAGATAAAAAGCAAGGCAATGGTTGAAATGTGTATGAAATATATCGATAAGTGAAGATAT
alpha factor terminator
5,460 5,480 5,500 5,520 5,540 5,560

CACTAAAAAACTAAGACAATTTTAATTTTGCTGCCTGCCATATTTCAATTTGTTATAAATTCCTATAATTTATCCTATTAGTAGCTAAAAAAAGATGAATGTGAATC
GTGATTTTTTGATTCTGTTAAAATTAAAACGACGGACGGTATAAAGTTAAACAATATTTAAGGATATTAAATAGGATAATCATCGATTTTTTTCTACTTACACTTAG
alpha factor terminator
5,580 5,600 5,620 5,640 5,660

GAATCCTAAGAGAATTGGCCGGTACCCAATTCGCCCTATAGTGAGTCGTATTACGCGCGCTCACTGGCCGTCGTTTTACAACGTCGTGACTGGGAAAACCCTGGCGT
CTTAGGATTCTCTTAACCGGCCATGGGTTAAGCGGGATATCACTCAGCATAATGCGCGCGAGTGACCGGCAGCAAAATGTTGCAGCACTGACCCTTTTGGGACCGCA
alpha...ator T7 M13-fwd
5,680 5,700 5,720 5,740 5,760

TACCCAACTTAATCGCCTTGCAGCACATCCCCCTTTCGCCAGCTGGCGTAATAGCGAAGAGGCCCGCACCGATCGCCCTTCCCAACAGTTGCGCAGCCTGAATGGCG
ATGGGTTGAATTAGCGGAACGTCGTGTAGGGGGAAAGCGGTCGACCGCATTATCGCTTCTCCGGGCGTGGCTAGCGGGAAGGGTTGTCAACGCGTCGGACTTACCGC
LacZ alpha
5,780 5,800 5,820 5,840 5,860 5,880

```
AATGGACGCGCCCTGTAGCGGCGCATTAAGCGCGGCGGGTGTGGTGGTTACGCGCAGCGTGACCACTACACTTGCCAGCGCCCTAGCGCCCGCTCCTTTCGCTTTCT
TTACCTGCGCGGGACATCGCCGCGTAATTCGCGCCGCCCACACCACCAATGCGCGTCGCACTGGTGATGTGAACGGTCGCGGGATCGCGGGCGAGGAAAGCGAAAGA
          5,900               5,920               5,940               5,960               5,980

TCCCTTCCTTTCTCGCCACGTTCGCCGGCTTTCCCCGTCAAGCTCTAAATCGGGGGCTCCCTTTAGGGTTCCGATTTAGTGCTTTACGGCACCTCGACCCCAAAAAA
AGGGAAGGAAAGAGCGGTGCAAGCGGCCGAAAGGGGCAGTTCGAGATTTAGCCCCCGAGGGAAATCCCAAGGCTAAATCACGAAATGCCGTGGAGCTGGGGTTTTTT
       6,000               6,020               6,040               6,060               6,080

CTTGATTAGGGTGATGGTTCACGTAGTGGGCCATCGCCCTGATAGACGGTTTTTCGCCCTTTGACGTTGGAGTCCACGTTCTTTAATAGTGGACTCTTGTTCCAAAC
GAACTAATCCCACTACCAAGTGCATCACCCGGTAGCGGGACTATCTGCCAAAAAGCGGGAAACTGCAACCTCAGGTGCAAGAAATTATCACCTGAGAACAAGGTTTG
6,100               6,120               6,140               6,160               6,180               6,200

TGGAACAACACTCAACCCTATCTCGGTCTATTCTTTTGATTTATAAGGGATTTTGCCGATTTCGGCCTATTGGTTAAAAAATGAGCTGATTTAACAAAAATTTAACG
ACCTTGTTGTGAGTTGGGATAGAGCCAGATAAGAAAACTAAATATTCCCTAAAACGGCTAAAGCCGGATAACCAATTTTTTACTCGACTAAATTGTTTTTAAATTGC
             6,220               6,240               6,260               6,280               6,300

CGAATTTTAACAAAATATTAACGCTTACAATTTCCTGATGCGGTATTTTCTCCTTACGCATCTGTGCGGTATTTCACACCGCATAGGGTAATAACTGATATAATTAA
GCTTAAAATTGTTTTATAATTGCGAATGTTAAAGGACTACGCCATAAAAGAGGAATGCGTAGACACGCCATAAAGTGTGGCGTATCCCATTATTGACTATATTAATT
      6,320               6,340               6,360               6,380               6,400               6,420

ATTGAAGCTCTAATTTGTGAGTTTAGTATACATGCATTTACTTATAATACAGTTTTTTAAGCAAGGATTTTCTTAACTTCTTCGGCGACAGCATCACCGACTTCGGT
TAACTTCGAGATTAAACACTCAAATCATATGTACGTAAATGAATATTATGTCAAAAAATTCGTTCCTAAAAGAATTGAAGAAGCCGCTGTCGTAGTGGCTGAAGCCA
                                                                    LEU2
                   6,440               6,460               6,480               6,500               6,520

GGTACTGTTGGAACCACCTAAATCACCAGTTCTGATACCTGCATCCAAAACCTTTTTAACTGCATCTTCAATGGCCTTACCTTCTTCAGGCAAGTTCAATGACAATT
CCATGACAACCTTGGTGGATTTAGTGGTCAAGACTATGGACGTAGGTTTTGGAAAAATTGACGTAGAAGTTACCGGAATGGAAGAAGTCCGTTCAAGTTACTGTTAA
                                                       LEU2
            6,540               6,560               6,580               6,600               6,620

TCAACATCATTGCAGCAGACAAGATAGTGGCGATAGGGTTGACCTTATTCTTTGGCAAATCTGGAGCAGAACCGTGGCATGGTTCGTACAAACCAAATGCGGTGTTC
AGTTGTAGTAACGTCGTCTGTTCTATCACCGCTATCCCAACTGGAATAAGAAACCGTTTAGACCTCGTCTTGGCACCGTACCAAGCATGTTTGGTTTACGCCACAAG
                                                       LEU2
     6,640               6,660               6,680               6,700               6,720               6,740

TTGTCTGGCAAAGAGGCCAAGGACGCAGATGGCAACAAACCCAAGGAACCTGGGATAACGGAGGCTTCATCGGAGATGATATCACCAAACATGTTGCTGGTGATTAT
AACAGACCGTTTCTCCGGTTCCTGCGTCTACCGTTGTTTGGGTTCCTTGGACCCTATTGCCTCCGAAGTAGCCTCTACTATAGTGGTTTGTACAACGACCACTAATA
                                                       LEU2
                  6,760               6,780               6,800               6,820               6,840
```

AATACCATTTAGGTGGGTTGGGTTCTTAACTAGGATCATGGCGGCAGAATCAATCAATTGATGTTGAACCTTCAATGTAGGGAATTCGTTCTTGATGGTTTCCTCCA
TTATGGTAAATCCACCCAACCCAAGAATTGATCCTAGTACCGCCGTCTTAGTTAGTTAACTACAACTTGGAAGTTACATCCCTTAAGCAAGAACTACCAAAGGAGGT
LEU2
6,860 6,880 6,900 6,920 6,940

CAGTTTTTCTCCATAATCTTGAAGAGGCCAAAACATTAGCTTTATCCAAGGACCAAATAGGCAATGGTGGCTCATGTTGTAGGGCCATGAAAGCGGCCATTCTTGTG
GTCAAAAAGAGGTATTAGAACTTCTCCGGTTTTGTAATCGAAATAGGTTCCTGGTTTATCCGTTACCACCGAGTACAACATCCCGGTACTTTCGCCGGTAAGAACAC
LEU2
6,960 6,980 7,000 7,020 7,040 7,060

ATTCTTTGCACTTCTGGAACGGTGTATTGTTCACTATCCCAAGCGACACCATCACCATCGTCTTCCTTTCTCTTACCAAAGTAAATACCTCCCACTAATTCTCTGAC
TAAGAAACGTGAAGACCTTGCCACATAACAAGTGATAGGGTTCGCTGTGGTAGTGGTAGCAGAAGGAAAGAGAATGGTTTCATTTATGGAGGGTGATTAAGAGACTG
LEU2
7,080 7,100 7,120 7,140 7,160

AACAACGAAGTCAGTACCTTTAGCAAATTGTGGCTTGATTGGAGATAAGTCTAAAAGAGAGTCGGATGCAAAGTTACATGGTCTTAAGTTGGCGTACAATTGAAGTT
TTGTTGCTTCAGTCATGGAAATCGTTTAACACCGAACTAACCTCTATTCAGATTTTCTCTCAGCCTACGTTTCAATGTACCAGAATTCAACCGCATGTTAACTTCAA
LEU2
7,180 7,200 7,220 7,240 7,260

CTTTACGGATTTTTAGTAAACCTTGTTCAGGTCTAACACTACCGGTACCCCATTTAGGACCACCCACAGCACCTAACAAAACGGCATCAGCCTTCTTGGAGGCTTCC
GAAATGCCTAAAAATCATTTGGAACAAGTCCAGATTGTGATGGCCATGGGGTAAATCCTGGTGGGTGTCGTGGATTGTTTTGCCGTAGTCGGAAGAACCTCCGAAGG
LEU2
7,280 7,300 7,320 7,340 7,360 7,380

AGCGCCTCATCTGGAAGTGGAACACCTGTAGCATCGATAGCAGCACCACCAATTAAATGATTTTCGAAATCGAACTTGACATTGGAACGAACATCAGAAATAGCTTT
TCGCGGAGTAGACCTTCACCTTGTGGACATCGTAGCTATCGTCGTGGTGGTTAATTTACTAAAAGCTTTAGCTTGAACTGTAACCTTGCTTGTAGTCTTTATCGAAA
LEU2
7,400 7,420 7,440 7,460 7,480

AAGAACCTTAATGGCTTCGGCTGTGATTTCTTGACCAACGTGGTCACCTGGCAAAACGACGATCTTCTTAGGGGCAGACATTAGAATGGTATATCCTTGAAATATAT
TTCTTGGAATTACCGAAGCCGACACTAAAGAACTGGTTGCACCAGTGGACCGTTTTGCTGCTAGAAGAATCCCCGTCTGTAATCTTACCATATAGGAACTTTATATA
LEU2
7,500 7,520 7,540 7,560 7,580

ATATATATATNTNGCTGAAATGTAAAAGGTAAGAAAAGTTAGAAAGTAAGACGATTGCTAACCACCTATTGGAAAAAAACAATAGGTCCTTAAATAATATTGTCAACT
TATATATATANANCGACTTTACATTTTCCATTCTTTTCAATCTTTCATTCTGCTAACGATTGGTGGATAACCTTTTTTTGTTATCCAGGAATTTATTATAACAGTTGA
7,600 7,620 7,640 7,660 7,680 7,700

TCAAGTATTGTGATGCAAGCATTTAGTCATGAACGCTTCTCTATTCTATATGAAAAGCCGGTTCCGGCGCTCTCACCTTTCCTTTTTCTCCCAATTTTTCAGTTGAA
AGTTCATAACACTACGTTCGTAAATCAGTACTTGCGAAGAGATAAGATATACTTTTCGGCCAAGGCCGCGAGAGTGGAAAGGAAAAAGAGGGTTAAAAAGTCAACTT
7,720 7,740 7,760 7,780 7,800

```
AAAGGTATATGCGTCAGGCGACCTCTGAAATTAACAAAAAATTTCCAGTCATCGAATTTGATTCTGTGCGATAGCGCCCCTGTGTGTTCTCGTTATGTTGAGGAAAA
TTTCCATATACGCAGTCCGCTGGAGACTTTAATTGTTTTTTAAAGGTCAGTAGCTTAAACTAAGACACGCTATCGCGGGGACACACAAGAGCAATACAACTCCTTTT
      7,820               7,840               7,860               7,880               7,900

AAATAATGGTTGCTAAGAGATTCGAACTCTTGCATCTTACGATACCTGAGTATTCCCACAGTTTGAAAAGCTGTGGTATGGTGCACTCTCAGTACAATCTGCTCTGA
TTTATTACCAACGATTCTCTAAGCTTGAGAACGTAGAATGCTATGGACTCATAAGGGTGTCAAACTTTTCGACACCATACCACGTGAGAGTCATGTTAGACGAGACT
7,920               7,940               7,960               7,980               8,000               8,020

TGCCGCATAGTTAAGCCAGCCCCGACACCCGCCAACACCCGCTGACGCGCCCTGACGGGCTTGTCTGCTCCCGGCATCCGCTTACAGACAAGCTGTGACCGTCTCCG
ACGGCGTATCAATTCGGTCGGGGCTGTGGGCGGTTGTGGGCGACTGCGCGGGACTGCCCGAACAGACGAGGGCCGTAGGCGAATGTCTGTTCGACACTGGCAGAGGC
             8,040               8,060               8,080               8,100               8,120

GGAGCTGCATGTGTCAGAGGTTTTCACCGTCATCACCGAAACGCGCGAGACGAAAGGGCCTCGTGATACGCCTATTTTTATAGGTTAATGTCATGATAATAATGGTT
CCTCGACGTACACAGTCTCCAAAAGTGGCAGTAGTGGCTTTGCGCGCTCTGCTTTCCCGGAGCACTATGCGGATAAAAATATCCAATTACAGTACTATTATTACCAA
      8,140               8,160               8,180               8,200               8,220

TCTTAGTATGATCCAATATCAAAGGAAATGATAGCATTGAAGGATGAGACTAATCCAATTGAGGAGTGGCAGCATATAGAACAGCTAAAGGGTAGTGCTGAAGGAAG
AGAATCATACTAGGTTATAGTTTCCTTTACTATCGTAACTTCCTACTCTGATTAGGTTAACTCCTCACCGTCGTATATCTTGTCGATTTCCCATCACGACTTCCTTC
       <======================================= 2 micron origin ======================================<<
8,240               8,260               8,280               8,300               8,320               8,340

CATACGATACCCCGCATGGAATGGGATAATATCACAGGAGGTACTAGACTACCTTTCATCCTACATAAATAGACGCATATAAGTACGCATTTAAGCATAAACACGCA
GTATGCTATGGGGCGTACCTTACCCTATTATAGTGTCCTCCATGATCTGATGGAAAGTAGGATGTATTTATCTGCGTATATTCATGCGTAAATTCGTATTTGTGCGT
<<===================================== 2 micron origin ========================================<<
            8,360               8,380               8,400               8,420               8,440

CTATGCCGTTCTTCTCATGTATATATATATATACAGGCAACACGCAGATATAGGTGCGACGTGAACAGTGAGCTGTATGTGCGCAGCTCGCGTTGCATTTTCGGAAGCG
GATACGGCAAGAAGAGTACATATATATATATGTCCGTTGTGCGTCTATATCCACGCTGCACTTGTCACTCGACATACACGCGTCGAGCGCAACGTAAAAGCCTTCGC
<<===================================== 2 micron origin ========================================<<
    8,460               8,480               8,500               8,520               8,540               8,560

CTCGTTTTCGGAAACGCTTTGAAGTTCCTATTCCGAAGTTCCTATTCTCTAGAAAGTATAGGAACTTCAGAGCGCTTTTGAAAACCAAAAGCGCTCTGAAGACGCAC
GAGCAAAAGCCTTTGCGAAACTTCAAGGATAAGGCTTCAAGGATAAGAGATCTTTCATATCCTTGAAGTCTCGCGAAAACTTTTGGTTTTCGCGAGACTTCTGCGTG
                               ============ FRT ============>
<<===================================== 2 micron origin ========================================<<
                8,580               8,600               8,620               8,640               8,660

TTTCAAAAAACCAAAAACGCACCGGACTGTAACGAGCTACTAAAATATTGCGAATACCGCTTCCACAAACATTGCTCAAAAGTATCTCTTTGCTATATATCTCTGTG
AAAGTTTTTTGGTTTTTGCGTGGCCTGACATTGCTCGATGATTTTATAACGCTTATGGCGAAGGTGTTTGTAACGAGTTTTCATAGAGAAACGATATATAGAGACAC
<<===================================== 2 micron origin ========================================<<
          8,680               8,700               8,720               8,740               8,760
```

CTATATCCCTATATAACCTACCCATCCACCTTTCGCTCCTTGAACTTGCATCTAAACTCGACCTCTACATTTTTTATGTTTATCTCTAGTATTACTCTTTAGACAAA
GATATAGGGATATATTGGATGGGTAGGTGGAAAGCGAGGAACTTGAACGTAGATTTGAGCTGGAGATGTAAAAAATACAAATAGAGATCATAATGAGAAATCTGTTT
2 micron origin
8,780 8,800 8,820 8,840 8,860 8,880

AAAATTGTAGTAAGAACTATTCATAGAGTGAATCGAAAACAATACGAAAATGTAAACATTTCCTATACGTAGTATATAGAGACAAAATAGAAGAAACCGTTCATAAT
TTTTAACATCATTCTTGATAAGTATCTCACTTAGCTTTTGTTATGCTTTTACATTTGTAAAGGATATGCATCATATATCTCTGTTTTATCTTCTTTGGCAAGTATTA
2 micron origin
8,900 8,920 8,940 8,960 8,980

TTTCTGACCAATGAAGAATCATCAACGCTATCACTTTCTGTTCACAAAGTATGCGCAATCCACATCGGTATAGAATATAATCGGGGATGCCTTTATCTTGAAAAAAT
AAAGACTGGTTACTTCTTAGTAGTTGCGATAGTGAAAGACAAGTGTTTCATACGCGTTAGGTGTAGCCATATCTTATATTAGCCCCTACGGAAATAGAACTTTTTTA
2 micron origin
9,000 9,020 9,040 9,060 9,080

GCACCCGCAGCTTCGCTAGTAATCAGTAAACGCGGGAAGTGGAGTCAGGCTTTTTTTATGGAAGAGAAAATAGACACCAAAGTAGCCTTCTTCTAACCTTAACGGAC
CGTGGGCGTCGAAGCGATCATTAGTCATTTGCGCCCTTCACCTCAGTCCGAAAAAAATACCTTCTCTTTTATCTGTGGTTTCATCGGAAGAAGATTGGAATTGCCTG
2 micron origin
9,100 9,120 9,140 9,160 9,180 9,200

CTACAGTGCAAAAAGTTATCAAGAGACTGCATTATAGAGCGCACAAAGGAGAAAAAAAGTAATCTAAGATGCTTTGTTAGAAAAATAGCGCTCTCGGGATGCATTTT
GATGTCACGTTTTTCAATAGTTCTCTGACGTAATATCTCGCGTGTTTCCTCTTTTTTTCATTAGATTCTACGAAACAATCTTTTTATCGCGAGAGCCCTACGTAAAA
2 micron origin
9,220 9,240 9,260 9,280 9,300

TGTAGAACAAAAAAGAAGTATAGATTCTTTGTTGGTAAAATAGCGCTCTCGCGTTGCATTTCTGTTCTGTAAAAATGCAGCTCAGATTCTTTGTTTGAAAAAATTAGC
ACATCTTGTTTTTTCTTCATATCTAAGAAACAACCATTTTATCGCGAGAGCGCAACGTAAAGACAAGACATTTTTACGTCGAGTCTAAGAAACAAACTTTTTAATCG
2 micron origin
9,320 9,340 9,360 9,380 9,400

GCTCTCGCGTTGCATTTTTGTTTTACAAAAATGAAGCACAGATTCTTCGTTGGTAAAATAGCGCTTTCGCGTTGCATTTCTGTTCTGTAAAAATGCAGCTCAGATTC
CGAGAGCGCAACGTAAAAACAAAATGTTTTTACTTCGTGTCTAAGAAGCAACCATTTTATCGCGAAAGCGCAACGTAAAGACAAGACATTTTTACGTCGAGTCTAAG
2 micron origin
9,420 9,440 9,460 9,480 9,500 9,520

TTTGTTTGAAAAATTAGCGCTCTCGCGTTGCATTTTTGTTCTACAAAATGAAGCACAGATGCTTCGTTCAGGTGGCACTTTTCGGGGAAATGTGCGCGGAACCCCTA
AAACAAACTTTTTAATCGCGAGAGCGCAACGTAAAAACAAGATGTTTTACTTCGTGTCTACGAAGCAAGTCCACCGTGAAAAGCCCCTTTACACGCGCCTTGGGGAT
2 micron origin
9,540 9,560 9,580 9,600 9,620

TTTGTTTATTTTTCTAAATACATTCAAATATGTATCCGCTCATGAGACAATAACCCTGATAAATGCTTCAATAATATTGAAAAAGGAAGAGTATGAGTATTCAACAT
AAACAAATAAAAAGATTTATGTAAGTTTATACATAGGCGAGTACTCTGTTATTGGGACTATTTACGAAGTTATTATAACTTTTTCCTTCTCATACTCATAAGTTGTA
9,640 9,660 9,680 9,700 9,720

```
TTCCGTGTCGCCCTTATTCCCTTTTTTGCGGCATTTTGCCTTCCTGTTTTTGCTCACCCAGAAACGCTGGTGAAAGTAAAAGATGCTGAAGATCAGTTGGGTGCACG
AAGGCACAGCGGGAATAAGGGAAAAAACGCCGTAAAACGGAAGGACAAAAACGAGTGGGTCTTTGCGACCACTTTCATTTTCTACGACTTCTAGTCAACCCACGTGC
9,740    9,760    9,780    9,800    9,820    9,840

AGTGGGTTACATCGAACTGGATCTCAACAGCGGTAAGATCCTTGAGAGTTTTCGCCCCGAAGAACGTTTTCCAATGATGAGCACTTTTAAAGTTCTGCTATGTGGCG
TCACCCAATGTAGCTTGACCTAGAGTTGTCGCCATTCTAGGAACTCTCAAAAGCGGGGCTTCTTGCAAAAGGTTACTACTCGTGAAAATTTCAAGACGATACACCGC
AmpR
9,860    9,880    9,900    9,920    9,940

CGGTATTATCCCGTATTGACGCCGGGCAAGAGCAACTCGGTCGCCGCATACACTATTCTCAGAATGACTTGGTTGAGTACTCACCAGTCACAGAAAAGCATCTTACG
GCCATAATAGGGCATAACTGCGGCCCGTTCTCGTTGAGCCAGCGGCGTATGTGATAAGAGTCTTACTGAACCAACTCATGAGTGGTCAGTGTCTTTTCGTAGAATGC
AmpR
9,960    9,980    10,000    10,020    10,040

GATGGCATGACAGTAAGAGAATTATGCAGTGCTGCCATAACCATGAGTGATAACACTGCGGCCAACTTACTTCTGACAACGATCGGAGGACCGAAGGAGCTAACCGC
CTACCGTACTGTCATTCTCTTAATACGTCACGACGGTATTGGTACTCACTATTGTGACGCCGGTTGAATGAAGACTGTTGCTAGCCTCCTGGCTTCCTCGATTGGCG
AmpR
10,060    10,080    10,100    10,120    10,140    10,160

TTTTTTGCACAACATGGGGGATCATGTAACTCGCCTTGATCGTTGGGAACCGGAGCTGAATGAAGCCATACCAAACGACGAGCGTGACACCACGATGCCTGTAGCAA
AAAAAACGTGTTGTACCCCCTAGTACATTGAGCGGAACTAGCAACCCTTGGCCTCGACTTACTTCGGTATGGTTTGCTGCTCGCACTGTGGTGCTACGGACATCGTT
AmpR
10,180    10,200    10,220    10,240    10,260

TGGCAACAACGTTGCGCAAACTATTAACTGGCGAACTACTTACTCTAGCTTCCCGGCAACAATTAATAGACTGGATGGAGGCGGATAAAGTTGCAGGACCACTTCTG
ACCGTTGTTGCAACGCGTTTGATAATTGACCGCTTGATGAATGAGATCGAAGGGCCGTTGTTAATTATCTGACCTACCTCCGCCTATTTCAACGTCCTGGTGAAGAC
AmpR
10,280    10,300    10,320    10,340    10,360

CGCTCGGCCCTTCCGGCTGGCTGGTTTATTGCTGATAAATCTGGAGCCGGTGAGCGTGGGTCTCGCGGTATCATTGCAGCACTGGGGCCAGATGGTAAGCCCTCCCG
GCGAGCCGGGAAGGCCGACCGACCAAATAACGACTATTTAGACCTCGGCCACTCGCACCCAGAGCGCCATAGTAACGTCGTGACCCCGGTCTACCATTCGGGAGGGC
AmpR
10,400    10,420    10,440    10,460    10,480

TATCGTAGTTATCTACACGACGGGGAGTCAGGCAACTATGGATGAACGAAATAGACAGATCGCTGAGATAGGTGCCTCACTGATTAAGCATTGGTAACTGTCAGACC
ATAGCATCAATAGATGTGCTGCCCCTCAGTCCGTTGATACCTACTTGCTTTATCTGTCTAGCGACTCTATCCACGGAGTGACTAATTCGTAACCATTGACAGTCTGG
AmpR
10,500    10,520    10,540    10,560    10,580

AAGTTTACTCATATATACTTTAGATTGATTTAAAACTTCATTTTTAATTTAAAAGGATCTAGGTGAAGATCCTTTTTGATAATCTCATGACCAAAATCCCTTAACGT
TTCAAATGAGTATATATGAAATCTAACTAAATTTTGAAGTAAAAATTAAATTTTCCTAGATCCACTTCTAGGAAAAACTATTAGAGTACTGGTTTTAGGGAATTGCA
10,600    10,620    10,640    10,660    10,680    10,700
```

```
GAGTTTTCGTTCCACTGAGCGTCAGACCCCGTAGAAAAGATCAAAGGATCTTCTTGAGATCCTTTTTTTCTGCGCGTAATCTGCTGCTTGCAAACAAAAAAACCACC
CTCAAAAGCAAGGTGACTCGCAGTCTGGGGCATCTTTTCTAGTTTCCTAGAAGAACTCTAGGAAAAAAAGACGCGCATTAGACGACGAACGTTTGTTTTTTTGGTGG
         10,720              10,740              10,760              10,780              10,800

GCTACCAGCGGTGGTTTGTTTGCCGGATCAAGAGCTACCAACTCTTTTTCCGAAGGTAACTGGCTTCAGCAGAGCGCAGATACCAAATACTGTTCTTCTAGTGTAGC
CGATGGTCGCCACCAAACAAACGGCCTAGTTCTCGATGGTTGAGAAAAAGGCTTCCATTGACCGAAGTCGTCTCGCGTCTATGGTTTATGACAAGAAGATCACATCG
      10,820              10,840              10,860              10,880              10,900

CGTAGTTAGGCCACCACTTCAAGAACTCTGTAGCACCGCCTACATACCTCGCTCTGCTAATCCTGTTACCAGTGGCTGCTGCCAGTGGCGATAAGTCGTGTCTTACC
GCATCAATCCGGTGGTGAAGTTCTTGAGACATCGTGGCGGATGTATGGAGCGAGACGATTAGGACAATGGTCACCGACGACGGTCACCGCTATTCAGCACAGAATGG
10,920              10,940              10,960              10,980              11,000              11,020

GGGTTGGACTCAAGACGATAGTTACCGGATAAGGCGCAGCGGTCGGGCTGAACGGGGGGTTCGTGCACACAGCCCAGCTTGGAGCGAACGACCT
CCCAACCTGAGTTCTGCTATCAATGGCCTATTCCGCGTCGCCAGCCCGACTTGCCCCCCAAGCACGTGTGTCGGGTCGAACCTCGCTTGCTGGA
   11,030    11,040    11,050    11,060    11,070    11,080    11,090    11,100    11,110
```

ST/SC ligation / pAnchor_P.C. of ST/SC coupling...

```
ACACCGAACTGAGATACCTACAGCGTGAGCTATGAGAAAGCGCCACGCTTCCCGAAGGGAGAAAGGCGGACAGGTATCCGGTAAGCGGCAGGGTCGGAACAGGAGAG
TGTGGCTTGACTCTATGGATGTCGCACTCGATACTCTTTCGCGGTGCGAAGGGCTTCCCTCTTTCCGCCTGTCCATAGGCCATTCGCCGTCCCAGCCTTGTCCTCTC
20 40 60 80 100

CGCACGAGGGAGCTTCCAGGGGGAAACGCCTGGTATCTTTATAGTCCTGTCGGGTTTCGCCACCTCTGACTTGAGCGTCGATTTTTGTGATGCTCGTCAGGGGGGCG
GCGTGCTCCCTCGAAGGTCCCCCTTTGCGGACCATAGAAATATCAGGACAGCCCAAAGCGGTGGAGACTGAACTCGCAGCTAAAAACACTACGAGCAGTCCCCCCGC
120 140 160 180 200

GAGCCTATGGAAAAACGCCAGCAACGCGGCCTTTTTACGGTTCCTGGCCTTTTGCTGGCCTTTTGCTCACATGTTCTTTCCTGCGTTATCCCCTGATTCTGTGGATA
CTCGGATACCTTTTTGCGGTCGTTGCGCCGGAAAAATGCCAAGGACCGGAAAACGACCGGAAAACGAGTGTACAAGAAAGGACGCAATAGGGGACTAAGACACCTAT
220 240 260 280 300 320

ACCGTATTACCGCCTTTGAGTGAGCTGATACCGCTCGCCGCAGCCGAACGACCGAGCGCAGCGAGTCAGTGAGCGAGGAAGCGGAAGAGCGCCCAATACGCAAACCG
TGGCATAATGGCGGAAACTCACTCGACTATGGCGAGCGGCGTCGGCTTGCTGGCTCGCGTCGCTCAGTCACTCGCTCCTTCGCCTTCTCGCGGGTTATGCGTTTGGC
340 360 380 400 420

CCTCTCCCCGCGCGTTGGCCGATTCATTAATGCAGCTGGCACGACAGGTTTCCCGACTGGAAAGCGGGCAGTGAGCGCAACGCAATTAATGTGAGTTAGCTCACTCA
GGAGAGGGGCGCGCAACCGGCTAAGTAATTACGTCGACCGTGCTGTCCAAAGGGCTGACCTTTCGCCCGTCACTCGCGTTGCGTTAATTACACTCAATCGAGTGAGT
440 460 480 500 520

TTAGGCACCCCAGGCTTTACACTTTATGCTTCCGGCTCGTATGTTGTGTGGAATTGTGAGCGGATAACAATTTCACACAGGAAACAGCTATGACCATGATTACGCCA
AATCCGTGGGGTCCGAAATGTGAAATACGAAGGCCGAGCATACAACACACCTTAACACTCGCCTATTGTTAAAGTGTGTCCTTTGTCGATACTGGTACTAATGCGGT
lac promoter LacO M13-rev
540 560 580 600 620 640

AGCGCGCAATTAACCCTCACTAAAGGGAACAAAAGCTGGAGCTAGTATACTCTTTCTTCAACAATTAAATACTCTCGGTAGCCAAGTTGGTTTAAGGCGCAAGACTG
TCGCGCGTTAATTGGGAGTGATTTCCCTTGTTTTCGACCTCGATCATATGAGAAAGAAGTTGTTAATTTATGAGAGCCATCGGTTCAACCAAATTCCGCGTTCTGAC
T3
T3 promoter
660 680 700 720 740

TAATTTATCACTACGAAATCTTGAGATCGGGCGTTCGACTCGCCCCCGGGAGAGATGGCCGGCATGGTCCCAGCCTCCTCGCTGGCGCCGGCTGGGCAACACCTTCG
ATTAAATAGTGATGCTTTAGAACTCTAGCCCGCAAGCTGAGCGGGGGCCCTCTCTACCGGCCGTACCAGGGTCGGAGGAGCGACCGCGGCCGACCCGTTGTGGAAGC
760 780 800 820 840

GGTGGCGAATGGGACTTTTTATGTGCGTATTGCTTTCAGTTTTAGAGCTAGAAATAGCAAGTTAAAATAAGGCTAGTCCGTTATCAACTTGAAAAAGTGGCACCGAG
CCACCGCTTACCCTGAAAAATACACGCATAACGAAAGTCAAAATCTCGATCTTTATCGTTCAATTTTATTCCGATCAGGCAATAGTTGAACTTTTTCACCGTGGCTC
860 880 900 920 940 960

TCGGTGCTTTTTTTATTTTTTGTCACTATTGTTATGTAAAATGCCACCTCTGACAGTATGGAACGCAAACTTCTGTCTAGTGGATAGTCGACAAGCTTACCAGTTCT
AGCCACGAAAAAAATAAAAAACAGTGATAACAATACATTTTACGGTGGAGACTGTCATACCTTGCGTTTGAAGACAGATCACCTATCAGCTGTTCGAATGGTCAAGA
GPD ...ter
980 1,000 1,020 1,040 1,060
```

CACACGGAACACCACTAATGGACACAAATTCGAAATACTTTGACCCTATTTTCGAGGACCTTGTCACCTTGAGCCCAAGAGAGCCAAGATTTAAATTTTCCTATGAC
GTGTGCCTTGTGGTGATTACCTGTGTTTAAGCTTTATGAAACTGGGATAAAAGCTCCTGGAACAGTGGAACTCGGGTTCTCTCGGTTCTAAATTTAAAAGGATACTG
GPD promoter
1,080 1,100 1,120 1,140 1,160

TTGATGCAAATTCCCAAAGCTAATAACATGCAAGACACGTACGGTCAAGAAGACATATTTGACCTCTTAACAGGTTCAGACGCGACTGCCTCATCAGTAAGACCCGT
AACTACGTTTAAGGGTTTCGATTATTGTACGTTCTGTGCATGCCAGTTCTTCTGTATAAACTGGAGAATTGTCCAAGTCTGCGCTGACGGAGTAGTCATTCTGGGCA
GPD promoter
1,180 1,200 1,220 1,240 1,260 1,280

TGAAAAGAACTTACCTGAAAAAAACGAATATATACTAGCGTTGAATGTTAGCGTCAACAACAAGAAGTTTAATGACGCGGAGGCCAAGGCAAAAAGATTCCTTGATT
ACTTTTCTTGAATGGACTTTTTTTGCTTATATATGATCGCAACTTACAATCGCAGTTGTTGTTCTTCAAATTACTGCGCCTCCGGTTCCGTTTTTCTAAGGAACTAA
GPD promoter
1,300 1,320 1,340 1,360 1,380

ACGTAAGGGAGTTAGAATCATTTTGAATAAAAAACACGCTTTTTCAGTTCGAGTTTATCATTATCAATACTGCCATTTCAAAGAATACGTAAATAATTAATAGTAGT
TGCATTCCCTCAATCTTAGTAAAACTTATTTTTTGTGCGAAAAAGTCAAGCTCAAATAGTAATAGTTATGACGGTAAAGTTTCTTATGCATTTATTAATTATCATCA
GPD promoter
1,400 1,420 1,440 1,460 1,480

GATTTTCCTAACTTTATTTAGTCAAAAAATTAGCCTTTTAATTCTGCTGTAACCCGTACATGCCCAAAATAGGGGGCGGGTTACACAGAATATATAACATCGTAGGT
CTAAAAGGATTGAAATAAATCAGTTTTTTAATCGGAAAATTAAGACGACATTGGGCATGTACGGGTTTTATCCCCCGCCCAATGTGTCTTATATATTGTAGCATCCA
GPD promoter
1,500 1,520 1,540 1,560 1,580 1,600

GTCTGGGTGAACAGTTTATTCCTGGCATCCACTAAATATAATGGAGCCCGCTTTTTAAGCTGGCATCCAGAAAAAAAAAGAATCCCAGCACCAAAATATTGTTTTCT
CAGACCCACTTGTCAAATAAGGACCGTAGGTGATTTATATTACCTCGGGCGAAAAATTCGACCGTAGGTCTTTTTTTTTCTTAGGGTCGTGGTTTTATAACAAAAGA
GPD promoter
1,620 1,640 1,660 1,680 1,700

TCACCAACCATCAGTTCATAGGTCCATTCTCTTAGCGCAACTACAGAGAACAGGGGCACAAACAGGCAAAAAACGGGCACAACCTCAATGGAGTGATGCAACCTGCC
AGTGGTTGGTAGTCAAGTATCCAGGTAAGAGAATCGCGTTGATGTCTCTTGTCCCCGTGTTTGTCCGTTTTTTGCCCGTGTTGGAGTTACCTCACTACGTTGGACGG
GPD promoter
1,720 1,740 1,760 1,780 1,800

TGGAGTAAATGATGACACAAGGCAATTGACCCACGCATGTATCTATCTCATTTTCTTACACCTTCTATTACCTTCTGCTCTCTCTGATTTGGAAAAAGCTGAAAAAA
ACCTCATTTACTACTGTGTTCCGTTAACTGGGTGCGTACATAGATAGAGTAAAAGAATGTGGAAGATAATGGAAGACGAGAGAGACTAAACCTTTTTCGACTTTTTT
GPD promoter
1,820 1,840 1,860 1,880 1,900 1,920

AAGGTTGAAACCAGTTCCCTGAAATTATTCCCCTACTTGACTAATAAGTATATAAAGACGGTAGGTATTGATTGTAATTCTGTAAATCTATTTCTTAAACTTCTTAA
TTCCAACTTTGGTCAAGGGACTTTAATAAGGGGATGAACTGATTATTCATATATTTCTGCCATCCATAACTAACATTAAGACATTTAGATAAAGAATTTGAAGAATT

GPD promoter

1,940 1,960 1,980 2,000 2,020

ATTCTACTTTTATAGTTAGTCTTTTTTTTAGTTTTAAAACACCAGAACTTAGTTTCGACGGATTCTAGAACTAGTGGATCCATGAGATTCCCATCTATCTTCACCGC
TAAGATGAAAATATCAATCAGAAAAAAAATCAAAATTTTGTGGTCTTGAATCAAAGCTGCCTAAGATCTTGATCACCTAGGTACTCTAAGGGTAGATAGAAGTGGCG

1 2 3 4 5 6 7 8 9
M R F P S I F T A

GPD promoter | Mating fac...ha leader

2,040 2,060 2,080 2,100 2,120 2,140

TGTTTTGTTCGCTGCTTCTTCTGCTTTGGCTGCTCCAGCTAACACCACCACCGAAGACGAAACCGCTCAAATCCCAGCTGAAGCTGTTATCGACTACTCTGACTTGG
ACAAAACAAGCGACGAAGAAGACGAAACCGACGAGGTCGATTGTGGTGGTGGCTTCTGCTTTGGCGAGTTTAGGGTCGACTTCGACAATAGCTGATGAGACTGAACC

10 11 12 13 14 15 16 17 18 19 20 21 22 23 24 25 26 27 28 29 30 31 32 33 34 35 36 37 38 39 40 41 42 43 44
V L F A A S S A L A A P A N T T T E D E T A Q I P A E A V I D Y S D L

Mating factor alpha leader

2,160 2,180 2,200 2,220 2,240

AAGGTGACTTCGACGCTGCTGCTTTGCCATTGTCTAACTCTACCAACAACGGTTTGTCTTCTACCAACACCACCATCGCTTCTATCGCTGCTAAGGAAGAAGGTGTT
TTCCACTGAAGCTGCGACGACGAAACGGTAACAGATTGAGATGGTTGTTGCCAAACAGAAGATGGTTGTGGTGGTAGCGAAGATAGCGACGATTCCTTCTTCCACAA

45 46 47 48 49 50 51 52 53 54 55 56 57 58 59 60 61 62 63 64 65 66 67 68 69 70 71 72 73 74 75 76 77 78 79 80
E G D F D A A A L P L S N S T N N G L S S T N T T I A S I A A K E E G V

Mating factor alpha leader

2,260 2,280 2,300 2,320 2,340

CAATTGGACAAGAGAGAAagatctggcggccgcgcatgcgctagcctcgagGTAACTACCTTGTCTGGCTTATCAGGGGAACAGGGTCCTAGCGGTGACATGACGAC
GTTAACCTGTTCTCTCTTtctagaccgccggcgcgtacgcgatcggagctcCATTGATGGAACAGACCGAATAGTCCCCTTGTCCCAGGATCGCCACTGTACTGCTG

81 82 83 84 85 86
Q L D K R E

2 4 6 8 10 12 14 16 18
V T T L S G L S G E Q G P S G D M T T

Mating...eader | SpyCatcher

2,360 2,380 2,400 2,420 2,440 2,460

AGAGGAGGACAGTGCGACCCACATAAAGTTTAGCAAAAGAGATGAAGATGGAAGGGAACTAGCTGGAGCGACCATGGAACTTCGTGACAGCTCAGGGAAGACTATAT
TCTCCTCCTGTCACGCTGGGTGTATTTCAAATCGTTTTCTCTACTTCTACCTTCCCTTGATCGACCTCGCTGGTACCTTGAAGCACTGTCGAGTCCCTTCTGATATA

20 22 24 26 28 30 32 34 36 38 40 42 44 46 48 50 52 54
E E D S A T H I K F S K R D E D G R E L A G A T M E L R D S S G K T I

SpyCatcher

2,480 2,500 2,520 2,540 2,560

```
CAACGTGGATCTCAGATGGTCACGTCAAGGACTTCTATTTGTACCCCGGAAAGTACACATTTGTGGAAACCGCCGCGCCGGATGGCTACGAGGTAGCGACCCCGATA
GTTGCACCTAGAGTCTACCAGTGCAGTTCCTGAAGATAAACATGGGGCCTTTCATGTGTAAACACCTTTGGCGGCGCGGCCTACCGATGCTCCATCGCTGGGGCTAT
56 58 60 62 64 66 68 70 72 74 76 78 80 82 84 86 88 90
S T W I S D G H V K D F Y L Y P G K Y T F V E T A A P D G Y E V A T P I
SpyCatcher
2,580 2,600 2,620 2,640 2,660

GAATTCACCGTTAACGAGGACGGCCAAGTCACCGTAGATGGAGAGGCGACAGAGGGTGATGCCCACACTGGGGGCTCCGGCGGATCTGGTGGGAGCGGATCCTACCC
CTTAAGTGGCAATTGCTCCTGCCGGTTCAGTGGCATCTACCTCTCCGCTGTCTCCCACTACGGGTGTGACCCCCGAGGCCGCCTAGACCACCCTCGCCTAGGATGGG
92 94 96 98 100 102 104 106 108 110 112 1 2 3 4 5 6 7 8 9 10 11 1 2
E F T V N E D G Q V T V D G E A T E G D A H T G G S G G S G G S G S Y P
SpyCatcher
2,680 2,700 2,720 2,740 2,760 2,780

ATACGATGTTCCAGATTACGCTCAAACATCCTCACCCTCTACAGAAGCATCAGTATCAACCTCTAGTACCTCTTCCTCTGCTTCACAATCATCTGACCCAACTACAA
TATGCTACAAGGTCTAATGCGAGTTTGTAGGAGTGGGAGATGTCTTCGTAGTCATAGTTGGAGATCATGGAGAAGGAGACGAAGTGTTAGTAGACTGGGTTGATGTT
3 4 5 6 7 8 9 2 4 6 8 10 12 14 16 18 20 22 24 26 28
Y D V P D Y A Q T S S P S T E A S V S T S S T S S S A S Q S S D P T T
HA tag 649 stalk
2,800 2,820 2,840 2,860 2,880

CATCTTCGTCCAGTTCATCGTCTTCCCCATCTTCCCAATCTGAGGAAATTTCATCATCACCGACGGTCTCAACTACACCATCAACTTCTTCATCATCTTCCTCAATG
GTAGAAGCAGGTCAAGTAGCAGAAGGGGTAGAAGGGTTAGACTCCTTTAAAGTAGTAGTGGCTGCCAGAGTTGATGTGGTAGTTGAAGAAGTAGTAGAAGGAGTTAC
30 32 34 36 38 40 42 44 46 48 50 52 54 56 58 60 62 64
T S S S S S S S S P S S Q S E E I S S S P T V S T T P S T S S S S S S M
649 stalk
2,900 2,920 2,940 2,960 2,980

ACTTCAACCACCACAACAAAGTCAATCTCAACTTCCACTACAAGTTCAGCTCCAGTTACAGATGTGACAGTTTCCTCATCGCCTAGTAAATCTACCTCTACTTCGAC
TGAAGTTGGTGGTGTTGTTTCAGTTAGAGTTGAAGGTGATGTTCAAGTCGAGGTCAATGTCTACACTGTCAAAGGAGTAGCGGATCATTTAGATGGAGATGAAGCTG
66 68 70 72 74 76 78 80 82 84 86 88 90 92 94 96 98 100
T S T T T T K S I S T S T T S S A P V T D V T V S S S P S K S T S T S T
649 stalk
3,000 3,020 3,040 3,060 3,080 3,100

AAGTACAGAAACATCTAAAACACCTACTTCAATGACAGAGTATACATCTAGTACATCGATAATTTCGACTCCAGTTAGTCACTCGCAGACAGGTTTGTCGGCTTCAT
TTCATGTCTTTGTAGATTTTGTGGATGAAGTTACTGTCTCATATGTAGATCATGTAGCTATTAAAGCTGAGGTCAATCAGTGAGCGTCTGTCCAAACAGCCGAAGTA
102 104 106 108 110 112 114 116 118 120 122 124 126 128 130 132 134
S T E T S K T P T S M T E Y T S S T S I I S T P V S H S Q T G L S A S
649 stalk
3,120 3,140 3,160 3,180 3,200
```

```
CAAGTTCATCATCTACAACATCCGGTTCTTCGTCCACTAAATCAGAAAGTTCGACAACATCTGGCTCTTCCCAGTCCGTGGAATCAACCTCCAGCCACGCCACTGTT
GTTCAAGTAGTAGATGTTGTAGGCCAAGAAGCAGGTGATTTAGTCTTTCAAGCTGTTGTAGACCGAGAAGGGTCAGGCACCTTAGTTGGAGGTCGGTGCGGTGACAA
136  138  140  142  144  146  148  150  152  154  156  158  160  162  164  166  168  170
S S S S S T T S G S S S T K S E S S T T S G S S Q S V E S T S S H A T V
649 stalk
3,220  3,240  3,260  3,280  3,300

CTTGCTAATTCCGCAGAAATGGTCACAACATCCTCTAGTTCATCCTCAACATCCGAAATGTCATTAACTAGTACTGCTACCAGTGTACCAGTCTCATCTAGTAGCAG
GAACGATTAAGGCGTCTTTACCAGTGTTGTAGGAGATCAAGTAGGAGTTGTAGGCTTTACAGTAATTGATCATGACGATGGTCACATGGTCAGAGTAGATCATCGTC
172  174  176  178  180  182  184  186  188  190  192  194  196  198  200  202  204  206
L A N S A E M V T T S S S S S T S E M S L T S T A T S V P V S S S S S
649 stalk
3,320  3,340  3,360  3,380  3,400  3,420

TACGACATATTCTACTAGCGCATCTACACAAGCCGTCACTACAACATCTTCTTCCACTGTATCTACAACTTCTTCTAGTACAACGTTAACAAGCGCATTCACACATT
ATGCTGTATAAGATGATCGCGTAGATGTGTTCGGCAGTGATGTTGTAGAAGAAGGTGACATAGATGTTGAAGAAGATCATGTTGCAATTGTTCGCGTAAGTGTGTAA
208  210  212  214  216  218  220  222  224  226  228  230  232  234  236  238  240  242
T T Y S T S A S T Q A V T T T S S S T V S T T S S S T T L T S A F T H
649 stalk
3,440  3,460  3,480  3,500  3,520

CTTCAACCACATCGTCCGACCAGCCACCCAGCGACACTACAAGTCCATCTACGACACACGAACCTCATGTAACCACTCAGACGTCATCAGAAACATCTTCTTCTAAG
GAAGTTGGTGTAGCAGGCTGGTCGGTGGGTCGCTGTGATGTTCAGGTAGATGCTGTGTGCTTGGAGTACATTGGTGAGTCTGCAGTAGTCTTTGTAGAAGAAGATTC
244  246  248  250  252  254  256  258  260  262  264  266  268  270  272  274  276  278
S S T T S S D Q P P S D T T S P S T T H E P H V T T Q T S S E T S S S K
649 stalk
3,540  3,560  3,580  3,600  3,620

TCATCTTCTACTTCTTCTTCAAGTACATCTCAAACCTCTGAGTCTGCAACACCATCCGATTCCGTATCACCTGGAAGTTCTACATCAACATCTTCTAGTAGCACTTC
AGTAGAAGATGAAGAAGAAGTTCATGTAGAGTTTGGAGACTCAGACGTTGTGGTAGGCTAAGGCATAGTGGACCTTCAAGATGTAGTTGTAGAAGATCATCGTGAAG
280  282  284  286  288  290  292  294  296  298  300  302  304  306  308  310  312  314
S S S T S S S S T S Q T S E S A T P S D S V S P G S S T S T S S S S T S
649 stalk
3,640  3,660  3,680  3,700  3,720  3,740

TACTTCCACTTCTATTTCCAGTGGAGAAACGACAACTTCTTCTTCTTCATCATCTGCCACGACCACTTCTAACAGCGCAACCTTGTCAGTCTCTACCACACAAACTT
ATGAAGGTGAAGATAAAGGTCACCTCTTTGCTGTTGAAGAAGAAGAAGTAGTAGACGGTGCTGGTGAAGATTGTCGCGTTGGAACAGTCAGAGATGGTGTGTTTGAA
316  318  320  322  324  326  328  330  332  334  336  338  340  342  344  346  348
T S T S I S S G E T T T S S S S S S A T T T S N S A T L S V S T T Q T
649 stalk
3,760  3,780  3,800  3,820  3,840
```

```
CGATTGAAGCCAGTTCATCTACTACATCTACATCTAGTTCAACAATTACAACTTCAAGTAGTAGCGCTCACATATCGTCGAAATCTCAATCTAGTATTACCTATCCC
GCTAACTTCGGTCAAGTAGATGATGTAGATGTAGATCAAGTTGTTAATGTTGAAGTTCATCATCGCGAGTGTATAGCAGCTTTAGAGTTAGATCATAATGGATAGGG
350 352 354 356 358 360 362 364 366 368 370 372 374 376 378 380 382 384
S I E A S S S T T S T S S S T I T T S S S S A H I S S K S Q S S I T Y P
649 stalk
3,860 3,880 3,900 3,920 3,940

TCTTCCTCGACATCTTCATCTACATCGTCCTCAATTTCTAGCGAATCTGAAAGTTTTGAATCGACATCAGCAGAAGATGCTCCATCAACAGCACCTTCATCAAGTGT
AGAAGGAGCTGTAGAAGTAGATGTAGCAGGAGTTAAAGATCGCTTAGACTTTCAAAACTTAGCTGTAGTCGTCTTCTACGAGGTAGTTGTCGTGGAAGTAGTTCACA
386 388 390 392 394 396 398 400 402 404 406 408 410 412 414 416 418 420
S S S T S S S T S S S I S S E S E S F E S T S A E D A P S T A P S S S V
649 stalk
3,960 3,980 4,000 4,020 4,040 4,060

CTCTTCTAAGAGTTCTACCTCTACAACATCAAGCACATCGACATCTTCAAGCACTCCATCTCCATCACCATCTTCCGTGAGTTCTTCCTCCACCAGCTCATTGACAA
GAGAAGATTCTCAAGATGGAGATGTTGTAGTTCGTGTAGCTGTAGAAGTTCGTGAGGTAGAGGTAGTGGTAGAAGGCACTCAAGAAGGAGGTGGTCGAGTAACTGTT
422 424 426 428 430 432 434 436 438 440 442 444 446 448 450 452 454 456
S S K S S T S T T S S T S T S S S T P S P S P S S V S S S S T S S L T
649 stalk
4,080 4,100 4,120 4,140 4,160

CTTCTGCTGTATCAACACCAGCTACCTCTCATTCTCAAAGTACTGTAGTAACCACCACTACTATTACTACATCAACAGGTCCAGTGATGTCTACGACAACAGCTTAT
GAAGACGACATAGTTGTGGTCGATGGAGAGTAAGAGTTTCATGACATCATTGGTGGTGATGATAATGATGTAGTTGTCCAGGTCACTACAGATGCTGTTGTCGAATA
458 460 462 464 466 468 470 472 474 476 478 480 482 484 486 488 490 492
T S A V S T P A T S H S Q S T V V T T T T I T T S T G P V M S T T T A Y
649 stalk
4,180 4,200 4,220 4,240 4,260 4,280

TCTTCTAGTTCTACTAGCAGCTCGGAATCTTCTGAGGTTCAGTCTGTCATGTCATCTACGCCTAGTTCAACATCAACAACAACCAGTTCGGAATCTACTTCATCTAG
AGAAGATCAAGATGATCGTCGAGCCTTAGAAGACTCCAAGTCAGACAGTACAGTAGATGCGGATCAAGTTGTAGTTGTTGTTGGTCAAGCCTTAGATGAAGTAGATC
494 496 498 500 502 504 506 508 510 512 514 516 518 520 522 524 526 528
S S S S T S S S E S S E V Q S V M S S T P S S T S T T T S S E S T S S S
649 stalk
4,300 4,320 4,340 4,360 4,380

CTCCACAGCTTCTACCTCACCATCAACCTCGCAAACTTTCGAAACTTCTCCTACTATAGGAGGTGTCCCCTCAACCACTTCATTTGTCTCTACGCCAACAACGAAAT
GAGGTGTCGAAGATGGAGTGGTAGTTGGAGCGTTTGAAAGCTTTGAAGAGGATGATATCCTCCACAGGGGAGTTGGTGAAGTAAACAGAGATGCGGTTGTTGCTTTA
530 532 534 536 538 540 542 544 546 548 550 552 554 556 558 560 562
S T A S T S P S T S Q T F E T S P T I G G V P S T T S F V S T P T T K
649 stalk
4,400 4,420 4,440 4,460 4,480
```

```
TGTCGCACACTACTTCCACTATGACAGCACAGTCCGATAGTAAGTCTACCCACTCCTCAAGCACATCGACAGAAGATAAATCATCCACTGCTTCTGCAGTTGACGAA
ACAGCGTGTGATGAAGGTGATACTGTCGTGTCAGGCTATCATTCAGATGGGTGAGGAGTTCGTGTAGCTGTCTTCTATTTAGTAGGTGACGAAGACGTCAACTGCTT
564 566 568 570 572 574 576 578 580 582 584 586 588 590 592 594 596 598
L S H T T S T M T A Q S D S K S T H S S S T S T E D K S S T A S A V D E
649 stalk
4,500 4,520 4,540 4,560 4,580 4,600

AGCACTACAACATCCACTTCCACGGAGTCTACTACATCAGTAACATCAGGCACCTCCCATTCCGCTAAAGAATCTTCGTCAAATTCTAAGGTGTATAGTTCACAGAC
TCGTGATGTTGTAGGTGAAGGTGCCTCAGATGATGTAGTCATTGTAGTCCGTGGAGGGTAAGGCGATTTCTTAGAAGCAGTTTAAGATTCCACATATCAAGTGTCTG
600 602 604 606 608 610 612 614 616 618 620 622 624 626 628 630 632 634
S T T T S T S T E S T T S V T S G T S H S A K E S S S N S K V Y S S Q T
649 stalk
4,620 4,640 4,660 4,680 4,700

AGCACACTCATCCATAAGTGTTGCATCATCACCTAGTACAAAGGGCGCCCAAATCCAATCTTCTATGGTTGAAATCTCTACCTACGCTGGTTCTGCTAACTCTGTTA
TCGTGTGAGTAGGTATTCACAACGTAGTAGTGGATCATGTTTCCCGCGGGTTTAGGTTAGAAGATACCAACTTTAGAGATGGATGCGACCAAGACGATTGAGACAAT
636 638 640 642 644 646 648 650 652 654 656 658 660 662 664 666 668 670
A H S S I S V A S S P S T K G A Q I Q S S M V E I S T Y A G S A N S V
649 stalk    GPI anchor
4,720 4,740 4,760 4,780 4,800

ACGCTGGTGCTGGTGCTGGTGCTTTGTTCTTGTTGTTGTCTTTGGCTATCATCTAATGATTAATTAACTCGAGATCTGATAACAACAGTGTAGATGTAACAAAATCG
TGCGACCACGACCACGACCACGAAACAAGAACAACAACAGAAACCGATAGTAGATTACTAATTAATTGAGCTCTAGACTATTGTTGTCACATCTACATTGTTTTAGC
672 674 676 678 680 682 684 686 688 1
N A G A G A G A L F L L L S L A I I *
GPI anchor    alpha factor terminator
4,820 4,840 4,860 4,880 4,900 4,920

ACTTTGTTCCCACTGTACTTTTAGCTCGTACAAAATACAATATACTTTTCATTTCTCCGTAAACAACATGTTTTCCCATGTAATATCCTTTTCTATTTTTCGTTCCG
TGAAACAAGGGTGACATGAAAATCGAGCATGTTTTATGTTATATGAAAAGTAAAGAGGCATTTGTTGTACAAAAGGGTACATTATAGGAAAAGATAAAAAGCAAGGC
alpha factor terminator
4,940 4,960 4,980 5,000 5,020

TTACCAACTTTACACATACTTTATATAGCTATTCACTTCTATACACTAAAAAACTAAGACAATTTTAATTTTGCTGCCTGCCATATTTCAATTTGTTATAAATTCCT
AATGGTTGAAATGTGTATGAAATATATCGATAAGTGAAGATATGTGATTTTTTGATTCTGTTAAAATTAAAACGACGGACGGTATAAAGTTAAACAATATTTAAGGA
alpha factor terminator
5,040 5,060 5,080 5,100 5,120

ATAATTTATCCTATTAGTAGCTAAAAAAAGATGAATGTGAATCGAATCCTAAGAGAATTGGCCGGTACCCAATTCGCCCTATAGTGAGTCGTATTACGCGCGCTCAC
TATTAAATAGGATAATCATCGATTTTTTTCTACTTACACTTAGCTTAGGATTCTCTTAACCGGCCATGGGTTAAGCGGGATATCACTCAGCATAATGCGCGCGAGTG
alpha factor terminator    T7
5,140 5,160 5,180 5,200 5,220 5,240
```

TGGCCGTCGTTTTACAACGTCGTGACTGGGAAAACCCTGGCGTTACCCAACTTAATCGCCTTGCAGCACATCCCCCTTTCGCCAGCTGGCGTAATAGCGAAGAGGCC
ACCGGCAGCAAAATGTTGCAGCACTGACCCTTTTGGGACCGCAATGGGTTGAATTAGCGGAACGTCGTGTAGGGGGAAAGCGGTCGACCGCATTATCGCTTCTCCGG
M13-fwd LacZ alpha
5,260 5,280 5,300 5,320 5,340

CGCACCGATCGCCCTTCCCAACAGTTGCGCAGCCTGAATGGCGAATGGACGCGCCCTGTAGCGGCGCATTAAGCGCGGCGGGTGTGGTGGTTACGCGCAGCGTGACC
GCGTGGCTAGCGGGAAGGGTTGTCAACGCGTCGGACTTACCGCTTACCTGCGCGGGACATCGCCGCGTAATTCGCGCCGCCCACACCACCAATGCGCGTCGCACTGG
LacZ alpha
5,360 5,380 5,400 5,420 5,440

ACTACACTTGCCAGCGCCCTAGCGCCCGCTCCTTTCGCTTTCTTCCCTTCCTTTCTCGCCACGTTCGCCGGCTTTCCCCGTCAAGCTCTAAATCGGGGGCTCCCTTT
TGATGTGAACGGTCGCGGGATCGCGGGCGAGGAAAGCGAAAGAAGGGAAGGAAAGAGCGGTGCAAGCGGCCGAAAGGGGCAGTTCGAGATTTAGCCCCCGAGGGAAA
5,460 5,480 5,500 5,520 5,540 5,560

AGGGTTCCGATTTAGTGCTTTACGGCACCTCGACCCCAAAAAACTTGATTAGGGTGATGGTTCACGTAGTGGGCCATCGCCCTGATAGACGGTTTTTCGCCCTTTGA
TCCCAAGGCTAAATCACGAAATGCCGTGGAGCTGGGGTTTTTTGAACTAATCCCACTACCAAGTGCATCACCCGGTAGCGGGACTATCTGCCAAAAAGCGGGAAACT
5,580 5,600 5,620 5,640 5,660

CGTTGGAGTCCACGTTCTTTAATAGTGGACTCTTGTTCCAAACTGGAACAACACTCAACCCTATCTCGGTCTATTCTTTTGATTTATAAGGGATTTTGCCGATTTCG
GCAACCTCAGGTGCAAGAAATTATCACCTGAGAACAAGGTTTGACCTTGTTGTGAGTTGGGATAGAGCCAGATAAGAAAACTAAATATTCCCTAAAACGGCTAAAGC
5,680 5,700 5,720 5,740 5,760

GCCTATTGGTTAAAAAATGAGCTGATTTAACAAAAATTTAACGCGAATTTTAACAAAATATTAACGCTTACAATTTCCTGATGCGGTATTTTCTCCTTACGCATCTG
CGGATAACCAATTTTTTACTCGACTAAATTGTTTTTAAATTGCGCTTAAAATTGTTTTATAATTGCGAATGTTAAAGGACTACGCCATAAAAGAGGAATGCGTAGAC
5,780 5,800 5,820 5,840 5,860 5,880

TGCGGTATTTCACACCGCATAGGGTAATAACTGATATAATTAAATTGAAGCTCTAATTTGTGAGTTTAGTATACATGCATTTACTTATAATACAGTTTTTTAAGCAA
ACGCCATAAAGTGTGGCGTATCCCATTATTGACTATATTAATTTAACTTCGAGATTAAACACTCAAATCATATGTACGTAAATGAATATTATGTCAAAAAATTCGTT
5,900 5,920 5,940 5,960 5,980

GGATTTTCTTAACTTCTTCGGCGACAGCATCACCGACTTCGGTGGTACTGTTGGAACCACCTAAATCACCAGTTCTGATACCTGCATCCAAAACCTTTTTAACTGCA
CCTAAAAGAATTGAAGAAGCCGCTGTCGTAGTGGCTGAAGCCACCATGACAACCTTGGTGGATTTAGTGGTCAAGACTATGGACGTAGGTTTTGGAAAAATTGACGT
LEU2
6,000 6,020 6,040 6,060 6,080

TCTTCAATGGCCTTACCTTCTTCAGGCAAGTTCAATGACAATTTCAACATCATTGCAGCAGACAAGATAGTGGCGATAGGGTTGACCTTATTCTTTGGCAAATCTGG
AGAAGTTACCGGAATGGAAGAAGTCCGTTCAAGTTACTGTTAAAGTTGTAGTAACGTCGTCTGTTCTATCACCGCTATCCCAACTGGAATAAGAAACCGTTTAGACC
LEU2
6,100 6,120 6,140 6,160 6,180 6,200

AGCAGAACCGTGGCATGGTTCGTACAAACCAAATGCGGTGTTCTTGTCTGGCAAAGAGGCCAAGGACGCAGATGGCAACAAACCCAAGGAACCTGGGATAACGGAGG
TCGTCTTGGCACCGTACCAAGCATGTTTGGTTTACGCCACAAGAACAGACCGTTTCTCCGGTTCCTGCGTCTACCGTTGTTTGGGTTCCTTGGACCCTATTGCCTCC
LEU2
6,220 6,240 6,260 6,280 6,300

CTTCATCGGAGATGATATCACCAAACATGTTGCTGGTGATTATAATACCATTTAGGTGGGTTGGGTTCTTAACTAGGATCATGGCGGCAGAATCAATCAATTGATGT
GAAGTAGCCTCTACTATAGTGGTTTGTACAACGACCACTAATATTATGGTAAATCCACCCAACCCAAGAATTGATCCTAGTACCGCCGTCTTAGTTAGTTAACTACA
LEU2
6,320 6,340 6,360 6,380 6,400 6,420

TGAACCTTCAATGTAGGGAATTCGTTCTTGATGGTTTCCTCCACAGTTTTTCTCCATAATCTTGAAGAGGCCAAAACATTAGCTTTATCCAAGGACCAAATAGGCAA
ACTTGGAAGTTACATCCCTTAAGCAAGAACTACCAAAGGAGGTGTCAAAAAGAGGTATTAGAACTTCTCCGGTTTTGTAATCGAAATAGGTTCCTGGTTTATCCGTT
LEU2
6,440 6,460 6,480 6,500 6,520

TGGTGGCTCATGTTGTAGGGCCATGAAAGCGGCCATTCTTGTGATTCTTTGCACTTCTGGAACGGTGTATTGTTCACTATCCCAAGCGACACCATCACCATCGTCTT
ACCACCGAGTACAACATCCCGGTACTTTCGCCGGTAAGAACACTAAGAAACGTGAAGACCTTGCCACATAACAAGTGATAGGGTTCGCTGTGGTAGTGGTAGCAGAA
LEU2
6,540 6,560 6,580 6,600 6,620

CCTTTCTCTTACCAAAGTAAATACCTCCCACTAATTCTCTGACAACAACGAAGTCAGTACCTTTAGCAAATTGTGGCTTGATTGGAGATAAGTCTAAAAGAGAGTCG
GGAAAGAGAATGGTTTCATTTATGGAGGGTGATTAAGAGACTGTTGTTGCTTCAGTCATGGAAATCGTTTAACACCGAACTAACCTCTATTCAGATTTTCTCTCAGC
LEU2
6,640 6,660 6,680 6,700 6,720 6,740

GATGCAAAGTTACATGGTCTTAAGTTGGCGTACAATTGAAGTTCTTTACGGATTTTTAGTAAACCTTGTTCAGGTCTAACACTACCGGTACCCCATTTAGGACCACC
CTACGTTTCAATGTACCAGAATTCAACCGCATGTTAACTTCAAGAAATGCCTAAAAATCATTTGGAACAAGTCCAGATTGTGATGGCCATGGGGTAAATCCTGGTGG
LEU2
6,760 6,780 6,800 6,820 6,840

CACAGCACCTAACAAAACGGCATCAGCCTTCTTGGAGGCTTCCAGCGCCTCATCTGGAAGTGGAACACCTGTAGCATCGATAGCAGCACCACCAATTAAATGATTTT
GTGTCGTGGATTGTTTTGCCGTAGTCGGAAGAACCTCCGAAGGTCGCGGAGTAGACCTTCACCTTGTGGACATCGTAGCTATCGTCGTGGTGGTTAATTTACTAAAA
LEU2
6,860 6,880 6,900 6,920 6,940

CGAAATCGAACTTGACATTGGAACGAACATCAGAAATAGCTTTAAGAACCTTAATGGCTTCGGCTGTGATTTCTTGACCAACGTGGTCACCTGGCAAAACGACGATC
GCTTTAGCTTGAACTGTAACCTTGCTTGTAGTCTTTATCGAAATTCTTGGAATTACCGAAGCCGACACTAAAGAACTGGTTGCACCAGTGGACCGTTTTGCTGCTAG
LEU2
6,960 6,980 7,000 7,020 7,040 7,060

```
TTCTTAGGGGCAGACATTAGAATGGTATATCCTTGAAATATATATATATATATNTNGCTGAAATGTAAAAGGTAAGAAAAGTTAGAAAGTAAGACGATTGCTAACCA
AAGAATCCCCGTCTGTAATCTTACCATATAGGAACTTTATATATATATATATANANCGACTTTACATTTTCCATTCTTTTCAATCTTTCATTCTGCTAACGATTGGT
LEU2
     7,080     7,100     7,120     7,140     7,160

CCTATTGGAAAAAACAATAGGTCCTTAAATAATATTGTCAACTTCAAGTATTGTGATGCAAGCATTTAGTCATGAACGCTTCTCTATTCTATATGAAAAGCCGGTTC
GGATAACCTTTTTTGTTATCCAGGAATTTATTATAACAGTTGAAGTTCATAACACTACGTTCGTAAATCAGTACTTGCGAAGAGATAAGATATACTTTTCGGCCAAG
   7,180     7,200     7,220     7,240     7,260

CGGCGCTCTCACCTTTCCTTTTTCTCCCAATTTTTCAGTTGAAAAAGGTATATGCGTCAGGCGACCTCTGAAATTAACAAAAAATTTCCAGTCATCGAATTTGATTC
GCCGCGAGAGTGGAAAGGAAAAAGAGGGTTAAAAAGTCAACTTTTTCCATATACGCAGTCCGCTGGAGACTTTAATTGTTTTTTAAAGGTCAGTAGCTTAAACTAAG
7,280     7,300     7,320     7,340     7,360     7,380

TGTGCGATAGCGCCCCTGTGTGTTCTCGTTATGTTGAGGAAAAAAATAATGGTTGCTAAGAGATTCGAACTCTTGCATCTTACGATACCTGAGTATTCCCACAGTTT
ACACGCTATCGCGGGGACACACAAGAGCAATACAACTCCTTTTTTTATTACCAACGATTCTCTAAGCTTGAGAACGTAGAATGCTATGGACTCATAAGGGTGTCAAA
     7,400     7,420     7,440     7,460     7,480

GAAAAGCTGTGGTATGGTGCACTCTCAGTACAATCTGCTCTGATGCCGCATAGTTAAGCCAGCCCCGACACCCGCCAACACCCGCTGACGCGCCCTGACGGGCTTGT
CTTTTCGACACCATACCACGTGAGAGTCATGTTAGACGAGACTACGGCGTATCAATTCGGTCGGGGCTGTGGGCGGTTGTGGGCGACTGCGCGGGACTGCCCGAACA
   7,500     7,520     7,540     7,560     7,580

CTGCTCCCGGCATCCGCTTACAGACAAGCTGTGACCGTCTCCGGGAGCTGCATGTGTCAGAGGTTTTCACCGTCATCACCGAAACGCGCGAGACGAAAGGGCCTCGT
GACGAGGGCCGTAGGCGAATGTCTGTTCGACACTGGCAGAGGCCCTCGACGTACACAGTCTCCAAAAGTGGCAGTAGTGGCTTTGCGCGCTCTGCTTTCCCGGAGCA
7,600     7,620     7,640     7,660     7,680     7,700

GATACGCCTATTTTTATAGGTTAATGTCATGATAATAATGGTTTCTTAGTATGATCCAATATCAAAGGAAATGATAGCATTGAAGGATGAGACTAATCCAATTGAGG
CTATGCGGATAAAAATATCCAATTACAGTACTATTATTACCAAAGAATCATACTAGGTTATAGTTTCCTTTACTATCGTAACTTCCTACTCTGATTAGGTTAACTCC
                                                     2 micron origin
     7,720     7,740     7,760     7,780     7,800

AGTGGCAGCATATAGAACAGCTAAAGGGTAGTGCTGAAGGAAGCATACGATACCCCGCATGGAATGGGATAATATCACAGGAGGTACTAGACTACCTTTCATCCTAC
TCACCGTCGTATATCTTGTCGATTTCCCATCACGACTTCCTTCGTATGCTATGGGGCGTACCTTACCCTATTATAGTGTCCTCCATGATCTGATGGAAAGTAGGATG
                                   2 micron origin
   7,820     7,840     7,860     7,880     7,900

ATAAATAGACGCATATAAGTACGCATTTAAGCATAAACACGCACTATGCCGTTCTTCTCATGTATATATATATACAGGCAACACGCAGATATAGGTGCGACGTGAAC
TATTTATCTGCGTATATTCATGCGTAAATTCGTATTTGTGCGTGATACGGCAAGAAGAGTACATATATATATATGTCCGTTGTGCGTCTATATCCACGCTGCACTTG
                                   2 micron origin
7,920     7,940     7,960     7,980     8,000     8,020
```

AGTGAGCTGTATGTGCGCAGCTCGCGTTGCATTTTCGGAAGCGCTCGTTTTCGGAAACGCTTTGAAGTTCCTATTCCGAAGTTCCTATTCTCTAGAAAGTATAGGAA
TCACTCGACATACACGCGTCGAGCGCAACGTAAAAGCCTTCGCGAGCAAAAGCCTTTGCGAAACTTCAAGGATAAGGCTTCAAGGATAAGAGATCTTTCATATCCTT

FRT

2 micron origin

8,040 8,060 8,080 8,100 8,120

CTTCAGAGCGCTTTTGAAAACCAAAAGCGCTCTGAAGACGCACTTTCAAAAAACCAAAAACGCACCGGACTGTAACGAGCTACTAAAATATTGCGAATACCGCTTCC
GAAGTCTCGCGAAAACTTTTGGTTTTCGCGAGACTTCTGCGTGAAAGTTTTTTGGTTTTTGCGTGGCCTGACATTGCTCGATGATTTTATAACGCTTATGGCGAAGG

2 micron origin

8,140 8,160 8,180 8,200 8,220

ACAAACATTGCTCAAAAGTATCTCTTTGCTATATATCTCTGTGCTATATCCCTATATAACCTACCCATCCACCTTTCGCTCCTTGAACTTGCATCTAAACTCGACCT
TGTTTGTAACGAGTTTTCATAGAGAAACGATATATAGAGACACGATATAGGGATATATTGGATGGGTAGGTGGAAAGCGAGGAACTTGAACGTAGATTTGAGCTGGA

2 micron origin

8,240 8,260 8,280 8,300 8,320 8,340

CTACATTTTTTATGTTTATCTCTAGTATTACTCTTTAGACAAAAAAATTGTAGTAAGAACTATTCATAGAGTGAATCGAAAACAATACGAAAATGTAAACATTTCCT
GATGTAAAAAATACAAATAGAGATCATAATGAGAAATCTGTTTTTTTAACATCATTCTTGATAAGTATCTCACTTAGCTTTTGTTATGCTTTTACATTTGTAAAGGA

2 micron origin

8,360 8,380 8,400 8,420 8,440

ATACGTAGTATATAGAGACAAAATAGAAGAAACCGTTCATAATTTTCTGACCAATGAAGAATCATCAACGCTATCACTTTCTGTTCACAAAGTATGCGCAATCCACA
TATGCATCATATATCTCTGTTTTATCTTCTTTGGCAAGTATTAAAAGACTGGTTACTTCTTAGTAGTTGCGATAGTGAAAGACAAGTGTTTCATACGCGTTAGGTGT

2 micron origin

8,460 8,480 8,500 8,520 8,540 8,560

TCGGTATAGAATATAATCGGGGATGCCTTTATCTTGAAAAAATGCACCCGCAGCTTCGCTAGTAATCAGTAAACGCGGGAAGTGGAGTCAGGCTTTTTTTATGGAAG
AGCCATATCTTATATTAGCCCCTACGGAAATAGAACTTTTTTACGTGGGCGTCGAAGCGATCATTAGTCATTTGCGCCCTTCACCTCAGTCCGAAAAAAATACCTTC

2 micron origin

8,580 8,600 8,620 8,640 8,660

AGAAAATAGACACCAAAGTAGCCTTCTTCTAACCTTAACGGACCTACAGTGCAAAAAGTTATCAAGAGACTGCATTATAGAGCGCACAAAGGAGAAAAAAAGTAATC
TCTTTTATCTGTGGTTTCATCGGAAGAAGATTGGAATTGCCTGGATGTCACGTTTTTCAATAGTTCTCTGACGTAATATCTCGCGTGTTTCCTCTTTTTTTCATTAG

2 micron origin

8,680 8,700 8,720 8,740 8,760

TAAGATGCTTTGTTAGAAAAATAGCGCTCTCGGGATGCATTTTTGTAGAACAAAAAAGAAGTATAGATTCTTTGTTGGTAAAATAGCGCTCTCGCGTTGCATTTCTG
ATTCTACGAAACAATCTTTTTATCGCGAGAGCCCTACGTAAAAACATCTTGTTTTTTCTTCATATCTAAGAAACAACCATTTTATCGCGAGAGCGCAACGTAAAGAC

2 micron origin

8,780 8,800 8,820 8,840 8,860 8,880

TTCTGTAAAAATGCAGCTCAGATTCTTTGTTTGAAAAATTAGCGCTCTCGCGTTGCATTTTTGTTTTACAAAAATGAAGCACAGATTCTTCGTTGGTAAAATAGCGC
AAGACATTTTTACGTCGAGTCTAAGAAACAAACTTTTTAATCGCGAGAGCGCAACGTAAAAACAAAATGTTTTTACTTCGTGTCTAAGAAGCAACCATTTTATCGCG

2 micron origin

8,900 8,920 8,940 8,960 8,980

TTTCGCGTTGCATTTCTGTTCTGTAAAAATGCAGCTCAGATTCTTTGTTTGAAAAATTAGCGCTCTCGCGTTGCATTTTTGTTCTACAAAATGAAGCACAGATGCTT
AAAGCGCAACGTAAAGACAAGACATTTTTACGTCGAGTCTAAGAAACAAACTTTTTAATCGCGAGAGCGCAACGTAAAAACAAGATGTTTTACTTCGTGTCTACGAA

2 micron origin

9,000 9,020 9,040 9,060 9,080

CGTTCAGGTGGCACTTTTCGGGGAAATGTGCGCGGAACCCCTATTTGTTTATTTTTCTAAATACATTCAAATATGTATCCGCTCATGAGACAATAACCCTGATAAAT
GCAAGTCCACCGTGAAAAGCCCCTTTACACGCGCCTTGGGGATAAACAAATAAAAAGATTTATGTAAGTTTATACATAGGCGAGTACTCTGTTATTGGGACTATTTA

9,100 9,120 9,140 9,160 9,180 9,200

GCTTCAATAATATTGAAAAAGGAAGAGTATGAGTATTCAACATTTCCGTGTCGCCCTTATTCCCTTTTTTGCGGCATTTTGCCTTCCTGTTTTTGCTCACCCAGAAA
CGAAGTTATTATAACTTTTTCCTTCTCATACTCATAAGTTGTAAAGGCACAGCGGGAATAAGGGAAAAAACGCCGTAAAACGGAAGGACAAAAACGAGTGGGTCTTT

9,220 9,240 9,260 9,280 9,300

CGCTGGTGAAAGTAAAAGATGCTGAAGATCAGTTGGGTGCACGAGTGGGTTACATCGAACTGGATCTCAACAGCGGTAAGATCCTTGAGAGTTTTCGCCCCGAAGAA
GCGACCACTTTCATTTTCTACGACTTCTAGTCAACCCACGTGCTCACCCAATGTAGCTTGACCTAGAGTTGTCGCCATTCTAGGAACTCTCAAAAGCGGGGCTTCTT

9,320 9,340 9,360 9,380 9,400

CGTTTTCCAATGATGAGCACTTTTAAAGTTCTGCTATGTGGCGCGGTATTATCCCGTATTGACGCCGGGCAAGAGCAACTCGGTCGCCGCATACACTATTCTCAGAA
GCAAAAGGTTACTACTCGTGAAAATTTCAAGACGATACACCGCGCCATAATAGGGCATAACTGCGGCCCGTTCTCGTTGAGCCAGCGGCGTATGTGATAAGAGTCTT

AmpR

9,420 9,440 9,460 9,480 9,500 9,520

TGACTTGGTTGAGTACTCACCAGTCACAGAAAAGCATCTTACGGATGGCATGACAGTAAGAGAATTATGCAGTGCTGCCATAACCATGAGTGATAACACTGCGGCCA
ACTGAACCAACTCATGAGTGGTCAGTGTCTTTTCGTAGAATGCCTACCGTACTGTCATTCTCTTAATACGTCACGACGGTATTGGTACTCACTATTGTGACGCCGGT

AmpR

9,540 9,560 9,580 9,600 9,620

ACTTACTTCTGACAACGATCGGAGGACCGAAGGAGCTAACCGCTTTTTTGCACAACATGGGGGATCATGTAACTCGCCTTGATCGTTGGGAACCGGAGCTGAATGAA
TGAATGAAGACTGTTGCTAGCCTCCTGGCTTCCTCGATTGGCGAAAAAACGTGTTGTACCCCCTAGTACATTGAGCGGAACTAGCAACCCTTGGCCTCGACTTACTT

AmpR

9,640 9,660 9,680 9,700 9,720

GCCATACCAAACGACGAGCGTGACACCACGATGCCTGTAGCAATGGCAACAACGTTGCGCAAACTATTAACTGGCGAACTACTTACTCTAGCTTCCCGGCAACAATT
CGGTATGGTTTGCTGCTCGCACTGTGGTGCTACGGACATCGTTACCGTTGTTGCAACGCGTTTGATAATTGACCGCTTGATGAATGAGATCGAAGGGCCGTTGTTAA

AmpR

9,740 9,760 9,780 9,800 9,820 9,840

AATAGACTGGATGGAGGCGGATAAAGTTGCAGGACCACTTCTGCGCTCGGCCCTTCCGGCTGGCTGGTTTATTGCTGATAAATCTGGAGCCGGTGAGCGTGGGTCTC
TTATCTGACCTACCTCCGCCTATTTCAACGTCCTGGTGAAGACGCGAGCCGGGAAGGCCGACCGACCAAATAACGACTATTTAGACCTCGGCCACTCGCACCCAGAG

AmpR

9,860 9,880 9,900 9,920 9,940

GCGGTATCATTGCAGCACTGGGGCCAGATGGTAAGCCCTCCCGTATCGTAGTTATCTACACGACGGGGAGTCAGGCAACTATGGATGAACGAAATAGACAGATCGCT
CGCCATAGTAACGTCGTGACCCCGGTCTACCATTCGGGAGGGCATAGCATCAATAGATGTGCTGCCCCTCAGTCCGTTGATACCTACTTGCTTTATCTGTCTAGCGA

AmpR

9,960 9,980 10,000 10,020 10,040

GAGATAGGTGCCTCACTGATTAAGCATTGGTAACTGTCAGACCAAGTTTACTCATATATACTTTAGATTGATTTAAAACTTCATTTTTAATTTAAAAGGATCTAGGT
CTCTATCCACGGAGTGACTAATTCGTAACCATTGACAGTCTGGTTCAAATGAGTATATATGAAATCTAACTAAATTTTGAAGTAAAAATTAAATTTTCCTAGATCCA

AmpR

10,060 10,080 10,100 10,120 10,140 10,160

GAAGATCCTTTTTGATAATCTCATGACCAAAATCCCTTAACGTGAGTTTTCGTTCCACTGAGCGTCAGACCCCGTAGAAAAGATCAAAGGATCTTCTTGAGATCCTT
CTTCTAGGAAAAACTATTAGAGTACTGGTTTTAGGGAATTGCACTCAAAAGCAAGGTGACTCGCAGTCTGGGGCATCTTTTCTAGTTTCCTAGAAGAACTCTAGGAA

10,180 10,200 10,220 10,240 10,260

TTTTTCTGCGCGTAATCTGCTGCTTGCAAACAAAAAAACCACCGCTACCAGCGGTGGTTTGTTTGCCGGATCAAGAGCTACCAACTCTTTTTCCGAAGGTAACTGGC
AAAAAGACGCGCATTAGACGACGAACGTTTGTTTTTTTGGTGGCGATGGTCGCCACCAAACAAACGGCCTAGTTCTCGATGGTTGAGAAAAAGGCTTCCATTGACCG

10,280 10,300 10,320 10,340 10,360

TTCAGCAGAGCGCAGATACCAAATACTGTTCTTCTAGTGTAGCCGTAGTTAGGCCACCACTTCAAGAACTCTGTAGCACCGCCTACATACCTCGCTCTGCTAATCCT
AAGTCGTCTCGCGTCTATGGTTTATGACAAGAAGATCACATCGGCATCAATCCGGTGGTGAAGTTCTTGAGACATCGTGGCGGATGTATGGAGCGAGACGATTAGGA

10,400 10,420 10,440 10,460 10,480

GTTACCAGTGGCTGCTGCCAGTGGCGATAAGTCGTGTCTTACCGGGTTGGACTCAAGACGATAGTTACCGGATAAGGCGCAGCGGTCGGGCTGAACGGGGGGTTCGT
CAATGGTCACCGACGACGGTCACCGCTATTCAGCACAGAATGGCCCAACCTGAGTTCTGCTATCAATGGCCTATTCCGCGTCGCCAGCCCGACTTGCCCCCCAAGCA

10,500 10,520 10,540 10,560 10,580

GCACACAGCCCAGCTTGGAGCGAACGACCT
CGTGTGTCGGGTCGAACCTCGCTTGCTGGA

10,600 10,610 10,620

ST/SC ligation / pAnchor_N.C. of immunostaining...

```
ACACCGAACTGAGATACCTACAGCGTGAGCTATGAGAAAGCGCCACGCTTCCCGAAGGGAGAAAGGCGGACAGGTATCCGGTAAGCGGCAGGGTCGGAACAGGAGAG
TGTGGCTTGACTCTATGGATGTCGCACTCGATACTCTTTCGCGGTGCGAAGGGCTTCCCTCTTTCCGCCTGTCCATAGGCCATTCGCCGTCCCAGCCTTGTCCTCTC
                    20                  40                  60                  80                  100

CGCACGAGGGAGCTTCCAGGGGGAAACGCCTGGTATCTTTATAGTCCTGTCGGGTTTCGCCACCTCTGACTTGAGCGTCGATTTTTGTGATGCTCGTCAGGGGGGCG
GCGTGCTCCCTCGAAGGTCCCCCTTTGCGGACCATAGAAATATCAGGACAGCCCAAAGCGGTGGAGACTGAACTCGCAGCTAAAAACACTACGAGCAGTCCCCCCGC
             120                 140                 160                 180                 200

GAGCCTATGGAAAAACGCCAGCAACGCGGCCTTTTTACGGTTCCTGGCCTTTTGCTGGCCTTTTGCTCACATGTTCTTTCCTGCGTTATCCCCTGATTCTGTGGATA
CTCGGATACCTTTTTGCGGTCGTTGCGCCGGAAAAATGCCAAGGACCGGAAAACGACCGGAAAACGAGTGTACAAGAAAGGACGCAATAGGGGACTAAGACACCTAT
      220                 240                 260                 280                 300                 320

ACCGTATTACCGCCTTTGAGTGAGCTGATACCGCTCGCCGCAGCCGAACGACCGAGCGCAGCGAGTCAGTGAGCGAGGAAGCGGAAGAGCGCCCAATACGCAAACCG
TGGCATAATGGCGGAAACTCACTCGACTATGGCGAGCGGCGTCGGCTTGCTGGCTCGCGTCGCTCAGTCACTCGCTCCTTCGCCTTCTCGCGGGTTATGCGTTTGGC
                   340                 360                 380                 400                 420

CCTCTCCCCGCGCGTTGGCCGATTCATTAATGCAGCTGGCACGACAGGTTTCCCGACTGGAAAGCGGGCAGTGAGCGCAACGCAATTAATGTGAGTTAGCTCACTCA
GGAGAGGGGCGCGCAACCGGCTAAGTAATTACGTCGACCGTGCTGTCCAAAGGGCTGACCTTTCGCCCGTCACTCGCGTTGCGTTAATTACACTCAATCGAGTGAGT
          440                 460                 480                 500                 520

TTAGGCACCCCAGGCTTTACACTTTATGCTTCCGGCTCGTATGTTGTGTGGAATTGTGAGCGGATAACAATTTCACACAGGAAACAGCTATGACCATGATTACGCCA
AATCCGTGGGGTCCGAAATGTGAAATACGAAGGCCGAGCATACAACACACCTTAACACTCGCCTATTGTTAAAGTGTGTCCTTTGTCGATACTGGTACTAATGCGGT
               lac promoter                 LacO                   M13-rev
    540                 560                 580                 600                 620                 640

AGCGCGCAATTAACCCTCACTAAAGGGAACAAAAGCTGGAGCTAGTATACTCTTTCTTCAACAATTAAATACTCTCGGTAGCCAAGTTGGTTTAAGGCGCAAGACTG
TCGCGCGTTAATTGGGAGTGATTTCCCTTGTTTTCGACCTCGATCATATGAGAAAGAAGTTGTTAATTTATGAGAGCCATCGGTTCAACCAAATTCCGCGTTCTGAC
         T3
     T3 promoter
                  660                 680                 700                 720                 740

TAATTTATCACTACGAAATCTTGAGATCGGGCGTTCGACTCGCCCCCGGGAGAGATGGCCGGCATGGTCCCAGCCTCCTCGCTGGCGCCGGCTGGGCAACACCTTCG
ATTAAATAGTGATGCTTTAGAACTCTAGCCCGCAAGCTGAGCGGGGGCCCTCTCTACCGGCCGTACCAGGGTCGGAGGAGCGACCGCGGCCGACCCGTTGTGGAAGC
           760                 780                 800                 820                 840

GGTGGCGAATGGGACTTTTTATGTGCGTATTGCTTTCAGTTTTAGAGCTAGAAATAGCAAGTTAAAATAAGGCTAGTCCGTTATCAACTTGAAAAAGTGGCACCGAG
CCACCGCTTACCCTGAAAAATACACGCATAACGAAAGTCAAAATCTCGATCTTTATCGTTCAATTTTATTCCGATCAGGCAATAGTTGAACTTTTTCACCGTGGCTC
   860                 880                 900                 920                 940                 960

TCGGTGCTTTTTTTATTTTTTGTCACTATTGTTATGTAAAATGCCACCTCTGACAGTATGGAACGCAAACTTCTGTCTAGTGGATAGTCGACAAGCTTACCAGTTCT
AGCCACGAAAAAAATAAAAAACAGTGATAACAATACATTTTACGGTGGAGACTGTCATACCTTGCGTTTGAAGACAGATCACCTATCAGCTGTTCGAATGGTCAAGA
                                                                                                  GPD ...ter
           980                1,000               1,020               1,040               1,060
```

CACACGGAACACCACTAATGGACACAAATTCGAAATACTTTGACCCTATTTTCGAGGACCTTGTCACCTTGAGCCCAAGAGAGCCAAGATTTAAATTTTCCTATGAC
GTGTGCCTTGTGGTGATTACCTGTGTTTAAGCTTTATGAAACTGGGATAAAAGCTCCTGGAACAGTGGAACTCGGGTTCTCTCGGTTCTAAATTTAAAAGGATACTG
GPD promoter
1,080 1,100 1,120 1,140 1,160

TTGATGCAAATTCCCAAAGCTAATAACATGCAAGACACGTACGGTCAAGAAGACATATTTGACCTCTTAACAGGTTCAGACGCGACTGCCTCATCAGTAAGACCCGT
AACTACGTTTAAGGGTTTCGATTATTGTACGTTCTGTGCATGCCAGTTCTTCTGTATAAACTGGAGAATTGTCCAAGTCTGCGCTGACGGAGTAGTCATTCTGGGCA
GPD promoter
1,180 1,200 1,220 1,240 1,260 1,280

TGAAAAGAACTTACCTGAAAAAAACGAATATATACTAGCGTTGAATGTTAGCGTCAACAACAAGAAGTTTAATGACGCGGAGGCCAAGGCAAAAAGATTCCTTGATT
ACTTTTCTTGAATGGACTTTTTTTGCTTATATATGATCGCAACTTACAATCGCAGTTGTTGTTCTTCAAATTACTGCGCCTCCGGTTCCGTTTTTCTAAGGAACTAA
GPD promoter
1,300 1,320 1,340 1,360 1,380

ACGTAAGGGAGTTAGAATCATTTTGAATAAAAAACACGCTTTTTCAGTTCGAGTTTATCATTATCAATACTGCCATTTCAAAGAATACGTAAATAATTAATAGTAGT
TGCATTCCCTCAATCTTAGTAAAACTTATTTTTTGTGCGAAAAAGTCAAGCTCAAATAGTAATAGTTATGACGGTAAAGTTTCTTATGCATTTATTAATTATCATCA
GPD promoter
1,400 1,420 1,440 1,460 1,480

GATTTTCCTAACTTTATTTAGTCAAAAAATTAGCCTTTTAATTCTGCTGTAACCCGTACATGCCCAAAATAGGGGGCGGGTTACACAGAATATATAACATCGTAGGT
CTAAAAGGATTGAAATAAATCAGTTTTTTAATCGGAAAATTAAGACGACATTGGGCATGTACGGGTTTTATCCCCCGCCCAATGTGTCTTATATATTGTAGCATCCA
GPD promoter
1,500 1,520 1,540 1,560 1,580 1,600

GTCTGGGTGAACAGTTTATTCCTGGCATCCACTAAATATAATGGAGCCCGCTTTTTAAGCTGGCATCCAGAAAAAAAAAGAATCCCAGCACCAAAATATTGTTTTCT
CAGACCCACTTGTCAAATAAGGACCGTAGGTGATTTATATTACCTCGGGCGAAAAATTCGACCGTAGGTCTTTTTTTTTCTTAGGGTCGTGGTTTTATAACAAAAGA
GPD promoter
1,620 1,640 1,660 1,680 1,700

TCACCAACCATCAGTTCATAGGTCCATTCTCTTAGCGCAACTACAGAGAACAGGGGCACAAACAGGCAAAAAACGGGCACAACCTCAATGGAGTGATGCAACCTGCC
AGTGGTTGGTAGTCAAGTATCCAGGTAAGAGAATCGCGTTGATGTCTCTTGTCCCCGTGTTTGTCCGTTTTTTGCCCGTGTTGGAGTTACCTCACTACGTTGGACGG
GPD promoter
1,720 1,740 1,760 1,780 1,800

TGGAGTAAATGATGACACAAGGCAATTGACCCACGCATGTATCTATCTCATTTTCTTACACCTTCTATTACCTTCTGCTCTCTCTGATTTGGAAAAAGCTGAAAAAA
ACCTCATTTACTACTGTGTTCCGTTAACTGGGTGCGTACATAGATAGAGTAAAAGAATGTGGAAGATAATGGAAGACGAGAGAGACTAAACCTTTTTCGACTTTTTT
GPD promoter
1,820 1,840 1,860 1,880 1,900 1,920

AAGGTTGAAACCAGTTCCCTGAAATTATTCCCCTACTTGACTAATAAGTATATAAAGACGGTAGGTATTGATTGTAATTCTGTAAATCTATTTCTTAAACTTCTTAA
TTCCAACTTTGGTCAAGGGACTTTAATAAGGGGATGAACTGATTATTCATATATTTCTGCCATCCATAACTAACATTAAGACATTTAGATAAAGAATTTGAAGAATT

GPD promoter

1,940 1,960 1,980 2,000 2,020

ATTCTACTTTTATAGTTAGTCTTTTTTTTAGTTTTAAAACACCAGAACTTAGTTTCGACGGATTCTAGAACTAGTGGATCCATGAGATTCCCATCTATCTTCACCGC
TAAGATGAAAATATCAATCAGAAAAAAAATCAAAATTTTGTGGTCTTGAATCAAAGCTGCCTAAGATCTTGATCACCTAGGTACTCTAAGGGTAGATAGAAGTGGCG

1 2 3 4 5 6 7 8 9
M R F P S I F T A

GPD promoter | Mating fac...ha leader

2,040 2,060 2,080 2,100 2,120 2,140

TGTTTTGTTCGCTGCTTCTTCTGCTTTGGCTGCTCCAGCTAACACCACCACCGAAGACGAAACCGCTCAAATCCCAGCTGAAGCTGTTATCGACTACTCTGACTTGG
ACAAAACAAGCGACGAAGAAGACGAAACCGACGAGGTCGATTGTGGTGGTGGCTTCTGCTTTGGCGAGTTTAGGGTCGACTTCGACAATAGCTGATGAGACTGAACC

10 11 12 13 14 15 16 17 18 19 20 21 22 23 24 25 26 27 28 29 30 31 32 33 34 35 36 37 38 39 40 41 42 43 44
V L F A A S S A L A A P A N T T T E D E T A Q I P A E A V I D Y S D L

Mating factor alpha leader

2,160 2,180 2,200 2,220 2,240

AAGGTGACTTCGACGCTGCTGCTTTGCCATTGTCTAACTCTACCAACAACGGTTTGTCTTCTACCAACACCACCATCGCTTCTATCGCTGCTAAGGAAGAAGGTGTT
TTCCACTGAAGCTGCGACGACGAAACGGTAACAGATTGAGATGGTTGTTGCCAAACAGAAGATGGTTGTGGTGGTAGCGAAGATAGCGACGATTCCTTCTTCCACAA

45 46 47 48 49 50 51 52 53 54 55 56 57 58 59 60 61 62 63 64 65 66 67 68 69 70 71 72 73 74 75 76 77 78 79 80
E G D F D A A A L P L S N S T N N G L S S T N T T I A S I A A K E E G V

Mating factor alpha leader

2,260 2,280 2,300 2,320 2,340

CAATTGGACAAGAGAGAAGCTAGCGCAGGCGGATCAGACTATAAGGATGACGACGATAAGGCTGGAGGTTCGAGGGGAGTCCCTCACATAGTGATGGTGGATGCTTA
GTTAACCTGTTCTCTCTTCGATCGCGTCCGCCTAGTCTGATATTCCTACTGCTGCTATTCCGACCTCCAAGCTCCCCTCAGGGAGTGTATCACTACCACCTACGAAT

81 82 83 84 85 86 | 1 2 3 1 2 3 4 5 6 7 8 1 2 3 4 1 2 3 4 5 6 7 8 9 10 11 12
Q L D K R E | G G S D Y K D D D D K A G G S R G V P H I V M V D A Y

Mating...eader | SpyTag

2,360 2,380 2,400 2,420 2,440 2,460

CAAAAGGTATAAAGTAACTACCTTGTCTGGCTTATCAGGGGAACAGGGTCCTAGCGGTGACATGACGACAGAGGAGGACAGTGCGACCCACATAAAGTTTAGCAAAA
GTTTTCCATATTTCATTGATGGAACAGACCGAATAGTCCCCTTGTCCCAGGATCGCCACTGTACTGCTGTCTCCTCCTGTCACGCTGGGTGTATTTCAAATCGTTTT

13 14 15 16 2 4 6 8 10 12 14 16 18 20 22 24 26 28 30
K R Y K V T T L S G L S G E Q G P S G D M T T E E D S A T H I K F S K

SpyTag | SpyCatcher

2,480 2,500 2,520 2,540 2,560

```
GAGATGAAGATGGAAGGGAACTAGCTGGAGCGACCATGGAACTTCGTGACAGCTCAGGGAAGACTATATCAACGTGGATCTCAGATGGTCACGTCAAGGACTTCTAT
CTCTACTTCTACCTTCCCTTGATCGACCTCGCTGGTACCTTGAAGCACTGTCGAGTCCCTTCTGATATAGTTGCACCTAGAGTCTACCAGTGCAGTTCCTGAAGATA
32  34  36  38  40  42  44  46  48  50  52  54  56  58  60  62  64  66
R D E D G R E L A G A T M E L R D S S G K T I S T W I S D G H V K D F Y
SpyCatcher
2,580  2,600  2,620  2,640  2,660
```

```
TTGTACCCCGGAAAGTACACATTTGTGGAAACCGCCGCGCCGGATGGCTACGAGGTAGCGACCCCGATAGAATTCACCGTTAACGAGGACGGCCAAGTCACCGTAGA
AACATGGGGCCTTTCATGTGTAAACACCTTTGGCGGCGCGGCCTACCGATGCTCCATCGCTGGGGCTATCTTAAGTGGCAATTGCTCCTGCCGGTTCAGTGGCATCT
68  70  72  74  76  78  80  82  84  86  88  90  92  94  96  98  100  102
L Y P G K Y T F V E T A A P D G Y E V A T P I E F T V N E D G Q V T V D
SpyCatcher
2,680  2,700  2,720  2,740  2,760  2,780
```

```
TGGAGAGGCGACAGAGGGTGATGCCCACACTGGGGGCTCCGGCGGATCTGGTGGGAGCGGATCCTGGTCTCATCCACAATTTGAAAAACAAACATCCTCACCCTCTA
ACCTCTCCGCTGTCTCCCACTACGGGTGTGACCCCCGAGGCCGCCTAGACCACCCTCGCCTAGGACCAGAGTAGGTGTTAAACTTTTTGTTTGTAGGAGTGGGAGAT
104  106  108  110  112  1  2  3  4  5  6  7  8  9  10  11  1  2  3  4  5  6  7  8  2  4  6
G E A T E G D A H T G G S G G S G G S G S W S H P Q F E K Q T S S P S
SpyCatcher  strep tag  649 stalk
2,800  2,820  2,840  2,860  2,880
```

```
CAGAAGCATCAGTATCAACCTCTAGTACCTCTTCCTCTGCTTCACAATCATCTGACCCAACTACAACATCTTCGTCCAGTTCATCGTCTTCCCCATCTTCCCAATCT
GTCTTCGTAGTCATAGTTGGAGATCATGGAGAAGGAGACGAAGTGTTAGTAGACTGGGTTGATGTTGTAGAAGCAGGTCAAGTAGCAGAAGGGGTAGAAGGGTTAGA
8  10  12  14  16  18  20  22  24  26  28  30  32  34  36  38  40  42
T E A S V S T S S T S S S A S Q S S D P T T T S S S S S S S S P S S Q S
649 stalk
2,900  2,920  2,940  2,960  2,980
```

```
GAGGAAATTTCATCATCACCGACGGTCTCAACTACACCATCAACTTCTTCATCATCTTCCTCAATGACTTCAACCACCACAACAAAGTCAATCTCAACTTCCACTAC
CTCCTTTAAAGTAGTAGTGGCTGCCAGAGTTGATGTGGTAGTTGAAGAAGTAGTAGAAGGAGTTACTGAAGTTGGTGGTGTTGTTTCAGTTAGAGTTGAAGGTGATG
44  46  48  50  52  54  56  58  60  62  64  66  68  70  72  74  76  78
E E I S S S P T V S T T P S T S S S S S S M T S T T T T K S I S T S T T
649 stalk
3,000  3,020  3,040  3,060  3,080  3,100
```

```
AAGTTCAGCTCCAGTTACAGATGTGACAGTTTCCTCATCGCCTAGTAAATCTACCTCTACTTCGACAAGTACAGAAACATCTAAAACACCTACTTCAATGACAGAGT
TTCAAGTCGAGGTCAATGTCTACACTGTCAAAGGAGTAGCGGATCATTTAGATGGAGATGAAGCTGTTCATGTCTTTGTAGATTTTGTGGATGAAGTTACTGTCTCA
80  82  84  86  88  90  92  94  96  98  100  102  104  106  108  110  112
S S A P V T D V T V S S S P S K S T S T S T S T E T S K T P T S M T E
649 stalk
3,120  3,140  3,160  3,180  3,200
```

```
ATACATCTAGTACATCGATAATTTCGACTCCAGTTAGTCACTCGCAGACAGGTTTGTCGGCTTCATCAAGTTCATCATCTACAACATCCGGTTCTTCGTCCACTAAA
TATGTAGATCATGTAGCTATTAAAGCTGAGGTCAATCAGTGAGCGTCTGTCCAAACAGCCGAAGTAGTTCAAGTAGTAGATGTTGTAGGCCAAGAAGCAGGTGATTT
114  116  118  120  122  124  126  128  130  132  134  136  138  140  142  144  146  148
Y T S S T S I I S T P V S H S Q T G L S A S S S S S S T T S G S S S T K
649 stalk
3,220  3,240  3,260  3,280  3,300

TCAGAAAGTTCGACAACATCTGGCTCTTCCCAGTCCGTGGAATCAACCTCCAGCCACGCCACTGTTCTTGCTAATTCCGCAGAAATGGTCACAACATCCTCTAGTTC
AGTCTTTCAAGCTGTTGTAGACCGAGAAGGGTCAGGCACCTTAGTTGGAGGTCGGTGCGGTGACAAGAACGATTAAGGCGTCTTTACCAGTGTTGTAGGAGATCAAG
150  152  154  156  158  160  162  164  166  168  170  172  174  176  178  180  182  184
S E S S T T S G S S Q S V E S T S S H A T V L A N S A E M V T T S S S S
649 stalk
3,320  3,340  3,360  3,380  3,400  3,420

ATCCTCAACATCCGAAATGTCATTAACTAGTACTGCTACCAGTGTACCAGTCTCATCTAGTAGCAGTACGACATATTCTACTAGCGCATCTACACAAGCCGTCACTA
TAGGAGTTGTAGGCTTTACAGTAATTGATCATGACGATGGTCACATGGTCAGAGTAGATCATCGTCATGCTGTATAAGATGATCGCGTAGATGTGTTCGGCAGTGAT
186  188  190  192  194  196  198  200  202  204  206  208  210  212  214  216  218  220
S S T S E M S L T S T A T S V P V S S S S S T T Y S T S A S T Q A V T
649 stalk
3,440  3,460  3,480  3,500  3,520

CAACATCTTCTTCCACTGTATCTACAACTTCTTCTAGTACAACGTTAACAAGCGCATTCACACATTCTTCAACCACATCGTCCGACCAGCCACCCAGCGACACTACA
GTTGTAGAAGAAGGTGACATAGATGTTGAAGAAGATCATGTTGCAATTGTTCGCGTAAGTGTGTAAGAAGTTGGTGTAGCAGGCTGGTCGGTGGGTCGCTGTGATGT
222  224  226  228  230  232  234  236  238  240  242  244  246  248  250  252  254  256
T T S S S T V S T T S S S T T L T S A F T H S S T T S S D Q P P S D T T
649 stalk
3,540  3,560  3,580  3,600  3,620

AGTCCATCTACGACACACGAACCTCATGTAACCACTCAGACGTCATCAGAAACATCTTCTTCTAAGTCATCTTCTACTTCTTCTTCAAGTACATCTCAAACCTCTGA
TCAGGTAGATGCTGTGTGCTTGGAGTACATTGGTGAGTCTGCAGTAGTCTTTGTAGAAGAAGATTCAGTAGAAGATGAAGAAGAAGTTCATGTAGAGTTTGGAGACT
258  260  262  264  266  268  270  272  274  276  278  280  282  284  286  288  290  292
S P S T T H E P H V T T Q T S S E T S S S K S S S T S S S S T S Q T S E
649 stalk
3,640  3,660  3,680  3,700  3,720  3,740

GTCTGCAACACCATCCGATTCCGTATCACCTGGAAGTTCTACATCAACATCTTCTAGTAGCACTTCTACTTCCACTTCTATTTCCAGTGGAGAAACGACAACTTCTT
CAGACGTTGTGGTAGGCTAAGGCATAGTGGACCTTCAAGATGTAGTTGTAGAAGATCATCGTGAAGATGAAGGTGAAGATAAAGGTCACCTCTTTGCTGTTGAAGAA
294  296  298  300  302  304  306  308  310  312  314  316  318  320  322  324  326
S A T P S D S V S P G S S T S T S S S S T S T S T S I S S G E T T T S
649 stalk
3,760  3,780  3,800  3,820  3,840
```

```
CTTCTTCATCATCTGCCACGACCACTTCTAACAGCGCAACCTTGTCAGTCTCTACCACACAAACTTCGATTGAAGCCAGTTCATCTACTACATCTACATCTAGTTCA
GAAGAAGTAGTAGACGGTGCTGGTGAAGATTGTCGCGTTGGAACAGTCAGAGATGGTGTGTTTGAAGCTAACTTCGGTCAAGTAGATGATGTAGATGTAGATCAAGT
328 330 332 334 336 338 340 342 344 346 348 350 352 354 356 358 360 362
S S S S S A T T T S N S A T L S V S T T Q T S I E A S S S T T S T S S S
649 stalk
3,860 3,880 3,900 3,920 3,940

ACAATTACAACTTCAAGTAGTAGCGCTCACATATCGTCGAAATCTCAATCTAGTATTACCTATCCCTCTTCCTCGACATCTTCATCTACATCGTCCTCAATTTCTAG
TGTTAATGTTGAAGTTCATCATCGCGAGTGTATAGCAGCTTTAGAGTTAGATCATAATGGATAGGGAGAAGGAGCTGTAGAAGTAGATGTAGCAGGAGTTAAAGATC
364 366 368 370 372 374 376 378 380 382 384 386 388 390 392 394 396 398
T I T T S S S S A H I S S K S Q S S I T Y P S S S T S S S T S S S I S S
649 stalk
3,960 3,980 4,000 4,020 4,040 4,060

CGAATCTGAAAGTTTTGAATCGACATCAGCAGAAGATGCTCCATCAACAGCACCTTCATCAAGTGTCTCTTCTAAGAGTTCTACCTCTACAACATCAAGCACATCGA
GCTTAGACTTTCAAAACTTAGCTGTAGTCGTCTTCTACGAGGTAGTTGTCGTGGAAGTAGTTCACAGAGAAGATTCTCAAGATGGAGATGTTGTAGTTCGTGTAGCT
400 402 404 406 408 410 412 414 416 418 420 422 424 426 428 430 432 434
E S E S F E S T S A E D A P S T A P S S S V S S K S S T S T T S S T S
649 stalk
4,080 4,100 4,120 4,140 4,160

CATCTTCAAGCACTCCATCTCCATCACCATCTTCCGTGAGTTCTTCCTCCACCAGCTCATTGACAACTTCTGCTGTATCAACACCAGCTACCTCTCATTCTCAAAGT
GTAGAAGTTCGTGAGGTAGAGGTAGTGGTAGAAGGCACTCAAGAAGGAGGTGGTCGAGTAACTGTTGAAGACGACATAGTTGTGGTCGATGGAGAGTAAGAGTTTCA
436 438 440 442 444 446 448 450 452 454 456 458 460 462 464 466 468 470
T S S S T P S P S P S S V S S S S T S S L T T S A V S T P A T S H S Q S
649 stalk
4,180 4,200 4,220 4,240 4,260 4,280

ACTGTAGTAACCACCACTACTATTACTACATCAACAGGTCCAGTGATGTCTACGACAACAGCTTATTCTTCTAGTTCTACTAGCAGCTCGGAATCTTCTGAGGTTCA
TGACATCATTGGTGGTGATGATAATGATGTAGTTGTCCAGGTCACTACAGATGCTGTTGTCGAATAAGAAGATCAAGATGATCGTCGAGCCTTAGAAGACTCCAAGT
472 474 476 478 480 482 484 486 488 490 492 494 496 498 500 502 504 506
T V V T T T T I T T S T G P V M S T T T A Y S S S S T S S S E S S E V Q
649 stalk
4,300 4,320 4,340 4,360 4,380

GTCTGTCATGTCATCTACGCCTAGTTCAACATCAACAACAACCAGTTCGGAATCTACTTCATCTAGCTCCACAGCTTCTACCTCACCATCAACCTCGCAAACTTTCG
CAGACAGTACAGTAGATGCGGATCAAGTTGTAGTTGTTGTTGGTCAAGCCTTAGATGAAGTAGATCGAGGTGTCGAAGATGGAGTGGTAGTTGGAGCGTTTGAAAGC
508 510 512 514 516 518 520 522 524 526 528 530 532 534 536 538 540
S V M S S T P S S T S T T T S S E S T S S S S T A S T S P S T S Q T F
649 stalk
4,400 4,420 4,440 4,460 4,480
```

AAACTTCTCCTACTATAGGAGGTGTCCCCTCAACCACTTCATTTGTCTCTACGCCAACAACGAAATTGTCGCACACTACTTCCACTATGACAGCACAGTCCGATAGT

TTTGAAGAGGATGATATCCTCCACAGGGGAGTTGGTGAAGTAAACAGAGATGCGGTTGTTGCTTTAACAGCGTGTGATGAAGGTGATACTGTCGTGTCAGGCTATCA

542 544 546 548 550 552 554 556 558 560 562 564 566 568 570 572 574 576

E T S P T I G G V P S T T S F V S T P T T K L S H T T S T M T A Q S D S

649 stalk

4,500 4,520 4,540 4,560 4,580 4,600

AAGTCTACCCACTCCTCAAGCACATCGACAGAAGATAAATCATCCACTGCTTCTGCAGTTGACGAAAGCACTACAACATCCACTTCCACGGAGTCTACTACATCAGT

TTCAGATGGGTGAGGAGTTCGTGTAGCTGTCTTCTATTTAGTAGGTGACGAAGACGTCAACTGCTTTCGTGATGTTGTAGGTGAAGGTGCCTCAGATGATGTAGTCA

578 580 582 584 586 588 590 592 594 596 598 600 602 604 606 608 610 612

K S T H S S S T S T E D K S S T A S A V D E S T T T S T S T E S T T S V

649 stalk

4,620 4,640 4,660 4,680 4,700

AACATCAGGCACCTCCCATTCCGCTAAAGAATCTTCGTCAAATTCTAAGGTGTATAGTTCACAGACAGCACACTCATCCATAAGTGTTGCATCATCACCTAGTACAA

TTGTAGTCCGTGGAGGGTAAGGCGATTTCTTAGAAGCAGTTTAAGATTCCACATATCAAGTGTCTGTCGTGTGAGTAGGTATTCACAACGTAGTAGTGGATCATGTT

614 616 618 620 622 624 626 628 630 632 634 636 638 640 642 644 646 648

T S G T S H S A K E S S S N S K V Y S S Q T A H S S I S V A S S P S T

649 stalk

4,720 4,740 4,760 4,780 4,800

AGGGCGCCCAAATCCAATCTTCTATGGTTGAAATCTCTACCTACGCTGGTTCTGCTAACTCTGTTAACGCTGGTGCTGGTGCTGGTGCTTTGTTCTTGTTGTTGTCT

TCCCGCGGGTTTAGGTTAGAAGATACCAACTTTAGAGATGGATGCGACCAAGACGATTGAGACAATTGCGACCACGACCACGACCACGAAACAAGAACAACAACAGA

650 652 654 656 658 660 662 664 666 668 670 672 674 676 678 680 682 684

K G A Q I Q S S M V E I S T Y A G S A N S V N A G A G A G A L F L L L S

GPI anchor

4,820 4,840 4,860 4,880 4,900 4,920

TTGGCTATCATCTAATGATTAATTAACTCGAGATCTGATAACAACAGTGTAGATGTAACAAAATCGACTTTGTTCCCACTGTACTTTTAGCTCGTACAAAATACAAT

AACCGATAGTAGATTACTAATTAATTGAGCTCTAGACTATTGTTGTCACATCTACATTGTTTTAGCTGAAACAAGGGTGACATGAAAATCGAGCATGTTTTATGTTA

686 688 1

L A I I *

GPI...or

alpha factor terminator

4,940 4,960 4,980 5,000 5,020

ATACTTTTCATTTCTCCGTAAACAACATGTTTTCCCATGTAATATCCTTTTCTATTTTTCGTTCCGTTACCAACTTTACACATACTTTATATAGCTATTCACTTCTA

TATGAAAAGTAAAGAGGCATTTGTTGTACAAAAGGGTACATTATAGGAAAAGATAAAAAGCAAGGCAATGGTTGAAATGTGTATGAAATATATCGATAAGTGAAGAT

alpha factor terminator

5,040 5,060 5,080 5,100 5,120

TACACTAAAAAAACTAAGACAATTTTAATTTTGCTGCCTGCCATATTTCAATTTGTTATAAATTCCTATAATTTATCCTATTAGTAGCTAAAAAAAGATGAATGTGAA

ATGTGATTTTTTTGATTCTGTTAAAATTAAAACGACGGACGGTATAAAGTTAAACAATATTTAAGGATATTAAATAGGATAATCATCGATTTTTTTCTACTTACACTT

alpha factor terminator

5,140 5,160 5,180 5,200 5,220 5,240

TCGAATCCTAAGAGAATTGGCCGGTACCCAATTCGCCCTATAGTGAGTCGTATTACGCGCGCTCACTGGCCGTCGTTTTACAACGTCGTGACTGGGAAAACCCTGGC
AGCTTAGGATTCTCTTAACCGGCCATGGGTTAAGCGGGATATCACTCAGCATAATGCGCGCGAGTGACCGGCAGCAAAATGTTGCAGCACTGACCCTTTTGGGACCG

alpha ...nator T7 M13-fwd

5,260 5,280 5,300 5,320 5,340

GTTACCCAACTTAATCGCCTTGCAGCACATCCCCCTTTCGCCAGCTGGCGTAATAGCGAAGAGGCCCGCACCGATCGCCCTTCCCAACAGTTGCGCAGCCTGAATGG
CAATGGGTTGAATTAGCGGAACGTCGTGTAGGGGGAAAGCGGTCGACCGCATTATCGCTTCTCCGGGCGTGGCTAGCGGGAAGGGTTGTCAACGCGTCGGACTTACC

LacZ alpha

5,360 5,380 5,400 5,420 5,440

CGAATGGACGCGCCCTGTAGCGGCGCATTAAGCGCGGCGGGTGTGGTGGTTACGCGCAGCGTGACCACTACACTTGCCAGCGCCCTAGCGCCCGCTCCTTTCGCTTT
GCTTACCTGCGCGGGACATCGCCGCGTAATTCGCGCCGCCCACACCACCAATGCGCGTCGCACTGGTGATGTGAACGGTCGCGGGATCGCGGGCGAGGAAAGCGAAA

5,460 5,480 5,500 5,520 5,540 5,560

CTTCCCTTCCTTTCTCGCCACGTTCGCCGGCTTTCCCCGTCAAGCTCTAAATCGGGGGCTCCCTTTAGGGTTCCGATTTAGTGCTTTACGGCACCTCGACCCCAAAA
GAAGGGAAGGAAAGAGCGGTGCAAGCGGCCGAAAGGGGCAGTTCGAGATTTAGCCCCCGAGGGAAATCCCAAGGCTAAATCACGAAATGCCGTGGAGCTGGGGTTTT

5,580 5,600 5,620 5,640 5,660

AACTTGATTAGGGTGATGGTTCACGTAGTGGGCCATCGCCCTGATAGACGGTTTTTCGCCCTTTGACGTTGGAGTCCACGTTCTTTAATAGTGGACTCTTGTTCCAA
TTGAACTAATCCCACTACCAAGTGCATCACCCGGTAGCGGGACTATCTGCCAAAAAGCGGGAAACTGCAACCTCAGGTGCAAGAAATTATCACCTGAGAACAAGGTT

5,680 5,700 5,720 5,740 5,760

ACTGGAACAACACTCAACCCTATCTCGGTCTATTCTTTTGATTTATAAGGGATTTTGCCGATTTCGGCCTATTGGTTAAAAAATGAGCTGATTTAACAAAAATTTAA
TGACCTTGTTGTGAGTTGGGATAGAGCCAGATAAGAAAACTAAATATTCCCTAAAACGGCTAAAGCCGGATAACCAATTTTTTACTCGACTAAATTGTTTTTAAATT

5,780 5,800 5,820 5,840 5,860 5,880

CGCGAATTTTAACAAAATATTAACGCTTACAATTTCCTGATGCGGTATTTTCTCCTTACGCATCTGTGCGGTATTTCACACCGCATAGGGTAATAACTGATATAATT
GCGCTTAAAATTGTTTTATAATTGCGAATGTTAAAGGACTACGCCATAAAAGAGGAATGCGTAGACACGCCATAAAGTGTGGCGTATCCCATTATTGACTATATTAA

5,900 5,920 5,940 5,960 5,980

AAATTGAAGCTCTAATTTGTGAGTTTAGTATACATGCATTTACTTATAATACAGTTTTTTAAGCAAGGATTTTCTTAACTTCTTCGGCGACAGCATCACCGACTTCG
TTTAACTTCGAGATTAAACACTCAAATCATATGTACGTAAATGAATATTATGTCAAAAAATTCGTTCCTAAAAGAATTGAAGAAGCCGCTGTCGTAGTGGCTGAAGC

LEU2

6,000 6,020 6,040 6,060 6,080

GTGGTACTGTTGGAACCACCTAAATCACCAGTTCTGATACCTGCATCCAAAACCTTTTTAACTGCATCTTCAATGGCCTTACCTTCTTCAGGCAAGTTCAATGACAA
CACCATGACAACCTTGGTGGATTTAGTGGTCAAGACTATGGACGTAGGTTTTGGAAAAATTGACGTAGAAGTTACCGGAATGGAAGAAGTCCGTTCAAGTTACTGTT

LEU2

6,100 6,120 6,140 6,160 6,180 6,200

TTTCAACATCATTGCAGCAGACAAGATAGTGGCGATAGGGTTGACCTTATTCTTTGGCAAATCTGGAGCAGAACCGTGGCATGGTTCGTACAAACCAAATGCGGTGT
AAAGTTGTAGTAACGTCGTCTGTTCTATCACCGCTATCCCAACTGGAATAAGAAACCGTTTAGACCTCGTCTTGGCACCGTACCAAGCATGTTTGGTTTACGCCACA
LEU2
6,220 6,240 6,260 6,280 6,300

TCTTGTCTGGCAAAGAGGCCAAGGACGCAGATGGCAACAAACCCAAGGAACCTGGGATAACGGAGGCTTCATCGGAGATGATATCACCAAACATGTTGCTGGTGATT
AGAACAGACCGTTTCTCCGGTTCCTGCGTCTACCGTTGTTTGGGTTCCTTGGACCCTATTGCCTCCGAAGTAGCCTCTACTATAGTGGTTTGTACAACGACCACTAA
LEU2
6,320 6,340 6,360 6,380 6,400 6,420

ATAATACCATTTAGGTGGGTTGGGTTCTTAACTAGGATCATGGCGGCAGAATCAATCAATTGATGTTGAACCTTCAATGTAGGGAATTCGTTCTTGATGGTTTCCTC
TATTATGGTAAATCCACCCAACCCAAGAATTGATCCTAGTACCGCCGTCTTAGTTAGTTAACTACAACTTGGAAGTTACATCCCTTAAGCAAGAACTACCAAAGGAG
LEU2
6,440 6,460 6,480 6,500 6,520

CACAGTTTTTCTCCATAATCTTGAAGAGGCCAAAACATTAGCTTTATCCAAGGACCAAATAGGCAATGGTGGCTCATGTTGTAGGGCCATGAAAGCGGCCATTCTTG
GTGTCAAAAAGAGGTATTAGAACTTCTCCGGTTTTGTAATCGAAATAGGTTCCTGGTTTATCCGTTACCACCGAGTACAACATCCCGGTACTTTCGCCGGTAAGAAC
LEU2
6,540 6,560 6,580 6,600 6,620

TGATTCTTTGCACTTCTGGAACGGTGTATTGTTCACTATCCCAAGCGACACCATCACCATCGTCTTCCTTTCTCTTACCAAAGTAAATACCTCCCACTAATTCTCTG
ACTAAGAAACGTGAAGACCTTGCCACATAACAAGTGATAGGGTTCGCTGTGGTAGTGGTAGCAGAAGGAAAGAGAATGGTTTCATTTATGGAGGGTGATTAAGAGAC
LEU2
6,640 6,660 6,680 6,700 6,720 6,740

ACAACAACGAAGTCAGTACCTTTAGCAAATTGTGGCTTGATTGGAGATAAGTCTAAAAGAGAGTCGGATGCAAAGTTACATGGTCTTAAGTTGGCGTACAATTGAAG
TGTTGTTGCTTCAGTCATGGAAATCGTTTAACACCGAACTAACCTCTATTCAGATTTTCTCTCAGCCTACGTTTCAATGTACCAGAATTCAACCGCATGTTAACTTC
LEU2
6,760 6,780 6,800 6,820 6,840

TTCTTTACGGATTTTTAGTAAACCTTGTTCAGGTCTAACACTACCGGTACCCCATTTAGGACCACCCACAGCACCTAACAAAACGGCATCAGCCTTCTTGGAGGCTT
AAGAAATGCCTAAAAATCATTTGGAACAAGTCCAGATTGTGATGGCCATGGGGTAAATCCTGGTGGGTGTCGTGGATTGTTTTGCCGTAGTCGGAAGAACCTCCGAA
LEU2
6,860 6,880 6,900 6,920 6,940

CCAGCGCCTCATCTGGAAGTGGAACACCTGTAGCATCGATAGCAGCACCACCAATTAAATGATTTTCGAAATCGAACTTGACATTGGAACGAACATCAGAAATAGCT
GGTCGCGGAGTAGACCTTCACCTTGTGGACATCGTAGCTATCGTCGTGGTGGTTAATTTACTAAAAGCTTTAGCTTGAACTGTAACCTTGCTTGTAGTCTTTATCGA
LEU2
6,960 6,980 7,000 7,020 7,040 7,060

TTAAGAACCTTAATGGCTTCGGCTGTGATTTCTTGACCAACGTGGTCACCTGGCAAAACGACGATCTTCTTAGGGGCAGACATTAGAATGGTATATCCTTGAAATAT
AATTCTTGGAATTACCGAAGCCGACACTAAAGAACTGGTTGCACCAGTGGACCGTTTTGCTGCTAGAAGAATCCCCGTCTGTAATCTTACCATATAGGAACTTTATA

LEU2

7,080 7,100 7,120 7,140 7,160

ATATATATATATNTNGCTGAAATGTAAAAGGTAAGAAAAGTTAGAAAGTAAGACGATTGCTAACCACCTATTGGAAAAAAACAATAGGTCCTTAAATAATATTGTCAA
TATATATATATANANCGACTTTACATTTTCCATTCTTTTCAATCTTTCATTCTGCTAACGATTGGTGGATAACCTTTTTTTGTTATCCAGGAATTTATTATAACAGTT

7,180 7,200 7,220 7,240 7,260

CTTCAAGTATTGTGATGCAAGCATTTAGTCATGAACGCTTCTCTATTCTATATGAAAAGCCGGTTCCGGCGCTCTCACCTTTCCTTTTTCTCCCAATTTTTCAGTTG
GAAGTTCATAACACTACGTTCGTAAATCAGTACTTGCGAAGAGATAAGATATACTTTTCGGCCAAGGCCGCGAGAGTGGAAAGGAAAAAGAGGGTTAAAAAGTCAAC

7,280 7,300 7,320 7,340 7,360 7,380

AAAAAGGTATATGCGTCAGGCGACCTCTGAAATTAACAAAAAATTTCCAGTCATCGAATTTGATTCTGTGCGATAGCGCCCCTGTGTGTTCTCGTTATGTTGAGGAA
TTTTTCCATATACGCAGTCCGCTGGAGACTTTAATTGTTTTTTAAAGGTCAGTAGCTTAAACTAAGACACGCTATCGCGGGGACACACAAGAGCAATACAACTCCTT

7,400 7,420 7,440 7,460 7,480

AAAAATAATGGTTGCTAAGAGATTCGAACTCTTGCATCTTACGATACCTGAGTATTCCCACAGTTTGAAAAGCTGTGGTATGGTGCACTCTCAGTACAATCTGCTCT
TTTTTATTACCAACGATTCTCTAAGCTTGAGAACGTAGAATGCTATGGACTCATAAGGGTGTCAAACTTTTCGACACCATACCACGTGAGAGTCATGTTAGACGAGA

7,500 7,520 7,540 7,560 7,580

GATGCCGCATAGTTAAGCCAGCCCCGACACCCGCCAACACCCGCTGACGCGCCCTGACGGGCTTGTCTGCTCCCGGCATCCGCTTACAGACAAGCTGTGACCGTCTC
CTACGGCGTATCAATTCGGTCGGGGCTGTGGGCGGTTGTGGGCGACTGCGCGGGACTGCCCGAACAGACGAGGGCCGTAGGCGAATGTCTGTTCGACACTGGCAGAG

7,600 7,620 7,640 7,660 7,680 7,700

CGGGAGCTGCATGTGTCAGAGGTTTTCACCGTCATCACCGAAACGCGCGAGACGAAAGGGCCTCGTGATACGCCTATTTTTATAGGTTAATGTCATGATAATAATGG
GCCCTCGACGTACACAGTCTCCAAAAGTGGCAGTAGTGGCTTTGCGCGCTCTGCTTTCCCGGAGCACTATGCGGATAAAAATATCCAATTACAGTACTATTATTACC

7,720 7,740 7,760 7,780 7,800

TTTCTTAGTATGATCCAATATCAAAGGAAATGATAGCATTGAAGGATGAGACTAATCCAATTGAGGAGTGGCAGCATATAGAACAGCTAAAGGGTAGTGCTGAAGGA
AAAGAATCATACTAGGTTATAGTTTCCTTTACTATCGTAACTTCCTACTCTGATTAGGTTAACTCCTCACCGTCGTATATCTTGTCGATTTCCCATCACGACTTCCT

2 micron origin

7,820 7,840 7,860 7,880 7,900

AGCATACGATACCCCGCATGGAATGGGATAATATCACAGGAGGTACTAGACTACCTTTCATCCTACATAAATAGACGCATATAAGTACGCATTTAAGCATAAACACG
TCGTATGCTATGGGGCGTACCTTACCCTATTATAGTGTCCTCCATGATCTGATGGAAAGTAGGATGTATTTATCTGCGTATATTCATGCGTAAATTCGTATTTGTGC

2 micron origin

7,920 7,940 7,960 7,980 8,000 8,020

CACTATGCCGTTCTTCTCATGTATATATATATACAGGCAACACGCAGATATAGGTGCGACGTGAACAGTGAGCTGTATGTGCGCAGCTCGCGTTGCATTTTCGGAAG
GTGATACGGCAAGAAGAGTACATATATATATATGTCCGTTGTGCGTCTATATCCACGCTGCACTTGTCACTCGACATACACGCGTCGAGCGCAACGTAAAAGCCTTC

2 micron origin

8,040 8,060 8,080 8,100 8,120

CGCTCGTTTTCGGAAACGCTTTGAAGTTCCTATTCCGAAGTTCCTATTCTCTAGAAAGTATAGGAACTTCAGAGCGCTTTTGAAAACCAAAAGCGCTCTGAAGACGC
GCGAGCAAAAGCCTTTGCGAAACTTCAAGGATAAGGCTTCAAGGATAAGAGATCTTTCATATCCTTGAAGTCTCGCGAAAACTTTTGGTTTTCGCGAGACTTCTGCG

FRT
2 micron origin

8,140 8,160 8,180 8,200 8,220

ACTTTCAAAAAACCAAAAACGCACCGGACTGTAACGAGCTACTAAAATATTGCGAATACCGCTTCCACAAACATTGCTCAAAAGTATCTCTTTGCTATATATCTCTG
TGAAAGTTTTTTGGTTTTTGCGTGGCCTGACATTGCTCGATGATTTTATAACGCTTATGGCGAAGGTGTTTGTAACGAGTTTTCATAGAGAAACGATATATAGAGAC

2 micron origin

8,240 8,260 8,280 8,300 8,320 8,340

TGCTATATCCCTATATAACCTACCCATCCACCTTTCGCTCCTTGAACTTGCATCTAAACTCGACCTCTACATTTTTTATGTTTATCTCTAGTATTACTCTTTAGACA
ACGATATAGGGATATATTGGATGGGTAGGTGGAAAGCGAGGAACTTGAACGTAGATTTGAGCTGGAGATGTAAAAAATACAAATAGAGATCATAATGAGAAATCTGT

2 micron origin

8,360 8,380 8,400 8,420 8,440

AAAAAATTGTAGTAAGAACTATTCATAGAGTGAATCGAAAACAATACGAAAATGTAAACATTTCCTATACGTAGTATATAGAGACAAAATAGAAGAAACCGTTCATA
TTTTTTAACATCATTCTTGATAAGTATCTCACTTAGCTTTTGTTATGCTTTTACATTTGTAAAGGATATGCATCATATATCTCTGTTTTATCTTCTTTGGCAAGTAT

2 micron origin

8,460 8,480 8,500 8,520 8,540 8,560

ATTTTCTGACCAATGAAGAATCATCAACGCTATCACTTTCTGTTCACAAAGTATGCGCAATCCACATCGGTATAGAATATAATCGGGGATGCCTTTATCTTGAAAAA
TAAAAGACTGGTTACTTCTTAGTAGTTGCGATAGTGAAAGACAAGTGTTTCATACGCGTTAGGTGTAGCCATATCTTATATTAGCCCCTACGGAAATAGAACTTTTT

2 micron origin

8,580 8,600 8,620 8,640 8,660

ATGCACCCGCAGCTTCGCTAGTAATCAGTAAACGCGGGAAGTGGAGTCAGGCTTTTTTTATGGAAGAGAAAATAGACACCAAAGTAGCCTTCTTCTAACCTTAACGG
TACGTGGGCGTCGAAGCGATCATTAGTCATTTGCGCCCTTCACCTCAGTCCGAAAAAAATACCTTCTCTTTTATCTGTGGTTTCATCGGAAGAAGATTGGAATTGCC

2 micron origin

8,680 8,700 8,720 8,740 8,760

ACCTACAGTGCAAAAAGTTATCAAGAGACTGCATTATAGAGCGCACAAAGGAGAAAAAAAGTAATCTAAGATGCTTTGTTAGAAAAATAGCGCTCTCGGGATGCATT
TGGATGTCACGTTTTTCAATAGTTCTCTGACGTAATATCTCGCGTGTTTCCTCTTTTTTTCATTAGATTCTACGAAACAATCTTTTTATCGCGAGAGCCCTACGTAA

2 micron origin

8,780 8,800 8,820 8,840 8,860 8,880

TTTGTAGAACAAAAAAGAAGTATAGATTCTTTGTTGGTAAAATAGCGCTCTCGCGTTGCATTTCTGTTCTGTAAAAATGCAGCTCAGATTCTTTGTTTGAAAAATTA
AAACATCTTGTTTTTTCTTCATATCTAAGAAACAACCATTTTATCGCGAGAGCGCAACGTAAAGACAAGACATTTTTACGTCGAGTCTAAGAAACAAACTTTTTAAT

2 micron origin

8,900 8,920 8,940 8,960 8,980

GCGCTCTCGCGTTGCATTTTTGTTTTACAAAAATGAAGCACAGATTCTTCGTTGGTAAAATAGCGCTTTCGCGTTGCATTTCTGTTCTGTAAAAATGCAGCTCAGAT
CGCGAGAGCGCAACGTAAAAACAAAATGTTTTTACTTCGTGTCTAAGAAGCAACCATTTTATCGCGAAAGCGCAACGTAAAGACAAGACATTTTTACGTCGAGTCTA
2 micron origin
9,000 9,020 9,040 9,060 9,080

TCTTTGTTTGAAAAATTAGCGCTCTCGCGTTGCATTTTTGTTCTACAAAATGAAGCACAGATGCTTCGTTCAGGTGGCACTTTTCGGGGAAATGTGCGCGGAACCCC
AGAAACAAACTTTTTAATCGCGAGAGCGCAACGTAAAAACAAGATGTTTTACTTCGTGTCTACGAAGCAAGTCCACCGTGAAAAGCCCCTTTACACGCGCCTTGGGG
2 micron origin
9,100 9,120 9,140 9,160 9,180 9,200

TATTTGTTTATTTTTCTAAATACATTCAAATATGTATCCGCTCATGAGACAATAACCCTGATAAATGCTTCAATAATATTGAAAAAGGAAGAGTATGAGTATTCAAC
ATAAACAAATAAAAAGATTTATGTAAGTTTATACATAGGCGAGTACTCTGTTATTGGGACTATTTACGAAGTTATTATAACTTTTTCCTTCTCATACTCATAAGTTG
9,220 9,240 9,260 9,280 9,300

ATTTCCGTGTCGCCCTTATTCCCTTTTTTGCGGCATTTTGCCTTCCTGTTTTTGCTCACCCAGAAACGCTGGTGAAAGTAAAAGATGCTGAAGATCAGTTGGGTGCA
TAAAGGCACAGCGGGAATAAGGGAAAAAACGCCGTAAAACGGAAGGACAAAAACGAGTGGGTCTTTGCGACCACTTTCATTTTCTACGACTTCTAGTCAACCCACGT
9,320 9,340 9,360 9,380 9,400

CGAGTGGGTTACATCGAACTGGATCTCAACAGCGGTAAGATCCTTGAGAGTTTTCGCCCCGAAGAACGTTTTCCAATGATGAGCACTTTTAAAGTTCTGCTATGTGG
GCTCACCCAATGTAGCTTGACCTAGAGTTGTCGCCATTCTAGGAACTCTCAAAAGCGGGGCTTCTTGCAAAAGGTTACTACTCGTGAAAATTTCAAGACGATACACC
AmpR
9,420 9,440 9,460 9,480 9,500 9,520

CGCGGTATTATCCCGTATTGACGCCGGGCAAGAGCAACTCGGTCGCCGCATACACTATTCTCAGAATGACTTGGTTGAGTACTCACCAGTCACAGAAAAGCATCTTA
GCGCCATAATAGGGCATAACTGCGGCCCGTTCTCGTTGAGCCAGCGGCGTATGTGATAAGAGTCTTACTGAACCAACTCATGAGTGGTCAGTGTCTTTTCGTAGAAT
AmpR
9,540 9,560 9,580 9,600 9,620

CGGATGGCATGACAGTAAGAGAATTATGCAGTGCTGCCATAACCATGAGTGATAACACTGCGGCCAACTTACTTCTGACAACGATCGGAGGACCGAAGGAGCTAACC
GCCTACCGTACTGTCATTCTCTTAATACGTCACGACGGTATTGGTACTCACTATTGTGACGCCGGTTGAATGAAGACTGTTGCTAGCCTCCTGGCTTCCTCGATTGG
AmpR
9,640 9,660 9,680 9,700 9,720

GCTTTTTTGCACAACATGGGGGATCATGTAACTCGCCTTGATCGTTGGGAACCGGAGCTGAATGAAGCCATACCAAACGACGAGCGTGACACCACGATGCCTGTAGC
CGAAAAAACGTGTTGTACCCCCTAGTACATTGAGCGGAACTAGCAACCCTTGGCCTCGACTTACTTCGGTATGGTTTGCTGCTCGCACTGTGGTGCTACGGACATCG
AmpR
9,740 9,760 9,780 9,800 9,820 9,840

AATGGCAACAACGTTGCGCAAACTATTAACTGGCGAACTACTTACTCTAGCTTCCCGGCAACAATTAATAGACTGGATGGAGGCGGATAAAGTTGCAGGACCACTTC
TTACCGTTGTTGCAACGCGTTTGATAATTGACCGCTTGATGAATGAGATCGAAGGGCCGTTGTTAATTATCTGACCTACCTCCGCCTATTTCAACGTCCTGGTGAAG
AmpR
9,860 9,880 9,900 9,920 9,940

TGCGCTCGGCCCTTCCGGCTGGCTGGTTTATTGCTGATAAATCTGGAGCCGGTGAGCGTGGGTCTCGCGGTATCATTGCAGCACTGGGGCCAGATGGTAAGCCCTCC
ACGCGAGCCGGGAAGGCCGACCGACCAAATAACGACTATTTAGACCTCGGCCACTCGCACCCAGAGCGCCATAGTAACGTCGTGACCCCGGTCTACCATTCGGGAGG

AmpR

9,960 9,980 10,000 10,020 10,040

CGTATCGTAGTTATCTACACGACGGGGAGTCAGGCAACTATGGATGAACGAAATAGACAGATCGCTGAGATAGGTGCCTCACTGATTAAGCATTGGTAACTGTCAGA
GCATAGCATCAATAGATGTGCTGCCCCTCAGTCCGTTGATACCTACTTGCTTTATCTGTCTAGCGACTCTATCCACGGAGTGACTAATTCGTAACCATTGACAGTCT

AmpR

10,060 10,080 10,100 10,120 10,140 10,160

CCAAGTTTACTCATATATACTTTAGATTGATTTAAAACTTCATTTTTAATTTAAAAGGATCTAGGTGAAGATCCTTTTTGATAATCTCATGACCAAAATCCCTTAAC
GGTTCAAATGAGTATATATGAAATCTAACTAAATTTTGAAGTAAAAATTAAATTTTCCTAGATCCACTTCTAGGAAAAACTATTAGAGTACTGGTTTTAGGGAATTG

10,180 10,200 10,220 10,240 10,260

GTGAGTTTTCGTTCCACTGAGCGTCAGACCCCGTAGAAAAGATCAAAGGATCTTCTTGAGATCCTTTTTTTCTGCGCGTAATCTGCTGCTTGCAAACAAAAAAACCA
CACTCAAAAGCAAGGTGACTCGCAGTCTGGGGCATCTTTTCTAGTTTCCTAGAAGAACTCTAGGAAAAAAAGACGCGCATTAGACGACGAACGTTTGTTTTTTTGGT

10,280 10,300 10,320 10,340 10,360

CCGCTACCAGCGGTGGTTTGTTTGCCGGATCAAGAGCTACCAACTCTTTTTCCGAAGGTAACTGGCTTCAGCAGAGCGCAGATACCAAATACTGTTCTTCTAGTGTA
GGCGATGGTCGCCACCAAACAAACGGCCTAGTTCTCGATGGTTGAGAAAAAGGCTTCCATTGACCGAAGTCGTCTCGCGTCTATGGTTTATGACAAGAAGATCACAT

10,400 10,420 10,440 10,460 10,480

GCCGTAGTTAGGCCACCACTTCAAGAACTCTGTAGCACCGCCTACATACCTCGCTCTGCTAATCCTGTTACCAGTGGCTGCTGCCAGTGGCGATAAGTCGTGTCTTA
CGGCATCAATCCGGTGGTGAAGTTCTTGAGACATCGTGGCGGATGTATGGAGCGAGACGATTAGGACAATGGTCACCGACGACGGTCACCGCTATTCAGCACAGAAT

10,500 10,520 10,540 10,560 10,580

CCGGGTTGGACTCAAGACGATAGTTACCGGATAAGGCGCAGCGGTCGGGCTGAACGGGGGGTTCGTGCACACAGCCCAGCTTGGAGCGAACGACCT
GGCCCAACCTGAGTTCTGCTATCAATGGCCTATTCCGCGTCGCCAGCCCGACTTGCCCCCCAAGCACGTGTGTCGGGTCGAACCTCGCTTGCTGGA

10,600 10,610 10,620 10,630 10,640 10,650 10,660 10,670 10,680

ST/SC ligation / pAnchor_N.C. of ST/SC coupling...

ACACCGAACTGAGATACCTACAGCGTGAGCTATGAGAAAGCGCCACGCTTCCCGAAGGGAGAAAGGCGGACAGGTATCCGGTAAGCGGCAGGGTCGGAACAGGAGAG
TGTGGCTTGACTCTATGGATGTCGCACTCGATACTCTTTCGCGGTGCGAAGGGCTTCCCTCTTTCCGCCTGTCCATAGGCCATTCGCCGTCCCAGCCTTGTCCTCTC

20 40 60 80 100

CGCACGAGGGAGCTTCCAGGGGGAAACGCCTGGTATCTTTATAGTCCTGTCGGGTTTCGCCACCTCTGACTTGAGCGTCGATTTTTGTGATGCTCGTCAGGGGGGCG
GCGTGCTCCCTCGAAGGTCCCCCTTTGCGGACCATAGAAATATCAGGACAGCCCAAAGCGGTGGAGACTGAACTCGCAGCTAAAAACACTACGAGCAGTCCCCCCGC

120 140 160 180 200

GAGCCTATGGAAAAACGCCAGCAACGCGGCCTTTTTACGGTTCCTGGCCTTTTGCTGGCCTTTTGCTCACATGTTCTTTCCTGCGTTATCCCCTGATTCTGTGGATA
CTCGGATACCTTTTTGCGGTCGTTGCGCCGGAAAAATGCCAAGGACCGGAAAACGACCGGAAAACGAGTGTACAAGAAAGGACGCAATAGGGGACTAAGACACCTAT

220 240 260 280 300 320

ACCGTATTACCGCCTTTGAGTGAGCTGATACCGCTCGCCGCAGCCGAACGACCGAGCGCAGCGAGTCAGTGAGCGAGGAAGCGGAAGAGCGCCCAATACGCAAACCG
TGGCATAATGGCGGAAACTCACTCGACTATGGCGAGCGGCGTCGGCTTGCTGGCTCGCGTCGCTCAGTCACTCGCTCCTTCGCCTTCTCGCGGGTTATGCGTTTGGC

340 360 380 400 420

CCTCTCCCCGCGCGTTGGCCGATTCATTAATGCAGCTGGCACGACAGGTTTCCCGACTGGAAAGCGGGCAGTGAGCGCAACGCAATTAATGTGAGTTAGCTCACTCA
GGAGAGGGGCGCGCAACCGGCTAAGTAATTACGTCGACCGTGCTGTCCAAAGGGCTGACCTTTCGCCCGTCACTCGCGTTGCGTTAATTACACTCAATCGAGTGAGT

440 460 480 500 520

TTAGGCACCCCAGGCTTTACACTTTATGCTTCCGGCTCGTATGTTGTGTGGAATTGTGAGCGGATAACAATTTCACACAGGAAACAGCTATGACCATGATTACGCCA
AATCCGTGGGGTCCGAAATGTGAAATACGAAGGCCGAGCATACAACACACCTTAACACTCGCCTATTGTTAAAGTGTGTCCTTTGTCGATACTGGTACTAATGCGGT

lac promoter LacO M13-rev

540 560 580 600 620 640

AGCGCGCAATTAACCCTCACTAAAGGGAACAAAAGCTGGAGCTAGTATACTCTTTCTTCAACAATTAAATACTCTCGGTAGCCAAGTTGGTTTAAGGCGCAAGACTG
TCGCGCGTTAATTGGGAGTGATTTCCCTTGTTTTCGACCTCGATCATATGAGAAAGAAGTTGTTAATTTATGAGAGCCATCGGTTCAACCAAATTCCGCGTTCTGAC

T3
T3 promoter

660 680 700 720 740

TAATTTATCACTACGAAATCTTGAGATCGGGCGTTCGACTCGCCCCCGGGAGAGATGGCCGGCATGGTCCCAGCCTCCTCGCTGGCGCCGGCTGGGCAACACCTTCG
ATTAAATAGTGATGCTTTAGAACTCTAGCCCGCAAGCTGAGCGGGGGCCCTCTCTACCGGCCGTACCAGGGTCGGAGGAGCGACCGCGGCCGACCCGTTGTGGAAGC

760 780 800 820 840

GGTGGCGAATGGGACTTTTTATGTGCGTATTGCTTTCAGTTTTAGAGCTAGAAATAGCAAGTTAAAATAAGGCTAGTCCGTTATCAACTTGAAAAAGTGGCACCGAG
CCACCGCTTACCCTGAAAAATACACGCATAACGAAAGTCAAAATCTCGATCTTTATCGTTCAATTTTATTCCGATCAGGCAATAGTTGAACTTTTTCACCGTGGCTC

860 880 900 920 940 960

TCGGTGCTTTTTTTATTTTTTGTCACTATTGTTATGTAAAATGCCACCTCTGACAGTATGGAACGCAAACTTCTGTCTAGTGGATAGTCGACAAGCTTACCAGTTCT
AGCCACGAAAAAAATAAAAAACAGTGATAACAATACATTTTACGGTGGAGACTGTCATACCTTGCGTTTGAAGACAGATCACCTATCAGCTGTTCGAATGGTCAAGA

GPD ...ter

980 1,000 1,020 1,040 1,060

CACACGGAACACCACTAATGGACACAAATTCGAAATACTTTGACCCTATTTTCGAGGACCTTGTCACCTTGAGCCCAAGAGAGCCAAGATTTAAATTTTCCTATGAC
GTGTGCCTTGTGGTGATTACCTGTGTTTAAGCTTTATGAAACTGGGATAAAAGCTCCTGGAACAGTGGAACTCGGGTTCTCTCGGTTCTAAATTTAAAAGGATACTG

GPD promoter

1,080 1,100 1,120 1,140 1,160

TTGATGCAAATTCCCAAAGCTAATAACATGCAAGACACGTACGGTCAAGAAGACATATTTGACCTCTTAACAGGTTCAGACGCGACTGCCTCATCAGTAAGACCCGT
AACTACGTTTAAGGGTTTCGATTATTGTACGTTCTGTGCATGCCAGTTCTTCTGTATAAACTGGAGAATTGTCCAAGTCTGCGCTGACGGAGTAGTCATTCTGGGCA

GPD promoter

1,180 1,200 1,220 1,240 1,260 1,280

TGAAAAGAACTTACCTGAAAAAAACGAATATATACTAGCGTTGAATGTTAGCGTCAACAACAAGAAGTTTAATGACGCGGAGGCCAAGGCAAAAAGATTCCTTGATT
ACTTTTCTTGAATGGACTTTTTTTGCTTATATATGATCGCAACTTACAATCGCAGTTGTTGTTCTTCAAATTACTGCGCCTCCGGTTCCGTTTTTCTAAGGAACTAA

GPD promoter

1,300 1,320 1,340 1,360 1,380

ACGTAAGGGAGTTAGAATCATTTTGAATAAAAAACACGCTTTTTCAGTTCGAGTTTATCATTATCAATACTGCCATTTCAAAGAATACGTAAATAATTAATAGTAGT
TGCATTCCCTCAATCTTAGTAAAACTTATTTTTTGTGCGAAAAAGTCAAGCTCAAATAGTAATAGTTATGACGGTAAAGTTTCTTATGCATTTATTAATTATCATCA

GPD promoter

1,400 1,420 1,440 1,460 1,480

GATTTTCCTAACTTTATTTAGTCAAAAAATTAGCCTTTTAATTCTGCTGTAACCCGTACATGCCCAAAATAGGGGGCGGGTTACACAGAATATATAACATCGTAGGT
CTAAAAGGATTGAAATAAATCAGTTTTTTAATCGGAAAATTAAGACGACATTGGGCATGTACGGGTTTTATCCCCCGCCCAATGTGTCTTATATATTGTAGCATCCA

GPD promoter

1,500 1,520 1,540 1,560 1,580 1,600

GTCTGGGTGAACAGTTTATTCCTGGCATCCACTAAATATAATGGAGCCCGCTTTTTAAGCTGGCATCCAGAAAAAAAAAGAATCCCAGCACCAAAATATTGTTTTCT
CAGACCCACTTGTCAAATAAGGACCGTAGGTGATTTATATTACCTCGGGCGAAAAATTCGACCGTAGGTCTTTTTTTTTCTTAGGGTCGTGGTTTTATAACAAAAGA

GPD promoter

1,620 1,640 1,660 1,680 1,700

TCACCAACCATCAGTTCATAGGTCCATTCTCTTAGCGCAACTACAGAGAACAGGGGCACAAACAGGCAAAAAACGGGCACAACCTCAATGGAGTGATGCAACCTGCC
AGTGGTTGGTAGTCAAGTATCCAGGTAAGAGAATCGCGTTGATGTCTCTTGTCCCCGTGTTTGTCCGTTTTTTGCCCGTGTTGGAGTTACCTCACTACGTTGGACGG

GPD promoter

1,720 1,740 1,760 1,780 1,800

TGGAGTAAATGATGACACAAGGCAATTGACCCACGCATGTATCTATCTCATTTTCTTACACCTTCTATTACCTTCTGCTCTCTCTGATTTGGAAAAAGCTGAAAAAA
ACCTCATTTACTACTGTGTTCCGTTAACTGGGTGCGTACATAGATAGAGTAAAAGAATGTGGAAGATAATGGAAGACGAGAGAGACTAAACCTTTTTCGACTTTTTT

GPD promoter

1,820 1,840 1,860 1,880 1,900 1,920

AAGGTTGAAACCAGTTCCCTGAAATTATTCCCCTACTTGACTAATAAGTATATAAAGACGGTAGGTATTGATTGTAATTCTGTAAATCTATTTCTTAAACTTCTTAA
TTCCAACTTTGGTCAAGGGACTTTAATAAGGGGATGAACTGATTATTCATATATTTCTGCCATCCATAACTAACATTAAGACATTTAGATAAAGAATTTGAAGAATT

GPD promoter

1,940 1,960 1,980 2,000 2,020

ATTCTACTTTTATAGTTAGTCTTTTTTTTAGTTTTAAAACACCAGAACTTAGTTTCGACGGATTCTAGAACTAGTGGATCCATGAGATTCCCATCTATCTTCACCGC
TAAGATGAAAATATCAATCAGAAAAAAAATCAAAATTTTGTGGTCTTGAATCAAAGCTGCCTAAGATCTTGATCACCTAGGTACTCTAAGGGTAGATAGAAGTGGCG

1 2 3 4 5 6 7 8 9
M R F P S I F T A

GPD promoter | Mating fac...ha leader

2,040 2,060 2,080 2,100 2,120 2,140

TGTTTTGTTCGCTGCTTCTTCTGCTTTGGCTGCTCCAGCTAACACCACCACCGAAGACGAAACCGCTCAAATCCCAGCTGAAGCTGTTATCGACTACTCTGACTTGG
ACAAAACAAGCGACGAAGAAGACGAAACCGACGAGGTCGATTGTGGTGGTGGCTTCTGCTTTGGCGAGTTTAGGGTCGACTTCGACAATAGCTGATGAGACTGAACC

10 11 12 13 14 15 16 17 18 19 20 21 22 23 24 25 26 27 28 29 30 31 32 33 34 35 36 37 38 39 40 41 42 43 44
V L F A A S S A L A A P A N T T T E D E T A Q I P A E A V I D Y S D L

Mating factor alpha leader

2,160 2,180 2,200 2,220 2,240

AAGGTGACTTCGACGCTGCTGCTTTGCCATTGTCTAACTCTACCAACAACGGTTTGTCTTCTACCAACACCACCATCGCTTCTATCGCTGCTAAGGAAGAAGGTGTT
TTCCACTGAAGCTGCGACGACGAAACGGTAACAGATTGAGATGGTTGTTGCCAAACAGAAGATGGTTGTGGTGGTAGCGAAGATAGCGACGATTCCTTCTTCCACAA

45 46 47 48 49 50 51 52 53 54 55 56 57 58 59 60 61 62 63 64 65 66 67 68 69 70 71 72 73 74 75 76 77 78 79 80
E G D F D A A A L P L S N S T N N G L S S T N T T I A S I A A K E E G V

Mating factor alpha leader

2,260 2,280 2,300 2,320 2,340

CAATTGGACAAGAGAGAAGCTAGCGCAGGATCCTACCCATACGATGTTCCAGATTACGCTCAAACATCCTCACCCTCTACAGAAGCATCAGTATCAACCTCTAGTAC
GTTAACCTGTTCTCTCTTCGATCGCGTCCTAGGATGGGTATGCTACAAGGTCTAATGCGAGTTTGTAGGAGTGGGAGATGTCTTCGTAGTCATAGTTGGAGATCATG

81 82 83 84 85 86
Q L D K R E

1 2 1 2 3 4 5 6 7 8 9 2 4 6 8 10 12 14 16
G S Y P Y D V P D Y A Q T S S P S T E A S V S T S S T

Mating...eader | HA tag | 649 stalk

2,360 2,380 2,400 2,420 2,440 2,460

CTCTTCCTCTGCTTCACAATCATCTGACCCAACTACAACATCTTCGTCCAGTTCATCGTCTTCCCCATCTTCCCAATCTGAGGAAATTTCATCATCACCGACGGTCT
GAGAAGGAGACGAAGTGTTAGTAGACTGGGTTGATGTTGTAGAAGCAGGTCAAGTAGCAGAAGGGGTAGAAGGGTTAGACTCCTTTAAAGTAGTAGTGGCTGCCAGA

18 20 22 24 26 28 30 32 34 36 38 40 42 44 46 48 50
S S S A S Q S S D P T T T S S S S S S S S P S S Q S E E I S S S P T V

649 stalk

2,480 2,500 2,520 2,540 2,560

CAACTACACCATCAACTTCTTCATCATCTTCCTCAATGACTTCAACCACCACAACAAAGTCAATCTCAACTTCCACTACAAGTTCAGCTCCAGTTACAGATGTGACA
GTTGATGTGGTAGTTGAAGAAGTAGTAGAAGGAGTTACTGAAGTTGGTGGTGTTGTTTCAGTTAGAGTTGAAGGTGATGTTCAAGTCGAGGTCAATGTCTACACTGT
52 54 56 58 60 62 64 66 68 70 72 74 76 78 80 82 84 86
S T T P S T S S S S S S M T S T T T T K S I S T S T T S S A P V T D V T

649 stalk

2,580 2,600 2,620 2,640 2,660

GTTTCCTCATCGCCTAGTAAATCTACCTCTACTTCGACAAGTACAGAAACATCTAAAACACCTACTTCAATGACAGAGTATACATCTAGTACATCGATAATTTCGAC
CAAAGGAGTAGCGGATCATTTAGATGGAGATGAAGCTGTTCATGTCTTTGTAGATTTTGTGGATGAAGTTACTGTCTCATATGTAGATCATGTAGCTATTAAAGCTG
88 90 92 94 96 98 100 102 104 106 108 110 112 114 116 118 120 122
V S S S P S K S T S T S T S T E T S K T P T S M T E Y T S S T S I I S T

649 stalk

2,680 2,700 2,720 2,740 2,760 2,780

TCCAGTTAGTCACTCGCAGACAGGTTTGTCGGCTTCATCAAGTTCATCATCTACAACATCCGGTTCTTCGTCCACTAAATCAGAAAGTTCGACAACATCTGGCTCTT
AGGTCAATCAGTGAGCGTCTGTCCAAACAGCCGAAGTAGTTCAAGTAGTAGATGTTGTAGGCCAAGAAGCAGGTGATTTAGTCTTTCAAGCTGTTGTAGACCGAGAA
124 126 128 130 132 134 136 138 140 142 144 146 148 150 152 154 156 158
P V S H S Q T G L S A S S S S S S T T S G S S S T K S E S S T T S G S

649 stalk

2,800 2,820 2,840 2,860 2,880

CCCAGTCCGTGGAATCAACCTCCAGCCACGCCACTGTTCTTGCTAATTCCGCAGAAATGGTCACAACATCCTCTAGTTCATCCTCAACATCCGAAATGTCATTAACT
GGGTCAGGCACCTTAGTTGGAGGTCGGTGCGGTGACAAGAACGATTAAGGCGTCTTTACCAGTGTTGTAGGAGATCAAGTAGGAGTTGTAGGCTTTACAGTAATTGA
160 162 164 166 168 170 172 174 176 178 180 182 184 186 188 190 192 194
S Q S V E S T S S H A T V L A N S A E M V T T S S S S S S T S E M S L T

649 stalk

2,900 2,920 2,940 2,960 2,980

AGTACTGCTACCAGTGTACCAGTCTCATCTAGTAGCAGTACGACATATTCTACTAGCGCATCTACACAAGCCGTCACTACAACATCTTCTTCCACTGTATCTACAAC
TCATGACGATGGTCACATGGTCAGAGTAGATCATCGTCATGCTGTATAAGATGATCGCGTAGATGTGTTCGGCAGTGATGTTGTAGAAGAAGGTGACATAGATGTTG
196 198 200 202 204 206 208 210 212 214 216 218 220 222 224 226 228 230
S T A T S V P V S S S S S T T Y S T S A S T Q A V T T T S S S T V S T T

649 stalk

3,000 3,020 3,040 3,060 3,080 3,100

TTCTTCTAGTACAACGTTAACAAGCGCATTCACACATTCTTCAACCACATCGTCCGACCAGCCACCCAGCGACACTACAAGTCCATCTACGACACACGAACCTCATG
AAGAAGATCATGTTGCAATTGTTCGCGTAAGTGTGTAAGAAGTTGGTGTAGCAGGCTGGTCGGTGGGTCGCTGTGATGTTCAGGTAGATGCTGTGTGCTTGGAGTAC
232 234 236 238 240 242 244 246 248 250 252 254 256 258 260 262 264
S S S T T L T S A F T H S S T T S S D Q P P S D T T S P S T T H E P H

649 stalk

3,120 3,140 3,160 3,180 3,200

TAACCACTCAGACGTCATCAGAAACATCTTCTTCTAAGTCATCTTCTACTTCTTCTTCAAGTACATCTCAAACCTCTGAGTCTGCAACACCATCCGATTCCGTATCA
ATTGGTGAGTCTGCAGTAGTCTTTGTAGAAGAAGATTCAGTAGAAGATGAAGAAGAAGTTCATGTAGAGTTTGGAGACTCAGACGTTGTGGTAGGCTAAGGCATAGT
266 268 270 272 274 276 278 280 282 284 286 288 290 292 294 296 298 300
V T T Q T S S E T S S S K S S S T S S S S T S Q T S E S A T P S D S V S
649 stalk
3,220 3,240 3,260 3,280 3,300

CCTGGAAGTTCTACATCAACATCTTCTAGTAGCACTTCTACTTCCACTTCTATTTCCAGTGGAGAAACGACAACTTCTTCTTCTTCATCATCTGCCACGACCACTTC
GGACCTTCAAGATGTAGTTGTAGAAGATCATCGTGAAGATGAAGGTGAAGATAAAGGTCACCTCTTTGCTGTTGAAGAAGAAGAAGTAGTAGACGGTGCTGGTGAAG
302 304 306 308 310 312 314 316 318 320 322 324 326 328 330 332 334 336
P G S S T S T S S S S T S T S T S I S S G E T T T S S S S S S A T T T S
649 stalk
3,320 3,340 3,360 3,380 3,400 3,420

TAACAGCGCAACCTTGTCAGTCTCTACCACACAAACTTCGATTGAAGCCAGTTCATCTACTACATCTACATCTAGTTCAACAATTACAACTTCAAGTAGTAGCGCTC
ATTGTCGCGTTGGAACAGTCAGAGATGGTGTGTTTGAAGCTAACTTCGGTCAAGTAGATGATGTAGATGTAGATCAAGTTGTTAATGTTGAAGTTCATCATCGCGAG
338 340 342 344 346 348 350 352 354 356 358 360 362 364 366 368 370 372
N S A T L S V S T T Q T S I E A S S S T T S T S S S T I T T S S S S A
649 stalk
3,440 3,460 3,480 3,500 3,520

ACATATCGTCGAAATCTCAATCTAGTATTACCTATCCCTCTTCCTCGACATCTTCATCTACATCGTCCTCAATTTCTAGCGAATCTGAAAGTTTTGAATCGACATCA
TGTATAGCAGCTTTAGAGTTAGATCATAATGGATAGGGAGAAGGAGCTGTAGAAGTAGATGTAGCAGGAGTTAAAGATCGCTTAGACTTTCAAAACTTAGCTGTAGT
374 376 378 380 382 384 386 388 390 392 394 396 398 400 402 404 406 408
H I S S K S Q S S I T Y P S S S T S S S T S S S I S S E S E S F E S T S
649 stalk
3,540 3,560 3,580 3,600 3,620

GCAGAAGATGCTCCATCAACAGCACCTTCATCAAGTGTCTCTTCTAAGAGTTCTACCTCTACAACATCAAGCACATCGACATCTTCAAGCACTCCATCTCCATCACC
CGTCTTCTACGAGGTAGTTGTCGTGGAAGTAGTTCACAGAGAAGATTCTCAAGATGGAGATGTTGTAGTTCGTGTAGCTGTAGAAGTTCGTGAGGTAGAGGTAGTGG
410 412 414 416 418 420 422 424 426 428 430 432 434 436 438 440 442 444
A E D A P S T A P S S S V S S K S S T S T T S S T S T S S S T P S P S P
649 stalk
3,640 3,660 3,680 3,700 3,720 3,740

ATCTTCCGTGAGTTCTTCCTCCACCAGCTCATTGACAACTTCTGCTGTATCAACACCAGCTACCTCTCATTCTCAAAGTACTGTAGTAACCACCACTACTATTACTA
TAGAAGGCACTCAAGAAGGAGGTGGTCGAGTAACTGTTGAAGACGACATAGTTGTGGTCGATGGAGAGTAAGAGTTTCATGACATCATTGGTGGTGATGATAATGAT
446 448 450 452 454 456 458 460 462 464 466 468 470 472 474 476 478
S S V S S S S T S S L T T S A V S T P A T S H S Q S T V V T T T T I T
649 stalk
3,760 3,780 3,800 3,820 3,840

```
CATCAACAGGTCCAGTGATGTCTACGACAACAGCTTATTCTTCTAGTTCTACTAGCAGCTCGGAATCTTCTGAGGTTCAGTCTGTCATGTCATCTACGCCTAGTTCA
GTAGTTGTCCAGGTCACTACAGATGCTGTTGTCGAATAAGAAGATCAAGATGATCGTCGAGCCTTAGAAGACTCCAAGTCAGACAGTACAGTAGATGCGGATCAAGT
480 482 484 486 488 490 492 494 496 498 500 502 504 506 508 510 512 514
T S T G P V M S T T T A Y S S S S T S S S E S S E V Q S V M S S T P S S
649 stalk
3,860 3,880 3,900 3,920 3,940
```

```
ACATCAACAACAACCAGTTCGGAATCTACTTCATCTAGCTCCACAGCTTCTACCTCACCATCAACCTCGCAAACTTTCGAAACTTCTCCTACTATAGGAGGTGTCCC
TGTAGTTGTTGTTGGTCAAGCCTTAGATGAAGTAGATCGAGGTGTCGAAGATGGAGTGGTAGTTGGAGCGTTTGAAAGCTTTGAAGAGGATGATATCCTCCACAGGG
516 518 520 522 524 526 528 530 532 534 536 538 540 542 544 546 548 550
T S T T T S S E S T S S S S T A S T S P S T S Q T F E T S P T I G G V P
649 stalk
3,960 3,980 4,000 4,020 4,040 4,060
```

```
CTCAACCACTTCATTTGTCTCTACGCCAACAACGAAATTGTCGCACACTACTTCCACTATGACAGCACAGTCCGATAGTAAGTCTACCCACTCCTCAAGCACATCGA
GAGTTGGTGAAGTAAACAGAGATGCGGTTGTTGCTTTAACAGCGTGTGATGAAGGTGATACTGTCGTGTCAGGCTATCATTCAGATGGGTGAGGAGTTCGTGTAGCT
552 554 556 558 560 562 564 566 568 570 572 574 576 578 580 582 584 586
S T T S F V S T P T T K L S H T T S T M T A Q S D S K S T H S S S T S
649 stalk
4,080 4,100 4,120 4,140 4,160
```

```
CAGAAGATAAATCATCCACTGCTTCTGCAGTTGACGAAAGCACTACAACATCCACTTCCACGGAGTCTACTACATCAGTAACATCAGGCACCTCCCATTCCGCTAAA
GTCTTCTATTTAGTAGGTGACGAAGACGTCAACTGCTTTCGTGATGTTGTAGGTGAAGGTGCCTCAGATGATGTAGTCATTGTAGTCCGTGGAGGGTAAGGCGATTT
588 590 592 594 596 598 600 602 604 606 608 610 612 614 616 618 620 622
T E D K S S T A S A V D E S T T T S T S T E S T T S V T S G T S H S A K
649 stalk
4,180 4,200 4,220 4,240 4,260 4,280
```

```
GAATCTTCGTCAAATTCTAAGGTGTATAGTTCACAGACAGCACACTCATCCATAAGTGTTGCATCATCACCTAGTACAAAGGGCGCCCAAATCCAATCTTCTATGGT
CTTAGAAGCAGTTTAAGATTCCACATATCAAGTGTCTGTCGTGTGAGTAGGTATTCACAACGTAGTAGTGGATCATGTTTCCCGCGGGTTTAGGTTAGAAGATACCA
624 626 628 630 632 634 636 638 640 642 644 646 648 650 652 654 656 658
E S S S N S K V Y S S Q T A H S S I S V A S S P S T K G A Q I Q S S M V
649 stalk GPI anchor
4,300 4,320 4,340 4,360 4,380
```

```
TGAAATCTCTACCTACGCTGGTTCTGCTAACTCTGTTAACGCTGGTGCTGGTGCTGGTGCTTTGTTCTTGTTGTTGTCTTTGGCTATCATCTAATGATTAATTAACT
ACTTTAGAGATGGATGCGACCAAGACGATTGAGACAATTGCGACCACGACCACGACCACGAAACAAGAACAACAACAGAAACCGATAGTAGATTACTAATTAATTGA
660 662 664 666 668 670 672 674 676 678 680 682 684 686 688 1
E I S T Y A G S A N S V N A G A G A G A L F L L L S L A I I *
GPI anchor
4,400 4,420 4,440 4,460 4,480
```

CGAGATCTGATAACAACAGTGTAGATGTAACAAAATCGACTTTGTTCCCACTGTACTTTTAGCTCGTACAAAATACAATATACTTTTCATTTCTCCGTAAACAACAT
GCTCTAGACTATTGTTGTCACATCTACATTGTTTTAGCTGAAACAAGGGTGACATGAAAATCGAGCATGTTTTATGTTATATGAAAAGTAAAGAGGCATTTGTTGTA

alpha factor terminator

4,500 4,520 4,540 4,560 4,580 4,600

GTTTTCCCATGTAATATCCTTTTCTATTTTTCGTTCCGTTACCAACTTTACACATACTTTATATAGCTATTCACTTCTATACACTAAAAAACTAAGACAATTTTAAT
CAAAAGGGTACATTATAGGAAAAGATAAAAAGCAAGGCAATGGTTGAAATGTGTATGAAATATATCGATAAGTGAAGATATGTGATTTTTTGATTCTGTTAAAATTA

alpha factor terminator

4,620 4,640 4,660 4,680 4,700

TTTGCTGCCTGCCATATTTCAATTTGTTATAAATTCCTATAATTTATCCTATTAGTAGCTAAAAAAAGATGAATGTGAATCGAATCCTAAGAGAATTGGCCGGTACC
AAACGACGGACGGTATAAAGTTAAACAATATTTAAGGATATTAAATAGGATAATCATCGATTTTTTTCTACTTACACTTAGCTTAGGATTCTCTTAACCGGCCATGG

alpha factor terminator

4,720 4,740 4,760 4,780 4,800

CAATTCGCCCTATAGTGAGTCGTATTACGCGCGCTCACTGGCCGTCGTTTTACAACGTCGTGACTGGGAAAACCCTGGCGTTACCCAACTTAATCGCCTTGCAGCAC
GTTAAGCGGGATATCACTCAGCATAATGCGCGCGAGTGACCGGCAGCAAAATGTTGCAGCACTGACCCTTTTGGGACCGCAATGGGTTGAATTAGCGGAACGTCGTG

T7 M13-fwd

4,820 4,840 4,860 4,880 4,900 4,920

ATCCCCCTTTCGCCAGCTGGCGTAATAGCGAAGAGGCCCGCACCGATCGCCCTTCCCAACAGTTGCGCAGCCTGAATGGCGAATGGACGCGCCCTGTAGCGGCGCAT
TAGGGGGAAAGCGGTCGACCGCATTATCGCTTCTCCGGGCGTGGCTAGCGGGAAGGGTTGTCAACGCGTCGGACTTACCGCTTACCTGCGCGGGACATCGCCGCGTA

LacZ alpha

4,940 4,960 4,980 5,000 5,020

TAAGCGCGGCGGGTGTGGTGGTTACGCGCAGCGTGACCACTACACTTGCCAGCGCCCTAGCGCCCGCTCCTTTCGCTTTCTTCCCTTCCTTTCTCGCCACGTTCGCC
ATTCGCGCCGCCCACACCACCAATGCGCGTCGCACTGGTGATGTGAACGGTCGCGGGATCGCGGGCGAGGAAAGCGAAAGAAGGGAAGGAAAGAGCGGTGCAAGCGG

5,040 5,060 5,080 5,100 5,120

GGCTTTCCCCGTCAAGCTCTAAATCGGGGGCTCCCTTTAGGGTTCCGATTTAGTGCTTTACGGCACCTCGACCCCAAAAAACTTGATTAGGGTGATGGTTCACGTAG
CCGAAAGGGGCAGTTCGAGATTTAGCCCCCGAGGGAAATCCCAAGGCTAAATCACGAAATGCCGTGGAGCTGGGGTTTTTTGAACTAATCCCACTACCAAGTGCATC

5,140 5,160 5,180 5,200 5,220 5,240

TGGGCCATCGCCCTGATAGACGGTTTTTCGCCCTTTGACGTTGGAGTCCACGTTCTTTAATAGTGGACTCTTGTTCCAAACTGGAACAACACTCAACCCTATCTCGG
ACCCGGTAGCGGGACTATCTGCCAAAAAGCGGGAAACTGCAACCTCAGGTGCAAGAAATTATCACCTGAGAACAAGGTTTGACCTTGTTGTGAGTTGGGATAGAGCC

5,260 5,280 5,300 5,320 5,340

TCTATTCTTTTGATTTATAAGGGATTTTGCCGATTTCGGCCTATTGGTTAAAAAATGAGCTGATTTAACAAAAATTTAACGCGAATTTTAACAAAATATTAACGCTT
AGATAAGAAAACTAAATATTCCCTAAAACGGCTAAAGCCGGATAACCAATTTTTTACTCGACTAAATTGTTTTTAAATTGCGCTTAAAATTGTTTTATAATTGCGAA

5,360 5,380 5,400 5,420 5,440

ACAATTTCCTGATGCGGTATTTTCTCCTTACGCATCTGTGCGGTATTTCACACCGCATAGGGTAATAACTGATATAATTAAATTGAAGCTCTAATTTGTGAGTTTAG
TGTTAAAGGACTACGCCATAAAAGAGGAATGCGTAGACACGCCATAAAGTGTGGCGTATCCCATTATTGACTATATTAATTTAACTTCGAGATTAAACACTCAAATC

5,460 5,480 5,500 5,520 5,540 5,560

TATACATGCATTTACTTATAATACAGTTTTTTAAGCAAGGATTTTCTTAACTTCTTCGGCGACAGCATCACCGACTTCGGTGGTACTGTTGGAACCACCTAAATCAC
ATATGTACGTAAATGAATATTATGTCAAAAAATTCGTTCCTAAAAGAATTGAAGAAGCCGCTGTCGTAGTGGCTGAAGCCACCATGACAACCTTGGTGGATTTAGTG
LEU2

5,580 5,600 5,620 5,640 5,660

CAGTTCTGATACCTGCATCCAAAACCTTTTTAACTGCATCTTCAATGGCCTTACCTTCTTCAGGCAAGTTCAATGACAATTTCAACATCATTGCAGCAGACAAGATA
GTCAAGACTATGGACGTAGGTTTTGGAAAAATTGACGTAGAAGTTACCGGAATGGAAGAAGTCCGTTCAAGTTACTGTTAAAGTTGTAGTAACGTCGTCTGTTCTAT
LEU2

5,680 5,700 5,720 5,740 5,760

GTGGCGATAGGGTTGACCTTATTCTTTGGCAAATCTGGAGCAGAACCGTGGCATGGTTCGTACAAACCAAATGCGGTGTTCTTGTCTGGCAAAGAGGCCAAGGACGC
CACCGCTATCCCAACTGGAATAAGAAACCGTTTAGACCTCGTCTTGGCACCGTACCAAGCATGTTTGGTTTACGCCACAAGAACAGACCGTTTCTCCGGTTCCTGCG
LEU2

5,780 5,800 5,820 5,840 5,860 5,880

AGATGGCAACAAACCCAAGGAACCTGGGATAACGGAGGCTTCATCGGAGATGATATCACCAAACATGTTGCTGGTGATTATAATACCATTTAGGTGGGTTGGGTTCT
TCTACCGTTGTTTGGGTTCCTTGGACCCTATTGCCTCCGAAGTAGCCTCTACTATAGTGGTTTGTACAACGACCACTAATATTATGGTAAATCCACCCAACCCAAGA
LEU2

5,900 5,920 5,940 5,960 5,980

TAACTAGGATCATGGCGGCAGAATCAATCAATTGATGTTGAACCTTCAATGTAGGGAATTCGTTCTTGATGGTTTCCTCCACAGTTTTTCTCCATAATCTTGAAGAG
ATTGATCCTAGTACCGCCGTCTTAGTTAGTTAACTACAACTTGGAAGTTACATCCCTTAAGCAAGAACTACCAAAGGAGGTGTCAAAAAGAGGTATTAGAACTTCTC
LEU2

6,000 6,020 6,040 6,060 6,080

GCCAAAACATTAGCTTTATCCAAGGACCAAATAGGCAATGGTGGCTCATGTTGTAGGGCCATGAAAGCGGCCATTCTTGTGATTCTTTGCACTTCTGGAACGGTGTA
CGGTTTTGTAATCGAAATAGGTTCCTGGTTTATCCGTTACCACCGAGTACAACATCCCGGTACTTTCGCCGGTAAGAACACTAAGAAACGTGAAGACCTTGCCACAT
LEU2

6,100 6,120 6,140 6,160 6,180 6,200

TTGTTCACTATCCCAAGCGACACCATCACCATCGTCTTCCTTTCTCTTACCAAAGTAAATACCTCCCACTAATTCTCTGACAACAACGAAGTCAGTACCTTTAGCAA
AACAAGTGATAGGGTTCGCTGTGGTAGTGGTAGCAGAAGGAAAGAGAATGGTTTCATTTATGGAGGGTGATTAAGAGACTGTTGTTGCTTCAGTCATGGAAATCGTT
LEU2

6,220 6,240 6,260 6,280 6,300

ATTGTGGCTTGATTGGAGATAAGTCTAAAAGAGAGTCGGATGCAAAGTTACATGGTCTTAAGTTGGCGTACAATTGAAGTTCTTTACGGATTTTTAGTAAACCTTGT
TAACACCGAACTAACCTCTATTCAGATTTTCTCTCAGCCTACGTTTCAATGTACCAGAATTCAACCGCATGTTAACTTCAAGAAATGCCTAAAAATCATTTGGAACA
LEU2

6,320 6,340 6,360 6,380 6,400 6,420

TCAGGTCTAACACTACCGGTACCCCATTTAGGACCACCCACAGCACCTAACAAAACGGCATCAGCCTTCTTGGAGGCTTCCAGCGCCTCATCTGGAAGTGGAACACC
AGTCCAGATTGTGATGGCCATGGGGTAAATCCTGGTGGGTGTCGTGGATTGTTTTGCCGTAGTCGGAAGAACCTCCGAAGGTCGCGGAGTAGACCTTCACCTTGTGG
LEU2
6,440 6,460 6,480 6,500 6,520

TGTAGCATCGATAGCAGCACCACCAATTAAATGATTTTCGAAATCGAACTTGACATTGGAACGAACATCAGAAATAGCTTTAAGAACCTTAATGGCTTCGGCTGTGA
ACATCGTAGCTATCGTCGTGGTGGTTAATTTACTAAAAGCTTTAGCTTGAACTGTAACCTTGCTTGTAGTCTTTATCGAAATTCTTGGAATTACCGAAGCCGACACT
LEU2
6,540 6,560 6,580 6,600 6,620

TTTCTTGACCAACGTGGTCACCTGGCAAAACGACGATCTTCTTAGGGGCAGACATTAGAATGGTATATCCTTGAAATATATATATATATATATNTNGCTGAAATGTAAA
AAAGAACTGGTTGCACCAGTGGACCGTTTTGCTGCTAGAAGAATCCCCGTCTGTAATCTTACCATATAGGAACTTTATATATATATATATATANANCGACTTTACATTT
LEU2
6,640 6,660 6,680 6,700 6,720 6,740

AGGTAAGAAAAGTTAGAAAGTAAGACGATTGCTAACCACCTATTGGAAAAAACAATAGGTCCTTAAATAATATTGTCAACTTCAAGTATTGTGATGCAAGCATTTAG
TCCATTCTTTTCAATCTTTCATTCTGCTAACGATTGGTGGATAACCTTTTTTGTTATCCAGGAATTTATTATAACAGTTGAAGTTCATAACACTACGTTCGTAAATC
6,760 6,780 6,800 6,820 6,840

TCATGAACGCTTCTCTATTCTATATGAAAAGCCGGTTCCGGCGCTCTCACCTTTCCTTTTTCTCCCAATTTTTCAGTTGAAAAAGGTATATGCGTCAGGCGACCTCT
AGTACTTGCGAAGAGATAAGATATACTTTTCGGCCAAGGCCGCGAGAGTGGAAAGGAAAAAGAGGGTTAAAAAGTCAACTTTTTCCATATACGCAGTCCGCTGGAGA
6,860 6,880 6,900 6,920 6,940

GAAATTAACAAAAAATTTCCAGTCATCGAATTTGATTCTGTGCGATAGCGCCCCTGTGTGTTCTCGTTATGTTGAGGAAAAAAATAATGGTTGCTAAGAGATTCGAA
CTTTAATTGTTTTTTAAAGGTCAGTAGCTTAAACTAAGACACGCTATCGCGGGGACACACAAGAGCAATACAACTCCTTTTTTTATTACCAACGATTCTCTAAGCTT
6,960 6,980 7,000 7,020 7,040 7,060

CTCTTGCATCTTACGATACCTGAGTATTCCCACAGTTTGAAAAGCTGTGGTATGGTGCACTCTCAGTACAATCTGCTCTGATGCCGCATAGTTAAGCCAGCCCCGAC
GAGAACGTAGAATGCTATGGACTCATAAGGGTGTCAAACTTTTCGACACCATACCACGTGAGAGTCATGTTAGACGAGACTACGGCGTATCAATTCGGTCGGGGCTG
7,080 7,100 7,120 7,140 7,160

ACCCGCCAACACCCGCTGACGCGCCCTGACGGGCTTGTCTGCTCCCGGCATCCGCTTACAGACAAGCTGTGACCGTCTCCGGGAGCTGCATGTGTCAGAGGTTTTCA
TGGGCGGTTGTGGGCGACTGCGCGGGACTGCCCGAACAGACGAGGGCCGTAGGCGAATGTCTGTTCGACACTGGCAGAGGCCCTCGACGTACACAGTCTCCAAAAGT
7,180 7,200 7,220 7,240 7,260

CCGTCATCACCGAAACGCGCGAGACGAAAGGGCCTCGTGATACGCCTATTTTTATAGGTTAATGTCATGATAATAATGGTTTCTTAGTATGATCCAATATCAAAGGA
GGCAGTAGTGGCTTTGCGCGCTCTGCTTTCCCGGAGCACTATGCGGATAAAAATATCCAATTACAGTACTATTATTACCAAAGAATCATACTAGGTTATAGTTTCCT
2 micr...rigin
7,280 7,300 7,320 7,340 7,360 7,380

AATGATAGCATTGAAGGATGAGACTAATCCAATTGAGGAGTGGCAGCATATAGAACAGCTAAAGGGTAGTGCTGAAGGAAGCATACGATACCCCGCATGGAATGGGA
TTACTATCGTAACTTCCTACTCTGATTAGGTTAACTCCTCACCGTCGTATATCTTGTCGATTTCCCATCACGACTTCCTTCGTATGCTATGGGGCGTACCTTACCCT

2 micron origin

7,400 7,420 7,440 7,460 7,480

TAATATCACAGGAGGTACTAGACTACCTTTCATCCTACATAAATAGACGCATATAAGTACGCATTTAAGCATAAACACGCACTATGCCGTTCTTCTCATGTATATAT
ATTATAGTGTCCTCCATGATCTGATGGAAAGTAGGATGTATTTATCTGCGTATATTCATGCGTAAATTCGTATTTGTGCGTGATACGGCAAGAAGAGTACATATATA

2 micron origin

7,500 7,520 7,540 7,560 7,580

ATATACAGGCAACACGCAGATATAGGTGCGACGTGAACAGTGAGCTGTATGTGCGCAGCTCGCGTTGCATTTTCGGAAGCGCTCGTTTTCGGAAACGCTTTGAAGTT
TATATGTCCGTTGTGCGTCTATATCCACGCTGCACTTGTCACTCGACATACACGCGTCGAGCGCAACGTAAAAGCCTTCGCGAGCAAAAGCCTTTGCGAAACTTCAA

2 micron origin

7,600 7,620 7,640 7,660 7,680 7,700

CCTATTCCGAAGTTCCTATTCTCTAGAAAGTATAGGAACTTCAGAGCGCTTTTGAAAACCAAAAGCGCTCTGAAGACGCACTTTCAAAAAACCAAAAACGCACCGGA
GGATAAGGCTTCAAGGATAAGAGATCTTTCATATCCTTGAAGTCTCGCGAAAACTTTTGGTTTTCGCGAGACTTCTGCGTGAAAGTTTTTTGGTTTTTGCGTGGCCT

FRT

2 micron origin

7,720 7,740 7,760 7,780 7,800

CTGTAACGAGCTACTAAAATATTGCGAATACCGCTTCCACAAACATTGCTCAAAAGTATCTCTTTGCTATATATCTCTGTGCTATATCCCTATATAACCTACCCATC
GACATTGCTCGATGATTTTATAACGCTTATGGCGAAGGTGTTTGTAACGAGTTTTCATAGAGAAACGATATATAGAGACACGATATAGGGATATATTGGATGGGTAG

2 micron origin

7,820 7,840 7,860 7,880 7,900

CACCTTTCGCTCCTTGAACTTGCATCTAAACTCGACCTCTACATTTTTTATGTTTATCTCTAGTATTACTCTTTAGACAAAAAAATTGTAGTAAGAACTATTCATAG
GTGGAAAGCGAGGAACTTGAACGTAGATTTGAGCTGGAGATGTAAAAAATACAAATAGAGATCATAATGAGAAATCTGTTTTTTTAACATCATTCTTGATAAGTATC

2 micron origin

7,920 7,940 7,960 7,980 8,000 8,020

AGTGAATCGAAAACAATACGAAAATGTAAACATTTCCTATACGTAGTATATAGAGACAAAATAGAAGAAACCGTTCATAATTTTCTGACCAATGAAGAATCATCAAC
TCACTTAGCTTTTGTTATGCTTTTACATTTGTAAAGGATATGCATCATATATCTCTGTTTTATCTTCTTTGGCAAGTATTAAAAGACTGGTTACTTCTTAGTAGTTG

2 micron origin

8,040 8,060 8,080 8,100 8,120

GCTATCACTTTCTGTTCACAAAGTATGCGCAATCCACATCGGTATAGAATATAATCGGGGATGCCTTTATCTTGAAAAAATGCACCCGCAGCTTCGCTAGTAATCAG
CGATAGTGAAAGACAAGTGTTTCATACGCGTTAGGTGTAGCCATATCTTATATTAGCCCCTACGGAAATAGAACTTTTTTACGTGGGCGTCGAAGCGATCATTAGTC

2 micron origin

8,140 8,160 8,180 8,200 8,220

TAAACGCGGGAAGTGGAGTCAGGCTTTTTTTATGGAAGAGAAAATAGACACCAAAGTAGCCTTCTTCTAACCTTAACGGACCTACAGTGCAAAAAGTTATCAAGAGA
ATTTGCGCCCTTCACCTCAGTCCGAAAAAAATACCTTCTCTTTTATCTGTGGTTTCATCGGAAGAAGATTGGAATTGCCTGGATGTCACGTTTTTCAATAGTTCTCT

2 micron origin

8,240 8,260 8,280 8,300 8,320 8,340

CTGCATTATAGAGCGCACAAAGGAGAAAAAAAGTAATCTAAGATGCTTTGTTAGAAAAATAGCGCTCTCGGGATGCATTTTTGTAGAACAAAAAAGAAGTATAGATT
GACGTAATATCTCGCGTGTTTCCTCTTTTTTTCATTAGATTCTACGAAACAATCTTTTTATCGCGAGAGCCCTACGTAAAAACATCTTGTTTTTTCTTCATATCTAA

2 micron origin

8,360 8,380 8,400 8,420 8,440

CTTTGTTGGTAAAATAGCGCTCTCGCGTTGCATTTCTGTTCTGTAAAAATGCAGCTCAGATTCTTTGTTTGAAAAATTAGCGCTCTCGCGTTGCATTTTTGTTTTAC
GAAACAACCATTTTATCGCGAGAGCGCAACGTAAAGACAAGACATTTTTACGTCGAGTCTAAGAAACAAACTTTTTAATCGCGAGAGCGCAACGTAAAAACAAAATG

2 micron origin

8,460 8,480 8,500 8,520 8,540 8,560

AAAAATGAAGCACAGATTCTTCGTTGGTAAAATAGCGCTTTCGCGTTGCATTTCTGTTCTGTAAAAATGCAGCTCAGATTCTTTGTTTGAAAAATTAGCGCTCTCGC
TTTTTACTTCGTGTCTAAGAAGCAACCATTTTATCGCGAAAGCGCAACGTAAAGACAAGACATTTTTACGTCGAGTCTAAGAAACAAACTTTTTAATCGCGAGAGCG

2 micron origin

8,580 8,600 8,620 8,640 8,660

GTTGCATTTTTGTTCTACAAAATGAAGCACAGATGCTTCGTTCAGGTGGCACTTTTCGGGGAAATGTGCGCGGAACCCCTATTTGTTTATTTTTCTAAATACATTCA
CAACGTAAAAACAAGATGTTTTACTTCGTGTCTACGAAGCAAGTCCACCGTGAAAAGCCCCTTTACACGCGCCTTGGGGATAAACAAATAAAAAGATTTATGTAAGT

2 micron origin

8,680 8,700 8,720 8,740 8,760

AATATGTATCCGCTCATGAGACAATAACCCTGATAAATGCTTCAATAATATTGAAAAAGGAAGAGTATGAGTATTCAACATTTCCGTGTCGCCCTTATTCCCTTTTT
TTATACATAGGCGAGTACTCTGTTATTGGGACTATTTACGAAGTTATTATAACTTTTTCCTTCTCATACTCATAAGTTGTAAAGGCACAGCGGGAATAAGGGAAAAA

8,780 8,800 8,820 8,840 8,860 8,880

TGCGGCATTTTGCCTTCCTGTTTTTGCTCACCCAGAAACGCTGGTGAAAGTAAAAGATGCTGAAGATCAGTTGGGTGCACGAGTGGGTTACATCGAACTGGATCTCA
ACGCCGTAAAACGGAAGGACAAAAACGAGTGGGTCTTTGCGACCACTTTCATTTTCTACGACTTCTAGTCAACCCACGTGCTCACCCAATGTAGCTTGACCTAGAGT

8,900 8,920 8,940 8,960 8,980

ACAGCGGTAAGATCCTTGAGAGTTTTCGCCCCGAAGAACGTTTTCCAATGATGAGCACTTTTAAAGTTCTGCTATGTGGCGCGGTATTATCCCGTATTGACGCCGGG
TGTCGCCATTCTAGGAACTCTCAAAAGCGGGGCTTCTTGCAAAAGGTTACTACTCGTGAAAATTTCAAGACGATACACCGCGCCATAATAGGGCATAACTGCGGCCC

AmpR

9,000 9,020 9,040 9,060 9,080

CAAGAGCAACTCGGTCGCCGCATACACTATTCTCAGAATGACTTGGTTGAGTACTCACCAGTCACAGAAAAGCATCTTACGGATGGCATGACAGTAAGAGAATTATG
GTTCTCGTTGAGCCAGCGGCGTATGTGATAAGAGTCTTACTGAACCAACTCATGAGTGGTCAGTGTCTTTTCGTAGAATGCCTACCGTACTGTCATTCTCTTAATAC

AmpR

9,100 9,120 9,140 9,160 9,180 9,200

CAGTGCTGCCATAACCATGAGTGATAACACTGCGGCCAACTTACTTCTGACAACGATCGGAGGACCGAAGGAGCTAACCGCTTTTTTGCACAACATGGGGGATCATG
GTCACGACGGTATTGGTACTCACTATTGTGACGCCGGTTGAATGAAGACTGTTGCTAGCCTCCTGGCTTCCTCGATTGGCGAAAAAACGTGTTGTACCCCCTAGTAC

AmpR

9,220 9,240 9,260 9,280 9,300

TAACTCGCCTTGATCGTTGGGAACCGGAGCTGAATGAAGCCATACCAAACGACGAGCGTGACACCACGATGCCTGTAGCAATGGCAACAACGTTGCGCAAACTATTA
ATTGAGCGGAACTAGCAACCCTTGGCCTCGACTTACTTCGGTATGGTTTGCTGCTCGCACTGTGGTGCTACGGACATCGTTACCGTTGTTGCAACGCGTTTGATAAT

AmpR

9,320 9,340 9,360 9,380 9,400

ACTGGCGAACTACTTACTCTAGCTTCCCGGCAACAATTAATAGACTGGATGGAGGCGGATAAAGTTGCAGGACCACTTCTGCGCTCGGCCCTTCCGGCTGGCTGGTT
TGACCGCTTGATGAATGAGATCGAAGGGCCGTTGTTAATTATCTGACCTACCTCCGCCTATTTCAACGTCCTGGTGAAGACGCGAGCCGGGAAGGCCGACCGACCAA

AmpR

9,420 9,440 9,460 9,480 9,500 9,520

TATTGCTGATAAATCTGGAGCCGGTGAGCGTGGGTCTCGCGGTATCATTGCAGCACTGGGGCCAGATGGTAAGCCCTCCCGTATCGTAGTTATCTACACGACGGGGA
ATAACGACTATTTAGACCTCGGCCACTCGCACCCAGAGCGCCATAGTAACGTCGTGACCCCGGTCTACCATTCGGGAGGGCATAGCATCAATAGATGTGCTGCCCCT

AmpR

9,540 9,560 9,580 9,600 9,620

GTCAGGCAACTATGGATGAACGAAATAGACAGATCGCTGAGATAGGTGCCTCACTGATTAAGCATTGGTAACTGTCAGACCAAGTTTACTCATATATACTTTAGATT
CAGTCCGTTGATACCTACTTGCTTTATCTGTCTAGCGACTCTATCCACGGAGTGACTAATTCGTAACCATTGACAGTCTGGTTCAAATGAGTATATATGAAATCTAA

AmpR

9,640 9,660 9,680 9,700 9,720

GATTTAAAACTTCATTTTTAATTTAAAAGGATCTAGGTGAAGATCCTTTTTGATAATCTCATGACCAAAATCCCTTAACGTGAGTTTTCGTTCCACTGAGCGTCAGA
CTAAATTTTGAAGTAAAAATTAAATTTTCCTAGATCCACTTCTAGGAAAAACTATTAGAGTACTGGTTTTAGGGAATTGCACTCAAAAGCAAGGTGACTCGCAGTCT

9,740 9,760 9,780 9,800 9,820 9,840

CCCCGTAGAAAAGATCAAAGGATCTTCTTGAGATCCTTTTTTTCTGCGCGTAATCTGCTGCTTGCAAACAAAAAAACCACCGCTACCAGCGGTGGTTTGTTTGCCGG
GGGGCATCTTTTCTAGTTTCCTAGAAGAACTCTAGGAAAAAAAGACGCGCATTAGACGACGAACGTTTGTTTTTTTGGTGGCGATGGTCGCCACCAAACAAACGGCC

9,860 9,880 9,900 9,920 9,940

ATCAAGAGCTACCAACTCTTTTTCCGAAGGTAACTGGCTTCAGCAGAGCGCAGATACCAAATACTGTTCTTCTAGTGTAGCCGTAGTTAGGCCACCACTTCAAGAAC
TAGTTCTCGATGGTTGAGAAAAAGGCTTCCATTGACCGAAGTCGTCTCGCGTCTATGGTTTATGACAAGAAGATCACATCGGCATCAATCCGGTGGTGAAGTTCTTG

9,960 9,980 10,000 10,020 10,040

TCTGTAGCACCGCCTACATACCTCGCTCTGCTAATCCTGTTACCAGTGGCTGCTGCCAGTGGCGATAAGTCGTGTCTTACCGGGTTGGACTCAAGACGATAGTTACC
AGACATCGTGGCGGATGTATGGAGCGAGACGATTAGGACAATGGTCACCGACGACGGTCACCGCTATTCAGCACAGAATGGCCCAACCTGAGTTCTGCTATCAATGG

10,060 10,080 10,100 10,120 10,140 10,160

```
GGATAAGGCGCAGCGGTCGGGCTGAACGGGGGGTTCGTGCACACAGCCCAGCTTGGAGCGAACGACCT
CCTATTCCGCGTCGCCAGCCCGACTTGCCCCCCAAGCACGTGTGTCGGGTCGAACCTCGCTTGCTGGA
    10,170    10,180    10,190    10,200    10,210    10,220    10,230
```

ST/SC ligation / pAnchor-HA (pULD1) (10496 bp)

GTTAACGAAGCATCTGTGCTTCATTTTGTAGAACAAAAATGCAACGCGAGAGCGCTAATTTTTCAAACAAAGAATCTGAGCTGCATTTTTACAGAACAGAAATGCAA
CAATTGCTTCGTAGACACGAAGTAAAACATCTTGTTTTTACGTTGCGCTCTCGCGATTAAAAAGTTTGTTTCTTAGACTCGACGTAAAAATGTCTTGTCTTTACGTT

2 micron origin

20 40 60 80 100

CGCGAAAGCGCTATTTTACCAACGAAGAATCTGTGCTTCATTTTTGTAAAACAAAAATGCAACGCGAGAGCGCTAATTTTTCAAACAAAGAATCTGAGCTGCATTTT
GCGCTTTCGCGATAAAATGGTTGCTTCTTAGACACGAAGTAAAAACATTTTGTTTTTACGTTGCGCTCTCGCGATTAAAAAGTTTGTTTCTTAGACTCGACGTAAAA

2 micron origin

120 140 160 180 200

TACAGAACAGAAATGCAACGCGAGAGCGCTATTTTACCAACAAAGAATCTATACTTCTTTTTTGTTCTACAAAAATGCATCCCGAGAGCGCTATTTTTCTAACAAAG
ATGTCTTGTCTTTACGTTGCGCTCTCGCGATAAAATGGTTGTTTCTTAGATATGAAGAAAAAACAAGATGTTTTTACGTAGGGCTCTCGCGATAAAAAGATTGTTTC

2 micron origin

220 240 260 280 300 320

CATCTTAGATTACTTTTTTTCTCCTTTGTGCGCTCTATAATGCAGTCTCTTGATAACTTTTTGCACTGTAGGTCCGTTAAGGTTAGAAGAAGGCTACTTTGGTGTCT
GTAGAATCTAATGAAAAAAAGAGGAAACACGCGAGATATTACGTCAGAGAACTATTGAAAAACGTGACATCCAGGCAATTCCAATCTTCTTCCGATGAAACCACAGA

2 micron origin

340 360 380 400 420

ATTTTCTCTTCCATAAAAAAAGCCTGACTCCACTTCCCGCGTTTACTGATTACTAGCGAAGCTGCGGGTGCATTTTTTCAAGATAAAGGCATCCCCGATTATATTCT
TAAAAGAGAAGGTATTTTTTTCGGACTGAGGTGAAGGGCGCAAATGACTAATGATCGCTTCGACGCCCACGTAAAAAAGTTCTATTTCCGTAGGGGCTAATATAAGA

2 micron origin

440 460 480 500 520

ATACCGATGTGGATTGCGCATACTTTGTGAACAGAAAGTGATAGCGTTGATGATTCTTCATTGGTCAGAAAATTATGAACGGTTTCTTCTATTTTGTCTCTATATAC
TATGGCTACACCTAACGCGTATGAAACACTTGTCTTTCACTATCGCAACTACTAAGAAGTAACCAGTCTTTTAATACTTGCCAAAGAAGATAAAACAGAGATATATG

2 micron origin

540 560 580 600 620 640

TACGTATAGGAAATGTTTACATTTTCGTATTGTTTTCGATTCACTCTATGAATAGTTCTTACTACAATTTTTTTGTCTAAAGAGTAATACTAGAGATAAACATAAAA
ATGCATATCCTTTACAAATGTAAAAGCATAACAAAAGCTAAGTGAGATACTTATCAAGAATGATGTTAAAAAAACAGATTTCTCATTATGATCTCTATTTGTATTTT

2 micron origin

660 680 700 720 740

AATGTAGAGGTCGAGTTTAGATGCAAGTTCAAGGAGCGAAAGGTGGATGGGTAGGTTATATAGGGATATAGCACAGAGATATATAGCAAAGAGATACTTTTGAGCAA
TTACATCTCCAGCTCAAATCTACGTTCAAGTTCCTCGCTTTCCACCTACCCATCCAATATATCCCTATATCGTGTCTCTATATATCGTTTCTCTATGAAAACTCGTT

2 micron origin

760 780 800 820 840

TGTTTGTGGAAGCGGTATTCGCAATATTTTAGTAGCTCGTTACAGTCCGGTGCGTTTTTGGTTTTTTGAAAGTGCGTCTTCAGAGCGCTTTTGGTTTTCAAAAGCGC
ACAAACACCTTCGCCATAAGCGTTATAAAATCATCGAGCAATGTCAGGCCACGCAAAAACCAAAAAACTTTCACGCAGAAGTCTCGCGAAAACCAAAAGTTTTCGCG

2 micron origin

860 880 900 920 940 960

TCTGAAGTTCCTATACTTTCTAGCTAGAGAATAGGAACTTCGGAATAGGAACTTCAAAGCGTTTCCGAAAACGAGCGCTTCCGAAAATGCAACGCGAGCTGCGCACA
AGACTTCAAGGATATGAAAGATCGATCTCTTATCCTTGAAGCCTTATCCTTGAAGTTTCGCAAAGGCTTTTGCTCGCGAAGGCTTTTACGTTGCGCTCGACGCGTGT

2 micron origin

980 1,000 1,020 1,040 1,060

TACAGCTCACTGTTCACGTCGCACCTATATCTGCGTGTTGCCTGTATATATATATACATGAGAAGAACGGCATAGTGCGTGTTTATGCTTAAATGCGTACTTATATG
ATGTCGAGTGACAAGTGCAGCGTGGATATAGACGCACAACGGACATATATATATATGTACTCTTCTTGCCGTATCACGCACAAATACGAATTTACGCATGAATATAC

2 micron origin

1,080 1,100 1,120 1,140 1,160

CGTCTATTTATGTAGGATGAAAGGTAGTCTAGTACCTCCTGTGATATTATCCCATTCCATGCGGGGTATCGTATGCTTCCTTCAGCACTACCCTTTAGCTGTTCTAT
GCAGATAAATACATCCTACTTTCCATCAGATCATGGAGGACACTATAATAGGGTAAGGTACGCCCCATAGCATACGAAGGAAGTCGTGATGGGAAATCGACAAGATA

2 micron origin

1,180 1,200 1,220 1,240 1,260 1,280

ATGCTGCCACTCCTCAATTGGATTAGTCTCATCCTTCAATGCTATCATTTCCTTTGATATTGGATCGATCCGATGATAAGCTGTCAAACATGAGAATTGGGTAATAA
TACGACGGTGAGGAGTTAACCTAATCAGAGTAGGAAGTTACGATAGTAAAGGAAACTATAACCTAGCTAGGCTACTATTCGACAGTTTGTACTCTTAACCCATTATT

2 micron origin URA3

1,300 1,320 1,340 1,360 1,380

CTGATATAATTAAATTGAAGCTCTAATTTGTGAGTTTAGTATACATGCATTTACTTATAATACAGTTTTTTAGTTTTGCTGGCCGCATCTTCTCAAATATGCTTCCC
GACTATATTAATTTAACTTCGAGATTAAACACTCAAATCATATGTACGTAAATGAATATTATGTCAAAAAATCAAAACGACCGGCGTAGAAGAGTTTATACGAAGGG

URA3

1,400 1,420 1,440 1,460 1,480

AGCCTGCTTTTCTGTAACGTTCACCCTCTACCTTAGCATCCCTTCCCTTTGCAAATAGTCCTCTTCCAACAATAATAATGTCAGATCCTGTAGAGACCACATCATCC
TCGGACGAAAAGACATTGCAAGTGGGAGATGGAATCGTAGGGAAGGGAAACGTTTATCAGGAGAAGGTTGTTATTATTACAGTCTAGGACATCTCTGGTGTAGTAGG

URA3

1,500 1,520 1,540 1,560 1,580 1,600

ACGGTTCTATACTGTTGACCCAATGCGTCTCCCTTGTCATCTAAACCCACACCGGGTGTCATAATCAACCAATCGTAACCTTCATCTCTTCCACCCATGTCTCTTTG
TGCCAAGATATGACAACTGGGTTACGCAGAGGGAACAGTAGATTTGGGTGTGGCCCACAGTATTAGTTGGTTAGCATTGGAAGTAGAGAAGGTGGGTACAGAGAAAC

URA3

1,620 1,640 1,660 1,680 1,700

AGCAATAAAGCCGATAACAAAATCTTTGTCGCTCTTCGCAATGTCAACAGTACCCTTAGTATATTCTCCAGTAGATAGGGAGCCCTTGCATGACAATTCTGCTAACA
TCGTTATTTCGGCTATTGTTTTAGAAACAGCGAGAAGCGTTACAGTTGTCATGGGAATCATATAAGAGGTCATCTATCCCTCGGGAACGTACTGTTAAGACGATTGT
URA3
1,720 1,740 1,760 1,780 1,800

TCAAAAGGCCTCTAGGTTCCTTTGTTACTTCTTCTGCCGCCTGCTTCAAACCGCTAACAATACCTGGGCCCACCACACCGTGTGCATTCGTAATGTCTGCCCATTCT
AGTTTTCCGGAGATCCAAGGAAACAATGAAGAAGACGGCGGACGAAGTTTGGCGATTGTTATGGACCCGGGTGGTGTGGCACACGTAAGCATTACAGACGGGTAAGA
URA3
1,820 1,840 1,860 1,880 1,900 1,920

GCTATTCTGTATACACCCGCAGAGTACTGCAATTTGACTGTATTACCAATGTCAGCAAATTTTCTGTCTTCGAAGAGTAAAAAATTGTACTTGGCGGATAATGCCTT
CGATAAGACATATGTGGGCGTCTCATGACGTTAAACTGACATAATGGTTACAGTCGTTTAAAAGACAGAAGCTTCTCATTTTTTAACATGAACCGCCTATTACGGAA
URA3
1,940 1,960 1,980 2,000 2,020

TAGCGGCTTAACTGTGCCCTCCATGGAAAAATCAGTCAAGATATCCACATGTGTTTTTAGTAAACAAATTTTGGGACCTAATGCTTCAACTAACTCCAGTAATTCCT
ATCGCCGAATTGACACGGGAGGTACCTTTTTAGTCAGTTCTATAGGTGTACACAAAAATCATTTGTTTAAAACCCTGGATTACGAAGTTGATTGAGGTCATTAAGGA
URA3
2,040 2,060 2,080 2,100 2,120 2,140

TGGTGGTACGAACATCCAATGAAGCACACAAGTTTGTTTGCTTTTCGTGCATGATATTAAATAGCTTGGCAGCAACAGGACTAGGATGAGTAGCAGCACGTTCCTTA
ACCACCATGCTTGTAGGTTACTTCGTGTGTTCAAACAAACGAAAAGCACGTACTATAATTTATCGAACCGTCGTTGTCCTGATCCTACTCATCGTCGTGCAAGGAAT
URA3
2,160 2,180 2,200 2,220 2,240

TATGTAGCTTTCGACATGATTTATCTTCGTTTCCTGCATGTTTTTGTTCTGTGCAGTTGGGTTAAGAATACTGGGCAATTTCATGTTTCTTCAACACTACATATGCG
ATACATCGAAAGCTGTACTAAATAGAAGCAAAGGACGTACAAAAACAAGACACGTCAACCCAATTCTTATGACCCGTTAAAGTACAAAGAAGTTGTGATGTATACGC
URA3
2,260 2,280 2,300 2,320 2,340

TATATATACCAATCTAAGTCTGTGCTCCTTCCTTCGTTCTTCCTTCTGTTCGGAGATTACCGAATCAAAAAAATTTCAAAGAAACCGAAATCAAAAAAAAGAATAAA
ATATATATGGTTAGATTCAGACACGAGGAAGGAAGCAAGAAGGAAGACAAGCCTCTAATGGCTTAGTTTTTTTAAAGTTTCTTTGGCTTTAGTTTTTTTTCTTATTT
URA3
2,360 2,380 2,400 2,420 2,440 2,460

AAAAAAATGATGAATTGAATTGAAAAGCTAATTCTTGAAGACGAAAGGGCCTCGTGATACGCCTATTTTTATAGGTTAATGTCATGATAATAATGGTTTCTTAGACG
TTTTTTTACTACTTAACTTAACTTTTCGATTAAGAACTTCTGCTTTCCCGGAGCACTATGCGGATAAAAATATCCAATTACAGTACTATTATTACCAAAGAATCTGC
URA3
2,480 2,500 2,520 2,540 2,560

TCAGGTGGCACTTTTCGGGGAAATGTGCGCGGAACCCCTATTTGTTTATTTTTCTAAATACATTCAAATATGTATCCGCTCATGAGACAATAACCCTGATAAATGCT
AGTCCACCGTGAAAAGCCCCTTTACACGCGCCTTGGGGATAAACAAATAAAAAGATTTATGTAAGTTTATACATAGGCGAGTACTCTGTTATTGGGACTATTTACGA
2,580 2,600 2,620 2,640 2,660

TCAATAATATTGAAAAAGGAAGAGTATGAGTATTCAACATTTCCGTGTCGCCCTTATTCCCTTTTTTGCGGCATTTTGCCTTCCTGTTTTTGCTCACCCAGAAACGC
AGTTATTATAACTTTTTCCTTCTCATACTCATAAGTTGTAAAGGCACAGCGGGAATAAGGGAAAAAACGCCGTAAAACGGAAGGACAAAAACGAGTGGGTCTTTGCG

2,680 2,700 2,720 2,740 2,760 2,780

TGGTGAAAGTAAAAGATGCTGAAGATCAGTTGGGTGCACGAGTGGGTTACATCGAACTGGATCTCAACAGCGGTAAGATCCTTGAGAGTTTTCGCCCCGAAGAACGT
ACCACTTTCATTTTCTACGACTTCTAGTCAACCCACGTGCTCACCCAATGTAGCTTGACCTAGAGTTGTCGCCATTCTAGGAACTCTCAAAAGCGGGGCTTCTTGCA

2,800 2,820 2,840 2,860 2,880

TTTCCAATGATGAGCACTTTTAAAGTTCTGCTATGTGGCGCGGTATTATCCCGTATTGACGCCGGGCAAGAGCAACTCGGTCGCCGCATACACTATTCTCAGAATGA
AAAGGTTACTACTCGTGAAAATTTCAAGACGATACACCGCGCCATAATAGGGCATAACTGCGGCCCGTTCTCGTTGAGCCAGCGGCGTATGTGATAAGAGTCTTACT

AmpR

2,900 2,920 2,940 2,960 2,980

CTTGGTTGAGTACTCACCAGTCACAGAAAAGCATCTTACGGATGGCATGACAGTAAGAGAATTATGCAGTGCTGCCATAACCATGAGTGATAACACTGCGGCCAACT
GAACCAACTCATGAGTGGTCAGTGTCTTTTCGTAGAATGCCTACCGTACTGTCATTCTCTTAATACGTCACGACGGTATTGGTACTCACTATTGTGACGCCGGTTGA

AmpR

3,000 3,020 3,040 3,060 3,080 3,100

TACTTCTGACAACGATCGGAGGACCGAAGGAGCTAACCGCTTTTTTGCACAACATGGGGGATCATGTAACTCGCCTTGATCGTTGGGAACCGGAGCTGAATGAAGCC
ATGAAGACTGTTGCTAGCCTCCTGGCTTCCTCGATTGGCGAAAAAACGTGTTGTACCCCCTAGTACATTGAGCGGAACTAGCAACCCTTGGCCTCGACTTACTTCGG

AmpR

3,120 3,140 3,160 3,180 3,200

ATACCAAACGACGAGCGTGACACCACGATGCCTGTAGCAATGGCAACAACGTTGCGCAAACTATTAACTGGCGAACTACTTACTCTAGCTTCCCGGCAACAATTAAT
TATGGTTTGCTGCTCGCACTGTGGTGCTACGGACATCGTTACCGTTGTTGCAACGCGTTTGATAATTGACCGCTTGATGAATGAGATCGAAGGGCCGTTGTTAATTA

AmpR

3,220 3,240 3,260 3,280 3,300

AGACTGGATGGAGGCGGATAAAGTTGCAGGACCACTTCTGCGCTCGGCCCTTCCGGCTGGCTGGTTTATTGCTGATAAATCTGGAGCCGGTGAGCGTGGGTCTCGCG
TCTGACCTACCTCCGCCTATTTCAACGTCCTGGTGAAGACGCGAGCCGGGAAGGCCGACCGACCAAATAACGACTATTTAGACCTCGGCCACTCGCACCCAGAGCGC

AmpR

3,320 3,340 3,360 3,380 3,400 3,420

GTATCATTGCAGCACTGGGGCCAGATGGTAAGCCCTCCCGTATCGTAGTTATCTACACGACGGGGAGTCAGGCAACTATGGATGAACGAAATAGACAGATCGCTGAG
CATAGTAACGTCGTGACCCCGGTCTACCATTCGGGAGGGCATAGCATCAATAGATGTGCTGCCCCTCAGTCCGTTGATACCTACTTGCTTTATCTGTCTAGCGACTC

AmpR

3,440 3,460 3,480 3,500 3,520

ATAGGTGCCTCACTGATTAAGCATTGGTAACTGTCAGACCAAGTTTACTCATATATACTTTAGATTGATTTAAAACTTCATTTTTAATTTAAAAGGATCTAGGTGAA
TATCCACGGAGTGACTAATTCGTAACCATTGACAGTCTGGTTCAAATGAGTATATATGAAATCTAACTAAATTTTGAAGTAAAAATTAAATTTTCCTAGATCCACTT

AmpR

3,540 3,560 3,580 3,600 3,620

GATCCTTTTTGATAATCTCATGACCAAAATCCCTTAACGTGAGTTTTCGTTCCACTGAGCGTCAGACCCCGTAGAAAAGATCAAAGGATCTTCTTGAGATCCTTTTT
CTAGGAAAAACTATTAGAGTACTGGTTTTAGGGAATTGCACTCAAAAGCAAGGTGACTCGCAGTCTGGGGCATCTTTTCTAGTTTCCTAGAAGAACTCTAGGAAAAA
ColE1 origin
3,640 3,660 3,680 3,700 3,720 3,740

TTCTGCGCGTAATCTGCTGCTTGCAAACAAAAAAACCACCGCTACCAGCGGTGGTTTGTTTGCCGGATCAAGAGCTACCAACTCTTTTTCCGAAGGTAACTGGCTTC
AAGACGCGCATTAGACGACGAACGTTTGTTTTTTTGGTGGCGATGGTCGCCACCAAACAAACGGCCTAGTTCTCGATGGTTGAGAAAAAGGCTTCCATTGACCGAAG
ColE1 origin
3,760 3,780 3,800 3,820 3,840

AGCAGAGCGCAGATACCAAATACTGTCCTTCTAGTGTAGCCGTAGTTAGGCCACCACTTCAAGAACTCTGTAGCACCGCCTACATACCTCGCTCTGCTAATCCTGTT
TCGTCTCGCGTCTATGGTTTATGACAGGAAGATCACATCGGCATCAATCCGGTGGTGAAGTTCTTGAGACATCGTGGCGGATGTATGGAGCGAGACGATTAGGACAA
ColE1 origin
3,860 3,880 3,900 3,920 3,940

ACCAGTGGCTGCTGCCAGTGGCGATAAGTCGTGTCTTACCGGGTTGGACTCAAGACGATAGTTACCGGATAAGGCGCAGCGGTCGGGCTGAACGGGGGGTTCGTGCA
TGGTCACCGACGACGGTCACCGCTATTCAGCACAGAATGGCCCAACCTGAGTTCTGCTATCAATGGCCTATTCCGCGTCGCCAGCCCGACTTGCCCCCCAAGCACGT
ColE1 origin
3,960 3,980 4,000 4,020 4,040 4,060

CACAGCCCAGCTTGGAGCGAACGACCTACACCGAACTGAGATACCTACAGCGTGAGCTATGAGAAAGCGCCACGCTTCCCGAAGGGAGAAAGGCGGACAGGTATCCG
GTGTCGGGTCGAACCTCGCTTGCTGGATGTGGCTTGACTCTATGGATGTCGCACTCGATACTCTTTCGCGGTGCGAAGGGCTTCCCTCTTTCCGCCTGTCCATAGGC
ColE1 origin
4,080 4,100 4,120 4,140 4,160

GTAAGCGGCAGGGTCGGAACAGGAGAGCGCACGAGGGAGCTTCCAGGGGGAAACGCCTGGTATCTTTATAGTCCTGTCGGGTTTCGCCACCTCTGACTTGAGCGTCG
CATTCGCCGTCCCAGCCTTGTCCTCTCGCGTGCTCCCTCGAAGGTCCCCCTTTGCGGACCATAGAAATATCAGGACAGCCCAAAGCGGTGGAGACTGAACTCGCAGC
ColE1 origin
4,180 4,200 4,220 4,240 4,260 4,280

ATTTTTGTGATGCTCGTCAGGGGGGCGGAGCCTATGGAAAAACGCCAGCAACGCGGCCTTTTTACGGTTCCTGGCCTTTTGCTGGCCTTTTGCTCACATGTTCTTTC
TAAAAACACTACGAGCAGTCCCCCCGCCTCGGATACCTTTTTGCGGTCGTTGCGCCGGAAAAATGCCAAGGACCGGAAAACGACCGGAAAACGAGTGTACAAGAAAG
ColE1 origin
4,300 4,320 4,340 4,360 4,380

CTGCGTTATCCCCTGATTCTGTGGATAACCGTATTACCGCCTTTGAGTGAGCTGATACCGCTCGCCGCAGCCGAACGACCGAGCGCAGCGAGTCAGTGAGCGAGGAA
GACGCAATAGGGGACTAAGACACCTATTGGCATAATGGCGGAAACTCACTCGACTATGGCGAGCGGCGTCGGCTTGCTGGCTCGCGTCGCTCAGTCACTCGCTCCTT
4,400 4,420 4,440 4,460 4,480

GCGGAAGAGCGCCCAATACGCAAACCGCCTCTCCCCGCGCGTTGGCCGATTCATTAATGCAGCTGGCACGACAGGTTTCCCGACTGGAAAGCGGGCAGTGAGCGCAA
CGCCTTCTCGCGGGTTATGCGTTTGGCGGAGAGGGGCGCGCAACCGGCTAAGTAATTACGTCGACCGTGCTGTCCAAAGGGCTGACCTTTCGCCCGTCACTCGCGTT
4,500 4,520 4,540 4,560 4,580 4,600

CGCAATTAATGTGAGTTAGCTCACTCATTAGGCACCCCAGGCTTTACACTTTATGCTTCCGGCTCGTATGTTGTGTGGAATTGTGAGCGGATAACAATTTCACACAG
GCGTTAATTACACTCAATCGAGTGAGTAATCCGTGGGGTCCGAAATGTGAAATACGAAGGCCGAGCATACAACACACCTTAACACTCGCCTATTGTTAAAGTGTGTC

lac promoter LacO

4,620 4,640 4,660 4,680 4,700

GAAACAGCTATGACCATGATTACGCCAAGCTTACCAGTTCTCACACGGAACACCACTAATGGACACAAATTCGAAATACTTTGACCCTATTTTCGAGGACCTTGTCA
CTTTGTCGATACTGGTACTAATGCGGTTCGAATGGTCAAGAGTGTGCCTTGTGGTGATTACCTGTGTTTAAGCTTTATGAAACTGGGATAAAAGCTCCTGGAACAGT

M13-rev GPD promoter

4,720 4,740 4,760 4,780 4,800

CCTTGAGCCCAAGAGAGCCAAGATTTAAATTTTCCTATGACTTGATGCAAATTCCCAAAGCTAATAACATGCAAGACACGTACGGTCAAGAAGACATATTTGACCTC
GGAACTCGGGTTCTCTCGGTTCTAAATTTAAAAGGATACTGAACTACGTTTAAGGGTTTCGATTATTGTACGTTCTGTGCATGCCAGTTCTTCTGTATAAACTGGAG

GPD promoter

4,820 4,840 4,860 4,880 4,900 4,920

TTAACAGGTTCAGACGCGACTGCCTCATCAGTAAGACCCGTTGAAAAGAACTTACCTGAAAAAAACGAATATATACTAGCGTTGAATGTTAGCGTCAACAACAAGAA
AATTGTCCAAGTCTGCGCTGACGGAGTAGTCATTCTGGGCAACTTTTCTTGAATGGACTTTTTTTGCTTATATATGATCGCAACTTACAATCGCAGTTGTTGTTCTT

GPD promoter

4,940 4,960 4,980 5,000 5,020

GTTTAATGACGCGGAGGCCAAGGCAAAAAGATTCCTTGATTACGTAAGGGAGTTAGAATCATTTTGAATAAAAAACACGCTTTTTCAGTTCGAGTTTATCATTATCA
CAAATTACTGCGCCTCCGGTTCCGTTTTTCTAAGGAACTAATGCATTCCCTCAATCTTAGTAAAACTTATTTTTTGTGCGAAAAAGTCAAGCTCAAATAGTAATAGT

GPD promoter

5,040 5,060 5,080 5,100 5,120

ATACTGCCATTTCAAAGAATACGTAAATAATTAATAGTAGTGATTTTCCTAACTTTATTTAGTCAAAAAATTAGCCTTTTAATTCTGCTGTAACCCGTACATGCCCA
TATGACGGTAAAGTTTCTTATGCATTTATTAATTATCATCACTAAAAGGATTGAAATAAATCAGTTTTTTAATCGGAAAATTAAGACGACATTGGGCATGTACGGGT

GPD promoter

5,140 5,160 5,180 5,200 5,220 5,240

AAATAGGGGGCGGGTTACACAGAATATATAACATCGTAGGTGTCTGGGTGAACAGTTTATTCCTGGCATCCACTAAATATAATGGAGCCCGCTTTTTAAGCTGGCAT
TTTATCCCCCGCCCAATGTGTCTTATATATTGTAGCATCCACAGACCCACTTGTCAAATAAGGACCGTAGGTGATTTATATTACCTCGGGCGAAAAATTCGACCGTA

GPD promoter

5,260 5,280 5,300 5,320 5,340

CCAGAAAAAAAAAGAATCCCAGCACCAAAATATTGTTTTCTTCACCAACCATCAGTTCATAGGTCCATTCTCTTAGCGCAACTACAGAGAACAGGGGCACAAACAGG
GGTCTTTTTTTTTCTTAGGGTCGTGGTTTTATAACAAAAGAAGTGGTTGGTAGTCAAGTATCCAGGTAAGAGAATCGCGTTGATGTCTCTTGTCCCCGTGTTTGTCC

GPD promoter

5,360 5,380 5,400 5,420 5,440

```
CAAAAAACGGGCACAACCTCAATGGAGTGATGCAACCTGCCTGGAGTAAATGATGACACAAGGCAATTGACCCACGCATGTATCTATCTCATTTTCTTACACCTTCT
GTTTTTTGCCCGTGTTGGAGTTACCTCACTACGTTGGACGGACCTCATTTACTACTGTGTTCCGTTAACTGGGTGCGTACATAGATAGAGTAAAAGAATGTGGAAGA
GPD promoter
5,460 5,480 5,500 5,520 5,540 5,560
```

```
ATTACCTTCTGCTCTCTCTGATTTGGAAAAAGCTGAAAAAAAAGGTTGAAACCAGTTCCCTGAAATTATTCCCCTACTTGACTAATAAGTATATAAAGACGGTAGGT
TAATGGAAGACGAGAGAGACTAAACCTTTTTCGACTTTTTTTTCCAACTTTGGTCAAGGGACTTTAATAAGGGGATGAACTGATTATTCATATATTTCTGCCATCCA
GPD promoter
5,580 5,600 5,620 5,640 5,660
```

```
ATTGATTGTAATTCTGTAAATCTATTTCTTAAACTTCTTAAATTCTACTTTTATAGTTAGTCTTTTTTTTAGTTTTAAAACACCAAGAACTTAGTTTCGAATAAACA
TAACTAACATTAAGACATTTAGATAAAGAATTTGAAGAATTTAAGATGAAAATATCAATCAGAAAAAAAATCAAAATTTTGTGGTTCTTGAATCAAAGCTTATTTGT
GPD promoter
5,680 5,700 5,720 5,740 5,760
```

```
CACATAAACAGAATTCATGAGATTCCCATCTATCTTCACCGCTGTTTTGTTCGCTGCTTCTTCTGCTTTGGCTGCTCCAGCTAACACCACCACCGAAGACGAAACCG
GTGTATTTGTCTTAAGTACTCTAAGGGTAGATAGAAGTGGCGACAAAACAAGCGACGAAGAAGACGAAACCGACGAGGTCGATTGTGGTGGTGGCTTCTGCTTTGGC
1 2 3 4 5 6 7 8 9 10 11 12 13 14 15 16 17 18 19 20 21 22 23 24 25 26 27 28 29 30
M R F P S I F T A V L F A A S S A L A A P A N T T T E D E T
GPD p...oter   Mating factor alpha leader
5,780 5,800 5,820 5,840 5,860 5,880
```

```
CTCAAATCCCAGCTGAAGCTGTTATCGACTACTCTGACTTGGAAGGTGACTTCGACGCTGCTGCTTTGCCATTGTCTAACTCTACCAACAACGGTTTGTCTTCTACC
GAGTTTAGGGTCGACTTCGACAATAGCTGATGAGACTGAACCTTCCACTGAAGCTGCGACGACGAAACGGTAACAGATTGAGATGGTTGTTGCCAAACAGAAGATGG
31 32 33 34 35 36 37 38 39 40 41 42 43 44 45 46 47 48 49 50 51 52 53 54 55 56 57 58 59 60 61 62 63 64 65 66
A Q I P A E A V I D Y S D L E G D F D A A A L P L S N S T N N G L S S T
Mating factor alpha leader
5,900 5,920 5,940 5,960 5,980
```

```
AACACCACCATCGCTTCTATCGCTGCTAAGGAAGAAGGTGTTCAATTGGACAAGAGAGAAGCTAGCGCAGTAACTACCTTGTCTGGCTTATCAGGGGAACAGGGTCC
TTGTGGTGGTAGCGAAGATAGCGACGATTCCTTCTTCCACAAGTTAACCTGTTCTCTCTTCGATCGCGTCATTGATGGAACAGACCGAATAGTCCCCTTGTCCCAGG
67 68 69 70 71 72 73 74 75 76 77 78 79 80 81 82 83 84 85 86      2   4   6   8   10   12
N T T I A S I A A K E E G V Q L D K R E      V T T L S G L S G E Q G P
Mating factor alpha leader      SpyCatcher
6,000 6,020 6,040 6,060 6,080
```

```
TAGCGGTGACATGACGACAGAGGAGGACAGTGCGACCCACATAAAGTTTAGCAAAAGAGATGAAGATGGAAGGGAACTAGCTGGAGCGACCATGGAACTTCGTGACA
ATCGCCACTGTACTGCTGTCTCCTCCTGTCACGCTGGGTGTATTTCAAATCGTTTTCTCTACTTCTACCTTCCCTTGATCGACCTCGCTGGTACCTTGAAGCACTGT
14 16 18 20 22 24 26 28 30 32 34 36 38 40 42 44 46 48
S G D M T T E E D S A T H I K F S K R D E D G R E L A G A T M E L R D
SpyCatcher
6,100 6,120 6,140 6,160 6,180 6,200
```

GCTCAGGGAAGACTATATCAACGTGGATCTCAGATGGTCACGTCAAGGACTTCTATTTGTACCCCGGAAAGTACACATTTGTGGAAACCGCCGCGCCGGATGGCTAC
CGAGTCCCTTCTGATATAGTTGCACCTAGAGTCTACCAGTGCAGTTCCTGAAGATAAACATGGGGCCTTTCATGTGTAAACACCTTTGGCGGCGCGGCCTACCGATG
50 52 54 56 58 60 62 64 66 68 70 72 74 76 78 80 82 84
S S G K T I S T W I S D G H V K D F Y L Y P G K Y T F V E T A A P D G Y

SpyCatcher

6,220 6,240 6,260 6,280 6,300

GAGGTAGCGACCCCGATAGAATTCACCGTTAACGAGGACGGCCAAGTCACCGTAGATGGAGAGGCGACAGAGGGTGATGCCCACACTGGGGGCTCCGGCGGATCTGG
CTCCATCGCTGGGGCTATCTTAAGTGGCAATTGCTCCTGCCGGTTCAGTGGCATCTACCTCTCCGCTGTCTCCCACTACGGGTGTGACCCCCGAGGCCGCCTAGACC
86 88 90 92 94 96 98 100 102 104 106 108 110 112 1 2 3 4 5 6 7
E V A T P I E F T V N E D G Q V T V D G E A T E G D A H T G G S G G S G

SpyCatcher

6,320 6,340 6,360 6,380 6,400 6,420

TGGGAGCGGATCCTACCCATACGATGTTCCAGATTACGCTCAAACATCCTCACCCTCTACAGAAGCATCAGTATCAACCTCTAGTACCTCTTCCTCTGCTTCACAAT
ACCCTCGCCTAGGATGGGTATGCTACAAGGTCTAATGCGAGTTTGTAGGAGTGGGAGATGTCTTCGTAGTCATAGTTGGAGATCATGGAGAAGGAGACGAAGTGTTA
8 9 10 11 1 2 3 4 5 6 7 8 9 2 4 6 8 10 12 14 16 18 20 22
G S G S Y P Y D V P D Y A Q T S S P S T E A S V S T S S T S S S A S Q

HA tag 649 stalk

6,440 6,460 6,480 6,500 6,520

CATCTGACCCAACTACAACATCTTCGTCCAGTTCATCGTCTTCCCCATCTTCCCAATCTGAGGAAATTTCATCATCACCGACGGTCTCAACTACACCATCAACTTCT
GTAGACTGGGTTGATGTTGTAGAAGCAGGTCAAGTAGCAGAAGGGGTAGAAGGGTTAGACTCCTTTAAAGTAGTAGTGGCTGCCAGAGTTGATGTGGTAGTTGAAGA
24 26 28 30 32 34 36 38 40 42 44 46 48 50 52 54 56 58
S S D P T T T S S S S S S S S P S S Q S E E I S S S P T V S T T P S T S

649 stalk

6,540 6,560 6,580 6,600 6,620

TCATCATCTTCCTCAATGACTTCAACCACCACAACAAAGTCAATCTCAACTTCCACTACAAGTTCAGCTCCAGTTACAGATGTGACAGTTTCCTCATCGCCTAGTAA
AGTAGTAGAAGGAGTTACTGAAGTTGGTGGTGTTGTTTCAGTTAGAGTTGAAGGTGATGTTCAAGTCGAGGTCAATGTCTACACTGTCAAAGGAGTAGCGGATCATT
60 62 64 66 68 70 72 74 76 78 80 82 84 86 88 90 92 94
S S S S S M T S T T T T K S I S T S T T S S A P V T D V T V S S S P S K

649 stalk

6,640 6,660 6,680 6,700 6,720 6,740

ATCTACCTCTACTTCGACAAGTACAGAAACATCTAAAACACCTACTTCAATGACAGAGTATACATCTAGTACATCGATAATTTCGACTCCAGTTAGTCACTCGCAGA
TAGATGGAGATGAAGCTGTTCATGTCTTTGTAGATTTTGTGGATGAAGTTACTGTCTCATATGTAGATCATGTAGCTATTAAAGCTGAGGTCAATCAGTGAGCGTCT
96 98 100 102 104 106 108 110 112 114 116 118 120 122 124 126 128
S T S T S T S T E T S K T P T S M T E Y T S S T S I I S T P V S H S Q

649 stalk

6,760 6,780 6,800 6,820 6,840

CAGGTTTGTCGGCTTCATCAAGTTCATCATCTACAACATCCGGTTCTTCGTCCACTAAATCAGAAAGTTCGACAACATCTGGCTCTTCCCAGTCCGTGGAATCAACC
GTCCAAACAGCCGAAGTAGTTCAAGTAGTAGATGTTGTAGGCCAAGAAGCAGGTGATTTAGTCTTTCAAGCTGTTGTAGACCGAGAAGGGTCAGGCACCTTAGTTGG
130 132 134 136 138 140 142 144 146 148 150 152 154 156 158 160 162 164
T G L S A S S S S S S T T S G S S S T K S E S S T T S G S S Q S V E S T
649 stalk
6,860 6,880 6,900 6,920 6,940

TCCAGCCACGCCACTGTTCTTGCTAATTCCGCAGAAATGGTCACAACATCCTCTAGTTCATCCTCAACATCCGAAATGTCATTAACTAGTACTGCTACCAGTGTACC
AGGTCGGTGCGGTGACAAGAACGATTAAGGCGTCTTTACCAGTGTTGTAGGAGATCAAGTAGGAGTTGTAGGCTTTACAGTAATTGATCATGACGATGGTCACATGG
166 168 170 172 174 176 178 180 182 184 186 188 190 192 194 196 198 200
S S H A T V L A N S A E M V T T S S S S S S T S E M S L T S T A T S V P
649 stalk
6,960 6,980 7,000 7,020 7,040 7,060

AGTCTCATCTAGTAGCAGTACGACATATTCTACTAGCGCATCTACACAAGCCGTCACTACAACATCTTCTTCCACTGTATCTACAACTTCTTCTAGTACAACGTTAA
TCAGAGTAGATCATCGTCATGCTGTATAAGATGATCGCGTAGATGTGTTCGGCAGTGATGTTGTAGAAGAAGGTGACATAGATGTTGAAGAAGATCATGTTGCAATT
202 204 206 208 210 212 214 216 218 220 222 224 226 228 230 232 234 236
V S S S S S T T Y S T S A S T Q A V T T T S S S T V S T T S S S T T L
649 stalk
7,080 7,100 7,120 7,140 7,160

CAAGCGCATTCACACATTCTTCAACCACATCGTCCGACCAGCCACCCAGCGACACTACAAGTCCATCTACGACACACGAACCTCATGTAACCACTCAGACGTCATCA
GTTCGCGTAAGTGTGTAAGAAGTTGGTGTAGCAGGCTGGTCGGTGGGTCGCTGTGATGTTCAGGTAGATGCTGTGTGCTTGGAGTACATTGGTGAGTCTGCAGTAGT
238 240 242 244 246 248 250 252 254 256 258 260 262 264 266 268 270 272
T S A F T H S S T T S S D Q P P S D T T S P S T T H E P H V T T Q T S S
649 stalk
7,180 7,200 7,220 7,240 7,260

GAAACATCTTCTTCTAAGTCATCTTCTACTTCTTCTTCAAGTACATCTCAAACCTCTGAGTCTGCAACACCATCCGATTCCGTATCACCTGGAAGTTCTACATCAAC
CTTTGTAGAAGAAGATTCAGTAGAAGATGAAGAAGAAGTTCATGTAGAGTTTGGAGACTCAGACGTTGTGGTAGGCTAAGGCATAGTGGACCTTCAAGATGTAGTTG
274 276 278 280 282 284 286 288 290 292 294 296 298 300 302 304 306 308
E T S S S K S S S T S S S S T S Q T S E S A T P S D S V S P G S S T S T
649 stalk
7,280 7,300 7,320 7,340 7,360 7,380

ATCTTCTAGTAGCACTTCTACTTCCACTTCTATTTCCAGTGGAGAAACGACAACTTCTTCTTCTTCATCATCTGCCACGACCACTTCTAACAGCGCAACCTTGTCAG
TAGAAGATCATCGTGAAGATGAAGGTGAAGATAAAGGTCACCTCTTTGCTGTTGAAGAAGAAGAAGTAGTAGACGGTGCTGGTGAAGATTGTCGCGTTGGAACAGTC
310 312 314 316 318 320 322 324 326 328 330 332 334 336 338 340 342
S S S S T S T S T S I S S G E T T T S S S S S S A T T T S N S A T L S
649 stalk
7,400 7,420 7,440 7,460 7,480

```
TCTCTACCACACAAACTTCGATTGAAGCCAGTTCATCTACTACATCTACATCTAGTTCAACAATTACAACTTCAAGTAGTAGCGCTCACATATCGTCGAAATCTCAA
AGAGATGGTGTGTTTGAAGCTAACTTCGGTCAAGTAGATGATGTAGATGTAGATCAAGTTGTTAATGTTGAAGTTCATCATCGCGAGTGTATAGCAGCTTTAGAGTT
344 346 348 350 352 354 356 358 360 362 364 366 368 370 372 374 376 378
V S T T Q T S I E A S S S T T S T S S S T I T T S S S S A H I S S K S Q
649 stalk
7,500 7,520 7,540 7,560 7,580

TCTAGTATTACCTATCCCTCTTCCTCGACATCTTCATCTACATCGTCCTCAATTTCTAGCGAATCTGAAAGTTTTGAATCGACATCAGCAGAAGATGCTCCATCAAC
AGATCATAATGGATAGGGAGAAGGAGCTGTAGAAGTAGATGTAGCAGGAGTTAAAGATCGCTTAGACTTTCAAAACTTAGCTGTAGTCGTCTTCTACGAGGTAGTTG
380 382 384 386 388 390 392 394 396 398 400 402 404 406 408 410 412 414
S S I T Y P S S S T S S S T S S S I S S E S E S F E S T S A E D A P S T
649 stalk
7,600 7,620 7,640 7,660 7,680 7,700

AGCACCTTCATCAAGTGTCTCTTCTAAGAGTTCTACCTCTACAACATCAAGCACATCGACATCTTCAAGCACTCCATCTCCATCACCATCTTCCGTGAGTTCTTCCT
TCGTGGAAGTAGTTCACAGAGAAGATTCTCAAGATGGAGATGTTGTAGTTCGTGTAGCTGTAGAAGTTCGTGAGGTAGAGGTAGTGGTAGAAGGCACTCAAGAAGGA
416 418 420 422 424 426 428 430 432 434 436 438 440 442 444 446 448 450
A P S S S V S S K S S T S T T S S T S T S S S T P S P S P S S V S S S
649 stalk
7,720 7,740 7,760 7,780 7,800

CCACCAGCTCATTGACAACTTCTGCTGTATCAACACCAGCTACCTCTCATTCTCAAAGTACTGTAGTAACCACCACTACTATTACTACATCAACAGGTCCAGTGATG
GGTGGTCGAGTAACTGTTGAAGACGACATAGTTGTGGTCGATGGAGAGTAAGAGTTTCATGACATCATTGGTGGTGATGATAATGATGTAGTTGTCCAGGTCACTAC
452 454 456 458 460 462 464 466 468 470 472 474 476 478 480 482 484 486
S T S S L T T S A V S T P A T S H S Q S T V V T T T T I T T S T G P V M
649 stalk
7,820 7,840 7,860 7,880 7,900

TCTACGACAACAGCTTATTCTTCTAGTTCTACTAGCAGCTCGGAATCTTCTGAGGTTCAGTCTGTCATGTCATCTACGCCTAGTTCAACATCAACAACAACCAGTTC
AGATGCTGTTGTCGAATAAGAAGATCAAGATGATCGTCGAGCCTTAGAAGACTCCAAGTCAGACAGTACAGTAGATGCGGATCAAGTTGTAGTTGTTGTTGGTCAAG
488 490 492 494 496 498 500 502 504 506 508 510 512 514 516 518 520 522
S T T T A Y S S S S T S S S E S S E V Q S V M S S T P S S T S T T T S S
649 stalk
7,920 7,940 7,960 7,980 8,000 8,020

GGAATCTACTTCATCTAGCTCCACAGCTTCTACCTCACCATCAACCTCGCAAACTTTCGAAACTTCTCCTACTATAGGAGGTGTCCCCTCAACCACTTCATTTGTCT
CCTTAGATGAAGTAGATCGAGGTGTCGAAGATGGAGTGGTAGTTGGAGCGTTTGAAAGCTTTGAAGAGGATGATATCCTCCACAGGGGAGTTGGTGAAGTAAACAGA
524 526 528 530 532 534 536 538 540 542 544 546 548 550 552 554 556
E S T S S S S T A S T S P S T S Q T F E T S P T I G G V P S T T S F V
649 stalk
8,040 8,060 8,080 8,100 8,120
```

CTACGCCAACAACGAAATTGTCGCACACTACTTCCACTATGACAGCACAGTCCGATAGTAAGTCTACCCACTCCTCAAGCACATCGACAGAAGATAAATCATCCACT
GATGCGGTTGTTGCTTTAACAGCGTGTGATGAAGGTGATACTGTCGTGTCAGGCTATCATTCAGATGGGTGAGGAGTTCGTGTAGCTGTCTTCTATTTAGTAGGTGA
558 560 562 564 566 568 570 572 574 576 578 580 582 584 586 588 590 592
S T P T T K L S H T T S T M T A Q S D S K S T H S S S T S T E D K S S T

649 stalk

8,140 8,160 8,180 8,200 8,220

GCTTCTGCAGTTGACGAAAGCACTACAACATCCACTTCCACGGAGTCTACTACATCAGTAACATCAGGCACCTCCCATTCCGCTAAAGAATCTTCGTCAAATTCTAA
CGAAGACGTCAACTGCTTTCGTGATGTTGTAGGTGAAGGTGCCTCAGATGATGTAGTCATTGTAGTCCGTGGAGGGTAAGGCGATTTCTTAGAAGCAGTTTAAGATT
594 596 598 600 602 604 606 608 610 612 614 616 618 620 622 624 626 628
A S A V D E S T T T S T S T E S T T S V T S G T S H S A K E S S S N S K

649 stalk

8,240 8,260 8,280 8,300 8,320 8,340

GGTGTATAGTTCACAGACAGCACACTCATCCATAAGTGTTGCATCATCACCTAGTACAAAGGGCGCCCAAATCCAATCTTCTATGGTTGAAATCTCTACCTACGCTG
CCACATATCAAGTGTCTGTCGTGTGAGTAGGTATTCACAACGTAGTAGTGGATCATGTTTCCCGCGGGTTTAGGTTAGAAGATACCAACTTTAGAGATGGATGCGAC
630 632 634 636 638 640 642 644 646 648 650 652 654 656 658 660 662 664
V Y S S Q T A H S S I S V A S S P S T K G A Q I Q S S M V E I S T Y A

649 stalk GPI anchor

8,360 8,380 8,400 8,420 8,440

GTTCTGCTAACTCTGTTAACGCTGGTGCTGGTGCTGGTGCTTTGTTCTTGTTGTTGTCTTTGGCTATCATCTAATGATTAATTAACTCGAGATCTGATAACAACAGT
CAAGACGATTGAGACAATTGCGACCACGACCACGACCACGAAACAAGAACAACAACAGAAACCGATAGTAGATTACTAATTAATTGAGCTCTAGACTATTGTTGTCA
666 668 670 672 674 676 678 680 682 684 686 688 1
G S A N S V N A G A G A G A L F L L L S L A I I *

GPI anchor alph...tor

8,460 8,480 8,500 8,520 8,540 8,560

GTAGATGTAACAAAATCGACTTTGTTCCCACTGTACTTTTAGCTCGTACAAAATACAATATACTTTTCATTTCTCCGTAAACAACATGTTTTCCCATGTAATATCCT
CATCTACATTGTTTTAGCTGAAACAAGGGTGACATGAAAATCGAGCATGTTTTATGTTATATGAAAAGTAAAGAGGCATTTGTTGTACAAAAGGGTACATTATAGGA

alpha factor terminator

8,580 8,600 8,620 8,640 8,660

TTTCTATTTTTCGTTCCGTTACCAACTTTACACATACTTTATATAGCTATTCACTTCTATACACTAAAAAACTAAGACAATTTTAATTTTGCTGCCTGCCATATTTC
AAAGATAAAAAGCAAGGCAATGGTTGAAATGTGTATGAAATATATCGATAAGTGAAGATATGTGATTTTTTGATTCTGTTAAAATTAAAACGACGGACGGTATAAAG

alpha factor terminator

8,680 8,700 8,720 8,740 8,760

AATTTGTTATAAATTCCTATAATTTATCCTATTAGTAGCTAAAAAAAGATGAATGTGAATCGAATCCTAAGAGAATTGGTACCGCGATGTAGTAAAACTAGCTAGAC
TTAAACAATATTTAAGGATATTAAATAGGATAATCATCGATTTTTTTCTACTTACACTTAGCTTAGGATTCTCTTAACCATGGCGCTACATCATTTTGATCGATCTG

alpha factor terminator

8,780 8,800 8,820 8,840 8,860 8,880

```
CGAGAAAGAGACTAGAAATGCAAAAGGCACTTCTACAATGGCTGCCATCATTATTATCCGATGTGACGCTGCATTTTTTTTTTTTTTTTTTTTTTTTTTTTTTTTTT
GCTCTTTCTCTGATCTTTACGTTTTCCGTGAAGATGTTACCGACGGTAGTAATAATAGGCTACACTGCGACGTAAAAAAAAAAAAAAAAAAAAAAAAAAAAAAAAAA
                  8,900               8,920               8,940               8,960               8,980

TTTTTTTTTTTTTGTACAAATATCATAAAAAAAGAGAATCTTTTTAAGCAAGGATTTTCTTAACTTCTTCGGCGACAGCATCACCGACTTCGGTGGTACTGTTGGAA
AAAAAAAAAAAAACATGTTTATAGTATTTTTTTCTCTTAGAAAAATTCGTTCCTAAAAGAATTGAAGAAGCCGCTGTCGTAGTGGCTGAAGCCACCATGACAACCTT
           9,000               9,020               9,040               9,060               9,080

CCACCTAAATCACCAGTTCTGATACCTGCATCCAAAACCTTTTTAACTGCATCTTCAATGGCCTTACCTTCTTCAGGCAAGTTCAATGACAATTTCAACATCATTGC
GGTGGATTTAGTGGTCAAGACTATGGACGTAGGTTTTGGAAAAATTGACGTAGAAGTTACCGGAATGGAAGAAGTCCGTTCAAGTTACTGTTAAAGTTGTAGTAACG
    9,100               9,120               9,140               9,160               9,180               9,200

AGCAGACAAGATAGTGGCGATAGGGTCAACCTTATTCTTTGGCAAATCTGGAGCAGAACCGTGGCATGGTTCGTACAAACCAAATGCGGTGTTCTTGTCTGGCAAAG
TCGTCTGTTCTATCACCGCTATCCCAGTTGGAATAAGAAACCGTTTAGACCTCGTCTTGGCACCGTACCAAGCATGTTTGGTTTACGCCACAAGAACAGACCGTTTC
                 9,220               9,240               9,260               9,280               9,300

AGGCCAAGGACGCAGATGGCAACAAACCCAAGGAACCTGGGATAACGGAGGCTTCATCGGAGATGATATCACCAAACATGTTGCTGGTGATTATAATACCATTTAGG
TCCGGTTCCTGCGTCTACCGTTGTTTGGGTTCCTTGGACCCTATTGCCTCCGAAGTAGCCTCTACTATAGTGGTTTGTACAACGACCACTAATATTATGGTAAATCC
        9,320               9,340               9,360               9,380               9,400

TGGGTTGGGTTCTTAACTAGGATCATGGCGGCAGAATCAATCAATTGATGTTGAACCTTCAATGTAGGAAATTCGTTCTTGATGGTTTCCTCCACAGTTTTTCTCCA
ACCCAACCCAAGAATTGATCCTAGTACCGCCGTCTTAGTTAGTTAACTACAACTTGGAAGTTACATCCTTTAAGCAAGAACTACCAAAGGAGGTGTCAAAAAGAGGT
  9,420               9,440               9,460               9,480               9,500               9,520

TAATCTTGAAGAGGCCAAAACATTAGCTTTATCCAAGGACCAAATAGGCAATGGTGGCTCATGTTGTAGGGCCATGAAAGCGGCCATTCTTGTGATTCTTTGCACTT
ATTAGAACTTCTCCGGTTTTGTAATCGAAATAGGTTCCTGGTTTATCCGTTACCACCGAGTACAACATCCCGGTACTTTCGCCGGTAAGAACACTAAGAAACGTGAA
              9,540               9,560               9,580               9,600               9,620

CTGGAACGGTGTATTGTTCACTATCCCAAGCGACACCATCACCATCGTCTTCCTTTCTCTTACCAAAGTAAATACCTCCCACTAATTCTCTGACAACAACGAAGTCA
GACCTTGCCACATAACAAGTGATAGGGTTCGCTGTGGTAGTGGTAGCAGAAGGAAAGAGAATGGTTTCATTTATGGAGGGTGATTAAGAGACTGTTGTTGCTTCAGT
       9,640               9,660               9,680               9,700               9,720

GTACCTTTAGCAAATTGTGGCTTGATTGGAGATAAGTCTAAAAGAGAGTCGGATGCAAAGTTACATGGTCTTAAGTTGGCGTACAATTGAAGTTCTTTACGGATTTT
CATGGAAATCGTTTAACACCGAACTAACCTCTATTCAGATTTTCTCTCAGCCTACGTTTCAATGTACCAGAATTCAACCGCATGTTAACTTCAAGAAATGCCTAAAA
  9,740               9,760               9,780               9,800               9,820               9,840

TAGTAAACCTTGTTCAGGTCTAACACTACCTGTACCCCATTTAGGACCACCCACAGCACCTAACAAAACGGCATCAACCTTCTTGGAGGCTTCCAGCGCCTCATCTG
ATCATTTGGAACAAGTCCAGATTGTGATGGACATGGGGTAAATCCTGGTGGGTGTCGTGGATTGTTTTGCCGTAGTTGGAAGAACCTCCGAAGGTCGCGGAGTAGAC
                9,860               9,880               9,900               9,920               9,940

GAAGTGGGACACCTGTAGCATCGATAGCAGCACCACCAATTAAATGATTTTCGAAATCGAACTTGACATTGGAACGAACATCAGAAATAGCTTTAAGAACCTTAATG
CTTCACCCTGTGGACATCGTAGCTATCGTCGTGGTGGTTAATTTACTAAAAGCTTTAGCTTGAACTGTAACCTTGCTTGTAGTCTTTATCGAAATTCTTGGAATTAC
      9,960               9,980              10,000              10,020              10,040
```

GCTTCGGCTGTGATTTCTTGACCAACGTGGTCACCTGGCAAAACGACGATCTTCTTAGGGGCAGACATTACAATGGTATATCCTTGAAATATATATAAAAAAAAAAA
CGAAGCCGACACTAAAGAACTGGTTGCACCAGTGGACCGTTTTGCTGCTAGAAGAATCCCCGTCTGTAATGTTACCATATAGGAACTTTATATATATTTTTTTTTTT

10,060 10,080 10,100 10,120 10,140 10,160

AAAAAAAAAAAAAAAAAAAATGCAGCTTCTCAATGATATTCGAATACGCTTTGAGGAGATACAGCCTAATATCCGACAAACTGTTTTACAGATTTACGATCGTACTT
TTTTTTTTTTTTTTTTTTTTACGTCGAAGAGTTACTATAAGCTTATGCGAAACTCCTCTATGTCGGATTATAGGCTGTTTGACAAAATGTCTAAATGCTAGCATGAA

10,180 10,200 10,220 10,240 10,260

GTTACCCATCATTGAATTTTGAACATCCGAACCTGGGAGTTTTCCCTGAAACAGATAGTATATTTGAACCTGTATAATAATATATAGTCTAGCGCTTTACGGAAGAC
CAATGGGTAGTAACTTAAAACTTGTAGGCTTGGACCCTCAAAAGGGACTTTGTCTATCATATAAACTTGGACATATTATTATATATCAGATCGCGAAATGCCTTCTG

10,280 10,300 10,320 10,340 10,360

AATGTATGTATTTCGGTTCCTGGAGAAACTATTGCATCTATTGCATAGGTAATCTTGCACGTCGCATCCCCGGTTCATTTTCTGCGTTTCCATCTTGCACTTCAATA
TTACATACATAAAGCCAAGGACCTCTTTGATAACGTAGATAACGTATCCATTAGAACGTGCAGCGTAGGGGCCAAGTAAAAGACGCAAAGGTAGAACGTGAAGTTAT

10,400 10,420 10,440 10,460 10,480

GCATATCTTT
CGTATAGAAA

10,490

ST/SC ligation / pAnchor-HA (pRS415) (8969 bp)

ACACCGAACTGAGATACCTACAGCGTGAGCTATGAGAAAGCGCCACGCTTCCCGAAGGGAGAAAGGCGGACAGGTATCCGGTAAGCGGCAGGGTCGGAACAGGAGAG
TGTGGCTTGACTCTATGGATGTCGCACTCGATACTCTTTCGCGGTGCGAAGGGCTTCCCTCTTTCCGCCTGTCCATAGGCCATTCGCCGTCCCAGCCTTGTCCTCTC

20 40 60 80 100

CGCACGAGGGAGCTTCCAGGGGGAAACGCCTGGTATCTTTATAGTCCTGTCGGGTTTCGCCACCTCTGACTTGAGCGTCGATTTTTGTGATGCTCGTCAGGGGGGCG
GCGTGCTCCCTCGAAGGTCCCCCTTTGCGGACCATAGAAATATCAGGACAGCCCAAAGCGGTGGAGACTGAACTCGCAGCTAAAAACACTACGAGCAGTCCCCCCGC

120 140 160 180 200

GAGCCTATGGAAAAACGCCAGCAACGCGGCCTTTTTACGGTTCCTGGCCTTTTGCTGGCCTTTTGCTCACATGTTCTTTCCTGCGTTATCCCCTGATTCTGTGGATA
CTCGGATACCTTTTTGCGGTCGTTGCGCCGGAAAAATGCCAAGGACCGGAAAACGACCGGAAAACGAGTGTACAAGAAAGGACGCAATAGGGGACTAAGACACCTAT

220 240 260 280 300 320

ACCGTATTACCGCCTTTGAGTGAGCTGATACCGCTCGCCGCAGCCGAACGACCGAGCGCAGCGAGTCAGTGAGCGAGGAAGCGGAAGAGCGCCCAATACGCAAACCG
TGGCATAATGGCGGAAACTCACTCGACTATGGCGAGCGGCGTCGGCTTGCTGGCTCGCGTCGCTCAGTCACTCGCTCCTTCGCCTTCTCGCGGGTTATGCGTTTGGC

340 360 380 400 420

CCTCTCCCCGCGCGTTGGCCGATTCATTAATGCAGCTGGCACGACAGGTTTCCCGACTGGAAAGCGGGCAGTGAGCGCAACGCAATTAATGTGAGTTAGCTCACTCA
GGAGAGGGGCGCGCAACCGGCTAAGTAATTACGTCGACCGTGCTGTCCAAAGGGCTGACCTTTCGCCCGTCACTCGCGTTGCGTTAATTACACTCAATCGAGTGAGT

440 460 480 500 520

TTAGGCACCCCAGGCTTTACACTTTATGCTTCCGGCTCGTATGTTGTGTGGAATTGTGAGCGGATAACAATTTCACACAGGAAACAGCTATGACCATGATTACGCCA
AATCCGTGGGGTCCGAAATGTGAAATACGAAGGCCGAGCATACAACACACCTTAACACTCGCCTATTGTTAAAGTGTGTCCTTTGTCGATACTGGTACTAATGCGGT

lac promoter LacO M13-rev

540 560 580 600 620 640

AGCGCGCAATTAACCCTCACTAAAGGGAACAAAAGCTGGAGCTCAGTTTATCATTATCAATACTCGCCATTTCAAAGAATACGTAAATAATTAATAGTAGTGATTTT
TCGCGCGTTAATTGGGAGTGATTTCCCTTGTTTTCGACCTCGAGTCAAATAGTAATAGTTATGAGCGGTAAAGTTTCTTATGCATTTATTAATTATCATCACTAAAA

T3
T3 promoter GPD promoter

660 680 700 720 740

CCTAACTTTATTTAGTCAAAAAATTAGCCTTTTAATTCTGCTGTAACCCGTACATGCCCAAAATAGGGGGCGGGTTACACAGAATATATAACATCGTAGGTGTCTGG
GGATTGAAATAAATCAGTTTTTTAATCGGAAAATTAAGACGACATTGGGCATGTACGGGTTTTATCCCCCGCCCAATGTGTCTTATATATTGTAGCATCCACAGACC

GPD promoter

760 780 800 820 840

GTGAACAGTTTATTCCTGGCATCCACTAAATATAATGGAGCCCGCTTTTTAAGCTGGCATCCAGAAAAAAAAAGAATCCCAGCACCAAAATATTGTTTTCTTCACCA
CACTTGTCAAATAAGGACCGTAGGTGATTTATATTACCTCGGGCGAAAAATTCGACCGTAGGTCTTTTTTTTTCTTAGGGTCGTGGTTTTATAACAAAAGAAGTGGT

GPD promoter

860 880 900 920 940 960

```
ACCATCAGTTCATAGGTCCATTCTCTTAGCGCAACTACAGAGAACAGGGGCACAAACAGGCAAAAAACGGGCACAACCTCAATGGAGTGATGCAACCTGCCTGGAGT
TGGTAGTCAAGTATCCAGGTAAGAGAATCGCGTTGATGTCTCTTGTCCCCGTGTTTGTCCGTTTTTTGCCCGTGTTGGAGTTACCTCACTACGTTGGACGGACCTCA
GPD promoter
980 1,000 1,020 1,040 1,060

AAATGATGACACAAGGCAATTGACCCACGCATGTATCTATCTCATTTTCTTACACCTTCTATTACCTTCTGCTCTCTCTGATTTGGAAAAAGCTGAAAAAAAAGGTT
TTTACTACTGTGTTCCGTTAACTGGGTGCGTACATAGATAGAGTAAAAGAATGTGGAAGATAATGGAAGACGAGAGAGACTAAACCTTTTTCGACTTTTTTTTCCAA
GPD promoter
1,080 1,100 1,120 1,140 1,160

GAAACCAGTTCCCTGAAATTATTCCCCTACTTGACTAATAAGTATATAAAGACGGTAGGTATTGATTGTAATTCTGTAAATCTATTTCTTAAACTTCTTAAATTCTA
CTTTGGTCAAGGGACTTTAATAAGGGGATGAACTGATTATTCATATATTTCTGCCATCCATAACTAACATTAAGACATTTAGATAAAGAATTTGAAGAATTTAAGAT
GPD promoter
1,180 1,200 1,220 1,240 1,260 1,280

CTTTTATAGTTAGTCTTTTTTTTAGTTTTAAAACACCAGAACTTAGTTTCGACGGATTCTAGAACTAGTGGATCCATGAGATTCCCATCTATCTTCACCGCTGTTTT
GAAAATATCAATCAGAAAAAAAATCAAAATTTTGTGGTCTTGAATCAAAGCTGCCTAAGATCTTGATCACCTAGGTACTCTAAGGGTAGATAGAAGTGGCGACAAAA
1 2 3 4 5 6 7 8 9 10 11
M R F P S I F T A V L
GPD promoter    Mating factor alpha leader
1,300 1,320 1,340 1,360 1,380

GTTCGCTGCTTCTTCTGCTTTGGCTGCTCCAGCTAACACCACCACCGAAGACGAAACCGCTCAAATCCCAGCTGAAGCTGTTATCGACTACTCTGACTTGGAAGGTG
CAAGCGACGAAGAAGACGAAACCGACGAGGTCGATTGTGGTGGTGGCTTCTGCTTTGGCGAGTTTAGGGTCGACTTCGACAATAGCTGATGAGACTGAACCTTCCAC
12 13 14 15 16 17 18 19 20 21 22 23 24 25 26 27 28 29 30 31 32 33 34 35 36 37 38 39 40 41 42 43 44 45 46
F A A S S A L A A P A N T T T E D E T A Q I P A E A V I D Y S D L E G
Mating factor alpha leader
1,400 1,420 1,440 1,460 1,480

ACTTCGACGCTGCTGCTTTGCCATTGTCTAACTCTACCAACAACGGTTTGTCTTCTACCAACACCACCATCGCTTCTATCGCTGCTAAGGAAGAAGGTGTTCAATTG
TGAAGCTGCGACGACGAAACGGTAACAGATTGAGATGGTTGTTGCCAAACAGAAGATGGTTGTGGTGGTAGCGAAGATAGCGACGATTCCTTCTTCCACAAGTTAAC
47 48 49 50 51 52 53 54 55 56 57 58 59 60 61 62 63 64 65 66 67 68 69 70 71 72 73 74 75 76 77 78 79 80 81 82
D F D A A A L P L S N S T N N G L S S T N T T I A S I A A K E E G V Q L
Mating factor alpha leader
1,500 1,520 1,540 1,560 1,580 1,600

GACAAGAGAGAAGCTAGCGCAGTAACTACCTTGTCTGGCTTATCAGGGGAACAGGGTCCTAGCGGTGACATGACGACAGAGGAGGACAGTGCGACCCACATAAAGTT
CTGTTCTCTCTTCGATCGCGTCATTGATGGAACAGACCGAATAGTCCCCTTGTCCCAGGATCGCCACTGTACTGCTGTCTCCTCCTGTCACGCTGGGTGTATTTCAA
83 84 85 86    2 4 6 8 10 12 14 16 18 20 22 24 26 28
D K R E    V T T L S G L S G E Q G P S G D M T T E E D S A T H I K F
Mat...er    SpyCatcher
1,620 1,640 1,660 1,680 1,700
```

TAGCAAAAGAGATGAAGATGGAAGGGAACTAGCTGGAGCGACCATGGAACTTCGTGACAGCTCAGGGAAGACTATATCAACGTGGATCTCAGATGGTCACGTCAAGG
ATCGTTTTCTCTACTTCTACCTTCCCTTGATCGACCTCGCTGGTACCTTGAAGCACTGTCGAGTCCCTTCTGATATAGTTGCACCTAGAGTCTACCAGTGCAGTTCC
30 32 34 36 38 40 42 44 46 48 50 52 54 56 58 60 62 64
S K R D E D G R E L A G A T M E L R D S S G K T I S T W I S D G H V K
SpyCatcher
1,720 1,740 1,760 1,780 1,800

ACTTCTATTTGTACCCCGGAAAGTACACATTTGTGGAAACCGCCGCGCCGGATGGCTACGAGGTAGCGACCCCGATAGAATTCACCGTTAACGAGGACGGCCAAGTC
TGAAGATAAACATGGGGCCTTTCATGTGTAAACACCTTTGGCGGCGCGGCCTACCGATGCTCCATCGCTGGGGCTATCTTAAGTGGCAATTGCTCCTGCCGGTTCAG
66 68 70 72 74 76 78 80 82 84 86 88 90 92 94 96 98 100
D F Y L Y P G K Y T F V E T A A P D G Y E V A T P I E F T V N E D G Q V
SpyCatcher
1,820 1,840 1,860 1,880 1,900 1,920

ACCGTAGATGGAGAGGCGACAGAGGGTGATGCCCACACTGGGGGCTCCGGCGGATCTGGTGGGAGCGGATCCTACCCATACGATGTTCCAGATTACGCTCAAACATC
TGGCATCTACCTCTCCGCTGTCTCCCACTACGGGTGTGACCCCCGAGGCCGCCTAGACCACCCTCGCCTAGGATGGGTATGCTACAAGGTCTAATGCGAGTTTGTAG
102 104 106 108 110 112 1 2 3 4 5 6 7 8 9 10 11 1 2 3 4 5 6 7 8 9 2
T V D G E A T E G D A H T G G S G G S G G S G S Y P Y D V P D Y A Q T S
SpyCatcher HA tag
1,940 1,960 1,980 2,000 2,020

CTCACCCTCTACAGAAGCATCAGTATCAACCTCTAGTACCTCTTCCTCTGCTTCACAATCATCTGACCCAACTACAACATCTTCGTCCAGTTCATCGTCTTCCCCAT
GAGTGGGAGATGTCTTCGTAGTCATAGTTGGAGATCATGGAGAAGGAGACGAAGTGTTAGTAGACTGGGTTGATGTTGTAGAAGCAGGTCAAGTAGCAGAAGGGGTA
4 6 8 10 12 14 16 18 20 22 24 26 28 30 32 34 36 38
S P S T E A S V S T S S T S S S A S Q S S D P T T T S S S S S S S S P
649 stalk
2,040 2,060 2,080 2,100 2,120 2,140

CTTCCCAATCTGAGGAAATTTCATCATCACCGACGGTCTCAACTACACCATCAACTTCTTCATCATCTTCCTCAATGACTTCAACCACCACAACAAAGTCAATCTCA
GAAGGGTTAGACTCCTTTAAAGTAGTAGTGGCTGCCAGAGTTGATGTGGTAGTTGAAGAAGTAGTAGAAGGAGTTACTGAAGTTGGTGGTGTTGTTTCAGTTAGAGT
40 42 44 46 48 50 52 54 56 58 60 62 64 66 68 70 72 74
S S Q S E E I S S S P T V S T T P S T S S S S S S M T S T T T T K S I S
649 stalk
2,160 2,180 2,200 2,220 2,240

ACTTCCACTACAAGTTCAGCTCCAGTTACAGATGTGACAGTTTCCTCATCGCCTAGTAAATCTACCTCTACTTCGACAAGTACAGAAACATCTAAAACACCTACTTC
TGAAGGTGATGTTCAAGTCGAGGTCAATGTCTACACTGTCAAAGGAGTAGCGGATCATTTAGATGGAGATGAAGCTGTTCATGTCTTTGTAGATTTTGTGGATGAAG
76 78 80 82 84 86 88 90 92 94 96 98 100 102 104 106 108 110
T S T T S S A P V T D V T V S S S P S K S T S T S T S T E T S K T P T S
649 stalk
2,260 2,280 2,300 2,320 2,340

AATGACAGAGTATACATCTAGTACATCGATAATTTCGACTCCAGTTAGTCACTCGCAGACAGGTTTGTCGGCTTCATCAAGTTCATCATCTACAACATCCGGTTCTT
TTACTGTCTCATATGTAGATCATGTAGCTATTAAAGCTGAGGTCAATCAGTGAGCGTCTGTCCAAACAGCCGAAGTAGTTCAAGTAGTAGATGTTGTAGGCCAAGAA
112 114 116 118 120 122 124 126 128 130 132 134 136 138 140 142 144
M T E Y T S S T S I I S T P V S H S Q T G L S A S S S S S S T T S G S
649 stalk
2,360 2,380 2,400 2,420 2,440 2,460

CGTCCACTAAATCAGAAAGTTCGACAACATCTGGCTCTTCCCAGTCCGTGGAATCAACCTCCAGCCACGCCACTGTTCTTGCTAATTCCGCAGAAATGGTCACAACA
GCAGGTGATTTAGTCTTTCAAGCTGTTGTAGACCGAGAAGGGTCAGGCACCTTAGTTGGAGGTCGGTGCGGTGACAAGAACGATTAAGGCGTCTTTACCAGTGTTGT
146 148 150 152 154 156 158 160 162 164 166 168 170 172 174 176 178 180
S S T K S E S S T T S G S S Q S V E S T S S H A T V L A N S A E M V T T
649 stalk
2,480 2,500 2,520 2,540 2,560

TCCTCTAGTTCATCCTCAACATCCGAAATGTCATTAACTAGTACTGCTACCAGTGTACCAGTCTCATCTAGTAGCAGTACGACATATTCTACTAGCGCATCTACACA
AGGAGATCAAGTAGGAGTTGTAGGCTTTACAGTAATTGATCATGACGATGGTCACATGGTCAGAGTAGATCATCGTCATGCTGTATAAGATGATCGCGTAGATGTGT
182 184 186 188 190 192 194 196 198 200 202 204 206 208 210 212 214 216
S S S S S S T S E M S L T S T A T S V P V S S S S S T T Y S T S A S T Q
649 stalk
2,580 2,600 2,620 2,640 2,660

AGCCGTCACTACAACATCTTCTTCCACTGTATCTACAACTTCTTCTAGTACAACGTTAACAAGCGCATTCACACATTCTTCAACCACATCGTCCGACCAGCCACCCA
TCGGCAGTGATGTTGTAGAAGAAGGTGACATAGATGTTGAAGAAGATCATGTTGCAATTGTTCGCGTAAGTGTGTAAGAAGTTGGTGTAGCAGGCTGGTCGGTGGGT
218 220 222 224 226 228 230 232 234 236 238 240 242 244 246 248 250 252
A V T T T S S S T V S T T S S S T T L T S A F T H S S T T S S D Q P P
649 stalk
2,680 2,700 2,720 2,740 2,760 2,780

GCGACACTACAAGTCCATCTACGACACACGAACCTCATGTAACCACTCAGACGTCATCAGAAACATCTTCTTCTAAGTCATCTTCTACTTCTTCTTCAAGTACATCT
CGCTGTGATGTTCAGGTAGATGCTGTGTGCTTGGAGTACATTGGTGAGTCTGCAGTAGTCTTTGTAGAAGAAGATTCAGTAGAAGATGAAGAAGAAGTTCATGTAGA
254 256 258 260 262 264 266 268 270 272 274 276 278 280 282 284 286 288
S D T T S P S T T H E P H V T T Q T S S E T S S S K S S S T S S S S T S
649 stalk
2,800 2,820 2,840 2,860 2,880

CAAACCTCTGAGTCTGCAACACCATCCGATTCCGTATCACCTGGAAGTTCTACATCAACATCTTCTAGTAGCACTTCTACTTCCACTTCTATTTCCAGTGGAGAAAC
GTTTGGAGACTCAGACGTTGTGGTAGGCTAAGGCATAGTGGACCTTCAAGATGTAGTTGTAGAAGATCATCGTGAAGATGAAGGTGAAGATAAAGGTCACCTCTTTG
290 292 294 296 298 300 302 304 306 308 310 312 314 316 318 320 322 324
Q T S E S A T P S D S V S P G S S T S T S S S S T S T S T S I S S G E T
649 stalk
2,900 2,920 2,940 2,960 2,980

GACAACTTCTTCTTCTTCATCATCTGCCACGACCACTTCTAACAGCGCAACCTTGTCAGTCTCTACCACACAAACTTCGATTGAAGCCAGTTCATCTACTACATCTA
CTGTTGAAGAAGAAGAAGTAGTAGACGGTGCTGGTGAAGATTGTCGCGTTGGAACAGTCAGAGATGGTGTGTTTGAAGCTAACTTCGGTCAAGTAGATGATGTAGAT
326 328 330 332 334 336 338 340 342 344 346 348 350 352 354 356 358
T T S S S S S S A T T T S N S A T L S V S T T Q T S I E A S S S T T S
649 stalk
3,000 3,020 3,040 3,060 3,080 3,100

CATCTAGTTCAACAATTACAACTTCAAGTAGTAGCGCTCACATATCGTCGAAATCTCAATCTAGTATTACCTATCCCTCTTCCTCGACATCTTCATCTACATCGTCC
GTAGATCAAGTTGTTAATGTTGAAGTTCATCATCGCGAGTGTATAGCAGCTTTAGAGTTAGATCATAATGGATAGGGAGAAGGAGCTGTAGAAGTAGATGTAGCAGG
360 362 364 366 368 370 372 374 376 378 380 382 384 386 388 390 392 394
T S S S T I T T S S S S A H I S S K S Q S S I T Y P S S S T S S S T S S
649 stalk
3,120 3,140 3,160 3,180 3,200

TCAATTTCTAGCGAATCTGAAAGTTTTGAATCGACATCAGCAGAAGATGCTCCATCAACAGCACCTTCATCAAGTGTCTCTTCTAAGAGTTCTACCTCTACAACATC
AGTTAAAGATCGCTTAGACTTTCAAAACTTAGCTGTAGTCGTCTTCTACGAGGTAGTTGTCGTGGAAGTAGTTCACAGAGAAGATTCTCAAGATGGAGATGTTGTAG
396 398 400 402 404 406 408 410 412 414 416 418 420 422 424 426 428 430
S I S S E S E S F E S T S A E D A P S T A P S S S V S S K S S T S T T S
649 stalk
3,220 3,240 3,260 3,280 3,300

AAGCACATCGACATCTTCAAGCACTCCATCTCCATCACCATCTTCCGTGAGTTCTTCCTCCACCAGCTCATTGACAACTTCTGCTGTATCAACACCAGCTACCTCTC
TTCGTGTAGCTGTAGAAGTTCGTGAGGTAGAGGTAGTGGTAGAAGGCACTCAAGAAGGAGGTGGTCGAGTAACTGTTGAAGACGACATAGTTGTGGTCGATGGAGAG
432 434 436 438 440 442 444 446 448 450 452 454 456 458 460 462 464 466
S T S T S S S T P S P S P S S V S S S S T S S L T T S A V S T P A T S
649 stalk
3,320 3,340 3,360 3,380 3,400 3,420

ATTCTCAAAGTACTGTAGTAACCACCACTACTATTACTACATCAACAGGTCCAGTGATGTCTACGACAACAGCTTATTCTTCTAGTTCTACTAGCAGCTCGGAATCT
TAAGAGTTTCATGACATCATTGGTGGTGATGATAATGATGTAGTTGTCCAGGTCACTACAGATGCTGTTGTCGAATAAGAAGATCAAGATGATCGTCGAGCCTTAGA
468 470 472 474 476 478 480 482 484 486 488 490 492 494 496 498 500 502
H S Q S T V V T T T T I T T S T G P V M S T T T A Y S S S S T S S S E S
649 stalk
3,440 3,460 3,480 3,500 3,520

TCTGAGGTTCAGTCTGTCATGTCATCTACGCCTAGTTCAACATCAACAACAACCAGTTCGGAATCTACTTCATCTAGCTCCACAGCTTCTACCTCACCATCAACCTC
AGACTCCAAGTCAGACAGTACAGTAGATGCGGATCAAGTTGTAGTTGTTGTTGGTCAAGCCTTAGATGAAGTAGATCGAGGTGTCGAAGATGGAGTGGTAGTTGGAG
504 506 508 510 512 514 516 518 520 522 524 526 528 530 532 534 536 538
S E V Q S V M S S T P S S T S T T T S S E S T S S S S T A S T S P S T S
649 stalk
3,540 3,560 3,580 3,600 3,620

```
GCAAACTTTCGAAACTTCTCCTACTATAGGAGGTGTCCCCTCAACCACTTCATTTGTCTCTACGCCAACAACGAAATTGTCGCACACTACTTCCACTATGACAGCAC
CGTTTGAAAGCTTTGAAGAGGATGATATCCTCCACAGGGGAGTTGGTGAAGTAAACAGAGATGCGGTTGTTGCTTTAACAGCGTGTGATGAAGGTGATACTGTCGTG
    540   542   544   546   548   550   552   554   556   558   560   562   564   566   568   570   572
 Q  T  F  E  T  S  P  T  I  G  G  V  P  S  T  T  S  F  V  S  T  P  T  T  K  L  S  H  T  T  S  T  M  T  A
649 stalk
3,640    3,660    3,680    3,700    3,720    3,740

AGTCCGATAGTAAGTCTACCCACTCCTCAAGCACATCGACAGAAGATAAATCATCCACTGCTTCTGCAGTTGACGAAAGCACTACAACATCCACTTCCACGGAGTCT
TCAGGCTATCATTCAGATGGGTGAGGAGTTCGTGTAGCTGTCTTCTATTTAGTAGGTGACGAAGACGTCAACTGCTTTCGTGATGTTGTAGGTGAAGGTGCCTCAGA
574   576   578   580   582   584   586   588   590   592   594   596   598   600   602   604   606   608
 Q  S  D  S  K  S  T  H  S  S  S  T  S  T  E  D  K  S  S  T  A  S  A  V  D  E  S  T  T  T  S  T  S  T  E  S
649 stalk
3,760    3,780    3,800    3,820    3,840

ACTACATCAGTAACATCAGGCACCTCCCATTCCGCTAAAGAATCTTCGTCAAATTCTAAGGTGTATAGTTCACAGACAGCACACTCATCCATAAGTGTTGCATCATC
TGATGTAGTCATTGTAGTCCGTGGAGGGTAAGGCGATTTCTTAGAAGCAGTTTAAGATTCCACATATCAAGTGTCTGTCGTGTGAGTAGGTATTCACAACGTAGTAG
610   612   614   616   618   620   622   624   626   628   630   632   634   636   638   640   642   644
 T  T  S  V  T  S  G  T  S  H  S  A  K  E  S  S  S  N  S  K  V  Y  S  S  Q  T  A  H  S  S  I  S  V  A  S  S
649 stalk
3,860    3,880    3,900    3,920    3,940

ACCTAGTACAAAGGGCGCCCAAATCCAATCTTCTATGGTTGAAATCTCTACCTACGCTGGTTCTGCTAACTCTGTTAACGCTGGTGCTGGTGCTGGTGCTTTGTTCT
TGGATCATGTTTCCCGCGGGTTTAGGTTAGAAGATACCAACTTTAGAGATGGATGCGACCAAGACGATTGAGACAATTGCGACCACGACCACGACCACGAAACAAGA
 646   648   650   652   654   656   658   660   662   664   666   668   670   672   674   676   678   680
 P  S  T  K  G  A  Q  I  Q  S  S  M  V  E  I  S  T  Y  A  G  S  A  N  S  V  N  A  G  A  G  A  G  A  L  F
649...lk        GPI anchor
3,960    3,980    4,000    4,020    4,040    4,060

TGTTGTTGTCTTTGGCTATCATCTAATGATTAATTAACTCGAGATCTGATAACAACAGTGTAGATGTAACAAAATCGACTTTGTTCCCACTGTACTTTTAGCTCGTA
ACAACAACAGAAACCGATAGTAGATTACTAATTAATTGAGCTCTAGACTATTGTTGTCACATCTACATTGTTTTAGCTGAAACAAGGGTGACATGAAAATCGAGCAT
 682   684   686   688 1
 L L L S L A I I *
GPI anchor        alpha factor terminator
4,080    4,100    4,120    4,140    4,160

CAAAATACAATATACTTTTCATTTCTCCGTAAACAACATGTTTTCCCATGTAATATCCTTTTCTATTTTTCGTTCCGTTACCAACTTTACACATACTTTATATAGCT
GTTTTATGTTATATGAAAAGTAAAGAGGCATTTGTTGTACAAAAGGGTACATTATAGGAAAAGATAAAAAGCAAGGCAATGGTTGAAATGTGTATGAAATATATCGA
alpha factor terminator
4,180    4,200    4,220    4,240    4,260    4,280

ATTCACTTCTATACACTAAAAAACTAAGACAATTTTAATTTTGCTGCCTGCCATATTTCAATTTGTTATAAATTCCTATAATTTATCCTATTAGTAGCTAAAAAAAG
TAAGTGAAGATATGTGATTTTTTGATTCTGTTAAAATTAAAACGACGGACGGTATAAAGTTAAACAATATTTAAGGATATTAAATAGGATAATCATCGATTTTTTTC
alpha factor terminator
4,300    4,320    4,340    4,360    4,380
```

ATGAATGTGAATCGAATCCTAAGAGAATTGCGCGCTCACTGGCCGTCGTTTTACAACGTCGTGACTGGGAAAACCCTGGCGTTACCCAACTTAATCGCCTTGCAGCA
TACTTACACTTAGCTTAGGATTCTCTTAACGCGCGAGTGACCGGCAGCAAAATGTTGCAGCACTGACCCTTTTGGGACCGCAATGGGTTGAATTAGCGGAACGTCGT

alpha factor terminator M13-fwd

4,400 4,420 4,440 4,460 4,480

CATCCCCCTTTCGCCAGCTGGCGTAATAGCGAAGAGGCCCGCACCGATCGCCCTTCCCAACAGTTGCGCAGCCTGAATGGCGAATGGACGCGCCCTGTAGCGGCGCA
GTAGGGGGAAAGCGGTCGACCGCATTATCGCTTCTCCGGGCGTGGCTAGCGGGAAGGGTTGTCAACGCGTCGGACTTACCGCTTACCTGCGCGGGACATCGCCGCGT

LacZ alpha

4,500 4,520 4,540 4,560 4,580 4,600

TTAAGCGCGGCGGGTGTGGTGGTTACGCGCAGCGTGACCACTACACTTGCCAGCGCCCTAGCGCCCGCTCCTTTCGCTTTCTTCCCTTCCTTTCTCGCCACGTTCGC
AATTCGCGCCGCCCACACCACCAATGCGCGTCGCACTGGTGATGTGAACGGTCGCGGGATCGCGGGCGAGGAAAGCGAAAGAAGGGAAGGAAAGAGCGGTGCAAGCG

4,620 4,640 4,660 4,680 4,700

CGGCTTTCCCCGTCAAGCTCTAAATCGGGGGCTCCCTTTAGGGTTCCGATTTAGTGCTTTACGGCACCTCGACCCCAAAAAACTTGATTAGGGTGATGGTTCACGTA
GCCGAAAGGGGCAGTTCGAGATTTAGCCCCCGAGGGAAATCCCAAGGCTAAATCACGAAATGCCGTGGAGCTGGGGTTTTTTGAACTAATCCCACTACCAAGTGCAT

4,720 4,740 4,760 4,780 4,800

GTGGGCCATCGCCCTGATAGACGGTTTTTCGCCCTTTGACGTTGGAGTCCACGTTCTTTAATAGTGGACTCTTGTTCCAAACTGGAACAACACTCAACCCTATCTCG
CACCCGGTAGCGGGACTATCTGCCAAAAAGCGGGAAACTGCAACCTCAGGTGCAAGAAATTATCACCTGAGAACAAGGTTTGACCTTGTTGTGAGTTGGGATAGAGC

4,820 4,840 4,860 4,880 4,900 4,920

GTCTATTCTTTTGATTTATAAGGGATTTTGCCGATTTCGGCCTATTGGTTAAAAAATGAGCTGATTTAACAAAAATTTAACGCGAATTTTAACAAAATATTAACGCT
CAGATAAGAAAACTAAATATTCCCTAAAACGGCTAAAGCCGGATAACCAATTTTTTACTCGACTAAATTGTTTTTAAATTGCGCTTAAAATTGTTTTATAATTGCGA

4,940 4,960 4,980 5,000 5,020

TACAATTTCCTGATGCGGTATTTTCTCCTTACGCATCTGTGCGGTATTTCACACCGCATAGGGTAATAACTGATATAATTAAATTGAAGCTCTAATTTGTGAGTTTA
ATGTTAAAGGACTACGCCATAAAAGAGGAATGCGTAGACACGCCATAAAGTGTGGCGTATCCCATTATTGACTATATTAATTTAACTTCGAGATTAAACACTCAAAT

5,040 5,060 5,080 5,100 5,120

GTATACATGCATTTACTTATAATACAGTTTTTTAAGCAAGGATTTTCTTAACTTCTTCGGCGACAGCATCACCGACTTCGGTGGTACTGTTGGAACCACCTAAATCA
CATATGTACGTAAATGAATATTATGTCAAAAAATTCGTTCCTAAAAGAATTGAAGAAGCCGCTGTCGTAGTGGCTGAAGCCACCATGACAACCTTGGTGGATTTAGT

LEU2

5,140 5,160 5,180 5,200 5,220 5,240

CCAGTTCTGATACCTGCATCCAAAACCTTTTTAACTGCATCTTCAATGGCCTTACCTTCTTCAGGCAAGTTCAATGACAATTTCAACATCATTGCAGCAGACAAGAT
GGTCAAGACTATGGACGTAGGTTTTGGAAAAATTGACGTAGAAGTTACCGGAATGGAAGAAGTCCGTTCAAGTTACTGTTAAAGTTGTAGTAACGTCGTCTGTTCTA

LEU2

5,260 5,280 5,300 5,320 5,340

AGTGGCGATAGGGTTGACCTTATTCTTTGGCAAATCTGGAGCAGAACCGTGGCATGGTTCGTACAAACCAAATGCGGTGTTCTTGTCTGGCAAAGAGGCCAAGGACG
TCACCGCTATCCCAACTGGAATAAGAAACCGTTTAGACCTCGTCTTGGCACCGTACCAAGCATGTTTGGTTTACGCCACAAGAACAGACCGTTTCTCCGGTTCCTGC
LEU2
5,360 5,380 5,400 5,420 5,440

CAGATGGCAACAAACCCAAGGAACCTGGGATAACGGAGGCTTCATCGGAGATGATATCACCAAACATGTTGCTGGTGATTATAATACCATTTAGGTGGGTTGGGTTC
GTCTACCGTTGTTTGGGTTCCTTGGACCCTATTGCCTCCGAAGTAGCCTCTACTATAGTGGTTTGTACAACGACCACTAATATTATGGTAAATCCACCCAACCCAAG
LEU2
5,460 5,480 5,500 5,520 5,540 5,560

TTAACTAGGATCATGGCGGCAGAATCAATCAATTGATGTTGAACCTTCAATGTAGGGAATTCGTTCTTGATGGTTTCCTCCACAGTTTTTCTCCATAATCTTGAAGA
AATTGATCCTAGTACCGCCGTCTTAGTTAGTTAACTACAACTTGGAAGTTACATCCCTTAAGCAAGAACTACCAAAGGAGGTGTCAAAAAGAGGTATTAGAACTTCT
LEU2
5,580 5,600 5,620 5,640 5,660

GGCCAAAACATTAGCTTTATCCAAGGACCAAATAGGCAATGGTGGCTCATGTTGTAGGGCCATGAAAGCGGCCATTCTTGTGATTCTTTGCACTTCTGGAACGGTGT
CCGGTTTTGTAATCGAAATAGGTTCCTGGTTTATCCGTTACCACCGAGTACAACATCCCGGTACTTTCGCCGGTAAGAACACTAAGAAACGTGAAGACCTTGCCACA
LEU2
5,680 5,700 5,720 5,740 5,760

ATTGTTCACTATCCCAAGCGACACCATCACCATCGTCTTCCTTTCTCTTACCAAAGTAAATACCTCCCACTAATTCTCTGACAACAACGAAGTCAGTACCTTTAGCA
TAACAAGTGATAGGGTTCGCTGTGGTAGTGGTAGCAGAAGGAAAGAGAATGGTTTCATTTATGGAGGGTGATTAAGAGACTGTTGTTGCTTCAGTCATGGAAATCGT
LEU2
5,780 5,800 5,820 5,840 5,860 5,880

AATTGTGGCTTGATTGGAGATAAGTCTAAAAGAGAGTCGGATGCAAAGTTACATGGTCTTAAGTTGGCGTACAATTGAAGTTCTTTACGGATTTTTAGTAAACCTTG
TTAACACCGAACTAACCTCTATTCAGATTTTCTCTCAGCCTACGTTTCAATGTACCAGAATTCAACCGCATGTTAACTTCAAGAAATGCCTAAAAATCATTTGGAAC
LEU2
5,900 5,920 5,940 5,960 5,980

TTCAGGTCTAACACTACCGGTACCCCATTTAGGACCACCCACAGCACCTAACAAAACGGCATCAGCCTTCTTGGAGGCTTCCAGCGCCTCATCTGGAAGTGGAACAC
AAGTCCAGATTGTGATGGCCATGGGGTAAATCCTGGTGGGTGTCGTGGATTGTTTTGCCGTAGTCGGAAGAACCTCCGAAGGTCGCGGAGTAGACCTTCACCTTGTG
LEU2
6,000 6,020 6,040 6,060 6,080

CTGTAGCATCGATAGCAGCACCACCAATTAAATGATTTTCGAAATCGAACTTGACATTGGAACGAACATCAGAAATAGCTTTAAGAACCTTAATGGCTTCGGCTGTG
GACATCGTAGCTATCGTCGTGGTGGTTAATTTACTAAAAGCTTTAGCTTGAACTGTAACCTTGCTTGTAGTCTTTATCGAAATTCTTGGAATTACCGAAGCCGACAC
LEU2
6,100 6,120 6,140 6,160 6,180 6,200

ATTTCTTGACCAACGTGGTCACCTGGCAAAACGACGATCTTCTTAGGGGCAGACATTAGAATGGTATATCCTTGAAATATATATATATATATNTNGCTGAAATGTAA
TAAAGAACTGGTTGCACCAGTGGACCGTTTTGCTGCTAGAAGAATCCCCGTCTGTAATCTTACCATATAGGAACTTTATATATATATATATANANCGACTTTACATT

LEU2

6,220 6,240 6,260 6,280 6,300

AAGGTAAGAAAAGTTAGAAAGTAAGACGATTGCTAACCACCTATTGGAAAAAACAATAGGTCCTTAAATAATATTGTCAACTTCAAGTATTGTGATGCAAGCATTTA
TTCCATTCTTTTCAATCTTTCATTCTGCTAACGATTGGTGGATAACCTTTTTTGTTATCCAGGAATTTATTATAACAGTTGAAGTTCATAACACTACGTTCGTAAAT

6,320 6,340 6,360 6,380 6,400 6,420

GTCATGAACGCTTCTCTATTCTATATGAAAAGCCGGTTCCGGCGCTCTCACCTTTCCTTTTTCTCCCAATTTTTCAGTTGAAAAAGGTATATGCGTCAGGCGACCTC
CAGTACTTGCGAAGAGATAAGATATACTTTTCGGCCAAGGCCGCGAGAGTGGAAAGGAAAAAGAGGGTTAAAAAGTCAACTTTTTCCATATACGCAGTCCGCTGGAG

6,440 6,460 6,480 6,500 6,520

TGAAATTAACAAAAAATTTCCAGTCATCGAATTTGATTCTGTGCGATAGCGCCCCTGTGTGTTCTCGTTATGTTGAGGAAAAAAATAATGGTTGCTAAGAGATTCGA
ACTTTAATTGTTTTTTAAAGGTCAGTAGCTTAAACTAAGACACGCTATCGCGGGGACACACAAGAGCAATACAACTCCTTTTTTTATTACCAACGATTCTCTAAGCT

6,540 6,560 6,580 6,600 6,620

ACTCTTGCATCTTACGATACCTGAGTATTCCCACAGTTTGAAAAGCTGTGGTATGGTGCACTCTCAGTACAATCTGCTCTGATGCCGCATAGTTAAGCCAGCCCCGA
TGAGAACGTAGAATGCTATGGACTCATAAGGGTGTCAAACTTTTCGACACCATACCACGTGAGAGTCATGTTAGACGAGACTACGGCGTATCAATTCGGTCGGGGCT

6,640 6,660 6,680 6,700 6,720 6,740

CACCCGCCAACACCCGCTGACGCGCCCTGACGGGCTTGTCTGCTCCCGGCATCCGCTTACAGACAAGCTGTGACCGTCTCCGGGAGCTGCATGTGTCAGAGGTTTTC
GTGGGCGGTTGTGGGCGACTGCGCGGGACTGCCCGAACAGACGAGGGCCGTAGGCGAATGTCTGTTCGACACTGGCAGAGGCCCTCGACGTACACAGTCTCCAAAAG

6,760 6,780 6,800 6,820 6,840

ACCGTCATCACCGAAACGCGCGAGACGAAAGGGCCTCGTGATACGCCTATTTTTATAGGTTAATGTCATGATAATAATGGTTTCTTAGTAgatcgcttgcctgtaac
TGGCAGTAGTGGCTTTGCGCGCTCTGCTTTCCCGGAGCACTATGCGGATAAAAATATCCAATTACAGTACTATTATTACCAAAGAATCATctagcgaacggacattg

CEN/ARS

6,860 6,880 6,900 6,920 6,940

ttacacgcgcctcgtatcttttaatgatggaataatttgggaatttactctgtgtttatttatttttatgttttgtatttggatttagaaagtaaataaagaaggt
aatgtgcgcggagcatagaaaattactaccttattaaacccttaaatgagacacaaataaataaaaatacaaaacataaacctaaaatctttcatttatttcttcca

CEN/ARS

6,960 6,980 7,000 7,020 7,040 7,060

agaagagttacggaatgaagaaaaaaaaataaacaaaggtttaaaaaatttcaacaaaaagcgtactttacatatatatttattagacaagaaaagcagattaaata
tcttctcaatgccttacttcttttttttttatttgtttccaaatttttaaagttgtttttcgcatgaaatgtatatataaataatctgttcttttcgtctaatttat

CEN/ARS

7,080 7,100 7,120 7,140 7,160

gatatacattcgattaacgataagtaaaatgtaaaatcacaggattttcgtgtgtggtcttctacacagacaagatgaaacaattcggcattaatacctgagagcag
ctatatgtaagctaattgctattcattttacattttagtgtcctaaaagcacacaccagaagatgtgtctgttctactttgttaagccgtaattatggactctcgtc
CEN/ARS
7,180 7,200 7,220 7,240 7,260

gaagagcaagataaaaggtagtatttgttggcgatcccccctagagtcttttacatcttcggaaaacaaaaactattttttctttaatttctttttttactttctatt
cttctcgttctattttccatcataaacaaccgctaggggggatctcagaaaatgtagaagccttttgtttttgataaaaaagaaattaaagaaaaaaatgaaagataa
CEN/ARS
7,280 7,300 7,320 7,340 7,360 7,380

tttaatttatatatttatattaaaaaatttaaattataattatttttatagcacgtgatGTTCAGGTGGCACTTTTCGGGGAAATGTGCGCGGAACCCCTATTTGTT
aaattaaatatataaatataattttttaaatttaatattaataaaaatatcgtgcactaCAAGTCCACCGTGAAAAGCCCCTTTACACGCGCCTTGGGGATAAACAA
CEN/ARS
7,400 7,420 7,440 7,460 7,480

TATTTTTCTAAATACATTCAAATATGTATCCGCTCATGAGACAATAACCCTGATAAATGCTTCAATAATATTGAAAAAGGAAGAGTATGAGTATTCAACATTTCCGT
ATAAAAAGATTTATGTAAGTTTATACATAGGCGAGTACTCTGTTATTGGGACTATTTACGAAGTTATTATAACTTTTTCCTTCTCATACTCATAAGTTGTAAAGGCA
7,500 7,520 7,540 7,560 7,580

GTCGCCCTTATTCCCTTTTTTGCGGCATTTTGCCTTCCTGTTTTTGCTCACCCAGAAACGCTGGTGAAAGTAAAAGATGCTGAAGATCAGTTGGGTGCACGAGTGGG
CAGCGGGAATAAGGGAAAAAACGCCGTAAAACGGAAGGACAAAAACGAGTGGGTCTTTGCGACCACTTTCATTTTCTACGACTTCTAGTCAACCCACGTGCTCACCC
7,600 7,620 7,640 7,660 7,680 7,700

TTACATCGAACTGGATCTCAACAGCGGTAAGATCCTTGAGAGTTTTCGCCCCGAAGAACGTTTTCCAATGATGAGCACTTTTAAAGTTCTGCTATGTGGCGCGGTAT
AATGTAGCTTGACCTAGAGTTGTCGCCATTCTAGGAACTCTCAAAAGCGGGGCTTCTTGCAAAAGGTTACTACTCGTGAAAATTTCAAGACGATACACCGCGCCATA
AmpR
7,720 7,740 7,760 7,780 7,800

TATCCCGTATTGACGCCGGGCAAGAGCAACTCGGTCGCCGCATACACTATTCTCAGAATGACTTGGTTGAGTACTCACCAGTCACAGAAAAGCATCTTACGGATGGC
ATAGGGCATAACTGCGGCCCGTTCTCGTTGAGCCAGCGGCGTATGTGATAAGAGTCTTACTGAACCAACTCATGAGTGGTCAGTGTCTTTTCGTAGAATGCCTACCG
AmpR
7,820 7,840 7,860 7,880 7,900

ATGACAGTAAGAGAATTATGCAGTGCTGCCATAACCATGAGTGATAACACTGCGGCCAACTTACTTCTGACAACGATCGGAGGACCGAAGGAGCTAACCGCTTTTTT
TACTGTCATTCTCTTAATACGTCACGACGGTATTGGTACTCACTATTGTGACGCCGGTTGAATGAAGACTGTTGCTAGCCTCCTGGCTTCCTCGATTGGCGAAAAAA
AmpR
7,920 7,940 7,960 7,980 8,000 8,020

GCACAACATGGGGGATCATGTAACTCGCCTTGATCGTTGGGAACCGGAGCTGAATGAAGCCATACCAAACGACGAGCGTGACACCACGATGCCTGTAGCAATGGCAA
CGTGTTGTACCCCCTAGTACATTGAGCGGAACTAGCAACCCTTGGCCTCGACTTACTTCGGTATGGTTTGCTGCTCGCACTGTGGTGCTACGGACATCGTTACCGTT
AmpR
8,040 8,060 8,080 8,100 8,120

```
CAACGTTGCGCAAACTATTAACTGGCGAACTACTTACTCTAGCTTCCCGGCAACAATTAATAGACTGGATGGAGGCGGATAAAGTTGCAGGACCACTTCTGCGCTCG
GTTGCAACGCGTTTGATAATTGACCGCTTGATGAATGAGATCGAAGGGCCGTTGTTAATTATCTGACCTACCTCCGCCTATTTCAACGTCCTGGTGAAGACGCGAGC
                                                 AmpR
      8,140               8,160               8,180               8,200               8,220

GCCCTTCCGGCTGGCTGGTTTATTGCTGATAAATCTGGAGCCGGTGAGCGTGGGTCTCGCGGTATCATTGCAGCACTGGGGCCAGATGGTAAGCCCTCCCGTATCGT
CGGGAAGGCCGACCGACCAAATAACGACTATTTAGACCTCGGCCACTCGCACCCAGAGCGCCATAGTAACGTCGTGACCCCGGTCTACCATTCGGGAGGGCATAGCA
                                                 AmpR
8,240               8,260               8,280               8,300               8,320               8,340

AGTTATCTACACGACGGGGAGTCAGGCAACTATGGATGAACGAAATAGACAGATCGCTGAGATAGGTGCCTCACTGATTAAGCATTGGTAACTGTCAGACCAAGTTT
TCAATAGATGTGCTGCCCCTCAGTCCGTTGATACCTACTTGCTTTATCTGTCTAGCGACTCTATCCACGGAGTGACTAATTCGTAACCATTGACAGTCTGGTTCAAA
                                         AmpR
            8,360               8,380               8,400               8,420               8,440

ACTCATATATACTTTAGATTGATTTAAAACTTCATTTTTAATTTAAAAGGATCTAGGTGAAGATCCTTTTTGATAATCTCATGACCAAAATCCCTTAACGTGAGTTT
TGAGTATATATGAAATCTAACTAAATTTTGAAGTAAAAATTAAATTTTCCTAGATCCACTTCTAGGAAAAACTATTAGAGTACTGGTTTTAGGGAATTGCACTCAAA
      8,460               8,480               8,500               8,520               8,540               8,560

TCGTTCCACTGAGCGTCAGACCCCGTAGAAAAGATCAAAGGATCTTCTTGAGATCCTTTTTTTCTGCGCGTAATCTGCTGCTTGCAAACAAAAAAACCACCGCTACC
AGCAAGGTGACTCGCAGTCTGGGGCATCTTTTCTAGTTTCCTAGAAGAACTCTAGGAAAAAAAGACGCGCATTAGACGACGAACGTTTGTTTTTTTGGTGGCGATGG
                  8,580               8,600               8,620               8,640               8,660

AGCGGTGGTTTGTTTGCCGGATCAAGAGCTACCAACTCTTTTTCCGAAGGTAACTGGCTTCAGCAGAGCGCAGATACCAAATACTGTTCTTCTAGTGTAGCCGTAGT
TCGCCACCAAACAAACGGCCTAGTTCTCGATGGTTGAGAAAAAGGCTTCCATTGACCGAAGTCGTCTCGCGTCTATGGTTTATGACAAGAAGATCACATCGGCATCA
            8,680               8,700               8,720               8,740               8,760

TAGGCCACCACTTCAAGAACTCTGTAGCACCGCCTACATACCTCGCTCTGCTAATCCTGTTACCAGTGGCTGCTGCCAGTGGCGATAAGTCGTGTCTTACCGGGTTG
ATCCGGTGGTGAAGTTCTTGAGACATCGTGGCGGATGTATGGAGCGAGACGATTAGGACAATGGTCACCGACGACGGTCACCGCTATTCAGCACAGAATGGCCCAAC
     8,780               8,800               8,820               8,840               8,860               8,880

GACTCAAGACGATAGTTACCGGATAAGGCGCAGCGGTCGGGCTGAACGGGGGGTTCGTGCACACAGCCCAGCTTGGAGCGAACGACCT
CTGAGTTCTGCTATCAATGGCCTATTCCGCGTCGCCAGCCCGACTTGCCCCCCAAGCACGTGTGTCGGGTCGAACCTCGCTTGCTGGA
        8,890     8,900     8,910     8,920     8,930     8,940     8,950     8,960
```

ST/SC ligation / pAnchor-HA (pRS403) (8552 bp)

```
tcgcgcgtttcggtgatgacggtgaaaacctctgacacatgcagctcccggagacggtcacagcttgtctgtaagcggatgccgggagcagacaagcccgtcagggc
agcgcgcaaagccactactgccacttttggagactgtgtacgtcgagggcctctgccagtgtcgaacagacattcgcctacggccctcgtctgttcgggcagtcccg
20 40 60 80 100

gcgtcagcgggtgttggcgggtgtcggggctggcttaactatgcggcatcagagcagattgtactgagagtgcaccataaattcccgttttaagagcttggtgagcg
cgcagtcgcccacaaccgcccacagccccgaccgaattgatacgccgtagtctcgtctaacatgactctcacgtggtatttaagggcaaaattctcgaaccactcgc
HIS3
120 140 160 180 200

ctaggagtcactgccaggtatcgtttgaacacggcattagtcagggaagtcataacacagtcctttcccgcaattttctttttctattactcttggcctcctctagt
gatcctcagtgacggtccatagcaaacttgtgccgtaatcagtcccttcagtattgtgtcaggaaagggcgttaaaagaaaaagataatgagaaccggaggagatca
HIS3
220 240 260 280 300 320

acactctatatttttttatgcctcggtaatgattttcattttttttttttcccctagcggatgactcttttttttttcttagcgattggcattatcacataatgaatta
tgtgagatataaaaaaatacggagccattactaaaagtaaaaaaaaaaaaggggatcgcctactgagaaaaaaaaaagaatcgctaaccgtaatagtgtattacttaat
HIS3
340 360 380 400 420

tacattatataaagtaatgtgatttcttcgaagaatatactaaaaaatgagcaggcaagataaacgaaggcaaagatgacagagcagaaagccctagtaaagcgtat
atgtaatatatttcattacactaaagaagcttcttatatgattttttactcgtccgttctatttgcttccgtttctactgtctcgtctttcgggatcatttcgcata
HIS3
440 460 480 500 520

tacaaatgaaaccaagattcagattgcgatctctttaaagggtggtcccctagcgatagagcactcgatcttcccagaaaaagaggcagaagcagtagcagaacagg
atgtttactttggttctaagtctaacgctagagaaatttcccaccaggggatcgctatctcgtgagctagaagggtctttttctccgtcttcgtcatcgtcttgtcc
HIS3
540 560 580 600 620 640

ccacacaatcgcaagtgattaacgtccacacaggtatagggtttctggaccatatgatacatgctctggccaagcattccggctggtcgctaatcgttgagtgcatt
ggtgtgttagcgttcactaattgcaggtgtgtccatatcccaaagacctggtatactatgtacgagaccggttcgtaaggccgaccagcgattagcaactcacgtaa
HIS3
660 680 700 720 740

ggtgacttacacatagacgaccatcacaccactgaagactgcgggattgctctcggtcaagcttttaaagaggccctactggcgcgtggagtaaaaaggtttggatc
ccactgaatgtgtatctgctggtagtgtggtgacttctgacgccctaacgagagccagttcgaaaatttctccgggatgaccgcgcacctcatttttccaaacctag
HIS3
760 780 800 820 840
```

aggatttgcgcctttggatgaggcactttccagagcggtggtagatctttcgaacaggccgtacgcagttgtcgaacttggtttgcaaagggagaaagtaggagatc
tcctaaacgcggaaacctactccgtgaaaggtctcgccaccatctagaaagcttgtccggcatgcgtcaacagcttgaaccaaacgtttccctctttcatcctctag
HIS3
860 880 900 920 940 960

tctcttgcgagatgatcccgcattttcttgaaagctttgcagaggctagcagaattaccctccacgttgattgtctgcgaggcaagaatgatcatcaccgtagtgag
agagaacgctctactagggcgtaaaagaactttcgaaacgtctccgatcgtcttaatgggaggtgcaactaacagacgctccgttcttactagtagtggcatcactc
HIS3
980 1,000 1,020 1,040 1,060

agtgcgttcaaggctcttgcggttgccataagagaagccacctcgcccaatggtaccaacgatgttccctccaccaaaggtgttcttatgtagtgacaccgattatt
tcacgcaagttccgagaacgccaacggtattctcttcggtggagcgggttaccatggttgctacaagggaggtggtttccacaagaatacatcactgtggctaataa
HIS3
1,080 1,100 1,120 1,140 1,160

taaagctgcagcatacgatatatatacatgtgtatatatgtatacctatgaatgtcagtaagtatgtatacgaacagtatgatactgaagatgacaaggtaatgcat
atttcgacgtcgtatgctatatatatgtacacatatatacatatggatacttacagtcattcatacatatgcttgtcatactatgacttctactgttccattacgta
HIS3
1,180 1,200 1,220 1,240 1,260 1,280

cattctatacgtgtcattctgaacgaggcgcgctttcctttttttctttttgctttttctttttttttctcttgaactcgacggatctatgcggtgtgaaataccgca
gtaagatatgcacagtaagacttgctccgcgcgaaaggaaaaaagaaaaacgaaaaagaaaaaaaaagagaacttgagctgcctagatacgccacactttatggcgt
HIS3
1,300 1,320 1,340 1,360 1,380

cagatgcgtaaggagaaaataccgcatcaggaaattgtaaacgttaatattttgttaaaattcgcgttaaatttttgttaaatcagctcattttttaaccaataggc
gtctacgcattcctcttttatggcgtagtcctttaacatttgcaattataaaacaattttaagcgcaatttaaaaacaatttagtcgagtaaaaaattggttatccg
1,400 1,420 1,440 1,460 1,480

cgaaatcggcaaaatcccttataaatcaaaagaatagaccgagatagggttgagtgttgttccagtttggaacaagagtccactattaaagaacgtggactccaacg
gctttagccgttttagggaatatttagttttcttatctggctctatcccaactcacaacaaggtcaaaccttgttctcaggtgataatttcttgcacctgaggttgc
F1 ori
1,500 1,520 1,540 1,560 1,580 1,600

tcaaagggcgaaaaaccgtctatcagggcgatggcccactacgtgaaccatcaccctaatcaagttttttggggtcgaggtgccgtaaagcactaaatcggaaccct
agtttcccgctttttggcagatagtcccgctaccgggtgatgcacttggtagtgggattagttcaaaaaaccccagctccacggcatttcgtgatttagccttggga
F1 ori
1,620 1,640 1,660 1,680 1,700

aaagggagcccccgatttagagcttgacggggaaagccggcgaacgtggcgagaaaggaagggaagaaagcgaaaggagcgggcgctagggcgctggcaagtgtagc
tttccctcgggggctaaatctcgaactgcccctttcggccgcttgcaccgctctttccttcccttctttcgctttcctcgcccgcgatcccgcgaccgttcacatcg
F1 ori
1,720 1,740 1,760 1,780 1,800

ggtcacgctgcgcgtaaccaccacacccgccgcgcttaatgcgccgctacagggcgcgtcgcgccattcgccattcaggctgcgcaactgttgggaagggcgatcgg
ccagtgcgacgcgcattggtggtgtgggcggcgcgaattacgcggcgatgtcccgcgcagcgcggtaagcggtaagtccgacgcgttgacaacccttcccgctagcc

F1 ori LacZ alpha

1,820 1,840 1,860 1,880 1,900 1,920

tgcgggcctcttcgctattacgccagctggcgaaagggggatgtgctgcaaggcgattaagttgggtaacgccagggttttcccagtcacgacgttgtaaaacgacg
acgcccggagaagcgataatgcggtcgaccgctttccccctacacgacgttccgctaattcaacccattgcggtcccaaaagggtcagtgctgcaacattttgctgc

LacZ alpha

1,940 1,960 1,980 2,000 2,020

gccagtgagcgcgcAAGCTTACCAGTTCTCACACGGAACACCACTAATGGACACAAATTCGAAATACTTTGACCCTATTTTCGAGGACCTTGTCACCTTGAGCCCAA
cggtcactcgcgcgTTCGAATGGTCAAGAGTGTGCCTTGTGGTGATTACCTGTGTTTAAGCTTTATGAAACTGGGATAAAAGCTCCTGGAACAGTGGAACTCGGGTT

GPD promoter

2,040 2,060 2,080 2,100 2,120 2,140

GAGAGCCAAGATTTAAATTTTCCTATGACTTGATGCAAATTCCCAAAGCTAATAACATGCAAGACACGTACGGTCAAGAAGACATATTTGACCTCTTAACAGGTTCA
CTCTCGGTTCTAAATTTAAAAGGATACTGAACTACGTTTAAGGGTTTCGATTATTGTACGTTCTGTGCATGCCAGTTCTTCTGTATAAACTGGAGAATTGTCCAAGT

GPD promoter

2,160 2,180 2,200 2,220 2,240

GACGCGACTGCCTCATCAGTAAGACCCGTTGAAAAGAACTTACCTGAAAAAAACGAATATATACTAGCGTTGAATGTTAGCGTCAACAACAAGAAGTTTAATGACGC
CTGCGCTGACGGAGTAGTCATTCTGGGCAACTTTTCTTGAATGGACTTTTTTTGCTTATATATGATCGCAACTTACAATCGCAGTTGTTGTTCTTCAAATTACTGCG

GPD promoter

2,260 2,280 2,300 2,320 2,340

GGAGGCCAAGGCAAAAAGATTCCTTGATTACGTAAGGGAGTTAGAATCATTTTGAATAAAAAACACGCTTTTTCAGTTCGAGTTTATCATTATCAATACTGCCATTT
CCTCCGGTTCCGTTTTTCTAAGGAACTAATGCATTCCCTCAATCTTAGTAAAACTTATTTTTTGTGCGAAAAAGTCAAGCTCAAATAGTAATAGTTATGACGGTAAA

GPD promoter

2,360 2,380 2,400 2,420 2,440 2,460

CAAAGAATACGTAAATAATTAATAGTAGTGATTTTCCTAACTTTATTTAGTCAAAAAATTAGCCTTTTAATTCTGCTGTAACCCGTACATGCCCAAAATAGGGGGCG
GTTTCTTATGCATTTATTAATTATCATCACTAAAAGGATTGAAATAAATCAGTTTTTTAATCGGAAAATTAAGACGACATTGGGCATGTACGGGTTTTATCCCCCGC

GPD promoter

2,480 2,500 2,520 2,540 2,560

GGTTACACAGAATATATAACATCGTAGGTGTCTGGGTGAACAGTTTATTCCTGGCATCCACTAAATATAATGGAGCCCGCTTTTTAAGCTGGCATCCAGAAAAAAAA
CCAATGTGTCTTATATATTGTAGCATCCACAGACCCACTTGTCAAATAAGGACCGTAGGTGATTTATATTACCTCGGGCGAAAAATTCGACCGTAGGTCTTTTTTTT

GPD promoter

2,580 2,600 2,620 2,640 2,660

AGAATCCCAGCACCAAAATATTGTTTTCTTCACCAACCATCAGTTCATAGGTCCATTCTCTTAGCGCAACTACAGAGAACAGGGGCACAAACAGGCAAAAAACGGGC
TCTTAGGGTCGTGGTTTTATAACAAAAGAAGTGGTTGGTAGTCAAGTATCCAGGTAAGAGAATCGCGTTGATGTCTCTTGTCCCCGTGTTTGTCCGTTTTTTGCCCG
GPD promoter
2,680 2,700 2,720 2,740 2,760 2,780

ACAACCTCAATGGAGTGATGCAACCTGCCTGGAGTAAATGATGACACAAGGCAATTGACCCACGCATGTATCTATCTCATTTTCTTACACCTTCTATTACCTTCTGC
TGTTGGAGTTACCTCACTACGTTGGACGGACCTCATTTACTACTGTGTTCCGTTAACTGGGTGCGTACATAGATAGAGTAAAAGAATGTGGAAGATAATGGAAGACG
GPD promoter
2,800 2,820 2,840 2,860 2,880

TCTCTCTGATTTGGAAAAAGCTGAAAAAAAAAGGTTGAAACCAGTTCCCTGAAATTATTCCCCTACTTGACTAATAAGTATATAAAGACGGTAGGTATTGATTGTAAT
AGAGAGACTAAACCTTTTTCGACTTTTTTTTTCCAACTTTGGTCAAGGGACTTTAATAAGGGGATGAACTGATTATTCATATATTTCTGCCATCCATAACTAACATTA
GPD promoter
2,900 2,920 2,940 2,960 2,980

TCTGTAAATCTATTTCTTAAACTTCTTAAATTCTACTTTTATAGTTAGTCTTTTTTTTAGTTTTAAAACACCAAGAACTTAGTTTCGAATAAACACACATAAACAGA
AGACATTTAGATAAAGAATTTGAAGAATTTAAGATGAAAATATCAATCAGAAAAAAAATCAAAATTTTGTGGTTCTTGAATCAAAGCTTATTTGTGTGTATTTGTCT
GPD promoter
3,000 3,020 3,040 3,060 3,080 3,100

ATTCATGAGATTCCCATCTATCTTCACCGCTGTTTTGTTCGCTGCTTCTTCTGCTTTGGCTGCTCCAGCTAACACCACCACCGAAGACGAAACCGCTCAAATCCCAG
TAAGTACTCTAAGGGTAGATAGAAGTGGCGACAAAACAAGCGACGAAGAAGACGAAACCGACGAGGTCGATTGTGGTGGTGGCTTCTGCTTTGGCGAGTTTAGGGTC
1 2 3 4 5 6 7 8 9 10 11 12 13 14 15 16 17 18 19 20 21 22 23 24 25 26 27 28 29 30 31 32 33 34
M R F P S I F T A V L F A A S S A L A A P A N T T T E D E T A Q I P
Mating factor alpha leader
3,120 3,140 3,160 3,180 3,200

CTGAAGCTGTTATCGACTACTCTGACTTGGAAGGTGACTTCGACGCTGCTGCTTTGCCATTGTCTAACTCTACCAACAACGGTTTGTCTTCTACCAACACCACCATC
GACTTCGACAATAGCTGATGAGACTGAACCTTCCACTGAAGCTGCGACGACGAAACGGTAACAGATTGAGATGGTTGTTGCCAAACAGAAGATGGTTGTGGTGGTAG
35 36 37 38 39 40 41 42 43 44 45 46 47 48 49 50 51 52 53 54 55 56 57 58 59 60 61 62 63 64 65 66 67 68 69 70
A E A V I D Y S D L E G D F D A A A L P L S N S T N N G L S S T N T T I
Mating factor alpha leader
3,220 3,240 3,260 3,280 3,300

GCTTCTATCGCTGCTAAGGAAGAAGGTGTTCAATTGGACAAGAGAGAAGCTAGCGCAGTAACTACCTTGTCTGGCTTATCAGGGGAACAGGGTCCTAGCGGTGACAT
CGAAGATAGCGACGATTCCTTCTTCCACAAGTTAACCTGTTCTCTCTTCGATCGCGTCATTGATGGAACAGACCGAATAGTCCCCTTGTCCCAGGATCGCCACTGTA
71 72 73 74 75 76 77 78 79 80 81 82 83 84 85 86 2 4 6 8 10 12 14 16
A S I A A K E E G V Q L D K R E V T T L S G L S G E Q G P S G D M
Mating factor alpha leader SpyCatcher
3,320 3,340 3,360 3,380 3,400 3,420

GACGACAGAGGAGGACAGTGCGACCCACATAAAGTTTAGCAAAAGAGATGAAGATGGAAGGGAACTAGCTGGAGCGACCATGGAACTTCGTGACAGCTCAGGGAAGA
CTGCTGTCTCCTCCTGTCACGCTGGGTGTATTTCAAATCGTTTTCTCTACTTCTACCTTCCCTTGATCGACCTCGCTGGTACCTTGAAGCACTGTCGAGTCCCTTCT
18 20 22 24 26 28 30 32 34 36 38 40 42 44 46 48 50 52
T T E E D S A T H I K F S K R D E D G R E L A G A T M E L R D S S G K
SpyCatcher
3,440 3,460 3,480 3,500 3,520

CTATATCAACGTGGATCTCAGATGGTCACGTCAAGGACTTCTATTTGTACCCCGGAAAGTACACATTTGTGGAAACCGCCGCGCCGGATGGCTACGAGGTAGCGACC
GATATAGTTGCACCTAGAGTCTACCAGTGCAGTTCCTGAAGATAAACATGGGGCCTTTCATGTGTAAACACCTTTGGCGGCGCGGCCTACCGATGCTCCATCGCTGG
54 56 58 60 62 64 66 68 70 72 74 76 78 80 82 84 86 88
T I S T W I S D G H V K D F Y L Y P G K Y T F V E T A A P D G Y E V A T
SpyCatcher
3,540 3,560 3,580 3,600 3,620

CCGATAGAATTCACCGTTAACGAGGACGGCCAAGTCACCGTAGATGGAGAGGCGACAGAGGGTGATGCCCACACTGGGGGCTCCGGCGGATCTGGTGGGAGCGGATC
GGCTATCTTAAGTGGCAATTGCTCCTGCCGGTTCAGTGGCATCTACCTCTCCGCTGTCTCCCACTACGGGTGTGACCCCCGAGGCCGCCTAGACCACCCTCGCCTAG
90 92 94 96 98 100 102 104 106 108 110 112 1 2 3 4 5 6 7 8 9 10 11
P I E F T V N E D G Q V T V D G E A T E G D A H T G G S G G S G G S G S
SpyCatcher
3,640 3,660 3,680 3,700 3,720 3,740

CTACCCATACGATGTTCCAGATTACGCTCAAACATCCTCACCCTCTACAGAAGCATCAGTATCAACCTCTAGTACCTCTTCCTCTGCTTCACAATCATCTGACCCAA
GATGGGTATGCTACAAGGTCTAATGCGAGTTTGTAGGAGTGGGAGATGTCTTCGTAGTCATAGTTGGAGATCATGGAGAAGGAGACGAAGTGTTAGTAGACTGGGTT
1 2 3 4 5 6 7 8 9 2 4 6 8 10 12 14 16 18 20 22 24 26
Y P Y D V P D Y A Q T S S P S T E A S V S T S S T S S S A S Q S S D P
HA tag | 649 stalk
3,760 3,780 3,800 3,820 3,840

CTACAACATCTTCGTCCAGTTCATCGTCTTCCCCATCTTCCCAATCTGAGGAAATTTCATCATCACCGACGGTCTCAACTACACCATCAACTTCTTCATCATCTTCC
GATGTTGTAGAAGCAGGTCAAGTAGCAGAAGGGGTAGAAGGGTTAGACTCCTTTAAAGTAGTAGTGGCTGCCAGAGTTGATGTGGTAGTTGAAGAAGTAGTAGAAGG
28 30 32 34 36 38 40 42 44 46 48 50 52 54 56 58 60 62
T T T S S S S S S S S P S S Q S E E I S S S P T V S T T P S T S S S S S
649 stalk
3,860 3,880 3,900 3,920 3,940

TCAATGACTTCAACCACCACAACAAAGTCAATCTCAACTTCCACTACAAGTTCAGCTCCAGTTACAGATGTGACAGTTTCCTCATCGCCTAGTAAATCTACCTCTAC
AGTTACTGAAGTTGGTGGTGTTGTTTCAGTTAGAGTTGAAGGTGATGTTCAAGTCGAGGTCAATGTCTACACTGTCAAAGGAGTAGCGGATCATTTAGATGGAGATG
64 66 68 70 72 74 76 78 80 82 84 86 88 90 92 94 96 98
S M T S T T T T K S I S T S T T S S A P V T D V T V S S S P S K S T S T
649 stalk
3,960 3,980 4,000 4,020 4,040 4,060

TTCGACAAGTACAGAAACATCTAAAACACCTACTTCAATGACAGAGTATACATCTAGTACATCGATAATTTCGACTCCAGTTAGTCACTCGCAGACAGGTTTGTCGG
AAGCTGTTCATGTCTTTGTAGATTTTGTGGATGAAGTTACTGTCTCATATGTAGATCATGTAGCTATTAAAGCTGAGGTCAATCAGTGAGCGTCTGTCCAAACAGCC
100 102 104 106 108 110 112 114 116 118 120 122 124 126 128 130 132
S T S T E T S K T P T S M T E Y T S S T S I I S T P V S H S Q T G L S
649 stalk
4,080 4,100 4,120 4,140 4,160

CTTCATCAAGTTCATCATCTACAACATCCGGTTCTTCGTCCACTAAATCAGAAAGTTCGACAACATCTGGCTCTTCCCAGTCCGTGGAATCAACCTCCAGCCACGCC
GAAGTAGTTCAAGTAGTAGATGTTGTAGGCCAAGAAGCAGGTGATTTAGTCTTTCAAGCTGTTGTAGACCGAGAAGGGTCAGGCACCTTAGTTGGAGGTCGGTGCGG
134 136 138 140 142 144 146 148 150 152 154 156 158 160 162 164 166 168
A S S S S S S T T S G S S S T K S E S S T T S G S S Q S V E S T S S H A
649 stalk
4,180 4,200 4,220 4,240 4,260 4,280

ACTGTTCTTGCTAATTCCGCAGAAATGGTCACAACATCCTCTAGTTCATCCTCAACATCCGAAATGTCATTAACTAGTACTGCTACCAGTGTACCAGTCTCATCTAG
TGACAAGAACGATTAAGGCGTCTTTACCAGTGTTGTAGGAGATCAAGTAGGAGTTGTAGGCTTTACAGTAATTGATCATGACGATGGTCACATGGTCAGAGTAGATC
170 172 174 176 178 180 182 184 186 188 190 192 194 196 198 200 202 204
T V L A N S A E M V T T S S S S S S T S E M S L T S T A T S V P V S S S
649 stalk
4,300 4,320 4,340 4,360 4,380

TAGCAGTACGACATATTCTACTAGCGCATCTACACAAGCCGTCACTACAACATCTTCTTCCACTGTATCTACAACTTCTTCTAGTACAACGTTAACAAGCGCATTCA
ATCGTCATGCTGTATAAGATGATCGCGTAGATGTGTTCGGCAGTGATGTTGTAGAAGAAGGTGACATAGATGTTGAAGAAGATCATGTTGCAATTGTTCGCGTAAGT
206 208 210 212 214 216 218 220 222 224 226 228 230 232 234 236 238 240
S S T T Y S T S A S T Q A V T T T S S S T V S T T S S S T T L T S A F
649 stalk
4,400 4,420 4,440 4,460 4,480

CACATTCTTCAACCACATCGTCCGACCAGCCACCCAGCGACACTACAAGTCCATCTACGACACACGAACCTCATGTAACCACTCAGACGTCATCAGAAACATCTTCT
GTGTAAGAAGTTGGTGTAGCAGGCTGGTCGGTGGGTCGCTGTGATGTTCAGGTAGATGCTGTGTGCTTGGAGTACATTGGTGAGTCTGCAGTAGTCTTTGTAGAAGA
242 244 246 248 250 252 254 256 258 260 262 264 266 268 270 272 274 276
T H S S T T S S D Q P P S D T T S P S T T H E P H V T T Q T S S E T S S
649 stalk
4,500 4,520 4,540 4,560 4,580 4,600

TCTAAGTCATCTTCTACTTCTTCTTCAAGTACATCTCAAACCTCTGAGTCTGCAACACCATCCGATTCCGTATCACCTGGAAGTTCTACATCAACATCTTCTAGTAG
AGATTCAGTAGAAGATGAAGAAGAAGTTCATGTAGAGTTTGGAGACTCAGACGTTGTGGTAGGCTAAGGCATAGTGGACCTTCAAGATGTAGTTGTAGAAGATCATC
278 280 282 284 286 288 290 292 294 296 298 300 302 304 306 308 310 312
S K S S S T S S S S T S Q T S E S A T P S D S V S P G S S T S T S S S S
649 stalk
4,620 4,640 4,660 4,680 4,700

```
CACTTCTACTTCCACTTCTATTTCCAGTGGAGAAACGACAACTTCTTCTTCTTCATCATCTGCCACGACCACTTCTAACAGCGCAACCTTGTCAGTCTCTACCACAC
GTGAAGATGAAGGTGAAGATAAAGGTCACCTCTTTGCTGTTGAAGAAGAAGAAGTAGTAGACGGTGCTGGTGAAGATTGTCGCGTTGGAACAGTCAGAGATGGTGTG
314 316 318 320 322 324 326 328 330 332 334 336 338 340 342 344 346
T S T S T S I S S G E T T T S S S S S S A T T T S N S A T L S V S T T
649 stalk
4,720 4,740 4,760 4,780 4,800
```

```
AAACTTCGATTGAAGCCAGTTCATCTACTACATCTACATCTAGTTCAACAATTACAACTTCAAGTAGTAGCGCTCACATATCGTCGAAATCTCAATCTAGTATTACC
TTTGAAGCTAACTTCGGTCAAGTAGATGATGTAGATGTAGATCAAGTTGTTAATGTTGAAGTTCATCATCGCGAGTGTATAGCAGCTTTAGAGTTAGATCATAATGG
348 350 352 354 356 358 360 362 364 366 368 370 372 374 376 378 380 382
Q T S I E A S S S T T S T S S S T I T T S S S S A H I S S K S Q S S I T
649 stalk
4,820 4,840 4,860 4,880 4,900 4,920
```

```
TATCCCTCTTCCTCGACATCTTCATCTACATCGTCCTCAATTTCTAGCGAATCTGAAAGTTTTGAATCGACATCAGCAGAAGATGCTCCATCAACAGCACCTTCATC
ATAGGGAGAAGGAGCTGTAGAAGTAGATGTAGCAGGAGTTAAAGATCGCTTAGACTTTCAAAACTTAGCTGTAGTCGTCTTCTACGAGGTAGTTGTCGTGGAAGTAG
384 386 388 390 392 394 396 398 400 402 404 406 408 410 412 414 416 418
Y P S S S T S S S T S S S I S S E S E S F E S T S A E D A P S T A P S S
649 stalk
4,940 4,960 4,980 5,000 5,020
```

```
AAGTGTCTCTTCTAAGAGTTCTACCTCTACAACATCAAGCACATCGACATCTTCAAGCACTCCATCTCCATCACCATCTTCCGTGAGTTCTTCCTCCACCAGCTCAT
TTCACAGAGAAGATTCTCAAGATGGAGATGTTGTAGTTCGTGTAGCTGTAGAAGTTCGTGAGGTAGAGGTAGTGGTAGAAGGCACTCAAGAAGGAGGTGGTCGAGTA
420 422 424 426 428 430 432 434 436 438 440 442 444 446 448 450 452 454
S V S S K S S T S T T S S T S T S S S T P S P S P S S V S S S S T S S
649 stalk
5,040 5,060 5,080 5,100 5,120
```

```
TGACAACTTCTGCTGTATCAACACCAGCTACCTCTCATTCTCAAAGTACTGTAGTAACCACCACTACTATTACTACATCAACAGGTCCAGTGATGTCTACGACAACA
ACTGTTGAAGACGACATAGTTGTGGTCGATGGAGAGTAAGAGTTTCATGACATCATTGGTGGTGATGATAATGATGTAGTTGTCCAGGTCACTACAGATGCTGTTGT
456 458 460 462 464 466 468 470 472 474 476 478 480 482 484 486 488 490
L T T S A V S T P A T S H S Q S T V V T T T T I T T S T G P V M S T T T
649 stalk
5,140 5,160 5,180 5,200 5,220 5,240
```

```
GCTTATTCTTCTAGTTCTACTAGCAGCTCGGAATCTTCTGAGGTTCAGTCTGTCATGTCATCTACGCCTAGTTCAACATCAACAACAACCAGTTCGGAATCTACTTC
CGAATAAGAAGATCAAGATGATCGTCGAGCCTTAGAAGACTCCAAGTCAGACAGTACAGTAGATGCGGATCAAGTTGTAGTTGTTGTTGGTCAAGCCTTAGATGAAG
492 494 496 498 500 502 504 506 508 510 512 514 516 518 520 522 524 526
A Y S S S S T S S S E S S E V Q S V M S S T P S S T S T T T S S E S T S
649 stalk
5,260 5,280 5,300 5,320 5,340
```

ATCTAGCTCCACAGCTTCTACCTCACCATCAACCTCGCAAACTTTCGAAACTTCTCCTACTATAGGAGGTGTCCCCTCAACCACTTCATTTGTCTCTACGCCAACAA
TAGATCGAGGTGTCGAAGATGGAGTGGTAGTTGGAGCGTTTGAAAGCTTTGAAGAGGATGATATCCTCCACAGGGGAGTTGGTGAAGTAAACAGAGATGCGGTTGTT
528 530 532 534 536 538 540 542 544 546 548 550 552 554 556 558 560
S S S T A S T S P S T S Q T F E T S P T I G G V P S T T S F V S T P T
649 stalk
5,360 5,380 5,400 5,420 5,440

CGAAATTGTCGCACACTACTTCCACTATGACAGCACAGTCCGATAGTAAGTCTACCCACTCCTCAAGCACATCGACAGAAGATAAATCATCCACTGCTTCTGCAGTT
GCTTTAACAGCGTGTGATGAAGGTGATACTGTCGTGTCAGGCTATCATTCAGATGGGTGAGGAGTTCGTGTAGCTGTCTTCTATTTAGTAGGTGACGAAGACGTCAA
562 564 566 568 570 572 574 576 578 580 582 584 586 588 590 592 594 596
T K L S H T T S T M T A Q S D S K S T H S S S T S T E D K S S T A S A V
649 stalk
5,460 5,480 5,500 5,520 5,540 5,560

GACGAAAGCACTACAACATCCACTTCCACGGAGTCTACTACATCAGTAACATCAGGCACCTCCCATTCCGCTAAAGAATCTTCGTCAAATTCTAAGGTGTATAGTTC
CTGCTTTCGTGATGTTGTAGGTGAAGGTGCCTCAGATGATGTAGTCATTGTAGTCCGTGGAGGGTAAGGCGATTTCTTAGAAGCAGTTTAAGATTCCACATATCAAG
598 600 602 604 606 608 610 612 614 616 618 620 622 624 626 628 630 632
D E S T T T S T S T E S T T S V T S G T S H S A K E S S S N S K V Y S S
649 stalk
5,580 5,600 5,620 5,640 5,660

ACAGACAGCACACTCATCCATAAGTGTTGCATCATCACCTAGTACAAAGGGCGCCCAAATCCAATCTTCTATGGTTGAAATCTCTACCTACGCTGGTTCTGCTAACT
TGTCTGTCGTGTGAGTAGGTATTCACAACGTAGTAGTGGATCATGTTTCCCGCGGGTTTAGGTTAGAAGATACCAACTTTAGAGATGGATGCGACCAAGACGATTGA
634 636 638 640 642 644 646 648 650 652 654 656 658 660 662 664 666 668
Q T A H S S I S V A S S P S T K G A Q I Q S S M V E I S T Y A G S A N
649 stalk GPI anchor
5,680 5,700 5,720 5,740 5,760

CTGTTAACGCTGGTGCTGGTGCTGGTGCTTTGTTCTTGTTGTTGTCTTTGGCTATCATCTAATGATTAATTAACTCGAGATCTGATAACAACAGTGTAGATGTAACA
GACAATTGCGACCACGACCACGACCACGAAACAAGAACAACAACAGAAACCGATAGTAGATTACTAATTAATTGAGCTCTAGACTATTGTTGTCACATCTACATTGT
670 672 674 676 678 680 682 684 686 688 1
S V N A G A G A G A L F L L L S L A I I *
GPI anchor alpha fact...erminator
5,780 5,800 5,820 5,840 5,860 5,880

AAATCGACTTTGTTCCCACTGTACTTTTAGCTCGTACAAAATACAATATACTTTTCATTTCTCCGTAAACAACATGTTTTCCCATGTAATATCCTTTTCTATTTTTC
TTTAGCTGAAACAAGGGTGACATGAAAATCGAGCATGTTTTATGTTATATGAAAAGTAAAGAGGCATTTGTTGTACAAAAGGGTACATTATAGGAAAAGATAAAAAG
alpha factor terminator
5,900 5,920 5,940 5,960 5,980

GTTCCGTTACCAACTTTACACATACTTTATATAGCTATTCACTTCTATACACTAAAAAACTAAGACAATTTTAATTTTGCTGCCTGCCATATTTCAATTTGTTATAA
CAAGGCAATGGTTGAAATGTGTATGAAATATATCGATAAGTGAAGATATGTGATTTTTTGATTCTGTTAAAATTAAAACGACGGACGGTATAAAGTTAACAATATT
alpha factor terminator
6,000 6,020 6,040 6,060 6,080

ATTCCTATAATTTATCCTATTAGTAGCTAAAAAAAGATGAATGTGAATCGAATCCTAAGAGAATTcgaattgggtaccgggccccccctcgaggtcgacggtatcga
TAAGGATATTAAATAGGATAATCATCGATTTTTTTCTACTTACACTTAGCTTAGGATTCTCTTAAgcttaacccatggcccggggggggagctccagctgccatagct

alpha factor terminator

6,100 6,120 6,140 6,160 6,180 6,200

taagcttgatatcgaattcctgcagcccgggggatccactagttctagagcggccgccaccgcggtggagctccagcttttgttccctttagtgagggttaattgcg
attcgaactatagcttaaggacgtcgggccccctaggtgatcaagatctcgccggcggtggcgccacctcgaggtcgaaaacaagggaaatcactcccaattaacgc

6,220 6,240 6,260 6,280 6,300

cgcttggcgtaatcatggtcatagctgtttcctgtgtgaaattgttatccgctcacaattccacacaacataggagccggaagcataaagtgtaaagcctggggtgc
gcgaaccgcattagtaccagtatcgacaaaggacacactttaacaataggcgagtgttaaggtgtgttgtatcctcggccttcgtatttcacatttcggaccccacg

LacO

6,320 6,340 6,360 6,380 6,400 6,420

ctaatgagtgaggtaactcacattaattgcgttgcgctcactgcccgctttccagtcgggaaacctgtcgtgccagctgcattaatgaatcggccaacgcgcgggga
gattactcactccattgagtgtaattaacgcaacgcgagtgacgggcgaaaggtcagccctttggacagcacggtcgacgtaattacttagccggttgcgcgcccct

6,440 6,460 6,480 6,500 6,520

gaggcggtttgcgtattgggcgctcttccgcttcctcgctcactgactcgctgcgctcggtcgttcggctgcggcgagcggtatcagctcactcaaaggcggtaata
ctccgccaaacgcataacccgcgagaaggcgaaggagcgagtgactgagcgacgcgagccagcaagccgacgccgctcgccatagtcgagtgagtttccgccattat

6,540 6,560 6,580 6,600 6,620

cggttatccacagaatcaggggataacgcaggaaagaacatgtgagcaaaaggccagcaaaaggccaggaaccgtaaaaaggccgcgttgctggcgtttttccatag
gccaataggtgtcttagtcccctattgcgtcctttcttgtacactcgttttccggtcgttttccggtccttggcatttttccggcgcaacgaccgcaaaaaggtatc

ColE1 origin

6,640 6,660 6,680 6,700 6,720 6,740

gctccgcccccctgacgagcatcacaaaaatcgacgctcaagtcagaggtggcgaaacccgacaggactataaagataccaggcgtttccccctggaagctccctcg
cgaggcggggggactgctcgtagtgtttttagctgcgagttcagtctccaccgctttgggctgtcctgatatttctatggtccgcaaagggggaccttcgagggagc

ColE1 origin

6,760 6,780 6,800 6,820 6,840

tgcgctctcctgttccgaccctgccgcttaccggatacctgtccgcctttctcccttcgggaagcgtggcgctttctcatagctcacgctgtaggtatctcagttcg
acgcgagaggacaaggctgggacggcgaatggcctatggacaggcggaaagagggaagcccttcgcaccgcgaaagagtatcgagtgcgacatccatagagtcaagc

ColE1 origin

6,860 6,880 6,900 6,920 6,940

gtgtaggtcgttcgctccaagctgggctgtgtgcacgaaccccccgttcagcccgaccgctgcgccttatccggtaactatcgtcttgagtccaacccggtaagaca
cacatccagcaagcgaggttcgacccgacacacgtgcttggggggcaagtcgggctggcgacgcggaataggccattgatagcagaactcaggttgggccattctgt

ColE1 origin

6,960 6,980 7,000 7,020 7,040 7,060

cgacttatcgccactggcagcagccactggtaacaggattagcagagcgaggtatgtaggcggtgctacagagttcttgaagtggtggcctaactacggctacacta
gctgaatagcggtgaccgtcgtcggtgaccattgtcctaatcgtctcgctccatacatccgccacgatgtctcaagaacttcaccaccggattgatgccgatgtgat

ColE1 origin

7,080 7,100 7,120 7,140 7,160

gaaggacagtatttggtatctgcgctctgctgaagccagttaccttcggaaaaagagttggtagctcttgatccggcaaacaaaccaccgctggtagcggtggtttt
cttcctgtcataaaccatagacgcgagacgacttcggtcaatggaagcctttttctcaaccatcgagaactaggccgtttgtttggtggcgaccatcgccaccaaaa

ColE1 origin

7,180 7,200 7,220 7,240 7,260

tttgtttgcaagcagcagattacgcgcagaaaaaaaggatctcaagaagatcctttgatcttttctacggggtctgacgctcagtggaacgaaaactcacgttaagg
aaacaaacgttcgtcgtctaatgcgcgtcttttttttcctagagttcttctaggaaactagaaaagatgccccagactgcgagtcaccttgcttttgagtgcaattcc

ColE1 origin

7,280 7,300 7,320 7,340 7,360 7,380

gattttggtcatgagattatcaaaaaggatcttcacctagatccttttaaattaaaaatgaagttttaaatcaatctaaagtatatatgagtaaacttggtctgaca
ctaaaaccagtactctaatagtttttcctagaagtggatctaggaaaatttaatttttacttcaaaatttagttagatttcatatatactcatttgaaccagactgt

ColE...gin

7,400 7,420 7,440 7,460 7,480

gttaccaatgcttaatcagtgaggcacctatctcagcgatctgtctatttcgttcatccatagttgcctgactccccgtcgtgtagataactacgatacgggagggc
caatggttacgaattagtcactccgtggatagagtcgctagacagataaagcaagtaggtatcaacggactgaggggcagcacatctattgatgctatgccctcccg

AmpR

7,500 7,520 7,540 7,560 7,580

ttaccatctggccccagtgctgcaatgataccgcgagacccacgctcaccggctccagatttatcagcaataaaccagccagccggaagggccgagcgcagaagtgg
aatggtagaccggggtcacgacgttactatggcgctctgggtgcgagtggccgaggtctaaatagtcgttatttggtcggtcggccttcccggctcgcgtcttcacc

AmpR

7,600 7,620 7,640 7,660 7,680 7,700

tcctgcaactttatccgcctccatccagtctattaattgttgccgggaagctagagtaagtagttcgccagttaatagtttgcgcaacgttgttgccattgctacag
aggacgttgaaataggcggaggtaggtcagataattaacaacggcccttcgatctcattcatcaagcggtcaattatcaaacgcgttgcaacaacggtaacgatgtc

AmpR

7,720 7,740 7,760 7,780 7,800

gcatcgtggtgtcacgctcgtcgtttggtatggcttcattcagctccggttcccaacgatcaaggcgagttacatgatcccccatgttgtgcaaaaaagcggttagc
cgtagcaccacagtgcgagcagcaaaccataccgaagtaagtcgaggccaagggttgctagttccgctcaatgtactagggggtacaacacgttttttcgccaatcg

AmpR

7,820 7,840 7,860 7,880 7,900

tccttcggtcctccgatcgttgtcagaagtaagttggccgcagtgttatcactcatggttatggcagcactgcataattctcttactgtcatgccatccgtaagatg
aggaagccaggaggctagcaacagtcttcattcaaccggcgtcacaatagtgagtaccaataccgtcgtgacgtattaagagaatgacagtacggtaggcattctac

AmpR

7,920 7,940 7,960 7,980 8,000 8,020

cttttctgtgactggtgagtactcaaccaagtcattctgagaatagtgtatgcggcgaccgagttgctcttgcccggcgtcaatacgggataataccgcgccacata
gaaaagacactgaccactcatgagttggttcagtaagactcttatcacatacgccgctggctcaacgagaacgggccgcagttatgccctattatggcgcggtgtat

AmpR

8,040 8,060 8,080 8,100 8,120

gcagaactttaaaagtgctcatcattggaaaacgttcttcggggcgaaaactctcaaggatcttaccgctgttgagatccagttcgatgtaacccactcgtgcaccc
cgtcttgaaattttcacgagtagtaaccttttgcaagaagccccgcttttgagagttcctagaatggcgacaactctaggtcaagctacattgggtgagcacgtggg

AmpR

8,140 8,160 8,180 8,200 8,220

aactgatcttcagcatcttttactttcaccagcgtttctgggtgagcaaaaacaggaaggcaaaatgccgcaaaaaagggaataagggcgacacggaaatgttgaat
ttgactagaagtcgtagaaaatgaaagtggtcgcaaagacccactcgtttttgtccttccgttttacggcgttttttcccttattcccgctgtgcctttacaactta

8,240 8,260 8,280 8,300 8,320 8,340

actcatactcttcctttttcaatattattgaagcatttatcagggttattgtctcatgagcggatacatatttgaatgtatttagaaaaataaacaaataggggttc
tgagtatgagaaggaaaaagttataataacttcgtaaatagtcccaataacagagtactcgcctatgtataaacttacataaatctttttatttgtttatccccaag

8,360 8,380 8,400 8,420 8,440

cgcgcacatttccccgaaaagtgccacctgacgtctaagaaaccattattatcatgacattaacctataaaaataggcgtatcacgaggccctttcgtc
gcgcgtgtaaaggggcttttcacggtggactgcagattctttggtaataatagtactgtaattggatatttttatccgcatagtgctccgggaaagcag

8,460 8,470 8,480 8,490 8,500 8,510 8,520 8,530 8,540 8,550

ST/SC ligation / pAnchor-strep (pRS403) (8549 bp)

tcgcgcgtttcggtgatgacggtgaaaacctctgacacatgcagctcccggagacggtcacagcttgtctgtaagcggatgccgggagcagacaagcccgtcagggc
agcgcgcaaagccactactgccacttttggagactgtgtacgtcgagggcctctgccagtgtcgaacagacattcgcctacggccctcgtctgttcgggcagtcccg

20 40 60 80 100

gcgtcagcgggtgttggcgggtgtcggggctggcttaactatgcggcatcagagcagattgtactgagagtgcaccatatcccgttttaagagcttggtgagcg
cgcagtcgcccacaaccgcccacagccccgaccgaattgatacgccgtagtctcgtctaacatgactctcacgtggtatttaagggcaaaattctcgaaccactcgc

HIS3

120 140 160 180 200

ctaggagtcactgccaggtatcgtttgaacacggcattagtcagggaagtcataacacagtcctttcccgcaatttttctttttctattactcttggcctcctctagt
gatcctcagtgacggtccatagcaaacttgtgccgtaatcagtcccttcagtattgtgtcaggaaagggcgttaaaagaaaaagataatgagaaccggaggagatca

HIS3

220 240 260 280 300 320

acactctatatttttttatgcctcggtaatgattttcattttttttttttcccctagcggatgactcttttttttttcttagcgattggcattatcacataatgaatta
tgtgagatataaaaaaatacggagccattactaaaagtaaaaaaaaaaaggggatcgcctactgagaaaaaaaaaagaatcgctaaccgtaatagtgtattacttaat

HIS3

340 360 380 400 420

tacattatataaagtaatgtgatttcttcgaagaatatactaaaaaatgagcaggcaagataaacgaaggcaaagatgacagagcagaaagccctagtaaagcgtat
atgtaatatatttcattacactaaagaagcttcttatatgattttttactcgtccgttctatttgcttccgtttctactgtctcgtctttcgggatcatttcgcata

HIS3

440 460 480 500 520

tacaaatgaaaccaagattcagattgcgatctctttaaagggtggtcccctagcgatagagcactcgatcttcccagaaaaagaggcagaagcagtagcagaacagg
atgtttactttggttctaagtctaacgctagagaaatttcccaccaggggatcgctatctcgtgagctagaagggtctttttctccgtcttcgtcatcgtcttgtcc

HIS3

540 560 580 600 620 640

ccacacaatcgcaagtgattaacgtccacacaggtatagggtttctggaccatatgatacatgctctggccaagcattccggctggtcgctaatcgttgagtgcatt
ggtgtgttagcgttcactaattgcaggtgtgtccatatcccaaagacctggtatactatgtacgagaccggttcgtaaggccgaccagcgattagcaactcacgtaa

HIS3

660 680 700 720 740

ggtgacttacacatagacgaccatcacaccactgaagactgcgggattgctctcggtcaagcttttaaagaggccctactggcgcgtggagtaaaaaggtttggatc
ccactgaatgtgtatctgctggtagtgtggtgacttctgacgccctaacgagagccagttcgaaaatttctccgggatgaccgcgcacctcatttttccaaacctag

HIS3

760 780 800 820 840

```
aggatttgcgcctttggatgaggcactttccagagcggtggtagatctttcgaacaggccgtacgcagttgtcgaacttggtttgcaaagggagaaagtaggagatc
tcctaaacgcggaaacctactccgtgaaaggtctcgccaccatctagaaagcttgtccggcatgcgtcaacagcttgaaccaaacgtttccctctttcatcctctag
HIS3
860 880 900 920 940 960

tctcttgcgagatgatcccgcatttcttgaaagctttgcagaggctagcagaattaccctccacgttgattgtctgcgaggcaagaatgatcatcaccgtagtgag
agagaacgctctactagggcgtaaagaactttcgaaacgtctccgatcgtcttaatgggaggtgcaactaacagacgctccgttcttactagtagtggcatcactc
HIS3
980 1,000 1,020 1,040 1,060

agtgcgttcaaggctcttgcggttgccataagagaagccacctcgcccaatggtaccaacgatgttccctccaccaaaggtgttcttatgtagtgacaccgattatt
tcacgcaagttccgagaacgccaacggtattctcttcggtggagcgggttaccatggttgctacaagggaggtggtttccacaagaatacatcactgtggctaataa
HIS3
1,080 1,100 1,120 1,140 1,160

taaagctgcagcatacgatatatatacatgtgtatatatgtatacctatgaatgtcagtaagtatgtatacgaacagtatgatactgaagatgacaaggtaatgcat
atttcgacgtcgtatgctatatatatgtacacatatatacatatggatacttacagtcattcatacatatgcttgtcatactatgacttctactgttccattacgta
HIS3
1,180 1,200 1,220 1,240 1,260 1,280

cattctatacgtgtcattctgaacgaggcgcgctttccttttttcttttttgcttttttcttttttttttctcttgaactcgacggatctatgcggtgtgaaataccgca
gtaagatatgcacagtaagacttgctccgcgcgaaaggaaaaaagaaaaacgaaaaagaaaaaaaaagagaacttgagctgcctagatacgccacactttatggcgt
HIS3
1,300 1,320 1,340 1,360 1,380

cagatgcgtaaggagaaaataccgcatcaggaaattgtaaacgttaatattttgttaaaattcgcgttaaatttttgttaaatcagctcattttttaaccaataggc
gtctacgcattcctcttttatggcgtagtcctttaacatttgcaattataaaacaattttaagcgcaatttaaaaacaatttagtcgagtaaaaaattggttatccg
1,400 1,420 1,440 1,460 1,480

cgaaatcggcaaaatcccttataaatcaaaagaatagaccgagatagggttgagtgttgttccagtttggaacaagagtccactattaaagaacgtggactccaacg
gctttagccgttttagggaatatttagttttcttatctggctctatcccaactcacaacaaggtcaaaccttgttctcaggtgataatttcttgcacctgaggttgc
F1 ori
1,500 1,520 1,540 1,560 1,580 1,600

tcaaagggcgaaaaaccgtctatcagggcgatggcccactacgtgaaccatcaccctaatcaagttttttggggtcgaggtgccgtaaagcactaaatcggaaccct
agtttcccgctttttggcagatagtcccgctaccgggtgatgcacttggtagtgggattagttcaaaaaaccccagctccacggcatttcgtgatttagccttggga
F1 ori
1,620 1,640 1,660 1,680 1,700

aaagggagcccccgatttagagcttgacggggaaagccggcgaacgtggcgagaaaggaagggaagaaagcgaaaggagcgggcgctagggcgctggcaagtgtagc
tttccctcgggggctaaatctcgaactgcccctttcggccgcttgcaccgctctttccttcccttctttcgctttcctcgcccgcgatcccgcgaccgttcacatcg
F1 ori
1,720 1,740 1,760 1,780 1,800
```

ggtcacgctgcgcgtaaccaccacacccgccgcgcttaatgcgccgctacagggcgcgtcgcgccattcgccattcaggctgcgcaactgttgggaagggcgatcgg
ccagtgcgacgcgcattggtggtgtgggcggcgcgaattacgcggcgatgtcccgcgcagcgcggtaagcggtaagtccgacgcgttgacaacccttcccgctagcc

F1 ori LacZ alpha

1,820 1,840 1,860 1,880 1,900 1,920

tgcgggcctcttcgctattacgccagctggcgaaagggggatgtgctgcaaggcgattaagttgggtaacgccagggttttcccagtcacgacgttgtaaaacgacg
acgcccggagaagcgataatgcggtcgaccgctttccccctacacgacgttccgctaattcaacccattgcggtcccaaaagggtcagtgctgcaacattttgctgc

LacZ alpha

1,940 1,960 1,980 2,000 2,020

gccagtgagcgcgcAAGCTTACCAGTTCTCACACGGAACACCACTAATGGACACAAATTCGAAATACTTTGACCCTATTTTCGAGGACCTTGTCACCTTGAGCCCAA
cggtcactcgcgcgTTCGAATGGTCAAGAGTGTGCCTTGTGGTGATTACCTGTGTTTAAGCTTTATGAAACTGGGATAAAAGCTCCTGGAACAGTGGAACTCGGGTT

GPD promoter

2,040 2,060 2,080 2,100 2,120 2,140

GAGAGCCAAGATTTAAATTTTCCTATGACTTGATGCAAATTCCCAAAGCTAATAACATGCAAGACACGTACGGTCAAGAAGACATATTTGACCTCTTAACAGGTTCA
CTCTCGGTTCTAAATTTAAAAGGATACTGAACTACGTTTAAGGGTTTCGATTATTGTACGTTCTGTGCATGCCAGTTCTTCTGTATAAACTGGAGAATTGTCCAAGT

GPD promoter

2,160 2,180 2,200 2,220 2,240

GACGCGACTGCCTCATCAGTAAGACCCGTTGAAAAGAACTTACCTGAAAAAAACGAATATATACTAGCGTTGAATGTTAGCGTCAACAACAAGAAGTTTAATGACGC
CTGCGCTGACGGAGTAGTCATTCTGGGCAACTTTTCTTGAATGGACTTTTTTTGCTTATATATGATCGCAACTTACAATCGCAGTTGTTGTTCTTCAAATTACTGCG

GPD promoter

2,260 2,280 2,300 2,320 2,340

GGAGGCCAAGGCAAAAAGATTCCTTGATTACGTAAGGGAGTTAGAATCATTTTGAATAAAAAACACGCTTTTTCAGTTCGAGTTTATCATTATCAATACTGCCATTT
CCTCCGGTTCCGTTTTTCTAAGGAACTAATGCATTCCCTCAATCTTAGTAAAACTTATTTTTTGTGCGAAAAAGTCAAGCTCAAATAGTAATAGTTATGACGGTAAA

GPD promoter

2,360 2,380 2,400 2,420 2,440 2,460

CAAAGAATACGTAAATAATTAATAGTAGTGATTTTCCTAACTTTATTTAGTCAAAAAATTAGCCTTTTAATTCTGCTGTAACCCGTACATGCCCAAAATAGGGGGCG
GTTTCTTATGCATTTATTAATTATCATCACTAAAAGGATTGAAATAAATCAGTTTTTTAATCGGAAAATTAAGACGACATTGGGCATGTACGGGTTTTATCCCCCGC

GPD promoter

2,480 2,500 2,520 2,540 2,560

GGTTACACAGAATATATAACATCGTAGGTGTCTGGGTGAACAGTTTATTCCTGGCATCCACTAAATATAATGGAGCCCGCTTTTTAAGCTGGCATCCAGAAAAAAAA
CCAATGTGTCTTATATATTGTAGCATCCACAGACCCACTTGTCAAATAAGGACCGTAGGTGATTTATATTACCTCGGGCGAAAAATTCGACCGTAGGTCTTTTTTTT

GPD promoter

2,580 2,600 2,620 2,640 2,660

AGAATCCCAGCACCAAAATATTGTTTTCTTCACCAACCATCAGTTCATAGGTCCATTCTCTTAGCGCAACTACAGAGAACAGGGGCACAAACAGGCAAAAAACGGGC
TCTTAGGGTCGTGGTTTTATAACAAAAGAAGTGGTTGGTAGTCAAGTATCCAGGTAAGAGAATCGCGTTGATGTCTCTTGTCCCCGTGTTTGTCCGTTTTTTGCCCG
GPD promoter
2,680 2,700 2,720 2,740 2,760 2,780

ACAACCTCAATGGAGTGATGCAACCTGCCTGGAGTAAATGATGACACAAGGCAATTGACCCACGCATGTATCTATCTCATTTTCTTACACCTTCTATTACCTTCTGC
TGTTGGAGTTACCTCACTACGTTGGACGGACCTCATTTACTACTGTGTTCCGTTAACTGGGTGCGTACATAGATAGAGTAAAAGAATGTGGAAGATAATGGAAGACG
GPD promoter
2,800 2,820 2,840 2,860 2,880

TCTCTCTGATTTGGAAAAAGCTGAAAAAAAAAGGTTGAAACCAGTTCCCTGAAATTATTCCCCTACTTGACTAATAAGTATATAAAGACGGTAGGTATTGATTGTAAT
AGAGAGACTAAACCTTTTTCGACTTTTTTTTTCCAACTTTGGTCAAGGGACTTTAATAAGGGGATGAACTGATTATTCATATATTTCTGCCATCCATAACTAACATTA
GPD promoter
2,900 2,920 2,940 2,960 2,980

TCTGTAAATCTATTTCTTAAACTTCTTAAATTCTACTTTTATAGTTAGTCTTTTTTTTAGTTTTAAAACACCAAGAACTTAGTTTCGAATAAACACACATAAACAGA
AGACATTTAGATAAAGAATTTGAAGAATTTAAGATGAAAATATCAATCAGAAAAAAAATCAAAATTTTGTGGTTCTTGAATCAAAGCTTATTTGTGTGTATTTGTCT
GPD promoter
3,000 3,020 3,040 3,060 3,080 3,100

ATTCATGAGATTCCCATCTATCTTCACCGCTGTTTTGTTCGCTGCTTCTTCTGCTTTGGCTGCTCCAGCTAACACCACCACCGAAGACGAAACCGCTCAAATCCCAG
TAAGTACTCTAAGGGTAGATAGAAGTGGCGACAAAACAAGCGACGAAGAAGACGAAACCGACGAGGTCGATTGTGGTGGTGGCTTCTGCTTTGGCGAGTTTAGGGTC
1 2 3 4 5 6 7 8 9 10 11 12 13 14 15 16 17 18 19 20 21 22 23 24 25 26 27 28 29 30 31 32 33 34
M R F P S I F T A V L F A A S S A L A A P A N T T T E D E T A Q I P
Mating factor alpha leader
3,120 3,140 3,160 3,180 3,200

CTGAAGCTGTTATCGACTACTCTGACTTGGAAGGTGACTTCGACGCTGCTGCTTTGCCATTGTCTAACTCTACCAACAACGGTTTGTCTTCTACCAACACCACCATC
GACTTCGACAATAGCTGATGAGACTGAACCTTCCACTGAAGCTGCGACGACGAAACGGTAACAGATTGAGATGGTTGTTGCCAAACAGAAGATGGTTGTGGTGGTAG
35 36 37 38 39 40 41 42 43 44 45 46 47 48 49 50 51 52 53 54 55 56 57 58 59 60 61 62 63 64 65 66 67 68 69 70
A E A V I D Y S D L E G D F D A A A L P L S N S T N N G L S S T N T T I
Mating factor alpha leader
3,220 3,240 3,260 3,280 3,300

GCTTCTATCGCTGCTAAGGAAGAAGGTGTTCAATTGGACAAGAGAGAAGCTAGCGCAGTAACTACCTTGTCTGGCTTATCAGGGGAACAGGGTCCTAGCGGTGACAT
CGAAGATAGCGACGATTCCTTCTTCCACAAGTTAACCTGTTCTCTCTTCGATCGCGTCATTGATGGAACAGACCGAATAGTCCCCTTGTCCCAGGATCGCCACTGTA
71 72 73 74 75 76 77 78 79 80 81 82 83 84 85 86 2 4 6 8 10 12 14 16
A S I A A K E E G V Q L D K R E V T T L S G L S G E Q G P S G D M
Mating factor alpha leader SpyCatcher
3,320 3,340 3,360 3,380 3,400 3,420

```
GACGACAGAGGAGGACAGTGCGACCCACATAAAGTTTAGCAAAAGAGATGAAGATGGAAGGGAACTAGCTGGAGCGACCATGGAACTTCGTGACAGCTCAGGGAAGA
CTGCTGTCTCCTCCTGTCACGCTGGGTGTATTTCAAATCGTTTTCTCTACTTCTACCTTCCCTTGATCGACCTCGCTGGTACCTTGAAGCACTGTCGAGTCCCTTCT
 18    20    22    24    26    28    30    32    34    36    38    40    42    44    46    48    50    52
 T  T  E  E  D  S  A  T  H  I  K  F  S  K  R  D  E  D  G  R  E  L  A  G  A  T  M  E  L  R  D  S  S  G  K
SpyCatcher
3,440    3,460    3,480    3,500    3,520

CTATATCAACGTGGATCTCAGATGGTCACGTCAAGGACTTCTATTTGTACCCCGGAAAGTACACATTTGTGGAAACCGCCGCGCCGGATGGCTACGAGGTAGCGACC
GATATAGTTGCACCTAGAGTCTACCAGTGCAGTTCCTGAAGATAAACATGGGGCCTTTCATGTGTAAACACCTTTGGCGGCGCGGCCTACCGATGCTCCATCGCTGG
 54    56    58    60    62    64    66    68    70    72    74    76    78    80    82    84    86    88
 T  I  S  T  W  I  S  D  G  H  V  K  D  F  Y  L  Y  P  G  K  Y  T  F  V  E  T  A  A  P  D  G  Y  E  V  A  T
SpyCatcher
3,540    3,560    3,580    3,600    3,620

CCGATAGAATTCACCGTTAACGAGGACGGCCAAGTCACCGTAGATGGAGAGGCGACAGAGGGTGATGCCCACACTGGGGGCTCCGGCGGATCTGGTGGGAGCGGATC
GGCTATCTTAAGTGGCAATTGCTCCTGCCGGTTCAGTGGCATCTACCTCTCCGCTGTCTCCCACTACGGGTGTGACCCCCGAGGCCGCCTAGACCACCCTCGCCTAG
 90    92    94    96    98    100   102   104   106   108   110   112   1  2  3  4  5  6  7  8  9  10 11
 P  I  E  F  T  V  N  E  D  G  Q  V  T  V  D  G  E  A  T  E  G  D  A  H  T  G  G  S  G  G  S  G  G  S  G  S
SpyCatcher
3,640    3,660    3,680    3,700    3,720    3,740

CTGGTCTCATCCACAATTTGAAAAACAAACATCCTCACCCTCTACAGAAGCATCAGTATCAACCTCTAGTACCTCTTCCTCTGCTTCACAATCATCTGACCCAACTA
GACCAGAGTAGGTGTTAAACTTTTTGTTTGTAGGAGTGGGAGATGTCTTCGTAGTCATAGTTGGAGATCATGGAGAAGGAGACGAAGTGTTAGTAGACTGGGTTGAT
 1  2  3  4  5  6  7  8     2     4     6     8     10    12    14    16    18    20    22    24    26
 W  S  H  P  Q  F  E  K  Q  T  S  S  P  S  T  E  A  S  V  S  T  S  S  T  S  S  S  A  S  Q  S  S  D  P  T
strep tag    649 stalk
3,760    3,780    3,800    3,820    3,840

CAACATCTTCGTCCAGTTCATCGTCTTCCCCATCTTCCCAATCTGAGGAAATTTCATCATCACCGACGGTCTCAACTACACCATCAACTTCTTCATCATCTTCCTCA
GTTGTAGAAGCAGGTCAAGTAGCAGAAGGGGTAGAAGGGTTAGACTCCTTTAAAGTAGTAGTGGCTGCCAGAGTTGATGTGGTAGTTGAAGAAGTAGTAGAAGGAGT
28    30    32    34    36    38    40    42    44    46    48    50    52    54    56    58    60    62
 T  T  S  S  S  S  S  S  S  S  P  S  S  Q  S  E  E  I  S  S  S  P  T  V  S  T  T  P  S  T  S  S  S  S  S  S
649 stalk
3,860    3,880    3,900    3,920    3,940

ATGACTTCAACCACCACAACAAAGTCAATCTCAACTTCCACTACAAGTTCAGCTCCAGTTACAGATGTGACAGTTTCCTCATCGCCTAGTAAATCTACCTCTACTTC
TACTGAAGTTGGTGGTGTTGTTTCAGTTAGAGTTGAAGGTGATGTTCAAGTCGAGGTCAATGTCTACACTGTCAAAGGAGTAGCGGATCATTTAGATGGAGATGAAG
 64    66    68    70    72    74    76    78    80    82    84    86    88    90    92    94    96    98
 M  T  S  T  T  T  T  K  S  I  S  T  S  T  T  S  S  A  P  V  T  D  V  T  V  S  S  S  P  S  K  S  T  S  T  S
649 stalk
3,960    3,980    4,000    4,020    4,040    4,060
```

```
GACAAGTACAGAAACATCTAAAACACCTACTTCAATGACAGAGTATACATCTAGTACATCGATAATTTCGACTCCAGTTAGTCACTCGCAGACAGGTTTGTCGGCTT
CTGTTCATGTCTTTGTAGATTTTGTGGATGAAGTTACTGTCTCATATGTAGATCATGTAGCTATTAAAGCTGAGGTCAATCAGTGAGCGTCTGTCCAAACAGCCGAA
 100    102    104    106    108    110    112    114    116    118    120    122    124    126    128    130    132    134
T S T E T S K T P T S M T E Y T S S T S I I S T P V S H S Q T G L S A
649 stalk
4,080 4,100 4,120 4,140 4,160
```

```
CATCAAGTTCATCATCTACAACATCCGGTTCTTCGTCCACTAAATCAGAAAGTTCGACAACATCTGGCTCTTCCCAGTCCGTGGAATCAACCTCCAGCCACGCCACT
GTAGTTCAAGTAGTAGATGTTGTAGGCCAAGAAGCAGGTGATTTAGTCTTTCAAGCTGTTGTAGACCGAGAAGGGTCAGGCACCTTAGTTGGAGGTCGGTGCGGTGA
 136    138    140    142    144    146    148    150    152    154    156    158    160    162    164    166    168    170
S S S S S S T T S G S S S T K S E S S T T S G S S Q S V E S T S S H A T
649 stalk
4,180 4,200 4,220 4,240 4,260 4,280
```

```
GTTCTTGCTAATTCCGCAGAAATGGTCACAACATCCTCTAGTTCATCCTCAACATCCGAAATGTCATTAACTAGTACTGCTACCAGTGTACCAGTCTCATCTAGTAG
CAAGAACGATTAAGGCGTCTTTACCAGTGTTGTAGGAGATCAAGTAGGAGTTGTAGGCTTTACAGTAATTGATCATGACGATGGTCACATGGTCAGAGTAGATCATC
 172    174    176    178    180    182    184    186    188    190    192    194    196    198    200    202    204    206
V L A N S A E M V T T S S S S S S T S E M S L T S T A T S V P V S S S S
649 stalk
4,300 4,320 4,340 4,360 4,380
```

```
CAGTACGACATATTCTACTAGCGCATCTACACAAGCCGTCACTACAACATCTTCTTCCACTGTATCTACAACTTCTTCTAGTACAACGTTAACAAGCGCATTCACAC
GTCATGCTGTATAAGATGATCGCGTAGATGTGTTCGGCAGTGATGTTGTAGAAGAAGGTGACATAGATGTTGAAGAAGATCATGTTGCAATTGTTCGCGTAAGTGTG
 208    210    212    214    216    218    220    222    224    226    228    230    232    234    236    238    240
S T T Y S T S A S T Q A V T T T S S S T V S T T S S S T T L T S A F T
649 stalk
4,400 4,420 4,440 4,460 4,480
```

```
ATTCTTCAACCACATCGTCCGACCAGCCACCCAGCGACACTACAAGTCCATCTACGACACACGAACCTCATGTAACCACTCAGACGTCATCAGAAACATCTTCTTCT
TAAGAAGTTGGTGTAGCAGGCTGGTCGGTGGGTCGCTGTGATGTTCAGGTAGATGCTGTGTGCTTGGAGTACATTGGTGAGTCTGCAGTAGTCTTTGTAGAAGAAGA
242    244    246    248    250    252    254    256    258    260    262    264    266    268    270    272    274    276
H S S T T S S D Q P P S D T T S P S T T H E P H V T T Q T S S E T S S S
649 stalk
4,500 4,520 4,540 4,560 4,580 4,600
```

```
AAGTCATCTTCTACTTCTTCTTCAAGTACATCTCAAACCTCTGAGTCTGCAACACCATCCGATTCCGTATCACCTGGAAGTTCTACATCAACATCTTCTAGTAGCAC
TTCAGTAGAAGATGAAGAAGAAGTTCATGTAGAGTTTGGAGACTCAGACGTTGTGGTAGGCTAAGGCATAGTGGACCTTCAAGATGTAGTTGTAGAAGATCATCGTG
278    280    282    284    286    288    290    292    294    296    298    300    302    304    306    308    310    312
K S S S T S S S S T S Q T S E S A T P S D S V S P G S S T S T S S S S T
649 stalk
4,620 4,640 4,660 4,680 4,700
```

TTCTACTTCCACTTCTATTTCCAGTGGAGAAACGACAACTTCTTCTTCTTCATCATCTGCCACGACCACTTCTAACAGCGCAACCTTGTCAGTCTCTACCACACAAA
AAGATGAAGGTGAAGATAAAGGTCACCTCTTTGCTGTTGAAGAAGAAGAAGTAGTAGACGGTGCTGGTGAAGATTGTCGCGTTGGAACAGTCAGAGATGGTGTGTTT
314 316 318 320 322 324 326 328 330 332 334 336 338 340 342 344 346 348
S T S T S I S S G E T T T S S S S S S A T T T S N S A T L S V S T T Q

649 stalk

4,720 4,740 4,760 4,780 4,800

CTTCGATTGAAGCCAGTTCATCTACTACATCTACATCTAGTTCAACAATTACAACTTCAAGTAGTAGCGCTCACATATCGTCGAAATCTCAATCTAGTATTACCTAT
GAAGCTAACTTCGGTCAAGTAGATGATGTAGATGTAGATCAAGTTGTTAATGTTGAAGTTCATCATCGCGAGTGTATAGCAGCTTTAGAGTTAGATCATAATGGATA
350 352 354 356 358 360 362 364 366 368 370 372 374 376 378 380 382 384
T S I E A S S S T T S T S S S T I T T S S S S A H I S S K S Q S S I T Y

649 stalk

4,820 4,840 4,860 4,880 4,900 4,920

CCCTCTTCCTCGACATCTTCATCTACATCGTCCTCAATTTCTAGCGAATCTGAAAGTTTTGAATCGACATCAGCAGAAGATGCTCCATCAACAGCACCTTCATCAAG
GGGAGAAGGAGCTGTAGAAGTAGATGTAGCAGGAGTTAAAGATCGCTTAGACTTTCAAAACTTAGCTGTAGTCGTCTTCTACGAGGTAGTTGTCGTGGAAGTAGTTC
386 388 390 392 394 396 398 400 402 404 406 408 410 412 414 416 418 420
P S S S T S S S T S S S I S S E S E S F E S T S A E D A P S T A P S S S

649 stalk

4,940 4,960 4,980 5,000 5,020

TGTCTCTTCTAAGAGTTCTACCTCTACAACATCAAGCACATCGACATCTTCAAGCACTCCATCTCCATCACCATCTTCCGTGAGTTCTTCCTCCACCAGCTCATTGA
ACAGAGAAGATTCTCAAGATGGAGATGTTGTAGTTCGTGTAGCTGTAGAAGTTCGTGAGGTAGAGGTAGTGGTAGAAGGCACTCAAGAAGGAGGTGGTCGAGTAACT
422 424 426 428 430 432 434 436 438 440 442 444 446 448 450 452 454
V S S K S S T S T T S S T S T S S S T P S P S P S S V S S S S T S S L

649 stalk

5,040 5,060 5,080 5,100 5,120

CAACTTCTGCTGTATCAACACCAGCTACCTCTCATTCTCAAAGTACTGTAGTAACCACCACTACTATTACTACATCAACAGGTCCAGTGATGTCTACGACAACAGCT
GTTGAAGACGACATAGTTGTGGTCGATGGAGAGTAAGAGTTTCATGACATCATTGGTGGTGATGATAATGATGTAGTTGTCCAGGTCACTACAGATGCTGTTGTCGA
456 458 460 462 464 466 468 470 472 474 476 478 480 482 484 486 488 490
T T S A V S T P A T S H S Q S T V V T T T T I T T S T G P V M S T T T A

649 stalk

5,140 5,160 5,180 5,200 5,220 5,240

TATTCTTCTAGTTCTACTAGCAGCTCGGAATCTTCTGAGGTTCAGTCTGTCATGTCATCTACGCCTAGTTCAACATCAACAACAACCAGTTCGGAATCTACTTCATC
ATAAGAAGATCAAGATGATCGTCGAGCCTTAGAAGACTCCAAGTCAGACAGTACAGTAGATGCGGATCAAGTTGTAGTTGTTGTTGGTCAAGCCTTAGATGAAGTAG
492 494 496 498 500 502 504 506 508 510 512 514 516 518 520 522 524 526
Y S S S S T S S S E S S E V Q S V M S S T P S S T S T T T S S E S T S S

649 stalk

5,260 5,280 5,300 5,320 5,340

TAGCTCCACAGCTTCTACCTCACCATCAACCTCGCAAACTTTCGAAACTTCTCCTACTATAGGAGGTGTCCCCTCAACCACTTCATTTGTCTCTACGCCAACAACGA
ATCGAGGTGTCGAAGATGGAGTGGTAGTTGGAGCGTTTGAAAGCTTTGAAGAGGATGATATCCTCCACAGGGGAGTTGGTGAAGTAAACAGAGATGCGGTTGTTGCT
528 530 532 534 536 538 540 542 544 546 548 550 552 554 556 558 560 562
S S T A S T S P S T S Q T F E T S P T I G G V P S T T S F V S T P T T
649 stalk
5,360 5,380 5,400 5,420 5,440

AATTGTCGCACACTACTTCCACTATGACAGCACAGTCCGATAGTAAGTCTACCCACTCCTCAAGCACATCGACAGAAGATAAATCATCCACTGCTTCTGCAGTTGAC
TTAACAGCGTGTGATGAAGGTGATACTGTCGTGTCAGGCTATCATTCAGATGGGTGAGGAGTTCGTGTAGCTGTCTTCTATTTAGTAGGTGACGAAGACGTCAACTG
564 566 568 570 572 574 576 578 580 582 584 586 588 590 592 594 596 598
K L S H T T S T M T A Q S D S K S T H S S S T S T E D K S S T A S A V D
649 stalk
5,460 5,480 5,500 5,520 5,540 5,560

GAAAGCACTACAACATCCACTTCCACGGAGTCTACTACATCAGTAACATCAGGCACCTCCCATTCCGCTAAAGAATCTTCGTCAAATTCTAAGGTGTATAGTTCACA
CTTTCGTGATGTTGTAGGTGAAGGTGCCTCAGATGATGTAGTCATTGTAGTCCGTGGAGGGTAAGGCGATTTCTTAGAAGCAGTTTAAGATTCCACATATCAAGTGT
600 602 604 606 608 610 612 614 616 618 620 622 624 626 628 630 632 634
E S T T T S T S T E S T T S V T S G T S H S A K E S S S N S K V Y S S Q
649 stalk
5,580 5,600 5,620 5,640 5,660

GACAGCACACTCATCCATAAGTGTTGCATCATCACCTAGTACAAAGGGCGCCCAAATCCAATCTTCTATGGTTGAAATCTCTACCTACGCTGGTTCTGCTAACTCTG
CTGTCGTGTGAGTAGGTATTCACAACGTAGTAGTGGATCATGTTTCCCGCGGGTTTAGGTTAGAAGATACCAACTTTAGAGATGGATGCGACCAAGACGATTGAGAC
636 638 640 642 644 646 648 650 652 654 656 658 660 662 664 666 668
T A H S S I S V A S S P S T K G A Q I Q S S M V E I S T Y A G S A N S
649 stalk GPI anchor
5,680 5,700 5,720 5,740 5,760

TTAACGCTGGTGCTGGTGCTGGTGCTTTGTTCTTGTTGTTGTCTTTGGCTATCATCTAATGATTAATTAACTCGAGATCTGATAACAACAGTGTAGATGTAACAAAA
AATTGCGACCACGACCACGACCACGAAACAAGAACAACAACAGAAACCGATAGTAGATTACTAATTAATTGAGCTCTAGACTATTGTTGTCACATCTACATTGTTTT
670 672 674 676 678 680 682 684 686 688 1
V N A G A G A G A L F L L L S L A I I *
GPI anchor alpha factor terminator
5,780 5,800 5,820 5,840 5,860 5,880

TCGACTTTGTTCCCACTGTACTTTTAGCTCGTACAAAATACAATATACTTTTCATTTCTCCGTAAACAACATGTTTTCCCATGTAATATCCTTTTCTATTTTTCGTT
AGCTGAAACAAGGGTGACATGAAAATCGAGCATGTTTTATGTTATATGAAAAGTAAAGAGGCATTTGTTGTACAAAAGGGTACATTATAGGAAAAGATAAAAAGCAA
alpha factor terminator
5,900 5,920 5,940 5,960 5,980

CCGTTACCAACTTTACACATACTTTATATAGCTATTCACTTCTATACACTAAAAAACTAAGACAATTTTAATTTTGCTGCCTGCCATATTTCAATTTGTTATAAATT
GGCAATGGTTGAAATGTGTATGAAATATATCGATAAGTGAAGATATGTGATTTTTTGATTCTGTTAAAATTAAAACGACGGACGGTATAAAGTTAAACAATATTTAA
alpha factor terminator
6,000 6,020 6,040 6,060 6,080

```
CCTATAATTTATCCTATTAGTAGCTAAAAAAAGATGAATGTGAATCGAATCCTAAGAGAATTcgaattgggtaccgggccccccctcgaggtcgacggtatcgataa
GGATATTAAATAGGATAATCATCGATTTTTTTCTACTTACACTTAGCTTAGGATTCTCTTAAgcttaacccatggcccgggggggagctccagctgccatagctatt
alpha factor terminator
6,100  6,120  6,140  6,160  6,180  6,200

gcttgatatcgaattcctgcagcccgggggatccactagttctagagcggccgccaccgcggtggagctccagcttttgttccctttagtgagggttaattgcgcgc
cgaactatagcttaaggacgtcgggccccctaggtgatcaagatctcgccggcggtggcgccacctcgaggtcgaaaacaagggaaatcactcccaattaacgcgcg
6,220  6,240  6,260  6,280  6,300

ttggcgtaatcatggtcatagctgtttcctgtgtgaaattgttatccgctcacaattccacacaacataggagccggaagcataaagtgtaaagcctggggtgccta
aaccgcattagtaccagtatcgacaaaggacacactttaacaataggcgagtgttaaggtgtgttgtatcctcggccttcgtatttcacatttcggaccccacggat
LacO
6,320  6,340  6,360  6,380  6,400  6,420

atgagtgaggtaactcacattaattgcgttgcgctcactgcccgctttccagtcgggaaacctgtcgtgccagctgcattaatgaatcggccaacgcgcggggagag
tactcactccattgagtgtaattaacgcaacgcgagtgacgggcgaaaggtcagccctttggacagcacggtcgacgtaattacttagccggttgcgcgcccctctc
6,440  6,460  6,480  6,500  6,520

gcggtttgcgtattgggcgctcttccgcttcctcgctcactgactcgctgcgctcggtcgttcggctgcggcgagcggtatcagctcactcaaaggcggtaatacgg
cgccaaacgcataacccgcgagaaggcgaaggagcgagtgactgagcgacgcgagccagcaagccgacgccgctcgccatagtcgagtgagtttccgccattatgcc
6,540  6,560  6,580  6,600  6,620

ttatccacagaatcaggggataacgcaggaaagaacatgtgagcaaaaggccagcaaaaggccaggaaccgtaaaaaggccgcgttgctggcgtttttccataggct
aataggtgtcttagtcccctattgcgtcctttcttgtacactcgttttccggtcgttttccggtccttggcatttttccggcgcaacgaccgcaaaaaggtatccga
ColE1 origin
6,640  6,660  6,680  6,700  6,720  6,740

ccgcccccctgacgagcatcacaaaaatcgacgctcaagtcagaggtggcgaaacccgacaggactataaagataccaggcgtttccccctggaagctccctcgtgc
ggcggggggactgctcgtagtgtttttagctgcgagttcagtctccaccgctttgggctgtcctgatatttctatggtccgcaaagggggaccttcgagggagcacg
ColE1 origin
6,760  6,780  6,800  6,820  6,840

gctctcctgttccgaccctgccgcttaccggatacctgtccgcctttctcccttcgggaagcgtggcgctttctcatagctcacgctgtaggtatctcagttcggtg
cgagaggacaaggctgggacggcgaatggcctatggacaggcggaaagagggaagcccttcgcaccgcgaaagagtatcgagtgcgacatccatagagtcaagccac
ColE1 origin
6,860  6,880  6,900  6,920  6,940

taggtcgttcgctccaagctgggctgtgtgcacgaaccccccgttcagcccgaccgctgcgccttatccggtaactatcgtcttgagtccaacccggtaagacacga
atccagcaagcgaggttcgacccgacacacgtgcttggggggcaagtcgggctggcgacgcggaataggccattgatagcagaactcaggttgggccattctgtgct
ColE1 origin
6,960  6,980  7,000  7,020  7,040  7,060
```

cttatcgccactggcagcagccactggtaacaggattagcagagcgaggtatgtaggcggtgctacagagttcttgaagtggtggcctaactacggctacactagaa
gaatagcggtgaccgtcgtcggtgaccattgtcctaatcgtctcgctccatacatccgccacgatgtctcaagaacttcaccaccggattgatgccgatgtgatctt

ColE1 origin

7,080 7,100 7,120 7,140 7,160

ggacagtatttggtatctgcgctctgctgaagccagttaccttcggaaaaagagttggtagctcttgatccggcaaacaaaccaccgctggtagcggtggttttttt
cctgtcataaaccatagacgcgagacgacttcggtcaatggaagcctttttctcaaccatcgagaactaggccgtttgtttggtggcgaccatcgccaccaaaaaaa

ColE1 origin

7,180 7,200 7,220 7,240 7,260

gtttgcaagcagcagattacgcgcagaaaaaaaggatctcaagaagatcctttgatcttttctacggggtctgacgctcagtggaacgaaaactcacgttaagggat
caaacgttcgtcgtctaatgcgcgtcttttttttcctagagttcttctaggaaactagaaaagatgccccagactgcgagtcaccttgcttttgagtgcaattcccta

ColE1 origin

7,280 7,300 7,320 7,340 7,360 7,380

tttggtcatgagattatcaaaaaggatcttcacctagatccttttaaattaaaaatgaagttttaaatcaatctaaagtatatatgagtaaacttggtctgacagtt
aaaccagtactctaatagtttttcctagaagtggatctaggaaaatttaatttttacttcaaaatttagttagatttcatatatactcatttgaaccagactgtcaa

Co...n

7,400 7,420 7,440 7,460 7,480

accaatgcttaatcagtgaggcacctatctcagcgatctgtctatttcgttcatccatagttgcctgactccccgtcgtgtagataactacgatacgggagggctta
tggttacgaattagtcactccgtggatagagtcgctagacagataaagcaagtaggtatcaacggactgaggggcagcacatctattgatgctatgccctcccgaat

AmpR

7,500 7,520 7,540 7,560 7,580

ccatctggccccagtgctgcaatgataccgcgagacccacgctcaccggctccagatttatcagcaataaaccagccagccggaagggccgagcgcagaagtggtcc
ggtagaccggggtcacgacgttactatggcgctctgggtgcgagtggccgaggtctaaatagtcgttatttggtcggtcggccttcccggctcgcgtcttcaccagg

AmpR

7,600 7,620 7,640 7,660 7,680 7,700

tgcaactttatccgcctccatccagtctattaattgttgccgggaagctagagtaagtagttcgccagttaatagtttgcgcaacgttgttgccattgctacaggca
acgttgaaataggcggaggtaggtcagataattaacaacggcccttcgatctcattcatcaagcggtcaattatcaaacgcgttgcaacaacggtaacgatgtccgt

AmpR

7,720 7,740 7,760 7,780 7,800

tcgtggtgtcacgctcgtcgtttggtatggcttcattcagctccggttcccaacgatcaaggcgagttacatgatcccccatgttgtgcaaaaaagcggttagctcc
agcaccacagtgcgagcagcaaaccataccgaagtaagtcgaggccaagggttgctagttccgctcaatgtactagggggtacaacacgttttttcgccaatcgagg

AmpR

7,820 7,840 7,860 7,880 7,900

```
ttcggtcctccgatcgttgtcagaagtaagttggccgcagtgttatcactcatggttatggcagcactgcataattctcttactgtcatgccatccgtaagatgctt
aagccaggaggctagcaacagtcttcattcaaccggcgtcacaatagtgagtaccaataccgtcgtgacgtattaagagaatgacagtacggtaggcattctacgaa
                                              AmpR
7,920        7,940        7,960        7,980        8,000        8,020

ttctgtgactggtgagtactcaaccaagtcattctgagaatagtgtatgcggcgaccgagttgctcttgcccggcgtcaatacgggataataccgcgccacatagca
aagacactgaccactcatgagttggttcagtaagactcttatcacatacgccgctggctcaacgagaacgggccgcagttatgccctattatggcgcggtgtatcgt
                                              AmpR
        8,040        8,060        8,080        8,100        8,120

gaactttaaaagtgctcatcattggaaaacgttcttcggggcgaaaactctcaaggatcttaccgctgttgagatccagttcgatgtaacccactcgtgcacccaac
cttgaaattttcacgagtagtaaccttttgcaagaagccccgcttttgagagttcctagaatggcgacaactctaggtcaagctacattgggtgagcacgtgggttg
      AmpR
    8,140        8,160        8,180        8,200        8,220

tgatcttcagcatcttttactttcaccagcgtttctgggtgagcaaaaacaggaaggcaaaatgccgcaaaaaagggaataagggcgacacggaaatgttgaatact
actagaagtcgtagaaaatgaaagtggtcgcaaagacccactcgtttttgtccttccgttttacggcgttttttcccttattcccgctgtgcctttacaacttatga
8,240        8,260        8,280        8,300        8,320        8,340

catactcttcctttttcaatattattgaagcatttatcagggttattgtctcatgagcggatacatatttgaatgtatttagaaaaataaacaaataggggttccgc
gtatgagaaggaaaaagttataataacttcgtaaatagtcccaataacagagtactcgcctatgtataaacttacataaatctttttatttgtttatccccaaggcg
           8,360        8,380        8,400        8,420        8,440

gcacatttccccgaaaagtgccacctgacgtctaagaaaccattattatcatgacattaacctataaaaataggcgtatcacgaggccctttcgtc
cgtgtaaaggggcttttcacggtggactgcagattctttggtaataatagtactgtaattggatatttttatccgcatagtgctccgggaaagcag
    8,460   8,470   8,480   8,490   8,500   8,510   8,520   8,530   8,540
```

ST/SC ligation / pNb_lys Nb-FLAG (pRS423) (7440...

TTATGGAAGAGAAAATAGACACCAAAGTAGCCTTCTTCTAACCTTAACGGACCTACAGTGCAAAAAGTTATCAAGAGACTGCATTATAGAGCGCACAAAGGAGAAAA
AATACCTTCTCTTTTATCTGTGGTTTCATCGGAAGAAGATTGGAATTGCCTGGATGTCACGTTTTTCAATAGTTCTCTGACGTAATATCTCGCGTGTTTCCTCTTTT

2 micron origin

20 40 60 80 100

AAAGTAATCTAAGATGCTTTGTTAGAAAAATAGCGCTCTCGGGATGCATTTTTGTAGAACAAAAAAGAAGTATAGATTCTTTGTTGGTAAAATAGCGCTCTCGCGTT
TTTCATTAGATTCTACGAAACAATCTTTTTATCGCGAGAGCCCTACGTAAAAACATCTTGTTTTTTCTTCATATCTAAGAAACAACCATTTTATCGCGAGAGCGCAA

2 micron origin

120 140 160 180 200

GCATTTCTGTTCTGTAAAAATGCAGCTCAGATTCTTTGTTTGAAAAATTAGCGCTCTCGCGTTGCATTTTTGTTTTACAAAAATGAAGCACAGATTCTTCGTTGGTA
CGTAAAGACAAGACATTTTTACGTCGAGTCTAAGAAACAAACTTTTTAATCGCGAGAGCGCAACGTAAAAACAAAATGTTTTTACTTCGTGTCTAAGAAGCAACCAT

2 micron origin

220 240 260 280 300 320

AAATAGCGCTTTCGCGTTGCATTTCTGTTCTGTAAAAATGCAGCTCAGATTCTTTGTTTGAAAAATTAGCGCTCTCGCGTTGCATTTTTGTTCTACAAAATGAAGCA
TTTATCGCGAAAGCGCAACGTAAAGACAAGACATTTTTACGTCGAGTCTAAGAAACAAACTTTTTAATCGCGAGAGCGCAACGTAAAAACAAGATGTTTTACTTCGT

2 micron origin

340 360 380 400 420

CAGATGCTTCGTTCAGGTGGCACTTTTCGGGGAAATGTGCGCGGAACCCCTATTTGTTTATTTTTCTAAATACATTCAAATATGTATCCGCTCATGAGACAATAACC
GTCTACGAAGCAAGTCCACCGTGAAAAGCCCCTTTACACGCGCCTTGGGGATAAACAAATAAAAAGATTTATGTAAGTTTATACATAGGCGAGTACTCTGTTATTGG

2 ...n

440 460 480 500 520

CTGATAAATGCTTCAATAATATTGAAAAAGGAAGAGTATGAGTATTCAACATTTCCGTGTCGCCCTTATTCCCTTTTTTGCGGCATTTTGCCTTCCTGTTTTTGCTC
GACTATTTACGAAGTTATTATAACTTTTTCCTTCTCATACTCATAAGTTGTAAAGGCACAGCGGGAATAAGGGAAAAAACGCCGTAAAACGGAAGGACAAAAACGAG

540 560 580 600 620 640

ACCCAGAAACGCTGGTGAAAGTAAAAGATGCTGAAGATCAGTTGGGTGCACGAGTGGGTTACATCGAACTGGATCTCAACAGCGGTAAGATCCTTGAGAGTTTTCGC
TGGGTCTTTGCGACCACTTTCATTTTCTACGACTTCTAGTCAACCCACGTGCTCACCCAATGTAGCTTGACCTAGAGTTGTCGCCATTCTAGGAACTCTCAAAAGCG

660 680 700 720 740

CCCGAAGAACGTTTTCCAATGATGAGCACTTTTAAAGTTCTGCTATGTGGCGCGGTATTATCCCGTATTGACGCCGGGCAAGAGCAACTCGGTCGCCGCATACACTA
GGGCTTCTTGCAAAAGGTTACTACTCGTGAAAATTTCAAGACGATACACCGCGCCATAATAGGGCATAACTGCGGCCCGTTCTCGTTGAGCCAGCGGCGTATGTGAT

AmpR

760 780 800 820 840

TTCTCAGAATGACTTGGTTGAGTACTCACCAGTCACAGAAAAGCATCTTACGGATGGCATGACAGTAAGAGAATTATGCAGTGCTGCCATAACCATGAGTGATAACA
AAGAGTCTTACTGAACCAACTCATGAGTGGTCAGTGTCTTTTCGTAGAATGCCTACCGTACTGTCATTCTCTTAATACGTCACGACGGTATTGGTACTCACTATTGT

AmpR

860 880 900 920 940 960

CTGCGGCCAACTTACTTCTGACAACGATCGGAGGACCGAAGGAGCTAACCGCTTTTTTGCACAACATGGGGGATCATGTAACTCGCCTTGATCGTTGGGAACCGGAG
GACGCCGGTTGAATGAAGACTGTTGCTAGCCTCCTGGCTTCCTCGATTGGCGAAAAAACGTGTTGTACCCCCTAGTACATTGAGCGGAACTAGCAACCCTTGGCCTC

AmpR

980 1,000 1,020 1,040 1,060

CTGAATGAAGCCATACCAAACGACGAGCGTGACACCACGATGCCTGTAGCAATGGCAACAACGTTGCGCAAACTATTAACTGGCGAACTACTTACTCTAGCTTCCCG
GACTTACTTCGGTATGGTTTGCTGCTCGCACTGTGGTGCTACGGACATCGTTACCGTTGTTGCAACGCGTTTGATAATTGACCGCTTGATGAATGAGATCGAAGGGC

AmpR

1,080 1,100 1,120 1,140 1,160

GCAACAATTAATAGACTGGATGGAGGCGGATAAAGTTGCAGGACCACTTCTGCGCTCGGCCCTTCCGGCTGGCTGGTTTATTGCTGATAAATCTGGAGCCGGTGAGC
CGTTGTTAATTATCTGACCTACCTCCGCCTATTTCAACGTCCTGGTGAAGACGCGAGCCGGGAAGGCCGACCGACCAAATAACGACTATTTAGACCTCGGCCACTCG

AmpR

1,180 1,200 1,220 1,240 1,260 1,280

GTGGGTCTCGCGGTATCATTGCAGCACTGGGGCCAGATGGTAAGCCCTCCCGTATCGTAGTTATCTACACGACGGGGAGTCAGGCAACTATGGATGAACGAAATAGA
CACCCAGAGCGCCATAGTAACGTCGTGACCCCGGTCTACCATTCGGGAGGGCATAGCATCAATAGATGTGCTGCCCCTCAGTCCGTTGATACCTACTTGCTTTATCT

AmpR

1,300 1,320 1,340 1,360 1,380

CAGATCGCTGAGATAGGTGCCTCACTGATTAAGCATTGGTAACTGTCAGACCAAGTTTACTCATATATACTTTAGATTGATTTAAAACTTCATTTTTAATTTAAAAG
GTCTAGCGACTCTATCCACGGAGTGACTAATTCGTAACCATTGACAGTCTGGTTCAAATGAGTATATATGAAATCTAACTAAATTTTGAAGTAAAAATTAAATTTTC

AmpR

1,400 1,420 1,440 1,460 1,480

GATCTAGGTGAAGATCCTTTTTGATAATCTCATGACCAAAATCCCTTAACGTGAGTTTTCGTTCCACTGAGCGTCAGACCCCGTAGAAAAGATCAAAGGATCTTCTT
CTAGATCCACTTCTAGGAAAAACTATTAGAGTACTGGTTTTAGGGAATTGCACTCAAAAGCAAGGTGACTCGCAGTCTGGGGCATCTTTTCTAGTTTCCTAGAAGAA

1,500 1,520 1,540 1,560 1,580 1,600

GAGATCCTTTTTTTCTGCGCGTAATCTGCTGCTTGCAAACAAAAAAACCACCGCTACCAGCGGTGGTTTGTTTGCCGGATCAAGAGCTACCAACTCTTTTTCCGAAG
CTCTAGGAAAAAAAGACGCGCATTAGACGACGAACGTTTGTTTTTTTGGTGGCGATGGTCGCCACCAAACAAACGGCCTAGTTCTCGATGGTTGAGAAAAAGGCTTC

1,620 1,640 1,660 1,680 1,700

GTAACTGGCTTCAGCAGAGCGCAGATACCAAATACTGTTCTTCTAGTGTAGCCGTAGTTAGGCCACCACTTCAAGAACTCTGTAGCACCGCCTACATACCTCGCTCT
CATTGACCGAAGTCGTCTCGCGTCTATGGTTTATGACAAGAAGATCACATCGGCATCAATCCGGTGGTGAAGTTCTTGAGACATCGTGGCGGATGTATGGAGCGAGA

1,720 1,740 1,760 1,780 1,800

GCTAATCCTGTTACCAGTGGCTGCTGCCAGTGGCGATAAGTCGTGTCTTACCGGGTTGGACTCAAGACGATAGTTACCGGATAAGGCGCAGCGGTCGGGCTGAACGG
CGATTAGGACAATGGTCACCGACGACGGTCACCGCTATTCAGCACAGAATGGCCCAACCTGAGTTCTGCTATCAATGGCCTATTCCGCGTCGCCAGCCCGACTTGCC

1,820 1,840 1,860 1,880 1,900 1,920

GGGGTTCGTGCACACAGCCCAGCTTGGAGCGAACGACCTACACCGAACTGAGATACCTACAGCGTGAGCTATGAGAAAGCGCCACGCTTCCCGAAGGGAGAAAGGCG
CCCCAAGCACGTGTGTCGGGTCGAACCTCGCTTGCTGGATGTGGCTTGACTCTATGGATGTCGCACTCGATACTCTTTCGCGGTGCGAAGGGCTTCCCTCTTTCCGC

1,940 1,960 1,980 2,000 2,020

GACAGGTATCCGGTAAGCGGCAGGGTCGGAACAGGAGAGCGCACGAGGGAGCTTCCAGGGGGAAACGCCTGGTATCTTTATAGTCCTGTCGGGTTTCGCCACCTCTG
CTGTCCATAGGCCATTCGCCGTCCCAGCCTTGTCCTCTCGCGTGCTCCCTCGAAGGTCCCCCTTTGCGGACCATAGAAATATCAGGACAGCCCAAAGCGGTGGAGAC

2,040 2,060 2,080 2,100 2,120 2,140

ACTTGAGCGTCGATTTTTGTGATGCTCGTCAGGGGGGCGGAGCCTATGGAAAAACGCCAGCAACGCGGCCTTTTTACGGTTCCTGGCCTTTTGCTGGCCTTTTGCTC
TGAACTCGCAGCTAAAAACACTACGAGCAGTCCCCCCGCCTCGGATACCTTTTTGCGGTCGTTGCGCCGGAAAAATGCCAAGGACCGGAAAACGACCGGAAAACGAG

2,160 2,180 2,200 2,220 2,240

ACATGTTCTTTCCTGCGTTATCCCCTGATTCTGTGGATAACCGTATTACCGCCTTTGAGTGAGCTGATACCGCTCGCCGCAGCCGAACGACCGAGCGCAGCGAGTCA
TGTACAAGAAAGGACGCAATAGGGGACTAAGACACCTATTGGCATAATGGCGGAAACTCACTCGACTATGGCGAGCGGCGTCGGCTTGCTGGCTCGCGTCGCTCAGT

2,260 2,280 2,300 2,320 2,340

GTGAGCGAGGAAGCGGAAGAGCGCCCAATACGCAAACCGCCTCTCCCCGCGCGTTGGCCGATTCATTAATGCAGCTGGCACGACAGGTTTCCCGACTGGAAAGCGGG
CACTCGCTCCTTCGCCTTCTCGCGGGTTATGCGTTTGGCGGAGAGGGGCGCGCAACCGGCTAAGTAATTACGTCGACCGTGCTGTCCAAAGGGCTGACCTTTCGCCC

2,360 2,380 2,400 2,420 2,440 2,460

CAGTGAGCGCAACGCAATTAATGTGAGTTAGCTCACTCATTAGGCACCCCAGGCTTTACACTTTATGCTTCCGGCTCGTATGTTGTGTGGAATTGTGAGCGGATAAC
GTCACTCGCGTTGCGTTAATTACACTCAATCGAGTGAGTAATCCGTGGGGTCCGAAATGTGAAATACGAAGGCCGAGCATACAACACACCTTAACACTCGCCTATTG

lac promoter LacO

2,480 2,500 2,520 2,540 2,560

AATTTCACACAGGAAACAGCTATGACCATGATTACGCCAAGCGCGCAATTAACCCTCACTAAAGGGAACAAAAGCTGGAGCTcAGTTTATCATTATCAATACTcGCC
TTAAAGTGTGTCCTTTGTCGATACTGGTACTAATGCGGTTCGCGCGTTAATTGGGAGTGATTTCCCTTGTTTTCGACCTCGAgTCAAATAGTAATAGTTATGAgCGG

T3

M13-rev T3 promoter GPD promoter

2,580 2,600 2,620 2,640 2,660

ATTTCAAAGAATACGTAAATAATTAATAGTAGTGATTTTCCTAACTTTATTTAGTCAAAAAATTAGCCTTTTAATTCTGCTGTAACCCGTACATGCCCAAAATAGGG
TAAAGTTTCTTATGCATTTATTAATTATCATCACTAAAAGGATTGAAATAAATCAGTTTTTTAATCGGAAAATTAAGACGACATTGGGCATGTACGGGTTTTATCCC

GPD promoter

2,680 2,700 2,720 2,740 2,760 2,780

GGCGGGTTACACAGAATATATAACATCGTAGGTGTCTGGGTGAACAGTTTATTCCTGGCATCCACTAAATATAATGGAGCCCGCTTTTTAAGCTGGCATCCAGAAAA
CCGCCCAATGTGTCTTATATATTGTAGCATCCACAGACCCACTTGTCAAATAAGGACCGTAGGTGATTTATATTACCTCGGGCGAAAAATTCGACCGTAGGTCTTTT

GPD promoter

2,800 2,820 2,840 2,860 2,880

AAAAAGAATCCCAGCACCAAAATATTGTTTTCTTCACCAACCATCAGTTCATAGGTCCATTCTCTTAGCGCAACTACAGAGAACAGGGGCACAAACAGGCAAAAAAC
TTTTTCTTAGGGTCGTGGTTTTATAACAAAAGAAGTGGTTGGTAGTCAAGTATCCAGGTAAGAGAATCGCGTTGATGTCTCTTGTCCCCGTGTTTGTCCGTTTTTTG
GPD promoter
2,900 2,920 2,940 2,960 2,980

GGGCACAACCTCAATGGAGTGATGCAACCTGCCTGGAGTAAATGATGACACAAGGCAATTGACCCACGCATGTATCTATCTCATTTTCTTACACCTTCTATTACCTT
CCCGTGTTGGAGTTACCTCACTACGTTGGACGGACCTCATTTACTACTGTGTTCCGTTAACTGGGTGCGTACATAGATAGAGTAAAAGAATGTGGAAGATAATGGAA
GPD promoter
3,000 3,020 3,040 3,060 3,080 3,100

CTGCTCTCTCTGATTTGGAAAAAGCTGAAAAAAAAAGGTTGAAACCAGTTCCCTGAAATTATTCCCCTACTTGACTAATAAGTATATAAAGACGGTAGGTATTGATTG
GACGAGAGAGACTAAACCTTTTTCGACTTTTTTTTTCCAACTTTGGTCAAGGGACTTTAATAAGGGGATGAACTGATTATTCATATATTTCTGCCATCCATAACTAAC
GPD promoter
3,120 3,140 3,160 3,180 3,200

TAATTCTGTAAATCTATTTCTTAAACTTCTTAAATTCTACTTTTATAGTTAGTCTTTTTTTTAGTTTTAAAACACCAGAACTTAGTTTCGACGGATTCTAGAACTAG
ATTAAGACATTTAGATAAAGAATTTGAAGAATTTAAGATGAAAATATCAATCAGAAAAAAAATCAAAATTTTGTGGTCTTGAATCAAAGCTGCCTAAGATCTTGATC
GPD promoter
3,220 3,240 3,260 3,280 3,300

TGGATCCATGAGATTCCCATCTATCTTCACCGCTGTTTTGTTCGCTGCTTCTTCTGCTTTGGCTGCTCCAGCTAACACCACCACCGAAGACGAAACCGCTCAAATCC
ACCTAGGTACTCTAAGGGTAGATAGAAGTGGCGACAAAACAAGCGACGAAGAAGACGAAACCGACGAGGTCGATTGTGGTGGTGGCTTCTGCTTTGGCGAGTTTAGG
1 2 3 4 5 6 7 8 9 10 11 12 13 14 15 16 17 18 19 20 21 22 23 24 25 26 27 28 29 30 31 32 33
M R F P S I F T A V L F A A S S A L A A P A N T T T E D E T A Q I
Mating factor alpha leader
3,320 3,340 3,360 3,380 3,400 3,420

CAGCTGAAGCTGTTATCGACTACTCTGACTTGGAAGGTGACTTCGACGCTGCTGCTTTGCCATTGTCTAACTCTACCAACAACGGTTTGTCTTCTACCAACACCACC
GTCGACTTCGACAATAGCTGATGAGACTGAACCTTCCACTGAAGCTGCGACGACGAAACGGTAACAGATTGAGATGGTTGTTGCCAAACAGAAGATGGTTGTGGTGG
34 35 36 37 38 39 40 41 42 43 44 45 46 47 48 49 50 51 52 53 54 55 56 57 58 59 60 61 62 63 64 65 66 67 68 69
P A E A V I D Y S D L E G D F D A A A L P L S N S T N N G L S S T N T T
Mating factor alpha leader
3,440 3,460 3,480 3,500 3,520

ATCGCTTCTATCGCTGCTAAGGAAGAAGGTGTTCAATTGGACAAGAGAGAAagatctggcggccgcgcatgcgctagcctcgagGATGTGCAGTTGCAAGCATCCGG
TAGCGAAGATAGCGACGATTCCTTCTTCCACAAGTTAACCTGTTCTCTCTTtctagaccgccggcgcgtacgcgatcggagctcCTACACGTCAACGTTCGTAGGCC
70 71 72 73 74 75 76 77 78 79 80 81 82 83 84 85 86
I A S I A A K E E G V Q L D K R E
2 4 6 8
D V Q L Q A S G
Mating factor alpha leader
anti-lys...anobody
3,540 3,560 3,580 3,600 3,620

CGGCGGTTCTGTTCAGGCTGGTGGTTCTCTAAGATTAAGTTGTGCTGCTTCAGGTTATACCATCGGCCCATACTGCATGGGTTGGTTTCGTCAAGCTCCAGGCAAAG
GCCGCCAAGACAAGTCCGACCACCAAGAGATTCTAATTCAACACGACGAAGTCCAATATGGTAGCCGGGTATGACGTACCCAACCAAAGCAGTTCGAGGTCCGTTTC

10 12 14 16 18 20 22 24 26 28 30 32 34 36 38 40 42
G G S V Q A G G S L R L S C A A S G Y T I G P Y C M G W F R Q A P G K

anti-lysozyme nanobody

3,640 3,660 3,680 3,700 3,720 3,740

AAAGGGAGGGAGTCGCAGCCATTAACATGGGCGGCGGTATCACATATTATGCAGACTCCGTAAAAGGTAGATTTACTATCTCTCAGGACAATGCTAAGAACACGGTC
TTTCCCTCCCTCAGCGTCGGTAATTGTACCCGCCGCCATAGTGTATAATACGTCTGAGGCATTTTCCATCTAAATGATAGAGAGTCCTGTTACGATTCTTGTGCCAG

44 46 48 50 52 54 56 58 60 62 64 66 68 70 72 74 76 78
E R E G V A A I N M G G G I T Y Y A D S V K G R F T I S Q D N A K N T V

anti-lysozyme nanobody

3,760 3,780 3,800 3,820 3,840

TATCTGTTAATGAATTCATTGGAGCCCGAGGACACTGCCATATACTACTGTGCCGCAGATTCAACCATTTACGCTTCCTATTACGAATGCGGACACGGATTAAGTAC
ATAGACAATTACTTAAGTAACCTCGGGCTCCTGTGACGGTATATGATGACACGGCGTCTAAGTTGGTAAATGCGAAGGATAATGCTTACGCCTGTGCCTAATTCATG

80 82 84 86 88 90 92 94 96 98 100 102 104 106 108 110 112 114
Y L L M N S L E P E D T A I Y Y C A A D S T I Y A S Y Y E C G H G L S T

anti-lysozyme nanobody

3,860 3,880 3,900 3,920 3,940

TGGAGGTTACGGTTATGATTCCTGGGGTCAAGGCACACAAGTCACGGTATCATCAGGCGGATCAGACTATAAGGATGACGACGATAAGGCTGGAGGTTCGAGGGGAG
ACCTCCAATGCCAATACTAAGGACCCCAGTTCCGTGTGTTCAGTGCCATAGTAGTCCGCCTAGTCTGATATTCCTACTGCTGCTATTCCGACCTCCAAGCTCCCCTC

116 118 120 122 124 126 128 130 132 1 2 3 1 2 3 4 5 6 7 8 1 2 3 4 1 2
G G Y G Y D S W G Q G T Q V T V S S G G S D Y K D D D D K A G G S R G

anti-lysozyme nanobody FLAG tag

3,960 3,980 4,000 4,020 4,040 4,060

TCCCTCACATAGTGATGGTGGATGCTTACAAAAGGTATAAATAACCCGGGCTGCAGGAATTCGATATCAAGCTTATCGATACCGTCGACCTCGAGTCATGTAATTAG
AGGGAGTGTATCACTACCACCTACGAATGTTTTCCATATTTATTGGGCCCGACGTCCTTAAGCTATAGTTCGAATAGCTATGGCAGCTGGAGCTCAGTACATTAATC

3 4 5 6 7 8 9 10 11 12 13 14 15 16 1
V P H I V M V D A Y K R Y K *

SpyTag CY...or

4,080 4,100 4,120 4,140 4,160

TTATGTCACGCTTACGTTCACGCCCTCCCCCCACATCCGCTCTAACCGAAAAGGAAGGAGTTAGACAACCTGAAGTCTAGGTCCCTATTTATTTTTTTATAGTTATG
AATACAGTGCGAATGCAAGTGCGGGAGGGGGGTGTAGGCGAGATTGGCTTTTCCTTCCTCAATCTGTTGGACTTCAGATCCAGGGATAAATAAAAAAATATCAATAC

CYC1 terminator

4,180 4,200 4,220 4,240 4,260 4,280

TTAGTATTAAGAACGTTATTTATATTTCAAATTTTTCTTTTTTTTCTGTACAGACGCGTGTACGCATGTAACATTATACTGAAAACCTTGCTTGAGAAGGTTTTGGG
AATCATAATTCTTGCAATAAATATAAAGTTTAAAAAGAAAAAAAAGACATGTCTGCGCACATGCGTACATTGTAATATGACTTTTGGAACGAACTCTTCCAAAACCC

CYC1 terminator

4,300 4,320 4,340 4,360 4,380

ACGCTCGAAGGCTTTAATTTGCGGCCGGTACCCAATTCGCCCTATAGTGAGTCGTATTACGCGCGCTCACTGGCCGTCGTTTTACAACGTCGTGACTGGGAAAACCC
TGCGAGCTTCCGAAATTAAACGCCGGCCATGGGTTAAGCGGGATATCACTCAGCATAATGCGCGCGAGTGACCGGCAGCAAAATGTTGCAGCACTGACCCTTTTGGG
CYC1 terminator | T7 | M13-fwd
4,400 4,420 4,440 4,460 4,480

TGGCGTTACCCAACTTAATCGCCTTGCAGCACATCCCCCTTTCGCCAGCTGGCGTAATAGCGAAGAGGCCCGCACCGATCGCCCTTCCCAACAGTTGCGCAGCCTGA
ACCGCAATGGGTTGAATTAGCGGAACGTCGTGTAGGGGGAAAGCGGTCGACCGCATTATCGCTTCTCCGGGCGTGGCTAGCGGGAAGGGTTGTCAACGCGTCGGACT
LacZ alpha
4,500 4,520 4,540 4,560 4,580 4,600

ATGGCGAATGGACGCGCCCTGTAGCGGCGCATTAAGCGCGGCGGGTGTGGTGGTTACGCGCAGCGTGACCACTACACTTGCCAGCGCCCTAGCGCCCGCTCCTTTCG
TACCGCTTACCTGCGCGGGACATCGCCGCGTAATTCGCGCCGCCCACACCACCAATGCGCGTCGCACTGGTGATGTGAACGGTCGCGGGATCGCGGGCGAGGAAAGC
La...a
4,620 4,640 4,660 4,680 4,700

CTTTCTTCCCTTCCTTTCTCGCCACGTTCGCCGGCTTTCCCCGTCAAGCTCTAAATCGGGGGCTCCCTTTAGGGTTCCGATTTAGTGCTTTACGGCACCTCGACCCC
GAAAGAAGGGAAGGAAAGAGCGGTGCAAGCGGCCGAAAGGGGCAGTTCGAGATTTAGCCCCCGAGGGAAATCCCAAGGCTAAATCACGAAATGCCGTGGAGCTGGGG
4,720 4,740 4,760 4,780 4,800

AAAAAACTTGATTAGGGTGATGGTTCACGTAGTGGGCCATCGCCCTGATAGACGGTTTTTCGCCCTTTGACGTTGGAGTCCACGTTCTTTAATAGTGGACTCTTGTT
TTTTTTGAACTAATCCCACTACCAAGTGCATCACCCGGTAGCGGGACTATCTGCCAAAAAGCGGGAAACTGCAACCTCAGGTGCAAGAAATTATCACCTGAGAACAA
4,820 4,840 4,860 4,880 4,900 4,920

CCAAACTGGAACAACACTCAACCCTATCTCGGTCTATTCTTTTGATTTATAAGGGATTTTGCCGATTTCGGCCTATTGGTTAAAAAATGAGCTGATTTAACAAAAAT
GGTTTGACCTTGTTGTGAGTTGGGATAGAGCCAGATAAGAAAACTAAATATTCCCTAAAACGGCTAAAGCCGGATAACCAATTTTTTACTCGACTAAATTGTTTTTA
4,940 4,960 4,980 5,000 5,020

TTAACGCGAATTTTAACAAAATATTAACGCTTACAATTTCCTGATGCGGTATTTTCTCCTTACGCATCTGTGCGGTATTTCACACCGCATAGGGTAATAACTGATAT
AATTGCGCTTAAAATTGTTTTATAATTGCGAATGTTAAAGGACTACGCCATAAAAGAGGAATGCGTAGACACGCCATAAAGTGTGGCGTATCCCATTATTGACTATA
5,040 5,060 5,080 5,100 5,120

AATTAAATTGAAGCTCTAATTTGTGAGTTTAGTATACATGCATTTACTTATAATACAGTTTTATGACAGAGCAAAAAGCCCTAGTAAAGCGTATTACAAATGAAACC
TTAATTTAACTTCGAGATTAAACACTCAAATCATATGTACGTAAATGAATATTATGTCAAAATACTGTCTCGTTTTTCGGGATCATTTCGCATAATGTTTACTTTGG
HIS3
5,140 5,160 5,180 5,200 5,220 5,240

AAGATTCAGATTGCGATCTCTTTAAAGGGTGGTCCCCTAGCGCTAGAGCACTCGATCTTCCCAGAAAAAGAGGCAGAAGCAGTAGCAGAACAGGCCACACAATCGCA
TTCTAAGTCTAACGCTAGAGAAATTTCCCACCAGGGGATCGCGATCTCGTGAGCTAGAAGGGTCTTTTTCTCCGTCTTCGTCATCGTCTTGTCCGGTGTGTTAGCGT
HIS3
5,260 5,280 5,300 5,320 5,340

AGTGATTAACGTCCACACAGGTATAGGGTTTCTGGACCATATGATACATGCTCTGGCCAAGCATTCCGGCTGGTCGCTAATCGTTGAGTGCATTGGTGACTTACACA
TCACTAATTGCAGGTGTGTCCATATCCCAAAGACCTGGTATACTATGTACGAGACCGGTTCGTAAGGCCGACCAGCGATTAGCAACTCACGTAACCACTGAATGTGT

HIS3

5,360 5,380 5,400 5,420 5,440

TAGACGACCATCACACCACTGAAGACTGCGGGATTGCTCTCGGTCAAGCTTTTAAAGAGGCCCTAGGGGCCGTGCGTGGAGTAAAAAGGTTTGGATCAGGATTTGCG
ATCTGCTGGTAGTGTGGTGACTTCTGACGCCCTAACGAGAGCCAGTTCGAAAATTTCTCCGGGATCCCCGGCACGCACCTCATTTTTCCAAACCTAGTCCTAAACGC

HIS3

5,460 5,480 5,500 5,520 5,540 5,560

CCTTTGGATGAGGCACTTTCCAGAGCGGTGGTAGATCTTTCGAACAGGCCGTACGCAGTTGTCGAACTTGGTTTGCAAAGGGAGAAAGTAGGAGATCTCTCTTGCGA
GGAAACCTACTCCGTGAAAGGTCTCGCCACCATCTAGAAAGCTTGTCCGGCATGCGTCAACAGCTTGAACCAAACGTTTCCCTCTTTCATCCTCTAGAGAGAACGCT

HIS3

5,580 5,600 5,620 5,640 5,660

GATGATCCCGCATTTTCTTGAAAGCTTTGCAGAGGCTAGCAGAATTACCCTCCACGTTGATTGTCTGCGAGGCAAGAATGATCATCACCGTAGTGAGAGTGCGTTCA
CTACTAGGGCGTAAAAGAACTTTCGAAACGTCTCCGATCGTCTTAATGGGAGGTGCAACTAACAGACGCTCCGTTCTTACTAGTAGTGGCATCACTCTCACGCAAGT

HIS3

5,680 5,700 5,720 5,740 5,760

AGGCTCTTGCGGTTGCCATAAGAGAAGCTACCTCGCCCAATGGTACCAACGATGTTCCCTCCACCAAAGGTGTTCTTATGTAGTAGAATGGTATATCCTTGAAATAT
TCCGAGAACGCCAACGGTATTCTCTTCGATGGAGCGGGTTACCATGGTTGCTACAAGGGAGGTGGTTTCCACAAGAATACATCATCTTACCATATAGGAACTTTATA

HIS3

5,780 5,800 5,820 5,840 5,860 5,880

ATATATATATATNTNGCTGAAATGTAAAAGGTAAGAAAAGTTAGAAAGTAAGACGATTGCTAACCACCTATTGGAAAAAACAATAGGTCCTTAAATAATATTGTCAA
TATATATATATANANCGACTTTACATTTTCCATTCTTTTCAATCTTTCATTCTGCTAACGATTGGTGGATAACCTTTTTTGTTATCCAGGAATTTATTATAACAGTT

5,900 5,920 5,940 5,960 5,980

CTTCAAGTATTGTGATGCAAGCATTTAGTCATGAACGCTTCTCTATTCTATATGAAAAGCCGGTTCCGGCGCTCTCACCTTTCCTTTTTCTCCCAATTTTTCAGTTG
GAAGTTCATAACACTACGTTCGTAAATCAGTACTTGCGAAGAGATAAGATATACTTTTCGGCCAAGGCCGCGAGAGTGGAAAGGAAAAAGAGGGTTAAAAAGTCAAC

6,000 6,020 6,040 6,060 6,080

AAAAAGGTATATGCGTCAGGCGACCTCTGAAATTAACAAAAAATTTCCAGTCATCGAATTTGATTCTGTGCGATAGCGCCCCTGTGTGTTCTCGTTATGTTGAGGAA
TTTTTCCATATACGCAGTCCGCTGGAGACTTTAATTGTTTTTTAAAGGTCAGTAGCTTAAACTAAGACACGCTATCGCGGGGACACACAAGAGCAATACAACTCCTT

6,100 6,120 6,140 6,160 6,180 6,200

AAAAATAATGGTTGCTAAGAGATTCGAACTCTTGCATCTTACGATACCTGAGTATTCCCACAGTTTGAAAAGCTGTGGTATGGTGCACTCTCAGTACAATCTGCTCT
TTTTTATTACCAACGATTCTCTAAGCTTGAGAACGTAGAATGCTATGGACTCATAAGGGTGTCAAACTTTTCGACACCATACCACGTGAGAGTCATGTTAGACGAGA

6,220 6,240 6,260 6,280 6,300

```
GATGCCGCATAGTTAAGCCAGCCCCGACACCCGCCAACACCCGCTGACGCGCCCTGACGGGCTTGTCTGCTCCCGGCATCCGCTTACAGACAAGCTGTGACCGTCTC
CTACGGCGTATCAATTCGGTCGGGGCTGTGGGCGGTTGTGGGCGACTGCGCGGGACTGCCCGAACAGACGAGGGCCGTAGGCGAATGTCTGTTCGACACTGGCAGAG
      6,320               6,340               6,360               6,380               6,400               6,420

CGGGAGCTGCATGTGTCAGAGGTTTTCACCGTCATCACCGAAACGCGCGAGACGAAAGGGCCTCGTGATACGCCTATTTTTATAGGTTAATGTCATGATAATAATGG
GCCCTCGACGTACACAGTCTCCAAAAGTGGCAGTAGTGGCTTTGCGCGCTCTGCTTTCCCGGAGCACTATGCGGATAAAAATATCCAATTACAGTACTATTATTACC
                    6,440               6,460               6,480               6,500               6,520

TTTCTTAGTATGATCCAATATCAAAGGAAATGATAGCATTGAAGGATGAGACTAATCCAATTGAGGAGTGGCAGCATATAGAACAGCTAAAGGGTAGTGCTGAAGGA
AAAGAATCATACTAGGTTATAGTTTCCTTTACTATCGTAACTTCCTACTCTGATTAGGTTAACTCCTCACCGTCGTATATCTTGTCGATTTCCCATCACGACTTCCT
                                            2 micron origin
            6,540               6,560               6,580               6,600               6,620

AGCATACGATACCCCGCATGGAATGGGATAATATCACAGGAGGTACTAGACTACCTTTCATCCTACATAAATAGACGCATATAAGTACGCATTTAAGCATAAACACG
TCGTATGCTATGGGGCGTACCTTACCCTATTATAGTGTCCTCCATGATCTGATGGAAAGTAGGATGTATTTATCTGCGTATATTCATGCGTAAATTCGTATTTGTGC
                                            2 micron origin
     6,640               6,660               6,680               6,700               6,720               6,740

CACTATGCCGTTCTTCTCATGTATATATATATACAGGCAACACGCAGATATAGGTGCGACGTGAACAGTGAGCTGTATGTGCGCAGCTCGCGTTGCATTTTCGGAAG
GTGATACGGCAAGAAGAGTACATATATATATATGTCCGTTGTGCGTCTATATCCACGCTGCACTTGTCACTCGACATACACGCGTCGAGCGCAACGTAAAAGCCTTC
                                            2 micron origin
                  6,760               6,780               6,800               6,820               6,840

CGCTCGTTTTCGGAAACGCTTTGAAGTTCCTATTCCGAAGTTCCTATTCTCTAGAAAGTATAGGAACTTCAGAGCGCTTTTGAAAACCAAAAGCGCTCTGAAGACGC
GCGAGCAAAAGCCTTTGCGAAACTTCAAGGATAAGGCTTCAAGGATAAGAGATCTTTCATATCCTTGAAGTCTCGCGAAAACTTTTGGTTTTCGCGAGACTTCTGCG
                                              FRT
                                            2 micron origin
          6,860               6,880               6,900               6,920               6,940

ACTTTCAAAAAACCAAAAACGCACCGGACTGTAACGAGCTACTAAAATATTGCGAATACCGCTTCCACAAACATTGCTCAAAAGTATCTCTTTGCTATATATCTCTG
TGAAAGTTTTTTGGTTTTTGCGTGGCCTGACATTGCTCGATGATTTTATAACGCTTATGGCGAAGGTGTTTGTAACGAGTTTTCATAGAGAAACGATATATAGAGAC
                                            2 micron origin
    6,960               6,980               7,000               7,020               7,040               7,060

TGCTATATCCCTATATAACCTACCCATCCACCTTTCGCTCCTTGAACTTGCATCTAAACTCGACCTCTACATTTTTTATGTTTATCTCTAGTATTACTCTTTAGACA
ACGATATAGGGATATATTGGATGGGTAGGTGGAAAGCGAGGAACTTGAACGTAGATTTGAGCTGGAGATGTAAAAAATACAAATAGAGATCATAATGAGAAATCTGT
                                            2 micron origin
                 7,080               7,100               7,120               7,140               7,160

AAAAAATTGTAGTAAGAACTATTCATAGAGTGAATCGAAAACAATACGAAAATGTAAACATTTCCTATACGTAGTATATAGAGACAAAATAGAAGAAACCGTTCATA
TTTTTTAACATCATTCTTGATAAGTATCTCACTTAGCTTTTGTTATGCTTTTACATTTGTAAAGGATATGCATCATATATCTCTGTTTTATCTTCTTTGGCAAGTAT
                                            2 micron origin
       7,180               7,200               7,220               7,240               7,260
```

```
ATTTTCTGACCAATGAAGAATCATCAACGCTATCACTTTCTGTTCACAAAGTATGCGCAATCCACATCGGTATAGAATATAATCGGGGATGCCTTTATCTTGAAAAA
TAAAAGACTGGTTACTTCTTAGTAGTTGCGATAGTGAAAGACAAGTGTTTCATACGCGTTAGGTGTAGCCATATCTTATATTAGCCCCTACGGAAATAGAACTTTTT
                                    2 micron origin

7,280          7,300          7,320          7,340          7,360          7,380

ATGCACCCGCAGCTTCGCTAGTAATCAGTAAACGCGGGAAGTGGAGTCAGGCTTTTT
TACGTGGGCGTCGAAGCGATCATTAGTCATTTGCGCCCTTCACCTCAGTCCGAAAAA
                 2 micron origin

   7,390     7,400     7,410     7,420     7,430   7,440
```

ST/SC ligation / pNb_lys Nb-FLAG (pPIC9K) (9814...

AGATCTAACATCCAAAGACGAAAGGTTGAATGAAACCTTTTTGCCATCCGACATCCACAGGTCCATTCTCACACATAAGTGCCAAACGCAACAGGAGGGGATACACT
TCTAGATTGTAGGTTTCTGCTTTCCAACTTACTTTGGAAAAACGGTAGGCTGTAGGTGTCCAGGTAAGAGTGTGTATTCACGGTTTGCGTTGTCCTCCCCTATGTGA
AOX1 promoter
20 40 60 80 100

AGCAGCAGACCGTTGCAAACGCAGGACCTCCACTCCTCTTCTCCTCAACACCCACTTTTGCCATCGAAAAACCAGCCCAGTTATTGGGCTTGATTGGAGCTCGCTCA
TCGTCGTCTGGCAACGTTTGCGTCCTGGAGGTGAGGAGAAGAGGAGTTGTGGGTGAAAACGGTAGCTTTTTGGTCGGGTCAATAACCCGAACTAACCTCGAGCGAGT
AOX1 promoter
120 140 160 180 200

TTCCAATTCCTTCTATTAGGCTACTAACACCATGACTTTATTAGCCTGTCTATCCTGGCCCCCCTGGCGAGGTTCATGTTTGTTTATTTCCGAATGCAACAAGCTCC
AAGGTTAAGGAAGATAATCCGATGATTGTGGTACTGAAATAATCGGACAGATAGGACCGGGGGGACCGCTCCAAGTACAAACAAATAAAGGCTTACGTTGTTCGAGG
AOX1 promoter
220 240 260 280 300 320

GCATTACACCCGAACATCACTCCAGATGAGGGCTTTCTGAGTGTGGGGTCAAATAGTTTCATGTTCCCCAAATGGCCCAAAACTGACAGTTTAAACGCTGTCTTGGA
CGTAATGTGGGCTTGTAGTGAGGTCTACTCCCGAAAGACTCACACCCCAGTTTATCAAAGTACAAGGGGTTTACCGGGTTTTGACTGTCAAATTTGCGACAGAACCT
AOX1 promoter
340 360 380 400 420

ACCTAATATGACAAAAGCGTGATCTCATCCAAGATGAACTAAGTTTGGTTCGTTGAAATGCTAACGGCCAGTTGGTCAAAAAGAAACTTCCAAAAGTCGCCATACCG
TGGATTATACTGTTTTCGCACTAGAGTAGGTTCTACTTGATTCAAACCAAGCAACTTTACGATTGCCGGTCAACCAGTTTTTCTTTGAAGGTTTTCAGCGGTATGGC
AOX1 promoter
440 460 480 500 520

TTTGTCTTGTTTGGTATTGATTGACGAATGCTCAAAAATAATCTCATTAATGCTTAGCGCAGTCTCTCTATCGCTTCTGAACCCCGGTGCACCTGTGCCGAAACGCA
AAACAGAACAAACCATAACTAACTGCTTACGAGTTTTTATTAGAGTAATTACGAATCGCGTCAGAGAGATAGCGAAGACTTGGGGCCACGTGGACACGGCTTTGCGT
AOX1 promoter
540 560 580 600 620 640

AATGGGGAAACACCCGCTTTTTGGATGATTATGCATTGTCTCCACATTGTATGCTTCCAAGATTCTGGTGGGAATACTGCTGATAGCCTAACGTTCATGATCAAAAT
TTACCCCTTTGTGGGCGAAAAACCTACTAATACGTAACAGAGGTGTAACATACGAAGGTTCTAAGACCACCCTTATGACGACTATCGGATTGCAAGTACTAGTTTTA
AOX1 promoter
660 680 700 720 740

TTAACTGTTCTAACCCCTACTTGACAGCAATATATAAACAGAAGGAAGCTGCCCTGTCTTAAACCTTTTTTTTTATCATCATTATTAGCTTACTTTCATAATTGCGA
AATTGACAAGATTGGGGATGAACTGTCGTTATATATTTGTCTTCCTTCGACGGGACAGAATTTGGAAAAAAAAATAGTAGTAATAATCGAATGAAAGTATTAACGCT
AOX1 promoter
760 780 800 820 840

CTGGTTCCAATTGACAAGCTTTTGATTTTAACGACTTTTAACGACAACTTGAGAAGATCAAAAAACAACTAATTATTCGAAATGAGATTCCCATCTATCTTCACCGC
GACCAAGGTTAACTGTTCGAAAACTAAAATTGCTGAAAATTGCTGTTGAACTCTTCTAGTTTTTTGTTGATTAATAAGCTTTACTCTAAGGGTAGATAGAAGTGGCG
1 2 3 4 5 6 7 8 9
M R F P S I F T A
AOX1 promoter
Mating fac...ha leader
860 880 900 920 940 960

TGTTTTGTTCGCTGCTTCTTCTGCTTTGGCTGCTCCAGCTAACACCACCACCGAAGACGAAACCGCTCAAATCCCAGCTGAAGCTGTTATCGACTACTCTGACTTGG
ACAAAACAAGCGACGAAGAAGACGAAACCGACGAGGTCGATTGTGGTGGTGGCTTCTGCTTTGGCGAGTTTAGGGTCGACTTCGACAATAGCTGATGAGACTGAACC
10 11 12 13 14 15 16 17 18 19 20 21 22 23 24 25 26 27 28 29 30 31 32 33 34 35 36 37 38 39 40 41 42 43 44
V L F A A S S A L A A P A N T T T E D E T A Q I P A E A V I D Y S D L
Mating factor alpha leader
980 1,000 1,020 1,040 1,060

AAGGTGACTTCGACGCTGCTGCTTTGCCATTGTCTAACTCTACCAACAACGGTTTGTCTTCTACCAACACCACCATCGCTTCTATCGCTGCTAAGGAAGAAGGTGTT
TTCCACTGAAGCTGCGACGACGAAACGGTAACAGATTGAGATGGTTGTTGCCAAACAGAAGATGGTTGTGGTGGTAGCGAAGATAGCGACGATTCCTTCTTCCACAA
45 46 47 48 49 50 51 52 53 54 55 56 57 58 59 60 61 62 63 64 65 66 67 68 69 70 71 72 73 74 75 76 77 78 79 80
E G D F D A A A L P L S N S T N N G L S S T N T T I A S I A A K E E G V
Mating factor alpha leader
1,080 1,100 1,120 1,140 1,160

CAATTGGACAAGAGAGAAagatctggcggccgcgcatgcgctagcctcgagGATGTGCAGTTGCAAGCATCCGGCGGCGGTTCTGTTCAGGCTGGTGGTTCTCTAAG
GTTAACCTGTTCTCTCTTtctagaccgccggcgcgtacgcgatcggagctcCTACACGTCAACGTTCGTAGGCCGCCGCCAAGACAAGTCCGACCACCAAGAGATTC
81 82 83 84 85 86
Q L D K R E
2 4 6 8 10 12 14 16 18
D V Q L Q A S G G G S V Q A G G S L R
Mating...eader
anti-lysozyme nanobody
1,180 1,200 1,220 1,240 1,260 1,280

ATTAAGTTGTGCTGCTTCAGGTTATACCATCGGCCCATACTGCATGGGTTGGTTTCGTCAAGCTCCAGGCAAAGAAAGGGAGGGAGTCGCAGCCATTAACATGGGCG
TAATTCAACACGACGAAGTCCAATATGGTAGCCGGGTATGACGTACCCAACCAAAGCAGTTCGAGGTCCGTTTCTTTCCCTCCCTCAGCGTCGGTAATTGTACCCGC
20 22 24 26 28 30 32 34 36 38 40 42 44 46 48 50 52 54
L S C A A S G Y T I G P Y C M G W F R Q A P G K E R E G V A A I N M G
anti-lysozyme nanobody
1,300 1,320 1,340 1,360 1,380

GCGGTATCACATATTATGCAGACTCCGTAAAAGGTAGATTTACTATCTCTCAGGACAATGCTAAGAACACGGTCTATCTGTTAATGAATTCATTGGAGCCCGAGGAC
CGCCATAGTGTATAATACGTCTGAGGCATTTTCCATCTAAATGATAGAGAGTCCTGTTACGATTCTTGTGCCAGATAGACAATTACTTAAGTAACCTCGGGCTCCTG
56 58 60 62 64 66 68 70 72 74 76 78 80 82 84 86 88 90
G G I T Y Y A D S V K G R F T I S Q D N A K N T V Y L L M N S L E P E D
anti-lysozyme nanobody
1,400 1,420 1,440 1,460 1,480

ACTGCCATATACTACTGTGCCGCAGATTCAACCATTTACGCTTCCTATTACGAATGCGGACACGGATTAAGTACTGGAGGTTACGGTTATGATTCCTGGGGTCAAGG
TGACGGTATATGATGACACGGCGTCTAAGTTGGTAAATGCGAAGGATAATGCTTACGCCTGTGCCTAATTCATGACCTCCAATGCCAATACTAAGGACCCCAGTTCC
92 94 96 98 100 102 104 106 108 110 112 114 116 118 120 122 124 126
T A I Y Y C A A D S T I Y A S Y Y E C G H G L S T G G Y G Y D S W G Q G
anti-lysozyme nanobody
1,500 1,520 1,540 1,560 1,580 1,600

CACACAAGTCACGGTATCATCAGGCGGATCAGACTATAAGGATGACGACGATAAGGCTGGAGGTTCGAGGGGAGTCCCTCACATAGTGATGGTGGATGCTTACAAAA
GTGTGTTCAGTGCCATAGTAGTCCGCCTAGTCTGATATTCCTACTGCTGCTATTCCGACCTCCAAGCTCCCCTCAGGGAGTGTATCACTACCACCTACGAATGTTTT
128 130 132 1 2 3 1 2 3 4 5 6 7 8 1 2 3 4 1 2 3 4 5 6 7 8 9 10 11 12 13
T Q V T V S S G G S D Y K D D D D K A G G S R G V P H I V M V D A Y K
anti-lys...anobody FLAG tag SpyTag
1,620 1,640 1,660 1,680 1,700

GGTATAAATAAGGTACCGCGATGTAGTAAAACTAGCTAGACTACGTAGAATTCCCTAGGGCGGCCGCGAATTAATTCGCCTTAGACATGACTGTTCCTCAGTTCAAG
CCATATTTATTCCATGGCGCTACATCATTTTGATCGATCTGATGCATCTTAAGGGATCCCGCCGGCGCTTAATTAAGCGGAATCTGTACTGACAAGGAGTCAAGTTC
14 15 16 1
R Y K *
1,720 1,740 1,760 1,780 1,800

TTGGGCACTTACGAGAAGACCGGTCTTGCTAGATTCTAATCAAGAGGATGTCAGAATGCCATTTGCCTGAGAGATGCAGGCTTCATTTTTGATACTTTTTTATTTGT
AACCCGTGAATGCTCTTCTGGCCAGAACGATCTAAGATTAGTTCTCCTACAGTCTTACGGTAAACGGACTCTCTACGTCCGAAGTAAAAACTATGAAAAAATAAACA
AOX1 teminator
1,820 1,840 1,860 1,880 1,900 1,920

AACCTATATAGTATAGGATTTTTTTTGTCATTTTGTTTCTTCTCGTACGAGCTTGCTCCTGATCAGCCTATCTCGCAGCTGATGAATATCTTGTGGTAGGGGTTTGG
TTGGATATATCATATCCTAAAAAAAACAGTAAAACAAAGAAGAGCATGCTCGAACGAGGACTAGTCGGATAGAGCGTCGACTACTTATAGAACACCATCCCCAAACC
AOX1 teminator
1,940 1,960 1,980 2,000 2,020

GAAAATCATTCGAGTTTGATGTTTTTCTTGGTATTTCCCACTCCTCTTCAGAGTACAGAAGATTAAGTGAGACGTTCGTTTGTGCAAGCTTATCGATAAGCTTTAAT
CTTTTAGTAAGCTCAAACTACAAAAAGAACCATAAAGGGTGAGGAGAAGTCTCATGTCTTCTAATTCACTCTGCAAGCAAACACGTTCGAATAGCTATTCGAAATTA
AOX1 teminator
2,040 2,060 2,080 2,100 2,120 2,140

GCGGTAGTTTATCACAGTTAAATTGCTAACGCAGTCAGGCACCGTGTATGAAATCTAACAATGCGCTCATCGTCATCCTCGGCACCGTCACCCTGGATGCTGTAGGC
CGCCATCAAATAGTGTCAATTTAACGATTGCGTCAGTCCGTGGCACATACTTTAGATTGTTACGCGAGTAGCAGTAGGAGCCGTGGCAGTGGGACCTACGACATCCG
2,160 2,180 2,200 2,220 2,240

ATAGGCTTGGTTATGCCGGTACTGCCGGGCCTCTTGCGGGATATCGTCCATTCCGACAGCATCGCCAGTCACTATGGCGTGCTGCTAGCGCTATATGCGTTGATGCA
TATCCGAACCAATACGGCCATGACGGCCCGGAGAACGCCCTATAGCAGGTAAGGCTGTCGTAGCGGTCAGTGATACCGCACGACGATCGCGATATACGCAACTACGT
2,260 2,280 2,300 2,320 2,340

ATTTCTATGCGCACCCGTTCTCGGAGCACTGTCCGACCGCTTTGGCCGCCGCCCAGTCCTGCTCGCTTCGCTACTTGGAGCCACTATCGACTACGCGATCATGGCGA
TAAAGATACGCGTGGGCAAGAGCCTCGTGACAGGCTGGCGAAACCGGCGGCGGGTCAGGACGAGCGAAGCGATGAACCTCGGTGATAGCTGATGCGCTAGTACCGCT

2,360 2,380 2,400 2,420 2,440 2,460

CCACACCCGTCCTGTGGATCTATCGAATCTAAATGTAAGTTAAAATCTCTAAATAATTAAATAAGTCCCAGTTTCTCCATACGAACCTTAACAGCATTGCGGTGAGC
GGTGTGGGCAGGACACCTAGATAGCTTAGATTTACATTCAATTTTAGAGATTTATTAATTTATTCAGGGTCAAAGAGGTATGCTTGGAATTGTCGTAACGCCACTCG
PpHIS4

2,480 2,500 2,520 2,540 2,560

ATCTAGACCTTCAACAGCAGCCAGATCCATCACTGCTTGGCCAATATGTTTCAGTCCCTCAGGAGTTACGTCTTGTGAAGTGATGAACTTCTGGAAGGTTGCAGTGT
TAGATCTGGAAGTTGTCGTCGGTCTAGGTAGTGACGAACCGGTTATACAAAGTCAGGGAGTCCTCAATGCAGAACACTTCACTACTTGAAGACCTTCCAACGTCACA
PpHIS4

2,580 2,600 2,620 2,640 2,660

TAACTCCGCTGTATTGACGGGCATATCCGTACGTTGGCAAAGTGTGGTTGGTACCGGAGGAGTAATCTCCACAACTCTCTGGAGAGTAGGCACCAACAAACACAGAT
ATTGAGGCGACATAACTGCCCGTATAGGCATGCAACCGTTTCACACCAACCATGGCCTCCTCATTAGAGGTGTTGAGAGACCTCTCATCCGTGGTTGTTTGTGTCTA
PpHIS4

2,680 2,700 2,720 2,740 2,760 2,780

CCAGCGTGTTGTACTTGATCAACATAAGAAGAAGCATTCTCGATTTGCAGGATCAAGTGTTCAGGAGCGTACTGATTGGACATTTCCAAAGCCTGCTCGTAGGTTGC
GGTCGCACAACATGAACTAGTTGTATTCTTCTTCGTAAGAGCTAAACGTCCTAGTTCACAAGTCCTCGCATGACTAACCTGTAAAGGTTTCGGACGAGCATCCAACG
PpHIS4

2,800 2,820 2,840 2,860 2,880

AACCGATAGGGTTGTAGAGTGTGCAATACACTTGCGTACAATTTCAACCCTTGGCAACTGCACAGCTTGGTTGTGAACAGCATCTTCAATTCTGGCAAGCTCCTTGT
TTGGCTATCCCAACATCTCACACGTTATGTGAACGCATGTTAAAGTTGGGAACCGTTGACGTGTCGAACCAACACTTGTCGTAGAAGTTAAGACCGTTCGAGGAACA
PpHIS4

2,900 2,920 2,940 2,960 2,980

CTGTCATATCGACAGCCAACAGAATCACCTGGGAATCAATACCATGTTCAGCTTGAGACAGAAGGTCTGAGGCAACGAAATCTGGATCAGCGTATTTATCAGCAATA
GACAGTATAGCTGTCGGTTGTCTTAGTGGACCCTTAGTTATGGTACAAGTCGAACTCTGTCTTCCAGACTCCGTTGCTTTAGACCTAGTCGCATAAATAGTCGTTAT
PpHIS4

3,000 3,020 3,040 3,060 3,080 3,100

ACTAGAACTTCAGAAGGCCCAGCAGGCATGTCAATACTACACAGGGCTGATGTGTCATTTTGAACCATCATCTTGGCAGCAGTAACGAACTGGTTTCCTGGACCAAA
TGATCTTGAAGTCTTCCGGGTCGTCCGTACAGTTATGATGTGTCCCGACTACACAGTAAAACTTGGTAGTAGAACCGTCGTCATTGCTTGACCAAAGGACCTGGTTT
PpHIS4

3,120 3,140 3,160 3,180 3,200

TATTTTGTCACACTTAGGAACAGTTTCTGTTCCGTAAGCCATAGCAGCTACTGCCTGGGCGCCTCCTGCTAGCACGATACACTTAGCACCAACCTTGTGGGCAACGT
ATAAAACAGTGTGAATCCTTGTCAAAGACAAGGCATTCGGTATCGTCGATGACGGACCCGCGGAGGACGATCGTGCTATGTGAATCGTGGTTGGAACACCCGTTGCA
PpHIS4

3,220 3,240 3,260 3,280 3,300

AGATGACTTCTGGGGTAAGGGTACCATCCTTCTTAGGTGGAGATGCAAAAACAATTTCTTTGCAACCAGCAACTTTGGCAGGAACACCCAGCATCAGGGAAGTGGAA
TCTACTGAAGACCCCATTCCCATGGTAGGAAGAATCCACCTCTACGTTTTTGTTAAAGAAACGTTGGTCGTTGAAACCGTCCTTGTGGGTCGTAGTCCCTTCACCTT
PpHIS4
3,320 3,340 3,360 3,380 3,400 3,420

GGCAGAATTGCGGTTCCACCAGGAATATAGAGGCCAACTTTCTCAATAGGTCTTGCAAAACGAGAGCAGACTACACCAGGGCAAGTCTCAACTTGCAACGTCTCCGT
CCGTCTTAACGCCAAGGTGGTCCTTATATCTCCGGTTGAAAGAGTTATCCAGAACGTTTTGCTCTCGTCTGATGTGGTCCCGTTCAGAGTTGAACGTTGCAGAGGCA
PpHIS4
3,440 3,460 3,480 3,500 3,520

TAGTTGAGCTTCATGGAATTTCCTGACGTTATCTATAGAGAGATCAATGGCTCTCTTAACGTTATCTGGCAATTGCATAAGTTCCTCTGGGAAAGGAGCTTCTAACA
ATCAACTCGAAGTACCTTAAAGGACTGCAATAGATATCTCTCTAGTTACCGAGAGAATTGCAATAGACCGTTAACGTATTCAAGGAGACCCTTTCCTCGAAGATTGT
PpHIS4
3,540 3,560 3,580 3,600 3,620

CAGGTGTCTTCAAAGCGACTCCATCAAACTTGGCAGTTAGTTCTAAAAGGGCTTTGTCACCATTTTGACGAACATTGTCGACAATTGGTTTGACTAATTCCATAATC
GTCCACAGAAGTTTCGCTGAGGTAGTTTGAACCGTCAATCAAGATTTTCCCGAAACAGTGGTAAAACTGCTTGTAACAGCTGTTAACCAAACTGATTAAGGTATTAG
PpHIS4
3,640 3,660 3,680 3,700 3,720 3,740

TGTTCCGTTTTCTGGATAGGACGACGAAGGGCATCTTCAATTTCTTGTGAGGAGGCCTTAGAAACGTCAATTTTGCACAATTCAATACGACCTTCAGAAGGGACTTC
ACAAGGCAAAAGACCTATCCTGCTGCTTCCCGTAGAAGTTAAAGAACACTCCTCCGGAATCTTTGCAGTTAAAACGTGTTAAGTTATGCTGGAAGTCTTCCCTGAAG
PpHIS4
3,760 3,780 3,800 3,820 3,840

TTTAGGTTTGGATTCTTCTTTAGGTTGTTCCTTGGTGTATCCTGGCTTGGCATCTCCTTTCCTTCTAGTGACCTTTAGGGACTTCATATCCAGGTTTCTCTCCACCT
AAATCCAAACCTAAGAAGAAATCCAACAAGGAACCACATAGGACCGAACCGTAGAGGAAAGGAAGATCACTGGAAATCCCTGAAGTATAGGTCCAAAGAGAGGTGGA
PpHIS4
3,860 3,880 3,900 3,920 3,940

CGTCCAACGTCACACCGTACTTGGCACATCTAACTAATGCAAAATAAAATAAGTCAGCACATTCCCAGGCTATATCTTCCTTGGATTTAGCTTCTGCAAGTTCATCA
GCAGGTTGCAGTGTGGCATGAACCGTGTAGATTGATTACGTTTTATTTTATTCAGTCGTGTAAGGGTCCGATATAGAAGGAACCTAAATCGAAGACGTTCAAGTAGT
PpHIS4
3,960 3,980 4,000 4,020 4,040 4,060

GCTTCCTCCCTAATTTTAGCGTTCAACAAAACTTCGTCGTCAAATAACCGTTTGGTATAAGAACCTTCTGGAGCATTGCTCTTACGATCCCACAAGGTGGCTTCCAT
CGAAGGAGGGATTAAAATCGCAAGTTGTTTTGAAGCAGCAGTTTATTGGCAAACCATATTCTTGGAAGACCTCGTAACGAGAATGCTAGGGTGTTCCACCGAAGGTA
PpHIS4
4,080 4,100 4,120 4,140 4,160

```
GGCTCTAAGACCCTTTGATTGGCCAAAACAGGAAGTGCGTTCCAAGTGACAGAAACCAACACCTGTTTGTTCAACCACAAATTTCAAGCAGTCTCCATCACAATCCA
CCGAGATTCTGGGAAACTAACCGGTTTTGTCCTTCACGCAAGGTTCACTGTCTTTGGTTGTGGACAAACAAGTTGGTGTTTAAAGTTCGTCAGAGGTAGTGTTAGGT
PpHIS4
4,180 4,200 4,220 4,240 4,260 4,280

ATTCGATACCCAGCAACTTTTGAGTTGCTCCAGATGTAGCACCTTTATACCACAAACCGTGACGACGAGATTGGTAGACTCCAGTTTGTGTCCTTATAGCCTCCGGA
TAAGCTATGGGTCGTTGAAAACTCAACGAGGTCTACATCGTGGAAATATGGTGTTTGGCACTGCTGCTCTAACCATCTGAGGTCAAACACAGGAATATCGGAGGCCT
PpHIS4
4,300 4,320 4,340 4,360 4,380

ATAGACTTTTTGGACGAGTACACCAGGCCCAACGAGTAATTAGAAGAGTCAGCCACCAAAGTAGTGAATAGACCATCGGGGCGGTCAGTAGTCAAAGACGCCAACAA
TATCTGAAAAACCTGCTCATGTGGTCCGGGTTGCTCATTAATCTTCTCAGTCGGTGGTTTCATCACTTATCTGGTAGCCCCGCCAGTCATCAGTTTCTGCGGTTGTT
PpHIS4
4,400 4,420 4,440 4,460 4,480

AATTTCACTGACAGGGAACTTTTTGACATCTTCAGAAAGTTCGTATTCAGTAGTCAATTGCCGAGCATCAATAATGGGGATTATACCAGAAGCAACAGTGGAAGTCA
TTAAAGTGACTGTCCCTTGAAAAACTGTAGAAGTCTTTCAAGCATAAGTCATCAGTTAACGGCTCGTAGTTATTACCCCTAATATGGTCTTCGTTGTCACCTTCAGT
PpHIS4
4,500 4,520 4,540 4,560 4,580 4,600

CATCTACCAACTTTGCGGTCTCAGAAAAAGCATAAACAGTTCTACTACCGCCATTAGTGAAACTTTTCAAATCGCCCAGTGGAGAAGAAAAAGGCACAGCGATACTA
GTAGATGGTTGAAACGCCAGAGTCTTTTTCGTATTTGTCAAGATGATGGCGGTAATCACTTTGAAAAGTTTAGCGGGTCACCTCTTCTTTTTCCGTGTCGCTATGAT
PpHIS4
4,620 4,640 4,660 4,680 4,700

GCATTAGCGGGCAAGGATGCAACTTTATCAACCAGGGTCCTATAGATAACCCTAGCGCCTGGGATCATCCTTTGGACAACTCTTTCTGCCAAATCTAGGTCCAAAAT
CGTAATCGCCCGTTCCTACGTTGAAATAGTTGGTCCCAGGATATCTATTGGGATCGCGGACCCTAGTAGGAAACCTGTTGAGAAAGACGGTTTAGATCCAGGTTTTA
PpHIS4
4,720 4,740 4,760 4,780 4,800

CACTTCATTGATACCATTATTGTACAACTTGAGCAAGTTGTCGATCAGCTCCTCAAATTGGTCCTCTGTAACGGATGACTCAACTTGCACATTAACTTGAAGCTCAG
GTGAAGTAACTATGGTAATAACATGTTGAACTCGTTCAACAGCTAGTCGAGGAGTTTAACCAGGAGACATTGCCTACTGAGTTGAACGTGTAATTGAACTTCGAGTC
PpHIS4
4,820 4,840 4,860 4,880 4,900 4,920

TCGATTGAGTGAACTTGATCAGGTTGTGCAGCTGGTCAGCAGCATAGGGAAACACGGCTTTTCCTACCAAACTCAAGGAATTATCAAACTCTGCAACACTTGCGTAT
AGCTAACTCACTTGAACTAGTCCAACACGTCGACCAGTCGTCGTATCCCTTTGTGCCGAAAAGGATGGTTTGAGTTCCTTAATAGTTTGAGACGTTGTGAACGCATA
PpHIS4
4,940 4,960 4,980 5,000 5,020
```

GCAGGTAGCAAGGGAAATGTCATACTTGAAGTCGGACAGTGAGTGTAGTCTTGAGAAATTCTGAAGCCGTATTTTTATTATCAGTGAGTCAGTCATCAGGAGATCCT
CGTCCATCGTTCCCTTTACAGTATGAACTTCAGCCTGTCACTCACATCAGAACTCTTTAAGACTTCGGCATAAAAATAATAGTCACTCAGTCAGTAGTCCTCTAGGA
PpHIS4
5,040 5,060 5,080 5,100 5,120

CTACGCCGGACGCATCGTGGCCGACCTGCAGGGGGGGGGGGGGGGCGCTGAGGTCTGCCTCGTGAAGAAGGTGTTGCTGACTCATACCAGGCCTGAATCGCCCCATCAT
GATGCGGCCTGCGTAGCACCGGCTGGACGTCCCCCCCCCCCCCCCGCGACTCCAGACGGAGCACTTCTTCCACAACGACTGAGTATGGTCCGGACTTAGCGGGGTAGTA
5,140 5,160 5,180 5,200 5,220 5,240

CCAGCCAGAAAGTGAGGGAGCCACGGTTGATGAGAGCTTTGTTGTAGGTGGACCAGTTGGTGATTTTGAACTTTTGCTTTGCCACGGAACGGTCTGCGTTGTCGGGA
GGTCGGTCTTTCACTCCCTCGGTGCCAACTACTCTCGAAACAACATCCACCTGGTCAACCACTAAAACTTGAAAACGAAACGGTGCCTTGCCAGACGCAACAGCCCT
5,260 5,280 5,300 5,320 5,340

AGATGCGTGATCTGATCCTTCAACTCAGCAAAAGTTCGATTTATTCAACAAAGCCGCCGTCCCGTCAAGTCAGCGTAATGCTCTGCCAGTGTTACAACCAATTAACC
TCTACGCACTAGACTAGGAAGTTGAGTCGTTTTCAAGCTAAATAAGTTGTTTCGGCGGCAGGGCAGTTCAGTCGCATTACGAGACGGTCACAATGTTGGTTAATTGG
5,360 5,380 5,400 5,420 5,440

AATTCTGATTAGAAAAACTCATCGAGCATCAAATGAAACTGCAATTTATTCATATCAGGATTATCAATACCATATTTTTGAAAAAGCCGTTTCTGTAATGAAGGAGA
TTAAGACTAATCTTTTTGAGTAGCTCGTAGTTTACTTTGACGTTAAATAAGTATAGTCCTAATAGTTATGGTATAAAAACTTTTTCGGCAAAGACATTACTTCCTCT
KanR
5,460 5,480 5,500 5,520 5,540 5,560

AAACTCACCGAGGCAGTTCCATAGGATGGCAAGATCCTGGTATCGGTCTGCGATTCCGACTCGTCCAACATCAATACAACCTATTAATTTCCCCTCGTCAAAAATAA
TTTGAGTGGCTCCGTCAAGGTATCCTACCGTTCTAGGACCATAGCCAGACGCTAAGGCTGAGCAGGTTGTAGTTATGTTGGATAATTAAAGGGGAGCAGTTTTTATT
KanR
5,580 5,600 5,620 5,640 5,660

GGTTATCAAGTGAGAAATCACCATGAGTGACGACTGAATCCGGTGAGAATGGCAAAAGCTTATGCATTTCTTTCCAGACTTGTTCAACAGGCCAGCCATTACGCTCG
CCAATAGTTCACTCTTTAGTGGTACTCACTGCTGACTTAGGCCACTCTTACCGTTTTCGAATACGTAAAGAAAGGTCTGAACAAGTTGTCCGGTCGGTAATGCGAGC
KanR
5,680 5,700 5,720 5,740 5,760

TCATCAAAATCACTCGCATCAACCAAACCGTTATTCATTCGTGATTGCGCCTGAGCGAGACGAAATACGCGATCGCTGTTAAAAGGACAATTACAAACAGGAATCGA
AGTAGTTTTAGTGAGCGTAGTTGGTTTGGCAATAAGTAAGCACTAACGCGGACTCGCTCTGCTTTATGCGCTAGCGACAATTTTCCTGTTAATGTTTGTCCTTAGCT
KanR
5,780 5,800 5,820 5,840 5,860 5,880

ATGCAACCGGCGCAGGAACACTGCCAGCGCATCAACAATATTTTCACCTGAATCAGGATATTCTTCTAATACCTGGAATGCTGTTTTCCCGGGGATCGCAGTGGTGA
TACGTTGGCCGCGTCCTTGTGACGGTCGCGTAGTTGTTATAAAAGTGGACTTAGTCCTATAAGAAGATTATGGACCTTACGACAAAAGGGCCCCTAGCGTCACCACT
KanR
5,900 5,920 5,940 5,960 5,980

GTAACCATGCATCATCAGGAGTACGGATAAAATGCTTGATGGTCGGAAGAGGCATAAATTCCGTCAGCCAGTTTAGTCTGACCATCTCATCTGTAACATCATTGGCA
CATTGGTACGTAGTAGTCCTCATGCCTATTTTACGAACTACCAGCCTTCTCCGTATTTAAGGCAGTCGGTCAAATCAGACTGGTAGAGTAGACATTGTAGTAACCGT
KanR
6,000 6,020 6,040 6,060 6,080

ACGCTACCTTTGCCATGTTTCAGAAACAACTCTGGCGCATCGGGCTTCCCATACAATCGATAGATTGTCGCACCTGATTGCCCGACATTATCGCGAGCCCATTTATA
TGCGATGGAAACGGTACAAAGTCTTTGTTGAGACCGCGTAGCCCGAAGGGTATGTTAGCTATCTAACAGCGTGGACTAACGGGCTGTAATAGCGCTCGGGTAAATAT
KanR
6,100 6,120 6,140 6,160 6,180 6,200

CCCATATAAATCAGCATCCATGTTGGAATTTAATCGCGGCCTCGAGCAAGACGTTTCCCGTTGAATATGGCTCATAACACCCCTTGTATTACTGTTTATGTAAGCAG
GGGTATATTTAGTCGTAGGTACAACCTTAAATTAGCGCCGGAGCTCGTTCTGCAAAGGGCAACTTATACCGAGTATTGTGGGGAACATAATGACAAATACATTCGTC
KanR
6,220 6,240 6,260 6,280 6,300

ACAGTTTTATTGTTCATGATGATATATTTTTATCTTGTGCAATGTAACATCAGAGATTTTGAGACACAACGTGGCTTTCCCCCCCCCCCCTGCAGGTCGGCATCACC
TGTCAAAATAACAAGTACTACTATATAAAAATAGAACACGTTACATTGTAGTCTCTAAAACTCTGTGTTGCACCGAAAGGGGGGGGGGGGACGTCCAGCCGTAGTGG
6,320 6,340 6,360 6,380 6,400 6,420

GGCGCCACAGGTGCGGTTGCTGGCGCCTATATCGCCGACATCACCGATGGGGAAGATCGGGCTCGCCACTTCGGGCTCATGAGCGCTTGTTTCGGCGTGGGTATGGT
CCGCGGTGTCCACGCCAACGACCGCGGATATAGCGGCTGTAGTGGCTACCCCTTCTAGCCCGAGCGGTGAAGCCCGAGTACTCGCGAACAAAGCCGCACCCATACCA
6,440 6,460 6,480 6,500 6,520

GGCAGGCCCCGTGGCCGGGGGACTGTTGGGCGCCATCTCCTTGCATGCACCATTCCTTGCGGCGGCGGTGCTCAACGGCCTCAACCTACTACTGGGCTGCTTCCTAA
CCGTCCGGGGCACCGGCCCCCTGACAACCCGCGGTAGAGGAACGTACGTGGTAAGGAACGCCGCCGCCACGAGTTGCCGGAGTTGGATGATGACCCGACGAAGGATT
6,540 6,560 6,580 6,600 6,620

TGCAGGAGTCGCATAAGGGAGAGCGTCGAGTATCTATGATTGGAAGTATGGGAATGGTGATACCCGCATTCTTCAGTGTCTTGAGGTCTCCTATCAGATTATGCCCA
ACGTCCTCAGCGTATTCCCTCTCGCAGCTCATAGATACTAACCTTCATACCCTTACCACTATGGGCGTAAGAAGTCACAGAACTCCAGAGGATAGTCTAATACGGGT
6,640 6,660 6,680 6,700 6,720 6,740

ACTAAAGCAACCGGAGGAGGAGATTTCATGGTAAATTTCTCTGACTTTTGGTCATCAGTAGACTCGAACTGTGAGACTATCTCGGTTATGACAGCAGAAATGTCCTT
TGATTTCGTTGGCCTCCTCCTCTAAAGTACCATTTAAAGAGACTGAAAACCAGTAGTCATCTGAGCTTGACACTCTGATAGAGCCAATACTGTCGTCTTTACAGGAA
6,760 6,780 6,800 6,820 6,840

CTTGGAGACAGTAAATGAAGTCCCACCAATAAAGAAATCCTTGTTATCAGGAACAAACTTCTTGTTTCGAACTTTTTCGGTGCCTTGAACTATAAAATGTAGAGTGG
GAACCTCTGTCATTTACTTCAGGGTGGTTATTTCTTTAGGAACAATAGTCCTTGTTTGAAGAACAAAGCTTGAAAAAGCCACGGAACTTGATATTTTACATCTCACC
6,860 6,880 6,900 6,920 6,940

ATATGTCGGGTAGGAATGGAGCGGGCAAATGCTTACCTTCTGGACCTTCAAGAGGTATGTAGGGTTTGTAGATACTGATGCCAACTTCAGTGACAACGTTGCTATTT
TATACAGCCCATCCTTACCTCGCCCGTTTACGAATGGAAGACCTGGAAGTTCTCCATACATCCCAAACATCTATGACTACGGTTGAAGTCACTGTTGCAACGATAAA
6,960 6,980 7,000 7,020 7,040 7,060

CGTTCAAACCATTCCGAATCCAGAGAAATCAAAGTTGTTTGTCTACTATTGATCCAAGCCAGTGCGGTCTTGAAACTGACAATAGTGTGCTCGTGTTTTGAGGTCAT
GCAAGTTTGGTAAGGCTTAGGTCTCTTTAGTTTCAACAAACAGATGATAACTAGGTTCGGTCACGCCAGAACTTTGACTGTTATCACACGAGCACAAAACTCCAGTA

7,080 7,100 7,120 7,140 7,160

CTTTGTATGAATAAATCTAGTCTTTGATCTAAATAATCTTGACGAGCCAAGGCGATAAATACCCAAATCTAAAACTCTTTTAAAACGTTAAAAGGACAAGTATGTCT
GAAACATACTTATTTAGATCAGAAACTAGATTTATTAGAACTGCTCGGTTCCGCTATTTATGGGTTTAGATTTTGAGAAAATTTTGCAATTTTCCTGTTCATACAGA

7,180 7,200 7,220 7,240 7,260

GCCTGTATTAAACCCCAAATCAGCTCGTAGTCTGATCCTCATCAACTTGAGGGGCACTATCTTGTTTTAGAGAAATTTGCGGAGATGCGATATCGAGAAAAAGGTAC
CGGACATAATTTGGGGTTTAGTCGAGCATCAGACTAGGAGTAGTTGAACTCCCCGTGATAGAACAAAATCTCTTTAAACGCCTCTACGCTATAGCTCTTTTTCCATG

7,280 7,300 7,320 7,340 7,360 7,380

GCTGATTTTAAACGTGAAATTTATCTCAAGATCTCTGCCTCGCGCGTTTCGGTGATGACGGTGAAAACCTCTGACACATGCAGCTCCCGGAGACGGTCACAGCTTGT
CGACTAAAATTTGCACTTTAAATAGAGTTCTAGAGACGGAGCGCGCAAAGCCACTACTGCCACTTTTGGAGACTGTGTACGTCGAGGGCCTCTGCCAGTGTCGAACA

7,400 7,420 7,440 7,460 7,480

CTGTAAGCGGATGCCGGGAGCAGACAAGCCCGTCAGGGCGCGTCAGCGGGTGTTGGCGGGTGTCGGGGCGCAGCCATGACCCAGTCACGTAGCGATAGCGGAGTGTA
GACATTCGCCTACGGCCCTCGTCTGTTCGGGCAGTCCCGCGCAGTCGCCCACAACCGCCCACAGCCCCGCGTCGGTACTGGGTCAGTGCATCGCTATCGCCTCACAT

7,500 7,520 7,540 7,560 7,580

TACTGGCTTAACTATGCGGCATCAGAGCAGATTGTACTGAGAGTGCACCATATGCGGTGTGAAATACCGCACAGATGCGTAAGGAGAAAATACCGCATCAGGCGCTC
ATGACCGAATTGATACGCCGTAGTCTCGTCTAACATGACTCTCACGTGGTATACGCCACACTTTATGGCGTGTCTACGCATTCCTCTTTTATGGCGTAGTCCGCGAG

7,600 7,620 7,640 7,660 7,680 7,700

TTCCGCTTCCTCGCTCACTGACTCGCTGCGCTCGGTCGTTCGGCTGCGGCGAGCGGTATCAGCTCACTCAAAGGCGGTAATACGGTTATCCACAGAATCAGGGGATA
AAGGCGAAGGAGCGAGTGACTGAGCGACGCGAGCCAGCAAGCCGACGCCGCTCGCCATAGTCGAGTGAGTTTCCGCCATTATGCCAATAGGTGTCTTAGTCCCCTAT

7,720 7,740 7,760 7,780 7,800

ACGCAGGAAAGAACATGTGAGCAAAAGGCCAGCAAAAGGCCAGGAACCGTAAAAAGGCCGCGTTGCTGGCGTTTTTCCATAGGCTCCGCCCCCCTGACGAGCATCAC
TGCGTCCTTTCTTGTACACTCGTTTTCCGGTCGTTTTCCGGTCCTTGGCATTTTTCCGGCGCAACGACCGCAAAAAGGTATCCGAGGCGGGGGGACTGCTCGTAGTG

ColE1 origin

7,820 7,840 7,860 7,880 7,900

AAAAATCGACGCTCAAGTCAGAGGTGGCGAAACCCGACAGGACTATAAAGATACCAGGCGTTTCCCCCTGGAAGCTCCCTCGTGCGCTCTCCTGTTCCGACCCTGCC
TTTTTAGCTGCGAGTTCAGTCTCCACCGCTTTGGGCTGTCCTGATATTTCTATGGTCCGCAAAGGGGGACCTTCGAGGGAGCACGCGAGAGGACAAGGCTGGGACGG

ColE1 origin

7,920 7,940 7,960 7,980 8,000 8,020

GCTTACCGGATACCTGTCCGCCTTTCTCCCTTCGGGAAGCGTGGCGCTTTCTCAATGCTCACGCTGTAGGTATCTCAGTTCGGTGTAGGTCGTTCGCTCCAAGCTGG
CGAATGGCCTATGGACAGGCGGAAAGAGGGAAGCCCTTCGCACCGCGAAAGAGTTACGAGTGCGACATCCATAGAGTCAAGCCACATCCAGCAAGCGAGGTTCGACC

ColE1 origin

8,040 8,060 8,080 8,100 8,120

```
GCTGTGTGCACGAACCCCCCGTTCAGCCCGACCGCTGCGCCTTATCCGGTAACTATCGTCTTGAGTCCAACCCGGTAAGACACGACTTATCGCCACTGGCAGCAGCC
CGACACACGTGCTTGGGGGGCAAGTCGGGCTGGCGACGCGGAATAGGCCATTGATAGCAGAACTCAGGTTGGGCCATTCTGTGCTGAATAGCGGTGACCGTCGTCGG
ColE1 origin
    8,140     8,160     8,180     8,200     8,220

ACTGGTAACAGGATTAGCAGAGCGAGGTATGTAGGCGGTGCTACAGAGTTCTTGAAGTGGTGGCCTAACTACGGCTACACTAGAAGGACAGTATTTGGTATCTGCGC
TGACCATTGTCCTAATCGTCTCGCTCCATACATCCGCCACGATGTCTCAAGAACTTCACCACCGGATTGATGCCGATGTGATCTTCCTGTCATAAACCATAGACGCG
ColE1 origin
8,240     8,260     8,280     8,300     8,320     8,340

TCTGCTGAAGCCAGTTACCTTCGGAAAAAGAGTTGGTAGCTCTTGATCCGGCAAACAAACCACCGCTGGTAGCGGTGGTTTTTTTGTTTGCAAGCAGCAGATTACGC
AGACGACTTCGGTCAATGGAAGCCTTTTTCTCAACCATCGAGAACTAGGCCGTTTGTTTGGTGGCGACCATCGCCACCAAAAAAACAAACGTTCGTCGTCTAATGCG
ColE1 origin
    8,360     8,380     8,400     8,420     8,440

GCAGAAAAAAAGGATCTCAAGAAGATCCTTTGATCTTTTCTACGGGGTCTGACGCTCAGTGGAACGAAAACTCACGTTAAGGGATTTTGGTCATGAGATTATCAAAA
CGTCTTTTTTTCCTAGAGTTCTTCTAGGAAACTAGAAAAGATGCCCCAGACTGCGAGTCACCTTGCTTTTGAGTGCAATTCCCTAAAACCAGTACTCTAATAGTTTT
ColE1 origin
   8,460     8,480     8,500     8,520     8,540     8,560

AGGATCTTCACCTAGATCCTTTTAAATTAAAAATGAAGTTTTAAATCAATCTAAAGTATATATGAGTAAACTTGGTCTGACAGTTACCAATGCTTAATCAGTGAGGC
TCCTAGAAGTGGATCTAGGAAAATTTAATTTTTACTTCAAAATTTAGTTAGATTTCATATATACTCATTTGAACCAGACTGTCAATGGTTACGAATTAGTCACTCCG
AmpR
        8,580     8,600     8,620     8,640     8,660

ACCTATCTCAGCGATCTGTCTATTTCGTTCATCCATAGTTGCCTGACTCCCCGTCGTGTAGATAACTACGATACGGGAGGGCTTACCATCTGGCCCCAGTGCTGCAA
TGGATAGAGTCGCTAGACAGATAAAGCAAGTAGGTATCAACGGACTGAGGGGCAGCACATCTATTGATGCTATGCCCTCCCGAATGGTAGACCGGGGTCACGACGTT
AmpR
      8,680     8,700     8,720     8,740     8,760

TGATACCGCGAGACCCACGCTCACCGGCTCCAGATTTATCAGCAATAAACCAGCCAGCCGGAAGGGCCGAGCGCAGAAGTGGTCCTGCAACTTTATCCGCCTCCATC
ACTATGGCGCTCTGGGTGCGAGTGGCCGAGGTCTAAATAGTCGTTATTTGGTCGGTCGGCCTTCCCGGCTCGCGTCTTCACCAGGACGTTGAAATAGGCGGAGGTAG
AmpR
   8,780     8,800     8,820     8,840     8,860     8,880

CAGTCTATTAATTGTTGCCGGGAAGCTAGAGTAAGTAGTTCGCCAGTTAATAGTTTGCGCAACGTTGTTGCCATTGCTGCAGGCATCGTGGTGTCACGCTCGTCGTT
GTCAGATAATTAACAACGGCCCTTCGATCTCATTCATCAAGCGGTCAATTATCAAACGCGTTGCAACAACGGTAACGACGTCCGTAGCACCACAGTGCGAGCAGCAA
AmpR
        8,900     8,920     8,940     8,960     8,980
```

TGGTATGGCTTCATTCAGCTCCGGTTCCCAACGATCAAGGCGAGTTACATGATCCCCCATGTTGTGCAAAAAAGCGGTTAGCTCCTTCGGTCCTCCGATCGTTGTCA
ACCATACCGAAGTAAGTCGAGGCCAAGGGTTGCTAGTTCCGCTCAATGTACTAGGGGGTACAACACGTTTTTTCGCCAATCGAGGAAGCCAGGAGGCTAGCAACAGT

AmpR

9,000 9,020 9,040 9,060 9,080

GAAGTAAGTTGGCCGCAGTGTTATCACTCATGGTTATGGCAGCACTGCATAATTCTCTTACTGTCATGCCATCCGTAAGATGCTTTTCTGTGACTGGTGAGTACTCA
CTTCATTCAACCGGCGTCACAATAGTGAGTACCAATACCGTCGTGACGTATTAAGAGAATGACAGTACGGTAGGCATTCTACGAAAAGACACTGACCACTCATGAGT

AmpR

9,100 9,120 9,140 9,160 9,180 9,200

ACCAAGTCATTCTGAGAATAGTGTATGCGGCGACCGAGTTGCTCTTGCCCGGCGTCAACACGGGATAATACCGCGCCACATAGCAGAACTTTAAAAGTGCTCATCAT
TGGTTCAGTAAGACTCTTATCACATACGCCGCTGGCTCAACGAGAACGGGCCGCAGTTGTGCCCTATTATGGCGCGGTGTATCGTCTTGAAATTTTCACGAGTAGTA

AmpR

9,220 9,240 9,260 9,280 9,300

TGGAAAACGTTCTTCGGGGCGAAAACTCTCAAGGATCTTACCGCTGTTGAGATCCAGTTCGATGTAACCCACTCGTGCACCCAACTGATCTTCAGCATCTTTTACTT
ACCTTTTGCAAGAAGCCCCGCTTTTGAGAGTTCCTAGAATGGCGACAACTCTAGGTCAAGCTACATTGGGTGAGCACGTGGGTTGACTAGAAGTCGTAGAAAATGAA

9,320 9,340 9,360 9,380 9,400

TCACCAGCGTTTCTGGGTGAGCAAAAACAGGAAGGCAAAATGCCGCAAAAAAGGGAATAAGGGCGACACGGAAATGTTGAATACTCATACTCTTCCTTTTTCAATAT
AGTGGTCGCAAAGACCCACTCGTTTTTGTCCTTCCGTTTTACGGCGTTTTTTCCCTTATTCCCGCTGTGCCTTTACAACTTATGAGTATGAGAAGGAAAAAGTTATA

9,420 9,440 9,460 9,480 9,500 9,520

TATTGAAGCATTTATCAGGGTTATTGTCTCATGAGCGGATACATATTTGAATGTATTTAGAAAAATAAACAAATAGGGGTTCCGCGCACATTTCCCCGAAAAGTGCC
ATAACTTCGTAAATAGTCCCAATAACAGAGTACTCGCCTATGTATAAACTTACATAAATCTTTTTATTTGTTTATCCCCAAGGCGCGTGTAAAGGGGCTTTTCACGG

9,540 9,560 9,580 9,600 9,620

ACCTGACGTCTAAGAAACCATTATTATCATGACATTAACCTATAAAAATAGGCGTATCACGAGGCCCTTTCGTCTTCAAGAATTAATTCTCATGTTTGACAGCTTAT
TGGACTGCAGATTCTTTGGTAATAATAGTACTGTAATTGGATATTTTTATCCGCATAGTGCTCCGGGAAAGCAGAAGTTCTTAATTAAGAGTACAAACTGTCGAATA

9,640 9,660 9,680 9,700 9,720

CATCGATAAGCTGACTCATGTTGGTATTGTGAAATAGACGCAGATCGGGAACACTGAAAAATAACAGTTATTATTCG
GTAGCTATTCGACTGAGTACAACCATAACACTTTATCTGCGTCTAGCCCTTGTGACTTTTTATTGTCAATAATAAGC

9,740 9,750 9,760 9,770 9,780 9,790 9,800 9,810

ST/SC ligation / pNb_lys Nb-FLAG (pRS413) (6647...

```
tcgcgcgtttcggtgatgacggtgaaaacctctgacacatgcagctcccggagacggtcacagcttgtctgtaagcggatgccgggagcagacaagcccgtcagggc
agcgcgcaaagccactactgccacttttggagactgtgtacgtcgagggcctctgccagtgtcgaacagacattcgcctacggccctcgtctgttcgggcagtcccg
                  20                  40                  60                  80                 100

gcgtcagcgcgtgttggcgggtgtcggggctggcttaactatgcggcatcagagcagattgtactgagagtgcaccatatttcccgttttaagagcttggtgagcg
cgcagtcgcgcacaaccgcccacagccccgaccgaattgatacgccgtagtctcgtctaacatgactctcacgtggtatttaagggcaaaattctcgaaccactcgc
                                                                                          HIS3
      120                 140                 160                 180                 200

ctaggagtcactgccaggtatcgtttgaacacggcattagtcagggaagtcataacacagtcctttcccgcaattttctttttctattactcttggcctcctctagt
gatcctcagtgacggtccatagcaaacttgtgccgtaatcagtcccttcagtattgtgtcaggaaagggcgttaaaagaaaaagataatgagaaccggaggagatca
                                                         HIS3
     220                 240                 260                 280                 300                 320

acactctatatttttttatgcctcggtaatgattttcattttttttttttcccctagcggatgactcttttttttttcttagcgattggcattatcacataatgaatta
tgtgagatataaaaaaatacggagccattactaaaagtaaaaaaaaaaaaggggatcgcctactgagaaaaaaaaaagaatcgctaaccgtaatagtgtattacttaat
                                                         HIS3
                  340                 360                 380                 400                 420

tacattatataaagtaatgtgatttcttcgaagaatatactaaaaaatgagcaggcaagataaacgaaggcaaagatgacagagcagaaagccctagtaaagcgtat
atgtaatatatttcattacactaaagaagcttcttatatgattttttactcgtccgttctatttgcttccgtttctactgtctcgtctttcgggatcatttcgcata
                                                         HIS3
          440                 460                 480                 500                 520

tacaaatgaaaccaagattcagattgcgatctctttaaagggtggtcccctagcgatagagcactcgatcttcccagaaaaagaggcagaagcagtagcagaacagg
atgtttactttggttctaagtctaacgctagagaaatttcccaccaggggatcgctatctcgtgagctagaagggtctttttctccgtcttcgtcatcgtcttgtcc
                                                         HIS3
    540                 560                 580                 600                 620                 640

ccacacaatcgcaagtgattaacgtccacacaggtatagggtttctggaccatatgatacatgctctggccaagcattccggctggtcgctaatcgttgagtgcatt
ggtgtgttagcgttcactaattgcaggtgtgtccatatcccaaagacctggtatactatgtacgagaccggttcgtaaggccgaccagcgattagcaactcacgtaa
                                                         HIS3
                 660                 680                 700                 720                 740

ggtgacttacacatagacgaccatcacaccactgaagactgcgggattgctctcggtcaagcttttaaagaggccctactggcgcgtggagtaaaaaggtttggatc
ccactgaatgtgtatctgctggtagtgtggtgacttctgacgccctaacgagagccagttcgaaaatttctccgggatgaccgcgcacctcattttttccaaacctag
                                                         HIS3
          760                 780                 800                 820                 840
```

aggatttgcgcctttggatgaggcactttccagagcggtggtagatctttcgaacaggccgtacgcagttgtcgaacttggtttgcaaagggagaaagtaggagatc
tcctaaacgcggaaacctactccgtgaaaggtctcgccaccatctagaaagcttgtccggcatgcgtcaacagcttgaaccaaacgtttccctctttcatcctctag
HIS3
860 880 900 920 940 960

tctcttgcgagatgatcccgcattttcttgaaagctttgcagaggctagcagaattaccctccacgttgattgtctgcgaggcaagaatgatcatcaccgtagtgag
agagaacgctctactagggcgtaaaagaactttcgaaacgtctccgatcgtcttaatgggaggtgcaactaacagacgctccgttcttactagtagtggcatcactc
HIS3
980 1,000 1,020 1,040 1,060

agtgcgttcaaggctcttgcggttgccataagagaagccacctcgcccaatggtaccaacgatgttccctccaccaaaggtgttcttatgtagtgacaccgattatt
tcacgcaagttccgagaacgccaacggtattctcttcggtggagcgggttaccatggttgctacaagggaggtggtttccacaagaatacatcactgtggctaataa
HIS3
1,080 1,100 1,120 1,140 1,160

taaagctgcagcatacgatatatatacatgtgtatatatgtatacctatgaatgtcagtaagtatgtatacgaacagtatgatactgaagatgacaaggtaatgcat
atttcgacgtcgtatgctatatatatgtacacatatatacatatggatacttacagtcattcatacatatgcttgtcatactatgacttctactgttccattacgta
HIS3
1,180 1,200 1,220 1,240 1,260 1,280

cattctatacgtgtcattctgaacgaggcgcgctttcctttttcttttgctttttctttttttttctcttgaactcgacggatctatgcggtgtgaaataccgca
gtaagatatgcacagtaagacttgctccgcgcgaaaggaaaaagaaaaacgaaaaagaaaaaaaaagagaacttgagctgcctagatacgccacactttatggcgt
HIS3
1,300 1,320 1,340 1,360 1,380

cagatgcgtaaggagaaaataccgcatcaggaaattgtaaacgttaatattttgttaaaattcgcgttaaatttttgttaaatcagctcattttttaaccaataggc
gtctacgcattcctcttttatggcgtagtcctttaacatttgcaattataaaacaattttaagcgcaatttaaaaacaatttagtcgagtaaaaaattggttatccg
1,400 1,420 1,440 1,460 1,480

cgaaatcggcaaaatcccttataaatcaaaagaatagaccgagatagggttgagtgtgttccagttggaacaagagtccactattaaagaacgtggactccaacgtc
gctttagccgttttagggaatatttagttttcttatctggctctatcccaactcacacaaggtcaaccttgttctcaggtgataatttcttgcacctgaggttgcag
F1 ori
1,500 1,520 1,540 1,560 1,580 1,600

aaagggcgaaaaaccgtctatcagggcgatggcccactacgtgaaccatcaccctaatcaagttttttggggtcgaggtgccgtaaagcactaaatcggaaccctaaa
tttcccgcttttggcagatagtcccgctaccgggtgatgcacttggtagtgggattagttcaaaaaaccccagctccacggcatttcgtgatttagccttgggattt
F1 ori
1,620 1,640 1,660 1,680 1,700

gggagcccccgatttagagcttgacggggaaagccggcgaacgtggcgagaaaggaagggaagaaagcgaaaggagcgggcgctagggcgctggcaagtgtagcggt
ccctcgggggctaaatctcgaactgcccctttcggccgcttgcaccgctctttccttccttctttcgctttcctcgcccgcgatcccgcgaccgttcacatcgcca
F1 ori
1,720 1,740 1,760 1,780 1,800

```
cacgctgcgcgtaaccaccacacccgccgcgcttaatgcgccgctacagggcgcgtccattcgccattcaggctgcgcaactgttgggaagggcgatcggtgcgggc
gtgcgacgcgcattggtggtgtgggcggcgcgaattacgcggcgatgtcccgcgcaggtaagcggtaagtccgacgcgttgacaacccttcccgctagccacgcccg
F1 ori
1,820    1,840    1,860    1,880    1,900    1,920

ctcttcgctattacgccagctggcgaaagggggatgtgctgcaaggcgattaagttgggtaacgccagggttttcccagtcacgacgttgtaaaacgacggccagtg
gagaagcgataatgcggtcgaccgctttccccctacacgacgttccgctaattcaacccattgcggtcccaaaagggtcagtgctgcaacattttgctgccggtcac
1,940    1,960    1,980    2,000    2,020

agcgcgcAGTTTATCATTATCAATACTcGCCATTTCAAAGAATACGTAAATAATTAATAGTAGTGATTTTCCTAACTTTATTTAGTCAAAAAATTAGCCTTTTAATT
tcgcgcgTCAAATAGTAATAGTTATGAgCGGTAAAGTTTCTTATGCATTTATTAATTATCATCACTAAAAGGATTGAAATAAATCAGTTTTTTAATCGGAAAATTAA
GPD promoter
2,040    2,060    2,080    2,100    2,120    2,140

CTGCTGTAACCCGTACATGCCCAAAATAGGGGGCGGGTTACACAGAATATATAACATCGTAGGTGTCTGGGTGAACAGTTTATTCCTGGCATCCACTAAATATAATG
GACGACATTGGGCATGTACGGGTTTTATCCCCCGCCCAATGTGTCTTATATATTGTAGCATCCACAGACCCACTTGTCAAATAAGGACCGTAGGTGATTTATATTAC
GPD promoter
2,160    2,180    2,200    2,220    2,240

GAGCCCGCTTTTTAAGCTGGCATCCAGAAAAAAAAAGAATCCCAGCACCAAAATATTGTTTTCTTCACCAACCATCAGTTCATAGGTCCATTCTCTTAGCGCAACTA
CTCGGGCGAAAAATTCGACCGTAGGTCTTTTTTTTTCTTAGGGTCGTGGTTTTATAACAAAAGAAGTGGTTGGTAGTCAAGTATCCAGGTAAGAGAATCGCGTTGAT
GPD promoter
2,260    2,280    2,300    2,320    2,340

CAGAGAACAGGGGCACAAACAGGCAAAAAACGGGCACAACCTCAATGGAGTGATGCAACCTGCCTGGAGTAAATGATGACACAAGGCAATTGACCCACGCATGTATC
GTCTCTTGTCCCCGTGTTTGTCCGTTTTTTGCCCGTGTTGGAGTTACCTCACTACGTTGGACGGACCTCATTTACTACTGTGTTCCGTTAACTGGGTGCGTACATAG
GPD promoter
2,360    2,380    2,400    2,420    2,440    2,460

TATCTCATTTTCTTACACCTTCTATTACCTTCTGCTCTCTCTGATTTGGAAAAAGCTGAAAAAAAAAGGTTGAAACCAGTTCCCTGAAATTATTCCCCTACTTGACTA
ATAGAGTAAAAGAATGTGGAAGATAATGGAAGACGAGAGAGACTAAACCTTTTTCGACTTTTTTTTTCCAACTTTGGTCAAGGGACTTTAATAAGGGGATGAACTGAT
GPD promoter
2,480    2,500    2,520    2,540    2,560

ATAAGTATATAAAGACGGTAGGTATTGATTGTAATTCTGTAAATCTATTTCTTAAACTTCTTAAATTCTACTTTTATAGTTAGTCTTTTTTTTAGTTTTAAAACACC
TATTCATATATTTCTGCCATCCATAACTAACATTAAGACATTTAGATAAAGAATTTGAAGAATTTAAGATGAAAATATCAATCAGAAAAAAAATCAAAATTTTGTGG
GPD promoter
2,580    2,600    2,620    2,640    2,660
```

```
AGAACTTAGTTTCGACGGATTCTAGAACTAGTGGATCCATGAGATTCCCATCTATCTTCACCGCTGTTTTGTTCGCTGCTTCTTCTGCTTTGGCTGCTCCAGCTAAC
TCTTGAATCAAAGCTGCCTAAGATCTTGATCACCTAGGTACTCTAAGGGTAGATAGAAGTGGCGACAAAACAAGCGACGAAGAAGACGAAACCGACGAGGTCGATTG
1 2 3 4 5 6 7 8 9 10 11 12 13 14 15 16 17 18 19 20 21 22 23
M R F P S I F T A V L F A A S S A L A A P A N
GPD promoter | Mating factor alpha leader
2,680  2,700  2,720  2,740  2,760  2,780
```

```
ACCACCACCGAAGACGAAACCGCTCAAATCCCAGCTGAAGCTGTTATCGACTACTCTGACTTGGAAGGTGACTTCGACGCTGCTGCTTTGCCATTGTCTAACTCTAC
TGGTGGTGGCTTCTGCTTTGGCGAGTTTAGGGTCGACTTCGACAATAGCTGATGAGACTGAACCTTCCACTGAAGCTGCGACGACGAAACGGTAACAGATTGAGATG
24 25 26 27 28 29 30 31 32 33 34 35 36 37 38 39 40 41 42 43 44 45 46 47 48 49 50 51 52 53 54 55 56 57 58 59
T T T E D E T A Q I P A E A V I D Y S D L E G D F D A A A L P L S N S T
Mating factor alpha leader
2,800  2,820  2,840  2,860  2,880
```

```
CAACAACGGTTTGTCTTCTACCAACACCACCATCGCTTCTATCGCTGCTAAGGAAGAAGGTGTTCAATTGGACAAGAGAGAAagatctggcggccgcgcatgcgcta
GTTGTTGCCAAACAGAAGATGGTTGTGGTGGTAGCGAAGATAGCGACGATTCCTTCTTCCACAAGTTAACCTGTTCTCTCTTtctagaccgccggcgcgtacgcgat
60 61 62 63 64 65 66 67 68 69 70 71 72 73 74 75 76 77 78 79 80 81 82 83 84 85 86
N N G L S S T N T T I A S I A A K E E G V Q L D K R E
Mating factor alpha leader
2,900  2,920  2,940  2,960  2,980
```

```
gcctcgagGATGTGCAGTTGCAAGCATCCGGCGGCGGTTCTGTTCAGGCTGGTGGTTCTCTAAGATTAAGTTGTGCTGCTTCAGGTTATACCATCGGCCCATACTGC
cggagctcCTACACGTCAACGTTCGTAGGCCGCCGCCAAGACAAGTCCGACCACCAAGAGATTCTAATTCAACACGACGAAGTCCAATATGGTAGCCGGGTATGACG
2 4 6 8 10 12 14 16 18 20 22 24 26 28 30 32
D V Q L Q A S G G G S V Q A G G S L R L S C A A S G Y T I G P Y C
anti-lysozyme nanobody
3,000  3,020  3,040  3,060  3,080  3,100
```

```
ATGGGTTGGTTTCGTCAAGCTCCAGGCAAAGAAAGGGAGGGAGTCGCAGCCATTAACATGGGCGGCGGTATCACATATTATGCAGACTCCGTAAAAGGTAGATTTAC
TACCCAACCAAAGCAGTTCGAGGTCCGTTTCTTTCCCTCCCTCAGCGTCGGTAATTGTACCCGCCGCCATAGTGTATAATACGTCTGAGGCATTTTCCATCTAAATG
34 36 38 40 42 44 46 48 50 52 54 56 58 60 62 64 66 68
M G W F R Q A P G K E R E G V A A I N M G G G I T Y Y A D S V K G R F T
anti-lysozyme nanobody
3,120  3,140  3,160  3,180  3,200
```

```
TATCTCTCAGGACAATGCTAAGAACACGGTCTATCTGTTAATGAATTCATTGGAGCCCGAGGACACTGCCATATACTACTGTGCCGCAGATTCAACCATTTACGCTT
ATAGAGAGTCCTGTTACGATTCTTGTGCCAGATAGACAATTACTTAAGTAACCTCGGGCTCCTGTGACGGTATATGATGACACGGCGTCTAAGTTGGTAAATGCGAA
70 72 74 76 78 80 82 84 86 88 90 92 94 96 98 100 102 104
I S Q D N A K N T V Y L L M N S L E P E D T A I Y Y C A A D S T I Y A
anti-lysozyme nanobody
3,220  3,240  3,260  3,280  3,300
```

CCTATTACGAATGCGGACACGGATTAAGTACTGGAGGTTACGGTTATGATTCCTGGGGTCAAGGCACACAAGTCACGGTATCATCAGGCGGATCAGACTATAAGGAT
GGATAATGCTTACGCCTGTGCCTAATTCATGACCTCCAATGCCAATACTAAGGACCCCAGTTCCGTGTGTTCAGTGCCATAGTAGTCCGCCTAGTCTGATATTCCTA
106 108 110 112 114 116 118 120 122 124 126 128 130 132 1 2 3 1 2 3 4
S Y Y E C G H G L S T G G Y G Y D S W G Q G T Q V T V S S G G S D Y K D
anti-lysozyme nanobody
FLA...ag
3,320 3,340 3,360 3,380 3,400 3,420

GACGACGATAAGGCTGGAGGTTCGAGGGGAGTCCCTCACATAGTGATGGTGGATGCTTACAAAAGGTATAAATAACCCGGGCTGCAGGAATTCGATATCAAGCTTAT
CTGCTGCTATTCCGACCTCCAAGCTCCCCTCAGGGAGTGTATCACTACCACCTACGAATGTTTTCCATATTTATTGGGCCCGACGTCCTTAAGCTATAGTTCGAATA
5 6 7 8 1 2 3 4 1 2 3 4 5 6 7 8 9 10 11 12 13 14 15 16 1
D D D K A G G S R G V P H I V M V D A Y K R Y K *
FLA...ag
SpyTag
3,440 3,460 3,480 3,500 3,520

CGATACCGTCGACCTCGAGTCATGTAATTAGTTATGTCACGCTTACGTTCACGCCCTCCCCCCACATCCGCTCTAACCGAAAAGGAAGGAGTTAGACAACCTGAAGT
GCTATGGCAGCTGGAGCTCAGTACATTAATCAATACAGTGCGAATGCAAGTGCGGGAGGGGGGTGTAGGCGAGATTGGCTTTTCCTTCCTCAATCTGTTGGACTTCA
CYC1 terminator
3,540 3,560 3,580 3,600 3,620

CTAGGTCCCTATTTATTTTTTTATAGTTATGTTAGTATTAAGAACGTTATTTATATTTCAAATTTTTCTTTTTTTTCTGTACAGACGCGTGTACGCATGTAACATTA
GATCCAGGGATAAATAAAAAAATATCAATACAATCATAATTCTTGCAATAAATATAAAGTTTAAAAAGAAAAAAAAGACATGTCTGCGCACATGCGTACATTGTAAT
CYC1 terminator
3,640 3,660 3,680 3,700 3,720 3,740

TACTGAAAACCTTGCTTGAGAAGGTTTTGGGACGCTCGAAGGCTTTAATTTGCtcgaattcctgcagcccgggggatccactagttctagagcggccgccaccgcgg
ATGACTTTTGGAACGAACTCTTCCAAAACCCTGCGAGCTTCCGAAATTAAACGagcttaaggacgtcgggccccctaggtgatcaagatctcgccggcggtggcgcc
CYC1 terminator
3,760 3,780 3,800 3,820 3,840

tggagctccagcttttgttccctttagtgagggttaattgcgcgcttggcgtaatcatggtcatagctgtttcctgtgtgaaattgttatccgctcacaattccaca
acctcgaggtcgaaaacaagggaaatcactcccaattaacgcgcgaaccgcattagtaccagtatcgacaaaggacacactttaacaataggcgagtgttaaggtgt
LacO
3,860 3,880 3,900 3,920 3,940

caacataggagccggaagcataaagtgtaaagcctggggtgcctaatgagtgaggtaactcacattaattgcgttgcgctcactgcccgctttccagtcgggaaacc
gttgtatcctcggccttcgtatttcacatttcggaccccacggattactcactccattgagtgtaattaacgcaacgcgagtgacgggcgaaaggtcagccctttgg
3,960 3,980 4,000 4,020 4,040 4,060

tgtcgtgccagctgcattaatgaatcggccaacgcgcggggagaggcggtttgcgtattgggcgctcttccgcttcctcgctcactgactcgctgcgctcggtcgtt
acagcacggtcgacgtaattacttagccggttgcgcgcccctctccgccaaacgcataacccgcgagaaggcgaaggagcgagtgactgagcgacgcgagccagcaa
4,080 4,100 4,120 4,140 4,160

cggctgcggcgagcggtatcagctcactcaaaggcggtaatacggttatccacagaatcaggggataacgcaggaaagaacatgtgagcaaaaggccagcaaaaggc
gccgacgccgctcgccatagtcgagtgagtttccgccattatgccaataggtgtcttagtcccctattgcgtcctttcttgtacactcgttttccggtcgttttccg

4,180 4,200 4,220 4,240 4,260 4,280

caggaaccgtaaaaaggccgcgttgctggcgtttttccataggctccgcccccctgacgagcatcacaaaaatcgacgctcaagtcagaggtggcgaaacccgacag
gtccttggcatttttccggcgcaacgaccgcaaaaaggtatccgaggcggggggactgctcgtagtgtttttagctgcgagttcagtctccaccgctttgggctgtc

ColE1 origin

4,300 4,320 4,340 4,360 4,380

gactataaagataccaggcgtttccccctggaagctccctcgtgcgctctcctgttccgaccctgccgcttaccggatacctgtccgcctttctcccttcgggaagc
ctgatatttctatggtccgcaaagggggaccttcgagggagcacgcgagaggacaaggctgggacggcgaatggcctatggacaggcggaaagagggaagcccttcg

ColE1 origin

4,400 4,420 4,440 4,460 4,480

gtggcgctttctcatagctcacgctgtaggtatctcagttcggtgtaggtcgttcgctccaagctgggctgtgtgcacgaaccccccgttcagcccgaccgctgcgc
caccgcgaaagagtatcgagtgcgacatccatagagtcaagccacatccagcaagcgaggttcgacccgacacacgtgcttggggggcaagtcgggctggcgacgcg

ColE1 origin

4,500 4,520 4,540 4,560 4,580 4,600

cttatccggtaactatcgtcttgagtccaacccggtaagacacgacttatcgccactggcagcagccactggtaacaggattagcagagcgaggtatgtaggcggtg
gaataggccattgatagcagaactcaggttgggccattctgtgctgaatagcggtgaccgtcgtcggtgaccattgtcctaatcgtctcgctccatacatccgccac

ColE1 origin

4,620 4,640 4,660 4,680 4,700

ctacagagttcttgaagtggtggcctaactacggctacactagaaggacagtatttggtatctgcgctctgctgaagccagttaccttcggaaaaagagttggtagc
gatgtctcaagaacttcaccaccggattgatgccgatgtgatcttcctgtcataaaccatagacgcgagacgacttcggtcaatggaagcctttttctcaaccatcg

ColE1 origin

4,720 4,740 4,760 4,780 4,800

tcttgatccggcaaacaaaccaccgctggtagcggtggtttttttgtttgcaagcagcagattacgcgcagaaaaaaaggatctcaagaagatcctttgatcttttc
agaactaggccgtttgtttggtggcgaccatcgccaccaaaaaaacaaacgttcgtcgtctaatgcgcgtcttttttttcctagagttcttctaggaaactagaaaag

ColE1 origin

4,820 4,840 4,860 4,880 4,900 4,920

tacggggtctgacgctcagtggaacgaaaactcacgttaagggattttggtcatgagattatcaaaaaggatcttcacctagatccttttaaattaaaaatgaagtt
atgccccagactgcgagtcaccttgcttttgagtgcaattccctaaaaccagtactctaatagtttttcctagaagtggatctaggaaaatttaatttttacttcaa

ColE1 origin

4,940 4,960 4,980 5,000 5,020

ttaaatcaatctaaagtatatatgagtaaacttggtctgacagttaccaatgcttaatcagtgaggcacctatctcagcgatctgtctatttcgttcatccatagtt
aatttagttagatttcatatatactcatttgaaccagactgtcaatggttacgaattagtcactccgtggatagagtcgctagacagataaagcaagtaggtatcaa

AmpR

5,040 5,060 5,080 5,100 5,120

gcctgactccccgtcgtgtagataactacgatacgggagggcttaccatctggccccagtgctgcaatgataccgcgagacccacgctcaccggctccagatttatc
cggactgaggggcagcacatctattgatgctatgccctcccgaatggtagaccggggtcacgacgttactatggcgctctgggtgcgagtggccgaggtctaaatag
AmpR
5,140 5,160 5,180 5,200 5,220 5,240

agcaataaaccagccagccggaagggccgagcgcagaagtggtcctgcaactttatccgcctccatccagtctattaattgttgccgggaagctagagtaagtagtt
tcgttatttggtcggtcggccttcccggctcgcgtcttcaccaggacgttgaaataggcggaggtaggtcagataattaacaacggcccttcgatctcattcatcaa
AmpR
5,260 5,280 5,300 5,320 5,340

cgccagttaatagtttgcgcaacgttgttgccattgctacaggcatcgtggtgtcacgctcgtcgtttggtatggcttcattcagctccggttcccaacgatcaagg
gcggtcaattatcaaacgcgttgcaacaacggtaacgatgtccgtagcaccacagtgcgagcagcaaaccataccgaagtaagtcgaggccaagggttgctagttcc
AmpR
5,360 5,380 5,400 5,420 5,440

cgagttacatgatcccccatgttgtgcaaaaaagcggttagctccttcggtcctccgatcgttgtcagaagtaagttggccgcagtgttatcactcatggttatggc
gctcaatgtactagggggtacaacacgttttttcgccaatcgaggaagccaggaggctagcaacagtcttcattcaaccggcgtcacaatagtgagtaccaataccg
AmpR
5,460 5,480 5,500 5,520 5,540 5,560

agcactgcataattctcttactgtcatgccatccgtaagatgcttttctgtgactggtgagtactcaaccaagtcattctgagaatagtgtatgcggcgaccgagtt
tcgtgacgtattaagagaatgacagtacggtaggcattctacgaaaagacactgaccactcatgagttggttcagtaagactcttatcacatacgccgctggctcaa
AmpR
5,580 5,600 5,620 5,640 5,660

gctcttgcccggcgtcaatacgggataataccgcgccacatagcagaactttaaaagtgctcatcattggaaaacgttcttcggggcgaaaactctcaaggatctta
cgagaacgggccgcagttatgccctattatggcgcggtgtatcgtcttgaaattttcacgagtagtaaccttttgcaagaagccccgcttttgagagttcctagaat
AmpR
5,680 5,700 5,720 5,740 5,760

ccgctgttgagatccagttcgatgtaacccactcgtgcacccaactgatcttcagcatcttttactttcaccagcgtttctgggtgagcaaaaacaggaaggcaaaa
ggcgacaactctaggtcaagctacattgggtgagcacgtgggttgactagaagtcgtagaaaatgaaagtggtcgcaaagacccactcgtttttgtccttccgtttt
5,780 5,800 5,820 5,840 5,860 5,880

tgccgcaaaaaagggaataagggcgacacggaaatgttgaatactcatactcttcctttttcaatattattgaagcatttatcagggttattgtctcatgagcggat
acggcgttttttcccttattcccgctgtgcctttacaacttatgagtatgagaaggaaaaagttataataacttcgtaaatagtcccaataacagagtactcgccta
5,900 5,920 5,940 5,960 5,980

acatatttgaatgtatttagaaaaataaacaaataggggttccgcgcacatttccccgaaaagtgccacctgggtccttttcatcacgtgctataaaaataattata
tgtataaacttacataaatctttttatttgtttatccccaaggcgcgtgtaaaggggcttttcacggtggacccaggaaaagtagtgcacgatatttttattaatat
CEN-ARS pRS
6,000 6,020 6,040 6,060 6,080

atttaaatttttaatataaatatataaattaaaaatagaaagtaaaaaaagaaattaaagaaaaaatagtttttgttttccgaagatgtaaaagactctaggggga
taaatttaaaaattatatttatatatttaatttttatctttcatttttttctttaatttctttttttatcaaaaacaaaaggcttctacattttctgagatccccct

CEN-ARS pRS

6,100 6,120 6,140 6,160 6,180 6,200

tcgccaacaaatactaccttttatcttgctcttcctgctctcaggtattaatgccgaattgtttcatcttgtctgtgtagaagaccacacacgaaaatcctgtgatt
agcggttgtttatgatggaaaatagaacgagaaggacgagagtccataattacggcttaacaaagtagaacagacacatcttctggtgtgtgcttttaggacactaa

CEN-ARS pRS

6,220 6,240 6,260 6,280 6,300

ttacattttacttatcgttaatcgaatgtatatctatttaatctgcttttcttgtctaataaatatatatgtaaagtacgctttttgttgaaattttttaaacctttt
aatgtaaaatgaatagcaattagcttacatatagataaattagacgaaaagaacagattatttatatatacatttcatgcgaaaaacaactttaaaaaatttggaaa

CEN-ARS pRS

6,320 6,340 6,360 6,380 6,400 6,420

gtttatttttttttcttcattccgtaactcttctaccttctttatttactttctaaaatccaaatacaaaacataaaaataaataaacacagagtaaattcccaaat
caaataaaaaaaaagaagtaaggcattgagaagatggaagaaataaatgaaagattttaggtttatgttttgtatttttatttatttgtgtctcatttaagggttta

CEN-ARS pRS

6,440 6,460 6,480 6,500 6,520

tattccatcattaaaagatacgaggcgcgtgtaagttacaggcaagcgatccgtcctaagaaaccattattatcatgacattaacctataaaaataggcgtatcacg
ataaggtagtaattttctatgctccgcgcacattcaatgtccgttcgctaggcaggattctttggtaataatagtactgtaattggatatttttatccgcatagtgc

CEN-ARS pRS

6,540 6,560 6,580 6,600 6,620

aggccctttcgtc
tccgggaaagcag

6,640

ST/SC ligation / pNb_lys Nb-FLAG (pRS415) (7136...

GGTGGCTCATGTTGTAGGGCCATGAAAGCGGCCATTCTTGTGATTCTTTGCACTTCTGGAACGGTGTATTGTTCACTATCCCAAGCGACACCATCACCATCGTCTTC
CCACCGAGTACAACATCCCGGTACTTTCGCCGGTAAGAACACTAAGAAACGTGAAGACCTTGCCACATAACAAGTGATAGGGTTCGCTGTGGTAGTGGTAGCAGAAG
LEU2
20 40 60 80 100

CTTTCTCTTACCAAAGTAAATACCTCCCACTAATTCTCTGACAACAACGAAGTCAGTACCTTTAGCAAATTGTGGCTTGATTGGAGATAAGTCTAAAAGAGAGTCGG
GAAAGAGAATGGTTTCATTTATGGAGGGTGATTAAGAGACTGTTGTTGCTTCAGTCATGGAAATCGTTTAACACCGAACTAACCTCTATTCAGATTTTCTCTCAGCC
LEU2
120 140 160 180 200

ATGCAAAGTTACATGGTCTTAAGTTGGCGTACAATTGAAGTTCTTTACGGATTTTTAGTAAACCTTGTTCAGGTCTAACACTACCGGTACCCCATTTAGGACCACCC
TACGTTTCAATGTACCAGAATTCAACCGCATGTTAACTTCAAGAAATGCCTAAAAATCATTTGGAACAAGTCCAGATTGTGATGGCCATGGGGTAAATCCTGGTGGG
LEU2
220 240 260 280 300 320

ACAGCACCTAACAAAACGGCATCAGCCTTCTTGGAGGCTTCCAGCGCCTCATCTGGAAGTGGAACACCTGTAGCATCGATAGCAGCACCACCAATTAAATGATTTTC
TGTCGTGGATTGTTTTGCCGTAGTCGGAAGAACCTCCGAAGGTCGCGGAGTAGACCTTCACCTTGTGGACATCGTAGCTATCGTCGTGGTGGTTAATTTACTAAAAG
LEU2
340 360 380 400 420

GAAATCGAACTTGACATTGGAACGAACATCAGAAATAGCTTTAAGAACCTTAATGGCTTCGGCTGTGATTTCTTGACCAACGTGGTCACCTGGCAAAACGACGATCT
CTTTAGCTTGAACTGTAACCTTGCTTGTAGTCTTTATCGAAATTCTTGGAATTACCGAAGCCGACACTAAAGAACTGGTTGCACCAGTGGACCGTTTTGCTGCTAGA
LEU2
440 460 480 500 520

TCTTAGGGGCAGACATTAGAATGGTATATCCTTGAAATATATATATATATATATNTNGCTGAAATGTAAAAGGTAAGAAAAGTTAGAAAGTAAGACGATTGCTAACCAC
AGAATCCCCGTCTGTAATCTTACCATATAGGAACTTTATATATATATATATATANANCGACTTTACATTTTCCATTCTTTTCAATCTTTCATTCTGCTAACGATTGGTG
LEU2
540 560 580 600 620 640

CTATTGGAAAAAACAATAGGTCCTTAAATAATATTGTCAACTTCAAGTATTGTGATGCAAGCATTTAGTCATGAACGCTTCTCTATTCTATATGAAAAGCCGGTTCC
GATAACCTTTTTTGTTATCCAGGAATTTATTATAACAGTTGAAGTTCATAACACTACGTTCGTAAATCAGTACTTGCGAAGAGATAAGATATACTTTTCGGCCAAGG
660 680 700 720 740

GGCGCTCTCACCTTTCCTTTTTCTCCCAATTTTTCAGTTGAAAAAGGTATATGCGTCAGGCGACCTCTGAAATTAACAAAAAATTTCCAGTCATCGAATTTGATTCT
CCGCGAGAGTGGAAAGGAAAAAGAGGGTTAAAAAGTCAACTTTTTCCATATACGCAGTCCGCTGGAGACTTTAATTGTTTTTTAAAGGTCAGTAGCTTAAACTAAGA
760 780 800 820 840

GTGCGATAGCGCCCCTGTGTGTTCTCGTTATGTTGAGGAAAAAAATAATGGTTGCTAAGAGATTCGAACTCTTGCATCTTACGATACCTGAGTATTCCCACAGTTTG
CACGCTATCGCGGGGACACACAAGAGCAATACAACTCCTTTTTTTATTACCAACGATTCTCTAAGCTTGAGAACGTAGAATGCTATGGACTCATAAGGGTGTCAAAC
860 880 900 920 940 960

AAAAGCTGTGGTATGGTGCACTCTCAGTACAATCTGCTCTGATGCCGCATAGTTAAGCCAGCCCCGACACCCGCCAACACCCGCTGACGCGCCCTGACGGGCTTGTC
TTTTCGACACCATACCACGTGAGAGTCATGTTAGACGAGACTACGGCGTATCAATTCGGTCGGGGCTGTGGGCGGTTGTGGGCGACTGCGCGGGACTGCCCGAACAG

980 1,000 1,020 1,040 1,060

TGCTCCCGGCATCCGCTTACAGACAAGCTGTGACCGTCTCCGGGAGCTGCATGTGTCAGAGGTTTTCACCGTCATCACCGAAACGCGCGAGACGAAAGGGCCTCGTG
ACGAGGGCCGTAGGCGAATGTCTGTTCGACACTGGCAGAGGCCCTCGACGTACACAGTCTCCAAAAGTGGCAGTAGTGGCTTTGCGCGCTCTGCTTTCCCGGAGCAC

1,080 1,100 1,120 1,140 1,160

ATACGCCTATTTTTATAGGTTAATGTCATGATAATAATGGTTTCTTAGTAgatcgcttgcctgtaacttacacgcgcctcgtatcttttaatgatggaataatttgg
TATGCGGATAAAAATATCCAATTACAGTACTATTATTACCAAAGAATCATctagcgaacggacattgaatgtgcgcggagcatagaaaattactaccttattaaacc

CEN/ARS

1,180 1,200 1,220 1,240 1,260 1,280

gaatttactctgtgtttatttatttttatgttttgtatttggattttagaaagtaaataaagaaggtagaagagttacggaatgaagaaaaaaaaataaacaaaggt
cttaaatgagacacaaataaataaaaatacaaaacataaacctaaaatctttcatttatttcttccatcttctcaatgccttacttctttttttttatttgtttcca

CEN/ARS

1,300 1,320 1,340 1,360 1,380

ttaaaaaatttcaacaaaaagcgtactttacatatatatttattagacaagaaaagcagattaaatagatatacattcgattaacgataagtaaaatgtaaaatcac
aatttttttaaagttgtttttcgcatgaaatgtatatataaataatctgttcttttcgtctaatttatctatatgtaagctaattgctattcattttacattttagtg

CEN/ARS

1,400 1,420 1,440 1,460 1,480

aggattttcgtgtgtggtcttctacacagacaagatgaaacaattcggcattaatacctgagagcaggaagagcaagataaaaggtagtatttgttggcgatccccc
tcctaaaagcacacaccagaagatgtgtctgttctactttgttaagccgtaattatggactctcgtccttctcgttctattttccatcataaacaaccgctaggggg

CEN/ARS

1,500 1,520 1,540 1,560 1,580 1,600

tagagtcttttacatcttcggaaaacaaaaactattttttctttaatttctttttttactttctatttttaatttatatatttatattaaaaaatttaaattataat
atctcagaaaatgtagaagccttttgtttttgataaaaaagaaattaaagaaaaaaatgaaagataaaaattaaatatataaatataatttttttaaatttaatatta

CEN/ARS

1,620 1,640 1,660 1,680 1,700

tatttttatagcacgtgatGTTCAGGTGGCACTTTTCGGGGAAATGTGCGCGGAACCCCTATTTGTTTATTTTTCTAAATACATTCAAATATGTATCCGCTCATGAG
ataaaaatatcgtgcactaCAAGTCCACCGTGAAAAGCCCCTTTACACGCGCCTTGGGGATAAACAAATAAAAAGATTTATGTAAGTTTATACATAGGCGAGTACTC

CEN/ARS

1,720 1,740 1,760 1,780 1,800

ACAATAACCCTGATAAATGCTTCAATAATATTGAAAAAGGAAGAGTATGAGTATTCAACATTTCCGTGTCGCCCTTATTCCCTTTTTTGCGGCATTTTGCCTTCCTG
TGTTATTGGGACTATTTACGAAGTTATTATAACTTTTTCCTTCTCATACTCATAAGTTGTAAAGGCACAGCGGGAATAAGGGAAAAAACGCCGTAAAACGGAAGGAC

1,820 1,840 1,860 1,880 1,900 1,920

TTTTTGCTCACCCAGAAACGCTGGTGAAAGTAAAAGATGCTGAAGATCAGTTGGGTGCACGAGTGGGTTACATCGAACTGGATCTCAACAGCGGTAAGATCCTTGAG
AAAAACGAGTGGGTCTTTGCGACCACTTTCATTTTCTACGACTTCTAGTCAACCCACGTGCTCACCCAATGTAGCTTGACCTAGAGTTGTCGCCATTCTAGGAACTC

1,940 1,960 1,980 2,000 2,020

AGTTTTCGCCCCGAAGAACGTTTTCCAATGATGAGCACTTTTAAAGTTCTGCTATGTGGCGCGGTATTATCCCGTATTGACGCCGGGCAAGAGCAACTCGGTCGCCG
TCAAAAGCGGGGCTTCTTGCAAAAGGTTACTACTCGTGAAAATTTCAAGACGATACACCGCGCCATAATAGGGCATAACTGCGGCCCGTTCTCGTTGAGCCAGCGGC
AmpR

2,040 2,060 2,080 2,100 2,120 2,140

CATACACTATTCTCAGAATGACTTGGTTGAGTACTCACCAGTCACAGAAAAGCATCTTACGGATGGCATGACAGTAAGAGAATTATGCAGTGCTGCCATAACCATGA
GTATGTGATAAGAGTCTTACTGAACCAACTCATGAGTGGTCAGTGTCTTTTCGTAGAATGCCTACCGTACTGTCATTCTCTTAATACGTCACGACGGTATTGGTACT
AmpR

2,160 2,180 2,200 2,220 2,240

GTGATAACACTGCGGCCAACTTACTTCTGACAACGATCGGAGGACCGAAGGAGCTAACCGCTTTTTTGCACAACATGGGGGATCATGTAACTCGCCTTGATCGTTGG
CACTATTGTGACGCCGGTTGAATGAAGACTGTTGCTAGCCTCCTGGCTTCCTCGATTGGCGAAAAAACGTGTTGTACCCCCTAGTACATTGAGCGGAACTAGCAACC
AmpR

2,260 2,280 2,300 2,320 2,340

GAACCGGAGCTGAATGAAGCCATACCAAACGACGAGCGTGACACCACGATGCCTGTAGCAATGGCAACAACGTTGCGCAAACTATTAACTGGCGAACTACTTACTCT
CTTGGCCTCGACTTACTTCGGTATGGTTTGCTGCTCGCACTGTGGTGCTACGGACATCGTTACCGTTGTTGCAACGCGTTTGATAATTGACCGCTTGATGAATGAGA
AmpR

2,360 2,380 2,400 2,420 2,440 2,460

AGCTTCCCGGCAACAATTAATAGACTGGATGGAGGCGGATAAAGTTGCAGGACCACTTCTGCGCTCGGCCCTTCCGGCTGGCTGGTTTATTGCTGATAAATCTGGAG
TCGAAGGGCCGTTGTTAATTATCTGACCTACCTCCGCCTATTTCAACGTCCTGGTGAAGACGCGAGCCGGGAAGGCCGACCGACCAAATAACGACTATTTAGACCTC
AmpR

2,480 2,500 2,520 2,540 2,560

CCGGTGAGCGTGGGTCTCGCGGTATCATTGCAGCACTGGGGCCAGATGGTAAGCCCTCCCGTATCGTAGTTATCTACACGACGGGGAGTCAGGCAACTATGGATGAA
GGCCACTCGCACCCAGAGCGCCATAGTAACGTCGTGACCCCGGTCTACCATTCGGGAGGGCATAGCATCAATAGATGTGCTGCCCCTCAGTCCGTTGATACCTACTT
AmpR

2,580 2,600 2,620 2,640 2,660

CGAAATAGACAGATCGCTGAGATAGGTGCCTCACTGATTAAGCATTGGTAACTGTCAGACCAAGTTTACTCATATATACTTTAGATTGATTTAAAACTTCATTTTTA
GCTTTATCTGTCTAGCGACTCTATCCACGGAGTGACTAATTCGTAACCATTGACAGTCTGGTTCAAATGAGTATATATGAAATCTAACTAAATTTTGAAGTAAAAAT
AmpR

2,680 2,700 2,720 2,740 2,760 2,780

ATTTAAAAGGATCTAGGTGAAGATCCTTTTTGATAATCTCATGACCAAAATCCCTTAACGTGAGTTTTCGTTCCACTGAGCGTCAGACCCCGTAGAAAAGATCAAAG
TAAATTTTCCTAGATCCACTTCTAGGAAAAACTATTAGAGTACTGGTTTTAGGGAATTGCACTCAAAAGCAAGGTGACTCGCAGTCTGGGGCATCTTTTCTAGTTTC

2,800 2,820 2,840 2,860 2,880

```
GATCTTCTTGAGATCCTTTTTTTCTGCGCGTAATCTGCTGCTTGCAAACAAAAAAACCACCGCTACCAGCGGTGGTTTGTTTGCCGGATCAAGAGCTACCAACTCTT
CTAGAAGAACTCTAGGAAAAAAAGACGCGCATTAGACGACGAACGTTTGTTTTTTTGGTGGCGATGGTCGCCACCAAACAAACGGCCTAGTTCTCGATGGTTGAGAA
        2,900               2,920               2,940               2,960               2,980

TTTCCGAAGGTAACTGGCTTCAGCAGAGCGCAGATACCAAATACTGTTCTTCTAGTGTAGCCGTAGTTAGGCCACCACTTCAAGAACTCTGTAGCACCGCCTACATA
AAAGGCTTCCATTGACCGAAGTCGTCTCGCGTCTATGGTTTATGACAAGAAGATCACATCGGCATCAATCCGGTGGTGAAGTTCTTGAGACATCGTGGCGGATGTAT
3,000               3,020               3,040               3,060               3,080               3,100

CCTCGCTCTGCTAATCCTGTTACCAGTGGCTGCTGCCAGTGGCGATAAGTCGTGTCTTACCGGGTTGGACTCAAGACGATAGTTACCGGATAAGGCGCAGCGGTCGG
GGAGCGAGACGATTAGGACAATGGTCACCGACGACGGTCACCGCTATTCAGCACAGAATGGCCCAACCTGAGTTCTGCTATCAATGGCCTATTCCGCGTCGCCAGCC
              3,120               3,140               3,160               3,180               3,200

GCTGAACGGGGGGTTCGTGCACACAGCCCAGCTTGGAGCGAACGACCTACACCGAACTGAGATACCTACAGCGTGAGCTATGAGAAAGCGCCACGCTTCCCGAAGGG
CGACTTGCCCCCCAAGCACGTGTGTCGGGTCGAACCTCGCTTGCTGGATGTGGCTTGACTCTATGGATGTCGCACTCGATACTCTTTCGCGGTGCGAAGGGCTTCCC
       3,220               3,240               3,260               3,280               3,300

AGAAAGGCGGACAGGTATCCGGTAAGCGGCAGGGTCGGAACAGGAGAGCGCACGAGGGAGCTTCCAGGGGGAAACGCCTGGTATCTTTATAGTCCTGTCGGGTTTCG
TCTTTCCGCCTGTCCATAGGCCATTCGCCGTCCCAGCCTTGTCCTCTCGCGTGCTCCCTCGAAGGTCCCCCTTTGCGGACCATAGAAATATCAGGACAGCCCAAAGC
3,320               3,340               3,360               3,380               3,400               3,420

CCACCTCTGACTTGAGCGTCGATTTTTGTGATGCTCGTCAGGGGGGCGGAGCCTATGGAAAAACGCCAGCAACGCGGCCTTTTTACGGTTCCTGGCCTTTTGCTGGC
GGTGGAGACTGAACTCGCAGCTAAAAACACTACGAGCAGTCCCCCCGCCTCGGATACCTTTTTGCGGTCGTTGCGCCGGAAAAATGCCAAGGACCGGAAAACGACCG
             3,440               3,460               3,480               3,500               3,520

CTTTTGCTCACATGTTCTTTCCTGCGTTATCCCCTGATTCTGTGGATAACCGTATTACCGCCTTTGAGTGAGCTGATACCGCTCGCCGCAGCCGAACGACCGAGCGC
GAAAACGAGTGTACAAGAAAGGACGCAATAGGGGACTAAGACACCTATTGGCATAATGGCGGAAACTCACTCGACTATGGCGAGCGGCGTCGGCTTGCTGGCTCGCG
      3,540               3,560               3,580               3,600               3,620

AGCGAGTCAGTGAGCGAGGAAGCGGAAGAGCGCCCAATACGCAAACCGCCTCTCCCCGCGCGTTGGCCGATTCATTAATGCAGCTGGCACGACAGGTTTCCCGACTG
TCGCTCAGTCACTCGCTCCTTCGCCTTCTCGCGGGTTATGCGTTTGGCGGAGAGGGGCGCGCAACCGGCTAAGTAATTACGTCGACCGTGCTGTCCAAAGGGCTGAC
3,640               3,660               3,680               3,700               3,720               3,740

GAAAGCGGCCAGTGAGCGCGCGCAAATTAAAGCCTTCGAGCGTCCCAAAACGCAAACCGCCTCTCCCCGCGCGTTGGCCGATTCATTAATGCAGCTGGCACGACAGG
CTTTCGCCGGTCACTCGCGCGCGTTTAATTTCGGAAGCTCGCAGGGTTTTGCGTTTGGCGGAGAGGGGCGCGCAACCGGCTAAGTAATTACGTCGACCGTGCTGTCC
            3,760               3,780               3,800               3,820               3,840

TTTCCCGACTGGAAAGCGGGCAGTGAGCGCAACGCAATTAATGTGAGTTAGCTCACTCATTAGGCACCCCAGGCTTTACACTTTATGCTTCCGGCTCGTATGTTGTG
AAAGGGCTGACCTTTCGCCCGTCACTCGCGTTGCGTTAATTACACTCAATCGAGTGAGTAATCCGTGGGGTCCGAAATGTGAAATACGAAGGCCGAGCATACAACAC
                                                                                 lac promoter
      3,860               3,880               3,900               3,920               3,940
```

TGGAATTGTGAGCGGATAACAATTTCACACAGGAAACAGCTATGACCATGATTACGCCAAGCGCGCAATTAACCCTCACTAAAGGGAACAAAAGCTGGAGCTcAGTT
ACCTTAACACTCGCCTATTGTTAAAGTGTGTCCTTTGTCGATACTGGTACTAATGCGGTTCGCGCGTTAATTGGGAGTGATTTCCCTTGTTTTCGACCTCGAgTCAA

T3
LacO M13-rev T3 promoter

3,960 3,980 4,000 4,020 4,040 4,060

TATCATTATCAATACTcGCCATTTCAAAGAATACGTAAATAATTAATAGTAGTGATTTTCCTAACTTTATTTAGTCAAAAAATTAGCCTTTTAATTCTGCTGTAACC
ATAGTAATAGTTATGAgCGGTAAAGTTTCTTATGCATTTATTAATTATCATCACTAAAAGGATTGAAATAAATCAGTTTTTTAATCGGAAAATTAAGACGACATTGG

GPD promoter

4,080 4,100 4,120 4,140 4,160

CGTACATGCCCAAAATAGGGGGCGGGTTACACAGAATATATAACATCGTAGGTGTCTGGGTGAACAGTTTATTCCTGGCATCCACTAAATATAATGGAGCCCGCTTT
GCATGTACGGGTTTTATCCCCCGCCCAATGTGTCTTATATATTGTAGCATCCACAGACCCACTTGTCAAATAAGGACCGTAGGTGATTTATATTACCTCGGGCGAAA

GPD promoter

4,180 4,200 4,220 4,240 4,260 4,280

TTAAGCTGGCATCCAGAAAAAAAAAGAATCCCAGCACCAAAATATTGTTTTCTTCACCAACCATCAGTTCATAGGTCCATTCTCTTAGCGCAACTACAGAGAACAGG
AATTCGACCGTAGGTCTTTTTTTTTCTTAGGGTCGTGGTTTTATAACAAAAGAAGTGGTTGGTAGTCAAGTATCCAGGTAAGAGAATCGCGTTGATGTCTCTTGTCC

GPD promoter

4,300 4,320 4,340 4,360 4,380

GGCACAAACAGGCAAAAAACGGGCACAACCTCAATGGAGTGATGCAACCTGCCTGGAGTAAATGATGACACAAGGCAATTGACCCACGCATGTATCTATCTCATTTT
CCGTGTTTGTCCGTTTTTTGCCCGTGTTGGAGTTACCTCACTACGTTGGACGGACCTCATTTACTACTGTGTTCCGTTAACTGGGTGCGTACATAGATAGAGTAAAA

GPD promoter

4,400 4,420 4,440 4,460 4,480

CTTACACCTTCTATTACCTTCTGCTCTCTCTGATTTGGAAAAAGCTGAAAAAAAAAGGTTGAAACCAGTTCCCTGAAATTATTCCCCTACTTGACTAATAAGTATATA
GAATGTGGAAGATAATGGAAGACGAGAGAGACTAAACCTTTTTCGACTTTTTTTTTCCAACTTTGGTCAAGGGACTTTAATAAGGGGATGAACTGATTATTCATATAT

GPD promoter

4,500 4,520 4,540 4,560 4,580 4,600

AAGACGGTAGGTATTGATTGTAATTCTGTAAATCTATTTCTTAAACTTCTTAAATTCTACTTTTATAGTTAGTCTTTTTTTTAGTTTTAAAACACCAGAACTTAGTT
TTCTGCCATCCATAACTAACATTAAGACATTTAGATAAAGAATTTGAAGAATTTAAGATGAAAATATCAATCAGAAAAAAAATCAAAATTTTGTGGTCTTGAATCAA

GPD promoter

4,620 4,640 4,660 4,680 4,700

TCGACGGATTCTAGAACTAGTGGATCCATGAGATTCCCATCTATCTTCACCGCTGTTTTGTTCGCTGCTTCTTCTGCTTTGGCTGCTCCAGCTAACACCACCACCGA
AGCTGCCTAAGATCTTGATCACCTAGGTACTCTAAGGGTAGATAGAAGTGGCGACAAAACAAGCGACGAAGAAGACGAAACCGACGAGGTCGATTGTGGTGGTGGCT

1 2 3 4 5 6 7 8 9 10 11 12 13 14 15 16 17 18 19 20 21 22 23 24 25 26 27
M R F P S I F T A V L F A A S S A L A A P A N T T T E

GPD promoter Mating factor alpha leader

4,720 4,740 4,760 4,780 4,800

AGACGAAACCGCTCAAATCCCAGCTGAAGCTGTTATCGACTACTCTGACTTGGAAGGTGACTTCGACGCTGCTGCTTTGCCATTGTCTAACTCTACCAACAACGGTT
TCTGCTTTGGCGAGTTTAGGGTCGACTTCGACAATAGCTGATGAGACTGAACCTTCCACTGAAGCTGCGACGACGAAACGGTAACAGATTGAGATGGTTGTTGCCAA
28 29 30 31 32 33 34 35 36 37 38 39 40 41 42 43 44 45 46 47 48 49 50 51 52 53 54 55 56 57 58 59 60 61 62
D E T A Q I P A E A V I D Y S D L E G D F D A A A L P L S N S T N N G

Mating factor alpha leader

4,820 4,840 4,860 4,880 4,900 4,920

TGTCTTCTACCAACACCACCATCGCTTCTATCGCTGCTAAGGAAGAAGGTGTTCAATTGGACAAGAGAGAAagatctggcggccgcgcatgcgctagcctcgagGAT
ACAGAAGATGGTTGTGGTGGTAGCGAAGATAGCGACGATTCCTTCTTCCACAAGTTAACCTGTTCTCTCTTtctagaccgccggcgcgtacgcgatcggagctcCTA
63 64 65 66 67 68 69 70 71 72 73 74 75 76 77 78 79 80 81 82 83 84 85 86
L S S T N T T I A S I A A K E E G V Q L D K R E D

Mating factor alpha leader

4,940 4,960 4,980 5,000 5,020

GTGCAGTTGCAAGCATCCGGCGGCGGTTCTGTTCAGGCTGGTGGTTCTCTAAGATTAAGTTGTGCTGCTTCAGGTTATACCATCGGCCCATACTGCATGGGTTGGTT
CACGTCAACGTTCGTAGGCCGCCGCCAAGACAAGTCCGACCACCAAGAGATTCTAATTCAACACGACGAAGTCCAATATGGTAGCCGGGTATGACGTACCCAACCAA
2 4 6 8 10 12 14 16 18 20 22 24 26 28 30 32 34 36
V Q L Q A S G G G S V Q A G G S L R L S C A A S G Y T I G P Y C M G W F

anti-lysozyme nanobody

5,040 5,060 5,080 5,100 5,120

TCGTCAAGCTCCAGGCAAAGAAAGGGAGGGAGTCGCAGCCATTAACATGGGCGGCGGTATCACATATTATGCAGACTCCGTAAAAGGTAGATTTACTATCTCTCAGG
AGCAGTTCGAGGTCCGTTTCTTTCCCTCCCTCAGCGTCGGTAATTGTACCCGCCGCCATAGTGTATAATACGTCTGAGGCATTTTCCATCTAAATGATAGAGAGTCC
38 40 42 44 46 48 50 52 54 56 58 60 62 64 66 68 70 72
R Q A P G K E R E G V A A I N M G G G I T Y Y A D S V K G R F T I S Q

anti-lysozyme nanobody

5,140 5,160 5,180 5,200 5,220 5,240

ACAATGCTAAGAACACGGTCTATCTGTTAATGAATTCATTGGAGCCCGAGGACACTGCCATATACTACTGTGCCGCAGATTCAACCATTTACGCTTCCTATTACGAA
TGTTACGATTCTTGTGCCAGATAGACAATTACTTAAGTAACCTCGGGCTCCTGTGACGGTATATGATGACACGGCGTCTAAGTTGGTAAATGCGAAGGATAATGCTT
74 76 78 80 82 84 86 88 90 92 94 96 98 100 102 104 106 108
D N A K N T V Y L L M N S L E P E D T A I Y Y C A A D S T I Y A S Y Y E

anti-lysozyme nanobody

5,260 5,280 5,300 5,320 5,340

TGCGGACACGGATTAAGTACTGGAGGTTACGGTTATGATTCCTGGGGTCAAGGCACACAAGTCACGGTATCATCAGGCGGAGGAGGTTCCGGTGGAGGGGGGTCAGA
ACGCCTGTGCCTAATTCATGACCTCCAATGCCAATACTAAGGACCCCAGTTCCGTGTGTTCAGTGCCATAGTAGTCCGCCTCCTCCAAGGCCACCTCCCCCCAGTCT
110 112 114 116 118 120 122 124 126 128 130 132 1 2 3 4 5 6 7 8 9 10 1
C G H G L S T G G Y G Y D S W G Q G T Q V T V S S G G G G S G G G G S D

anti-lysozyme nanobody

5,360 5,380 5,400 5,420 5,440

CTATAAGGATGACGACGATAAGGCTGGAGGTTCGAGGGGAGTCCCTCACATAGTGATGGTGGATGCTTACAAAAGGTATAAATAACCCGGGCTGCAGGAATTCGATA
GATATTCCTACTGCTGCTATTCCGACCTCCAAGCTCCCCTCAGGGAGTGTATCACTACCACCTACGAATGTTTTCCATATTTATTGGGCCCGACGTCCTTAAGCTAT
2 3 4 5 6 7 8 1 2 3 4 1 2 3 4 5 6 7 8 9 10 11 12 13 14 15 16 1
Y K D D D D K A G G S R G V P H I V M V D A Y K R Y K *
FLAG tag SpyTag
5,460 5,480 5,500 5,520 5,540 5,560

TCAAGCTTATCGATACCGTCGACCTCGAGTCATGTAATTAGTTATGTCACGCTTACGTTCACGCCCTCCCCCCACATCCGCTCTAACCGAAAAGGAAGGAGTTAGAC
AGTTCGAATAGCTATGGCAGCTGGAGCTCAGTACATTAATCAATACAGTGCGAATGCAAGTGCGGGAGGGGGGTGTAGGCGAGATTGGCTTTTCCTTCCTCAATCTG
CYC1 terminator
5,580 5,600 5,620 5,640 5,660

AACCTGAAGTCTAGGTCCCTATTTATTTTTTTATAGTTATGTTAGTATTAAGAACGTTATTTATATTTCAAATTTTTCTTTTTTTTCTGTACAGACGCGTGTACGCA
TTGGACTTCAGATCCAGGGATAAATAAAAAAATATCAATACAATCATAATTCTTGCAATAAATATAAAGTTTAAAAAGAAAAAAAAGACATGTCTGCGCACATGCGT
CYC1 terminator
5,680 5,700 5,720 5,740 5,760

TGTAACATTATACTGAAAACCTTGCTTGAGAAGGTTTTGGGACGCTCGAAGGCTTTAATTTGCGCGCGCTCACTGGCCGTCGTTTTACAACGTCGTGACTGGGAAAA
ACATTGTAATATGACTTTTGGAACGAACTCTTCCAAAACCCTGCGAGCTTCCGAAATTAAACGCGCGCGAGTGACCGGCAGCAAAATGTTGCAGCACTGACCCTTTT
CYC1 terminator M13-fwd
5,780 5,800 5,820 5,840 5,860 5,880

CCCTGGCGTTACCCAACTTAATCGCCTTGCAGCACATCCCCCTTTCGCCAGCTGGCGTAATAGCGAAGAGGCCCGCACCGATCGCCCTTCCCAACAGTTGCGCAGCC
GGGACCGCAATGGGTTGAATTAGCGGAACGTCGTGTAGGGGGAAAGCGGTCGACCGCATTATCGCTTCTCCGGGCGTGGCTAGCGGGAAGGGTTGTCAACGCGTCGG
LacZ alpha
5,900 5,920 5,940 5,960 5,980

TGAATGGCGAATGGACGCGCCCTGTAGCGGCGCATTAAGCGCGGCGGGTGTGGTGGTTACGCGCAGCGTGACCACTACACTTGCCAGCGCCCTAGCGCCCGCTCCTT
ACTTACCGCTTACCTGCGCGGGACATCGCCGCGTAATTCGCGCCGCCCACACCACCAATGCGCGTCGCACTGGTGATGTGAACGGTCGCGGGATCGCGGGCGAGGAA
LacZ...pha
6,000 6,020 6,040 6,060 6,080

TCGCTTTCTTCCCTTCCTTTCTCGCCACGTTCGCCGGCTTTCCCCGTCAAGCTCTAAATCGGGGGCTCCCTTTAGGGTTCCGATTTAGTGCTTTACGGCACCTCGAC
AGCGAAAGAAGGGAAGGAAAGAGCGGTGCAAGCGGCCGAAAGGGGCAGTTCGAGATTTAGCCCCCGAGGGAAATCCCAAGGCTAAATCACGAAATGCCGTGGAGCTG
6,100 6,120 6,140 6,160 6,180 6,200

CCCAAAAAACTTGATTAGGGTGATGGTTCACGTAGTGGGCCATCGCCCTGATAGACGGTTTTTCGCCCTTTGACGTTGGAGTCCACGTTCTTTAATAGTGGACTCTT
GGGTTTTTTGAACTAATCCCACTACCAAGTGCATCACCCGGTAGCGGGACTATCTGCCAAAAAGCGGGAAACTGCAACCTCAGGTGCAAGAAATTATCACCTGAGAA
6,220 6,240 6,260 6,280 6,300

GTTCCAAACTGGAACAACACTCAACCCTATCTCGGTCTATTCTTTTGATTTATAAGGGATTTTGCCGATTTCGGCCTATTGGTTAAAAAATGAGCTGATTTAACAAA
CAAGGTTTGACCTTGTTGTGAGTTGGGATAGAGCCAGATAAGAAAACTAAATATTCCCTAAAACGGCTAAAGCCGGATAACCAATTTTTTACTCGACTAAATTGTTT
6,320 6,340 6,360 6,380 6,400 6,420

AATTTAACGCGAATTTTAACAAAATATTAACGCTTACAATTTCCTGATGCGGTATTTTCTCCTTACGCATCTGTGCGGTATTTCACACCGCATAGGGTAATAACTGA
TTAAATTGCGCTTAAAATTGTTTTATAATTGCGAATGTTAAAGGACTACGCCATAAAAGAGGAATGCGTAGACACGCCATAAAGTGTGGCGTATCCCATTATTGACT

6,440 6,460 6,480 6,500 6,520

TATAATTAAATTGAAGCTCTAATTTGTGAGTTTAGTATACATGCATTTACTTATAATACAGTTTTTTAAGCAAGGATTTTCTTAACTTCTTCGGCGACAGCATCACC
ATATTAATTTAACTTCGAGATTAAACACTCAAATCATATGTACGTAAATGAATATTATGTCAAAAAATTCGTTCCTAAAAGAATTGAAGAAGCCGCTGTCGTAGTGG
LEU2

6,540 6,560 6,580 6,600 6,620

GACTTCGGTGGTACTGTTGGAACCACCTAAATCACCAGTTCTGATACCTGCATCCAAAACCTTTTTAACTGCATCTTCAATGGCCTTACCTTCTTCAGGCAAGTTCA
CTGAAGCCACCATGACAACCTTGGTGGATTTAGTGGTCAAGACTATGGACGTAGGTTTTGGAAAAATTGACGTAGAAGTTACCGGAATGGAAGAAGTCCGTTCAAGT
LEU2

6,640 6,660 6,680 6,700 6,720 6,740

ATGACAATTTCAACATCATTGCAGCAGACAAGATAGTGGCGATAGGGTTGACCTTATTCTTTGGCAAATCTGGAGCAGAACCGTGGCATGGTTCGTACAAACCAAAT
TACTGTTAAAGTTGTAGTAACGTCGTCTGTTCTATCACCGCTATCCCAACTGGAATAAGAAACCGTTTAGACCTCGTCTTGGCACCGTACCAAGCATGTTTGGTTTA
LEU2

6,760 6,780 6,800 6,820 6,840

GCGGTGTTCTTGTCTGGCAAAGAGGCCAAGGACGCAGATGGCAACAAACCCAAGGAACCTGGGATAACGGAGGCTTCATCGGAGATGATATCACCAAACATGTTGCT
CGCCACAAGAACAGACCGTTTCTCCGGTTCCTGCGTCTACCGTTGTTTGGGTTCCTTGGACCCTATTGCCTCCGAAGTAGCCTCTACTATAGTGGTTTGTACAACGA
LEU2

6,860 6,880 6,900 6,920 6,940

GGTGATTATAATACCATTTAGGTGGGTTGGGTTCTTAACTAGGATCATGGCGGCAGAATCAATCAATTGATGTTGAACCTTCAATGTAGGGAATTCGTTCTTGATGG
CCACTAATATTATGGTAAATCCACCCAACCCAAGAATTGATCCTAGTACCGCCGTCTTAGTTAGTTAACTACAACTTGGAAGTTACATCCCTTAAGCAAGAACTACC
LEU2

6,960 6,980 7,000 7,020 7,040 7,060

TTTCCTCCACAGTTTTTCTCCATAATCTTGAAGAGGCCAAAACATTAGCTTTATCCAAGGACCAAATAGGCAAT
AAAGGAGGTGTCAAAAAGAGGTATTAGAACTTCTCCGGTTTTGTAATCGAAATAGGTTCCTGGTTTATCCGTTA
LEU2

7,070 7,080 7,090 7,100 7,110 7,120 7,130

ST/SC ligation / pNb_lys Nb-6×His (pRS415) (713...

GGTGGCTCATGTTGTAGGGCCATGAAAGCGGCCATTCTTGTGATTCTTTGCACTTCTGGAACGGTGTATTGTTCACTATCCCAAGCGACACCATCACCATCGTCTTC
CCACCGAGTACAACATCCCGGTACTTTCGCCGGTAAGAACACTAAGAAACGTGAAGACCTTGCCACATAACAAGTGATAGGGTTCGCTGTGGTAGTGGTAGCAGAAG
LEU2
20 40 60 80 100

CTTTCTCTTACCAAAGTAAATACCTCCCACTAATTCTCTGACAACAACGAAGTCAGTACCTTTAGCAAATTGTGGCTTGATTGGAGATAAGTCTAAAAGAGAGTCGG
GAAAGAGAATGGTTTCATTTATGGAGGGTGATTAAGAGACTGTTGTTGCTTCAGTCATGGAAATCGTTTAACACCGAACTAACCTCTATTCAGATTTTCTCTCAGCC
LEU2
120 140 160 180 200

ATGCAAAGTTACATGGTCTTAAGTTGGCGTACAATTGAAGTTCTTTACGGATTTTTAGTAAACCTTGTTCAGGTCTAACACTACCGGTACCCCATTTAGGACCACCC
TACGTTTCAATGTACCAGAATTCAACCGCATGTTAACTTCAAGAAATGCCTAAAAATCATTTGGAACAAGTCCAGATTGTGATGGCCATGGGGTAAATCCTGGTGGG
LEU2
220 240 260 280 300 320

ACAGCACCTAACAAAACGGCATCAGCCTTCTTGGAGGCTTCCAGCGCCTCATCTGGAAGTGGAACACCTGTAGCATCGATAGCAGCACCACCAATTAAATGATTTTC
TGTCGTGGATTGTTTTGCCGTAGTCGGAAGAACCTCCGAAGGTCGCGGAGTAGACCTTCACCTTGTGGACATCGTAGCTATCGTCGTGGTGGTTAATTTACTAAAAG
LEU2
340 360 380 400 420

GAAATCGAACTTGACATTGGAACGAACATCAGAAATAGCTTTAAGAACCTTAATGGCTTCGGCTGTGATTTCTTGACCAACGTGGTCACCTGGCAAAACGACGATCT
CTTTAGCTTGAACTGTAACCTTGCTTGTAGTCTTTATCGAAATTCTTGGAATTACCGAAGCCGACACTAAAGAACTGGTTGCACCAGTGGACCGTTTTGCTGCTAGA
LEU2
440 460 480 500 520

TCTTAGGGGCAGACATTAGAATGGTATATCCTTGAAATATATATATATATATATNTNGCTGAAATGTAAAAGGTAAGAAAAGTTAGAAAGTAAGACGATTGCTAACCAC
AGAATCCCCGTCTGTAATCTTACCATATAGGAACTTTATATATATATATATATANANCGACTTTACATTTTCCATTCTTTTCAATCTTTCATTCTGCTAACGATTGGTG
LEU2
540 560 580 600 620 640

CTATTGGAAAAAAACAATAGGTCCTTAAATAATATTGTCAACTTCAAGTATTGTGATGCAAGCATTTAGTCATGAACGCTTCTCTATTCTATATGAAAAGCCGGTTCC
GATAACCTTTTTTTGTTATCCAGGAATTTATTATAACAGTTGAAGTTCATAACACTACGTTCGTAAATCAGTACTTGCGAAGAGATAAGATATACTTTTCGGCCAAGG
660 680 700 720 740

GGCGCTCTCACCTTTCCTTTTTCTCCCAATTTTTCAGTTGAAAAAGGTATATGCGTCAGGCGACCTCTGAAATTAACAAAAAATTTCCAGTCATCGAATTTGATTCT
CCGCGAGAGTGGAAAGGAAAAAGAGGGTTAAAAAGTCAACTTTTTCCATATACGCAGTCCGCTGGAGACTTTAATTGTTTTTTAAAGGTCAGTAGCTTAAACTAAGA
760 780 800 820 840

GTGCGATAGCGCCCCTGTGTGTTCTCGTTATGTTGAGGAAAAAAATAATGGTTGCTAAGAGATTCGAACTCTTGCATCTTACGATACCTGAGTATTCCCACAGTTTG
CACGCTATCGCGGGGACACACAAGAGCAATACAACTCCTTTTTTTATTACCAACGATTCTCTAAGCTTGAGAACGTAGAATGCTATGGACTCATAAGGGTGTCAAAC
860 880 900 920 940 960

```
AAAAGCTGTGGTATGGTGCACTCTCAGTACAATCTGCTCTGATGCCGCATAGTTAAGCCAGCCCCGACACCCGCCAACACCCGCTGACGCGCCCTGACGGGCTTGTC
TTTTCGACACCATACCACGTGAGAGTCATGTTAGACGAGACTACGGCGTATCAATTCGGTCGGGGCTGTGGGCGGTTGTGGGCGACTGCGCGGGACTGCCCGAACAG
                980            1,000          1,020          1,040          1,060

TGCTCCCGGCATCCGCTTACAGACAAGCTGTGACCGTCTCCGGGAGCTGCATGTGTCAGAGGTTTTCACCGTCATCACCGAAACGCGCGAGACGAAAGGGCCTCGTG
ACGAGGGCCGTAGGCGAATGTCTGTTCGACACTGGCAGAGGCCCTCGACGTACACAGTCTCCAAAAGTGGCAGTAGTGGCTTTGCGCGCTCTGCTTTCCCGGAGCAC
         1,080          1,100          1,120          1,140          1,160

ATACGCCTATTTTTATAGGTTAATGTCATGATAATAATGGTTTCTTAGTAgatcgcttgcctgtaacttacacgcgcctcgtatcttttaatgatggaataatttgg
TATGCGGATAAAAATATCCAATTACAGTACTATTATTACCAAAGAATCATctagcgaacggacattgaatgtgcgcggagcatagaaaattactaccttattaaacc
                                                  CEN/ARS
1,180          1,200          1,220          1,240          1,260          1,280

gaatttactctgtgtttatttatttttatgttttgtatttggattttagaaagtaaataaagaaggtagaagagttacggaatgaagaaaaaaaaataaacaaaggt
cttaaatgagacacaaataaataaaaatacaaaacataaacctaaaatctttcatttatttcttccatcttctcaatgccttacttctttttttttatttgtttcca
CEN/ARS
               1,300          1,320          1,340          1,360          1,380

ttaaaaaatttcaacaaaaagcgtactttacatatatatttattagacaagaaaagcagattaaatagatatacattcgattaacgataagtaaaatgtaaaatcac
aatttttttaaagttgtttttcgcatgaaatgtatatataaataatctgttcttttcgtctaatttatctatatgtaagctaattgctattcattttacattttagtg
CEN/ARS
      1,400          1,420          1,440          1,460          1,480

aggattttcgtgtgtggtcttctacacagacaagatgaaacaattcggcattaatacctgagagcaggaagagcaagataaaaggtagtatttgttggcgatccccc
tcctaaaagcacacaccagaagatgtgtctgttctactttgttaagccgtaattatggactctcgtccttctcgttctattttccatcataaacaaccgctaggggg
CEN/ARS
1,500          1,520          1,540          1,560          1,580          1,600

tagagtcttttacatcttcggaaaacaaaaactatttttttctttaatttctttttttactttctatttttaatttatatatttatattaaaaaatttaaattataat
atctcagaaaatgtagaagccttttgtttttgataaaaaaagaaattaaagaaaaaaatgaaagataaaaattaaatatataaatataatttttttaaatttaatatta
CEN/ARS
             1,620          1,640          1,660          1,680          1,700

tatttttatagcacgtgatGTTCAGGTGGCACTTTTCGGGGAAATGTGCGCGGAACCCCTATTTGTTTATTTTTCTAAATACATTCAAATATGTATCCGCTCATGAG
ataaaaatatcgtgcactaCAAGTCCACCGTGAAAAGCCCCTTTACACGCGCCTTGGGGATAAACAAATAAAAAGATTTATGTAAGTTTATACATAGGCGAGTACTC
CEN/ARS
    1,720          1,740          1,760          1,780          1,800

ACAATAACCCTGATAAATGCTTCAATAATATTGAAAAAGGAAGAGTATGAGTATTCAACATTTCCGTGTCGCCCTTATTCCCTTTTTTGCGGCATTTTGCCTTCCTG
TGTTATTGGGACTATTTACGAAGTTATTATAACTTTTTCCTTCTCATACTCATAAGTTGTAAAGGCACAGCGGGAATAAGGGAAAAAACGCCGTAAAACGGAAGGAC
1,820          1,840          1,860          1,880          1,900          1,920
```

TTTTTGCTCACCCAGAAACGCTGGTGAAAGTAAAAGATGCTGAAGATCAGTTGGGTGCACGAGTGGGTTACATCGAACTGGATCTCAACAGCGGTAAGATCCTTGAG
AAAAACGAGTGGGTCTTTGCGACCACTTTCATTTTCTACGACTTCTAGTCAACCCACGTGCTCACCCAATGTAGCTTGACCTAGAGTTGTCGCCATTCTAGGAACTC

1,940 1,960 1,980 2,000 2,020

AGTTTTCGCCCCGAAGAACGTTTTCCAATGATGAGCACTTTTAAAGTTCTGCTATGTGGCGCGGTATTATCCCGTATTGACGCCGGGCAAGAGCAACTCGGTCGCCG
TCAAAAGCGGGGCTTCTTGCAAAAGGTTACTACTCGTGAAAATTTCAAGACGATACACCGCGCCATAATAGGGCATAACTGCGGCCCGTTCTCGTTGAGCCAGCGGC

AmpR

2,040 2,060 2,080 2,100 2,120 2,140

CATACACTATTCTCAGAATGACTTGGTTGAGTACTCACCAGTCACAGAAAAGCATCTTACGGATGGCATGACAGTAAGAGAATTATGCAGTGCTGCCATAACCATGA
GTATGTGATAAGAGTCTTACTGAACCAACTCATGAGTGGTCAGTGTCTTTTCGTAGAATGCCTACCGTACTGTCATTCTCTTAATACGTCACGACGGTATTGGTACT

AmpR

2,160 2,180 2,200 2,220 2,240

GTGATAACACTGCGGCCAACTTACTTCTGACAACGATCGGAGGACCGAAGGAGCTAACCGCTTTTTTGCACAACATGGGGGATCATGTAACTCGCCTTGATCGTTGG
CACTATTGTGACGCCGGTTGAATGAAGACTGTTGCTAGCCTCCTGGCTTCCTCGATTGGCGAAAAAACGTGTTGTACCCCCTAGTACATTGAGCGGAACTAGCAACC

AmpR

2,260 2,280 2,300 2,320 2,340

GAACCGGAGCTGAATGAAGCCATACCAAACGACGAGCGTGACACCACGATGCCTGTAGCAATGGCAACAACGTTGCGCAAACTATTAACTGGCGAACTACTTACTCT
CTTGGCCTCGACTTACTTCGGTATGGTTTGCTGCTCGCACTGTGGTGCTACGGACATCGTTACCGTTGTTGCAACGCGTTTGATAATTGACCGCTTGATGAATGAGA

AmpR

2,360 2,380 2,400 2,420 2,440 2,460

AGCTTCCCGGCAACAATTAATAGACTGGATGGAGGCGGATAAAGTTGCAGGACCACTTCTGCGCTCGGCCCTTCCGGCTGGCTGGTTTATTGCTGATAAATCTGGAG
TCGAAGGGCCGTTGTTAATTATCTGACCTACCTCCGCCTATTTCAACGTCCTGGTGAAGACGCGAGCCGGGAAGGCCGACCGACCAAATAACGACTATTTAGACCTC

AmpR

2,480 2,500 2,520 2,540 2,560

CCGGTGAGCGTGGGTCTCGCGGTATCATTGCAGCACTGGGGCCAGATGGTAAGCCCTCCCGTATCGTAGTTATCTACACGACGGGGAGTCAGGCAACTATGGATGAA
GGCCACTCGCACCCAGAGCGCCATAGTAACGTCGTGACCCCGGTCTACCATTCGGGAGGGCATAGCATCAATAGATGTGCTGCCCCTCAGTCCGTTGATACCTACTT

AmpR

2,580 2,600 2,620 2,640 2,660

CGAAATAGACAGATCGCTGAGATAGGTGCCTCACTGATTAAGCATTGGTAACTGTCAGACCAAGTTTACTCATATATACTTTAGATTGATTTAAAACTTCATTTTTA
GCTTTATCTGTCTAGCGACTCTATCCACGGAGTGACTAATTCGTAACCATTGACAGTCTGGTTCAAATGAGTATATATGAAATCTAACTAAATTTTGAAGTAAAAAT

AmpR

2,680 2,700 2,720 2,740 2,760 2,780

ATTTAAAAGGATCTAGGTGAAGATCCTTTTTGATAATCTCATGACCAAAATCCCTTAACGTGAGTTTTCGTTCCACTGAGCGTCAGACCCCGTAGAAAAGATCAAAG
TAAATTTTCCTAGATCCACTTCTAGGAAAAACTATTAGAGTACTGGTTTTAGGGAATTGCACTCAAAAGCAAGGTGACTCGCAGTCTGGGGCATCTTTTCTAGTTTC

2,800 2,820 2,840 2,860 2,880

```
GATCTTCTTGAGATCCTTTTTTTCTGCGCGTAATCTGCTGCTTGCAAACAAAAAAACCACCGCTACCAGCGGTGGTTTGTTTGCCGGATCAAGAGCTACCAACTCTT
CTAGAAGAACTCTAGGAAAAAAAGACGCGCATTAGACGACGAACGTTTGTTTTTTTGGTGGCGATGGTCGCCACCAAACAAACGGCCTAGTTCTCGATGGTTGAGAA
        2,900               2,920               2,940               2,960               2,980

TTTCCGAAGGTAACTGGCTTCAGCAGAGCGCAGATACCAAATACTGTTCTTCTAGTGTAGCCGTAGTTAGGCCACCACTTCAAGAACTCTGTAGCACCGCCTACATA
AAAGGCTTCCATTGACCGAAGTCGTCTCGCGTCTATGGTTTATGACAAGAAGATCACATCGGCATCAATCCGGTGGTGAAGTTCTTGAGACATCGTGGCGGATGTAT
3,000               3,020               3,040               3,060               3,080               3,100

CCTCGCTCTGCTAATCCTGTTACCAGTGGCTGCTGCCAGTGGCGATAAGTCGTGTCTTACCGGGTTGGACTCAAGACGATAGTTACCGGATAAGGCGCAGCGGTCGG
GGAGCGAGACGATTAGGACAATGGTCACCGACGACGGTCACCGCTATTCAGCACAGAATGGCCCAACCTGAGTTCTGCTATCAATGGCCTATTCCGCGTCGCCAGCC
              3,120               3,140               3,160               3,180               3,200

GCTGAACGGGGGGTTCGTGCACACAGCCCAGCTTGGAGCGAACGACCTACACCGAACTGAGATACCTACAGCGTGAGCTATGAGAAAGCGCCACGCTTCCCGAAGGG
CGACTTGCCCCCCAAGCACGTGTGTCGGGTCGAACCTCGCTTGCTGGATGTGGCTTGACTCTATGGATGTCGCACTCGATACTCTTTCGCGGTGCGAAGGGCTTCCC
       3,220               3,240               3,260               3,280               3,300

AGAAAGGCGGACAGGTATCCGGTAAGCGGCAGGGTCGGAACAGGAGAGCGCACGAGGGAGCTTCCAGGGGGAAACGCCTGGTATCTTTATAGTCCTGTCGGGTTTCG
TCTTTCCGCCTGTCCATAGGCCATTCGCCGTCCCAGCCTTGTCCTCTCGCGTGCTCCCTCGAAGGTCCCCCTTTGCGGACCATAGAAATATCAGGACAGCCCAAAGC
3,320               3,340               3,360               3,380               3,400               3,420

CCACCTCTGACTTGAGCGTCGATTTTTGTGATGCTCGTCAGGGGGGCGGAGCCTATGGAAAAACGCCAGCAACGCGGCCTTTTTACGGTTCCTGGCCTTTTGCTGGC
GGTGGAGACTGAACTCGCAGCTAAAAACACTACGAGCAGTCCCCCCGCCTCGGATACCTTTTTGCGGTCGTTGCGCCGGAAAAATGCCAAGGACCGGAAAACGACCG
             3,440               3,460               3,480               3,500               3,520

CTTTTGCTCACATGTTCTTTCCTGCGTTATCCCCTGATTCTGTGGATAACCGTATTACCGCCTTTGAGTGAGCTGATACCGCTCGCCGCAGCCGAACGACCGAGCGC
GAAAACGAGTGTACAAGAAAGGACGCAATAGGGGACTAAGACACCTATTGGCATAATGGCGGAAACTCACTCGACTATGGCGAGCGGCGTCGGCTTGCTGGCTCGCG
      3,540               3,560               3,580               3,600               3,620

AGCGAGTCAGTGAGCGAGGAAGCGGAAGAGCGCCCAATACGCAAACCGCCTCTCCCCGCGCGTTGGCCGATTCATTAATGCAGCTGGCACGACAGGTTTCCCGACTG
TCGCTCAGTCACTCGCTCCTTCGCCTTCTCGCGGGTTATGCGTTTGGCGGAGAGGGGCGCGCAACCGGCTAAGTAATTACGTCGACCGTGCTGTCCAAAGGGCTGAC
3,640               3,660               3,680               3,700               3,720               3,740

GAAAGCGGCCAGTGAGCGCGCGCAAATTAAAGCCTTCGAGCGTCCCAAAACGCAAACCGCCTCTCCCCGCGCGTTGGCCGATTCATTAATGCAGCTGGCACGACAGG
CTTTCGCCGGTCACTCGCGCGCGTTTAATTTCGGAAGCTCGCAGGGTTTTGCGTTTGGCGGAGAGGGGCGCGCAACCGGCTAAGTAATTACGTCGACCGTGCTGTCC
            3,760               3,780               3,800               3,820               3,840

TTTCCCGACTGGAAAGCGGGCAGTGAGCGCAACGCAATTAATGTGAGTTAGCTCACTCATTAGGCACCCCAGGCTTTACACTTTATGCTTCCGGCTCGTATGTTGTG
AAAGGGCTGACCTTTCGCCCGTCACTCGCGTTGCGTTAATTACACTCAATCGAGTGAGTAATCCGTGGGGTCCGAAATGTGAAATACGAAGGCCGAGCATACAACAC
                                                                          [lac promoter>
      3,860               3,880               3,900               3,920               3,940
```

TGGAATTGTGAGCGGATAACAATTTCACACAGGAAACAGCTATGACCATGATTACGCCAAGCGCGCAATTAACCCTCACTAAAGGGAACAAAAGCTGGAGCTcAGTT
ACCTTAACACTCGCCTATTGTTAAAGTGTGTCCTTTGTCGATACTGGTACTAATGCGGTTCGCGCGTTAATTGGGAGTGATTTCCCTTGTTTTCGACCTCGAgTCAA

T3
LacO M13-rev T3 promoter

3,960 3,980 4,000 4,020 4,040 4,060

TATCATTATCAATACTcGCCATTTCAAAGAATACGTAAATAATTAATAGTAGTGATTTTCCTAACTTTATTTAGTCAAAAAATTAGCCTTTTAATTCTGCTGTAACC
ATAGTAATAGTTATGAgCGGTAAAGTTTCTTATGCATTTATTAATTATCATCACTAAAAGGATTGAAATAAATCAGTTTTTTAATCGGAAAATTAAGACGACATTGG

GPD promoter

4,080 4,100 4,120 4,140 4,160

CGTACATGCCCAAAATAGGGGGCGGGTTACACAGAATATATAACATCGTAGGTGTCTGGGTGAACAGTTTATTCCTGGCATCCACTAAATATAATGGAGCCCGCTTT
GCATGTACGGGTTTTATCCCCCGCCCAATGTGTCTTATATATTGTAGCATCCACAGACCCACTTGTCAAATAAGGACCGTAGGTGATTTATATTACCTCGGGCGAAA

GPD promoter

4,180 4,200 4,220 4,240 4,260 4,280

TTAAGCTGGCATCCAGAAAAAAAAAAGAATCCCAGCACCAAAATATTGTTTTCTTCACCAACCATCAGTTCATAGGTCCATTCTCTTAGCGCAACTACAGAGAACAGG
AATTCGACCGTAGGTCTTTTTTTTTTCTTAGGGTCGTGGTTTTATAACAAAAGAAGTGGTTGGTAGTCAAGTATCCAGGTAAGAGAATCGCGTTGATGTCTCTTGTCC

GPD promoter

4,300 4,320 4,340 4,360 4,380

GGCACAAACAGGCAAAAAACGGGCACAACCTCAATGGAGTGATGCAACCTGCCTGGAGTAAATGATGACACAAGGCAATTGACCCACGCATGTATCTATCTCATTTT
CCGTGTTTGTCCGTTTTTTGCCCGTGTTGGAGTTACCTCACTACGTTGGACGGACCTCATTTACTACTGTGTTCCGTTAACTGGGTGCGTACATAGATAGAGTAAAA

GPD promoter

4,400 4,420 4,440 4,460 4,480

CTTACACCTTCTATTACCTTCTGCTCTCTCTGATTTGGAAAAAGCTGAAAAAAAAAGGTTGAAACCAGTTCCCTGAAATTATTCCCCTACTTGACTAATAAGTATATA
GAATGTGGAAGATAATGGAAGACGAGAGAGACTAAACCTTTTTCGACTTTTTTTTTCCAACTTTGGTCAAGGGACTTTAATAAGGGGATGAACTGATTATTCATATAT

GPD promoter

4,500 4,520 4,540 4,560 4,580 4,600

AAGACGGTAGGTATTGATTGTAATTCTGTAAATCTATTTCTTAAACTTCTTAAATTCTACTTTTATAGTTAGTCTTTTTTTTAGTTTTAAAACACCAGAACTTAGTT
TTCTGCCATCCATAACTAACATTAAGACATTTAGATAAAGAATTTGAAGAATTTAAGATGAAAATATCAATCAGAAAAAAAATCAAAATTTTGTGGTCTTGAATCAA

GPD promoter

4,620 4,640 4,660 4,680 4,700

TCGACGGATTCTAGAACTAGTGGATCCATGAGATTCCCATCTATCTTCACCGCTGTTTTGTTCGCTGCTTCTTCTGCTTTGGCTGCTCCAGCTAACACCACCACCGA
AGCTGCCTAAGATCTTGATCACCTAGGTACTCTAAGGGTAGATAGAAGTGGCGACAAAACAAGCGACGAAGAAGACGAAACCGACGAGGTCGATTGTGGTGGTGGCT

1 2 3 4 5 6 7 8 9 10 11 12 13 14 15 16 17 18 19 20 21 22 23 24 25 26 27
M R F P S I F T A V L F A A S S A L A A P A N T T T E

GPD promoter Mating factor alpha leader

4,720 4,740 4,760 4,780 4,800

AGACGAAACCGCTCAAATCCCAGCTGAAGCTGTTATCGACTACTCTGACTTGGAAGGTGACTTCGACGCTGCTGCTTTGCCATTGTCTAACTCTACCAACAACGGTT
TCTGCTTTGGCGAGTTTAGGGTCGACTTCGACAATAGCTGATGAGACTGAACCTTCCACTGAAGCTGCGACGACGAAACGGTAACAGATTGAGATGGTTGTTGCCAA
28 29 30 31 32 33 34 35 36 37 38 39 40 41 42 43 44 45 46 47 48 49 50 51 52 53 54 55 56 57 58 59 60 61 62
D E T A Q I P A E A V I D Y S D L E G D F D A A A L P L S N S T N N G
Mating factor alpha leader
4,820 4,840 4,860 4,880 4,900 4,920

TGTCTTCTACCAACACCACCATCGCTTCTATCGCTGCTAAGGAAGAAGGTGTTCAATTGGACAAGAGAGAAagatctggcggccgcgcatgcgctagcctcgagGAT
ACAGAAGATGGTTGTGGTGGTAGCGAAGATAGCGACGATTCCTTCTTCCACAAGTTAACCTGTTCTCTCTTtctagaccgccggcgcgtacgcgatcggagctcCTA
63 64 65 66 67 68 69 70 71 72 73 74 75 76 77 78 79 80 81 82 83 84 85 86
L S S T N T T I A S I A A K E E G V Q L D K R E D
Mating factor alpha leader
4,940 4,960 4,980 5,000 5,020

GTGCAGTTGCAAGCATCCGGCGGCGGTTCTGTTCAGGCTGGTGGTTCTCTAAGATTAAGTTGTGCTGCTTCAGGTTATACCATCGGCCCATACTGCATGGGTTGGTT
CACGTCAACGTTCGTAGGCCGCCGCCAAGACAAGTCCGACCACCAAGAGATTCTAATTCAACACGACGAAGTCCAATATGGTAGCCGGGTATGACGTACCCAACCAA
2 4 6 8 10 12 14 16 18 20 22 24 26 28 30 32 34 36
V Q L Q A S G G G S V Q A G G S L R L S C A A S G Y T I G P Y C M G W F
anti-lysozyme nanobody
5,040 5,060 5,080 5,100 5,120

TCGTCAAGCTCCAGGCAAAGAAAGGGAGGGAGTCGCAGCCATTAACATGGGCGGCGGTATCACATATTATGCAGACTCCGTAAAAGGTAGATTTACTATCTCTCAGG
AGCAGTTCGAGGTCCGTTTCTTTCCCTCCCTCAGCGTCGGTAATTGTACCCGCCGCCATAGTGTATAATACGTCTGAGGCATTTTCCATCTAAATGATAGAGAGTCC
38 40 42 44 46 48 50 52 54 56 58 60 62 64 66 68 70 72
R Q A P G K E R E G V A A I N M G G G I T Y Y A D S V K G R F T I S Q
anti-lysozyme nanobody
5,140 5,160 5,180 5,200 5,220 5,240

ACAATGCTAAGAACACGGTCTATCTGTTAATGAATTCATTGGAGCCCGAGGACACTGCCATATACTACTGTGCCGCAGATTCAACCATTTACGCTTCCTATTACGAA
TGTTACGATTCTTGTGCCAGATAGACAATTACTTAAGTAACCTCGGGCTCCTGTGACGGTATATGATGACACGGCGTCTAAGTTGGTAAATGCGAAGGATAATGCTT
74 76 78 80 82 84 86 88 90 92 94 96 98 100 102 104 106 108
D N A K N T V Y L L M N S L E P E D T A I Y Y C A A D S T I Y A S Y Y E
anti-lysozyme nanobody
5,260 5,280 5,300 5,320 5,340

TGCGGACACGGATTAAGTACTGGAGGTTACGGTTATGATTCCTGGGGTCAAGGCACACAAGTCACGGTATCATCAGGCGGAGGAGGTTCCGGTGGAGGGGGGTCACA
ACGCCTGTGCCTAATTCATGACCTCCAATGCCAATACTAAGGACCCCAGTTCCGTGTGTTCAGTGCCATAGTAGTCCGCCTCCTCCAAGGCCACCTCCCCCCAGTGT
110 112 114 116 118 120 122 124 126 128 130 132 1 2 3 4 5 6 7 8 9 10 1
C G H G L S T G G Y G Y D S W G Q G T Q V T V S S G G G G S G G G G S H
anti-lysozyme nanobody
5,360 5,380 5,400 5,420 5,440

```
CCATCATCATCATCACGCTGGAGGTTCGAGGGGAGTCCCTCACATAGTGATGGTGGATGCTTACAAAAGGTATAAATAACCCGGGCTGCAGGAATTCGATATCAAGC
GGTAGTAGTAGTAGTGCGACCTCCAAGCTCCCCTCAGGGAGTGTATCACTACCACCTACGAATGTTTTCCATATTTATTGGGCCCGACGTCCTTAAGCTATAGTTCG
  2   3   4   5   6   1   2   3   4   1   2   3   4   5   6   7   8   9   10  11  12  13  14  15  16  1
  H   H   H   H   H   A   G   G   S   R   G   V   P   H   I   V   M   V   D   A   Y   K   R   Y   K   *
6×His tag                    SpyTag
5,460   5,480   5,500   5,520   5,540   5,560

TTATCGATACCGTCGACCTCGAGTCATGTAATTAGTTATGTCACGCTTACGTTCACGCCCTCCCCCCACATCCGCTCTAACCGAAAAGGAAGGAGTTAGACAACCTG
AATAGCTATGGCAGCTGGAGCTCAGTACATTAATCAATACAGTGCGAATGCAAGTGCGGGAGGGGGGTGTAGGCGAGATTGGCTTTTCCTTCCTCAATCTGTTGGAC
CYC1 terminator
5,580   5,600   5,620   5,640   5,660

AAGTCTAGGTCCCTATTTATTTTTTTATAGTTATGTTAGTATTAAGAACGTTATTTATATTTCAAATTTTTCTTTTTTTTCTGTACAGACGCGTGTACGCATGTAAC
TTCAGATCCAGGGATAAATAAAAAAATATCAATACAATCATAATTCTTGCAATAAATATAAAGTTTAAAAAGAAAAAAAAGACATGTCTGCGCACATGCGTACATTG
CYC1 terminator
5,680   5,700   5,720   5,740   5,760

ATTATACTGAAAACCTTGCTTGAGAAGGTTTTGGGACGCTCGAAGGCTTTAATTTGCGCGCGCTCACTGGCCGTCGTTTTACAACGTCGTGACTGGGAAAACCCTGG
TAATATGACTTTTGGAACGAACTCTTCCAAAACCCTGCGAGCTTCCGAAATTAAACGCGCGCGAGTGACCGGCAGCAAAATGTTGCAGCACTGACCCTTTTGGGACC
CYC1 terminator                    M13-fwd
5,780   5,800   5,820   5,840   5,860   5,880

CGTTACCCAACTTAATCGCCTTGCAGCACATCCCCCTTTCGCCAGCTGGCGTAATAGCGAAGAGGCCCGCACCGATCGCCCTTCCCAACAGTTGCGCAGCCTGAATG
GCAATGGGTTGAATTAGCGGAACGTCGTGTAGGGGGAAAGCGGTCGACCGCATTATCGCTTCTCCGGGCGTGGCTAGCGGGAAGGGTTGTCAACGCGTCGGACTTAC
LacZ alpha
5,900   5,920   5,940   5,960   5,980

GCGAATGGACGCGCCCTGTAGCGGCGCATTAAGCGCGGCGGGTGTGGTGGTTACGCGCAGCGTGACCACTACACTTGCCAGCGCCCTAGCGCCCGCTCCTTTCGCTT
CGCTTACCTGCGCGGGACATCGCCGCGTAATTCGCGCCGCCCACACCACCAATGCGCGTCGCACTGGTGATGTGAACGGTCGCGGGATCGCGGGCGAGGAAAGCGAA
6,000   6,020   6,040   6,060   6,080

TCTTCCCTTCCTTTCTCGCCACGTTCGCCGGCTTTCCCCGTCAAGCTCTAAATCGGGGGCTCCCTTTAGGGTTCCGATTTAGTGCTTTACGGCACCTCGACCCCAAA
AGAAGGGAAGGAAAGAGCGGTGCAAGCGGCCGAAAGGGGCAGTTCGAGATTTAGCCCCCGAGGGAAATCCCAAGGCTAAATCACGAAATGCCGTGGAGCTGGGGTTT
6,100   6,120   6,140   6,160   6,180   6,200

AAACTTGATTAGGGTGATGGTTCACGTAGTGGGCCATCGCCCTGATAGACGGTTTTTCGCCCTTTGACGTTGGAGTCCACGTTCTTTAATAGTGGACTCTTGTTCCA
TTTGAACTAATCCCACTACCAAGTGCATCACCCGGTAGCGGGACTATCTGCCAAAAAGCGGGAAACTGCAACCTCAGGTGCAAGAAATTATCACCTGAGAACAAGGT
6,220   6,240   6,260   6,280   6,300

AACTGGAACAACACTCAACCCTATCTCGGTCTATTCTTTTGATTTATAAGGGATTTTGCCGATTTCGGCCTATTGGTTAAAAAATGAGCTGATTTAACAAAAATTTA
TTGACCTTGTTGTGAGTTGGGATAGAGCCAGATAAGAAAACTAAATATTCCCTAAAACGGCTAAAGCCGGATAACCAATTTTTTACTCGACTAAATTGTTTTTAAAT
6,320   6,340   6,360   6,380   6,400   6,420
```

ACGCGAATTTTAACAAAATATTAACGCTTACAATTTCCTGATGCGGTATTTTCTCCTTACGCATCTGTGCGGTATTTCACACCGCATAGGGTAATAACTGATATAAT
TGCGCTTAAAATTGTTTTATAATTGCGAATGTTAAAGGACTACGCCATAAAAGAGGAATGCGTAGACACGCCATAAAGTGTGGCGTATCCCATTATTGACTATATTA

6,440 6,460 6,480 6,500 6,520

TAAATTGAAGCTCTAATTTGTGAGTTTAGTATACATGCATTTACTTATAATACAGTTTTTTAAGCAAGGATTTTCTTAACTTCTTCGGCGACAGCATCACCGACTTC
ATTTAACTTCGAGATTAAACACTCAAATCATATGTACGTAAATGAATATTATGTCAAAAAATTCGTTCCTAAAAGAATTGAAGAAGCCGCTGTCGTAGTGGCTGAAG

LEU2

6,540 6,560 6,580 6,600 6,620

GGTGGTACTGTTGGAACCACCTAAATCACCAGTTCTGATACCTGCATCCAAAACCTTTTTAACTGCATCTTCAATGGCCTTACCTTCTTCAGGCAAGTTCAATGACA
CCACCATGACAACCTTGGTGGATTTAGTGGTCAAGACTATGGACGTAGGTTTTGGAAAAATTGACGTAGAAGTTACCGGAATGGAAGAAGTCCGTTCAAGTTACTGT

LEU2

6,640 6,660 6,680 6,700 6,720 6,740

ATTTCAACATCATTGCAGCAGACAAGATAGTGGCGATAGGGTTGACCTTATTCTTTGGCAAATCTGGAGCAGAACCGTGGCATGGTTCGTACAAACCAAATGCGGTG
TAAAGTTGTAGTAACGTCGTCTGTTCTATCACCGCTATCCCAACTGGAATAAGAAACCGTTTAGACCTCGTCTTGGCACCGTACCAAGCATGTTTGGTTTACGCCAC

LEU2

6,760 6,780 6,800 6,820 6,840

TTCTTGTCTGGCAAAGAGGCCAAGGACGCAGATGGCAACAAACCCAAGGAACCTGGGATAACGGAGGCTTCATCGGAGATGATATCACCAAACATGTTGCTGGTGAT
AAGAACAGACCGTTTCTCCGGTTCCTGCGTCTACCGTTGTTTGGGTTCCTTGGACCCTATTGCCTCCGAAGTAGCCTCTACTATAGTGGTTTGTACAACGACCACTA

LEU2

6,860 6,880 6,900 6,920 6,940

TATAATACCATTTAGGTGGGTTGGGTTCTTAACTAGGATCATGGCGGCAGAATCAATCAATTGATGTTGAACCTTCAATGTAGGGAATTCGTTCTTGATGGTTTCCT
ATATTATGGTAAATCCACCCAACCCAAGAATTGATCCTAGTACCGCCGTCTTAGTTAGTTAACTACAACTTGGAAGTTACATCCCTTAAGCAAGAACTACCAAAGGA

LEU2

6,960 6,980 7,000 7,020 7,040 7,060

CCACAGTTTTTCTCCATAATCTTGAAGAGGCCAAAACATTAGCTTTATCCAAGGACCAAATAGGCAAT
GGTGTCAAAAAGAGGTATTAGAACTTCTCCGGTTTTGTAATCGAAATAGGTTCCTGGTTTATCCGTTA

LEU2

7,070 7,080 7,090 7,100 7,110 7,120 7,130

ST/SC ligation / pNb_Syn Nb_e-FLAG (pRS415) (70...

GGTGGCTCATGTTGTAGGGCCATGAAAGCGGCCATTCTTGTGATTCTTTGCACTTCTGGAACGGTGTATTGTTCACTATCCCAAGCGACACCATCACCATCGTCTTC
CCACCGAGTACAACATCCCGGTACTTTCGCCGGTAAGAACACTAAGAAACGTGAAGACCTTGCCACATAACAAGTGATAGGGTTCGCTGTGGTAGTGGTAGCAGAAG
LEU2
20 40 60 80 100

CTTTCTCTTACCAAAGTAAATACCTCCCACTAATTCTCTGACAACAACGAAGTCAGTACCTTTAGCAAATTGTGGCTTGATTGGAGATAAGTCTAAAAGAGAGTCGG
GAAAGAGAATGGTTTCATTTATGGAGGGTGATTAAGAGACTGTTGTTGCTTCAGTCATGGAAATCGTTTAACACCGAACTAACCTCTATTCAGATTTTCTCTCAGCC
LEU2
120 140 160 180 200

ATGCAAAGTTACATGGTCTTAAGTTGGCGTACAATTGAAGTTCTTTACGGATTTTTAGTAAACCTTGTTCAGGTCTAACACTACCGGTACCCCATTTAGGACCACCC
TACGTTTCAATGTACCAGAATTCAACCGCATGTTAACTTCAAGAAATGCCTAAAAATCATTTGGAACAAGTCCAGATTGTGATGGCCATGGGGTAAATCCTGGTGGG
LEU2
220 240 260 280 300 320

ACAGCACCTAACAAAACGGCATCAGCCTTCTTGGAGGCTTCCAGCGCCTCATCTGGAAGTGGAACACCTGTAGCATCGATAGCAGCACCACCAATTAAATGATTTTC
TGTCGTGGATTGTTTTGCCGTAGTCGGAAGAACCTCCGAAGGTCGCGGAGTAGACCTTCACCTTGTGGACATCGTAGCTATCGTCGTGGTGGTTAATTTACTAAAAG
LEU2
340 360 380 400 420

GAAATCGAACTTGACATTGGAACGAACATCAGAAATAGCTTTAAGAACCTTAATGGCTTCGGCTGTGATTTCTTGACCAACGTGGTCACCTGGCAAAACGACGATCT
CTTTAGCTTGAACTGTAACCTTGCTTGTAGTCTTTATCGAAATTCTTGGAATTACCGAAGCCGACACTAAAGAACTGGTTGCACCAGTGGACCGTTTTGCTGCTAGA
LEU2
440 460 480 500 520

TCTTAGGGGCAGACATTAGAATGGTATATCCTTGAAATATATATATATATATATNTNGCTGAAATGTAAAAGGTAAGAAAAGTTAGAAAGTAAGACGATTGCTAACCAC
AGAATCCCCGTCTGTAATCTTACCATATAGGAACTTTATATATATATATATATANANCGACTTTACATTTTCCATTCTTTTCAATCTTTCATTCTGCTAACGATTGGTG
LEU2
540 560 580 600 620 640

CTATTGGAAAAAACAATAGGTCCTTAAATAATATTGTCAACTTCAAGTATTGTGATGCAAGCATTTAGTCATGAACGCTTCTCTATTCTATATGAAAAGCCGGTTCC
GATAACCTTTTTTGTTATCCAGGAATTTATTATAACAGTTGAAGTTCATAACACTACGTTCGTAAATCAGTACTTGCGAAGAGATAAGATATACTTTTCGGCCAAGG
660 680 700 720 740

GGCGCTCTCACCTTTCCTTTTTCTCCCAATTTTTCAGTTGAAAAAGGTATATGCGTCAGGCGACCTCTGAAATTAACAAAAAATTTCCAGTCATCGAATTTGATTCT
CCGCGAGAGTGGAAAGGAAAAAGAGGGTTAAAAAGTCAACTTTTTCCATATACGCAGTCCGCTGGAGACTTTAATTGTTTTTTAAAGGTCAGTAGCTTAAACTAAGA
760 780 800 820 840

GTGCGATAGCGCCCCTGTGTGTTCTCGTTATGTTGAGGAAAAAAATAATGGTTGCTAAGAGATTCGAACTCTTGCATCTTACGATACCTGAGTATTCCCACAGTTTG
CACGCTATCGCGGGGACACACAAGAGCAATACAACTCCTTTTTTTATTACCAACGATTCTCTAAGCTTGAGAACGTAGAATGCTATGGACTCATAAGGGTGTCAAAC
860 880 900 920 940 960

AAAAGCTGTGGTATGGTGCACTCTCAGTACAATCTGCTCTGATGCCGCATAGTTAAGCCAGCCCCGACACCCGCCAACACCCGCTGACGCGCCCTGACGGGCTTGTC
TTTTCGACACCATACCACGTGAGAGTCATGTTAGACGAGACTACGGCGTATCAATTCGGTCGGGGCTGTGGGCGGTTGTGGGCGACTGCGCGGGACTGCCCGAACAG

980 1,000 1,020 1,040 1,060

TGCTCCCGGCATCCGCTTACAGACAAGCTGTGACCGTCTCCGGGAGCTGCATGTGTCAGAGGTTTTCACCGTCATCACCGAAACGCGCGAGACGAAAGGGCCTCGTG
ACGAGGGCCGTAGGCGAATGTCTGTTCGACACTGGCAGAGGCCCTCGACGTACACAGTCTCCAAAAGTGGCAGTAGTGGCTTTGCGCGCTCTGCTTTCCCGGAGCAC

1,080 1,100 1,120 1,140 1,160

ATACGCCTATTTTTATAGGTTAATGTCATGATAATAATGGTTTCTTAGTAgatcgcttgcctgtaacttacacgcgcctcgtatcttttaatgatggaataatttgg
TATGCGGATAAAAATATCCAATTACAGTACTATTATTACCAAAGAATCATctagcgaacggacattgaatgtgcgcggagcatagaaaattactaccttattaaacc

CEN/ARS

1,180 1,200 1,220 1,240 1,260 1,280

gaatttactctgtgtttatttatttttatgttttgtatttggattttagaaagtaaataaagaaggtagaagagttacggaatgaagaaaaaaaaataaacaaaggt
cttaaatgagacacaaataaataaaaatacaaaacataaacctaaaatctttcatttatttcttccatcttctcaatgccttacttctttttttttatttgtttcca

CEN/ARS

1,300 1,320 1,340 1,360 1,380

ttaaaaaatttcaacaaaaagcgtactttacatatatatttattagacaagaaaagcagattaaatagatatacattcgattaacgataagtaaaatgtaaaatcac
aatttttaaagttgtttttcgcatgaaatgtatatataaataatctgttcttttcgtctaatttatctatatgtaagctaattgctattcattttacattttagtg

CEN/ARS

1,400 1,420 1,440 1,460 1,480

aggattttcgtgtgtggtcttctacacagacaagatgaaacaattcggcattaatacctgagagcaggaagagcaagataaaaggtagtatttgttggcgatccccc
tcctaaaagcacacaccagaagatgtgtctgttctactttgttaagccgtaattatggactctcgtccttctcgttctattttccatcataaacaaccgctagggggg

CEN/ARS

1,500 1,520 1,540 1,560 1,580 1,600

tagagtcttttacatcttcggaaaacaaaaactatttttttctttaatttctttttttactttctatttttaatttatatatttatattaaaaaatttaaattataat
atctcagaaaatgtagaagccttttgtttttgataaaaaagaaattaaagaaaaaaatgaaagataaaaattaaatatataaatataatttttttaaatttaatatta

CEN/ARS

1,620 1,640 1,660 1,680 1,700

tatttttatagcacgtgatGTTCAGGTGGCACTTTTCGGGGAAATGTGCGCGGAACCCCTATTTGTTTATTTTTCTAAATACATTCAAATATGTATCCGCTCATGAG
ataaaaatatcgtgcactaCAAGTCCACCGTGAAAAGCCCCTTTACACGCGCCTTGGGGATAAACAAATAAAAAGATTTATGTAAGTTTATACATAGGCGAGTACTC

CEN/ARS

1,720 1,740 1,760 1,780 1,800

ACAATAACCCTGATAAATGCTTCAATAATATTGAAAAAGGAAGAGTATGAGTATTCAACATTTCCGTGTCGCCCTTATTCCCTTTTTTGCGGCATTTTGCCTTCCTG
TGTTATTGGGACTATTTACGAAGTTATTATAACTTTTTCCTTCTCATACTCATAAGTTGTAAAGGCACAGCGGGAATAAGGGAAAAAACGCCGTAAAACGGAAGGAC

1,820 1,840 1,860 1,880 1,900 1,920

TTTTTGCTCACCCAGAAACGCTGGTGAAAGTAAAAGATGCTGAAGATCAGTTGGGTGCACGAGTGGGTTACATCGAACTGGATCTCAACAGCGGTAAGATCCTTGAG
AAAAACGAGTGGGTCTTTGCGACCACTTTCATTTTCTACGACTTCTAGTCAACCCACGTGCTCACCCAATGTAGCTTGACCTAGAGTTGTCGCCATTCTAGGAACTC

1,940 1,960 1,980 2,000 2,020

AGTTTTCGCCCCGAAGAACGTTTTCCAATGATGAGCACTTTTAAAGTTCTGCTATGTGGCGCGGTATTATCCCGTATTGACGCCGGGCAAGAGCAACTCGGTCGCCG
TCAAAAGCGGGGCTTCTTGCAAAAGGTTACTACTCGTGAAAATTTCAAGACGATACACCGCGCCATAATAGGGCATAACTGCGGCCCGTTCTCGTTGAGCCAGCGGC

AmpR

2,040 2,060 2,080 2,100 2,120 2,140

CATACACTATTCTCAGAATGACTTGGTTGAGTACTCACCAGTCACAGAAAAGCATCTTACGGATGGCATGACAGTAAGAGAATTATGCAGTGCTGCCATAACCATGA
GTATGTGATAAGAGTCTTACTGAACCAACTCATGAGTGGTCAGTGTCTTTTCGTAGAATGCCTACCGTACTGTCATTCTCTTAATACGTCACGACGGTATTGGTACT

AmpR

2,160 2,180 2,200 2,220 2,240

GTGATAACACTGCGGCCAACTTACTTCTGACAACGATCGGAGGACCGAAGGAGCTAACCGCTTTTTTGCACAACATGGGGGATCATGTAACTCGCCTTGATCGTTGG
CACTATTGTGACGCCGGTTGAATGAAGACTGTTGCTAGCCTCCTGGCTTCCTCGATTGGCGAAAAAACGTGTTGTACCCCCTAGTACATTGAGCGGAACTAGCAACC

AmpR

2,260 2,280 2,300 2,320 2,340

GAACCGGAGCTGAATGAAGCCATACCAAACGACGAGCGTGACACCACGATGCCTGTAGCAATGGCAACAACGTTGCGCAAACTATTAACTGGCGAACTACTTACTCT
CTTGGCCTCGACTTACTTCGGTATGGTTTGCTGCTCGCACTGTGGTGCTACGGACATCGTTACCGTTGTTGCAACGCGTTTGATAATTGACCGCTTGATGAATGAGA

AmpR

2,360 2,380 2,400 2,420 2,440 2,460

AGCTTCCCGGCAACAATTAATAGACTGGATGGAGGCGGATAAAGTTGCAGGACCACTTCTGCGCTCGGCCCTTCCGGCTGGCTGGTTTATTGCTGATAAATCTGGAG
TCGAAGGGCCGTTGTTAATTATCTGACCTACCTCCGCCTATTTCAACGTCCTGGTGAAGACGCGAGCCGGGAAGGCCGACCGACCAAATAACGACTATTTAGACCTC

AmpR

2,480 2,500 2,520 2,540 2,560

CCGGTGAGCGTGGGTCTCGCGGTATCATTGCAGCACTGGGGCCAGATGGTAAGCCCTCCCGTATCGTAGTTATCTACACGACGGGGAGTCAGGCAACTATGGATGAA
GGCCACTCGCACCCAGAGCGCCATAGTAACGTCGTGACCCCGGTCTACCATTCGGGAGGGCATAGCATCAATAGATGTGCTGCCCCTCAGTCCGTTGATACCTACTT

AmpR

2,580 2,600 2,620 2,640 2,660

CGAAATAGACAGATCGCTGAGATAGGTGCCTCACTGATTAAGCATTGGTAACTGTCAGACCAAGTTTACTCATATATACTTTAGATTGATTTAAAACTTCATTTTTA
GCTTTATCTGTCTAGCGACTCTATCCACGGAGTGACTAATTCGTAACCATTGACAGTCTGGTTCAAATGAGTATATATGAAATCTAACTAAATTTTGAAGTAAAAAT

AmpR

2,680 2,700 2,720 2,740 2,760 2,780

ATTTAAAAGGATCTAGGTGAAGATCCTTTTTGATAATCTCATGACCAAAATCCCTTAACGTGAGTTTTCGTTCCACTGAGCGTCAGACCCCGTAGAAAAGATCAAAG
TAAATTTTCCTAGATCCACTTCTAGGAAAAACTATTAGAGTACTGGTTTTAGGGAATTGCACTCAAAAGCAAGGTGACTCGCAGTCTGGGGCATCTTTTCTAGTTTC

2,800 2,820 2,840 2,860 2,880

GATCTTCTTGAGATCCTTTTTTTCTGCGCGTAATCTGCTGCTTGCAAACAAAAAAACCACCGCTACCAGCGGTGGTTTGTTTGCCGGATCAAGAGCTACCAACTCTT
CTAGAAGAACTCTAGGAAAAAAAGACGCGCATTAGACGACGAACGTTTGTTTTTTTGGTGGCGATGGTCGCCACCAAACAAACGGCCTAGTTCTCGATGGTTGAGAA

2,900 2,920 2,940 2,960 2,980

TTTCCGAAGGTAACTGGCTTCAGCAGAGCGCAGATACCAAATACTGTTCTTCTAGTGTAGCCGTAGTTAGGCCACCACTTCAAGAACTCTGTAGCACCGCCTACATA
AAAGGCTTCCATTGACCGAAGTCGTCTCGCGTCTATGGTTTATGACAAGAAGATCACATCGGCATCAATCCGGTGGTGAAGTTCTTGAGACATCGTGGCGGATGTAT

3,000 3,020 3,040 3,060 3,080 3,100

CCTCGCTCTGCTAATCCTGTTACCAGTGGCTGCTGCCAGTGGCGATAAGTCGTGTCTTACCGGGTTGGACTCAAGACGATAGTTACCGGATAAGGCGCAGCGGTCGG
GGAGCGAGACGATTAGGACAATGGTCACCGACGACGGTCACCGCTATTCAGCACAGAATGGCCCAACCTGAGTTCTGCTATCAATGGCCTATTCCGCGTCGCCAGCC

3,120 3,140 3,160 3,180 3,200

GCTGAACGGGGGGTTCGTGCACACAGCCCAGCTTGGAGCGAACGACCTACACCGAACTGAGATACCTACAGCGTGAGCTATGAGAAAGCGCCACGCTTCCCGAAGGG
CGACTTGCCCCCCAAGCACGTGTGTCGGGTCGAACCTCGCTTGCTGGATGTGGCTTGACTCTATGGATGTCGCACTCGATACTCTTTCGCGGTGCGAAGGGCTTCCC

3,220 3,240 3,260 3,280 3,300

AGAAAGGCGGACAGGTATCCGGTAAGCGGCAGGGTCGGAACAGGAGAGCGCACGAGGGAGCTTCCAGGGGGAAACGCCTGGTATCTTTATAGTCCTGTCGGGTTTCG
TCTTTCCGCCTGTCCATAGGCCATTCGCCGTCCCAGCCTTGTCCTCTCGCGTGCTCCCTCGAAGGTCCCCCTTTGCGGACCATAGAAATATCAGGACAGCCCAAAGC

3,320 3,340 3,360 3,380 3,400 3,420

CCACCTCTGACTTGAGCGTCGATTTTTGTGATGCTCGTCAGGGGGGCGGAGCCTATGGAAAAACGCCAGCAACGCGGCCTTTTTACGGTTCCTGGCCTTTTGCTGGC
GGTGGAGACTGAACTCGCAGCTAAAAACACTACGAGCAGTCCCCCCGCCTCGGATACCTTTTTGCGGTCGTTGCGCCGGAAAAATGCCAAGGACCGGAAAACGACCG

3,440 3,460 3,480 3,500 3,520

CTTTTGCTCACATGTTCTTTCCTGCGTTATCCCCTGATTCTGTGGATAACCGTATTACCGCCTTTGAGTGAGCTGATACCGCTCGCCGCAGCCGAACGACCGAGCGC
GAAAACGAGTGTACAAGAAAGGACGCAATAGGGGACTAAGACACCTATTGGCATAATGGCGGAAACTCACTCGACTATGGCGAGCGGCGTCGGCTTGCTGGCTCGCG

3,540 3,560 3,580 3,600 3,620

AGCGAGTCAGTGAGCGAGGAAGCGGAAGAGCGCCCAATACGCAAACCGCCTCTCCCCGCGCGTTGGCCGATTCATTAATGCAGCTGGCACGACAGGTTTCCCGACTG
TCGCTCAGTCACTCGCTCCTTCGCCTTCTCGCGGGTTATGCGTTTGGCGGAGAGGGGCGCGCAACCGGCTAAGTAATTACGTCGACCGTGCTGTCCAAAGGGCTGAC

3,640 3,660 3,680 3,700 3,720 3,740

GAAAGCGGCCAGTGAGCGCGCGCAAATTAAAGCCTTCGAGCGTCCCAAAACGCAAACCGCCTCTCCCCGCGCGTTGGCCGATTCATTAATGCAGCTGGCACGACAGG
CTTTCGCCGGTCACTCGCGCGCGTTTAATTTCGGAAGCTCGCAGGGTTTTGCGTTTGGCGGAGAGGGGCGCGCAACCGGCTAAGTAATTACGTCGACCGTGCTGTCC

3,760 3,780 3,800 3,820 3,840

TTTCCCGACTGGAAAGCGGGCAGTGAGCGCAACGCAATTAATGTGAGTTAGCTCACTCATTAGGCACCCCAGGCTTTACACTTTATGCTTCCGGCTCGTATGTTGTG
AAAGGGCTGACCTTTCGCCCGTCACTCGCGTTGCGTTAATTACACTCAATCGAGTGAGTAATCCGTGGGGTCCGAAATGTGAAATACGAAGGCCGAGCATACAACAC

lac promoter

3,860 3,880 3,900 3,920 3,940

TGGAATTGTGAGCGGATAACAATTTCACACAGGAAACAGCTATGACCATGATTACGCCAAGCGCGCAATTAACCCTCACTAAAGGGAACAAAAGCTGGAGCTcAGTT
ACCTTAACACTCGCCTATTGTTAAAGTGTGTCCTTTGTCGATACTGGTACTAATGCGGTTCGCGCGTTAATTGGGAGTGATTTCCCTTGTTTTCGACCTCGAgTCAA

T3
LacO M13-rev T3 promoter

3,960 3,980 4,000 4,020 4,040 4,060

TATCATTATCAATACTcGCCATTTCAAAGAATACGTAAATAATTAATAGTAGTGATTTTCCTAACTTTATTTAGTCAAAAAATTAGCCTTTTAATTCTGCTGTAACC
ATAGTAATAGTTATGAgCGGTAAAGTTTCTTATGCATTTATTAATTATCATCACTAAAAGGATTGAAATAAATCAGTTTTTTAATCGGAAAATTAAGACGACATTGG

GPD promoter

4,080 4,100 4,120 4,140 4,160

CGTACATGCCCAAAATAGGGGGCGGGTTACACAGAATATATAACATCGTAGGTGTCTGGGTGAACAGTTTATTCCTGGCATCCACTAAATATAATGGAGCCCGCTTT
GCATGTACGGGTTTTATCCCCCGCCCAATGTGTCTTATATATTGTAGCATCCACAGACCCACTTGTCAAATAAGGACCGTAGGTGATTTATATTACCTCGGGCGAAA

GPD promoter

4,180 4,200 4,220 4,240 4,260 4,280

TTAAGCTGGCATCCAGAAAAAAAAAGAATCCCAGCACCAAAATATTGTTTTCTTCACCAACCATCAGTTCATAGGTCCATTCTCTTAGCGCAACTACAGAGAACAGG
AATTCGACCGTAGGTCTTTTTTTTTCTTAGGGTCGTGGTTTTATAACAAAAGAAGTGGTTGGTAGTCAAGTATCCAGGTAAGAGAATCGCGTTGATGTCTCTTGTCC

GPD promoter

4,300 4,320 4,340 4,360 4,380

GGCACAAACAGGCAAAAAACGGGCACAACCTCAATGGAGTGATGCAACCTGCCTGGAGTAAATGATGACACAAGGCAATTGACCCACGCATGTATCTATCTCATTTT
CCGTGTTTGTCCGTTTTTTGCCCGTGTTGGAGTTACCTCACTACGTTGGACGGACCTCATTTACTACTGTGTTCCGTTAACTGGGTGCGTACATAGATAGAGTAAAA

GPD promoter

4,400 4,420 4,440 4,460 4,480

CTTACACCTTCTATTACCTTCTGCTCTCTCTGATTTGGAAAAAGCTGAAAAAAAAGGTTGAAACCAGTTCCCTGAAATTATTCCCCTACTTGACTAATAAGTATATA
GAATGTGGAAGATAATGGAAGACGAGAGAGACTAAACCTTTTTCGACTTTTTTTTCCAACTTTGGTCAAGGGACTTTAATAAGGGGATGAACTGATTATTCATATAT

GPD promoter

4,500 4,520 4,540 4,560 4,580 4,600

AAGACGGTAGGTATTGATTGTAATTCTGTAAATCTATTTCTTAAACTTCTTAAATTCTACTTTTATAGTTAGTCTTTTTTTTAGTTTTAAAACACCAGAACTTAGTT
TTCTGCCATCCATAACTAACATTAAGACATTTAGATAAAGAATTTGAAGAATTTAAGATGAAAATATCAATCAGAAAAAAAATCAAAATTTTGTGGTCTTGAATCAA

GPD promoter

4,620 4,640 4,660 4,680 4,700

TCGACGGATTCTAGAACTAGTGGATCCATGAGATTCCCATCTATCTTCACCGCTGTTTTGTTCGCTGCTTCTTCTGCTTTGGCTGCTCCAGCTAACACCACCACCGA
AGCTGCCTAAGATCTTGATCACCTAGGTACTCTAAGGGTAGATAGAAGTGGCGACAAAACAAGCGACGAAGAAGACGAAACCGACGAGGTCGATTGTGGTGGTGGCT

1 2 3 4 5 6 7 8 9 10 11 12 13 14 15 16 17 18 19 20 21 22 23 24 25 26 27
M R F P S I F T A V L F A A S S A L A A P A N T T T E

GPD promoter Mating factor alpha leader

4,720 4,740 4,760 4,780 4,800

```
AGACGAAACCGCTCAAATCCCAGCTGAAGCTGTTATCGACTACTCTGACTTGGAAGGTGACTTCGACGCTGCTGCTTTGCCATTGTCTAACTCTACCAACAACGGTT
TCTGCTTTGGCGAGTTTAGGGTCGACTTCGACAATAGCTGATGAGACTGAACCTTCCACTGAAGCTGCGACGACGAAACGGTAACAGATTGAGATGGTTGTTGCCAA
 28 29 30 31 32 33 34 35 36 37 38 39 40 41 42 43 44 45 46 47 48 49 50 51 52 53 54 55 56 57 58 59 60 61 62
 D  E  T  A  Q  I  P  A  E  A  V  I  D  Y  S  D  L  E  G  D  F  D  A  A  A  L  P  L  S  N  S  T  N  N  G
Mating factor alpha leader
4,820  4,840  4,860  4,880  4,900  4,920

TGTCTTCTACCAACACCACCATCGCTTCTATCGCTGCTAAGGAAGAAGGTGTTCAATTGGACAAGAGAGAAagatctggcggccgcgcatgcgctagcctcgagCAA
ACAGAAGATGGTTGTGGTGGTAGCGAAGATAGCGACGATTCCTTCTTCCACAAGTTAACCTGTTCTCTCTTtctagaccgccggcgcgtacgcgatcggagctcGTT
63 64 65 66 67 68 69 70 71 72 73 74 75 76 77 78 79 80 81 82 83 84 85 86
 L  S  S  T  N  T  T  I  A  S  I  A  A  K  E  E  G  V  Q  L  D  K  R  E                                  Q
Mating factor alpha leader
4,940  4,960  4,980  5,000  5,020

GTCCAGCTGGTCGAGTCAGGTGGTGGATCTGTCCAAGCCGGGGGGTCTCTAAGACTATCATGTACGGCGTCAGGCTATATTTTTGACTTCTCTTACATGTGGTTCAG
CAGGTCGACCAGCTCAGTCCACCACCTAGACAGGTTCGGCCCCCCAGAGATTCTGATAGTACATGCCGCAGTCCGATATAAAAACTGAAGAGAATGTACACCAAGTC
 2     4     6     8     10    12    14    16    18    20    22    24    26    28    30    32    34    36
 V  Q  L  V  E  S  G  G  G  S  V  Q  A  G  G  S  L  R  L  S  C  T  A  S  G  Y  I  F  D  F  S  Y  M  W  F  R
Syn Nb_e
5,040  5,060  5,080  5,100  5,120

GCAAGCACCGGGCCAGGAAAGAGAGGCAGTTGCGGAACTTGTTGCCGGTATTGATGAAGGTACTACTACCAATTATCGTTTTACTATTAGCAGAGACAACGCAAAAA
CGTTCGTGGCCCGGTCCTTTCTCTCCGTCAACGCCTTGAACAACGGCCATAACTACTTCCATGATGATGGTTAATAGCAAAATGATAATCGTCTCTGTTGCGTTTTT
 38    40    42    44    46    48    50    52    54    56    58    60    62    64    66    68    70    72
 Q  A  P  G  Q  E  R  E  A  V  A  E  L  V  A  G  I  D  E  G  T  T  T  N  Y  R  F  T  I  S  R  D  N  A  K
Syn Nb_e
5,140  5,160  5,180  5,200  5,220  5,240

ATACTGTTACGCTACAAATGAATAATCTGAAGCCAGAAGATACTGCGATCTATTATTGTGCCGCAGCGGTTCCGGACTACTCTTCTCCGCCGCTGCATTACTATTGG
TATGACAATGCGATGTTTACTTATTAGACTTCGGTCTTCTATGACGCTAGATAATAACACGGCGTCGCCAAGGCCTGATGAGAAGAGGCGGCGACGTAATGATAACC
   74    76    78    80    82    84    86    88    90    92    94    96    98    100   102   104   106   108
 N  T  V  T  L  Q  M  N  N  L  K  P  E  D  T  A  I  Y  Y  C  A  A  A  V  P  D  Y  S  S  P  P  L  H  Y  Y  W
Syn Nb_e
5,260  5,280  5,300  5,320  5,340

GGACAAGGAACACAGGTCACAGTATCATCTGGCGGAGGAGGTTCCGGTGGAGGGGGGTCAGACTATAAGGATGACGACGATAAGGCTGGAGGTTCGAGGGGAGTCCC
CCTGTTCCTTGTGTCCAGTGTCATAGTAGACCGCCTCCTCCAAGGCCACCTCCCCCCAGTCTGATATTCCTACTGCTGCTATTCCGACCTCCAAGCTCCCCTCAGGG
   110   112   114   116   118 1  2  3  4  5  6  7  8  9  10 1  2  3  4  5  6  7  8  1  2  3  4  1  2  3  4
 G  Q  G  T  Q  V  T  V  S  S  G  G  G  G  S  G  G  G  G  S  D  Y  K  D  D  D  D  K  A  G  G  S  R  G  V  P
Syn Nb_e                                  FLAG tag                          SpyTag
5,360  5,380  5,400  5,420  5,440
```

TCACATAGTGATGGTGGATGCTTACAAAAGGTATAAATAACCCGGGCTGCAGGAATTCGATATCAAGCTTATCGATACCGTCGACCTCGAGTCATGTAATTAGTTAT
AGTGTATCACTACCACCTACGAATGTTTTCCATATTTATTGGGCCCGACGTCCTTAAGCTATAGTTCGAATAGCTATGGCAGCTGGAGCTCAGTACATTAATCAATA
5 6 7 8 9 10 11 12 13 14 15 16 1
H I V M V D A Y K R Y K *
SpyTag CYC1...ator
5,460 5,480 5,500 5,520 5,540 5,560

GTCACGCTTACGTTCACGCCCTCCCCCCACATCCGCTCTAACCGAAAAGGAAGGAGTTAGACAACCTGAAGTCTAGGTCCCTATTTATTTTTTTATAGTTATGTTAG
CAGTGCGAATGCAAGTGCGGGAGGGGGGTGTAGGCGAGATTGGCTTTTCCTTCCTCAATCTGTTGGACTTCAGATCCAGGGATAAATAAAAAAATATCAATACAATC
CYC1 terminator
5,580 5,600 5,620 5,640 5,660

TATTAAGAACGTTATTTATATTTCAAATTTTTCTTTTTTTTCTGTACAGACGCGTGTACGCATGTAACATTATACTGAAAACCTTGCTTGAGAAGGTTTTGGGACGC
ATAATTCTTGCAATAAATATAAAGTTTAAAAAGAAAAAAAAGACATGTCTGCGCACATGCGTACATTGTAATATGACTTTTGGAACGAACTCTTCCAAAACCCTGCG
CYC1 terminator
5,680 5,700 5,720 5,740 5,760

TCGAAGGCTTTAATTTGCGCGCGCTCACTGGCCGTCGTTTTACAACGTCGTGACTGGGAAAACCCTGGCGTTACCCAACTTAATCGCCTTGCAGCACATCCCCCTTT
AGCTTCCGAAATTAAACGCGCGCGAGTGACCGGCAGCAAAATGTTGCAGCACTGACCCTTTTGGGACCGCAATGGGTTGAATTAGCGGAACGTCGTGTAGGGGGAAA
CYC1 ...nator M13-fwd
5,780 5,800 5,820 5,840 5,860 5,880

CGCCAGCTGGCGTAATAGCGAAGAGGCCCGCACCGATCGCCCTTCCCAACAGTTGCGCAGCCTGAATGGCGAATGGACGCGCCCTGTAGCGGCGCATTAAGCGCGGC
GCGGTCGACCGCATTATCGCTTCTCCGGGCGTGGCTAGCGGGAAGGGTTGTCAACGCGTCGGACTTACCGCTTACCTGCGCGGGACATCGCCGCGTAATTCGCGCCG
LacZ alpha
5,900 5,920 5,940 5,960 5,980

GGGTGTGGTGGTTACGCGCAGCGTGACCACTACACTTGCCAGCGCCCTAGCGCCCGCTCCTTTCGCTTTCTTCCCTTCCTTTCTCGCCACGTTCGCCGGCTTTCCCC
CCCACACCACCAATGCGCGTCGCACTGGTGATGTGAACGGTCGCGGGATCGCGGGCGAGGAAAGCGAAAGAAGGGAAGGAAAGAGCGGTGCAAGCGGCCGAAAGGGG
6,000 6,020 6,040 6,060 6,080

GTCAAGCTCTAAATCGGGGGCTCCCTTTAGGGTTCCGATTTAGTGCTTTACGGCACCTCGACCCCAAAAAACTTGATTAGGGTGATGGTTCACGTAGTGGGCCATCG
CAGTTCGAGATTTAGCCCCCGAGGGAAATCCCAAGGCTAAATCACGAAATGCCGTGGAGCTGGGGTTTTTTGAACTAATCCCACTACCAAGTGCATCACCCGGTAGC
6,100 6,120 6,140 6,160 6,180 6,200

CCCTGATAGACGGTTTTTCGCCCTTTGACGTTGGAGTCCACGTTCTTTAATAGTGGACTCTTGTTCCAAACTGGAACAACACTCAACCCTATCTCGGTCTATTCTTT
GGGACTATCTGCCAAAAAGCGGGAAACTGCAACCTCAGGTGCAAGAAATTATCACCTGAGAACAAGGTTTGACCTTGTTGTGAGTTGGGATAGAGCCAGATAAGAAA
6,220 6,240 6,260 6,280 6,300

TGATTTATAAGGGATTTTGCCGATTTCGGCCTATTGGTTAAAAAATGAGCTGATTTAACAAAAATTTAACGCGAATTTTAACAAAATATTAACGCTTACAATTTCCT
ACTAAATATTCCCTAAAACGGCTAAAGCCGGATAACCAATTTTTTACTCGACTAAATTGTTTTTAAATTGCGCTTAAAATTGTTTTATAATTGCGAATGTTAAAGGA
6,320 6,340 6,360 6,380 6,400 6,420

GATGCGGTATTTTCTCCTTACGCATCTGTGCGGTATTTCACACCGCATAGGGTAATAACTGATATAATTAAATTGAAGCTCTAATTTGTGAGTTTAGTATACATGCA
CTACGCCATAAAAGAGGAATGCGTAGACACGCCATAAAGTGTGGCGTATCCCATTATTGACTATATTAATTTAACTTCGAGATTAAACACTCAAATCATATGTACGT

6,440 6,460 6,480 6,500 6,520

TTTACTTATAATACAGTTTTTTAAGCAAGGATTTTCTTAACTTCTTCGGCGACAGCATCACCGACTTCGGTGGTACTGTTGGAACCACCTAAATCACCAGTTCTGAT
AAATGAATATTATGTCAAAAAATTCGTTCCTAAAAGAATTGAAGAAGCCGCTGTCGTAGTGGCTGAAGCCACCATGACAACCTTGGTGGATTTAGTGGTCAAGACTA
LEU2

6,540 6,560 6,580 6,600 6,620

ACCTGCATCCAAAACCTTTTTAACTGCATCTTCAATGGCCTTACCTTCTTCAGGCAAGTTCAATGACAATTTCAACATCATTGCAGCAGACAAGATAGTGGCGATAG
TGGACGTAGGTTTTGGAAAAATTGACGTAGAAGTTACCGGAATGGAAGAAGTCCGTTCAAGTTACTGTTAAAGTTGTAGTAACGTCGTCTGTTCTATCACCGCTATC
LEU2

6,640 6,660 6,680 6,700 6,720 6,740

GGTTGACCTTATTCTTTGGCAAATCTGGAGCAGAACCGTGGCATGGTTCGTACAAACCAAATGCGGTGTTCTTGTCTGGCAAAGAGGCCAAGGACGCAGATGGCAAC
CCAACTGGAATAAGAAACCGTTTAGACCTCGTCTTGGCACCGTACCAAGCATGTTTGGTTTACGCCACAAGAACAGACCGTTTCTCCGGTTCCTGCGTCTACCGTTG
LEU2

6,760 6,780 6,800 6,820 6,840

AAACCCAAGGAACCTGGGATAACGGAGGCTTCATCGGAGATGATATCACCAAACATGTTGCTGGTGATTATAATACCATTTAGGTGGGTTGGGTTCTTAACTAGGAT
TTTGGGTTCCTTGGACCCTATTGCCTCCGAAGTAGCCTCTACTATAGTGGTTTGTACAACGACCACTAATATTATGGTAAATCCACCCAACCCAAGAATTGATCCTA
LEU2

6,860 6,880 6,900 6,920 6,940

CATGGCGGCAGAATCAATCAATTGATGTTGAACCTTCAATGTAGGGAATTCGTTCTTGATGGTTTCCTCCACAGTTTTTCTCCATAATCTTGAAGAGGCCAAAACAT
GTACCGCCGTCTTAGTTAGTTAACTACAACTTGGAAGTTACATCCCTTAAGCAAGAACTACCAAAGGAGGTGTCAAAAAGAGGTATTAGAACTTCTCCGGTTTTGTA
LEU2

6,960 6,980 7,000 7,020 7,040 7,060

TAGCTTTATCCAAGGACCAAATAGGCAAT
ATCGAAATAGGTTCCTGGTTTATCCGTTA
LEU2

7,070 7,080 7,090

ST/SC ligation / pNb_Syn Nb_d-FLAG (pRS415) (71...

GGTGGCTCATGTTGTAGGGCCATGAAAGCGGCCATTCTTGTGATTCTTTGCACTTCTGGAACGGTGTATTGTTCACTATCCCAAGCGACACCATCACCATCGTCTTC
CCACCGAGTACAACATCCCGGTACTTTCGCCGGTAAGAACACTAAGAAACGTGAAGACCTTGCCACATAACAAGTGATAGGGTTCGCTGTGGTAGTGGTAGCAGAAG
LEU2
20 40 60 80 100

CTTTCTCTTACCAAAGTAAATACCTCCCACTAATTCTCTGACAACAACGAAGTCAGTACCTTTAGCAAATTGTGGCTTGATTGGAGATAAGTCTAAAAGAGAGTCGG
GAAAGAGAATGGTTTCATTTATGGAGGGTGATTAAGAGACTGTTGTTGCTTCAGTCATGGAAATCGTTTAACACCGAACTAACCTCTATTCAGATTTTCTCTCAGCC
LEU2
120 140 160 180 200

ATGCAAAGTTACATGGTCTTAAGTTGGCGTACAATTGAAGTTCTTTACGGATTTTTAGTAAACCTTGTTCAGGTCTAACACTACCGGTACCCCATTTAGGACCACCC
TACGTTTCAATGTACCAGAATTCAACCGCATGTTAACTTCAAGAAATGCCTAAAAATCATTTGGAACAAGTCCAGATTGTGATGGCCATGGGGTAAATCCTGGTGGG
LEU2
220 240 260 280 300 320

ACAGCACCTAACAAAACGGCATCAGCCTTCTTGGAGGCTTCCAGCGCCTCATCTGGAAGTGGAACACCTGTAGCATCGATAGCAGCACCACCAATTAAATGATTTTC
TGTCGTGGATTGTTTTGCCGTAGTCGGAAGAACCTCCGAAGGTCGCGGAGTAGACCTTCACCTTGTGGACATCGTAGCTATCGTCGTGGTGGTTAATTTACTAAAAG
LEU2
340 360 380 400 420

GAAATCGAACTTGACATTGGAACGAACATCAGAAATAGCTTTAAGAACCTTAATGGCTTCGGCTGTGATTTCTTGACCAACGTGGTCACCTGGCAAAACGACGATCT
CTTTAGCTTGAACTGTAACCTTGCTTGTAGTCTTTATCGAAATTCTTGGAATTACCGAAGCCGACACTAAAGAACTGGTTGCACCAGTGGACCGTTTTGCTGCTAGA
LEU2
440 460 480 500 520

TCTTAGGGGCAGACATTAGAATGGTATATCCTTGAAATATATATATATATATATNTNGCTGAAATGTAAAAGGTAAGAAAAGTTAGAAAGTAAGACGATTGCTAACCAC
AGAATCCCCGTCTGTAATCTTACCATATAGGAACTTTATATATATATATATATANANCGACTTTACATTTTCCATTCTTTTCAATCTTTCATTCTGCTAACGATTGGTG
LEU2
540 560 580 600 620 640

CTATTGGAAAAAACAATAGGTCCTTAAATAATATTGTCAACTTCAAGTATTGTGATGCAAGCATTTAGTCATGAACGCTTCTCTATTCTATATGAAAAGCCGGTTCC
GATAACCTTTTTTGTTATCCAGGAATTTATTATAACAGTTGAAGTTCATAACACTACGTTCGTAAATCAGTACTTGCGAAGAGATAAGATATACTTTTCGGCCAAGG
660 680 700 720 740

GGCGCTCTCACCTTTCCTTTTTCTCCCAATTTTTCAGTTGAAAAAGGTATATGCGTCAGGCGACCTCTGAAATTAACAAAAAATTTCCAGTCATCGAATTTGATTCT
CCGCGAGAGTGGAAAGGAAAAAGAGGGTTAAAAAGTCAACTTTTTCCATATACGCAGTCCGCTGGAGACTTTAATTGTTTTTTAAAGGTCAGTAGCTTAAACTAAGA
760 780 800 820 840

GTGCGATAGCGCCCCTGTGTGTTCTCGTTATGTTGAGGAAAAAAATAATGGTTGCTAAGAGATTCGAACTCTTGCATCTTACGATACCTGAGTATTCCCACAGTTTG
CACGCTATCGCGGGGACACACAAGAGCAATACAACTCCTTTTTTTATTACCAACGATTCTCTAAGCTTGAGAACGTAGAATGCTATGGACTCATAAGGGTGTCAAAC
860 880 900 920 940 960

AAAAGCTGTGGTATGGTGCACTCTCAGTACAATCTGCTCTGATGCCGCATAGTTAAGCCAGCCCCGACACCCGCCAACACCCGCTGACGCGCCCTGACGGGCTTGTC
TTTTCGACACCATACCACGTGAGAGTCATGTTAGACGAGACTACGGCGTATCAATTCGGTCGGGGCTGTGGGCGGTTGTGGGCGACTGCGCGGGACTGCCCGAACAG

980 1,000 1,020 1,040 1,060

TGCTCCCGGCATCCGCTTACAGACAAGCTGTGACCGTCTCCGGGAGCTGCATGTGTCAGAGGTTTTCACCGTCATCACCGAAACGCGCGAGACGAAAGGGCCTCGTG
ACGAGGGCCGTAGGCGAATGTCTGTTCGACACTGGCAGAGGCCCTCGACGTACACAGTCTCCAAAAGTGGCAGTAGTGGCTTTGCGCGCTCTGCTTTCCCGGAGCAC

1,080 1,100 1,120 1,140 1,160

ATACGCCTATTTTTATAGGTTAATGTCATGATAATAATGGTTTCTTAGTAgatcgcttgcctgtaacttacacgcgcctcgtatcttttaatgatggaataatttgg
TATGCGGATAAAAATATCCAATTACAGTACTATTATTACCAAAGAATCATctagcgaacggacattgaatgtgcgcggagcatagaaaattactaccttattaaacc

CEN/ARS

1,180 1,200 1,220 1,240 1,260 1,280

gaatttactctgtgtttatttattttttatgttttgtatttggatttttagaaagtaaataaagaaggtagaagagttacggaatgaagaaaaaaaaaataaacaaaggt
cttaaatgagacacaaataaataaaaatacaaaacataaacctaaaatctttcatttatttcttccatcttctcaatgccttacttcttttttttttatttgtttcca

CEN/ARS

1,300 1,320 1,340 1,360 1,380

ttaaaaaatttcaacaaaaagcgtactttacatatatatttattagacaagaaaagcagattaaatagatatacattcgattaacgataagtaaaatgtaaaatcac
aatttttttaaagttgtttttcgcatgaaatgtatatataaataatctgttcttttcgtctaatttatctatatgtaagctaattgctattcattttacattttagtg

CEN/ARS

1,400 1,420 1,440 1,460 1,480

aggattttcgtgtgtggtcttctacacagacaagatgaaacaattcggcattaatacctgagagcaggaagagcaagataaaaggtagtatttgttggcgatccccc
tcctaaaagcacacaccagaagatgtgtctgttctactttgttaagccgtaattatggactctcgtccttctcgttctattttccatcataaacaaccgctaggggg

CEN/ARS

1,500 1,520 1,540 1,560 1,580 1,600

tagagtcttttacatcttcggaaaacaaaaactatttttttctttaatttctttttttactttctatttttaatttatatatttatattaaaaaatttaaattataat
atctcagaaaatgtagaagccttttgtttttgataaaaaagaaattaaagaaaaaaatgaaagataaaaattaaatatataaatataatttttttaaatttaatatta

CEN/ARS

1,620 1,640 1,660 1,680 1,700

tatttttatagcacgtgatGTTCAGGTGGCACTTTTCGGGGAAATGTGCGCGGAACCCCTATTTGTTTATTTTTCTAAATACATTCAAATATGTATCCGCTCATGAG
ataaaaatatcgtgcactaCAAGTCCACCGTGAAAAGCCCCTTTACACGCGCCTTGGGGATAAACAAATAAAAAGATTTATGTAAGTTTATACATAGGCGAGTACTC

CEN/ARS

1,720 1,740 1,760 1,780 1,800

ACAATAACCCTGATAAATGCTTCAATAATATTGAAAAAGGAAGAGTATGAGTATTCAACATTTCCGTGTCGCCCTTATTCCCTTTTTTGCGGCATTTTGCCTTCCTG
TGTTATTGGGACTATTTACGAAGTTATTATAACTTTTTCCTTCTCATACTCATAAGTTGTAAAGGCACAGCGGGAATAAGGGAAAAAACGCCGTAAAACGGAAGGAC

1,820 1,840 1,860 1,880 1,900 1,920

TTTTTGCTCACCCAGAAACGCTGGTGAAAGTAAAAGATGCTGAAGATCAGTTGGGTGCACGAGTGGGTTACATCGAACTGGATCTCAACAGCGGTAAGATCCTTGAG
AAAAACGAGTGGGTCTTTGCGACCACTTTCATTTTCTACGACTTCTAGTCAACCCACGTGCTCACCCAATGTAGCTTGACCTAGAGTTGTCGCCATTCTAGGAACTC

1,940 1,960 1,980 2,000 2,020

AGTTTTCGCCCCGAAGAACGTTTTCCAATGATGAGCACTTTTAAAGTTCTGCTATGTGGCGCGGTATTATCCCGTATTGACGCCGGGCAAGAGCAACTCGGTCGCCG
TCAAAAGCGGGGCTTCTTGCAAAAGGTTACTACTCGTGAAAATTTCAAGACGATACACCGCGCCATAATAGGGCATAACTGCGGCCCGTTCTCGTTGAGCCAGCGGC

AmpR

2,040 2,060 2,080 2,100 2,120 2,140

CATACACTATTCTCAGAATGACTTGGTTGAGTACTCACCAGTCACAGAAAAGCATCTTACGGATGGCATGACAGTAAGAGAATTATGCAGTGCTGCCATAACCATGA
GTATGTGATAAGAGTCTTACTGAACCAACTCATGAGTGGTCAGTGTCTTTTCGTAGAATGCCTACCGTACTGTCATTCTCTTAATACGTCACGACGGTATTGGTACT

AmpR

2,160 2,180 2,200 2,220 2,240

GTGATAACACTGCGGCCAACTTACTTCTGACAACGATCGGAGGACCGAAGGAGCTAACCGCTTTTTTGCACAACATGGGGGATCATGTAACTCGCCTTGATCGTTGG
CACTATTGTGACGCCGGTTGAATGAAGACTGTTGCTAGCCTCCTGGCTTCCTCGATTGGCGAAAAAACGTGTTGTACCCCCTAGTACATTGAGCGGAACTAGCAACC

AmpR

2,260 2,280 2,300 2,320 2,340

GAACCGGAGCTGAATGAAGCCATACCAAACGACGAGCGTGACACCACGATGCCTGTAGCAATGGCAACAACGTTGCGCAAACTATTAACTGGCGAACTACTTACTCT
CTTGGCCTCGACTTACTTCGGTATGGTTTGCTGCTCGCACTGTGGTGCTACGGACATCGTTACCGTTGTTGCAACGCGTTTGATAATTGACCGCTTGATGAATGAGA

AmpR

2,360 2,380 2,400 2,420 2,440 2,460

AGCTTCCCGGCAACAATTAATAGACTGGATGGAGGCGGATAAAGTTGCAGGACCACTTCTGCGCTCGGCCCTTCCGGCTGGCTGGTTTATTGCTGATAAATCTGGAG
TCGAAGGGCCGTTGTTAATTATCTGACCTACCTCCGCCTATTTCAACGTCCTGGTGAAGACGCGAGCCGGGAAGGCCGACCGACCAAATAACGACTATTTAGACCTC

AmpR

2,480 2,500 2,520 2,540 2,560

CCGGTGAGCGTGGGTCTCGCGGTATCATTGCAGCACTGGGGCCAGATGGTAAGCCCTCCCGTATCGTAGTTATCTACACGACGGGGAGTCAGGCAACTATGGATGAA
GGCCACTCGCACCCAGAGCGCCATAGTAACGTCGTGACCCCGGTCTACCATTCGGGAGGGCATAGCATCAATAGATGTGCTGCCCCTCAGTCCGTTGATACCTACTT

AmpR

2,580 2,600 2,620 2,640 2,660

CGAAATAGACAGATCGCTGAGATAGGTGCCTCACTGATTAAGCATTGGTAACTGTCAGACCAAGTTTACTCATATATACTTTAGATTGATTTAAAACTTCATTTTTA
GCTTTATCTGTCTAGCGACTCTATCCACGGAGTGACTAATTCGTAACCATTGACAGTCTGGTTCAAATGAGTATATATGAAATCTAACTAAATTTTGAAGTAAAAAT

AmpR

2,680 2,700 2,720 2,740 2,760 2,780

ATTTAAAAGGATCTAGGTGAAGATCCTTTTTGATAATCTCATGACCAAAATCCCTTAACGTGAGTTTTCGTTCCACTGAGCGTCAGACCCCGTAGAAAAGATCAAAG
TAAATTTTCCTAGATCCACTTCTAGGAAAAACTATTAGAGTACTGGTTTTAGGGAATTGCACTCAAAAGCAAGGTGACTCGCAGTCTGGGGCATCTTTTCTAGTTTC

2,800 2,820 2,840 2,860 2,880

```
GATCTTCTTGAGATCCTTTTTTTCTGCGCGTAATCTGCTGCTTGCAAACAAAAAAACCACCGCTACCAGCGGTGGTTTGTTTGCCGGATCAAGAGCTACCAACTCTT
CTAGAAGAACTCTAGGAAAAAAAGACGCGCATTAGACGACGAACGTTTGTTTTTTTGGTGGCGATGGTCGCCACCAAACAAACGGCCTAGTTCTCGATGGTTGAGAA
        2,900                2,920                2,940                2,960                2,980

TTTCCGAAGGTAACTGGCTTCAGCAGAGCGCAGATACCAAATACTGTTCTTCTAGTGTAGCCGTAGTTAGGCCACCACTTCAAGAACTCTGTAGCACCGCCTACATA
AAAGGCTTCCATTGACCGAAGTCGTCTCGCGTCTATGGTTTATGACAAGAAGATCACATCGGCATCAATCCGGTGGTGAAGTTCTTGAGACATCGTGGCGGATGTAT
3,000                3,020                3,040                3,060                3,080                3,100

CCTCGCTCTGCTAATCCTGTTACCAGTGGCTGCTGCCAGTGGCGATAAGTCGTGTCTTACCGGGTTGGACTCAAGACGATAGTTACCGGATAAGGCGCAGCGGTCGG
GGAGCGAGACGATTAGGACAATGGTCACCGACGACGGTCACCGCTATTCAGCACAGAATGGCCCAACCTGAGTTCTGCTATCAATGGCCTATTCCGCGTCGCCAGCC
               3,120                3,140                3,160                3,180                3,200

GCTGAACGGGGGGTTCGTGCACACAGCCCAGCTTGGAGCGAACGACCTACACCGAACTGAGATACCTACAGCGTGAGCTATGAGAAAGCGCCACGCTTCCCGAAGGG
CGACTTGCCCCCCAAGCACGTGTGTCGGGTCGAACCTCGCTTGCTGGATGTGGCTTGACTCTATGGATGTCGCACTCGATACTCTTTCGCGGTGCGAAGGGCTTCCC
       3,220                3,240                3,260                3,280                3,300

AGAAAGGCGGACAGGTATCCGGTAAGCGGCAGGGTCGGAACAGGAGAGCGCACGAGGGAGCTTCCAGGGGGAAACGCCTGGTATCTTTATAGTCCTGTCGGGTTTCG
TCTTTCCGCCTGTCCATAGGCCATTCGCCGTCCCAGCCTTGTCCTCTCGCGTGCTCCCTCGAAGGTCCCCCTTTGCGGACCATAGAAATATCAGGACAGCCCAAAGC
3,320                3,340                3,360                3,380                3,400                3,420

CCACCTCTGACTTGAGCGTCGATTTTTGTGATGCTCGTCAGGGGGGCGGAGCCTATGGAAAAACGCCAGCAACGCGGCCTTTTTACGGTTCCTGGCCTTTTGCTGGC
GGTGGAGACTGAACTCGCAGCTAAAAACACTACGAGCAGTCCCCCCGCCTCGGATACCTTTTTGCGGTCGTTGCGCCGGAAAAATGCCAAGGACCGGAAAACGACCG
              3,440                3,460                3,480                3,500                3,520

CTTTTGCTCACATGTTCTTTCCTGCGTTATCCCCTGATTCTGTGGATAACCGTATTACCGCCTTTGAGTGAGCTGATACCGCTCGCCGCAGCCGAACGACCGAGCGC
GAAAACGAGTGTACAAGAAAGGACGCAATAGGGGACTAAGACACCTATTGGCATAATGGCGGAAACTCACTCGACTATGGCGAGCGGCGTCGGCTTGCTGGCTCGCG
      3,540                3,560                3,580                3,600                3,620

AGCGAGTCAGTGAGCGAGGAAGCGGAAGAGCGCCCAATACGCAAACCGCCTCTCCCCGCGCGTTGGCCGATTCATTAATGCAGCTGGCACGACAGGTTTCCCGACTG
TCGCTCAGTCACTCGCTCCTTCGCCTTCTCGCGGGTTATGCGTTTGGCGGAGAGGGGCGCGCAACCGGCTAAGTAATTACGTCGACCGTGCTGTCCAAAGGGCTGAC
3,640                3,660                3,680                3,700                3,720                3,740

GAAAGCGGCCAGTGAGCGCGCGCAAATTAAAGCCTTCGAGCGTCCCAAAACGCAAACCGCCTCTCCCCGCGCGTTGGCCGATTCATTAATGCAGCTGGCACGACAGG
CTTTCGCCGGTCACTCGCGCGCGTTTAATTTCGGAAGCTCGCAGGGTTTTGCGTTTGGCGGAGAGGGGCGCGCAACCGGCTAAGTAATTACGTCGACCGTGCTGTCC
             3,760                3,780                3,800                3,820                3,840

TTTCCCGACTGGAAAGCGGGCAGTGAGCGCAACGCAATTAATGTGAGTTAGCTCACTCATTAGGCACCCCAGGCTTTACACTTTATGCTTCCGGCTCGTATGTTGTG
AAAGGGCTGACCTTTCGCCCGTCACTCGCGTTGCGTTAATTACACTCAATCGAGTGAGTAATCCGTGGGGTCCGAAATGTGAAATACGAAGGCCGAGCATACAACAC
                                                                                       [    lac promoter    >
      3,860                3,880                3,900                3,920                3,940
```

TGGAATTGTGAGCGGATAACAATTTCACACAGGAAACAGCTATGACCATGATTACGCCAAGCGCGCAATTAACCCTCACTAAAGGGAACAAAAGCTGGAGCTcAGTT
ACCTTAACACTCGCCTATTGTTAAAGTGTGTCCTTTGTCGATACTGGTACTAATGCGGTTCGCGCGTTAATTGGGAGTGATTTCCCTTGTTTTCGACCTCGAgTCAA

T3
LacO M13-rev T3 promoter

3,960 3,980 4,000 4,020 4,040 4,060

TATCATTATCAATACTcGCCATTTCAAAGAATACGTAAATAATTAATAGTAGTGATTTTCCTAACTTTATTTAGTCAAAAAATTAGCCTTTTAATTCTGCTGTAACC
ATAGTAATAGTTATGAgCGGTAAAGTTTCTTATGCATTTATTAATTATCATCACTAAAAGGATTGAAATAAATCAGTTTTTTAATCGGAAAATTAAGACGACATTGG

GPD promoter

4,080 4,100 4,120 4,140 4,160

CGTACATGCCCAAAATAGGGGGCGGGTTACACAGAATATATAACATCGTAGGTGTCTGGGTGAACAGTTTATTCCTGGCATCCACTAAATATAATGGAGCCCGCTTT
GCATGTACGGGTTTTATCCCCCGCCCAATGTGTCTTATATATTGTAGCATCCACAGACCCACTTGTCAAATAAGGACCGTAGGTGATTTATATTACCTCGGGCGAAA

GPD promoter

4,180 4,200 4,220 4,240 4,260 4,280

TTAAGCTGGCATCCAGAAAAAAAAAGAATCCCAGCACCAAAATATTGTTTTCTTCACCAACCATCAGTTCATAGGTCCATTCTCTTAGCGCAACTACAGAGAACAGG
AATTCGACCGTAGGTCTTTTTTTTTCTTAGGGTCGTGGTTTTATAACAAAAGAAGTGGTTGGTAGTCAAGTATCCAGGTAAGAGAATCGCGTTGATGTCTCTTGTCC

GPD promoter

4,300 4,320 4,340 4,360 4,380

GGCACAAACAGGCAAAAAACGGGCACAACCTCAATGGAGTGATGCAACCTGCCTGGAGTAAATGATGACACAAGGCAATTGACCCACGCATGTATCTATCTCATTTT
CCGTGTTTGTCCGTTTTTTGCCCGTGTTGGAGTTACCTCACTACGTTGGACGGACCTCATTTACTACTGTGTTCCGTTAACTGGGTGCGTACATAGATAGAGTAAAA

GPD promoter

4,400 4,420 4,440 4,460 4,480

CTTACACCTTCTATTACCTTCTGCTCTCTCTGATTTGGAAAAAGCTGAAAAAAAAGGTTGAAACCAGTTCCCTGAAATTATTCCCCTACTTGACTAATAAGTATATA
GAATGTGGAAGATAATGGAAGACGAGAGAGACTAAACCTTTTTCGACTTTTTTTTCCAACTTTGGTCAAGGGACTTTAATAAGGGGATGAACTGATTATTCATATAT

GPD promoter

4,500 4,520 4,540 4,560 4,580 4,600

AAGACGGTAGGTATTGATTGTAATTCTGTAAATCTATTTCTTAAACTTCTTAAATTCTACTTTTATAGTTAGTCTTTTTTTTAGTTTTAAAACACCAGAACTTAGTT
TTCTGCCATCCATAACTAACATTAAGACATTTAGATAAAGAATTTGAAGAATTTAAGATGAAAATATCAATCAGAAAAAAAATCAAAATTTTGTGGTCTTGAATCAA

GPD promoter

4,620 4,640 4,660 4,680 4,700

TCGACGGATTCTAGAACTAGTGGATCCATGAGATTCCCATCTATCTTCACCGCTGTTTTGTTCGCTGCTTCTTCTGCTTTGGCTGCTCCAGCTAACACCACCACCGA
AGCTGCCTAAGATCTTGATCACCTAGGTACTCTAAGGGTAGATAGAAGTGGCGACAAAACAAGCGACGAAGAAGACGAAACCGACGAGGTCGATTGTGGTGGTGGCT

1 2 3 4 5 6 7 8 9 10 11 12 13 14 15 16 17 18 19 20 21 22 23 24 25 26 27
M R F P S I F T A V L F A A S S A L A A P A N T T T E

GPD promoter Mating factor alpha leader

4,720 4,740 4,760 4,780 4,800

```
AGACGAAACCGCTCAAATCCCAGCTGAAGCTGTTATCGACTACTCTGACTTGGAAGGTGACTTCGACGCTGCTGCTTTGCCATTGTCTAACTCTACCAACAACGGTT
TCTGCTTTGGCGAGTTTAGGGTCGACTTCGACAATAGCTGATGAGACTGAACCTTCCACTGAAGCTGCGACGACGAAACGGTAACAGATTGAGATGGTTGTTGCCAA
28 29 30 31 32 33 34 35 36 37 38 39 40 41 42 43 44 45 46 47 48 49 50 51 52 53 54 55 56 57 58 59 60 61 62
D  E  T  A  Q  I  P  A  E  A  V  I  D  Y  S  D  L  E  G  D  F  D  A  A  A  L  P  L  S  N  S  T  N  N  G
```

Mating factor alpha leader

4,820 4,840 4,860 4,880 4,900 4,920

```
TGTCTTCTACCAACACCACCATCGCTTCTATCGCTGCTAAGGAAGAAGGTGTTCAATTGGACAAGAGAGAAagatctggcggccgcgcatgcgctagcctcgagCAA
ACAGAAGATGGTTGTGGTGGTAGCGAAGATAGCGACGATTCCTTCTTCCACAAGTTAACCTGTTCTCTCTTtctagaccgccggcgcgtacgcgatcggagctcGTT
63 64 65 66 67 68 69 70 71 72 73 74 75 76 77 78 79 80 81 82 83 84 85 86
L  S  S  T  N  T  T  I  A  S  I  A  A  K  E  E  G  V  Q  L  D  K  R  E                                    Q
```

Mating factor alpha leader

4,940 4,960 4,980 5,000 5,020

```
GTCCAGCTGGTCGAGTCAGGTGGTGGATCTGTCCAAGCCGGGGGGTCTCTAAGACTATCATGTACGGCGTCAGGCAATATTTTTCGTGTTGCTACTATGTGGTTCAG
CAGGTCGACCAGCTCAGTCCACCACCTAGACAGGTTCGGCCCCCCAGAGATTCTGATAGTACATGCCGCAGTCCGTTATAAAAAGCACAACGATGATACACCAAGTC
2     4     6     8     10    12    14    16    18    20    22    24    26    28    30    32    34    36
V  Q  L  V  E  S  G  G  G  S  V  Q  A  G  G  S  L  R  L  S  C  T  A  S  G  N  I  F  R  V  A  T  M  W  F  R
```

Syn Nb_d

5,040 5,060 5,080 5,100 5,120

```
GCAAGTACCGGGCCAGGAAAGAGAGGCAGTTGCGGAACTTGTTGCCAGTATTGCTTCTGGTGGTGGTACTACCAATTATCGTTTTACTATTAGCAGAGACAACGCAA
CGTTCATGGCCCGGTCCTTTCTCTCCGTCAACGCCTTGAACAACGGTCATAACGAAGACCACCACCATGATGGTTAATAGCAAAATGATAATCGTCTCTGTTGCGTT
38    40    42    44    46    48    50    52    54    56    58    60    62    64    66    68    70    72
Q  V  P  G  Q  E  R  E  A  V  A  E  L  V  A  S  I  A  S  G  G  G  T  T  N  Y  R  F  T  I  S  R  D  N  A
```

Syn Nb_d

5,140 5,160 5,180 5,200 5,220 5,240

```
AAAATACTGTTACGCTACAAATGAATAATCTGAAGCCAGAAGATACTGCGATCTATTATTGTGCCGCAGCGGCTTCTCATTCTAACCGTGAAGCTCTGGGTGCTTCT
TTTTATGACAATGCGATGTTTACTTATTAGACTTCGGTCTTCTATGACGCTAGATAATAACACGGCGTCGCCGAAGAGTAAGATTGGCACTTCGAGACCCACGAAGA
74    76    78    80    82    84    86    88    90    92    94    96    98    100   102   104   106   108
K  N  T  V  T  L  Q  M  N  N  L  K  P  E  D  T  A  I  Y  Y  C  A  A  A  A  S  H  S  N  R  E  A  L  G  A  S
```

Syn Nb_d

5,260 5,280 5,300 5,320 5,340

```
CATGACTATTGGGGACAAGGAACACAGGTCACAGTATCATCTGGCGGAGGAGGTTCCGGTGGAGGGGGGTCAGACTATAAGGATGACGACGATAAGGCTGGAGGTTC
GTACTGATAACCCCTGTTCCTTGTGTCCAGTGTCATAGTAGACCGCCTCCTCCAAGGCCACCTCCCCCCAGTCTGATATTCCTACTGCTGCTATTCCGACCTCCAAG
   110   112   114   116   118   120   122 1  2  3  4  5  6  7  8  9  10 1  2  3  4  5  6  7  8  1  2  3  4
H  D  Y  W  G  Q  G  T  Q  V  T  V  S  S  G  G  G  G  S  G  G  G  G  S  D  Y  K  D  D  D  D  K  A  G  G  S
```

Syn Nb_d FLAG tag

5,360 5,380 5,400 5,420 5,440

GAGGGGAGTCCCTCACATAGTGATGGTGGATGCTTACAAAAGGTATAAATAACCCGGGCTGCAGGAATTCGATATCAAGCTTATCGATACCGTCGACCTCGAGTCAT
CTCCCCTCAGGGAGTGTATCACTACCACCTACGAATGTTTTCCATATTTATTGGGCCCGACGTCCTTAAGCTATAGTTCGAATAGCTATGGCAGCTGGAGCTCAGTA

1 2 3 4 5 6 7 8 9 10 11 12 13 14 15 16 1
R G V P H I V M V D A Y K R Y K *

SpyTag

5,460 5,480 5,500 5,520 5,540 5,560

GTAATTAGTTATGTCACGCTTACGTTCACGCCCTCCCCCCACATCCGCTCTAACCGAAAAGGAAGGAGTTAGACAACCTGAAGTCTAGGTCCCTATTTATTTTTTTA
CATTAATCAATACAGTGCGAATGCAAGTGCGGGAGGGGGGTGTAGGCGAGATTGGCTTTTCCTTCCTCAATCTGTTGGACTTCAGATCCAGGGATAAATAAAAAAAT

CYC1 terminator

5,580 5,600 5,620 5,640 5,660

TAGTTATGTTAGTATTAAGAACGTTATTTATATTTCAAATTTTTCTTTTTTTTCTGTACAGACGCGTGTACGCATGTAACATTATACTGAAAACCTTGCTTGAGAAG
ATCAATACAATCATAATTCTTGCAATAAATATAAAGTTTAAAAAGAAAAAAAAGACATGTCTGCGCACATGCGTACATTGTAATATGACTTTTGGAACGAACTCTTC

CYC1 terminator

5,680 5,700 5,720 5,740 5,760

GTTTTGGGACGCTCGAAGGCTTTAATTTGCGCGCGCTCACTGGCCGTCGTTTTACAACGTCGTGACTGGGAAAACCCTGGCGTTACCCAACTTAATCGCCTTGCAGC
CAAAACCCTGCGAGCTTCCGAAATTAAACGCGCGCGAGTGACCGGCAGCAAAATGTTGCAGCACTGACCCTTTTGGGACCGCAATGGGTTGAATTAGCGGAACGTCG

CYC1 terminator M13-fwd

5,780 5,800 5,820 5,840 5,860 5,880

ACATCCCCCTTTCGCCAGCTGGCGTAATAGCGAAGAGGCCCGCACCGATCGCCCTTCCCAACAGTTGCGCAGCCTGAATGGCGAATGGACGCGCCCTGTAGCGGCGC
TGTAGGGGGAAAGCGGTCGACCGCATTATCGCTTCTCCGGGCGTGGCTAGCGGGAAGGGTTGTCAACGCGTCGGACTTACCGCTTACCTGCGCGGGACATCGCCGCG

LacZ alpha

5,900 5,920 5,940 5,960 5,980

ATTAAGCGCGGCGGGTGTGGTGGTTACGCGCAGCGTGACCACTACACTTGCCAGCGCCCTAGCGCCCGCTCCTTTCGCTTTCTTCCCTTCCTTTCTCGCCACGTTCG
TAATTCGCGCCGCCCACACCACCAATGCGCGTCGCACTGGTGATGTGAACGGTCGCGGGATCGCGGGCGAGGAAAGCGAAAGAAGGGAAGGAAAGAGCGGTGCAAGC

6,000 6,020 6,040 6,060 6,080

CCGGCTTTCCCCGTCAAGCTCTAAATCGGGGGCTCCCTTTAGGGTTCCGATTTAGTGCTTTACGGCACCTCGACCCCAAAAAACTTGATTAGGGTGATGGTTCACGT
GGCCGAAAGGGGCAGTTCGAGATTTAGCCCCCGAGGGAAATCCCAAGGCTAAATCACGAAATGCCGTGGAGCTGGGGTTTTTTGAACTAATCCCACTACCAAGTGCA

6,100 6,120 6,140 6,160 6,180 6,200

AGTGGGCCATCGCCCTGATAGACGGTTTTTCGCCCTTTGACGTTGGAGTCCACGTTCTTTAATAGTGGACTCTTGTTCCAAACTGGAACAACACTCAACCCTATCTC
TCACCCGGTAGCGGGACTATCTGCCAAAAAGCGGGAAACTGCAACCTCAGGTGCAAGAAATTATCACCTGAGAACAAGGTTTGACCTTGTTGTGAGTTGGGATAGAG

6,220 6,240 6,260 6,280 6,300

GGTCTATTCTTTTGATTTATAAGGGATTTTGCCGATTTCGGCCTATTGGTTAAAAAATGAGCTGATTTAACAAAAATTTAACGCGAATTTTAACAAAATATTAACGC
CCAGATAAGAAAACTAAATATTCCCTAAAACGGCTAAAGCCGGATAACCAATTTTTTACTCGACTAAATTGTTTTTAAATTGCGCTTAAAATTGTTTTATAATTGCG

6,320 6,340 6,360 6,380 6,400 6,420

TTACAATTTCCTGATGCGGTATTTTCTCCTTACGCATCTGTGCGGTATTTCACACCGCATAGGGTAATAACTGATATAATTAAATTGAAGCTCTAATTTGTGAGTTT
AATGTTAAAGGACTACGCCATAAAAGAGGAATGCGTAGACACGCCATAAAGTGTGGCGTATCCCATTATTGACTATATTAATTTAACTTCGAGATTAAACACTCAAA

6,440 6,460 6,480 6,500 6,520

AGTATACATGCATTTACTTATAATACAGTTTTTTAAGCAAGGATTTTCTTAACTTCTTCGGCGACAGCATCACCGACTTCGGTGGTACTGTTGGAACCACCTAAATC
TCATATGTACGTAAATGAATATTATGTCAAAAAATTCGTTCCTAAAAGAATTGAAGAAGCCGCTGTCGTAGTGGCTGAAGCCACCATGACAACCTTGGTGGATTTAG

LEU2

6,540 6,560 6,580 6,600 6,620

ACCAGTTCTGATACCTGCATCCAAAACCTTTTTAACTGCATCTTCAATGGCCTTACCTTCTTCAGGCAAGTTCAATGACAATTTCAACATCATTGCAGCAGACAAGA
TGGTCAAGACTATGGACGTAGGTTTTGGAAAAATTGACGTAGAAGTTACCGGAATGGAAGAAGTCCGTTCAAGTTACTGTTAAAGTTGTAGTAACGTCGTCTGTTCT

LEU2

6,640 6,660 6,680 6,700 6,720 6,740

TAGTGGCGATAGGGTTGACCTTATTCTTTGGCAAATCTGGAGCAGAACCGTGGCATGGTTCGTACAAACCAAATGCGGTGTTCTTGTCTGGCAAAGAGGCCAAGGAC
ATCACCGCTATCCCAACTGGAATAAGAAACCGTTTAGACCTCGTCTTGGCACCGTACCAAGCATGTTTGGTTTACGCCACAAGAACAGACCGTTTCTCCGGTTCCTG

LEU2

6,760 6,780 6,800 6,820 6,840

GCAGATGGCAACAAACCCAAGGAACCTGGGATAACGGAGGCTTCATCGGAGATGATATCACCAAACATGTTGCTGGTGATTATAATACCATTTAGGTGGGTTGGGTT
CGTCTACCGTTGTTTGGGTTCCTTGGACCCTATTGCCTCCGAAGTAGCCTCTACTATAGTGGTTTGTACAACGACCACTAATATTATGGTAAATCCACCCAACCCAA

LEU2

6,860 6,880 6,900 6,920 6,940

CTTAACTAGGATCATGGCGGCAGAATCAATCAATTGATGTTGAACCTTCAATGTAGGGAATTCGTTCTTGATGGTTTCCTCCACAGTTTTTCTCCATAATCTTGAAG
GAATTGATCCTAGTACCGCCGTCTTAGTTAGTTAACTACAACTTGGAAGTTACATCCCTTAAGCAAGAACTACCAAAGGAGGTGTCAAAAAGAGGTATTAGAACTTC

LEU2

6,960 6,980 7,000 7,020 7,040 7,060

AGGCCAAAACATTAGCTTTATCCAAGGACCAAATAGGCAAT
TCCGGTTTTGTAATCGAAATAGGTTCCTGGTTTATCCGTTA

LEU2

7,070 7,080 7,090 7,100

ST/SC ligation / pNb_Syn Nb_c-FLAG (pRS415) (71...

GGTGGCTCATGTTGTAGGGCCATGAAAGCGGCCATTCTTGTGATTCTTTGCACTTCTGGAACGGTGTATTGTTCACTATCCCAAGCGACACCATCACCATCGTCTTC
CCACCGAGTACAACATCCCGGTACTTTCGCCGGTAAGAACACTAAGAAACGTGAAGACCTTGCCACATAACAAGTGATAGGGTTCGCTGTGGTAGTGGTAGCAGAAG
LEU2
20 40 60 80 100

CTTTCTCTTACCAAAGTAAATACCTCCCACTAATTCTCTGACAACAACGAAGTCAGTACCTTTAGCAAATTGTGGCTTGATTGGAGATAAGTCTAAAAGAGAGTCGG
GAAAGAGAATGGTTTCATTTATGGAGGGTGATTAAGAGACTGTTGTTGCTTCAGTCATGGAAATCGTTTAACACCGAACTAACCTCTATTCAGATTTTCTCTCAGCC
LEU2
120 140 160 180 200

ATGCAAAGTTACATGGTCTTAAGTTGGCGTACAATTGAAGTTCTTTACGGATTTTTAGTAAACCTTGTTCAGGTCTAACACTACCGGTACCCCATTTAGGACCACCC
TACGTTTCAATGTACCAGAATTCAACCGCATGTTAACTTCAAGAAATGCCTAAAAATCATTTGGAACAAGTCCAGATTGTGATGGCCATGGGGTAAATCCTGGTGGG
LEU2
220 240 260 280 300 320

ACAGCACCTAACAAAACGGCATCAGCCTTCTTGGAGGCTTCCAGCGCCTCATCTGGAAGTGGAACACCTGTAGCATCGATAGCAGCACCACCAATTAAATGATTTTC
TGTCGTGGATTGTTTTGCCGTAGTCGGAAGAACCTCCGAAGGTCGCGGAGTAGACCTTCACCTTGTGGACATCGTAGCTATCGTCGTGGTGGTTAATTTACTAAAAG
LEU2
340 360 380 400 420

GAAATCGAACTTGACATTGGAACGAACATCAGAAATAGCTTTAAGAACCTTAATGGCTTCGGCTGTGATTTCTTGACCAACGTGGTCACCTGGCAAAACGACGATCT
CTTTAGCTTGAACTGTAACCTTGCTTGTAGTCTTTATCGAAATTCTTGGAATTACCGAAGCCGACACTAAAGAACTGGTTGCACCAGTGGACCGTTTTGCTGCTAGA
LEU2
440 460 480 500 520

TCTTAGGGGCAGACATTAGAATGGTATATCCTTGAAATATATATATATATATATNTNGCTGAAATGTAAAAGGTAAGAAAAGTTAGAAAGTAAGACGATTGCTAACCAC
AGAATCCCCGTCTGTAATCTTACCATATAGGAACTTTATATATATATATATATANANCGACTTTACATTTTCCATTCTTTTCAATCTTTCATTCTGCTAACGATTGGTG
LEU2
540 560 580 600 620 640

CTATTGGAAAAAACAATAGGTCCTTAAATAATATTGTCAACTTCAAGTATTGTGATGCAAGCATTTAGTCATGAACGCTTCTCTATTCTATATGAAAAGCCGGTTCC
GATAACCTTTTTTGTTATCCAGGAATTTATTATAACAGTTGAAGTTCATAACACTACGTTCGTAAATCAGTACTTGCGAAGAGATAAGATATACTTTTCGGCCAAGG
660 680 700 720 740

GGCGCTCTCACCTTTCCTTTTTCTCCCAATTTTTCAGTTGAAAAAGGTATATGCGTCAGGCGACCTCTGAAATTAACAAAAAATTTCCAGTCATCGAATTTGATTCT
CCGCGAGAGTGGAAAGGAAAAAGAGGGTTAAAAAGTCAACTTTTTCCATATACGCAGTCCGCTGGAGACTTTAATTGTTTTTTAAAGGTCAGTAGCTTAAACTAAGA
760 780 800 820 840

GTGCGATAGCGCCCCTGTGTGTTCTCGTTATGTTGAGGAAAAAAATAATGGTTGCTAAGAGATTCGAACTCTTGCATCTTACGATACCTGAGTATTCCCACAGTTTG
CACGCTATCGCGGGGACACACAAGAGCAATACAACTCCTTTTTTTATTACCAACGATTCTCTAAGCTTGAGAACGTAGAATGCTATGGACTCATAAGGGTGTCAAAC
860 880 900 920 940 960

```
AAAAGCTGTGGTATGGTGCACTCTCAGTACAATCTGCTCTGATGCCGCATAGTTAAGCCAGCCCCGACACCCGCCAACACCCGCTGACGCGCCCTGACGGGCTTGTC
TTTTCGACACCATACCACGTGAGAGTCATGTTAGACGAGACTACGGCGTATCAATTCGGTCGGGGCTGTGGGCGGTTGTGGGCGACTGCGCGGGACTGCCCGAACAG
            980                 1,000               1,020               1,040               1,060

TGCTCCCGGCATCCGCTTACAGACAAGCTGTGACCGTCTCCGGGAGCTGCATGTGTCAGAGGTTTTCACCGTCATCACCGAAACGCGCGAGACGAAAGGGCCTCGTG
ACGAGGGCCGTAGGCGAATGTCTGTTCGACACTGGCAGAGGCCCTCGACGTACACAGTCTCCAAAAGTGGCAGTAGTGGCTTTGCGCGCTCTGCTTTCCCGGAGCAC
     1,080               1,100               1,120               1,140               1,160

ATACGCCTATTTTTATAGGTTAATGTCATGATAATAATGGTTTCTTAGTAgatcgcttgcctgtaacttacacgcgcctcgtatcttttaatgatggaataatttgg
TATGCGGATAAAAATATCCAATTACAGTACTATTATTACCAAAGAATCATctagcgaacggacattgaatgtgcgcggagcatagaaaattactaccttattaaacc
                                                   CEN/ARS
1,180               1,200               1,220               1,240               1,260               1,280

gaatttactctgtgtttatttatttttatgttttgtatttggattttagaaagtaaataaagaaggtagaagagttacggaatgaagaaaaaaaaataaacaaaggt
cttaaatgagacacaaataaataaaaatacaaaacataaacctaaaatctttcatttatttcttccatcttctcaatgccttacttctttttttttatttgtttcca
CEN/ARS
               1,300               1,320               1,340               1,360               1,380

ttaaaaaatttcaacaaaaagcgtactttacatatatatttattagacaagaaaagcagattaaatagatatacattcgattaacgataagtaaaatgtaaaatcac
aatttttttaaagttgtttttcgcatgaaatgtatatataaataatctgttcttttcgtctaatttatctatatgtaagctaattgctattcattttacattttagtg
CEN/ARS
      1,400               1,420               1,440               1,460               1,480

aggattttcgtgtgtggtcttctacacagacaagatgaaacaattcggcattaatacctgagagcaggaagagcaagataaaaggtagtatttgttggcgatccccc
tcctaaaagcacacaccagaagatgtgtctgttctactttgttaagccgtaattatggactctcgtccttctcgttctattttccatcataaacaaccgctaggggg
CEN/ARS
1,500               1,520               1,540               1,560               1,580               1,600

tagagtcttttacatcttcggaaaacaaaaactatttttttctttaatttcttttttttactttctatttttaatttatatatttatattaaaaaatttaaattataat
atctcagaaaatgtagaagccttttgtttttgataaaaaagaaattaaagaaaaaaaatgaaagataaaaattaaatatataaatataatttttttaaatttaatatta
CEN/ARS
              1,620               1,640               1,660               1,680               1,700

tatttttatagcacgtgatGTTCAGGTGGCACTTTTCGGGGAAATGTGCGCGGAACCCCTATTTGTTTATTTTTCTAAATACATTCAAATATGTATCCGCTCATGAG
ataaaaatatcgtgcactaCAAGTCCACCGTGAAAAGCCCCTTTACACGCGCCTTGGGGATAAACAAATAAAAAGATTTATGTAAGTTTATACATAGGCGAGTACTC
CEN/ARS
      1,720               1,740               1,760               1,780               1,800

ACAATAACCCTGATAAATGCTTCAATAATATTGAAAAAGGAAGAGTATGAGTATTCAACATTTCCGTGTCGCCCTTATTCCCTTTTTTGCGGCATTTTGCCTTCCTG
TGTTATTGGGACTATTTACGAAGTTATTATAACTTTTTCCTTCTCATACTCATAAGTTGTAAAGGCACAGCGGGAATAAGGGAAAAAACGCCGTAAAACGGAAGGAC
1,820               1,840               1,860               1,880               1,900               1,920
```

TTTTTGCTCACCCAGAAACGCTGGTGAAAGTAAAAGATGCTGAAGATCAGTTGGGTGCACGAGTGGGTTACATCGAACTGGATCTCAACAGCGGTAAGATCCTTGAG
AAAAACGAGTGGGTCTTTGCGACCACTTTCATTTTCTACGACTTCTAGTCAACCCACGTGCTCACCCAATGTAGCTTGACCTAGAGTTGTCGCCATTCTAGGAACTC

1,940 1,960 1,980 2,000 2,020

AGTTTTCGCCCCGAAGAACGTTTTCCAATGATGAGCACTTTTAAAGTTCTGCTATGTGGCGCGGTATTATCCCGTATTGACGCCGGGCAAGAGCAACTCGGTCGCCG
TCAAAAGCGGGGCTTCTTGCAAAAGGTTACTACTCGTGAAAATTTCAAGACGATACACCGCGCCATAATAGGGCATAACTGCGGCCCGTTCTCGTTGAGCCAGCGGC

AmpR

2,040 2,060 2,080 2,100 2,120 2,140

CATACACTATTCTCAGAATGACTTGGTTGAGTACTCACCAGTCACAGAAAAGCATCTTACGGATGGCATGACAGTAAGAGAATTATGCAGTGCTGCCATAACCATGA
GTATGTGATAAGAGTCTTACTGAACCAACTCATGAGTGGTCAGTGTCTTTTCGTAGAATGCCTACCGTACTGTCATTCTCTTAATACGTCACGACGGTATTGGTACT

AmpR

2,160 2,180 2,200 2,220 2,240

GTGATAACACTGCGGCCAACTTACTTCTGACAACGATCGGAGGACCGAAGGAGCTAACCGCTTTTTTGCACAACATGGGGGATCATGTAACTCGCCTTGATCGTTGG
CACTATTGTGACGCCGGTTGAATGAAGACTGTTGCTAGCCTCCTGGCTTCCTCGATTGGCGAAAAAACGTGTTGTACCCCCTAGTACATTGAGCGGAACTAGCAACC

AmpR

2,260 2,280 2,300 2,320 2,340

GAACCGGAGCTGAATGAAGCCATACCAAACGACGAGCGTGACACCACGATGCCTGTAGCAATGGCAACAACGTTGCGCAAACTATTAACTGGCGAACTACTTACTCT
CTTGGCCTCGACTTACTTCGGTATGGTTTGCTGCTCGCACTGTGGTGCTACGGACATCGTTACCGTTGTTGCAACGCGTTTGATAATTGACCGCTTGATGAATGAGA

AmpR

2,360 2,380 2,400 2,420 2,440 2,460

AGCTTCCCGGCAACAATTAATAGACTGGATGGAGGCGGATAAAGTTGCAGGACCACTTCTGCGCTCGGCCCTTCCGGCTGGCTGGTTTATTGCTGATAAATCTGGAG
TCGAAGGGCCGTTGTTAATTATCTGACCTACCTCCGCCTATTTCAACGTCCTGGTGAAGACGCGAGCCGGGAAGGCCGACCGACCAAATAACGACTATTTAGACCTC

AmpR

2,480 2,500 2,520 2,540 2,560

CCGGTGAGCGTGGGTCTCGCGGTATCATTGCAGCACTGGGGCCAGATGGTAAGCCCTCCCGTATCGTAGTTATCTACACGACGGGGAGTCAGGCAACTATGGATGAA
GGCCACTCGCACCCAGAGCGCCATAGTAACGTCGTGACCCCGGTCTACCATTCGGGAGGGCATAGCATCAATAGATGTGCTGCCCCTCAGTCCGTTGATACCTACTT

AmpR

2,580 2,600 2,620 2,640 2,660

CGAAATAGACAGATCGCTGAGATAGGTGCCTCACTGATTAAGCATTGGTAACTGTCAGACCAAGTTTACTCATATATACTTTAGATTGATTTAAAACTTCATTTTTA
GCTTTATCTGTCTAGCGACTCTATCCACGGAGTGACTAATTCGTAACCATTGACAGTCTGGTTCAAATGAGTATATATGAAATCTAACTAAATTTTGAAGTAAAAAT

AmpR

2,680 2,700 2,720 2,740 2,760 2,780

ATTTAAAAGGATCTAGGTGAAGATCCTTTTTGATAATCTCATGACCAAAATCCCTTAACGTGAGTTTTCGTTCCACTGAGCGTCAGACCCCGTAGAAAAGATCAAAG
TAAATTTTCCTAGATCCACTTCTAGGAAAAACTATTAGAGTACTGGTTTTAGGGAATTGCACTCAAAAGCAAGGTGACTCGCAGTCTGGGGCATCTTTTCTAGTTTC

2,800 2,820 2,840 2,860 2,880

GATCTTCTTGAGATCCTTTTTTTCTGCGCGTAATCTGCTGCTTGCAAACAAAAAAACCACCGCTACCAGCGGTGGTTTGTTTGCCGGATCAAGAGCTACCAACTCTT
CTAGAAGAACTCTAGGAAAAAAAGACGCGCATTAGACGACGAACGTTTGTTTTTTTGGTGGCGATGGTCGCCACCAAACAAACGGCCTAGTTCTCGATGGTTGAGAA

2,900 2,920 2,940 2,960 2,980

TTTCCGAAGGTAACTGGCTTCAGCAGAGCGCAGATACCAAATACTGTTCTTCTAGTGTAGCCGTAGTTAGGCCACCACTTCAAGAACTCTGTAGCACCGCCTACATA
AAAGGCTTCCATTGACCGAAGTCGTCTCGCGTCTATGGTTTATGACAAGAAGATCACATCGGCATCAATCCGGTGGTGAAGTTCTTGAGACATCGTGGCGGATGTAT

3,000 3,020 3,040 3,060 3,080 3,100

CCTCGCTCTGCTAATCCTGTTACCAGTGGCTGCTGCCAGTGGCGATAAGTCGTGTCTTACCGGGTTGGACTCAAGACGATAGTTACCGGATAAGGCGCAGCGGTCGG
GGAGCGAGACGATTAGGACAATGGTCACCGACGACGGTCACCGCTATTCAGCACAGAATGGCCCAACCTGAGTTCTGCTATCAATGGCCTATTCCGCGTCGCCAGCC

3,120 3,140 3,160 3,180 3,200

GCTGAACGGGGGGTTCGTGCACACAGCCCAGCTTGGAGCGAACGACCTACACCGAACTGAGATACCTACAGCGTGAGCTATGAGAAAGCGCCACGCTTCCCGAAGGG
CGACTTGCCCCCCAAGCACGTGTGTCGGGTCGAACCTCGCTTGCTGGATGTGGCTTGACTCTATGGATGTCGCACTCGATACTCTTTCGCGGTGCGAAGGGCTTCCC

3,220 3,240 3,260 3,280 3,300

AGAAAGGCGGACAGGTATCCGGTAAGCGGCAGGGTCGGAACAGGAGAGCGCACGAGGGAGCTTCCAGGGGGAAACGCCTGGTATCTTTATAGTCCTGTCGGGTTTCG
TCTTTCCGCCTGTCCATAGGCCATTCGCCGTCCCAGCCTTGTCCTCTCGCGTGCTCCCTCGAAGGTCCCCCTTTGCGGACCATAGAAATATCAGGACAGCCCAAAGC

3,320 3,340 3,360 3,380 3,400 3,420

CCACCTCTGACTTGAGCGTCGATTTTTGTGATGCTCGTCAGGGGGGCGGAGCCTATGGAAAAACGCCAGCAACGCGGCCTTTTTACGGTTCCTGGCCTTTTGCTGGC
GGTGGAGACTGAACTCGCAGCTAAAAACACTACGAGCAGTCCCCCCGCCTCGGATACCTTTTTGCGGTCGTTGCGCCGGAAAAATGCCAAGGACCGGAAAACGACCG

3,440 3,460 3,480 3,500 3,520

CTTTTGCTCACATGTTCTTTCCTGCGTTATCCCCTGATTCTGTGGATAACCGTATTACCGCCTTTGAGTGAGCTGATACCGCTCGCCGCAGCCGAACGACCGAGCGC
GAAAACGAGTGTACAAGAAAGGACGCAATAGGGGACTAAGACACCTATTGGCATAATGGCGGAAACTCACTCGACTATGGCGAGCGGCGTCGGCTTGCTGGCTCGCG

3,540 3,560 3,580 3,600 3,620

AGCGAGTCAGTGAGCGAGGAAGCGGAAGAGCGCCCAATACGCAAACCGCCTCTCCCCGCGCGTTGGCCGATTCATTAATGCAGCTGGCACGACAGGTTTCCCGACTG
TCGCTCAGTCACTCGCTCCTTCGCCTTCTCGCGGGTTATGCGTTTGGCGGAGAGGGGCGCGCAACCGGCTAAGTAATTACGTCGACCGTGCTGTCCAAAGGGCTGAC

3,640 3,660 3,680 3,700 3,720 3,740

GAAAGCGGCCAGTGAGCGCGCGCAAATTAAAGCCTTCGAGCGTCCCAAAACGCAAACCGCCTCTCCCCGCGCGTTGGCCGATTCATTAATGCAGCTGGCACGACAGG
CTTTCGCCGGTCACTCGCGCGCGTTTAATTTCGGAAGCTCGCAGGGTTTTGCGTTTGGCGGAGAGGGGCGCGCAACCGGCTAAGTAATTACGTCGACCGTGCTGTCC

3,760 3,780 3,800 3,820 3,840

TTTCCCGACTGGAAAGCGGGCAGTGAGCGCAACGCAATTAATGTGAGTTAGCTCACTCATTAGGCACCCCAGGCTTTACACTTTATGCTTCCGGCTCGTATGTTGTG
AAAGGGCTGACCTTTCGCCCGTCACTCGCGTTGCGTTAATTACACTCAATCGAGTGAGTAATCCGTGGGGTCCGAAATGTGAAATACGAAGGCCGAGCATACAACAC

lac promoter

3,860 3,880 3,900 3,920 3,940

TGGAATTGTGAGCGGATAACAATTTCACACAGGAAACAGCTATGACCATGATTACGCCAAGCGCGCAATTAACCCTCACTAAAGGGAACAAAAGCTGGAGCTcAGTT
ACCTTAACACTCGCCTATTGTTAAAGTGTGTCCTTTGTCGATACTGGTACTAATGCGGTTCGCGCGTTAATTGGGAGTGATTTCCCTTGTTTTCGACCTCGAgTCAA

T3
LacO M13-rev T3 promoter

3,960 3,980 4,000 4,020 4,040 4,060

TATCATTATCAATACTcGCCATTTCAAAGAATACGTAAATAATTAATAGTAGTGATTTTCCTAACTTTATTTAGTCAAAAAATTAGCCTTTTAATTCTGCTGTAACC
ATAGTAATAGTTATGAgCGGTAAAGTTTCTTATGCATTTATTAATTATCATCACTAAAAGGATTGAAATAAATCAGTTTTTTAATCGGAAAATTAAGACGACATTGG

GPD promoter

4,080 4,100 4,120 4,140 4,160

CGTACATGCCCAAAATAGGGGGCGGGTTACACAGAATATATAACATCGTAGGTGTCTGGGTGAACAGTTTATTCCTGGCATCCACTAAATATAATGGAGCCCGCTTT
GCATGTACGGGTTTTATCCCCCGCCCAATGTGTCTTATATATTGTAGCATCCACAGACCCACTTGTCAAATAAGGACCGTAGGTGATTTATATTACCTCGGGCGAAA

GPD promoter

4,180 4,200 4,220 4,240 4,260 4,280

TTAAGCTGGCATCCAGAAAAAAAAAGAATCCCAGCACCAAAATATTGTTTTCTTCACCAACCATCAGTTCATAGGTCCATTCTCTTAGCGCAACTACAGAGAACAGG
AATTCGACCGTAGGTCTTTTTTTTTCTTAGGGTCGTGGTTTTATAACAAAAGAAGTGGTTGGTAGTCAAGTATCCAGGTAAGAGAATCGCGTTGATGTCTCTTGTCC

GPD promoter

4,300 4,320 4,340 4,360 4,380

GGCACAAACAGGCAAAAAACGGGCACAACCTCAATGGAGTGATGCAACCTGCCTGGAGTAAATGATGACACAAGGCAATTGACCCACGCATGTATCTATCTCATTTT
CCGTGTTTGTCCGTTTTTTGCCCGTGTTGGAGTTACCTCACTACGTTGGACGGACCTCATTTACTACTGTGTTCCGTTAACTGGGTGCGTACATAGATAGAGTAAAA

GPD promoter

4,400 4,420 4,440 4,460 4,480

CTTACACCTTCTATTACCTTCTGCTCTCTCTGATTTGGAAAAAGCTGAAAAAAAAAGGTTGAAACCAGTTCCCTGAAATTATTCCCCTACTTGACTAATAAGTATATA
GAATGTGGAAGATAATGGAAGACGAGAGAGACTAAACCTTTTTCGACTTTTTTTTTCCAACTTTGGTCAAGGGACTTTAATAAGGGGATGAACTGATTATTCATATAT

GPD promoter

4,500 4,520 4,540 4,560 4,580 4,600

AAGACGGTAGGTATTGATTGTAATTCTGTAAATCTATTTCTTAAACTTCTTAAATTCTACTTTTATAGTTAGTCTTTTTTTTAGTTTTAAAACACCAGAACTTAGTT
TTCTGCCATCCATAACTAACATTAAGACATTTAGATAAAGAATTTGAAGAATTTAAGATGAAAATATCAATCAGAAAAAAAATCAAAATTTTGTGGTCTTGAATCAA

GPD promoter

4,620 4,640 4,660 4,680 4,700

TCGACGGATTCTAGAACTAGTGGATCCATGAGATTCCCATCTATCTTCACCGCTGTTTTGTTCGCTGCTTCTTCTGCTTTGGCTGCTCCAGCTAACACCACCACCGA
AGCTGCCTAAGATCTTGATCACCTAGGTACTCTAAGGGTAGATAGAAGTGGCGACAAAACAAGCGACGAAGAAGACGAAACCGACGAGGTCGATTGTGGTGGTGGCT

1 2 3 4 5 6 7 8 9 10 11 12 13 14 15 16 17 18 19 20 21 22 23 24 25 26 27
M R F P S I F T A V L F A A S S A L A A P A N T T T E

GPD promoter Mating factor alpha leader

4,720 4,740 4,760 4,780 4,800

```
AGACGAAACCGCTCAAATCCCAGCTGAAGCTGTTATCGACTACTCTGACTTGGAAGGTGACTTCGACGCTGCTGCTTTGCCATTGTCTAACTCTACCAACAACGGTT
TCTGCTTTGGCGAGTTTAGGGTCGACTTCGACAATAGCTGATGAGACTGAACCTTCCACTGAAGCTGCGACGACGAAACGGTAACAGATTGAGATGGTTGTTGCCAA
 28 29 30 31 32 33 34 35 36 37 38 39 40 41 42 43 44 45 46 47 48 49 50 51 52 53 54 55 56 57 58 59 60 61 62
 D  E  T  A  Q  I  P  A  E  A  V  I  D  Y  S  D  L  E  G  D  F  D  A  A  A  L  P  L  S  N  S  T  N  N  G
Mating factor alpha leader
4,820  4,840  4,860  4,880  4,900  4,920
```

```
TGTCTTCTACCAACACCACCATCGCTTCTATCGCTGCTAAGGAAGAAGGTGTTCAATTGGACAAGAGAGAAagatctggcggccgcgcatgcgctagcctcgagCAA
ACAGAAGATGGTTGTGGTGGTAGCGAAGATAGCGACGATTCCTTCTTCCACAAGTTAACCTGTTCTCTCTTtctagaccgccggcgcgtacgcgatcggagctcGTT
63 64 65 66 67 68 69 70 71 72 73 74 75 76 77 78 79 80 81 82 83 84 85 86
 L  S  S  T  N  T  T  I  A  S  I  A  A  K  E  E  G  V  Q  L  D  K  R  E                      Q
Mating factor alpha leader
4,940  4,960  4,980  5,000  5,020
```

```
GTCCAGCTGGTCGAGTCAGGTGGTGGATCTGTCCAAGCCGGGGGGTCTCTAAGACTATCATGTACGGCGTCAGGCTCTATTTCTTACGCTGAAAACATGTGGTTCAG
CAGGTCGACCAGCTCAGTCCACCACCTAGACAGGTTCGGCCCCCCAGAGATTCTGATAGTACATGCCGCAGTCCGAGATAAAGAATGCGACTTTTGTACACCAAGTC
 2     4     6     8     10    12    14    16    18    20    22    24    26    28    30    32    34    36
 V  Q  L  V  E  S  G  G  G  S  V  Q  A  G  G  S  L  R  L  S  C  T  A  S  G  S  I  S  Y  A  E  N  M  W  F  R
Syn Nb_c
5,040  5,060  5,080  5,100  5,120
```

```
GCAAGCACCGGGCCAGGAAAGAGAGGCAGTTGCGGAATTTGTTGCCAGTATTACTTCTGGTAGTATTACCTATTATCGTTTTACTATTAGCAGAGACAACGCAAAAA
CGTTCGTGGCCCGGTCCTTTCTCTCCGTCAACGCCTTAAACAACGGTCATAATGAAGACCATCATAATGGATAATAGCAAAATGATAATCGTCTCTGTTGCGTTTTT
 38    40    42    44    46    48    50    52    54    56    58    60    62    64    66    68    70    72
 Q  A  P  G  Q  E  R  E  A  V  A  E  F  V  A  S  I  T  S  G  S  I  T  Y  Y  R  F  T  I  S  R  D  N  A  K
Syn Nb_c
5,140  5,160  5,180  5,200  5,220  5,240
```

```
ATACTGTTACGCTACAAATGAATAATCTGAAGCCAGAAGATACTGCGATCTATTATTGTGCCGCAGCGGTTAACCGTTACGTTGACCCGTCTTCTCAGCCGCATCAT
TATGACAATGCGATGTTTACTTATTAGACTTCGGTCTTCTATGACGCTAGATAATAACACGGCGTCGCCAATTGGCAATGCAACTGGGCAGAAGAGTCGGCGTAGTA
   74    76    78    80    82    84    86    88    90    92    94    96    98    100   102   104   106   108
 N  T  V  T  L  Q  M  N  N  L  K  P  E  D  T  A  I  Y  Y  C  A  A  A  V  N  R  Y  V  D  P  S  S  Q  P  H  H
Syn Nb_c
5,260  5,280  5,300  5,320  5,340
```

```
TGGTATTGGGGACAAGGAACACAGGTCACAGTATCATCTGGCGGAGGAGGTTCCGGTGGAGGGGGGTCAGACTATAAGGATGACGACGATAAGGCTGGAGGTTCGAG
ACCATAACCCCTGTTCCTTGTGTCCAGTGTCATAGTAGACCGCCTCCTCCAAGGCCACCTCCCCCCAGTCTGATATTCCTACTGCTGCTATTCCGACCTCCAAGCTC
   110   112   114   116   118   120   1  2  3  4  5  6  7  8  9  10 1  2  3  4  5  6  7  8  1  2  3  4  1
 W  Y  W  G  Q  G  T  Q  V  T  V  S  S  G  G  G  G  S  G  G  G  G  S  D  Y  K  D  D  D  D  K  A  G  G  S  R
Syn Nb_c                                            FLAG tag
5,360  5,380  5,400  5,420  5,440
```

GGGAGTCCCTCACATAGTGATGGTGGATGCTTACAAAAGGTATAAATAACCCGGGCTGCAGGAATTCGATATCAAGCTTATCGATACCGTCGACCTCGAGTCATGTA
CCCTCAGGGAGTGTATCACTACCACCTACGAATGTTTTCCATATTTATTGGGCCCGACGTCCTTAAGCTATAGTTCGAATAGCTATGGCAGCTGGAGCTCAGTACAT

2 3 4 5 6 7 8 9 10 11 12 13 14 15 16 1
G V P H I V M V D A Y K R Y K *

SpyTag

5,460 5,480 5,500 5,520 5,540 5,560

ATTAGTTATGTCACGCTTACGTTCACGCCCTCCCCCCACATCCGCTCTAACCGAAAAGGAAGGAGTTAGACAACCTGAAGTCTAGGTCCCTATTTATTTTTTTATAG
TAATCAATACAGTGCGAATGCAAGTGCGGGAGGGGGGTGTAGGCGAGATTGGCTTTTCCTTCCTCAATCTGTTGGACTTCAGATCCAGGGATAAATAAAAAAATATC

CYC1 terminator

5,580 5,600 5,620 5,640 5,660

TTATGTTAGTATTAAGAACGTTATTTATATTTCAAATTTTTCTTTTTTTTCTGTACAGACGCGTGTACGCATGTAACATTATACTGAAAACCTTGCTTGAGAAGGTT
AATACAATCATAATTCTTGCAATAAATATAAAGTTTAAAAAGAAAAAAAAGACATGTCTGCGCACATGCGTACATTGTAATATGACTTTTGGAACGAACTCTTCCAA

CYC1 terminator

5,680 5,700 5,720 5,740 5,760

TTGGGACGCTCGAAGGCTTTAATTTGCGCGCGCTCACTGGCCGTCGTTTTACAACGTCGTGACTGGGAAAACCCTGGCGTTACCCAACTTAATCGCCTTGCAGCACA
AACCCTGCGAGCTTCCGAAATTAAACGCGCGCGAGTGACCGGCAGCAAAATGTTGCAGCACTGACCCTTTTGGGACCGCAATGGGTTGAATTAGCGGAACGTCGTGT

CYC1 terminator M13-fwd

5,780 5,800 5,820 5,840 5,860 5,880

TCCCCCTTTCGCCAGCTGGCGTAATAGCGAAGAGGCCCGCACCGATCGCCCTTCCCAACAGTTGCGCAGCCTGAATGGCGAATGGACGCGCCCTGTAGCGGCGCATT
AGGGGGAAAGCGGTCGACCGCATTATCGCTTCTCCGGGCGTGGCTAGCGGGAAGGGTTGTCAACGCGTCGGACTTACCGCTTACCTGCGCGGGACATCGCCGCGTAA

LacZ alpha

5,900 5,920 5,940 5,960 5,980

AAGCGCGGCGGGTGTGGTGGTTACGCGCAGCGTGACCACTACACTTGCCAGCGCCCTAGCGCCCGCTCCTTTCGCTTTCTTCCCTTCCTTTCTCGCCACGTTCGCCG
TTCGCGCCGCCCACACCACCAATGCGCGTCGCACTGGTGATGTGAACGGTCGCGGGATCGCGGGCGAGGAAAGCGAAAGAAGGGAAGGAAAGAGCGGTGCAAGCGGC

6,000 6,020 6,040 6,060 6,080

GCTTTCCCCGTCAAGCTCTAAATCGGGGGCTCCCTTTAGGGTTCCGATTTAGTGCTTTACGGCACCTCGACCCCAAAAAACTTGATTAGGGTGATGGTTCACGTAGT
CGAAAGGGGCAGTTCGAGATTTAGCCCCCGAGGGAAATCCCAAGGCTAAATCACGAAATGCCGTGGAGCTGGGGTTTTTTGAACTAATCCCACTACCAAGTGCATCA

6,100 6,120 6,140 6,160 6,180 6,200

GGGCCATCGCCCTGATAGACGGTTTTTCGCCCTTTGACGTTGGAGTCCACGTTCTTTAATAGTGGACTCTTGTTCCAAACTGGAACAACACTCAACCCTATCTCGGT
CCCGGTAGCGGGACTATCTGCCAAAAAGCGGGAAACTGCAACCTCAGGTGCAAGAAATTATCACCTGAGAACAAGGTTTGACCTTGTTGTGAGTTGGGATAGAGCCA

6,220 6,240 6,260 6,280 6,300

CTATTCTTTTGATTTATAAGGGATTTTGCCGATTTCGGCCTATTGGTTAAAAAATGAGCTGATTTAACAAAAATTTAACGCGAATTTTAACAAAATATTAACGCTTA
GATAAGAAAACTAAATATTCCCTAAAACGGCTAAAGCCGGATAACCAATTTTTTACTCGACTAAATTGTTTTTAAATTGCGCTTAAAATTGTTTTATAATTGCGAAT

6,320 6,340 6,360 6,380 6,400 6,420

```
CAATTTCCTGATGCGGTATTTTCTCCTTACGCATCTGTGCGGTATTTCACACCGCATAGGGTAATAACTGATATAATTAAATTGAAGCTCTAATTTGTGAGTTTAGT
GTTAAAGGACTACGCCATAAAAGAGGAATGCGTAGACACGCCATAAAGTGTGGCGTATCCCATTATTGACTATATTAATTTAACTTCGAGATTAAACACTCAAATCA
```

6,440 6,460 6,480 6,500 6,520

```
ATACATGCATTTACTTATAATACAGTTTTTTAAGCAAGGATTTTCTTAACTTCTTCGGCGACAGCATCACCGACTTCGGTGGTACTGTTGGAACCACCTAAATCACC
TATGTACGTAAATGAATATTATGTCAAAAAATTCGTTCCTAAAAGAATTGAAGAAGCCGCTGTCGTAGTGGCTGAAGCCACCATGACAACCTTGGTGGATTTAGTGG
```

LEU2

6,540 6,560 6,580 6,600 6,620

```
AGTTCTGATACCTGCATCCAAAACCTTTTTAACTGCATCTTCAATGGCCTTACCTTCTTCAGGCAAGTTCAATGACAATTTCAACATCATTGCAGCAGACAAGATAG
TCAAGACTATGGACGTAGGTTTTGGAAAAATTGACGTAGAAGTTACCGGAATGGAAGAAGTCCGTTCAAGTTACTGTTAAAGTTGTAGTAACGTCGTCTGTTCTATC
```

LEU2

6,640 6,660 6,680 6,700 6,720 6,740

```
TGGCGATAGGGTTGACCTTATTCTTTGGCAAATCTGGAGCAGAACCGTGGCATGGTTCGTACAAACCAAATGCGGTGTTCTTGTCTGGCAAAGAGGCCAAGGACGCA
ACCGCTATCCCAACTGGAATAAGAAACCGTTTAGACCTCGTCTTGGCACCGTACCAAGCATGTTTGGTTTACGCCACAAGAACAGACCGTTTCTCCGGTTCCTGCGT
```

LEU2

6,760 6,780 6,800 6,820 6,840

```
GATGGCAACAAACCCAAGGAACCTGGGATAACGGAGGCTTCATCGGAGATGATATCACCAAACATGTTGCTGGTGATTATAATACCATTTAGGTGGGTTGGGTTCTT
CTACCGTTGTTTGGGTTCCTTGGACCCTATTGCCTCCGAAGTAGCCTCTACTATAGTGGTTTGTACAACGACCACTAATATTATGGTAAATCCACCCAACCCAAGAA
```

LEU2

6,860 6,880 6,900 6,920 6,940

```
AACTAGGATCATGGCGGCAGAATCAATCAATTGATGTTGAACCTTCAATGTAGGGAATTCGTTCTTGATGGTTTCCTCCACAGTTTTTCTCCATAATCTTGAAGAGG
TTGATCCTAGTACCGCCGTCTTAGTTAGTTAACTACAACTTGGAAGTTACATCCCTTAAGCAAGAACTACCAAAGGAGGTGTCAAAAAGAGGTATTAGAACTTCTCC
```

LEU2

6,960 6,980 7,000 7,020 7,040 7,060

```
CCAAAACATTAGCTTTATCCAAGGACCAAATAGGCAAT
GGTTTTGTAATCGAAATAGGTTCCTGGTTTATCCGTTA
```

LEU2

7,070 7,080 7,090 7,100

ST/SC ligation / pNb_Syn Nb_b-FLAG (pRS415) (71...

GGTGGCTCATGTTGTAGGGCCATGAAAGCGGCCATTCTTGTGATTCTTTGCACTTCTGGAACGGTGTATTGTTCACTATCCCAAGCGACACCATCACCATCGTCTTC
CCACCGAGTACAACATCCCGGTACTTTCGCCGGTAAGAACACTAAGAAACGTGAAGACCTTGCCACATAACAAGTGATAGGGTTCGCTGTGGTAGTGGTAGCAGAAG
LEU2
20 40 60 80 100

CTTTCTCTTACCAAAGTAAATACCTCCCACTAATTCTCTGACAACAACGAAGTCAGTACCTTTAGCAAATTGTGGCTTGATTGGAGATAAGTCTAAAAGAGAGTCGG
GAAAGAGAATGGTTTCATTTATGGAGGGTGATTAAGAGACTGTTGTTGCTTCAGTCATGGAAATCGTTTAACACCGAACTAACCTCTATTCAGATTTTCTCTCAGCC
LEU2
120 140 160 180 200

ATGCAAAGTTACATGGTCTTAAGTTGGCGTACAATTGAAGTTCTTTACGGATTTTTAGTAAACCTTGTTCAGGTCTAACACTACCGGTACCCCATTTAGGACCACCC
TACGTTTCAATGTACCAGAATTCAACCGCATGTTAACTTCAAGAAATGCCTAAAAATCATTTGGAACAAGTCCAGATTGTGATGGCCATGGGGTAAATCCTGGTGGG
LEU2
220 240 260 280 300 320

ACAGCACCTAACAAAACGGCATCAGCCTTCTTGGAGGCTTCCAGCGCCTCATCTGGAAGTGGAACACCTGTAGCATCGATAGCAGCACCACCAATTAAATGATTTTC
TGTCGTGGATTGTTTTGCCGTAGTCGGAAGAACCTCCGAAGGTCGCGGAGTAGACCTTCACCTTGTGGACATCGTAGCTATCGTCGTGGTGGTTAATTTACTAAAAG
LEU2
340 360 380 400 420

GAAATCGAACTTGACATTGGAACGAACATCAGAAATAGCTTTAAGAACCTTAATGGCTTCGGCTGTGATTTCTTGACCAACGTGGTCACCTGGCAAAACGACGATCT
CTTTAGCTTGAACTGTAACCTTGCTTGTAGTCTTTATCGAAATTCTTGGAATTACCGAAGCCGACACTAAAGAACTGGTTGCACCAGTGGACCGTTTTGCTGCTAGA
LEU2
440 460 480 500 520

TCTTAGGGGCAGACATTAGAATGGTATATCCTTGAAATATATATATATATATATNTNGCTGAAATGTAAAAGGTAAGAAAAGTTAGAAAGTAAGACGATTGCTAACCAC
AGAATCCCCGTCTGTAATCTTACCATATAGGAACTTTATATATATATATATATANANCGACTTTACATTTTCCATTCTTTTCAATCTTTCATTCTGCTAACGATTGGTG
LEU2
540 560 580 600 620 640

CTATTGGAAAAAACAATAGGTCCTTAAATAATATTGTCAACTTCAAGTATTGTGATGCAAGCATTTAGTCATGAACGCTTCTCTATTCTATATGAAAAGCCGGTTCC
GATAACCTTTTTTGTTATCCAGGAATTTATTATAACAGTTGAAGTTCATAACACTACGTTCGTAAATCAGTACTTGCGAAGAGATAAGATATACTTTTCGGCCAAGG
660 680 700 720 740

GGCGCTCTCACCTTTCCTTTTTCTCCCAATTTTTCAGTTGAAAAAGGTATATGCGTCAGGCGACCTCTGAAATTAACAAAAAATTTCCAGTCATCGAATTTGATTCT
CCGCGAGAGTGGAAAGGAAAAAGAGGGTTAAAAAGTCAACTTTTTCCATATACGCAGTCCGCTGGAGACTTTAATTGTTTTTTAAAGGTCAGTAGCTTAAACTAAGA
760 780 800 820 840

GTGCGATAGCGCCCCTGTGTGTTCTCGTTATGTTGAGGAAAAAAATAATGGTTGCTAAGAGATTCGAACTCTTGCATCTTACGATACCTGAGTATTCCCACAGTTTG
CACGCTATCGCGGGGACACACAAGAGCAATACAACTCCTTTTTTTATTACCAACGATTCTCTAAGCTTGAGAACGTAGAATGCTATGGACTCATAAGGGTGTCAAAC
860 880 900 920 940 960

```
AAAAGCTGTGGTATGGTGCACTCTCAGTACAATCTGCTCTGATGCCGCATAGTTAAGCCAGCCCCGACACCCGCCAACACCCGCTGACGCGCCCTGACGGGCTTGTC
TTTTCGACACCATACCACGTGAGAGTCATGTTAGACGAGACTACGGCGTATCAATTCGGTCGGGGCTGTGGGCGGTTGTGGGCGACTGCGCGGGACTGCCCGAACAG
        980        1,000        1,020        1,040        1,060

TGCTCCCGGCATCCGCTTACAGACAAGCTGTGACCGTCTCCGGGAGCTGCATGTGTCAGAGGTTTTCACCGTCATCACCGAAACGCGCGAGACGAAAGGGCCTCGTG
ACGAGGGCCGTAGGCGAATGTCTGTTCGACACTGGCAGAGGCCCTCGACGTACACAGTCTCCAAAAGTGGCAGTAGTGGCTTTGCGCGCTCTGCTTTCCCGGAGCAC
    1,080        1,100        1,120        1,140        1,160

ATACGCCTATTTTTATAGGTTAATGTCATGATAATAATGGTTTCTTAGTAgatcgcttgcctgtaacttacacgcgcctcgtatcttttaatgatggaataatttgg
TATGCGGATAAAAATATCCAATTACAGTACTATTATTACCAAAGAATCATctagcgaacggacattgaatgtgcgcggagcatagaaaattactaccttattaaacc
                                                  [CEN/ARS >>
1,180        1,200        1,220        1,240        1,260        1,280

gaatttactctgtgtttatttatttttatgttttgtatttggattttagaaagtaaataaagaaggtagaagagttacggaatgaagaaaaaaaaataaacaaaggt
cttaaatgagacacaaataaataaaaatacaaaacataaacctaaaatctttcatttatttcttccatcttctcaatgccttacttctttttttttatttgtttcca
>> CEN/ARS >>
      1,300        1,320        1,340        1,360        1,380

ttaaaaaatttcaacaaaaagcgtactttacatatatatttattagacaagaaaagcagattaaatagatatacattcgattaacgataagtaaaatgtaaaatcac
aatttttttaaagttgtttttcgcatgaaatgtatatataaataatctgttcttttcgtctaatttatctatatgtaagctaattgctattcattttacattttagtg
>> CEN/ARS >>
   1,400        1,420        1,440        1,460        1,480

aggattttcgtgtgtggtcttctacacagacaagatgaaacaattcggcattaatacctgagagcaggaagagcaagataaaaggtagtatttgttggcgatccccc
tcctaaaagcacacaccagaagatgtgtctgttctactttgttaagccgtaattatggactctcgtccttctcgttctattttccatcataaacaaccgctaggggg
>> CEN/ARS >>
1,500        1,520        1,540        1,560        1,580        1,600

tagagtcttttacatcttcggaaaacaaaaactatttttttctttaatttctttttttactttctatttttaatttatatatttatattaaaaaatttaaattataat
atctcagaaaatgtagaagccttttgtttttgataaaaaagaaattaaagaaaaaaatgaaagataaaaattaaatatataaatataatttttttaaatttaatatta
>> CEN/ARS >>
      1,620        1,640        1,660        1,680        1,700

tatttttatagcacgtgatGTTCAGGTGGCACTTTTCGGGGAAATGTGCGCGGAACCCCTATTTGTTTATTTTTCTAAATACATTCAAATATGTATCCGCTCATGAG
ataaaaatatcgtgcactaCAAGTCCACCGTGAAAAGCCCCTTTACACGCGCCTTGGGGATAAACAAATAAAAAGATTTATGTAAGTTTATACATAGGCGAGTACTC
>> CEN/ARS]
   1,720        1,740        1,760        1,780        1,800

ACAATAACCCTGATAAATGCTTCAATAATATTGAAAAAGGAAGAGTATGAGTATTCAACATTTCCGTGTCGCCCTTATTCCCTTTTTTGCGGCATTTTGCCTTCCTG
TGTTATTGGGACTATTTACGAAGTTATTATAACTTTTTCCTTCTCATACTCATAAGTTGTAAAGGCACAGCGGGAATAAGGGAAAAAACGCCGTAAAACGGAAGGAC
1,820        1,840        1,860        1,880        1,900        1,920
```

TTTTTGCTCACCCAGAAACGCTGGTGAAAGTAAAAGATGCTGAAGATCAGTTGGGTGCACGAGTGGGTTACATCGAACTGGATCTCAACAGCGGTAAGATCCTTGAG
AAAAACGAGTGGGTCTTTGCGACCACTTTCATTTTCTACGACTTCTAGTCAACCCACGTGCTCACCCAATGTAGCTTGACCTAGAGTTGTCGCCATTCTAGGAACTC

1,940 1,960 1,980 2,000 2,020

AGTTTTCGCCCCGAAGAACGTTTTCCAATGATGAGCACTTTTAAAGTTCTGCTATGTGGCGCGGTATTATCCCGTATTGACGCCGGGCAAGAGCAACTCGGTCGCCG
TCAAAAGCGGGGCTTCTTGCAAAAGGTTACTACTCGTGAAAATTTCAAGACGATACACCGCGCCATAATAGGGCATAACTGCGGCCCGTTCTCGTTGAGCCAGCGGC

AmpR

2,040 2,060 2,080 2,100 2,120 2,140

CATACACTATTCTCAGAATGACTTGGTTGAGTACTCACCAGTCACAGAAAAGCATCTTACGGATGGCATGACAGTAAGAGAATTATGCAGTGCTGCCATAACCATGA
GTATGTGATAAGAGTCTTACTGAACCAACTCATGAGTGGTCAGTGTCTTTTCGTAGAATGCCTACCGTACTGTCATTCTCTTAATACGTCACGACGGTATTGGTACT

AmpR

2,160 2,180 2,200 2,220 2,240

GTGATAACACTGCGGCCAACTTACTTCTGACAACGATCGGAGGACCGAAGGAGCTAACCGCTTTTTTGCACAACATGGGGGATCATGTAACTCGCCTTGATCGTTGG
CACTATTGTGACGCCGGTTGAATGAAGACTGTTGCTAGCCTCCTGGCTTCCTCGATTGGCGAAAAAACGTGTTGTACCCCCTAGTACATTGAGCGGAACTAGCAACC

AmpR

2,260 2,280 2,300 2,320 2,340

GAACCGGAGCTGAATGAAGCCATACCAAACGACGAGCGTGACACCACGATGCCTGTAGCAATGGCAACAACGTTGCGCAAACTATTAACTGGCGAACTACTTACTCT
CTTGGCCTCGACTTACTTCGGTATGGTTTGCTGCTCGCACTGTGGTGCTACGGACATCGTTACCGTTGTTGCAACGCGTTTGATAATTGACCGCTTGATGAATGAGA

AmpR

2,360 2,380 2,400 2,420 2,440 2,460

AGCTTCCCGGCAACAATTAATAGACTGGATGGAGGCGGATAAAGTTGCAGGACCACTTCTGCGCTCGGCCCTTCCGGCTGGCTGGTTTATTGCTGATAAATCTGGAG
TCGAAGGGCCGTTGTTAATTATCTGACCTACCTCCGCCTATTTCAACGTCCTGGTGAAGACGCGAGCCGGGAAGGCCGACCGACCAAATAACGACTATTTAGACCTC

AmpR

2,480 2,500 2,520 2,540 2,560

CCGGTGAGCGTGGGTCTCGCGGTATCATTGCAGCACTGGGGCCAGATGGTAAGCCCTCCCGTATCGTAGTTATCTACACGACGGGGAGTCAGGCAACTATGGATGAA
GGCCACTCGCACCCAGAGCGCCATAGTAACGTCGTGACCCCGGTCTACCATTCGGGAGGGCATAGCATCAATAGATGTGCTGCCCCTCAGTCCGTTGATACCTACTT

AmpR

2,580 2,600 2,620 2,640 2,660

CGAAATAGACAGATCGCTGAGATAGGTGCCTCACTGATTAAGCATTGGTAACTGTCAGACCAAGTTTACTCATATATACTTTAGATTGATTTAAAACTTCATTTTTA
GCTTTATCTGTCTAGCGACTCTATCCACGGAGTGACTAATTCGTAACCATTGACAGTCTGGTTCAAATGAGTATATATGAAATCTAACTAAATTTTGAAGTAAAAAT

AmpR

2,680 2,700 2,720 2,740 2,760 2,780

ATTTAAAAGGATCTAGGTGAAGATCCTTTTTGATAATCTCATGACCAAAATCCCTTAACGTGAGTTTTCGTTCCACTGAGCGTCAGACCCCGTAGAAAAGATCAAAG
TAAATTTTCCTAGATCCACTTCTAGGAAAAACTATTAGAGTACTGGTTTTAGGGAATTGCACTCAAAAGCAAGGTGACTCGCAGTCTGGGGCATCTTTTCTAGTTTC

2,800 2,820 2,840 2,860 2,880

```
GATCTTCTTGAGATCCTTTTTTTCTGCGCGTAATCTGCTGCTTGCAAACAAAAAAACCACCGCTACCAGCGGTGGTTTGTTTGCCGGATCAAGAGCTACCAACTCTT
CTAGAAGAACTCTAGGAAAAAAAGACGCGCATTAGACGACGAACGTTTGTTTTTTTGGTGGCGATGGTCGCCACCAAACAAACGGCCTAGTTCTCGATGGTTGAGAA
      2,900               2,920               2,940               2,960               2,980

TTTCCGAAGGTAACTGGCTTCAGCAGAGCGCAGATACCAAATACTGTTCTTCTAGTGTAGCCGTAGTTAGGCCACCACTTCAAGAACTCTGTAGCACCGCCTACATA
AAAGGCTTCCATTGACCGAAGTCGTCTCGCGTCTATGGTTTATGACAAGAAGATCACATCGGCATCAATCCGGTGGTGAAGTTCTTGAGACATCGTGGCGGATGTAT
3,000               3,020               3,040               3,060               3,080               3,100

CCTCGCTCTGCTAATCCTGTTACCAGTGGCTGCTGCCAGTGGCGATAAGTCGTGTCTTACCGGGTTGGACTCAAGACGATAGTTACCGGATAAGGCGCAGCGGTCGG
GGAGCGAGACGATTAGGACAATGGTCACCGACGACGGTCACCGCTATTCAGCACAGAATGGCCCAACCTGAGTTCTGCTATCAATGGCCTATTCCGCGTCGCCAGCC
             3,120               3,140               3,160               3,180               3,200

GCTGAACGGGGGGTTCGTGCACACAGCCCAGCTTGGAGCGAACGACCTACACCGAACTGAGATACCTACAGCGTGAGCTATGAGAAAGCGCCACGCTTCCCGAAGGG
CGACTTGCCCCCCAAGCACGTGTGTCGGGTCGAACCTCGCTTGCTGGATGTGGCTTGACTCTATGGATGTCGCACTCGATACTCTTTCGCGGTGCGAAGGGCTTCCC
      3,220               3,240               3,260               3,280               3,300

AGAAAGGCGGACAGGTATCCGGTAAGCGGCAGGGTCGGAACAGGAGAGCGCACGAGGGAGCTTCCAGGGGGAAACGCCTGGTATCTTTATAGTCCTGTCGGGTTTCG
TCTTTCCGCCTGTCCATAGGCCATTCGCCGTCCCAGCCTTGTCCTCTCGCGTGCTCCCTCGAAGGTCCCCCTTTGCGGACCATAGAAATATCAGGACAGCCCAAAGC
3,320               3,340               3,360               3,380               3,400               3,420

CCACCTCTGACTTGAGCGTCGATTTTTGTGATGCTCGTCAGGGGGGCGGAGCCTATGGAAAAACGCCAGCAACGCGGCCTTTTTACGGTTCCTGGCCTTTTGCTGGC
GGTGGAGACTGAACTCGCAGCTAAAAACACTACGAGCAGTCCCCCCGCCTCGGATACCTTTTTGCGGTCGTTGCGCCGGAAAAATGCCAAGGACCGGAAAACGACCG
            3,440               3,460               3,480               3,500               3,520

CTTTTGCTCACATGTTCTTTCCTGCGTTATCCCCTGATTCTGTGGATAACCGTATTACCGCCTTTGAGTGAGCTGATACCGCTCGCCGCAGCCGAACGACCGAGCGC
GAAAACGAGTGTACAAGAAAGGACGCAATAGGGGACTAAGACACCTATTGGCATAATGGCGGAAACTCACTCGACTATGGCGAGCGGCGTCGGCTTGCTGGCTCGCG
     3,540               3,560               3,580               3,600               3,620

AGCGAGTCAGTGAGCGAGGAAGCGGAAGAGCGCCCAATACGCAAACCGCCTCTCCCCGCGCGTTGGCCGATTCATTAATGCAGCTGGCACGACAGGTTTCCCGACTG
TCGCTCAGTCACTCGCTCCTTCGCCTTCTCGCGGGTTATGCGTTTGGCGGAGAGGGGCGCGCAACCGGCTAAGTAATTACGTCGACCGTGCTGTCCAAAGGGCTGAC
3,640               3,660               3,680               3,700               3,720               3,740

GAAAGCGGCCAGTGAGCGCGCGCAAATTAAAGCCTTCGAGCGTCCCAAAACGCAAACCGCCTCTCCCCGCGCGTTGGCCGATTCATTAATGCAGCTGGCACGACAGG
CTTTCGCCGGTCACTCGCGCGCGTTTAATTTCGGAAGCTCGCAGGGTTTTGCGTTTGGCGGAGAGGGGCGCGCAACCGGCTAAGTAATTACGTCGACCGTGCTGTCC
           3,760               3,780               3,800               3,820               3,840

TTTCCCGACTGGAAAGCGGGCAGTGAGCGCAACGCAATTAATGTGAGTTAGCTCACTCATTAGGCACCCCAGGCTTTACACTTTATGCTTCCGGCTCGTATGTTGTG
AAAGGGCTGACCTTTCGCCCGTCACTCGCGTTGCGTTAATTACACTCAATCGAGTGAGTAATCCGTGGGGTCCGAAATGTGAAATACGAAGGCCGAGCATACAACAC
                                                                                    lac promoter
    3,860               3,880               3,900               3,920               3,940
```

TGGAATTGTGAGCGGATAACAATTTCACACAGGAAACAGCTATGACCATGATTACGCCAAGCGCGCAATTAACCCTCACTAAAGGGAACAAAAGCTGGAGCTcAGTT
ACCTTAACACTCGCCTATTGTTAAAGTGTGTCCTTTGTCGATACTGGTACTAATGCGGTTCGCGCGTTAATTGGGAGTGATTTCCCTTGTTTTCGACCTCGAgTCAA

T3
LacO M13-rev T3 promoter

3,960 3,980 4,000 4,020 4,040 4,060

TATCATTATCAATACTcGCCATTTCAAAGAATACGTAAATAATTAATAGTAGTGATTTTCCTAACTTTATTTAGTCAAAAAATTAGCCTTTTAATTCTGCTGTAACC
ATAGTAATAGTTATGAgCGGTAAAGTTTCTTATGCATTTATTAATTATCATCACTAAAAGGATTGAAATAAATCAGTTTTTTAATCGGAAAATTAAGACGACATTGG

GPD promoter

4,080 4,100 4,120 4,140 4,160

CGTACATGCCCAAAATAGGGGGCGGGTTACACAGAATATATAACATCGTAGGTGTCTGGGTGAACAGTTTATTCCTGGCATCCACTAAATATAATGGAGCCCGCTTT
GCATGTACGGGTTTTATCCCCCGCCCAATGTGTCTTATATATTGTAGCATCCACAGACCCACTTGTCAAATAAGGACCGTAGGTGATTTATATTACCTCGGGCGAAA

GPD promoter

4,180 4,200 4,220 4,240 4,260 4,280

TTAAGCTGGCATCCAGAAAAAAAAAGAATCCCAGCACCAAAATATTGTTTTCTTCACCAACCATCAGTTCATAGGTCCATTCTCTTAGCGCAACTACAGAGAACAGG
AATTCGACCGTAGGTCTTTTTTTTTCTTAGGGTCGTGGTTTTATAACAAAAGAAGTGGTTGGTAGTCAAGTATCCAGGTAAGAGAATCGCGTTGATGTCTCTTGTCC

GPD promoter

4,300 4,320 4,340 4,360 4,380

GGCACAAACAGGCAAAAAACGGGCACAACCTCAATGGAGTGATGCAACCTGCCTGGAGTAAATGATGACACAAGGCAATTGACCCACGCATGTATCTATCTCATTTT
CCGTGTTTGTCCGTTTTTTGCCCGTGTTGGAGTTACCTCACTACGTTGGACGGACCTCATTTACTACTGTGTTCCGTTAACTGGGTGCGTACATAGATAGAGTAAAA

GPD promoter

4,400 4,420 4,440 4,460 4,480

CTTACACCTTCTATTACCTTCTGCTCTCTCTGATTTGGAAAAAGCTGAAAAAAAAGGTTGAAACCAGTTCCCTGAAATTATTCCCCTACTTGACTAATAAGTATATA
GAATGTGGAAGATAATGGAAGACGAGAGAGACTAAACCTTTTTCGACTTTTTTTTCCAACTTTGGTCAAGGGACTTTAATAAGGGGATGAACTGATTATTCATATAT

GPD promoter

4,500 4,520 4,540 4,560 4,580 4,600

AAGACGGTAGGTATTGATTGTAATTCTGTAAATCTATTTCTTAAACTTCTTAAATTCTACTTTTATAGTTAGTCTTTTTTTTAGTTTTAAAACACCAGAACTTAGTT
TTCTGCCATCCATAACTAACATTAAGACATTTAGATAAAGAATTTGAAGAATTTAAGATGAAAATATCAATCAGAAAAAAAATCAAAATTTTGTGGTCTTGAATCAA

GPD promoter

4,620 4,640 4,660 4,680 4,700

TCGACGGATTCTAGAACTAGTGGATCCATGAGATTCCCATCTATCTTCACCGCTGTTTTGTTCGCTGCTTCTTCTGCTTTGGCTGCTCCAGCTAACACCACCACCGA
AGCTGCCTAAGATCTTGATCACCTAGGTACTCTAAGGGTAGATAGAAGTGGCGACAAAACAAGCGACGAAGAAGACGAAACCGACGAGGTCGATTGTGGTGGTGGCT

1 2 3 4 5 6 7 8 9 10 11 12 13 14 15 16 17 18 19 20 21 22 23 24 25 26 27
M R F P S I F T A V L F A A S S A L A A P A N T T T E

GPD promoter Mating factor alpha leader

4,720 4,740 4,760 4,780 4,800

```
AGACGAAACCGCTCAAATCCCAGCTGAAGCTGTTATCGACTACTCTGACTTGGAAGGTGACTTCGACGCTGCTGCTTTGCCATTGTCTAACTCTACCAACAACGGTT
TCTGCTTTGGCGAGTTTAGGGTCGACTTCGACAATAGCTGATGAGACTGAACCTTCCACTGAAGCTGCGACGACGAAACGGTAACAGATTGAGATGGTTGTTGCCAA
 28 29 30 31 32 33 34 35 36 37 38 39 40 41 42 43 44 45 46 47 48 49 50 51 52 53 54 55 56 57 58 59 60 61 62
  D  E  T  A  Q  I  P  A  E  A  V  I  D  Y  S  D  L  E  G  D  F  D  A  A  A  L  P  L  S  N  S  T  N  N  G
Mating factor alpha leader
4,820 4,840 4,860 4,880 4,900 4,920
```

```
TGTCTTCTACCAACACCACCATCGCTTCTATCGCTGCTAAGGAAGAAGGTGTTCAATTGGACAAGAGAGAAagatctggcggccgcgcatgcgctagcctcgagCAA
ACAGAAGATGGTTGTGGTGGTAGCGAAGATAGCGACGATTCCTTCTTCCACAAGTTAACCTGTTCTCTCTTtctagaccgccggcgcgtacgcgatcggagctcGTT
63 64 65 66 67 68 69 70 71 72 73 74 75 76 77 78 79 80 81 82 83 84 85 86
 L  S  S  T  N  T  T  I  A  S  I  A  A  K  E  E  G  V  Q  L  D  K  R  E                              Q
Mating factor alpha leader
4,940 4,960 4,980 5,000 5,020
```

```
GTCCAGCTGGTCGAGTCAGGTGGTGGATCTGTCCAAGCCGGGGGGTCTCTAAGACTATCATGTACGGCGTCAGGCAATATTTTTTACGTTCTGGACATGTGGTTCAG
CAGGTCGACCAGCTCAGTCCACCACCTAGACAGGTTCGGCCCCCCAGAGATTCTGATAGTACATGCCGCAGTCCGTTATAAAAAATGCAAGACCTGTACACCAAGTC
 2     4     6     8     10    12    14    16    18    20    22    24    26    28    30    32    34    36
 V  Q  L  V  E  S  G  G  G  S  V  Q  A  G  G  S  L  R  L  S  C  T  A  S  G  N  I  F  Y  V  L  D  M  W  F  R
Syn Nb_b
5,040 5,060 5,080 5,100 5,120
```

```
GCAAGCACCGGGCCAGGAAAGAGAGGCAGTTGCGGAATTTGTTGCCGCTATTGCTGGTACTGGTGGTACTACCTATTATCGTTTTACTATTAGCAGAGACAACGCAA
CGTTCGTGGCCCGGTCCTTTCTCTCCGTCAACGCCTTAAACAACGGCGATAACGACCATGACCACCATGATGGATAATAGCAAAATGATAATCGTCTCTGTTGCGTT
 38    40    42    44    46    48    50    52    54    56    58    60    62    64    66    68    70    72
 Q  A  P  G  Q  E  R  E  A  V  A  E  F  V  A  A  I  A  G  T  G  G  T  T  Y  Y  R  F  T  I  S  R  D  N  A
Syn Nb_b
5,140 5,160 5,180 5,200 5,220 5,240
```

```
AAAATACTGTTACGCTACAAATGAATAATCTGAAGCCAGAAGATACTGCGATCTATTATTGTGCCGCAGCGGCTTCTTACACTCTGGGTACTTACGACCAGACTTTT
TTTTATGACAATGCGATGTTTACTTATTAGACTTCGGTCTTCTATGACGCTAGATAATAACACGGCGTCGCCGAAGAATGTGAGACCCATGAATGCTGGTCTGAAAA
   74    76    78    80    82    84    86    88    90    92    94    96    98    100   102   104   106   108
 K  N  T  V  T  L  Q  M  N  N  L  K  P  E  D  T  A  I  Y  Y  C  A  A  A  A  S  Y  T  L  G  T  Y  D  Q  T  F
Syn Nb_b
5,260 5,280 5,300 5,320 5,340
```

```
TCTTATTGGGGACAAGGAACACAGGTCACAGTATCATCTGGCGGAGGAGGTTCCGGTGGAGGGGGGTCAGACTATAAGGATGACGACGATAAGGCTGGAGGTTCGAG
AGAATAACCCCTGTTCCTTGTGTCCAGTGTCATAGTAGACCGCCTCCTCCAAGGCCACCTCCCCCCAGTCTGATATTCCTACTGCTGCTATTCCGACCTCCAAGCTC
   110   112   114   116   118   120   1  2  3  4  5  6  7  8  9  10 1  2  3  4  5  6  7  8  1  2  3  4  1
 S  Y  W  G  Q  G  T  Q  V  T  V  S  S  G  G  G  G  S  G  G  G  G  S  D  Y  K  D  D  D  D  K  A  G  G  S  R
Syn Nb_b                                    FLAG tag
5,360 5,380 5,400 5,420 5,440
```

GGGAGTCCCTCACATAGTGATGGTGGATGCTTACAAAAGGTATAAATAACCCGGGCTGCAGGAATTCGATATCAAGCTTATCGATACCGTCGACCTCGAGTCATGTA
CCCTCAGGGAGTGTATCACTACCACCTACGAATGTTTTCCATATTTATTGGGCCCGACGTCCTTAAGCTATAGTTCGAATAGCTATGGCAGCTGGAGCTCAGTACAT

2 3 4 5 6 7 8 9 10 11 12 13 14 15 16 1
G V P H I V M V D A Y K R Y K *

SpyTag

5,460 5,480 5,500 5,520 5,540 5,560

ATTAGTTATGTCACGCTTACGTTCACGCCCTCCCCCCACATCCGCTCTAACCGAAAAGGAAGGAGTTAGACAACCTGAAGTCTAGGTCCCTATTTATTTTTTTATAG
TAATCAATACAGTGCGAATGCAAGTGCGGGAGGGGGGTGTAGGCGAGATTGGCTTTTCCTTCCTCAATCTGTTGGACTTCAGATCCAGGGATAAATAAAAAAATATC

CYC1 terminator

5,580 5,600 5,620 5,640 5,660

TTATGTTAGTATTAAGAACGTTATTTATATTTCAAATTTTTCTTTTTTTTCTGTACAGACGCGTGTACGCATGTAACATTATACTGAAAACCTTGCTTGAGAAGGTT
AATACAATCATAATTCTTGCAATAAATATAAAGTTTAAAAAGAAAAAAAAGACATGTCTGCGCACATGCGTACATTGTAATATGACTTTTGGAACGAACTCTTCCAA

CYC1 terminator

5,680 5,700 5,720 5,740 5,760

TTGGGACGCTCGAAGGCTTTAATTTGCGCGCGCTCACTGGCCGTCGTTTTACAACGTCGTGACTGGGAAAACCCTGGCGTTACCCAACTTAATCGCCTTGCAGCACA
AACCCTGCGAGCTTCCGAAATTAAACGCGCGCGAGTGACCGGCAGCAAAATGTTGCAGCACTGACCCTTTTGGGACCGCAATGGGTTGAATTAGCGGAACGTCGTGT

CYC1 terminator M13-fwd

5,780 5,800 5,820 5,840 5,860 5,880

TCCCCCTTTCGCCAGCTGGCGTAATAGCGAAGAGGCCCGCACCGATCGCCCTTCCCAACAGTTGCGCAGCCTGAATGGCGAATGGACGCGCCCTGTAGCGGCGCATT
AGGGGGAAAGCGGTCGACCGCATTATCGCTTCTCCGGGCGTGGCTAGCGGGAAGGGTTGTCAACGCGTCGGACTTACCGCTTACCTGCGCGGGACATCGCCGCGTAA

LacZ alpha

5,900 5,920 5,940 5,960 5,980

AAGCGCGGCGGGTGTGGTGGTTACGCGCAGCGTGACCACTACACTTGCCAGCGCCCTAGCGCCCGCTCCTTTCGCTTTCTTCCCTTCCTTTCTCGCCACGTTCGCCG
TTCGCGCCGCCCACACCACCAATGCGCGTCGCACTGGTGATGTGAACGGTCGCGGGATCGCGGGCGAGGAAAGCGAAAGAAGGGAAGGAAAGAGCGGTGCAAGCGGC

6,000 6,020 6,040 6,060 6,080

GCTTTCCCCGTCAAGCTCTAAATCGGGGGCTCCCTTTAGGGTTCCGATTTAGTGCTTTACGGCACCTCGACCCCAAAAAACTTGATTAGGGTGATGGTTCACGTAGT
CGAAAGGGGCAGTTCGAGATTTAGCCCCCGAGGGAAATCCCAAGGCTAAATCACGAAATGCCGTGGAGCTGGGGTTTTTTGAACTAATCCCACTACCAAGTGCATCA

6,100 6,120 6,140 6,160 6,180 6,200

GGGCCATCGCCCTGATAGACGGTTTTTCGCCCTTTGACGTTGGAGTCCACGTTCTTTAATAGTGGACTCTTGTTCCAAACTGGAACAACACTCAACCCTATCTCGGT
CCCGGTAGCGGGACTATCTGCCAAAAAGCGGGAAACTGCAACCTCAGGTGCAAGAAATTATCACCTGAGAACAAGGTTTGACCTTGTTGTGAGTTGGGATAGAGCCA

6,220 6,240 6,260 6,280 6,300

CTATTCTTTTGATTTATAAGGGATTTTGCCGATTTCGGCCTATTGGTTAAAAAATGAGCTGATTTAACAAAAATTTAACGCGAATTTTAACAAAATATTAACGCTTA
GATAAGAAAACTAAATATTCCCTAAAACGGCTAAAGCCGGATAACCAATTTTTTACTCGACTAAATTGTTTTTAAATTGCGCTTAAAATTGTTTTATAATTGCGAAT

6,320 6,340 6,360 6,380 6,400 6,420

CAATTTCCTGATGCGGTATTTTCTCCTTACGCATCTGTGCGGTATTTCACACCGCATAGGGTAATAACTGATATAATTAAATTGAAGCTCTAATTTGTGAGTTTAGT
GTTAAAGGACTACGCCATAAAAGAGGAATGCGTAGACACGCCATAAAGTGTGGCGTATCCCATTATTGACTATATTAATTTAACTTCGAGATTAAACACTCAAATCA

6,440 6,460 6,480 6,500 6,520

ATACATGCATTTACTTATAATACAGTTTTTTAAGCAAGGATTTTCTTAACTTCTTCGGCGACAGCATCACCGACTTCGGTGGTACTGTTGGAACCACCTAAATCACC
TATGTACGTAAATGAATATTATGTCAAAAAATTCGTTCCTAAAAGAATTGAAGAAGCCGCTGTCGTAGTGGCTGAAGCCACCATGACAACCTTGGTGGATTTAGTGG

LEU2

6,540 6,560 6,580 6,600 6,620

AGTTCTGATACCTGCATCCAAAACCTTTTTAACTGCATCTTCAATGGCCTTACCTTCTTCAGGCAAGTTCAATGACAATTTCAACATCATTGCAGCAGACAAGATAG
TCAAGACTATGGACGTAGGTTTTGGAAAAATTGACGTAGAAGTTACCGGAATGGAAGAAGTCCGTTCAAGTTACTGTTAAAGTTGTAGTAACGTCGTCTGTTCTATC

LEU2

6,640 6,660 6,680 6,700 6,720 6,740

TGGCGATAGGGTTGACCTTATTCTTTGGCAAATCTGGAGCAGAACCGTGGCATGGTTCGTACAAACCAAATGCGGTGTTCTTGTCTGGCAAAGAGGCCAAGGACGCA
ACCGCTATCCCAACTGGAATAAGAAACCGTTTAGACCTCGTCTTGGCACCGTACCAAGCATGTTTGGTTTACGCCACAAGAACAGACCGTTTCTCCGGTTCCTGCGT

LEU2

6,760 6,780 6,800 6,820 6,840

GATGGCAACAAACCCAAGGAACCTGGGATAACGGAGGCTTCATCGGAGATGATATCACCAAACATGTTGCTGGTGATTATAATACCATTTAGGTGGGTTGGGTTCTT
CTACCGTTGTTTGGGTTCCTTGGACCCTATTGCCTCCGAAGTAGCCTCTACTATAGTGGTTTGTACAACGACCACTAATATTATGGTAAATCCACCCAACCCAAGAA

LEU2

6,860 6,880 6,900 6,920 6,940

AACTAGGATCATGGCGGCAGAATCAATCAATTGATGTTGAACCTTCAATGTAGGGAATTCGTTCTTGATGGTTTCCTCCACAGTTTTTCTCCATAATCTTGAAGAGG
TTGATCCTAGTACCGCCGTCTTAGTTAGTTAACTACAACTTGGAAGTTACATCCCTTAAGCAAGAACTACCAAAGGAGGTGTCAAAAAGAGGTATTAGAACTTCTCC

LEU2

6,960 6,980 7,000 7,020 7,040 7,060

CCAAAACATTAGCTTTATCCAAGGACCAAATAGGCAAT
GGTTTTGTAATCGAAATAGGTTCCTGGTTTATCCGTTA

LEU2

7,070 7,080 7,090 7,100

ST/SC ligation / pNb_Syn Nb_a-FLAG (pRS415) (71...

GGTGGCTCATGTTGTAGGGCCATGAAAGCGGCCATTCTTGTGATTCTTTGCACTTCTGGAACGGTGTATTGTTCACTATCCCAAGCGACACCATCACCATCGTCTTC
CCACCGAGTACAACATCCCGGTACTTTCGCCGGTAAGAACACTAAGAAACGTGAAGACCTTGCCACATAACAAGTGATAGGGTTCGCTGTGGTAGTGGTAGCAGAAG
LEU2
20 40 60 80 100

CTTTCTCTTACCAAAGTAAATACCTCCCACTAATTCTCTGACAACAACGAAGTCAGTACCTTTAGCAAATTGTGGCTTGATTGGAGATAAGTCTAAAAGAGAGTCGG
GAAAGAGAATGGTTTCATTTATGGAGGGTGATTAAGAGACTGTTGTTGCTTCAGTCATGGAAATCGTTTAACACCGAACTAACCTCTATTCAGATTTTCTCTCAGCC
LEU2
120 140 160 180 200

ATGCAAAGTTACATGGTCTTAAGTTGGCGTACAATTGAAGTTCTTTACGGATTTTTAGTAAACCTTGTTCAGGTCTAACACTACCGGTACCCCATTTAGGACCACCC
TACGTTTCAATGTACCAGAATTCAACCGCATGTTAACTTCAAGAAATGCCTAAAAATCATTTGGAACAAGTCCAGATTGTGATGGCCATGGGGTAAATCCTGGTGGG
LEU2
220 240 260 280 300 320

ACAGCACCTAACAAAACGGCATCAGCCTTCTTGGAGGCTTCCAGCGCCTCATCTGGAAGTGGAACACCTGTAGCATCGATAGCAGCACCACCAATTAAATGATTTTC
TGTCGTGGATTGTTTTGCCGTAGTCGGAAGAACCTCCGAAGGTCGCGGAGTAGACCTTCACCTTGTGGACATCGTAGCTATCGTCGTGGTGGTTAATTTACTAAAAG
LEU2
340 360 380 400 420

GAAATCGAACTTGACATTGGAACGAACATCAGAAATAGCTTTAAGAACCTTAATGGCTTCGGCTGTGATTTCTTGACCAACGTGGTCACCTGGCAAAACGACGATCT
CTTTAGCTTGAACTGTAACCTTGCTTGTAGTCTTTATCGAAATTCTTGGAATTACCGAAGCCGACACTAAAGAACTGGTTGCACCAGTGGACCGTTTTGCTGCTAGA
LEU2
440 460 480 500 520

TCTTAGGGGCAGACATTAGAATGGTATATCCTTGAAATATATATATATATATATNTNGCTGAAATGTAAAAGGTAAGAAAAGTTAGAAAGTAAGACGATTGCTAACCAC
AGAATCCCCGTCTGTAATCTTACCATATAGGAACTTTATATATATATATATATANANCGACTTTACATTTTCCATTCTTTTCAATCTTTCATTCTGCTAACGATTGGTG
LEU2
540 560 580 600 620 640

CTATTGGAAAAAACAATAGGTCCTTAAATAATATTGTCAACTTCAAGTATTGTGATGCAAGCATTTAGTCATGAACGCTTCTCTATTCTATATGAAAAGCCGGTTCC
GATAACCTTTTTTGTTATCCAGGAATTTATTATAACAGTTGAAGTTCATAACACTACGTTCGTAAATCAGTACTTGCGAAGAGATAAGATATACTTTTCGGCCAAGG
660 680 700 720 740

GGCGCTCTCACCTTTCCTTTTTCTCCCAATTTTTCAGTTGAAAAAGGTATATGCGTCAGGCGACCTCTGAAATTAACAAAAAATTTCCAGTCATCGAATTTGATTCT
CCGCGAGAGTGGAAAGGAAAAAGAGGGTTAAAAAGTCAACTTTTTCCATATACGCAGTCCGCTGGAGACTTTAATTGTTTTTTAAAGGTCAGTAGCTTAAACTAAGA
760 780 800 820 840

GTGCGATAGCGCCCCTGTGTGTTCTCGTTATGTTGAGGAAAAAAATAATGGTTGCTAAGAGATTCGAACTCTTGCATCTTACGATACCTGAGTATTCCCACAGTTTG
CACGCTATCGCGGGGACACACAAGAGCAATACAACTCCTTTTTTTATTACCAACGATTCTCTAAGCTTGAGAACGTAGAATGCTATGGACTCATAAGGGTGTCAAAC
860 880 900 920 940 960

AAAAGCTGTGGTATGGTGCACTCTCAGTACAATCTGCTCTGATGCCGCATAGTTAAGCCAGCCCCGACACCCGCCAACACCCGCTGACGCGCCCTGACGGGCTTGTC
TTTTCGACACCATACCACGTGAGAGTCATGTTAGACGAGACTACGGCGTATCAATTCGGTCGGGGCTGTGGGCGGTTGTGGGCGACTGCGCGGGACTGCCCGAACAG

980 1,000 1,020 1,040 1,060

TGCTCCCGGCATCCGCTTACAGACAAGCTGTGACCGTCTCCGGGAGCTGCATGTGTCAGAGGTTTTCACCGTCATCACCGAAACGCGCGAGACGAAAGGGCCTCGTG
ACGAGGGCCGTAGGCGAATGTCTGTTCGACACTGGCAGAGGCCCTCGACGTACACAGTCTCCAAAAGTGGCAGTAGTGGCTTTGCGCGCTCTGCTTTCCCGGAGCAC

1,080 1,100 1,120 1,140 1,160

ATACGCCTATTTTTATAGGTTAATGTCATGATAATAATGGTTTCTTAGTAgatcgcttgcctgtaacttacacgcgcctcgtatcttttaatgatggaataatttgg
TATGCGGATAAAAATATCCAATTACAGTACTATTATTACCAAAGAATCATctagcgaacggacattgaatgtgcgcggagcatagaaaattactaccttattaaacc

CEN/ARS

1,180 1,200 1,220 1,240 1,260 1,280

gaatttactctgtgtttatttatttttatgttttgtatttggattttagaaagtaaataaagaaggtagaagagttacggaatgaagaaaaaaaaataaacaaaggt
cttaaatgagacacaaataaataaaaatacaaaacataaacctaaaatctttcatttatttcttccatcttctcaatgccttacttctttttttttatttgtttcca

CEN/ARS

1,300 1,320 1,340 1,360 1,380

ttaaaaaatttcaacaaaaagcgtactttacatatatatttattagacaagaaaagcagattaaatagatatacattcgattaacgataagtaaaatgtaaaatcac
aatttttaaagttgtttttcgcatgaaatgtatatataaataatctgttcttttcgtctaatttatctatatgtaagctaattgctattcattttacattttagtg

CEN/ARS

1,400 1,420 1,440 1,460 1,480

aggattttcgtgtgtggtcttctacacagacaagatgaaacaattcggcattaatacctgagagcaggaagagcaagataaaaggtagtatttgttggcgatccccc
tcctaaaagcacacaccagaagatgtgtctgttctactttgttaagccgtaattatggactctcgtccttctcgttctattttccatcataaacaaccgctaggggg

CEN/ARS

1,500 1,520 1,540 1,560 1,580 1,600

tagagtcttttacatcttcggaaaacaaaaactatttttttctttaatttctttttttactttctatttttaatttatatatttatattaaaaaatttaaattataat
atctcagaaaatgtagaagccttttgtttttgataaaaaagaaattaaagaaaaaaatgaaagataaaaattaaatatataaatataatttttttaaatttaatatta

CEN/ARS

1,620 1,640 1,660 1,680 1,700

tatttttatagcacgtgatGTTCAGGTGGCACTTTTCGGGGAAATGTGCGCGGAACCCCTATTTGTTTATTTTTCTAAATACATTCAAATATGTATCCGCTCATGAG
ataaaaatatcgtgcactaCAAGTCCACCGTGAAAAGCCCCTTTACACGCGCCTTGGGGATAAACAAATAAAAAGATTTATGTAAGTTTATACATAGGCGAGTACTC

CEN/ARS

1,720 1,740 1,760 1,780 1,800

ACAATAACCCTGATAAATGCTTCAATAATATTGAAAAAGGAAGAGTATGAGTATTCAACATTTCCGTGTCGCCCTTATTCCCTTTTTTGCGGCATTTTGCCTTCCTG
TGTTATTGGGACTATTTACGAAGTTATTATAACTTTTTCCTTCTCATACTCATAAGTTGTAAAGGCACAGCGGGAATAAGGGAAAAAACGCCGTAAAACGGAAGGAC

1,820 1,840 1,860 1,880 1,900 1,920

TTTTTGCTCACCCAGAAACGCTGGTGAAAGTAAAAGATGCTGAAGATCAGTTGGGTGCACGAGTGGGTTACATCGAACTGGATCTCAACAGCGGTAAGATCCTTGAG
AAAAACGAGTGGGTCTTTGCGACCACTTTCATTTTCTACGACTTCTAGTCAACCCACGTGCTCACCCAATGTAGCTTGACCTAGAGTTGTCGCCATTCTAGGAACTC

1,940 1,960 1,980 2,000 2,020

AGTTTTCGCCCCGAAGAACGTTTTCCAATGATGAGCACTTTTAAAGTTCTGCTATGTGGCGCGGTATTATCCCGTATTGACGCCGGGCAAGAGCAACTCGGTCGCCG
TCAAAAGCGGGGCTTCTTGCAAAAGGTTACTACTCGTGAAAATTTCAAGACGATACACCGCGCCATAATAGGGCATAACTGCGGCCCGTTCTCGTTGAGCCAGCGGC

AmpR

2,040 2,060 2,080 2,100 2,120 2,140

CATACACTATTCTCAGAATGACTTGGTTGAGTACTCACCAGTCACAGAAAAGCATCTTACGGATGGCATGACAGTAAGAGAATTATGCAGTGCTGCCATAACCATGA
GTATGTGATAAGAGTCTTACTGAACCAACTCATGAGTGGTCAGTGTCTTTTCGTAGAATGCCTACCGTACTGTCATTCTCTTAATACGTCACGACGGTATTGGTACT

AmpR

2,160 2,180 2,200 2,220 2,240

GTGATAACACTGCGGCCAACTTACTTCTGACAACGATCGGAGGACCGAAGGAGCTAACCGCTTTTTTGCACAACATGGGGGATCATGTAACTCGCCTTGATCGTTGG
CACTATTGTGACGCCGGTTGAATGAAGACTGTTGCTAGCCTCCTGGCTTCCTCGATTGGCGAAAAAACGTGTTGTACCCCCTAGTACATTGAGCGGAACTAGCAACC

AmpR

2,260 2,280 2,300 2,320 2,340

GAACCGGAGCTGAATGAAGCCATACCAAACGACGAGCGTGACACCACGATGCCTGTAGCAATGGCAACAACGTTGCGCAAACTATTAACTGGCGAACTACTTACTCT
CTTGGCCTCGACTTACTTCGGTATGGTTTGCTGCTCGCACTGTGGTGCTACGGACATCGTTACCGTTGTTGCAACGCGTTTGATAATTGACCGCTTGATGAATGAGA

AmpR

2,360 2,380 2,400 2,420 2,440 2,460

AGCTTCCCGGCAACAATTAATAGACTGGATGGAGGCGGATAAAGTTGCAGGACCACTTCTGCGCTCGGCCCTTCCGGCTGGCTGGTTTATTGCTGATAAATCTGGAG
TCGAAGGGCCGTTGTTAATTATCTGACCTACCTCCGCCTATTTCAACGTCCTGGTGAAGACGCGAGCCGGGAAGGCCGACCGACCAAATAACGACTATTTAGACCTC

AmpR

2,480 2,500 2,520 2,540 2,560

CCGGTGAGCGTGGGTCTCGCGGTATCATTGCAGCACTGGGGCCAGATGGTAAGCCCTCCCGTATCGTAGTTATCTACACGACGGGGAGTCAGGCAACTATGGATGAA
GGCCACTCGCACCCAGAGCGCCATAGTAACGTCGTGACCCCGGTCTACCATTCGGGAGGGCATAGCATCAATAGATGTGCTGCCCCTCAGTCCGTTGATACCTACTT

AmpR

2,580 2,600 2,620 2,640 2,660

CGAAATAGACAGATCGCTGAGATAGGTGCCTCACTGATTAAGCATTGGTAACTGTCAGACCAAGTTTACTCATATATACTTTAGATTGATTTAAAACTTCATTTTTA
GCTTTATCTGTCTAGCGACTCTATCCACGGAGTGACTAATTCGTAACCATTGACAGTCTGGTTCAAATGAGTATATATGAAATCTAACTAAATTTTGAAGTAAAAAT

AmpR

2,680 2,700 2,720 2,740 2,760 2,780

ATTTAAAAGGATCTAGGTGAAGATCCTTTTTGATAATCTCATGACCAAAATCCCTTAACGTGAGTTTTCGTTCCACTGAGCGTCAGACCCCGTAGAAAAGATCAAAG
TAAATTTTCCTAGATCCACTTCTAGGAAAAACTATTAGAGTACTGGTTTTAGGGAATTGCACTCAAAAGCAAGGTGACTCGCAGTCTGGGGCATCTTTTCTAGTTTC

2,800 2,820 2,840 2,860 2,880

GATCTTCTTGAGATCCTTTTTTTCTGCGCGTAATCTGCTGCTTGCAAACAAAAAAACCACCGCTACCAGCGGTGGTTTGTTTGCCGGATCAAGAGCTACCAACTCTT
CTAGAAGAACTCTAGGAAAAAAAGACGCGCATTAGACGACGAACGTTTGTTTTTTTGGTGGCGATGGTCGCCACCAAACAAACGGCCTAGTTCTCGATGGTTGAGAA

2,900 2,920 2,940 2,960 2,980

TTTCCGAAGGTAACTGGCTTCAGCAGAGCGCAGATACCAAATACTGTTCTTCTAGTGTAGCCGTAGTTAGGCCACCACTTCAAGAACTCTGTAGCACCGCCTACATA
AAAGGCTTCCATTGACCGAAGTCGTCTCGCGTCTATGGTTTATGACAAGAAGATCACATCGGCATCAATCCGGTGGTGAAGTTCTTGAGACATCGTGGCGGATGTAT

3,000 3,020 3,040 3,060 3,080 3,100

CCTCGCTCTGCTAATCCTGTTACCAGTGGCTGCTGCCAGTGGCGATAAGTCGTGTCTTACCGGGTTGGACTCAAGACGATAGTTACCGGATAAGGCGCAGCGGTCGG
GGAGCGAGACGATTAGGACAATGGTCACCGACGACGGTCACCGCTATTCAGCACAGAATGGCCCAACCTGAGTTCTGCTATCAATGGCCTATTCCGCGTCGCCAGCC

3,120 3,140 3,160 3,180 3,200

GCTGAACGGGGGGTTCGTGCACACAGCCCAGCTTGGAGCGAACGACCTACACCGAACTGAGATACCTACAGCGTGAGCTATGAGAAAGCGCCACGCTTCCCGAAGGG
CGACTTGCCCCCCAAGCACGTGTGTCGGGTCGAACCTCGCTTGCTGGATGTGGCTTGACTCTATGGATGTCGCACTCGATACTCTTTCGCGGTGCGAAGGGCTTCCC

3,220 3,240 3,260 3,280 3,300

AGAAAGGCGGACAGGTATCCGGTAAGCGGCAGGGTCGGAACAGGAGAGCGCACGAGGGAGCTTCCAGGGGGAAACGCCTGGTATCTTTATAGTCCTGTCGGGTTTCG
TCTTTCCGCCTGTCCATAGGCCATTCGCCGTCCCAGCCTTGTCCTCTCGCGTGCTCCCTCGAAGGTCCCCCTTTGCGGACCATAGAAATATCAGGACAGCCCAAAGC

3,320 3,340 3,360 3,380 3,400 3,420

CCACCTCTGACTTGAGCGTCGATTTTTGTGATGCTCGTCAGGGGGGCGGAGCCTATGGAAAAACGCCAGCAACGCGGCCTTTTTACGGTTCCTGGCCTTTTGCTGGC
GGTGGAGACTGAACTCGCAGCTAAAAACACTACGAGCAGTCCCCCCGCCTCGGATACCTTTTTGCGGTCGTTGCGCCGGAAAAATGCCAAGGACCGGAAAACGACCG

3,440 3,460 3,480 3,500 3,520

CTTTTGCTCACATGTTCTTTCCTGCGTTATCCCCTGATTCTGTGGATAACCGTATTACCGCCTTTGAGTGAGCTGATACCGCTCGCCGCAGCCGAACGACCGAGCGC
GAAAACGAGTGTACAAGAAAGGACGCAATAGGGGACTAAGACACCTATTGGCATAATGGCGGAAACTCACTCGACTATGGCGAGCGGCGTCGGCTTGCTGGCTCGCG

3,540 3,560 3,580 3,600 3,620

AGCGAGTCAGTGAGCGAGGAAGCGGAAGAGCGCCCAATACGCAAACCGCCTCTCCCCGCGCGTTGGCCGATTCATTAATGCAGCTGGCACGACAGGTTTCCCGACTG
TCGCTCAGTCACTCGCTCCTTCGCCTTCTCGCGGGTTATGCGTTTGGCGGAGAGGGGCGCGCAACCGGCTAAGTAATTACGTCGACCGTGCTGTCCAAAGGGCTGAC

3,640 3,660 3,680 3,700 3,720 3,740

GAAAGCGGCCAGTGAGCGCGCGCAAATTAAAGCCTTCGAGCGTCCCAAAACGCAAACCGCCTCTCCCCGCGCGTTGGCCGATTCATTAATGCAGCTGGCACGACAGG
CTTTCGCCGGTCACTCGCGCGCGTTTAATTTCGGAAGCTCGCAGGGTTTTGCGTTTGGCGGAGAGGGGCGCGCAACCGGCTAAGTAATTACGTCGACCGTGCTGTCC

3,760 3,780 3,800 3,820 3,840

TTTCCCGACTGGAAAGCGGGCAGTGAGCGCAACGCAATTAATGTGAGTTAGCTCACTCATTAGGCACCCCAGGCTTTACACTTTATGCTTCCGGCTCGTATGTTGTG
AAAGGGCTGACCTTTCGCCCGTCACTCGCGTTGCGTTAATTACACTCAATCGAGTGAGTAATCCGTGGGGTCCGAAATGTGAAATACGAAGGCCGAGCATACAACAC

lac promoter

3,860 3,880 3,900 3,920 3,940

TGGAATTGTGAGCGGATAACAATTTCACACAGGAAACAGCTATGACCATGATTACGCCAAGCGCGCAATTAACCCTCACTAAAGGGAACAAAAGCTGGAGCTcAGTT
ACCTTAACACTCGCCTATTGTTAAAGTGTGTCCTTTGTCGATACTGGTACTAATGCGGTTCGCGCGTTAATTGGGAGTGATTTCCCTTGTTTTCGACCTCGAgTCAA

T3
LacO M13-rev T3 promoter

3,960 3,980 4,000 4,020 4,040 4,060

TATCATTATCAATACTcGCCATTTCAAAGAATACGTAAATAATTAATAGTAGTGATTTTCCTAACTTTATTTAGTCAAAAAATTAGCCTTTTAATTCTGCTGTAACC
ATAGTAATAGTTATGAgCGGTAAAGTTTCTTATGCATTTATTAATTATCATCACTAAAAGGATTGAAATAAATCAGTTTTTTAATCGGAAAATTAAGACGACATTGG

GPD promoter

4,080 4,100 4,120 4,140 4,160

CGTACATGCCCAAAATAGGGGGCGGGTTACACAGAATATATAACATCGTAGGTGTCTGGGTGAACAGTTTATTCCTGGCATCCACTAAATATAATGGAGCCCGCTTT
GCATGTACGGGTTTTATCCCCCGCCCAATGTGTCTTATATATTGTAGCATCCACAGACCCACTTGTCAAATAAGGACCGTAGGTGATTTATATTACCTCGGGCGAAA

GPD promoter

4,180 4,200 4,220 4,240 4,260 4,280

TTAAGCTGGCATCCAGAAAAAAAAAGAATCCCAGCACCAAAATATTGTTTTCTTCACCAACCATCAGTTCATAGGTCCATTCTCTTAGCGCAACTACAGAGAACAGG
AATTCGACCGTAGGTCTTTTTTTTTCTTAGGGTCGTGGTTTTATAACAAAAGAAGTGGTTGGTAGTCAAGTATCCAGGTAAGAGAATCGCGTTGATGTCTCTTGTCC

GPD promoter

4,300 4,320 4,340 4,360 4,380

GGCACAAACAGGCAAAAAACGGGCACAACCTCAATGGAGTGATGCAACCTGCCTGGAGTAAATGATGACACAAGGCAATTGACCCACGCATGTATCTATCTCATTTT
CCGTGTTTGTCCGTTTTTTGCCCGTGTTGGAGTTACCTCACTACGTTGGACGGACCTCATTTACTACTGTGTTCCGTTAACTGGGTGCGTACATAGATAGAGTAAAA

GPD promoter

4,400 4,420 4,440 4,460 4,480

CTTACACCTTCTATTACCTTCTGCTCTCTCTGATTTGGAAAAAGCTGAAAAAAAAAGGTTGAAACCAGTTCCCTGAAATTATTCCCCTACTTGACTAATAAGTATATA
GAATGTGGAAGATAATGGAAGACGAGAGAGACTAAACCTTTTTCGACTTTTTTTTTCCAACTTTGGTCAAGGGACTTTAATAAGGGGATGAACTGATTATTCATATAT

GPD promoter

4,500 4,520 4,540 4,560 4,580 4,600

AAGACGGTAGGTATTGATTGTAATTCTGTAAATCTATTTCTTAAACTTCTTAAATTCTACTTTTATAGTTAGTCTTTTTTTTAGTTTTAAAACACCAGAACTTAGTT
TTCTGCCATCCATAACTAACATTAAGACATTTAGATAAAGAATTTGAAGAATTTAAGATGAAAATATCAATCAGAAAAAAAATCAAAATTTTGTGGTCTTGAATCAA

GPD promoter

4,620 4,640 4,660 4,680 4,700

TCGACGGATTCTAGAACTAGTGGATCCATGAGATTCCCATCTATCTTCACCGCTGTTTTGTTCGCTGCTTCTTCTGCTTTGGCTGCTCCAGCTAACACCACCACCGA
AGCTGCCTAAGATCTTGATCACCTAGGTACTCTAAGGGTAGATAGAAGTGGCGACAAAACAAGCGACGAAGAAGACGAAACCGACGAGGTCGATTGTGGTGGTGGCT

1 2 3 4 5 6 7 8 9 10 11 12 13 14 15 16 17 18 19 20 21 22 23 24 25 26 27
M R F P S I F T A V L F A A S S A L A A P A N T T T E

GPD promoter Mating factor alpha leader

4,720 4,740 4,760 4,780 4,800

AGACGAAACCGCTCAAATCCCAGCTGAAGCTGTTATCGACTACTCTGACTTGGAAGGTGACTTCGACGCTGCTGCTTTGCCATTGTCTAACTCTACCAACAACGGTT
TCTGCTTTGGCGAGTTTAGGGTCGACTTCGACAATAGCTGATGAGACTGAACCTTCCACTGAAGCTGCGACGACGAAACGGTAACAGATTGAGATGGTTGTTGCCAA
28 29 30 31 32 33 34 35 36 37 38 39 40 41 42 43 44 45 46 47 48 49 50 51 52 53 54 55 56 57 58 59 60 61 62
D E T A Q I P A E A V I D Y S D L E G D F D A A A L P L S N S T N N G

Mating factor alpha leader

4,820 4,840 4,860 4,880 4,900 4,920

TGTCTTCTACCAACACCACCATCGCTTCTATCGCTGCTAAGGAAGAAGGTGTTCAATTGGACAAGAGAGAAagatctggcggccgcgcatgcgctagcctcgagCAA
ACAGAAGATGGTTGTGGTGGTAGCGAAGATAGCGACGATTCCTTCTTCCACAAGTTAACCTGTTCTCTCTTtctagaccgccggcgcgtacgcgatcggagctcGTT
63 64 65 66 67 68 69 70 71 72 73 74 75 76 77 78 79 80 81 82 83 84 85 86
L S S T N T T I A S I A A K E E G V Q L D K R E Q

Mating factor alpha leader

4,940 4,960 4,980 5,000 5,020

GTCCAGCTGGTCGAGTCAGGTGGTGGATCTGTCCAAGCCGGGGGGTCTCTAAGACTATCATGTACGGCGTCAGGCTATATTTCTTCTGACAAACCGATGTGGTTCAG
CAGGTCGACCAGCTCAGTCCACCACCTAGACAGGTTCGGCCCCCCAGAGATTCTGATAGTACATGCCGCAGTCCGATATAAAGAAGACTGTTTGGCTACACCAAGTC
2 4 6 8 10 12 14 16 18 20 22 24 26 28 30 32 34 36
V Q L V E S G G G S V Q A G G S L R L S C T A S G Y I S S D K P M W F R

Syn Nb_a

5,040 5,060 5,080 5,100 5,120

GCAAGCACCGGGCCAGGAAAGAGAGGCAGTTGCGGAATTTGTTGCCGCTATTAGTGACGGTAGTAATACCAATTATCGTTTTACTATTAGCAGAGACAACGCAAAAA
CGTTCGTGGCCCGGTCCTTTCTCTCCGTCAACGCCTTAAACAACGGCGATAATCACTGCCATCATTATGGTTAATAGCAAAATGATAATCGTCTCTGTTGCGTTTTT
38 40 42 44 46 48 50 52 54 56 58 60 62 64 66 68 70 72
Q A P G Q E R E A V A E F V A A I S D G S N T N Y R F T I S R D N A K

Syn Nb_a

5,140 5,160 5,180 5,200 5,220 5,240

ATACTGTTACGCTACAAATGAATAATCTGAAGCCAGAAGATACTGCGATCTATTATTGTGCCGCAGCGGTTCCGGGTCCGGTTGACGTTACTATCTCTATCAAATTT
TATGACAATGCGATGTTTACTTATTAGACTTCGGTCTTCTATGACGCTAGATAATAACACGGCGTCGCCAAGGCCCAGGCCAACTGCAATGATAGAGATAGTTTAAA
74 76 78 80 82 84 86 88 90 92 94 96 98 100 102 104 106 108
N T V T L Q M N N L K P E D T A I Y Y C A A A V P G P V D V T I S I K F

Syn Nb_a

5,260 5,280 5,300 5,320 5,340

GTTTATTGGGGACAAGGAACACAGGTCACAGTATCATCTGGCGGAGGAGGTTCCGGTGGAGGGGGGTCAGACTATAAGGATGACGACGATAAGGCTGGAGGTTCGAG
CAAATAACCCCTGTTCCTTGTGTCCAGTGTCATAGTAGACCGCCTCCTCCAAGGCCACCTCCCCCCAGTCTGATATTCCTACTGCTGCTATTCCGACCTCCAAGCTC
110 112 114 116 118 120 1 2 3 4 5 6 7 8 9 10 1 2 3 4 5 6 7 8 1 2 3 4 1
V Y W G Q G T Q V T V S S G G G G S G G G G S D Y K D D D D K A G G S R

Syn Nb_a FLAG tag

5,360 5,380 5,400 5,420 5,440

```
GGGAGTCCCTCACATAGTGATGGTGGATGCTTACAAAAGGTATAAATAACCCGGGCTGCAGGAATTCGATATCAAGCTTATCGATACCGTCGACCTCGAGTCATGTA
CCCTCAGGGAGTGTATCACTACCACCTACGAATGTTTTCCATATTTATTGGGCCCGACGTCCTTAAGCTATAGTTCGAATAGCTATGGCAGCTGGAGCTCAGTACAT
  2  3  4  5  6  7  8  9 10 11 12 13 14 15 16  1
  G  V  P  H  I  V  M  V  D  A  Y  K  R  Y  K  *
SpyTag
5,460  5,480  5,500  5,520  5,540  5,560

ATTAGTTATGTCACGCTTACGTTCACGCCCTCCCCCCACATCCGCTCTAACCGAAAAGGAAGGAGTTAGACAACCTGAAGTCTAGGTCCCTATTTATTTTTTTATAG
TAATCAATACAGTGCGAATGCAAGTGCGGGAGGGGGGTGTAGGCGAGATTGGCTTTTCCTTCCTCAATCTGTTGGACTTCAGATCCAGGGATAAATAAAAAAATATC
CYC1 terminator
5,580  5,600  5,620  5,640  5,660

TTATGTTAGTATTAAGAACGTTATTTATATTTCAAATTTTTCTTTTTTTTCTGTACAGACGCGTGTACGCATGTAACATTATACTGAAAACCTTGCTTGAGAAGGTT
AATACAATCATAATTCTTGCAATAAATATAAAGTTTAAAAAGAAAAAAAAGACATGTCTGCGCACATGCGTACATTGTAATATGACTTTTGGAACGAACTCTTCCAA
CYC1 terminator
5,680  5,700  5,720  5,740  5,760

TTGGGACGCTCGAAGGCTTTAATTTGCGCGCGCTCACTGGCCGTCGTTTTACAACGTCGTGACTGGGAAAACCCTGGCGTTACCCAACTTAATCGCCTTGCAGCACA
AACCCTGCGAGCTTCCGAAATTAAACGCGCGCGAGTGACCGGCAGCAAAATGTTGCAGCACTGACCCTTTTGGGACCGCAATGGGTTGAATTAGCGGAACGTCGTGT
CYC1 terminator    M13-fwd
5,780  5,800  5,820  5,840  5,860  5,880

TCCCCCTTTCGCCAGCTGGCGTAATAGCGAAGAGGCCCGCACCGATCGCCCTTCCCAACAGTTGCGCAGCCTGAATGGCGAATGGACGCGCCCTGTAGCGGCGCATT
AGGGGGAAAGCGGTCGACCGCATTATCGCTTCTCCGGGCGTGGCTAGCGGGAAGGGTTGTCAACGCGTCGGACTTACCGCTTACCTGCGCGGGACATCGCCGCGTAA
LacZ alpha
5,900  5,920  5,940  5,960  5,980

AAGCGCGGCGGGTGTGGTGGTTACGCGCAGCGTGACCACTACACTTGCCAGCGCCCTAGCGCCCGCTCCTTTCGCTTTCTTCCCTTCCTTTCTCGCCACGTTCGCCG
TTCGCGCCGCCCACACCACCAATGCGCGTCGCACTGGTGATGTGAACGGTCGCGGGATCGCGGGCGAGGAAAGCGAAAGAAGGGAAGGAAAGAGCGGTGCAAGCGGC
6,000  6,020  6,040  6,060  6,080

GCTTTCCCCGTCAAGCTCTAAATCGGGGGCTCCCTTTAGGGTTCCGATTTAGTGCTTTACGGCACCTCGACCCCAAAAAACTTGATTAGGGTGATGGTTCACGTAGT
CGAAAGGGGCAGTTCGAGATTTAGCCCCCGAGGGAAATCCCAAGGCTAAATCACGAAATGCCGTGGAGCTGGGGTTTTTTGAACTAATCCCACTACCAAGTGCATCA
6,100  6,120  6,140  6,160  6,180  6,200

GGGCCATCGCCCTGATAGACGGTTTTTCGCCCTTTGACGTTGGAGTCCACGTTCTTTAATAGTGGACTCTTGTTCCAAACTGGAACAACACTCAACCCTATCTCGGT
CCCGGTAGCGGGACTATCTGCCAAAAAGCGGGAAACTGCAACCTCAGGTGCAAGAAATTATCACCTGAGAACAAGGTTTGACCTTGTTGTGAGTTGGGATAGAGCCA
6,220  6,240  6,260  6,280  6,300

CTATTCTTTTGATTTATAAGGGATTTTGCCGATTTCGGCCTATTGGTTAAAAAATGAGCTGATTTAACAAAAATTTAACGCGAATTTTAACAAAATATTAACGCTTA
GATAAGAAAACTAAATATTCCCTAAAACGGCTAAAGCCGGATAACCAATTTTTTACTCGACTAAATTGTTTTTAAATTGCGCTTAAAATTGTTTTATAATTGCGAAT
6,320  6,340  6,360  6,380  6,400  6,420
```

```
CAATTTCCTGATGCGGTATTTTCTCCTTACGCATCTGTGCGGTATTTCACACCGCATAGGGTAATAACTGATATAATTAAATTGAAGCTCTAATTTGTGAGTTTAGT
GTTAAAGGACTACGCCATAAAAGAGGAATGCGTAGACACGCCATAAAGTGTGGCGTATCCCATTATTGACTATATTAATTTAACTTCGAGATTAAACACTCAAATCA
                   6,440               6,460               6,480               6,500               6,520

ATACATGCATTTACTTATAATACAGTTTTTTAAGCAAGGATTTTCTTAACTTCTTCGGCGACAGCATCACCGACTTCGGTGGTACTGTTGGAACCACCTAAATCACC
TATGTACGTAAATGAATATTATGTCAAAAAATTCGTTCCTAAAAGAATTGAAGAAGCCGCTGTCGTAGTGGCTGAAGCCACCATGACAACCTTGGTGGATTTAGTGG
                                    LEU2
            6,540               6,560               6,580               6,600               6,620

AGTTCTGATACCTGCATCCAAAACCTTTTTAACTGCATCTTCAATGGCCTTACCTTCTTCAGGCAAGTTCAATGACAATTTCAACATCATTGCAGCAGACAAGATAG
TCAAGACTATGGACGTAGGTTTTGGAAAAATTGACGTAGAAGTTACCGGAATGGAAGAAGTCCGTTCAAGTTACTGTTAAAGTTGTAGTAACGTCGTCTGTTCTATC
                                                   LEU2
     6,640               6,660               6,680               6,700               6,720               6,740

TGGCGATAGGGTTGACCTTATTCTTTGGCAAATCTGGAGCAGAACCGTGGCATGGTTCGTACAAACCAAATGCGGTGTTCTTGTCTGGCAAAGAGGCCAAGGACGCA
ACCGCTATCCCAACTGGAATAAGAAACCGTTTAGACCTCGTCTTGGCACCGTACCAAGCATGTTTGGTTTACGCCACAAGAACAGACCGTTTCTCCGGTTCCTGCGT
                                                   LEU2
                  6,760               6,780               6,800               6,820               6,840

GATGGCAACAAACCCAAGGAACCTGGGATAACGGAGGCTTCATCGGAGATGATATCACCAAACATGTTGCTGGTGATTATAATACCATTTAGGTGGGTTGGGTTCTT
CTACCGTTGTTTGGGTTCCTTGGACCCTATTGCCTCCGAAGTAGCCTCTACTATAGTGGTTTGTACAACGACCACTAATATTATGGTAAATCCACCCAACCCAAGAA
                                                   LEU2
          6,860               6,880               6,900               6,920               6,940

AACTAGGATCATGGCGGCAGAATCAATCAATTGATGTTGAACCTTCAATGTAGGGAATTCGTTCTTGATGGTTTCCTCCACAGTTTTTCTCCATAATCTTGAAGAGG
TTGATCCTAGTACCGCCGTCTTAGTTAGTTAACTACAACTTGGAAGTTACATCCCTTAAGCAAGAACTACCAAAGGAGGTGTCAAAAAGAGGTATTAGAACTTCTCC
                                                   LEU2
    6,960               6,980               7,000               7,020               7,040               7,060

CCAAAACATTAGCTTTATCCAAGGACCAAATAGGCAAT
GGTTTTGTAATCGAAATAGGTTCCTGGTTTATCCGTTA
                     LEU2
      7,070    7,080     7,090   7,100
```
